# The design and development of a multicentric protocol to investigate the impact of adjunctive doxycycline on the management of peripheral lymphoedema caused by lymphatic filariasis and podoconiosis

**DOI:** 10.1186/s13071-020-04024-2

**Published:** 2020-03-30

**Authors:** John Horton, Ute Klarmann-Schulz, Mariana Stephens, Philip J. Budge, Yaya Coulibaly, Alex Debrah, Linda Batsa Debrah, Suma Krishnasastry, Upendo Mwingira, Abdallah Ngenya, Samuel Wanji, Mirani Weerasooriya, Channa Yahathugoda, Inge Kroidl, Drew Deathe, Andrew Majewski, Sarah Sullivan, Charles Mackenzie, Thomas B. Nutman, Joseph P. Shott, Gary Weil, Eric Ottesen, Achim Hoerauf

**Affiliations:** 1 Tropical Projects, 24 The Paddock, Hitchin, UK; 2 Institute for Medical Microbiology, Immunology and Parasitology (IMMIP), German Centre for Infection Research (DZIF), Bonn-Cologne Site, University Hospital Bonn, Venusberg-Campus 1, 53105 Bonn, Germany; 3 Neglected Tropical Diseases Support Center, Task Force for Global Health, Decatur, GA USA; 4 Washington University School of Medicine, St. Louis, MO USA; 5 Filariasis Research Unit, International Center for Excellence in Research, ICER-Mali, Bamako, Mali; 6 Faculty of Allied Health Sciences, Kwame Nkrumah University of Science and Technology (KNUST), Kumasi, Ghana; 7 Department of Microbiology, Kwame Nkrumah University of Science and Technology (KNUST), Kumasi, Ghana; 8 Filariasis Research Unit, Govt. T D Medical College, Kerala, 699005 India; 9 National Institute for Medical Research, Dar es Salaam, Tanzania; 10 Department of Microbiology and Parasitology, University of Buea, Buea, SW State Cameroon; 11 Filariasis Research Training and Services Unit (FRTSU), Faculty of Medicine, University of Ruhuna, Galle, Sri Lanka; 12 Division of Infectious Diseases and Tropical Medicine, University Hospital of the University of Munich (LMU), Munich, Germany; 13 Laboratory of Parasitic Diseases, National Institute of Allergy and Infectious Diseases, National Institutes of Health, Bethesda, MD 20892 USA; 14 Division of Neglected Tropical Diseases, US Agency for International Development, 1300 Pennsylvania Ave NW, Washington, DC USA

**Keywords:** Lymphatic filariasis, Podoconiosis, Doxycycline, Hygiene, Lymphoedema, Clinical trial, Morbidity management

## Abstract

**Background:**

As new lymphatic filariasis infections are eliminated through mass chemotherapy, previously affected individuals are left with the sequellae, especially chronic progressive lymphoedema. Currently this is managed by careful attention to limb hygiene to prevent infection. Studies over the past 15 years have suggested that the incorporation of doxycycline treatment may arrest or even reverse progression of lymphoedema. Most of this work has been observational or based on small studies, and if this intervention is effective, studies need to be conducted on a larger scale and under diverse geographical and social conditions before it can be incorporated into treatment policy.

**Methods/Design:**

The double-blind, placebo-controlled study was designed to investigate the impact of six weeks treatment with doxycycline added to standard limb hygiene on early stage filarial lymphoedema in five sites in Africa and the Indian subcontinent. One site in Cameroon is selected for studying lymphoedema in podoconiosis. Each site was individually powered with the potential to undertake a meta-analysis on completion. Evaluation methods followed those used in Ghana in 2012 with additions resulting from advances in technology. The details of the core protocol and how it was varied to take account of differing situations at each of the sites are provided. The study will enrol up to 1800 patients and will complete in mid-2021.

**Conclusions:**

This paper provides details of what challenges were faced during its development and discusses the issues and how they were resolved. In particular, the reasons for inclusion of new technology and the problems encountered with the supply of drugs for the studies are described in detail. By making these details available, it is hoped that the study protocol will help others interested in improving treatment for filarial lymphoedema in the design of future studies.

*Trial registration* India: Clintrials.gov. NCT02929121 registered 10 Oct 2016: https://clinicaltrials.gov/ct2/show/NCT02929121

Mali: Clintrials.gov. NCT02927496 registered 7 Oct 2016: https://clinicaltrials.gov/ct2/show/NCT0292749

Sri Lanka: Clintrials.gov. NCT02929134 registered 10 Oct 2016: https://clinicaltrials.gov/ct2/show/NCT02929134

Ghana: ISRCTN. 14042737 registered 10 July 2017: 10.1186/ISRCTN14042737

Tanzania: ISRCTN. 65756724 registered 21 July 2017: 10.1186/ISRCTN65756724

Cameroon: ISRCTN. 1181662 registered 25 July 2017: 10.1186/ISRCTN11881662
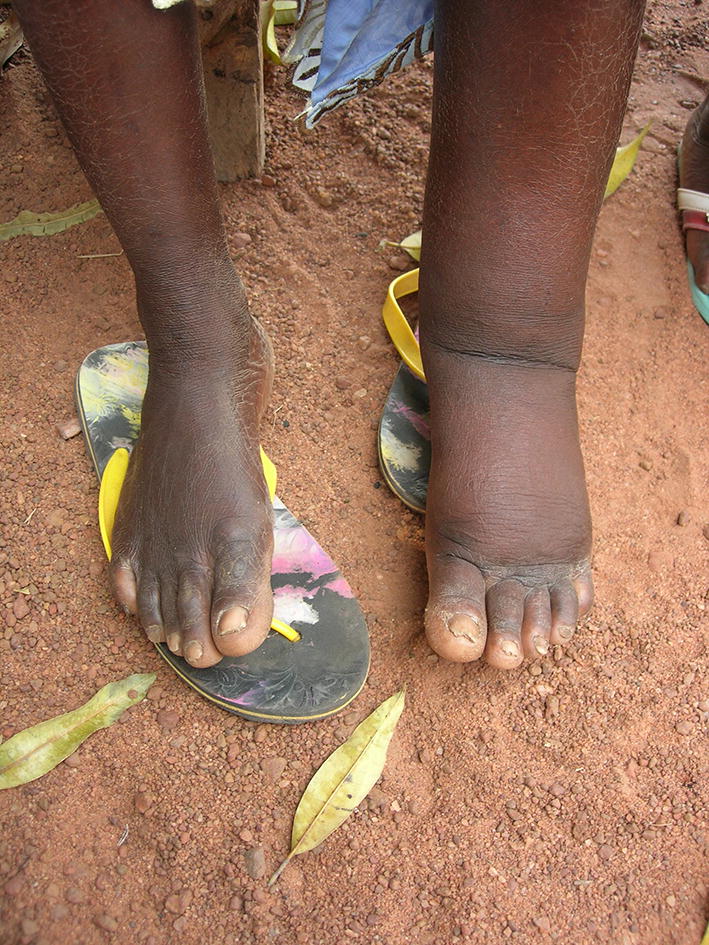

## Background

In the late 20th century lymphatic filariasis, caused by infections with either *Wuchereria bancrofti*, *Brugia malayi* or *B. timori*, was causing significant morbidity in approximately 20–40 million people globally, with a further 100 million infected but clinically asymptomatic, and up to one billion at risk of infection [1]. The development of annual combination treatment with albendazole together with either ivermectin or diethylcarbamazine and their deployment through mass community treatment under the Global Programme for the Elimination of Lymphatic Filariasis (GPELF) in all endemic areas has meant that, some 20 years later, transmission has ceased in many areas [2]. Over the coming decade it is expected that the infection will be eliminated in most parts of the world. However, one of the sequelae of the infection is chronic lymphatic damage, and thus those who have chronic manifestations will continue to suffer the consequences long after elimination of transmission and infection. Because of recurrent bacterial infections in the affected limbs, lymphatic damage becomes progressively worse, leading to the classical picture of elephantiasis. This can be controlled to some extent by assiduous attention to limb hygiene and use of topical and oral antibiotics to control acute episodes of infection. Limb hygiene should be taught to all affected persons as part of the elimination programmes but only about 35% of affected patients are actively applying this approach [3, 4].

In the search for effective medicines that kill the adult worms (macrofilaricides), there has been considerable interest in the potential of doxycycline and related antibiotics to kill the endosymbiotic bacterium, *Wolbachia*, which is essential for the survival and reproduction of the adult worms. In several studies, it was observed that both actively infected patients with lymphoedema and patients who had extinct infections appeared to benefit from six weeks of doxycycline treatment [5, 6]. A small prospective study was therefore undertaken in Ghana by Mand and colleagues [7] which compared patients with lymphoedema treated with either doxycycline, amoxicillin or placebo and followed the progress of their lymphoedema over a 24-month period after treatment. While there was a measurable improvement in the assessments in doxycycline-treated patients, those treated with either amoxicillin or placebo showed progression. This outcome was of considerable interest, since it suggested that active hygiene measures combined with doxycycline could provide greater benefit to patients with established lymphoedema, thereby reducing the morbidity associated in those already affected. This could be through the antimicrobial effects of doxycycline reducing chronic infection or possibly through the additional anti-inflammatory effects of doxycycline [8]. Since most patients will have inactive disease, either because of MDA or the macrofilaria have died, an effect *via Wolbachia* killing seems unlikely.

The Mand study was small, with only 100 participants (40 in the doxycycline arm) completing all evaluations [7]. Changes to the advice on the management of lymphoedema subjects would require confirmation of these findings with larger numbers distributed more widely geographically and including patients with *Brugia* infections. This paper presents the design and implementation of a study to provide these larger numbers.

Following the publication of the Mand study, two separate groups, the University of Bonn (UBonn) who conducted the original study, and the Task Force for Global Health (TFGH) in Atlanta, USA, began to develop plans to repeat the study with larger sample sizes to be conducted in several different geographical locations. Both groups initially intended to conduct the studies using Mand protocol design but with larger numbers and a double blind, placebo-controlled design. The two groups separately sought funding for their plans; eventually UBonn secured funding as part of the TAKeOFF project (“Tackling the obstacles to fight filariasis and podoconiosis”) which is one out of five research networks for health innovations in sub-Saharan Africa funded by the German Federal Ministry of Education and Research (Bundesministerium für Bildung und Forschung - BMBF) (https://www.gesundheitsforschung-bmbf.de/en/research-networks-for-health-innovations-in-sub-saharan-africa-7694.php); TFGH obtained funding from the United States Agency for International Development (USAID) (grant # AID-OAA-G-14-00008). Whereas the TFGH funding was specific for the lymphoedema study, the BMBF grant covered a further eight work packages associated with the management of morbidity and lymphoedema. The two groups were initially independent but came together in 2015, agreeing to conduct all studies to a single core protocol to maximise the impact of the work. Six country centres were included: Mali, Sri Lanka and Kerala, India (LEDoxy sites) were included under the TFGH grant; and Ghana, Tanzania and Cameroon (TAKeOFF) were included under the BMBF grant.

The primary aim of the studies was to determine whether a six-week course of doxycycline treatment, when added to standardised hygiene intervention, would arrest or reverse the progress of lymphoedema caused by lymphatic filariasis or in the case of Cameroon, caused by podoconiosis. Podoconiosis is a non-infectious tropical lymphoedema caused by long-term barefoot exposure to red clay soil derived from volcanic rocks [9] which has many characteristics similar to lymphatic filariasis and has been historically confused with it [10]. The probable mechanism is that the mineral particles penetrate the bare skin are engulfed by macrophages in the lower limb lymphatics to induce inflammatory responses [10].

In designing the protocol, it was agreed at the onset that the patient inclusion and exclusion criteria and the methods of measurement should follow those used in the original Mand study, thus replicating the patient population and assessments as closely as possible. During development of the protocol and early implementation of the studies several issues arose, and these are discussed.

## Methods/Design

### Overall study design

The protocol was designed to be a placebo-controlled comparison of the efficacy of doxycycline as an adjunctive treatment for lymphoedema. All subjects were to receive training and implement the established regular hygiene measures used for the management of lymphoedema defined in the GPELF Management Guidelines [11] and in addition received daily doxycycline or placebo for six weeks. It is unknown whether there would be variations in efficacy between geographical areas with *W. bancrofti* infection or in the case of India with *B. malayi* infection. Similarly, there are no data on the efficacy of doxycycline in podoconiosis. It was therefore decided to conduct six studies, each separately powered, but using the same core protocol.

Lymphoedema in lymphatic filariasis and podoconiosis is progressive, with intermittent infection and increasing oedema and scarring, eventually resulting in permanent changes in the lower limbs. However, symptoms and signs in the early stages are more likely to be reversible. Therefore, the core protocol focused on patients with early lymphoedema classified as being Dreyer stages 1 through 3 [3] and the individual lymphatic filariasis studies enrolled patients in these categories. Additionally, it was decided that a small, unpowered investigation of the impact of intervention on lymphoedema stages 4–6 should be included since data were lacking with advanced disease stage. The podoconiosis study was limited to persons with stages 2 and 3 disease [12]. All patients within the study are followed for a total of 24 months.

### Trial setting

The six trial sites had a proven history of lymphatic filariasis or podoconiosis endemicity with a large population of patients with lymphoedema to facilitate recruitment. All sites were either rural villages or associated with small towns to facilitate treatment and follow-up. The locations of the study sites are provided in Table 1.

**Table 1:** Trial sites

Programme	Country	Study sites	Environment	Principal investigator	Based at
LEDoxy	Sri Lanka	Pohlena, Walgama and Madihe suburbs, Galle	Semi-urban	Professor Channa Yahathugoda	Faculty of Medicine, University of Rahuna, Galle
	Mali	Sikasso and Koulikoro	Rural	Dr Yaya I Coulibaly	International Center for Excellence in Research, (ICER-Mali), Bamako
	India	Ambalappuzha and Cherthala Yaluks, Alappuzha, Kerala	Rural/Semi-urban	Professor T K Suma	Filariasis Research Unit, Govt. T. D. Medical College Hospital Alappuzha, Kerala
TAKeOFF	Ghana	Upper East Region: Kassena-Nankana Eeast and West Districts	Rural	Professor Alexander Yaw Debrah	Kumasi Centre for Collaborative Research (KCCR), Kwame Nkrumah University of Science and Technology (KNUST), Kumasi
	Tanzania	Lindi Region, Pwani Region	Rural/Semi-urban	Dr Upendo Mwingira	NIMR, Dar es Salaam
	Cameroon	North West Region	Rural	Professor Samuel Wanji	Dept of Microbiology & Parasitology, University of Buea, SW State

### Trial supplies

#### Active and placebo doxycycline

Since this study was to be conducted double-blind, it was necessary to source active and placebo doxycycline and to package the drugs in an appropriate manner to facilitate treatment in rural settings. All elements of the supply, analysis, packaging and distribution are being undertaken by Piramal Healthcare Ltd, Morpeth, UK, a company with extensive experience in GMP production of clinical trial supplies. Piramal undertook an analysis of potential sources of active doxycycline formulations to determine the most appropriate manufacturer. Several different options were investigated, and doxycycline hydrochloride was rapidly discounted as an option as manufacturers with appropriate approvals was very limited. Examination of the alternate doxycycline hyclate suggested that both 100 mg tablets and capsules were available, but that tablets would be the easiest option to follow especially as the supplies would be used in tropical zones. Several different manufacturers in the European Union were identified, and ultimately Remedica, Limassol, Cyprus, was chosen as the source. This was based on the ability to supply and the company experience in producing approved products for use by different international organizations. Placebo manufacture was undertaken by Piramal Healthcare Ltd, since placebo was not available from the manufacturer of the active. In addition, Piramal undertook all packaging activity, producing both the blister beds and the secondary treatment packs in a GMP facility.

#### Blister treatment packs

The design of the blister packs had to accommodate 16 tablets and to have space for labelling of blisters according to the different country requirements. Additionally, because doxycycline is normally used at a lower dose (100 mg) in patients below 18 years of age or under 50 kg body weight, it was necessary for one blister strip to be removed for these patients. In two study sites (in Ghana and Tanzania) it was decided to also examine the efficacy of 100 mg doxycycline in a third arm since, if effective, this could ultimately reduce the cost and complexity of treatment. To maintain double-blind conditions, this approach required the packing of both active and placebo within a single blister bed with the ability to remove one set of tablets for those under 18 years or 50 kg body weight. The individual blister beds contained enough medication for one week together with additional tablets to allow for visit delays. The final design had two parallel blister beds separated by a perforation and a third area for the label. Treatment packs were made containing 6 sets of blisters for the treatment of one patient for 6 weeks. Treatments were produced for each study site according to separate pre-determined randomization lists for the main study group and for the advanced lymphoedema group.

#### Shipping supplies

Since antibiotics are relatively thermo-labile, all supplies were transferred to trial sites by air courier in 7-day temperature-controlled gel packs with temperature logging. The logs indicated that all supplies were transferred to site within the prescribed storage limits for the product. On receipt at the trial sites, the supplies were transferred to temperature-controlled storage until required.

### Trial governance

The studies are funded through TFGH by USAID and by the BMBF (TAKeOFF) and their conduct is overseen by two Coordinating Committees at the TFGH (Mali, Sri Lanka, India) and UBonn (Ghana, Tanzania, Cameroon) which collaborate and share information on a regular basis. Separate Data Safety Monitoring Committees (DSMCs) comprised of independent scientists are established for each of the two groups of studies in order to monitor the safety aspects of the studies, and the data are shared quarterly (TFGH) or biannually (TAKeOFF). In cases where SAEs occurred, the information is shared immediately and evaluated by the PI, Safety Officer and the DSMC.

For the TFGH trials, the core protocol was approved by the Western Institutional Review Board (Olympia, Washington USA) and additionally by each site’s local Ethics Committee and/or National Ethics Review Board. For the TAKeOFF trials, the protocols were approved by the local Institutional Review Boards as well as by the Ghana FDA and Ghana Health Service, the Tanzania FDA, the CNERSH, Yaoundé, Cameroon and additionally by the University of Bonn Ethics Committee and for Tanzania by the LMU Munich Ethics Committee. Ministry of Health/Ghana FDA/Tanzania FDA approval was obtained to permit import of drug supplies and other trial related materials. All study sites were individually registered on Clintrials.gov (Mali: NCT 02927496; Sri Lanka: NCT02929134; India: NCT 02929121) or ISRCTN (Ghana: 14042737; Tanzania: 65756724; Cameroon: 11881662) and, if required, with local clinical trial databases. Minor variations were required by the review boards before approval which did not impact on the overall core protocol. If appropriate and following consultation with the trial sites, some of these were incorporated as protocol amendments in the core protocol and approved by the relevant review boards. The protocols for the LEDoxy (Additional file 1: Text S1), TAKeOFF filariasis (Additional file 2: Text S2) and TAKeOFF podoconiosis (Additional file 3: Text S3) studies are provided for reference.

Compliance with Good Clinical Practices (GCP) for Sri Lanka, Mali and India has been ensured by clinical monitors from FHI 360 (Durham, NC, USA) with visits at initiation, approximately one year after study initiation and at study closure. In addition, visits are made by members from TFGH at intervals. Compliance with GCP at the TAKeOFF sites is provided by local monitors with visits at initiation followed by regular visits (biannually or annually) during the conduct of the trial. Additionally, the Tanzanian trial site has been inspected by the Tanzania FDA and inspections of the Ghanaian trial site by the Ghana FDA and the Cameroon site by the Cameroon National Ethics Committee are planned. A GCP workshop was organised by Ghana FDA and all scientists and clinicians working on the TAKeOFF Ghana project as well as those from Cameroon attended this workshop before starting recruitment of patients.

### Study populations

The study aim is to evaluate the impact of doxycycline adjunctive treatment on lymphoedema, and therefore all subjects were required to have clinically evaluable disease. As a result of GPELF activities, most eligible subjects at each site have been exposed to several annual rounds of treatment with either DEC plus albendazole or ivermectin plus albendazole and thus most did not have active lymphatic filarial infection. Lymphatic vessel damage occurs during infection but then evolves from an asymptomatic phase to overt and progressive lymphoedema over many years even in the absence of active infection. The evolution of lymphoedema is well described and an agreed staging is accepted [3]. For the purposes of this study it was considered that the early stages (1–3) would be most likely to respond to additional treatment since the changes are potentially reversible as underlying fibrosis and scarring is not advanced. The primary focus of recruitment was therefore to screen and enrol subjects in stages 1–3. As previously noted, subjects who presented with more advanced lymphoedema (stages 4–6) could be enrolled to a blinded but unpowered cohort (LEDoxy sites) or as participants of a pilot trial with *n* = 60 per trial site (Ghana and Tanzania).

Since tetracyclines including doxycycline are known to cause damage to skeletal structures and teeth during foetal development and through the first decade of life, all at-risk subjects were excluded from the study. Therefore, the minimal age for enrolment was set at 14 years, thus avoiding the potential risk of skeletal and dental damage. In practice this should not impact the outcomes of the studies since, although lymphatic damage can occur in the first decade, evaluable clinical symptoms and signs do not generally become apparent until the middle of the second decade. Similarly, care had to be taken to avoid exposure of pregnant women to doxycycline, and women of childbearing potential were screened for pregnancy at enrolment, during and on completion of treatment. Pregnancy tests were additionally conducted in the TAKeOFF sites during the follow-up period since pregnancy can alter fluid load and might affect the lymphoedema measures. All women of childbearing potential were advised to avoid pregnancy during treatment. The full list of inclusion and exclusion criteria are provided in Table 2. Each of the TFGH study sites was required to enrol 100 subjects with Stage 1–3 in each arm (placebo and doxycycline); in the two TAKeOFF lymphatic filariasis studies 120 subjects were enrolled in each of the three arms to allow for the comparison of three groups (placebo, low- and standard-dose doxycycline). Enrolment of subjects with stages 4–6 continued until the main study group (stages 1–3) was complete or until 50 subjects had been enrolled (LEDoxy sites). The TAKeOFF sites planned to enrol 60 patients per site to be in line with a sample size normally chosen for pilot-trials. The TAKeOFF podoconiosis study in Cameroon entered 100 subjects in each of the standard 200 mg doxycycline and placebo arms comparable to the LEDoxy trials in Sri Lanka, Mali and India. The Cameroon study did not have an unpowered arm.

**Table 2:** Inclusion and exclusion criteria

Study selection criteria	Definition
Clinical inclusion criteria	Age ≥ 14 years and ≤ 65 years
	Male or non-pregnant woman of childbearing-potential using an approved, effective method of contraception before, during and for at least 2 weeks after the completion of the active intervention with doxycycline or placebo
	Able to give informed consent/assent to participate in the trial
	Resident in an LF endemic area for five years (> 2 years for TAKeOFF)
	Body weight ≥ 40 kg
	Lymphoedema of at least one limb Stage 1-6 measured on a 7-point scale. (Stage 2 or 3 for podoconiosis)
	Ability to use established standardized methods of hygiene and effectively applying it prior to initiation of drug treatment
	No evidence of severe or systemic co-morbidities (except for features of filarial disease)
	Normal laboratory profile (type of investigations and normal limits in the case of haematological abnormalities are site-specific)
	Consent to storage of blood samples for further study (site specific)
	Negative pregnancy test
Clinical exclusion criteria	No lymphoedema or lymphoedema stage 7
	Age < 14 years or > 65 years
	Body weight < 40 kg
	Pregnant or breastfeeding women
	Women of childbearing potential not using an agreed method of contraception
	Clinical or laboratory evidence of hepatic or renal dysfunction or CNS disease
	Alcohol or drug abuse
	History of adverse reactions to doxycycline or other tetracyclines
	Patient has any situation or condition that may interfere with participation in the study as judged by the clinical investigator
	History of photosensitivity reactions after taking drugs
	Concomitant medication with antacids containing aluminium, magnesium or sucralfate and not able to discontinue
	Concomitant medication with other antibiotics than doxycycline and not able to discontinue
	Concomitant medication with diuretics or sulfonylurea or coumarin
Laboratory exclusion criteria	Haemoglobin < 8 g/dl
	Neutrophil count < 1100/mm^3^
	Platelet count < 100,000/mm^3^
	Creatinine > 2 times upper limit of normal
	AST (GOT), ALT (GPT), gamma-GT > 2 times upper limit of normal
	Positive urine pregnancy test

### Evaluation criteria and methods

The Mand study in Ghana had evaluated lymphoedema in several different ways, and it was decided that all of these should be included in the current studies. The primary efficacy measure was to be the change in clinical staging (improve, no change, deteriorate) as in the Mand study, and these are described in Table 3 [7]. A similar set of stages are established for podoconiosis lymphoedema (Table 4) [12]. Staging can be subject to individual interpretation by observers and therefore additional measures are included to provide support for the primary evaluation. Classically, lymphoedema has been evaluated at predefined anatomical points on the affected limb(s), and this approach of measuring limb circumference was retained for these studies. Clinical observation is also supported by digital photographs taken at the same time as the staging using a standardised methodology. Although producing standardised images is a problem under field conditions, it does provide evidence where discrepant observations are encountered.

**Table 3:** Staging of lymphatic filariasis lymphoedema (after Dreyer (2002) [3])

Stage	Definition
1	Swelling is reversible (goes away) overnight
2	Swelling is not reversible (does not go away)
3	Presence of shallow skin folds (base of fold can be seen with movement of leg)
4	Presence of skin knobs
5	Presence of deep skin folds (base of fold can only be seen if opened up)
6	Presence of “mossy lesions”. Warty looking epidermal skin lesions
7	Unable to care for self or perform daily activities

**Table 4:** Staging of podoconiosis lymphoedema (after Tekola et al. (2008) [12])

Stage	Definition
1	Swelling reversible overnight: the swelling is not present when the patient first gets up in the morning
2	Below-knee swelling that is not completely reversible overnight; if present, knobs/bumps are below the ankle ONLY. Persistent swelling that does not reach above the knee. If knobs or bumps are seen or felt, they are only present below the ankle, NOT above the ankle
3	Below-knee swelling that is not completely reversible overnight; knobs/bumps present above the ankle. Persistent swelling that does not reach above the knee. Knobs or bumps can be seen or felt above the ankle as well as below
4	Above-knee swelling that is not completely reversible overnight; knobs/bumps present at any location. Persistent swelling that is present above the knee. Knobs or bumps can be seen or felt at any place on the foot or leg
5	Joint fixation; swelling at any place in the foot or leg. The ankle or toe joints becomes fixed and difficult to flex or dorsiflex. This may be accompanied by apparent shortening of the toes

Advances in mobile ultrasonography has permitted the measurement of skin and subcutaneous tissue thickness over the median and lateral malleolus which provides additional evidence of oedema in the affected limb [7]. While this technology has the potential to provide objective evidence of change, in practice it has proved to be quite time-consuming to conduct, and difficult to standardise, especially in the field. Therefore, ultrasonography has been retained for the LEDoxy trials but not for the TAKeOFF trials.

In lymphoedema not only does the circumference of the limb increase but also the limb volume. The classical approach for measuring volume is the use of water displacement where the limb is put into a container of known volume and water added to a defined level on the limb (usually a bony prominence), the volume being calculated by subtraction. This is clearly a cumbersome, although reasonably accurate measure but does not lend itself to field use. As a result, this method was not used in the present study but was replaced by a more efficient volume assessment technique.

This new technique was the development of a photo-digital scanner (LymphaTech; Lymphatech.com) for limb volume measurement. The tool uses computerised infrared imaging captured on computer tablets to generate virtual 3D images of each limb that are used to calculate the limb volume indirectly. This technology was in its early stages of validation when the study protocol was designed, but by the time the study was ready to start, it was advanced enough to be added alongside the other, traditional measures. Details of the methodology and validation have been published [13], and user protocols have advanced during the study as field experience has increased. The developers have continued to work closely with study investigators for training and monitoring the course of the study. More importantly, this new approach allows rapid image capture to accurately measure volume in real time, being a considerable advance on the traditional measures described above and still retained for these studies.

It is strongly held that secondary bacterial and fungal infections are important contributory elements to the development of lymphoedema and dermal changes: the inclusion of regular washing of the affected limb(s) in the WHO recommended basic package of care [11] is founded on this understanding. As these WHO recommendations have been, appropriately, included in the present study, and as positive effects of washing on the clinical condition of the affected limbs are a potential confounding factor to the outcomes of our study, we have included an assessment of each patient’s adherence to washing as part of the study site procedures.

This assessment is divided into observations by the attendant care-giver as to (i) the cleanliness of the limb(s) (e.g. dirt on the skin, in skin folds, or on the toe nail bed); and (ii) a simple assessment of the condition of the skin of the limb (e.g. presence of open wounds, or cracks between the toes). Since items for washing (soap, bowls and towels, replenished as necessary) have been provided, each participant is asked to show that they still possess these items at each evaluation time. The presence of these items has been used previously as a useful surrogate for compliance with washing [4, 11].

### Study structure

For the LEDoxy sites all potential subjects are provided with information on the study design by study staff in the local languages and consent for screening (medical history, lymphoedema staging, laboratory tests and pregnancy tests for females) are obtained. Potential subjects return in 1–2 weeks and if they fulfil the inclusion and exclusion criteria, they are formally consented and enrolled in the study. For the TAKeOFF sites, participants consent for screening and treatment together prior to the first screening procedures. Additionally, TAKeOFF study participants are asked to give consent for storage, shipment and re-utilization of the samples (blood, urine, saliva) taken during the conduct of the trial. Prior to enrolment all subjects are provided with training on regular limb hygiene and given the necessary supplies; compliance is assessed throughout treatment and follow-up using a questionnaire specifically developed for the study and additional training given at intervals to reinforce compliance. Baseline measurements of lymphoedema and laboratory parameters are made prior to starting treatment. A quality of life (QoL) assessment is also performed using a simplified version of the 12-item Self-Reporting World Health Organization Disability Assessment Schedule (WHODAS) 2.0 [14, 15]. Additionally, in Cameroon depression is being assessed in patients with podoconiosis [16].

Treatments were randomized prior to the study start and subjects were entered sequentially to receive placebo or doxycycline 200 mg (or if appropriate the lower 100 mg dose) daily for six weeks. Participants are regularly monitored during the six-week treatment period to collect safety information, to conduct pregnancy tests in women of childbearing potential and to issue new drug supplies. The subjects are assessed for lymphoedema measurements at 6, 12, 18 and 24 months after starting treatment as indicated in Additional file 4: Table S1.

The process of conducting subject visits varies from site to site and depends on the nature of the site and the distance from the Principal Investigator’s primary base. This varies from subjects travelling to the base hospital as in India where the subjects generally live close to the hospital, through a mixture of home visits during treatment and hospital visits for lymphoedema assessments (Sri Lanka), to fully field based investigations in villages (Mali, Ghana and Tanzania) where the study sites can be many hours travel from the primary base. All sites have developed a locally appropriate means of follow-up for participants who fail to attend scheduled visits, so that loss to follow-up would be kept to a minimum over the two years of the study.

### Data collection

It was decided early during study development to aim to collect all data electronically using a laptop computer system that could transmit the data to data managers and subsequently to a central database. The REDCap tool (https://www.project-redcap.org/, REDCap Consortium Emory University, Atlanta, USA) that was chosen provides an intuitive interface for validated data entry, audit trails for tracking data manipulation and export, automated export procedures for download to common statistical packages and the ability to import data from external sources [17, 18]. Data entry screens were developed in English and, for Mali, in English and French. Because of the nature of some of the sites and the need to maintain primary records it was decided that data would be initially entered onto paper Case Record Forms (CRFs) and subsequently entered onto the REDCap database by double data entry in all centres other than Mali. However, laptops are the primary source of data repository for the LymphaTech scans, ultrasound scans and digital photographs which are directly downloaded. To ensure linkage between individual patient CRFs, laboratory data, LymphaTech and other acquired data are all identified by bar-coding or the individual number of the participants which are composed of the country code, village number and number of the individual.

### Statistical considerations

The study was designed to demonstrate that adjunctive doxycycline is superior to standardized hygiene measures. The main outcome of the study therefore assesses whether there is a difference between the doxycycline and placebo groups regarding the proportion of participants with initial stage 1–3 whose lymphoedema grade did not progress (their lymphoedema grade decreased or remained the same) at 24 months using a two-sided Fisher’s exact test at an α-level of 5%.

From the beginning, the data from the Mand study [7] were key in determining the numbers to be enrolled since no other evaluable data existed. In that study, progression of lymphedema occurred in 55% of the placebo (hygiene only) group compared to progression in 5% of the active (doxycycline plus hygiene) group. However, since in the present study a greater emphasis is being placed on a regular hygiene protocol together with frequent checks on adherence and proper application, it was expected that progression in a hygiene-only group might be less pronounced and a progression in 25% might be expected for those on placebo (plus hygiene). Additionally, we chose to individually power each site since the geographical and cultural circumstances in each site were different and variations could preclude combination of the data.

Utilizing these estimates and assumptions, we calculated that 70 participants per arm would be required to see a statistical difference between the two groups with a significance level of 5% and with 95% power. A drop-out rate of < 30% over the two years was expected, so that a population of 100 patients in each of the two arms provided an appropriate trial size for the 3 LEDoxy study sites and the TAKeOFF podoconiosis trial. In the TAKeOFF studies where two active intervention dosages were being evaluated, and with the same expected progression in the placebo arm, a larger number was required, resulting in 120 subjects per arm (subsequent design with a power of 95% for the comparison of doxycycline 200 mg *vs* placebo and a power of 81% for the comparison of doxycycline 100 mg *vs* placebo if the progression in the doxycycline 100 mg group is ≤ 8%), again allowing for 30% loss to follow-up.

In addition to assessing progression of lymphoedema grade at 24 months, models that incorporate methods for analysing repeated measures will be used to determine whether differences in the measurement endpoints exist between study groups over time. Study group and time will be the main effects of the models, and if necessary, study sites will be included as blocking factors to account for site differences. All possible baseline characteristics, e.g. sex, age, race, baseline clinical data, will be evaluated for consideration of covariates. Analyses will be structured to include assessments of differences between 12- 18- and 24-month outcomes within the repeated measures design.

Secondary endpoints will also be assessed to determine the impact of doxycycline treatment on other aspects of lymphoedema. In addition to assessing the lack of progression of lymphoedema at 24 months, the lack of progression at 12 and 18 months after treatment onset will also be assessed. In addition to the primary outcome, it is most important to evaluate the improvement of lymphoedema grades at the different time points (comparison of the proportion of decreased lymphoedema *vs* the ones that increased or did not change). This will also be additionally evaluated using methods for repeated measurements (as described above for the primary outcome). As a third possibility to evaluate the main outcome of lymphoedema stage changes, the three outcomes (improvement, no change or progression of lymphoedema stages) will be considered separately and analysed accordingly.

The change in the circumference of the affected limb from baseline as measured by both tape-measure and by the Lymphatech scanner, is a secondary outcome. The Lymphatech scanner data will also be used to assess the change in limb volume from baseline to follow-up. Data on acute dermatolymphadenitis (ADLA) attacks are collected every two months after treatment onset. Reduction in the frequency of ADLA attacks will be evaluated from baseline to follow-up. Changes in skin thickness at 12 and 24 months compared to baseline will also be assessed in the sites where ultrasound measurements of skin thickness were collected (LEDoxy sites).

## Discussion

Although the original Mand study design was available, development of this protocol required a considerable amount of time to ensure that the two segments (LEDoxy and TAKeOFF) were aligned in their thinking and also ensuring that all the potential sites were equipped and capable of conducting all the elements of the study. Despite all proposed study sites having considerable experience in clinical trials, only the investigators in Ghana had any prior experience with the tools to be used in this study. Thus, site visits and audits were necessary to ensure all the necessary facilities were present, not only at the base but also at proposed field sites, and that they could conduct the study to satisfy International Conference on Harmonization: Good Clinical Practices (ICH-GCP) requirements. One of the problems faced by the teams is the climatic and political instability of some locations, such that visits had to be delayed, often for a considerable amount of time. For example, the India study start was delayed because of severe flooding.

The original concept was to establish a protocol that would replicate the Mand study but with larger numbers. It was originally conceived as a multi-centre investigation with identical evaluations at each site, but it was soon apparent that the differences in the sites and the comparison of efficacy of high and low doxycycline regimens at two of the TAKeOFF sites would require each site to be individually powered. This resulted in essentially six separate protocols built around a common structure.

These double-blind studies required sourcing of appropriate active and placebo and took a considerable time. An initial source failed compliance testing when the materials were shipped by sea without any temperature protection and had to be destroyed. A second source was found that was only able to manufacture doxycycline, and the placebo had to be made by Piramal (UK). This had to be fitted into the production schedules of the company who obtained the necessary tablet punches to match the active. All the quality controls required for Good Manufacturing Practices (GMP) compliance were undertaken. As a result, production of enough material for the study took approximately one year before the final blister packing and labelling could be undertaken. Blister design also was an issue since the blister packs had to allow for different dose regimens and be able to be produced under GMP conditions.

There were significant delays in getting ethics approval for the studies in some countries and further delays in obtaining the necessary import licences for the supplies, without which the drugs could not be sent to the sites. In Ghana for instance, the site needed three ethical approvals from KNUST IRB, Ghana Health Service and Ghana FDA before the trial could commence. Synchronising the protocol from three different ethical bodies in the same country was a challenge. Frequent contact between the manufacturers and the countries was required to ensure that all documentation was in place ahead of air freighting of the drugs, with almost a year passing between supply to the first and the last sites.

The evaluation tools used for the measurement of lymphoedema at the time of initial protocol design were generally very simple but subject to investigator accuracy to make them reproducible. For example, the measurement of leg circumference two centimetres above or below the original measurement point could completely mask any real changes. Ultrasound measurement of skin thickness over the median and lateral malleolus of affected limbs was used in the original study and was included in the protocol. In practice it is very time consuming to get reproducible results and the method becomes a problem when large numbers of patients need to be evaluated. Therefore, this evaluation was removed from the TAKeOFF protocols. It was fortuitous that the LymphaTech technology became available during protocol development and appeared to offer significant benefits in speed of evaluation and elimination of investigator bias. After some discussion the method was incorporated into the protocol and the LymphaTech team has worked closely with the sites to train operators, developed and improve standard operating procedures and troubleshooting manuals. A study validating the LymphaTech scanner in Sri Lanka was done expressly for the purpose of examining the reliability of the tools to be used in LEDoxy and was funded by USAID under the umbrella of the LEDoxy grant [13].

## Conclusions

This paper describes the conceptualisation, initial design and development of a multi-site protocol to determine whether adjunctive doxycycline given for six weeks provides benefit over standard care hygiene in patients with lymphoedema caused by lymphatic filariasis or podoconiosis. There have been numerous hurdles to be overcome before the first patients could be enrolled in 2018 and the first efficacy data will not be available until early 2021. By providing the information on the protocols in use at an early stage we hope that those involved in the management of lymphoedema in endemic areas will begin to think about what else may be needed before a treatment, if successful, is incorporated into practice. If additional work is to be undertaken, then it is important that protocol development start well in advance to avoid the not inconsiderable delays that we have experienced in implementing the current work. We believe that the present design has the potential to answer the overall question and should provide detail on the efficacy of the intervention, the size of the benefit and evidence of the safety and tolerability of long-term doxycycline in areas where lymphatic filariasis and podoconiosis are endemic.

## Supplementary information

**Additional file 1: Text S1.** LEDoxy protocol.

**Additional file 2: Text S2.** TAKeOFF filariasis protocol.

**Additional file 3: Text S3.** TAKeOFF podoconiosis protocol.

**Additional file 4: Table S1.** Study schedule.

## Data Availability

All data are included within the body of the published article and its additional files.

## Abbreviations

ADLA: acute dermatolymphadenitis
BMBF: Bundesministerium für Bildung und Forschung
CNERSH: Comité National d’Ethique de la Recherche en Santé Humaine
CRFs: case record forms
DEC: diethylcarbamazine
FDA: Food and Drug Authority
GMP: Good Manufacturing Practice
GPELF: Global Programme for the Elimination of Lymphatic Filariasis
ICH-GCP/GCP: International Conference of Harmonization-Good Clinical Practices
IRB: Institutional Review Board
ISRCTN: International Standard Randomised Clinical Trial Number
KNUST: Kwame Nkrumah University of Science and Technology
LEDoxy: lymphoedema/doxycycline
QoL: quality of life
TAKeOFF: Tackling the Obstacles to Fight Filariasis and Podoconiosis
TFGH: Task Force for Global Health
UBonn: University of Bonn
USAID: United States Agency for International Development
WHO: World Health Organization
WHODAS: World Health Organization Disability Assessment Schedule
