# Supplementary material for: The design and development of a multicentric protocol to investigate the impact of adjunctive doxycycline on the management of peripheral lymphoedema caused by lymphatic filariasis and podoconiosis
Source: Parasit Vectors. 2020 Mar 30;13:155. doi: 10.1186/s13071-020-04024-2 (PMC7106687; doi:10.1186/s13071-020-04024-2)
Supplement: Supplementary file 1 — Additional file 1: Text S1. LEDoxy protocol. [file 13071_2020_4024_MOESM1_ESM.docx]

**Additional file 1: Text S1.** LEDoxy protocol.

LEDoxy Protocol as used in India, Mali and Sri Lanka

**1. Title**:

**A multi-center, double-blind, randomized, 24-month study, to compare the efficacy of doxycycline once daily for 6 weeks versus placebo in improving filarial lymphedema independent of active filarial infection**

**2a. Trial Registration**:

ClinicalTrial.gov

Country registries: India

**2b. World Health Organization Trial Registration Data Set**

| **DATA CATEGORY** | **INFORMATION** |
| --- | --- |
| Primary registry and trial identifying number | ClinicalTrials.gov  SRI LANKA: [NCT02929134](https://clinicaltrials.gov/ct2/show/NCT02929134?term=NCT02929134&rank=1)  INDIA: [NCT02929121](https://clinicaltrials.gov/ct2/show/NCT02929121?term=NCT02929121&rank=1)  MALI: NCT02927496 |
| Date of registration in primary registry | October 7, 2016 |
| Secondary identifying numbers | Country Registries:  Clinical Trial Registry of India : CTRI/2017/08/009312 |
| Source(s) of monetary or material support | United States Agency for International Development (USAID) |
| Primary sponsor | Eric Ottesen, M.D., Task Force for Global Health, Decatur, GA. USA |
| Secondary sponsor(s) | TBD |
| Contact for public queries | Mariana Stephens, Task Force for Global Health, Decatur, GA. USA |
| Contact for scientific queries | Eric Ottesen, M.D., Task Force for Global Health, Decatur, GA. USA |
| Public title | Doxycycline for the treatment of filarial lymphedema |
| Scientific title | A Multi-center, Double-blind, Randomized, 24-month study, to Compare the Efficacy of Doxycycline [once daily x 6 weeks] versus Placebo in improving Filarial Lymphedema (independent of active filarial infection) |
| Countries of recruitment | 3 |
| Health condition(s) or problem(s) studied | Lymphatic filariasis lymphedema |
| Intervention(s) | Active comparator:Doxycycline hyclate 200 mg per day x 6 weeks for patients >40 kg or 100 mg per day for patients <40 kg) Placebo comparator: matching tablets containing no active ingredients |
| Key inclusion and exclusion criteria | **Inclusion:**   - Ages eligible for study: ≥14 years and ≤ 65 years Sexes eligible for study: Both - Resident in endemic area for ≥ 5 years - Lymphedema of a limb Grade 1-6 - Able to appropriately use standardized methods of hygiene   **Exclusion**:   - Women who are pregnant or breastfeeding - Clinical or biologic evidence of hepatic or renal dysfunction or CNS disease - Alcohol or drug abuse - History of adverse reactions to doxycycline or other tetracyclines |
| Study type | Interventional Allocation: randomized Intervention model: parallel assignment Masking: double blind (subject, caregiver, investigator, outcomes assessor) Primary purpose: prevention Phase III |
| Date of first enrolment | Expected: October 1st, 2017 |
| Target sample size | 200 patients with Grades 1-3 lymphedema per study site (based on end point and duration) and up to 50 patients with grade 4-6 lymphedema/per study site. |
| Recruitment status | Not yet started |
| Primary outcome(s) | Change in lymphedema grade at 24 months compared to baseline |
| Key secondary outcomes | Change in number of acute attacks |

**3. Date and Version Identifier:** 17AUG2017

Version 1.8

**4: Sources and types of financial, material, and other support.**

Doxycycline hyclate 100mg tablets (Remycin-brand name) will be manufactured by Remedica Ltd., Limasol, Cyprus and the and matching placebo will be produced by Piramal Healthcare, Morpeth, United Kingdom. The treatment packs for the active and placebo tablets will be prepared by Piramal Healthcare, Morpeth, United Kingdom.

- United States Agency for International Development (USAID) is funding the run-in costs
- Funding for this trial covers
  - XX,
  - XX, …
- The design, management, analysis and reporting of the study are entirely independent of the manufacturers of doxycycline and placebo

**5a: Names, affiliations, and roles of protocol contributors.**

Dr John Horton, Tropical Projects, Hitchin, UK (Design Detail)

Dr V Kumaraswamy, Chennai, India (Overall Design)

Dr Eric Ottesen, Taskforce for Global Health, Decatur, GA (Advice)

Dr Achim Hoerauf, University of Bonn, Germany (Advice)

**5b: Name and contact information for the trial sponsor**

Trial Sponsor: USAID via The Task Force for Global Health
Sponsor’s Reference:
Contact name: Eric Ottesen, M.D.Address: 325 Swanton Way, Decatur, GA 30030
Telephone: 404-592-1434
Email: [eottesen@taskforce.org](mailto:eottesen@taskforce.org)

**5c: Role of study sponsor and funders, if any, in study design; collection, management, analysis, and interpretation of data; writing of the report; and the decision to submit the report for publication, including whether they will have ultimate authority over any of these activities.**

USAID has no role in the design of this study and will not have any role during its execution, analyses, interpretation of the data, or decision to submit results. A central trial steering committee including members representing each site will be responsible for the study design, data management and analysis, report writing and publication.

**5d: Composition, roles, and responsibilities of the coordinating centre, trial steering committee, endpoint adjudication committee, data management team, and other individuals or groups overseeing the trial, if applicable**

- **Trial Steering Committee (TSC)**

This committee is composed of the coordinator of the trial, the medical statistician, other persons involved in the management of the trial and the Principal Investigators of all sites. Its role is to review the trial process and make decisions on salient issues related to the conduct of the trial. In particular, it will check the production of expected deliverables and reports and will evaluate the final results of the trial. The TSC will meet regularly (once a year) during the conduct of the trial. Additional ad-hoc meetings will be convened during the course of the project as needed by the coordinator of the trial.

**6a: Description of research question and justification for undertaking the trial, including summary of relevant studies (published and unpublished) examining benefits and harms for each intervention.**

Lymphatic filariasis (LF), caused by infection with *Wuchereria bancrofti*, *Brugia malayi* and *Brugia timori* is a major neglected tropical disease (NTD) identified as one of WHO’s 17 NTDs, has strong links with poverty and is associated with significant clinical morbidity and social stigma. The common clinical manifestations of the disease are hydrocele and lymphedema. The Global Programme to Eliminate Lymphatic Filariasis (GPELF) was launched in 2000 following the adoption of the World Health Assembly Resolution (WHA 50.29) to eliminate the disease as a public health problem by 2020. The twin goals of the programme are: 1) to reduce microfilaremia levels using mass drug administration (MDA) and thereby interrupt transmission and 2) to provide morbidity management to alleviate suffering for those who already have disease. (Gyapong 2005)

Significant progress has been made since the inception of the programme. By 2013, MDA had been implemented in 60 of the 72 endemic countries and a cumulative 4.9 billion doses of the drugs (albendazole in combination with either ivermectin or diethylcarbamazine) to interrupt transmission had been distributed to 1 billion people (WER 2014). Prior to the start of GPELF it was estimated that there were 91.13 million with microfilaremia, 29.94 million cases of hydrocele and 17.66 million cases of lymphedema (Ramaiah 2014). A recent assessment of the progress and impact of the programme indicated that it prevented or cured 96.71 million LF cases (79.20 million microfilaria carriers, 18.73 million hydrocele cases and a minimum of 5.49 million lymphedema cases) resulting in a 59% reduction of initial LF levels. The greatest decrease (68%) was in the number of individuals with microfilaremia, largely due to the widespread adoption of MDA programmes (Ramaiah 2014).

The progress in reducing morbidity due to the disease has been less striking. In 2013, only 27 countries had reported morbidity management and disability prevention activities and 13 years after the programme was initiated an estimated 19.43-million hydrocele cases and 16.68 million lymphedema cases still remained. While surgery is the treatment of choice for hydrocele and significant reductions in the number of hydrocele cases can be expected with increased access to surgical treatment of hydrocele, no immediate solutions are available for reducing the number of cases of lymphedema. The framework within GPELF to monitor the implementation and effectiveness of improved hygiene measures within the community is still under development. Even if the goal of interruption of transmission of GPELF is achieved and the development of disease in subsequent generations is blocked, currently affected populations will face a lifetime of progressive disability.

Current treatment practices of lymphedema rely on decreasing the number of acute attacks by improving the hygiene of affected limbs, use of appropriate topical antibiotics and antifungals, exercise, elevation of the limb and use of footwear. While this treatment package has been shown to be effective in halting the progression of lymphedema, it requires sustained access to resources required for limb care and strict adherence to the prescribed procedures.

The anti-*Wolbachia* effects of doxycycline and its potential role in filarial infections have been well documented (Taylor, 2005, Turner 2006). Recent observations have suggested a potential role for lymphatic endothelium-derived VEGF-C and sVEGFR3 and other angiopoetic factors in the pathogenesis of lymphedema that could be lowered by doxycycline. (Debrah 2006, Coulibaly et al 2009). A six-week course of Doxycycline (200 mg daily) prevented progression of lymphedema in patients with active infection of *W. bancrofti* (Debrah 2006). More recently, in a trial in Ghana, a similar course of doxycycline decreased severity of mild to moderate lymphedema independent of active filarial infection (Mand 2012).

The effect of Doxycycline in reversing or stopping the progression of lymphedema of patients with stage 1-3, irrespective of their filarial infection’s being active, provides an opportunity to include the drug as a new tool in LF morbidity management programs. However, before recommendations can be made regarding the frequency of its usage or alternate dosing patterns the findings of the Ghana study need to be replicated in other settings. This multi-center trial is designed to confirm the findings of the lone study that documented the efficacy of doxycycline treatment in patients with stages 1-3 lymphedema, irrespective of active LF infection, as well people with higher grades of lymphedema.

**6b: Explanation for choice of comparators.**

Current lymphedema management protocols are based on the use of simple measures of hygiene (regular washing with soap and water, skin and nail care), use of topical antibiotics or antifungal agents, exercise and footwear. Previous controlled clinical trials and extensive field experience have shown the benefit of these measures in reducing the frequency of attacks of acute dermato-lymphangio-adenitis (ADLA) that drive the progression of lymphedema (Shenoy, 1999). In most endemic countries they now represent the available “standard of care” in the absence of any structured treatment programs for the management of lymphedema of LF.

In the present study, the progression of lymphedema in a group of patients who receive a six-week course of doxycycline will be compared with that of a group who receives doxycycline “look-alike” placebo tablets. However, both groups will be enrolled into a standardized “regimen of hygiene” described above. Thus, patients enrolled in the “placebo” group also will receive the current standard of care, and the placebo used in the study will help to identify the benefits of doxycycline on a background of simple hygiene measures. The regimens will be explained to all participants who will be trained to use established standardized methods of hygiene and be effectively applying it prior to the initiation of the drug treatment. In addition, patients will be retrained at every contact point (3, 6, 12 and 18 months). A common, generic SOP with handouts that describes methods and the training schedule will be used so that similar methods are employed across all sites.

**7. STUDY OBJECTIVES**

**Hypothesis to be tested:** Doxycycline is superior to Placebo for management of lymphedema in patients from LF-endemic areas

**Primary Objective:**

To evaluate the efficacy of a 6-week course of daily doxycycline on changes in the grades of lymphedema

**Secondary Objectives**

To evaluate the efficacy of a 6-week course of doxycycline in:

a) changes in the circumference of affected limbs

b) reducing the skin thickness of affected limbs

c) reducing the frequency of acute attacks of ADLA and

d) assessing the tolerability and safety of doxycycline given for 6 weeks.

**8: Description of trial design**

This (**LEDOXY**) trial is designed as a randomized, controlled, observer-, provider- and patient-blinded multicenter **superiority** trial with two parallel groups and a primary endpoint of change in grade of lymphedema at 24 months. The population will be stratified according to the Grade (Early Grade 1-3; Late Grade 4-6 or herewith referred as Group A and Group B, respectively). Randomization will be performed as block randomization within each center in blocks of (N=4-10) for each of the groups (Early and Late).

**9: Description of study settings**

The effect of a 6-week course doxycycline on lymphedema without active filarial infection has been demonstrated in a single setting in Africa (Ghana; Mand, S., et al. 2012). In order to expand the benefits of this observation, similar studies need to be carried out in other endemic settings. Lymphatic filariasis is endemic in many countries of Africa and Asia that are yet to implement morbidity management programmes. In addition, the components of the proposed hygiene package are likely to vary depending on the availability of material and human resources. The conduct of this trial as a multi-center study will not only allow the evaluation of the efficacy of the drug in a variety of settings but also facilitate its rapid adoption by the respective control programmes of endemic countries, if proven to be useful.

The study will be conducted at the following sites

|  | Country | Study site | Research Group |
| --- | --- | --- | --- |
| Africa | Mali | Sikasso & Koulikoro | ICER-Mali, Filariasis Research Unit |
| Asia | India | Ambalappuzha and Cherthala Yaluks of Alappuzha District, Kerala | Filariasis Research Unit, Govt., T.D.Medical College, Alappuzha, Kerala |
| Asia | Sri Lanka | Pohlena, Walgama and Madihe suburbs, Galle | FRTSU, Faculty of Medicine, Univ. of Ruhuna, Galle |

The choice of study sites in endemic countries has been made based on the availability of a) adequate numbers of patients with various grades of lymphedema and b) clinical trial teams familiar with lymphedema management procedures and past experience with similar trials. See Annex 1

**10: Inclusion and exclusion criteria for participants.**

All patients will provide written informed consent for screening before any study procedures are done.

**10.1 Inclusion criteria**

Patients eligible for the trial must comply with all of the following at **randomization**:

1. Age ≥ 14 years and <65 years, male or non-pregnant women of childbearing-potential using an approved, effective method of contraception before, during and for at least 2 weeks after the completion of the active intervention with doxycycline or placebo

2. Able to give informed consent to participate in the trial (forms to be attached)

3. Resident in endemic area for five years or more

4. Body weight >40 kg

5. Lymphedema of a limb Grade 1-6 measured on a 7-point scale. (Appendix 2 for explanation of the grading system (Dreyer G et al. 2002).

6. Ability to use established standardized methods of hygiene and effectively applying it prior to the initiation of the drug treatment

7. No evidence of severe or systemic comorbidities except for features of filarial disease

8. Normal laboratory profile (Appendix 3 investigations and the maximum or minimum limits in the case of hematological abnormalities – Site Specific)

9. Consent to storage of blood samples for study

**10.2 Exclusion criteria**

Patients are ineligible to participate in the trial, if they have any of the following:

1. No lymphedema or lymphedema stage 7

2. Age < 14 years or > 65 years

3. Body weight < 40 kg

4. Pregnant or breastfeeding women

5. Women of childbearing potential not using an agreed method of contraception. (A pregnancy test will be conducted as part of the screening process to exclude pregnancy and repeated at 3 and 8 weeks. In addition, women of childbearing potential will be counseled against pregnancy during the treatment period)

6. Clinical or laboratory evidence of hepatic or renal dysfunction or CNS disease

7. Alcohol or drug abuse

8. History of adverse reactions to doxycycline or other tetracyclines

9. Patient has any situation or condition that may interfere with participation in the study as judged by the clinical investigator

**10.3 Justification for Exclusion of Women and Children**

Pregnant Women

Pregnant and/or breastfeeding women will be excluded because doxycycline is contraindicated in pregnancy and during breastfeeding. However, non-pregnant women of childbearing-potential using an approved, effective method of contraception before, during and for at least 2 weeks after the completion of the active intervention with doxycycline or placebo are eligible for inclusion. Since investigation of pregnancy status and use of contraception is a culturally sensitive issue, the approach to be used and the appropriateness of contraception will be defined by the local IRBs.

Children

Those less than 14 years of age will not be able to participate because of potential effects on bone development and discoloration of teeth by doxycycline in children

**11a: Interventions**

All screened patients will be enrolled into a programme of hygiene (described below) and will be required to demonstrate ability to use established standardized methods of hygiene and effectively applying it prior to the initiation of the drug treatment. Eligible patients will be randomized to receive either daily doxycycline or placebo. Doxycycline hyclate 100mg tablets are produced as Remycin 100mg tablets by Remedica Ltd, Limasol, Cyprus and the placebo are produced by Piramal Heathcare, Morpeth, United Kingdom. The treatment packs will be prepared by Piramal Healthcare, Morpeth, United Kingdom.

Additionally, after un-blinding and data analysis, the placebo group will be offered doxycycline treatment if the intervention proves to be more effective in ameliorating LE.

**11 a. 1. Hygiene**

All patients will be initiated to a programme of cleaning of the affected limb based on the principles outlined in the booklet “New Hope” for persons with lymphedema (Dreyer et al.2002) (Annex 2). A generic SOP with handouts that describes methods and the training schedule will be utilized so that similar methods are employed across all sites. In addition, patients will be retrained at every contact point (3, 6, 12 and 18 months). This will be standardized by the investigators and will include the following:
 1) Cleaning of the affected limb daily with soap and water.
 2) Keeping the affected limb dry

3) Clipping the nails

4) Appropriate antibiotics for ADLA episodes

5) Applying antifungal ointment to webs of the toes, nails and sides of the feet every night

6) Elevation of the affected extremity

7) Limb exercises as instructed

8) Encouraging and monitoring the use of appropriate footwear

Each patient will receive soap, towels and plastic bowls for washing the limbs and a diary (Annex 4) for recording ADLA attacks.

**11. a. 2. Doxycycline and Placebo**

Both doxycycline and placebo will be administered under supervision (directly observed treatment) for 6 weeks. The first dose of doxycycline (two 100 mg tablets for those over 50kg body weight and one 100 mg tablets for those between 40 and 50 kg body weight) or placebo will be given after all the investigations have been completed and informed consent has been obtained and the patient has been initiated into the programme of basic hygiene (Appendix 5 and 6, Study Schedule). Patients will be encouraged to eat before swallowing the tablets whole with a glass of water. Vomited doses will be replaced.

Ideally, patients will be required to come every day to the closest community health center to take their drugs under supervision. Subjects living in villages at a distance from any health center will be treated by the community health care provider or the local caregiver living in this village who will be informed and trained in the possible adverse events related to the drug.

Since some study sites may be village based without a health centre, patients may be treated in their village and required to attend daily at a fixed meeting point. The trial clinician and the research team with the help of trained community health workers will normally administer the treatment. They should come to the clinic every week with the patient and collect the drugs for one week. The provider will keep a diary where he or she will mark down the time of intake of the drug. A health worker may make surprise checks at the patient’s residence by looking at the diary and also by counting the remaining drugs to estimate compliance.

**11b: Criteria for discontinuing or modifying allocated interventions**

An individual subject will be withdrawn for any of the following:

- An individual subject’s decision.
- Any clinical AE, laboratory abnormality or other medical condition or situation such that continued participation in the study would not be in the best interest of the subject. Subjects will be followed for the duration of the study for indicated safety assessments.
- Non-compliance with study procedures to the extent that it is potentially harmful to the subject or to the integrity of the study data.
- A change in the subject’s baseline condition after enrollment so that the subject no longer meets the following inclusion/exclusion criterion.
  - Inclusion Criterion
    - Not pregnant or breastfeeding during the first six-weeks of the study
- The Investigator should attempt to determine the reason for the subject’s decision. There will be no disadvantage for the participant as a result of a withdrawal. If a participant does not return for a scheduled visit, every effort should be made to contact the participant. The investigator should inquire about the reason for withdrawal and the participant should be followed-up regarding any unresolved adverse events, if possible. In any circumstance, every effort should be made to document the participants’ outcome. Therefore, all participants, even if the participant was withdrawn from the trial treatment, will be encouraged to come for the follow-up visits.

**11c: Strategies to improve adherence and compliance**

Both doxycycline and placebo will be administered under supervision throughout the treatment period (directly observed treatment).

Patients will be expected to come every day to the closest community health center to take the drugs. Subjects living in villages at a distance from any health center will be treated by the community health care provider or the local caregiver living in this village who will be informed and trained in the possible adverse events related to the drug and its management.

All patients will be counseled at the time of the initial dose and during the treatment period. Key messages will include:

- - The importance of following study guidelines for adherence
  - Instructions about taking study pills whole, and what to do in the event of a missed dose.
  - Reinforcement that study pills may be doxycycline or placebo
  - Emphasize that all participants are expected to benefit from the hygiene intervention.
  - Importance of contacting the clinic if experiencing problems possibly related to study product
  - Patients will be questioned about problems with the drugs and motivated to complete treatment as planned
  - In addition, patients will be visited once every two months in the follow-up period to record the occurrence of ADLA attacks and reinforce the hygiene routine.
  - If ADL is present, return within one month, refer to SOP.

**11d: Relevant concomitant care and interventions that are permitted or prohibited during the trial**

A. Diet: There is no dietary restriction and participants will be encouraged to eat within one hour prior to drug intake.

B. Concurrent Medications: During the study period and 24 months of follow-up patients should not receive antifilarial drugs (except during MDA). Short courses of antibiotics are permitted for the treatment of ADLA attacks and infections such as UTI urinary tract infection or URTI –Upper Respiratory Tract Infection. Intake of all drugs other than study drugs will be documented on the diary cards (Annex 3) and transferred to the appropriate section of the case record forms (CRFs).

**12: Primary, secondary, and other outcomes (Details will be defined in Data Analysis Plan)**

Primary efficacy endpoint:

- Improvement or halt of progression (Lack of progression) of LE when examined 24 months after treatment onset

Secondary endpoint(s):

- Improvement or halt of progression (Lack of progression) of LE when examined 12 months after treatment onset
- Improvement of LE when examined 12 and 24 months after treatment onset
- Change of LE stages (reduction or increase) compared to baseline assessed at 12 and 24 months
- Change in the circumference of the affected limb from baseline
- Change in circumference through Lymphatech measurements compared to baseline
- Change in volume through Lymphatech scanning measurements compared to baseline
- Reduction in the frequency of acute ADLA attacks evaluated from 0-12 months and from 12-24 months after treatment onset
- Changes in skin thickness at 12 and 24 months compared to the baseline
- Change in angiogenic, pro-fibrotic or pro-inflammatory biomarkers at 12 and 24 months following doxycycline administration (at sites where these tests are done)

**13:  Time schedule of enrolment, interventions (including any run-ins and washouts), assessments, and visits for participants.**

Appendix 6 (Study Schedule) summarizes the procedures to be carried out at each visit.

**13.1** **Screening (Month -1)**

Health workers or other members of the research team will identify potential subjects. The following assessments / procedures will be performed in the month prior to enrollment:

- Informed consent for clinical trial participation, screening procedures, photographs of limbs, and biobanking
- Demographic data
- Complete medical history
- Physical examination and Vital signs
- History of lymphedema
- History of ADLA
- History of relevant (MDA) and concomitant medications
- Assessment and grading of lymphedema (if present)
- Assessment for the presence of hydrocele
- Complete blood count (CBC and Full Chem Screen)
- Filariasis Test Strip (FTS-Alere, Scarborough, ME) and Microfilaremia slide test on each participant, per the discretion of each country
- Saliva collection
- 10 ml of blood will be collected by venipuncture for hematological, biochemical tests (renal function, liver function tests), immunological tests (including filarial antigen), lymphopoietic and lymphangiogenic (VEGF), as directed.
- Liver Function Tests (AST/ALT/GGT)
- Serum Creatinine/BUN
- Serum or urine pregnancy test (for women of childbearing potential). (A pregnancy test will be conducted as part of the screening process to exclude pregnancy and again at 3 weeks and 3, 6, 12, 24 months. In addition, women of childbearing potential will be counseled on avoiding pregnancy during the treatment period in a culturally appropriate manner as defined by the local IRB)

**13.2** **Month 0 Day 0**

Baseline assessments will include

- Medical history: This will include medication history, practices related to lymphatic filariasis, past surgeries and family history of other illnesses
- Physical examination:
  - Vital signs
  - Recording of details of the lymphedema and assignment of grade
  - Measurement of 3 point limb circumference following the SOP Exclusion of other potential causes of lymphedema
- List of concomitant medications
- All women of childbearing age will have a urine pregnancy HCG test before participating in the study.
- ~ 10 ml of blood will be collected by venipuncture for hematological, biochemical tests (renal function, liver function tests), immunological tests (including filarial antigen), lymphopoietic and lymphangiogenic.
- Ultrasound measurements of the affected limbs and the normal limbs will be performed, specifically examining the thickness of the skin at specific sites (e.g. lateral malleolus).
- Measurement of volume from each limb by Lymphatech scanner
- A clinical photograph of the affected and normal limbs will be taken.
- ADLA survey will be conducted by a trained social worker / health worker
- A QOL questionnaire will be administered by a trained social worker / health worker
- Hygiene status will be collected by a trained social worker/ health worker
- Lymphedema management training will be provided by a trained social worker / health worker
- Subjects will be randomized to receive doxycycline or placebo.

**13.3 Days 1-42 (treatment phase)**

- Informed consent to participate in the trial will be obtained prior to drug administration.
- Participants will be assigned to Group A or B based on Lymphedema grade.
- Participants will receive the first dose of either doxycycline or placebo, according to body weight, under supervision of the investigators (or their designees) at the study sites.
- Subsequent doses will be administered under supervision using one of the following methods:
  - Subjects will be expected to come every day to the closest community health center to take the drugs under supervision
  - Subjects living in villages quite far from any health center will be treated by the community health care provider or the local caregiver living in this village (community health workers) who will be informed and trained in the possible adverse events of the study agents
  - Subjects living close to the study center will be encouraged to visit the center every day to receive their drugs
- AE recording will be performed at all sites where drugs are distributed.
- A bi-weekly supervision will be conducted and details of any episode of ADLA since the last visit (or ongoing) will recorded.
- After 3 weeks (Day 22) into treatment serum transaminases will be measured
- On day 1 before the first dose of treatment and every three weeks into treatment a urine pregnancy test will be performed on women of childbearing age.
- History of concomitant medication will be collected, if applicable.

**13.4 Follow-up**

A. Follow-up examinations will be conducted at months 3, 6, 12, and 24 following drug administration (day 42-49); all included patients will be reassessed.

Assessments will include:

- Clinical examination & vital signs
  - Lymphedema staging will be conducted on both limbs
  - Measurement of lymphedema will occur on both limbs
  - Ultrasound examination of skin thickness at the lateral malleolus on both limbs
  - Clinical photographs of both limbs following the SOP
  - Measurement of limb circumference and limb volume from each limb by Lymphatech scanner
  - Collection of blood samples for estimation of lymphopoietic and lymphangiogenic factors (at sites where facilities exist for such measurements)
  - Collections of urine (women only)
  - Collection of saliva
  - Pregnancy test will be conducted at each time point.
  - Assessment of compliance to hygiene measures during the treatment phase and during the post-treatment visits to record ADLA using data from the diary cards
  - History of concomitant medication

B. Subjects will be visited every 2 months during the follow up period to assess occurrence of ADLA. During the visit the health worker will record the entries made in the diaries and question the patients about the duration and treatment received for any episodes since the last visit.

C. Additional evaluations at 12 and 24 months

- Administration of QOL questionnaire Survey

**13.5 Study Procedures/Evaluations**

**13.5.1 Clinical Evaluation**

**13.5.1.1 History and Physical Examination**

A complete medical history and physical examination will be performed as part of the baseline evaluation. Subsequent clinical evaluations will focus on the assessment of new symptoms, signs or untoward medical events. Vital signs, including blood pressure, heart rate, and body temperature will be measured as part of all physical examinations, according to standard nursing practice.

**13.5.1.2 Staging of lymphedema**

. Staging of LE will be done using the 7 stage classification described by Dreyer et al. [3]. The following procedure for comparisons of LE staging in patients with either one or both legs affected will be used: (a) if only one leg has LE, this leg will be analyzed (b) if both legs are affected, one leg with stage 1-3 and the other leg with stage 4-7, the leg with the lower stage will be chosen for analysis   (c) if both legs are affected with stage 1-3, the leg with the higher stage will be chosen for analysis   (d) if both legs are affected  with stage 4-6, the leg with the lower stage will be chosen for analysis    (e) if both legs are affected equally (same stage) one of them will be chosen randomly for analysis. For secondary analysis, all legs should be analyzed.

**13.5.1.3 Circumference Measurement of Legs**

Leg circumferences will be measured using a tape measure. Circumference measurements will be made at 10 cm posterior to the tip of the large toe and 12, 20, and 30 cm from the sole of the foot, at least twice as described elsewhere [Appendix 7]. Averages of the 4 measurements will be determined before treatment and at follow-up.

**13.5.1.3 Training for care and hygiene of affected legs and arms**

All patients included in this study as well as the local health care providers will receive training on the measures of hygiene that will be followed by patients. Each patient will receive soap, towels, and plastic bowls for washing the limbs. Supplies will be replenished during the follow-up visits, if necessary. The regimens will be explained to all participants who will be trained to use established standardized methods of hygiene and be effectively applying it prior to the initiation of the drug treatment. In addition, patients will be retrained at every contact point (3, 6, 12 and 18 months). A generic SOP with handouts that describe methods and the training schedule will be developed so that similar methods are used across all sites.

All patients will be initiated to a programme of cleaning of the affected limb based on the principles outlined in the booklet “New Hope” for persons with lymphedema. This would be standardized by the investigator and will include the following:
 1) Cleaning of the affected limb every night with soap and water
 2) Keeping the affected limb dry

3) Clipping the nails

4) Appropriate antibiotics for ADLA episodes

5) Applying antifungal ointment to webs of the toes, nails and sides of the feet every night

6) Elevation of the affected extremity

7) Limb exercises as instructed

8) Encouraging and monitoring the use of appropriate footwear

**13.5.1.3 Ultrasound and Lymphatech Scanner**

Each site will develop SOPs for measuring ‘skin thickness’ (using ultrasound) that will be site-specific and consistent for any one site. Briefly, ultrasonography of the legs to assess lymphedema will be performed as described by Mand et al (2012). Patients will be examined between 2 and 7 PM. Repeat scans should be conducted within a 2 hour window +/- 1 from every visit to ensure consistency throughout the trial. Using a portable hand-carried ultrasound system equipped with a 38-mm 5–10-MHz linear-array transducer (Philips Lumify Probe, USA), patients will be scanned sitting with stretched legs and feet perpendicular to the legs. The transducer will be positioned on the malleolus (ankle) and kept at a 90° angle to the skin surface in transverse sections. The head of tibia or fibula should be visible, and the malleolus should appear as a sharp line to permit reproducibility. The thickness of the tissue (subcutis, dermis, and epidermis) will be measured from the malleolus to the skin surface. Lateral and medial malleoli of both legs will be measured before treatment and at follow-up. The Lymphatech scanning tool which was developed to measure volume differences through infra-red light emitting scanning technology. The use of the scanner should follow the SOP, occur immediately before the Ultrasound tool and within the same time reference.

Lymphatech Scanner Mechanism: (Patent Source: <https://www.google.com/patents/US20160235354> )

The scanner is designed to capture 3D image of patient body parts creating an electronic avatar that permits precise calculation of the volume of specific body parts. Unlike structured light imaging devices (e.g., MRI, ultrasound, or X-rays), this simple-to-use imaging device uses a portion of the normal light wavelength spectrum, specifically infra-red (INR) emitting light via the Microsoft® Kinect® 2nd Generation to measure the time of flight (“ToF”) reaching an object by INR light waves (Figure 1 & 2). Planted between 3 to 8 feet away from the stationary patient, this device records patient images from front, back, left and right side positioning, as opposed to the full patient body penetrating images displayed by prior imaging methods mentioned previously. These patient images each, independently, comprise from about 20 to 30 high resolution images serially taken along the length of the patient's arm or leg and creates a real-time imaging true to life, 3D anatomical representation that can be used to compute information on patient limb circumference or volume. When generated longitudinally, the scanner creates high depth resolution and more consistency in measurement data.

The technology makes it an ideal tool to monitor lymphedema progression or regression on patients as the aim is to determine a change of participant’s body part volume over time (baseline versus subsequent times). It is also particularly suitable for allowing a patient to be imaged in extra-clinical settings, such as the patient's home or other setting where medical imaging equipment and personnel are not generally available.

Figure 1:


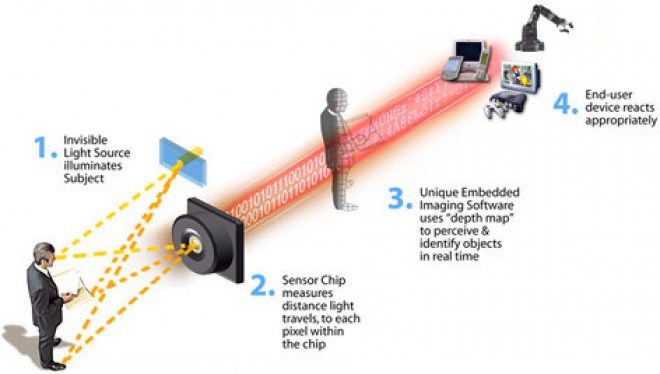


Figure 2:


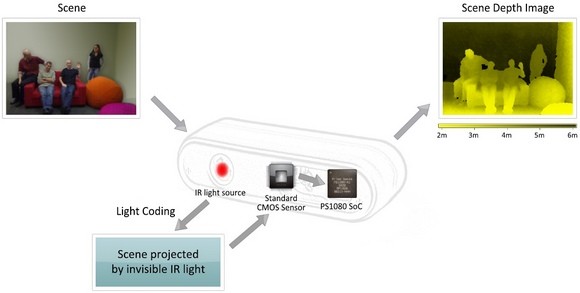


**13.5.2 Laboratory Evaluations**

**13.5.2.1** **Hematology and biochemistry**

At baseline and assigned time points (6, 12, & 24 months), venipuncture will be performed for assessing the leukocyte counts and eosinophil counts, hemoglobin levels, transaminases, creatinine, bilirubin, and urine test as applicable for pregnancy test.

- Haemoglobin < 8 gm/dL
- Neutrophil count <2 000/mm3
- Platelet count <100 000/mm3
- Creatinine > 2 x upper limit of normal (ULN)
- AST (GOT) > 2 x  upper limit of normal (ULN)
- ALT (GPT) > 2 x  upper limit of normal (ULN)
- γ-GT > 2 x   upper limit of normal (ULN)

**13.5.2.2** **Urine** samples will be collected in clean pots labeled with the identifiers of each study participant for routine urine examination using a dipstick method. In addition, the pregnancy test will be conducted within 1-2 hours at room temperature (RT) or within 24 hrs of collection if the sample was refrigerated at 4°C.

**13.5.2.3** At sites where facilities exist, **vascular endothelium growth factors** (VEGF and other appropriate biomarkers important in lymphangiogenesis and lymphatic filarial infections) will be assessed at baseline and during the different follow-up visits at appropriate time points identified by investigators after the study drugs administration (optional for sites that are equipped to measure these factors).

**13.5.3** **Clinical photographs**

Digital clinical photographs of the affected and normal limb will be taken at baseline and at all follow-up time points (6, 12, & 24 months). Four facets will be taken and the distance, lighting and background will be standardized for each site by the investigator and all efforts to ensure comparability will be taken at each site. Close up of mossy lesions will also be captured. The photographs will be stored as digital images, linked by barcode/patient id number and date and made available for analysis.

**13.5.4 Quality of life (QOL) assessment**

QOL assessments will be performed at baseline, 12 and 24 months using a modified version of the WHODAS 2.0, (Bedirhan Üstün, 2010) a generic health and disability assessment tool grounded in the conceptual framework of the ICF and capturing an individual’s level of functioning in six major life domains: (i) cognition (understanding and communication); (ii) mobility (ability to move and get around); (iii) self-care (ability to attend to personal hygiene, dressing and eating, and to live alone); (iv) getting along (ability to interact with other people); (v) life activities (ability to carry out responsibilities at home, work and school); (vi) participation in society (ability to engage in community, civil and recreational activities). The questionnaire will be administered using translations in the local language by trained social / health workers.

**14:  Sample size calculations**

The proposed multi-center study is a double blind RCT to compare the efficacy of doxycycline once daily versus placebo in improving filarial lymphedema, grades 1-6, (independent of active filarial infection) with the following arms:

Arm 1: Doxycycline daily x 6 weeks

Arm 2: Placebo daily x 6 weeks

(Measures of Hygiene common for both arms)

**Hypothesis to be tested**: Doxycycline is **superior** to placebo for management of lymphedema in patients from LF-endemic areas

Primary efficacy endpoint:

- Improvement or halt of progression (Lack of progression) of LE when examined 24 months after treatment onset

Secondary endpoint(s):

- Improvement or halt of progression (Lack of progression) of LE when examined 12 months after treatment onset
- Improvement of LE when examined 12 and 24 months after treatment onset
- Change of LE stages (reduction or increase) compared to baseline assessed at 12 and 24 months
- Reduction in the frequency of acute ADLA attacks evaluated from 0-12 months and from 12-24 months after treatment onset
- Change in the circumference of the affected limb from baseline
- Changes in skin thickness at 12 and 24 months compared to the baseline
- Changes in circumference and volume at 12 and 24 months compared to the baseline
- Change in angiogenic, pro-fibrotic or pro-inflammatory biomarkers at 12 and 24 months following doxycycline administration (at sites where these tests are done)

**Sample size estimation**

- Doxycycline will be tested for superiority to placebo
- Up to 250 patients (100 in each arm) to be allocated to treatment at each study site.
- Within each arm there will be two groups:
  - Group A (Stages 1-3; n=100)
  - Group B (Stages 4-6; n= up to 25 patients per site)
- Recognizing that a) most sites have a greater number of patients with earlier stages of lymphedema (Group A - Grades 1-3) and b) measurable change post- treatment is more likely to happen in this Group recruitment will be biased in favor of this group. Therefore, the population will be stratified according to grade, with separate randomization for these two groups. However, the study is only powered for the lower grades and each site will be encouraged to recruit patients with Stages 1-3 lymphedema. Recruitment of patients with 4-6 grades (Group B) will continue until all lower grade patients are recruited and closed. Available subjects will be allocated to treatment, and recruitment will cease when all subjects have been allocated to the lower stage group.
- The strict control implementation of hygiene measures may result in a stronger impact of this intervention on both arms. This would result in a smaller margin to verify the added benefit of doxycycline and as a consequence increase the number of patients in the study. In order to account for this influence the progression in the placebo group is assumed to be 25% instead of 55% as described in Mand et al.
- A dropout rate of 30% is assumed in these calculations.

**15: Strategies for achieving adequate participant enrolment to reach target sample size**

A multi-center study design has been chosen for the following reasons:

1) To study patients across different epidemiological settings (Asia vs Africa) drawn from different cultural backgrounds and governed by different clinical practices that impact on access to resources for treatment and whose filariasis is caused by different parasites (*W. bancrofti* vs *B. malayi*)

2) To facilitate recruitment of sufficient number of patients having different grades of lymphedema (1-6), recognizing that not every centre will have patients with the full range of lymphedema grades available for enrollment to the study.

Each clinical center involved in the study will be selected based on documentation for patient availability and availability of infrastructure and resources for the study. Each site will choose its resources for identifying and recruiting potential subjects. This will include details of where participants will be recruited (e.g., clinic, community), by whom (e.g., health workers, clinicians), when (e.g., time after diagnosis), and how (e.g., field visits, review of health records) and be recorded in the site records.

**16.** **Method of generating the allocation sequence**

A centre agreed by all the trial sites and the sponsor will generate allocation sequences for all the sites. Allocation sequence for each site will be generated for the two treatments in variable blocks of 4-10 and separate for both groups (grade 1-3, grade 4-6). Consenting subjects will be allocated to treatment sequentially according to the randomization list with treatment allocation being provided in individual envelopes

**17 Blinding**

At each trial site trial participants, care providers, and outcome assessors will be blinded to the drugs received by patients.

**18. Data collection methods**

Data will be captured using Clinical Report Forms (CRFs; Annex 8) specifically designed for the study and approved by the DSMC (and IRBs). Research data will be captured by mixed methods, paper and electronic. Research data collected on paper CRFs will be transcribed and entered onto the REDCap (Research Electronic Data Capture: <http://project-redcap.org/> ) system. Data may also be directly entered into the REDCap program using electronic equipment. The equipment may include laptops, or mobile applications for tablet computers and/or smartphones with either Android or iOS operating systems. All electronic tools will be password protected.

Lymphatech scanner’s captured digital data will be collected and uploaded onto Dropbox with participant’s ID number, reference number and date stamp. Data from Dropbox and REDCap will merged at the end of the trial and possibly at the interim analysis if performed.

All clinical and laboratory procedures will be performed according to standard protocols governed by GCP and GLP guidelines, 21 CFR Part 11. Tools for qualitative assessments will be made available in the local languages and validated prior to use.

All study personnel participating will undergo training in all procedures to be used in the study.

A work instruction’s manual / manual of SOPs will be prepared at each site and will be made available to all study personnel and study monitors.

**19. Data Management**

Local health care workers (nurses, clinical officer/physician assistants and counsellor/community health workers) will be able to enter CRF data. Only the Study PI, Data Manager (US), local PI and Data Manager, or other authorized and designated individual will be able to make any changes to the data.

All paper CRF forms will be secured in fireproof locking cabinet and ultimately entered using an electronic data capturing system (EDC) called REDCap using electronic tools. All tools will be password protected. The EDC application is a secure encrypted web application for building and managing online surveys and databases specifically geared to support data capture for research studies. REDCap meets regulatory requirements for GCP/GCLP, 21 CFR Part 21 and HIPAA compliant with full audit trails capability for tracking data manipulation and user activity, as well as automated export procedures for seamless data downloads to Excel, PDF, and common statistical packages (SPSS, SAS, Stata, R).

Digital data collected through the Lymphatech scanner will be uploaded onto Dropbox. Only the patient id number, reference number and date stamp will be the identifiers to each file. Dropbox is HIPAA-GCP compliant.

Data from each participants will merged from RedCAP and DropBox at the end of the trial for cleaning purposes and future analysis.

**20. Data Analysis Plan (DAP)**

**A detailed plan for data analysis will be written before final closure of the data file and before de-blinding of the research team. The Data Safety Monitoring Committee (DSMC) will have to agree on the data analysis plan before closure.**

Analyses will be done per protocol (PP) and per intention-to-treat (ITT). The primary outcome will be analyzed using Fisher’s exact test.

Nominal variables will be given per total number, in percent and with confidence intervals; quantitative variables will be described with mean, standard deviation, minimum, maximum, median, 25th and 75th-percentiles and confidence intervals for each intervention group and analyzed using appropriate statistical methods.

The two sample comparisons for the disease change measures will use the Wilcoxon-Mann-Whitney test. Confidence intervals on the difference in each change measure between the two groups will be done using the Hodges-Lehmann method. Additionally, box plots or other graphical methods may be used to present the data. Additional tests to be included based on choice of endpoints, if necessary.

Definitions of endpoints:

Primary efficacy endpoint:

- Improvement or halt of progression (Lack of progression) of LE when examined 24 months after treatment onset

Secondary endpoint(s):

- Improvement or halt of progression (Lack of progression) of LE when examined 12 months after treatment onset
- Improvement of LE when examined 12 and 24 months after treatment onset
- Change of LE stages (reduction or increase) compared to baseline assessed at 12 and 24 months
- Change in the circumference of the affected limb from baseline
- Reduction in the frequency of acute ADLA attacks evaluated from 0-12 months and from 12-24 months after treatment onset
- Changes in skin thickness at 12 and 24 months compared to the baseline by U/S
- Changes in leg volume at 12 and 24 months to be compared with baseline measurement with use of the Lymphatech scanner tool
- Change in angiogenic, pro-fibrotic or pro-inflammatory biomarkers at 12 and 24 months following doxycycline administration (at sites where these tests are done)

Two separate analyses will be carried out: a) An analysis of change from 0 to 24 months and b) An analysis of change from 0 to 12 months. Additional analyses may be conducted based on results obtained at 3 and 6 months by individual sites. At the discretion of country participation, an aggregated mega-analysis from all sites may be performed and likewise of subanalyses.

**21 Data monitoring**

All study data (other than Lymphascan data) will be maintained through an EDC system (REDCap). Lymphascan data will be captured directly and stored in the Dropbox Cloud. Data will be collected directly from subjects during study visits and telephone calls, or will be abstracted from subjects’ medical records. Source documents include all recordings of observations or notations of clinical activities and all reports and records necessary to confirm the data abstracted for this study. Authorized individuals will perform data entry. The Investigator is responsible for assuring that the data collected are complete, accurate, and recorded in a timely manner.

**21a. Composition of Data Safety Monitoring Committee (DSMC); summary of its role and reporting structure; statement of whether it is independent from the sponsor and competing interests**

A single central DSMC will be established with a charter (Annex 9) that defines the roles and responsibilities and details of meeting frequency and communications. The DSMC thus established will, in addition to the IRBs involved, have a chance to comment on the content of the protocol. In addition, site steering committees responsible for overseeing safety and operation of the study at the site, but dependent on the central DSMC for decisions affecting the whole study at all sites will be established.

**21b. Description of any interim analyses and stopping guidelines, including who will have access to these interim results and make the final decision to terminate the trial**

An interim analysis is planned at each study site when all patients have completed 12 months of follow-up. A decision at 12 months may be taken to terminate the study because of superiority of the doxycycline arm so that the placebo subjects can be treated. Superiority may be defined as non-progression of grade, reduced ADLA, etc. Details of the parameters to be used for the interim analysis will be defined prior to the closing of the database and incorporated in the plan for statistical analysis.

The IRB/EC, the Sponsor, the single central DSMC or the National Drug Authority may halt the study at any time following review of any safety concerns independent of the interim analysis and applicable to all centers.

Halting the study requires immediate discontinuation of study agent administered for all subjects and suspension of enrollment until a decision is made whether or not to continue study agent administration.

The halting criteria (as determined by site investigators) for an individual site include:

• two or more subjects experience the same or similar SAEs that are unexpected and are possibly, probably, or definitely related to the study agent

OR

• any safety issue that the site investigators determine should halt the study

The halting criteria (as determined by the study DSMC secondary to aggregate data review) for this study include:

• Two or more of the same or similar AE in different subjects that are grade 3 or above and are unexpected and possibly, probably, or definitely related to the study agent

OR

• any safety issue that the study DSMC determines should halt the study

**22:  Plans for collecting, assessing, reporting, and managing solicited and spontaneously reported adverse events and other unintended effects of trial interventions or trial conduct.**

**22.1 Documenting, Recording and Reporting Adverse Events**

At each contact with the subject, information regarding adverse events will be elicited by appropriate questioning and examinations and will be:

- immediately documented in the subject’s medical record/source document,
- recorded on the Adverse Event Case Report Form (AE CRF) or electronic database, and
- reported as outlined

**22.2 Definitions**

Adverse Event (AE)

An adverse event is any untoward or unfavorable medical occurrence in a human subject, including any abnormal sign (e.g., abnormal physical exam or laboratory finding), symptom, or disease, temporally associated with the subject’s participation in the research, whether or not considered related to the research.

Adverse Reaction (AR)

An adverse reaction is an adverse event that is caused by an investigational agent (drug or biologic).

Suspected Adverse Reaction (SAR)

An adverse event for which there is a reasonable possibility that the investigational agent caused the adverse event. ‘Reasonable possibility’ means that there is evidence to suggest a causal relationship between the drug and the adverse event. A suspected adverse reaction implies a lesser degree of certainty about causality than adverse reaction which implies a high degree of certainty.

Serious Adverse Event (SAE)

A Serious Adverse Event is an AE that results in one or more of the following outcomes:

- death
- a life threatening (i.e., an immediate threat to life) event
- an inpatient hospitalization or prolongation of an existing hospitalization
- a persistent or significant incapacity or substantial disruption of the ability to conduct normal life functions
- a congenital anomaly/birth defect
- a medically important event*

* Medical and scientific judgment should be exercised in deciding whether expedited reporting is appropriate in other situations, such as important medical events that may not be immediately life threatening or result in death or hospitalization but they may jeopardize the subject or may require intervention to prevent one of the other outcomes listed above.

Unexpected Adverse Event

An AE is unexpected if it is not listed in the Investigator’s Brochure (IB) or Package Insert (PI) (for marketed products) or is not listed at the specificity or severity that has been observed. It is the responsibility of the IND Sponsor to make this determination.

Serious and Unexpected Suspected Adverse Reaction (SUSAR)

A SUSAR is a Suspected Adverse Reaction that is both Serious and Unexpected.

Unanticipated Problem (UP)

An Unanticipated Problem is any event, incident, experience, or outcome that is

1. unexpected in terms of nature, severity, or frequency in relation to
2. the research risks that are described in the IRB-approved research protocol and informed consent document; Investigator’s Brochure or other study documents; and
3. the characteristics of the subject population being studied; and
4. possibly, probably, or definitely related to participation in the research; and
5. places subjects or others at a greater risk of harm (including physical, psychological, economic, or social harm) than was previously known or recognized. (Per the Sponsor, an AE with a serious outcome will be considered increased risk.)

Unanticipated Problem that is not an Adverse Event (UPnonAE)

Unanticipated problem that is not an Adverse Event (UPnonAE): An unanticipated problem that does not fit the definition of an adverse event, but which may, in the opinion of the investigator, involve risk to the subject, affect others in the research study, or significantly impact the integrity of research data. Such events would be considered a non-serious UP. For example, we will report occurrences of breaches of confidentiality, accidental destruction of study records, or unaccounted-for study drug

**22. 3. Investigator Assessment of Adverse Events**

If a diagnosis is clinically evident (or subsequently determined), the diagnosis rather than the individual signs and symptoms or lab abnormalities will be recorded as the AE.

All AEs occurring from the time the informed consent is signed through the end of study period will be documented, recorded, and reported.

**22.3.1 Severity**

The “Common Terminology Criteria for Adverse Events (CTCAE)” (v 4.0):

<http://ctep.cancer.gov/protocolDevelopment/electronic_applications/ctc.htm>

**22.3.2** The Investigator will evaluate all AEs with respect to **Seriousness** (criteria listed above), **Severity** (intensity or grade), and **Causality** (relationship to study agent and relationship to research) according to the following guidelines.

The intensity of the following AEs will be assessed by the trial clinician as described in the Table below:

**Assessment of Adverse Event Intensity for Doxycycline**

| **Adverse Event** | **Grade** | **Intensity** |
| --- | --- | --- |
| Stomach Pain | 0  1  2  3 | Absent  Pain is easily tolerated (able to eat)  Pain interferes with daily activities (unable to eat)  Pain that prevents daily activities (combined with vomiting and/or diarrhoea) |
| Loss of appetite | 0  1  2  3 | Absent  Accompanied with nausea  Accompanied with nausea and vomiting or diarrhoea  Accompanied with nausea and loss of weight |
| Nausea | 0  1  2  3 | None  Nausea that is easily tolerated (able to eat)  Nausea that interferes with daily activity (unable to eat)  Nausea that prevents daily activity (combined with vomiting and/or diarrhoea) |
| Vomiting | 0  1  2  3 | None  Vomiting that is easily tolerated (able to eat)  Vomiting that interferes with daily activity (unable to eat)  Vomiting that prevents daily activity (combined with nausea and/or diarrhoea) |
| Diarrhea | 0  1  2  3 | None  Diarrhoea that is easily tolerated (up to 3 times per day)  Diarrhoea that interferes with daily activity (more than 3 times per day combined with nausea and/or vomiting and weakness)  Diarrhoea that prevents daily activity  (combined with nausea, vomiting and/or acholic faeces) |
| Bloody diarrhea | 0  1  2  3 | None  Fresh or clotted in absence of haemorrhoids  Fresh or clotted blood combined with abdominal pain  Fresh and clotted blood combined with abdominal pain and fever |
| Headache | 0  1  2  3 | None  Headache that is easily tolerated  Headache that interferes with daily activity  Headache that prevents daily activity |
| Urticaria | 0  1  2  3 | None  Requiring no medication  Requiring oral and/or topical medication (including steroid)  for < 24h  Requiring oral, topical and/or medication IV medication (including steroid) for > 24h |
| Rashes | 0  1  2  3 | Absent  Localized, itching, no blisters, lasting one day  Localized itching, with blisters lasting longer than one day  Generalized combined with fever |
| Phototoxicity | 0  1  2  3 | Absent  Erythema in sun exposed skin  Erythema in sun exposed skin and rise of skin temperature  Erythema in sun exposed skin, blisters and fever |
| Fever | 0  1  2  3 | ≤ 37.5°C  >37.5°C-38°C  >38°C-39°C  >39°C |
| Anaphylactic reactions | 0  1  2  3 | None  headache accompanied by rash and itching  in addition to 1. blood pressure failure and tachycardia, oedema  in addition to 2. bronchial spasms (expiratory stridor)  apnoea or cardiac arrest  SAE |

**22.3.3 Causality**

Causality (likelihood that the event is related to the study agent) will be assessed considering the factors listed under the following categories:

**Definitely Related**

- reasonable temporal relationship
- follows a known response pattern
- clear evidence to suggest a causal relationship
- there is no alternative etiology

**Probably Related**

- reasonable temporal relationship
- follows a suspected response pattern (based on similar agents)
- no evidence of a more likely alternative etiology

**Possibly Related**

- reasonable temporal relationship
- little evidence for a more likely alternative etiology

**Unlikely Related**

- does not have a reasonable temporal relationship

OR

- good evidence for a more likely alternative etiology

**Not Related**

- does not have a temporal relationship

OR

- definitely due to an alternative etiology

**22.6 Investigator Reporting Responsibilities to the Sponsor**

Adverse Events

Line listings, frequency tables, and other summary AE data will be submitted to the Sponsor when needed for periodic safety assessments requested by the DSMC, review of annual reports, review of safety reports, and preparation of final study reports.

Serious Adverse Events (SAEs)

SAEs (whether or not they are also UPs) must be reported on the SAE/UP Report Form and sent to the Sponsor Clinical Safety Office (CSO) / Trial Steering Committee by fax or e-mail attachment. Deaths and immediately life threatening SAEs must be reported within 1 calendar day after the site becomes aware of the event. All other SAEs must be reported within 7 business days of site awareness. All SAEs will be reported to the DSMC on receipt, and followed up where necessary.

Unanticipated Problems (UPs)

Unanticipated Problems that are also adverse events must be reported to the CSO and sent by fax or e-mail attachment no later than 7 calendar days of site awareness of the event. UPs that are not AEs are not reported to the Sponsor CSO.

**23. Frequency and procedures for auditing trial conduct, if any, and whether the process will be independent from investigators and the sponsor**

**TBD**

**24. Plans for seeking research ethics committee/institutional review board (REC/IRB) approval**

The trial will be carried out conforming to the principles of the Declaration of Helsinki 1964 (amended most recently in 2013) and according to Good Clinical Practice (GCP) guidelines and according to 21 CFR Part 11 guidelines. Each trial site will submit a site-specific protocol to its REC / IRB prior to the commencement of the trial at that site. The principal investigator at each site is responsible for the preparation of the protocol based on the core (parent) protocol. The composition of the REC / IRB and procedures for approval of the protocol will be in accordance with the guidelines prescribed by the National authorities where the study will be conducted.

This trial will be registered at the following trial registries:

ClinicalTrial.gov

SRI LANKA: [NCT02929134](https://clinicaltrials.gov/ct2/show/NCT02929134?term=NCT02929134&rank=1)

INDIA: [NCT02929121](https://clinicaltrials.gov/ct2/show/NCT02929121?term=NCT02929121&rank=1)

MALI: NCT02927496

Clinical Trial Registry of India : CTRI/2017/08/009312

**25. Plans for communicating important protocol modifications (e.g. changes to eligibility criteria, outcomes, analyses) to relevant parties (e.g. investigators, REC/IRBs, trial participants, trial registries, journals, regulators)**

The Trial Steering Committee will be responsible for communicating changes to the protocol to the Principal Investigators at each site.

**26. Consent or assent**

**Informed Consent Process**

Informed consent is a process where information is presented to enable persons to voluntarily decide whether or not to participate as a research subject. It is an on-going conversation between the human research subject and the researchers that begins before consent is given and continues until the end of the subject's involvement in the research. **The process of obtaining informed consent and assent will be governed by the guidelines of the respective countries where the trial will take place.**

Broadly, discussions about the research will provide essential information about the study and include: purpose, duration, experimental procedures, alternatives, risks and benefits. Subjects will be given the opportunity to ask questions and have them answered. The subjects will sign the informed consent document prior to undergoing any research procedures. The subjects may withdraw consent at any time throughout the course of the trial. A copy of the informed consent document will be given to the subjects for their records. The researcher will document the signing of the consent form in the subject’s medical record and the original signed consent retained in a separate file along with other protocol specific documents required for compliance with GCP. The rights and welfare of the subjects will be protected by emphasizing to them that the quality of their medical care will not be adversely affected if they decline to participate in this study.

**26 a. Who will obtain informed consent or assent from potential trial participants or authorized surrogates, and how**

At each site, the principal investigator with the approval of the REC / IRB will finalize the consent seeking process including the allocation of responsibility of obtaining consent from trial participants.

**26 b. Additional consent provisions for collection and use of participant data and biological specimens in ancillary studies, if applicable**

At each site, the principal investigator with the approval of the REC / IRB will finalize the consent seeking process including the allocation of responsibility for collection and use of participant data and biological specimens in ancillary studies from trial participants. This will be subject to an additional informed consent process, separate to the protocol specific consent.

**27. Confidentiality**

All records will be kept confidential to the extent provided by national federal, state and local laws. The study monitors and other authorized representatives of the Sponsor may inspect all documents and records required to be maintained by the Investigator, including but not limited to, medical records. Specific study records will be kept in locked cabinets and all computer data, data entry programmes and networking programs will be password protected. Personal information such as patient names, hospital numbers and addresses will not be recorded on the CRFs used. If records are required for examination, this information will be blanked out; however, a secure record of the linkages between individuals and their records will be maintained. Clinical information will not be released without written permission of the subject, except as necessary for monitoring by IRB, the national regulatory authority, or the sponsor’s designee. All such information will be anonymized.

**28. Declaration of interests**

Principal investigators for the overall trial and each study site will declare their financial and other competing interests prior to the commencement of the trial and these declarations will be available on file for inspection.

**29. Access to data**

Statement of who will have access to the final trial dataset, and disclosure of contractual agreements that limit such access for investigators will be issued once the trial sponsors and funding agencies have been finalized.

The investigator is responsible for retaining all essential documents listed in the ICH Good Clinical Practice Guidelines. Study records will be maintained by the PI for a minimum of 3 years and in compliance with institutional, IRB, state, and federal medical records retention requirements whichever is longest. All stored records will be kept confidential to the extent required by federal, state, and local law.

Should the investigator wish to assign the study records to another party and/or move them to another location, the investigator will provide written notification of such intent with the name of the person who will accept responsibility for the transferred records and/or their new location. Destruction or relocation of research records will not proceed without written permission.

30. **Provisions, if any, for ancillary and post-trial care, and for compensation to those who suffer harm from trial participation.**

Monetary compensation limited to loss of wages and transportation costs will be made to the subjects involved in this study in accordance with the guidelines prescribed by the national regulatory authorities and the respective IRBs.

Each participant will receive a lymphedema hygiene kit consisting essentially of a plastic tub, 5 pieces of soap, 2 cotton towels.

Technical and material support will be provided to community health centers that will house the study.

**The sponsor will cover patient care as it is related to the study, for the life of the protocol, and after the protocol for a reasonable short amount of time as jointly agreed.**

**31. Dissemination policy**

| **31a** | **Plans for investigators and sponsor to communicate trial results to participants, healthcare professionals, the public, and other relevant groups (eg, via publication, reporting in results databases, or other data sharing arrangements), including any publication restrictions**  The results of the multi-center study will be published in a joint paper with authorship from all participating study sites. In addition, each site will be able to publish its site-specific results independently following the submission of the joint paper or 6 months after closure of the last collaborating study, whichever is earlier. |
| --- | --- |
| **31b** | **Authorship eligibility guidelines and any intended use of professional writers**  Authorship will be restricted to those persons that had a significant input into the design, implementation and analysis of the study. Professional writers will not be used. |
| **31c** | **Plans, if any, for granting public access to the full protocol, participant-level dataset, and statistical code**  This will be defined once the sites and investigators have been identified and the processes have been agreed by the investigators. |

**References**

Gyapong, J.O., et al. Treatment strategies underpinning the global programme to eliminate lymphatic filariasis. Expert Opin Pharmacother. 2005

Global programme to eliminate lymphatic filariasis: progress report, 2013. Weekly Epidemiol Rec. 2014 Sep 19; 89(38):409-18.

Ramaiah KD, Ottesen EA (2014) Progress and Impact of 13 Years of the Global Programme to Eliminate Lymphatic Filariasis on Reducing the Burden of Filarial Disease. PLoS Negl Trop Dis 8(11): e3319. doi: 10.1371/journal.pntd.0003319

Mand, S., et al. Doxycycline Improves Filarial Lymphedema Independent of Active Filarial Infection: A Randomized Controlled Trial. Clin Infect Dis. 2012

Mand, S., et al. Macrofilaricidal activity and amelioration of lymphatic pathology in Bancroftian filariasis after 3 weeks of doxycycline followed by single-dose diethylcarbamazine. Am J Trop Med Hyg. 2009.

Taylor, M.J. et al., Macrofilaricidal activity after doxycycline treatment of Wucheraria bancrofti: a double-blind, randomised placebo-controlled trial. Lancet. 2005.

Turner, J.D., et al. A randomized, double blind clinical trial of a 3-week course of doxycycline plus albendazole and ivermectin for the treatment of Wucheraria bancrofti infection. Clin Infect Dis. 2006.

Debrah, A.Y., et al. Macrofilaricidal effect of 4 weeks of treatment with doxycycline on *Wuchereria bancroft*i. Trop Med Int Health. 2007

Debrah, A.Y., et al. Reduction in levels of plasma vascular endothelial growth factor-A and improvement in hydrocele patients by targeting endosymbiotic Wolbachia sp. in *Wucheraria bancrofti* with doxycycline. Am J Trop Med Hyg. 2009

Debrah, A.Y., et al. Doxycycline reduces plasma VEGF-C/sVEGFR-3 and improves pathology in lymphatic filariasis. PLoS Pathog. 2006

Dreyer G., A.D., Dreyer P., Noroes J. Basic lymphedema management: Treatment and prevention of problems associated with lymphatic filariasis. New Hampshire: Hollis. 2002

Coulibaly YI, et al. A randomized trial of doxycycline for Mansonella perstans infection. N Engl J Med. 2009 Oct 8;361(15):1448-58.

Shenoy RK, Kumaraswami V, Suma TK, Rajan K, Radhakuttyamma G. A double-blind, placebo-controlled study of the efficacy of oral penicillin, diethylcarbamazine or local treatment of the affected limb in preventing acute adenolymphangitis in lymphoedema caused by brugian filariasis. Ann Trop Med Parasitol 1999; 93:367–77.

WHO. Lymphatic Filariasis: the disease and its control. Tech Rep Ser 1992; 821:1–71.

Bedirhan Üstün, et al. Developing the World Health Organization Disability Assessment Schedule 2.0 Bull World Health Organ 2010; 88:815–823

**Appendix 1: Summary of Country Specific Investigators**

| **Country** | **Investigator Name** | **Email** |
| --- | --- | --- |
| India | Dr. Suma T K | [sumatk@gmail.com](mailto:sumatk@gmail.com) |
| Mali | Channa Yahathugoda | [tcyahath@yahoo.co.uk](mailto:tcyahath@yahoo.co.uk) |
| Sri Lanka | Yaya Ibrahim Coulibaly | [yicoulibaly@icermali.org](mailto:yicoulibaly@icermali.org) |

**Appendix 2**

**Staging of lymphedema (Dreyer 2002)**

- Stage 1= Swelling is reversible (goes away) overnight
- Stage 2= Swelling is not reversible (doesn’t go away)
- Stage 3= Presence of shallow skin folds (base of fold can be seen with movement of leg)
- Stage 4= Presence of skin knobs
- Stage 5= Presence of deep skin folds (base of fold can only be seen if opened up)
- Stage 6= Presence of “mossy lesions” Warty looking epidermal skin lesions.
- Stage 7 = Unable to care for self or perform daily activities

**Appendix 3 - Lab Profile**

- Haemoglobin < 8 gm/dL
- Neutrophil count <2 000/mm3
- Platelet count <100 000/mm3
- Creatinine > 2 x upper limit of normal (ULN)
- AST (GOT) > 2 x  upper limit of normal (ULN)
- ALT (GPT) > 2 x  upper limit of normal (ULN)
- γ-GT > 2 x   upper limit of normal (ULN)

**Appendix 4 – WASH Diary**

To be inserted

**Appendix 5 – Basic Hygiene**

Standard Operating Procedure for the Care and Management of the Skin in

Patients with Lymphoedema in Tropical Regions, *Version 2.3 CDM 2013 - adapted from Rotherham Doncaster UK NHS*d

**Appendix 6 - Study Schedule**

| SCHEDULE OF ACTIVITIES |
| --- |
|  | **Visit 1**  **Screening** | **Visit 2**  **Baselinea** | **Visit 3**  **Treatment** | | | | | | |
|  |  |  | **Day 1** | **Day 2-21** | **Day 22** | **Day 23-41** | **Day 42** | ***Range Days***  ***43-49*** | **End of treatment**  **(**one day after treatment no. 42) |
| Informed Consents/ Assents (clinical trial and biobanking) | √ |  |  |  |  |  |  |  |  |
| Demographic data | √ |  |  |  |  |  |  |  |  |
| Lymphedema staging | √ | √ |  |  |  |  |  |  |  |
| Clinical photographs |  | √ |  |  |  |  |  |  |  |
| History of lymphedema | √ |  |  |  |  |  |  |  |  |
| History of ADLA | √ |  |  |  |  |  |  |  |  |
| Circumference - Tape |  | √ |  |  |  |  |  |  |  |
| Circumference - Lymphatech® |  | √ |  |  |  |  |  |  |  |
| Volume of LE - Lymphatech® |  | √ |  |  |  |  |  |  |  |
| Ultrasound |  | √ |  |  |  |  |  |  |  |
| Medical history | √ |  |  |  |  |  |  |  |  |
| Concomitant medication | √ | √ | √ | √ | √ | √ | √ | *(√)* | √ |
| History of relevant medications | √ |  |  |  |  |  |  |  |  |
| Vital signs | √ | √ |  |  |  |  |  |  |  |
| Physical examination | √ | √ |  |  |  |  |  |  |  |
| QoL |  | √ |  |  |  |  |  |  |  |
| Hygiene status |  | √ |  |  |  |  |  |  |  |
| Lymphedema management training |  | √ |  |  |  |  |  |  |  |
| Laboratory assessment (blood) | √ | *(√)a* | *(√)b* |  | √ (before treatment no. 22, AST/ALT/γ-GT, range +2 days) | *√ before treatment no. 22, AST/ALT/γ-GT, range +2 days)c* | √ (on the last day of treatment, AST/ALT/γ-GT, range +2 days) | *√ ( on the last day of treatment, AST/ALT/γ-GT, range +2 days)d* | *(√) d* |
| Urine sample collected | √ | √ | √ | √ |  | √ | √ | *(√) d* | *(√) d* |
| Saliva sample collected | √ |  |  |  |  |  |  |  |  |
| Pregnancy test | √ | √ | *(√)b* | √ (on day 15, range + 2 days) |  | √ (on day 29, range + 2 days) | √ (on day 42, range + 2 days) |  |  |
| In-/Exclusion criteria | √ | √ | *(√)b* |  |  |  |  |  |  |
| Randomization |  | √ |  |  |  |  |  |  |  |
| Presence for visit |  |  |  | √ | √ | √ | √ | *(√)* | √ |
| Individual treatment |  |  | √ | √ | √ | √ | √ | *(√)* |  |
| ADLA questionnaire |  | √ | √ | √ | √ | √ | √ | *(√)* | √ |
| AEs |  |  |  | √ | √ | √ | √ | *(√)* | √ |
| End of study record |  |  |  |  |  |  |  |  |  |

a Visit 2 (Baseline) should take place max. 28 days after visit 1 (Screening). If that is not the case, blood tests have to be repeated. bVisit 3 (Treatment) should start on the same day or one day after visit 2 (Baseline). If that is not the case, the pregnancy test has to be repeated and in case the period between the two visits is > 28 days also blood tests and check of in- and exclusion criteria have to be repeated.

c only to be done if not already done on day 22 d only to be done if not already done on day 42

|  | **Visit 4**  **2 months follow-upa** | **Visit 5**  **4 months follow-upb** | **Visit 6**  **6 months follow-upc** | **Visit 7**  **8 months**  **follow-upd** | **Visit 8**  **10 months follow-upe** | **Visit 9**  **12 months follow-upf** | **Visit 10**  **14 months follow-upg** | **Visit 11**  **16 months follow-uph** | **Visit 12**  **18 months follow-upi** | **Visit 13**  **20 months follow-upj** | **Visit 14**  **22 months follow-upk** | **Visit 15**  **24 months**  **follow-upl** |
| --- | --- | --- | --- | --- | --- | --- | --- | --- | --- | --- | --- | --- |
| Informed Consents/ Assents (clinical trial and biobanking) |  |  |  |  |  |  |  |  |  |  |  |  |
| Demographic data |  |  |  |  |  |  |  |  |  |  |  |  |
| Lymphedema staging |  |  | √ |  |  | √ |  |  |  |  |  | √ |
| Clinical photographs |  |  | √ |  |  | √ |  |  |  |  |  | √ |
| History of lymphedema |  |  |  |  |  |  |  |  |  |  |  |  |
| History of ADLA |  |  |  |  |  |  |  |  |  |  |  |  |
| Circumference - Tape |  |  | √ |  |  | √ |  |  |  |  |  | √ |
| Circumference - Lymphatech® |  |  | √ |  |  | √ |  |  |  |  |  | √ |
| Volume of LE - Lymphatech® |  |  | √ |  |  | √ |  |  |  |  |  | √ |
| Ultrasound |  |  | √ |  |  | √ |  |  |  |  |  | √ |
| Medical history |  |  |  |  |  |  |  |  |  |  |  |  |
| Concomitant medication | √ | √ | √ | √ | √ | √ | √ | √ | √ | √ | √ | √ |
| History of relevant medications |  |  |  |  |  |  |  |  |  |  |  |  |
| Vital signs |  |  | √ |  |  | √ |  |  |  |  |  | √ |
| Physical examination |  |  |  |  |  |  |  |  |  |  |  |  |
| QoL |  |  |  |  |  | √ |  |  |  |  |  | √ |
| Hygiene status |  | √ | √ |  |  | √ |  |  | √ |  |  | √ |
| Lymphedema management training |  | √ | √ |  |  | √ |  |  | √ |  |  | √ |
| Laboratory assessment (blood) |  |  | √ |  |  | √ |  |  |  |  |  | √ |
| Urine sample collected |  |  | √ |  |  | √ |  |  |  |  |  | √ |
| Saliva sample collected |  |  | √ |  |  | √ |  |  |  |  |  | √ |
| Pregnancy test | √ |  | √ |  |  | √ |  |  |  |  |  | √ |
| In-/Exclusion criteria |  |  |  |  |  |  |  |  |  |  |  |  |
| Randomization |  |  |  |  |  |  |  |  |  |  |  |  |
| Presence for visit | √ | √ | √ | √ | √ | √ | √ | √ | √ | √ | √ | √ |
| Individual treatment |  |  |  |  |  |  |  |  |  |  |  |  |
| ADLA questionnaire | √ | √ | √ | √ | √ | √ | √ | √ | √ | √ | √ | √ |
| AEs | √ | √ |  |  |  |  |  |  |  |  |  |  |
| End of study record |  |  |  |  |  |  |  |  |  |  |  | √ |

a the 2 months follow-up should take place 61 days (± 7 days) after treatment day 1 b the 4 months follow-up should take place 121 days (± 10 days) after treatment day 1

c the 6 months follow-up should take place 182 days (± 21 days) after treatment day 1 d the 8 months follow-up should take place 243 days (± 14 days) after treatment day 1

e the 10 months follow-up should take place 303 days (± 14 days) after treatment day 1 f the 12 months follow-up should take place 364 days (± 21 days) after treatment day 1

g the 14 months follow-up should take place 425 days (± 21 days) after treatment day 1 h the 16 months follow-up should take place 485 days (± 21 days) after treatment day 1

i the 18 months follow-up should take place 546 days (± 21 days) after treatment day 1 j the 20 months follow-up should take place 607 days (± 21 days) after treatment day 1

k the 22 months follow-up should take place 667 days (± 21 days) after treatment day 1 l the 24 months follow-up should take place 728 days (- 28 days; + 56 days) after treatment day 1

**Appendix 7 - Study Design**

Screening in villages / clinics in the endemic villages /districts in the hinterland of selected sites

Eligible subjects from

14 years (n =250)

Consent,

Clinical examination

Measurement and staging of Lymphoedema / elephantiasis

Consent for treatment, physical examination, ICT / Og4C3 (WB123), thick smear, ELISA, transaminases, creatinine, pregnancy test (HCG)

**BASELINE**

**Inclusion**:

Lymphedema (stage 1-6), HCG (-), Normal hematological and biochemical values

**Exclusion**:

Lymphedema (stage > 6), HGG (+), Abnormal hematological and biochemical values

**RANDOMIZATION**

Lymphedema

(n=200 Gr1-3; n2 =<50 Gr 4-6)

Placebo

-Re assessment of Lymphedema (measurement)

Re assessment of hematological and biochemical parameters

- Other re assessments

Follow up at months 3, 6, 12 and 24

**Appendix 8 Measurement points for Lymphedema of limbs**


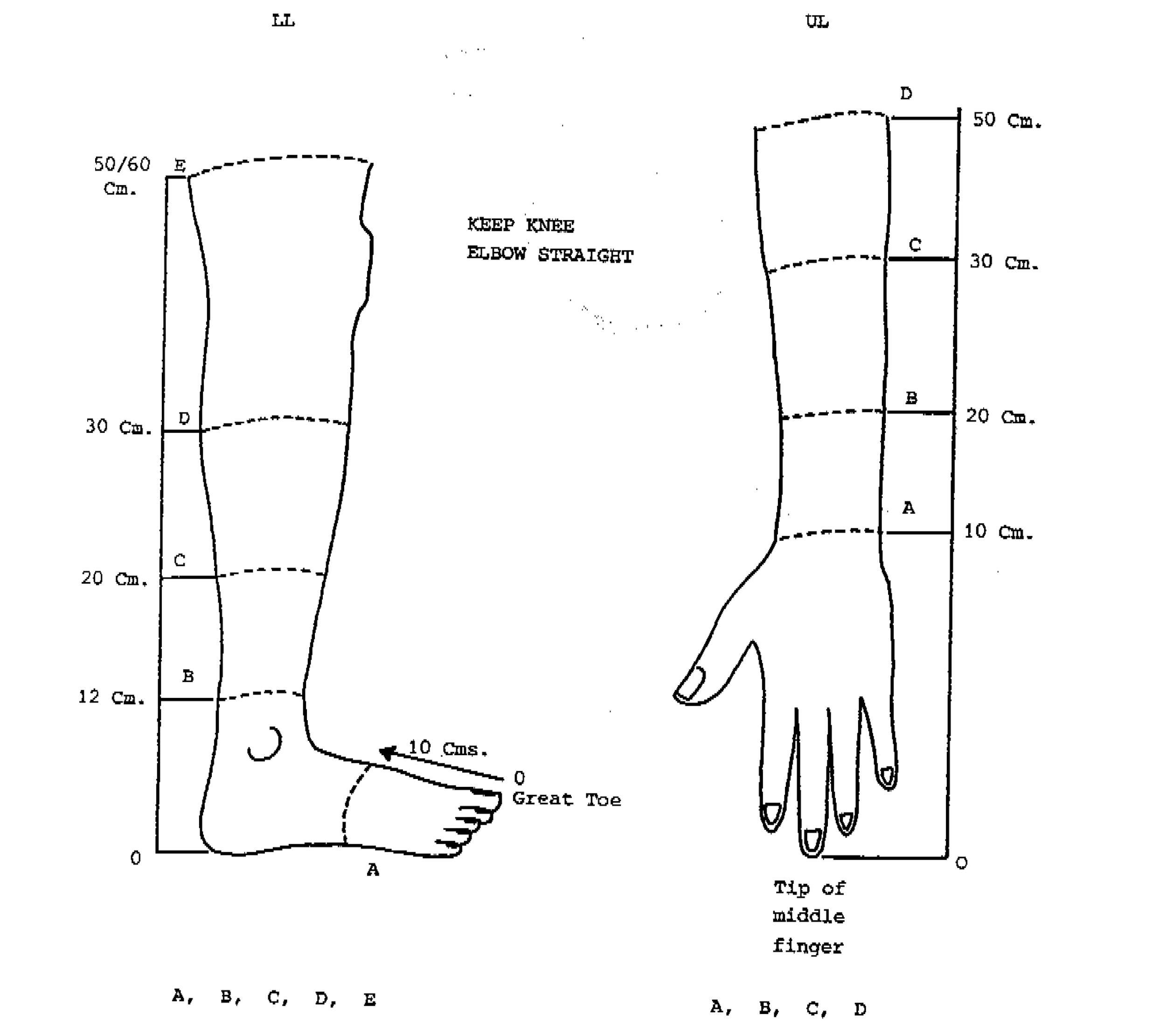


**Appendix 9: Case Report Form (CRFs)**

Available upon request

**Annex 10: Doxycycline Data Safety Monitoring Committee (DSMC)**

| David Addiss |
| --- |
| Title: Director, Children Without Worms |
| Institution: Task Force on Global Health |
| Address: 325 Swanton Way |
| Decatur, GA 30030 |
| Email: [daddiss@taskforce.org](mailto:daddiss@taskforce.org) and [daddiss1@nd.edu](mailto:daddiss1@nd.edu) |
| Phone: |
| AND |
| University of Notre Dame, Professor |
| David J Diemert |
| Title: Associate Professor |
| Institution: The George Washington University, Department of Microbiology, Immunology and Tropical Medicine. |
| AND: Director of Clinical Trials, Sabin Vaccine Institute Product Development Partnership, Albert B Sabin Vaccine Institute |
| Address: 2300 Eye Street NW, Ross Hall 723-D |
| Washington, DC 20037 |
| Email: [ddiemert@gwu.edu](mailto:ddiemert@gwu.edu) |
| Phone: 202.994.2909 |
| Martin Peter Grobusch |
| Title: Professor (Chair) of Tropical Medicine and Head |
| Institution: Center of Tropical Medicine and Travel Medicine, Department of Infectious Diseases |
| Address: Amsterdam Medical Center, University of Amsterdam, The Netherlands |
| Email: [m.p.grobusch@amc.uva.nl](mailto:m.p.grobusch@amc.uva.nl) |
| Phone: *To be inserted* |
| Sabine Klager |
| Title: Operations Director Clinical Trials, Cambridge Clinical Trials Unit |
| Institution: Cambridge University Hospitals NHS Foundation Trust – Addenbrooke’s Hospital |
| Address: Box 401, Hills Road |
| Cambridge, CB2 0QQ |
| Email: [sabine.klager@addenbrookes.nhs.uk](mailto:sabine.klager@addenbrookes.nhs.uk) |
| Phone: 01223 348179 ext 58179 |
