# Supplementary material for: The design and development of a multicentric protocol to investigate the impact of adjunctive doxycycline on the management of peripheral lymphoedema caused by lymphatic filariasis and podoconiosis
Source: Parasit Vectors. 2020 Mar 30;13:155. doi: 10.1186/s13071-020-04024-2 (PMC7106687; doi:10.1186/s13071-020-04024-2)
Supplement: Supplementary file 2 — Additional file 2: Text S2. TAKeOFF filariasis protocol. [file 13071_2020_4024_MOESM2_ESM.pdf]

Additional file 2: Text S2. TAKeOFF filariasis protocol.

Clinical Trial Protocol

LEDoxy

Doxycycline 200mg/d vs. 100mg/d for 6 weeks to improve filarial lymphedema - a multinational, double-blind, randomized, placebo-controlled trial

**Short Title:**	Doxycycline to improve filarial lymphedema
**Trial Acronym:**	TAKeOFF – LEDoxy
**Trial Design:**	multi-national, double-blind, randomized, placebo-controlled phase II trial
**Protocol Code:**	TAKeOFF-4-0117
**Protocol Version:**	GH 2.0 / November 13, 2017
**Principal Investigator:**	Dr. Alexander Yaw Debrah, KCCR/KNUST, Ghana
**PACTR Registration Number:**	PACTR201708002420261
**ISRCTN Registration Number:**	ISRCTN14042737
**Authors:**	Dr. Ute Klarmann-Schulz, Prof. Dr. Achim Hoerauf, Dr. Alexender Yaw Debrah, Dr. Linda Batsa Debrah, Dr. Inge Kroidl,

Confidential

The content of this document is strictly confidential and may not be copied or made accessible to third parties without the consent of the Institute for Medical Microbiology, Immunology and Parasitology (IMMIP), University Hospital of Bonn, Germany, the Division of Infectious Diseases and Tropical Medicine, Medical Centre of the University of Munich (LMU), Germany and the Kumasi Centre for Collaborative Research (KCCR), Kumasi, Ghana.

Protocol Approval Signatures

With our signature we confirm that we are familiar with and understand the protocol and will comply with the principles of Good Clinical Practice (GCP), all applicable local regulations ,and the ethical principles laid down in the Declaration of Helsinki..

Sponsor/Sponsor Delegated Person (SDP):

Prof. Dr. Richard Phillips, KCCR ____________ Date ____________ Signature

Principal Investigator:

Dr. Alexander Yaw Debrah ____________ Date ____________ Signature

Sponsor's medical expert /Trial Clinician:

Dr. Nana Kwame Ayisi-Boateng ____________ Date ____________ Signature

Project Manager:

Francis Adjei Osei ____________ Date ____________ Signature

Trial Pharmacist - Ghana:

Dr. Stephen Yao Gbedema ____________ Date ____________ Signature

Senior Coordinating Physician - Germany:

Prof. Dr. Achim Hoerauf ____________ Date ____________ Signature

Biometrician - Germany:

Dr. Rolf Fimmers ____________ Date ____________ Signature

Trial Clinician - Germany:

Dr. Inge Kroidl ____________ Date ____________ Signature

Project Manager - Germany:

Dr. Ute Klarmann-Schulz ____________ Date ____________ Signature

TABLE OF CONTENTS

1. TRIAL ADMINISTRATION STRUCTURE 7
1.1 Trial administration structure - Ghana 7
1.2 Trial Administration structure - Germany 9
1.3 Funding 10
2. PROTOCOL SYNOPSIS 11
3. SCHEDULE OF ACTIVITIES 18
4. BACKGROUND INFORMATION 20
5. RATIONALE FOR THE STUDY 21
6. STUDY OBJECTIVES 22
6.1 Primary Objectives 22
6.2 Secondary Objectives 22
7. STUDY DESIGN 22
7.1 Study design 22
7.2 Treatment groups 23
7.3 Number of subjects 23
7.4 Participating trial sites 23
7.4.1 Description of the study setting of the trial in Ghana 23
7.4.2 Description of the study setting of the trial in Tanzania 24
7.4.3 Description of the study settings of the three USAID funded trials 24
7.4.4 Overall timeline of the study 25
7.5 Recruitment 25
7.5.1 Recruitment procedure in Ghana 25
7.6 Bias reducing measures 26
7.6.1 Randomization 26
7.6.2 Blinding 26
7.7 Study Endpoints 26
7.7.1 Primary Endpoint 26
7.7.2 Secondary Endpoints 26
7.7.3 Assessment of safety 27
7.8 Study Population and Eligibility Criteria 27
7.8.1 Gender Distribution 27
7.8.2 Inclusion criteria 27
7.8.3 Exclusion criteria 27
7.8.4 Justification for exclusion of women and children 28
7.8.4.1 Pregnant Women 28
7.8.4.2 Children 28
8. TRIAL PROCEDURES 29
8.1 Clinical Evaluation 29
8.1.1 Medical history and physical examination 29
8.1.2 Staging of lymphedema 29
8.1.3 Assessment of acute dermatolymphangioadenitis (ADLA) 29
8.1.4 Circumference measurement of legs using a tape measure 29
8.1.5 Circumference measurement of legs using an infrared scanner (LymphaTech®) 29
8.1.6 Assessment of the leg volume using an infrared scanner (LymphaTech®) 29
8.1.7 Training for care and hygiene of affected legs and arms 29
8.1.8 Ultrasound 30
8.1.9 Clinical photographs 30

8.1.10 Quality of life (QOL) assessment 30
8.2 Laboratory Evaluation 30
8.2.1 Blood 30
8.2.1.1 Hematology and biochemistry 30
8.2.1.2 Filarial specific measurements 31
8.2.1.3 Biomarkers in blood 31
8.2.1.4 T cell activation and differentiation marker 31
8.2.2 Urine 31
8.2.2.1 Urine dipstick 31
8.2.2.2 Urine pregnancy tests 31
8.2.2.3 Biomarkers in urine 31
8.2.3 Saliva 31
8.3 Schedule of Events 31
8.3.1 Screening (Visit 1) 32
8.3.2 Enrolment (Visit 2) 32
8.3.3 Treatment (Visit 3, day 1 – day 43 + 7 days range) 33
8.3.3.1 Treatment day 1 33
8.3.3.2 Treatment days 2 – 42 (+ 7 days range) 33
8.3.3.3 End of treatment (day 43 + 7 days range) 33
8.3.4 2 months follow-up 33
8.3.5 4 months follow-up 34
8.3.6 6 months follow-up 34
8.3.7 Follow-ups at 8, 10, 14, 16, 20 and 22 months after the first day of treatment 34
8.3.8 18 months follow-up 34
8.3.9 12 and 24 months follow-ups 34
9. INVESTIGATIONAL MEDICINAL PRODUCT (IMP) 35
9.1 General description of the study drug doxycycline 35
9.2 Specification of IMP 35
9.3 Dosage and Route of Administration 35
9.4 Findings from clinical studies 36
9.5 Summary of known and potential risks of doxycycline 36
9.5.1 Side effects of doxycycline 36
9.5.2 Precautions to mitigate drug specific risks (Doxycycline) 37
9.5.3 General precautions and warnings 38
9.6 Blistering and Labeling of the IMP 38
9.7 Transport of IMP 38
9.8 Handling of IMP at the Site and Drug Accountability 38
9.9 Strategies to improve adherence and compliance 38
9.10 Unblinding 39
9.11 Prior and Concomitant Therapy/Medication 39
9.11.1 Previous therapy / medication of trial specific illness 39
9.11.2 Previous therapy / medication with doxycycline 39
9.11.3 Concomitant therapy / medication for other indications 39
9.11.4 Prohibited therapy / Concomitant medication 40
9.11.5 Rescue/Escape/Salvage Therapy 40
10. ADVERSE EVENTS 40
10.1 Documenting, Recording and Reporting Adverse Events 40
10.2 Definitions 40
10.2.1 Adverse Event (AE) 40

10.2.2 Unexpected Adverse Event (UAE) ... 40
10.2.3 Adverse (Drug) Reaction (AR) ... 41
10.2.4 Suspected Adverse (Drug) Reaction (SAR) ... 41
10.2.5 Unexpected Adverse (Drug) Reaction (UAR) ... 41
10.2.6 Serious Adverse Event (SAE) ... 41
10.2.7 Serious Adverse (Drug) Reaction (SAR) ... 41
10.2.8 Suspected Unexpected Serious Adverse (Drug) Reaction (SUSAR) ... 41
10.2.9 Unanticipated Problem (UP) ... 41
10.2.10 Unanticipated Problem that is not an Adverse Event (UPnonAE) ... 42
10.3 Criteria to be evaluated by the trial clinician ... 42
10.3.1 Assessment of Intensity ... 42
10.3.2 Assessment of Adverse Event Intensity for Doxycycline ... 42
10.3.3 Assessment of Seriousness ... 43
10.3.4 Assessment of Causality ... 43
10.4 Adverse Event Recording ... 44
10.5 Adverse Event Reporting ... 44
10.6 Serious Adverse Event Reporting ... 44
10.6.1 Serious Adverse Event Reporting ... 44
10.7 Reporting of Unanticipated Problems (UPs) ... 44
10.8 Follow-up of Adverse Events ... 45
10.9 Handling of emergency cases ... 45
10.10 Deaths ... 45
10.11 Pregnancies ... 45
10.11.1 Pregnancy Procedures ... 45
10.11.2 Pregnancy Reporting ... 45
11. STATISTICAL CONSIDERATIONS ... 46
11.1 Hypothesis ... 46
11.2 Primary target variable ... 46
11.3 Sample size estimations ... 46
11.3.1 Sample size estimations for group A (LE stage 1-3) ... 46
11.3.2 Sample size estimations for group B (LE stage 4-6) ... 47
11.4 Sample size calculation ... 47
11.4.1 Sample size calculation for group A (LE stage 1-3) ... 47
11.4.2 Sample size calculation for group B (LE stage 4-6) ... 47
11.5 Achievement of sample size ... 47
11.6 Populations to be analyzed ... 47
11.6.1 Intent-to treat (ITT) population ... 47
11.6.2 Per-Protocol (PP) population: ... 47
11.6.3 Safety Population ... 47
11.7 Statistical methodology ... 48
11.7.1 Analysis of baseline characteristics ... 48
11.7.2 Analysis of the primary endpoint ... 48
11.7.3 Analysis of the secondary endpoints ... 48
11.7.4 Safety Analysis ... 48
11.8 Interim Analysis ... 48
11.9 Protocol violations ... 48
11.10 Handling of Drop-outs, Withdrawal, and Missing Data ... 49
11.10.1 Screening failures ... 49
11.10.2 Drop-out/Withdrawal after randomization but before treatment start ... 49

11.10.3 Drop-out/Withdrawal during or after treatment 49
11.10.4 Missing treatment days 49
11.10.5 Replacement of Patients 49
11.11 Statistical report 49
12. DATA SAFETY AND MONITORING COMMITTEE (DMSC) 50
13. DEFINITION OF END OF TRIAL 50
13.1 Regular end of trial 50
13.2 Termination of the trial for individual subjects 50
13.2.1 Termination by the participant 50
13.2.2 Termination by the investigator 50
13.3 Early termination of the entire trial 51
13.4 Report of termination of the trial 51
13.5 Notification of the end of the trial 51
14. DATA COLLECTION, HANDLING AND RECORD KEEPING 51
14.1 Data Collection methods 51
14.2 Electronic data capture (EDC) 52
14.3 Data management 52
14.4 Trial site file 53
15. MONITORING AND QUALITY ASSURANCE 53
15.1 Study Monitoring 53
15.2 Audits and inspections 53
15.3 Archiving 54
15.3.1 Archiving by the sponsor 54
15.3.2 Archiving by the investigator 54
16. ETHICAL CONSIDERATIONS 54
16.1 Basic principles 54
16.2 Involvement of Ethics Committees and Regulatory Authorities 55
16.3 Responsibilities of the Investigator 55
16.4 Protocol amendments 55
16.5 Benefits and risks of the study 56
16.6 Subject information 56
16.7 Obtaining informed consent 57
16.8 Confidentiality 57
16.9 Declaration of interests 57
17. ADMINISTRATIVE CONSIDERATIONS 57
17.1 Patient Insurance 57
17.2 Incentives and expenses 58
17.3 Trial reports 58
17.3.1 Final Report 58
17.4 Publication Policy 58
18. REFERENCES 59
19. ABBREVIATIONS 60

1. TRIAL ADMINISTRATION STRUCTURE

1.1 Trial administration structure - Ghana

**Sponsor**	**Kumasi Centre for Collaborative Research (KCCR)**<br>Kwame Nkrumah University of Science and Technology<br>Kumasi<br>Ghana<br>Website: http://kccr-ghana.org/
**Sponsor Delegated Person:**	**Prof. Dr. Richard Phillips**<br>Kumasi Centre for Collaborative Research (KCCR)<br>Kwame Nkrumah University of Science and Technology<br>Kumasi<br>Ghana<br>Tel. No.: +233 3220 60351<br>Mobile: +233 20 196 4425<br>Fax No.: +233 3220 62017<br>Email: owusudabo@kccr.de
**Principal Investigator:**	**Dr. Alexander Yaw Debrah** (BSc. [Hons], M'Phil, PhD)<br>Faculty of Allied Health Sciences<br>Kwame Nkrumah University of Science and Technology<br>Kumasi<br>Ghana<br>Tel. No.: +233 3220 60351<br>Mobile: +233 209341317<br>Fax No.: +233 3220 62017<br>Email: yadebrah@yahoo.com
**Investigator/Head of research team and field laboratory:**	**Dr. Linda Batsa Debrah** (PhD)<br>Kumasi Centre for Collaborative Research (KCCR)<br>Kwame Nkrumah University of Science and Technology<br>Kumasi<br>Ghana<br>Tel. No.: +233 3220 60351<br>Mobile: +233 208174244<br>Fax No.: +233 3220 62017<br>Email: lindrousy@yahoo.com
**Sponsor's medical expert (Trial Clinician, Co-investigator):**	**Dr. Nana Kwame Ayisi-Boateng** (MD)<br>University Hospital of Kwame Nkrumah University of Science and Technology<br>Kumasi<br>Ghana<br>Tel. No.: +233 3220 60320<br>Mobile: +233 507171650<br>Email: ayisi31@gmail.com
**Study pediatrician**	**Dr. Anthony Enimil (MD)**<br>Komfo Anokye Teaching Hospital<br>KNUST Medical School<br>Kumasi<br>Ghana<br>Mobile: +233 208164433<br>Email:tenimil@live.com

**Pharmacist:**	**Dr. Stephen Yao Gbedema**<br>Faculty of Pharmacy<br>Kwame Nkrumah University of Science and Technology (KNUST)<br>Kumasi<br>Ghana<br>Tel. No.: + 233 3220 60374<br>Mobile: +233 243301676<br>Email: sgbedema@yahoo.com
**Project Manager:**	**Francis Adjei Osei**<br>Kumasi Centre for Collaborative Research (KCCR)<br>Kwame Nkrumah University of Science and Technology<br>Kumasi<br>Ghana<br>Tel. No.: +233 3220 60320<br>Mobile: +233 248168933<br>Fax No.: +233 3220 62017<br>Email: francisph1@hotmail.com
**Monitoring Ghana:**	**Carl Kwame Adoku**<br>P.O. Box CO 2546<br>Tema<br>Ghana<br>Tel: +233 248991647<br>Email: carl.adoku@gmail.com
**Local hospital/ laboratory**	Navrongo War Memorial District Hospital, Navrongo
**Sponsor central laboratory:**	**Kumasi Centre for Collaborative Research (KCCR)**<br>Kwame Nkrumah University of Science and Technology<br>Kumasi<br>Ghana
**GHS-ERC Administrator**	**Hannah Frimpong**<br>Ghana Health Ethics Committee<br>Research and Development Division<br>GHS, Accra<br>Tel: +233 507041223

1.2 Trial Administration structure - Germany

**Senior Coordinating Physician:**	**Prof. Dr. Achim Hoerauf (MD)**<br>Institute for Medical Microbiology, Immunology and Parasitology (IMMIP)<br>University Hospital of Bonn<br>Sigmund-Freud-Str. 25<br>53127 Bonn<br>Germany<br>Tel. No.: + 49 228 287-15673 or -15675<br>Fax No.: + 49 228 287-19573<br>Email: achim.hoerauf@ ukbonn.de
**Trial Clinician:**	**Dr. Inge Kroidl** (MD)<br>Department for Infectious Diseases & Tropical Medicine<br>Klinikum of the University of Munich, LMU<br>Leopoldstrasse 5<br>80802 Munich, Germany<br>Tel. No.: +49 89 2180 17637<br>Mobile: +49 1721815933<br>Fax: +49 89 33 6038<br>Email: ikroidl@lrz.uni-muenchen.de
**Project Manager:**	**Dr. Ute Klarmann-Schulz** (MD)<br>Institute for Medical Microbiology, Immunology and Parasitology (IMMIP)<br>University Hospital of Bonn<br>Sigmund-Freud-Str. 25<br>53127 Bonn<br>Germany<br>Tel. No.: + 49 228 287-14612<br>Mobile: + 49 162 9425822<br>Fax No.: + 49 228 287-19573<br>Email: ute.klarmann-schulz@ukbonn.de
**Statistician / Biometrician:**	**Dr. Rolf Fimmers** (PhD)<br>Institute for Medical Biometry, Informatics and Epidemiology (IMBIE)<br>Clinical Study Core Unit, Study Center Bonn (SZB)<br>University Hospital of Bonn<br>Sigmund-Freud-Str. 25<br>53127 Bonn<br>Germany<br>Tel. No.: + 49 228 287-16665<br>Fax No.: + 49 228 287-11324<br>Email: rolf.fimmers@ukbonn.de
**Data Management:**	**Arcangelo Ricchiuto**<br>Institute for Medical Microbiology, Immunology and Parasitology (IMMIP)<br>Clinical Study Core Unit, Study Center Bonn (SZB)<br>University Hospital of Bonn<br>Sigmund-Freud-Str. 25<br>53127 Bonn<br>Germany<br>Tel. No.: + 49 228 287-16482<br>Fax No.: + 49 228 287-11324<br>Email: arcangelo.ricchiuto@ukbonn.de

**Drug safety / Pharmacovigilance:**	Clinical Study Core Unit, Study Center Bonn (SZB)<br>Institute of Clinical Chemistry and Clinical Pharmacology<br>University Hospital of Bonn<br>Sigmund-Freud-Str. 25<br>53127 Bonn<br>Germany<br>Tel. No.: +49 228 287-16040<br>Fax No.: +49 228 287-16039<br>Email: Safety-SZB@ukb.uni-bonn.de

1.3 Funding

Research Networks for Health Innovations in Sub-Saharan Africa

SPONSORED BY THE
Federal Ministry of Education and Research

The design, management, analysis and reporting of the study are entirely independent of the manufacturers of doxycycline and placebo.
The Federal Ministry of Education and Research (BMBF), Germany, has no role in the design of this study and will not have any role during its execution, analyses, interpretation of the data, or decision to submit results.

2. PROTOCOL SYNOPSIS

**Protocol Title**	Doxycycline 200mg/d vs. 100mg/d for 6 weeks to improve filarial lymphedema - a multi-national, double –blind, randomized, placebo-controlled trial
**Phase of trial**	Group A (LE stage 1-3): Phase II (confirmatory)<br>Group B (LE stage 4-6): Phase II (pilot trial)
**Short Title**	Doxycycline to improve filarial lymphedema
**Trial Acronym**	TAKeOFF - LEDoxy
**Protocol Code**	TAKeOFF-4-0117
**Trial registration**	PACTR: PACTR201708002420261<br>ISRCTN: ISRCTN14042737
**Date of trial registration**	PACTR: July 11, 2017<br>ISRCTN: July 17, 2017
**Sponsor**	KCCR, Ghana<br>NIMR, Tanzania
**Source(s) of monetary or material support**	
**Health condition**	Lymphatic filariasis (LF)
**Study Centres**	Multinational, 2 trials in 2 African countries:<br>Ghana (KCCR)<br>Tanzania (NIMR)<br>Both trials will be carried out independently, this study protocol is for the Ghanaian trial only.
**Interventions**	**Treatment "DOX 200"**<br>**Doxycycline 200mg/d for 6 weeks** on top of standard MDA (ivermectin 200µg/kg plus albendazole 400mg once a year)<br><br>**Treatment "Placebo"** (control):<br>**Placebo matching Doxycycline for 6 weeks** on top of standard MDA (ivermectin 200µg/kg plus albendazole 400mg once a year)<br><br>**Treatment "DOX 100"** (additional arm for group A (LE stage 1-3):<br>**Doxycycline 100mg/d for 6 weeks** on top of standard MDA (ivermectin 200µg/kg plus albendazole 400mg once a year)<br><br>Treatment will be administered ad personam by the trial clinician directly in the villages in the form of daily observed treatment (DOT)
**Investigational medicinal product, Dose and Mode of Application**	**Treatment "DOX 200"**:<br>Trade Name: Remycin®<br>Substance: Doxycycline<br>Manufacturer: Remedica, Cyprus<br>Dose: 2 tablets á 100mg/day<br>Mode of application: orally

	Duration of treatment: 6 weeks (42 days)<br>On/off label use: off label (not registered as drug for the treatment of filariasis, but efficacy has been shown in two previous RCTs [1, 2])<br><br>**Treatment "Placebo"** (control):<br>Substance: Placebo matching doxycycline 100mg containing no active ingredients<br>Manufacturer: Piramal, UK<br>Dose: 2 tablets<br>Mode of application: orally<br>Duration of treatment: 6 weeks (42 days)<br><br>**Treatment "DOX 100"** (additional arm for group A (LE stage 1-3)):<br>Trade Name: Remycin®<br>Substance: Doxycycline<br>Manufacturer: Remedica, Cyprus<br>Dose: 1 tablet doxycycline á 100mg/day plus 1 tablet placebo matching doxycycline 100mg/day<br>Mode of application: orally<br>Duration of treatment: 6 weeks (42 days)<br>On/off label use: off label (not registered as drug for the treatment of filariasis)<br><br>Verum and Placebos will be blistered and labelled by Piramal Healthcare, Morpeth, UK.
**Trial Population**	Healthy adolescents or adults (14 – 65 years) with filarial lymphedema (LE, *Wuchereria bancrofti*).<br>Group A: LE stage 1-3<br>Group B: LE stage 4-6<br>Staging will be carried out by using the 7-point scale published by G. Dreyer, 2002 [3].
**Trial Design**	Interventional<br>Method of allocation: randomized<br>Masking: double-blind (caregiver, participants, investigator, outcome assessors)<br>Control: placebo-controlled<br>Assignment: parallel<br>Purpose: efficacy
**Rationale of the trial**	The previously demonstrated [1, 2] effect of doxycycline in reversing or stopping the progression of lymphedema of patients with stage 1-3, irrespective of their filarial infections being active or not, provides an opportunity to include the drug as a new tool in LF morbidity management programs. However, before recommendations can be made regarding the frequency of its usage or alternate dosing patterns the findings of the two RCTs should be replicated in other settings. This multi-national trial is designed to show efficacy of a lower dosage of

	doxycycline (100 mg instead of 200 mg) and to confirm the findings of the lone study that in patients with stages 1-3 lymphedema [3] irrespective of active LF infection (group A), as well as in people with higher grades of lymphedema (stage 4-6, group B).
**Trial Objectives**	**Primary Objectives:**<br>Group A (LE stage 1-3): - to confirm the efficacy of a 6-week course of daily doxycycline 200mg on lack of progression of filarial LE<br>- to reduce the dosage of doxycycline from 200mg/d to 100mg/d for 6 weeks for the treatment of filarial LE<br>Group B (LE stage 4-6): - to show efficacy of a 6-week course of daily doxycycline 200mg on lack of progression of filarial LE<br>**Secondary Objectives (group A <u>and</u> group B):**<br>• to show efficacy on improvement of filarial LE<br>• to evaluate changes in the circumference of the affected limbs measured with tape<br>• to evaluate changes of skin thickness of the affected limbs measured by ultrasound<br>• to evaluate changes in the circumference of the affected limbs measured with an infrared scanner (LymphaTech®)<br>• to evaluate changes in the volume of the affected limbs measured with an infrared scanner (LymphaTech®)<br>• to evaluate changes of the frequency of acute attacks of dermatolymphangioadenitis (ADLA)<br>• to evaluate changes in hygiene behaviour<br>• to evaluate changes in the quality of life (QoL)<br>• to measure biomarkers (angiogenic, lymphangiogenic, pro-fibrotic or pro-inflammatory such as VEGF, CECAM-a, MMPS) in blood and/or urine that are responsible for the progression of LE<br>• to measure T cell activation and differentiation marker in blood such as HLADR, Ki67 and CD38 (activation), PD-1, CTLA-4, Eomes (exhaustion), CD45RA, CD27, CCR7 (differentiation) on CD4 and CD8 T cells<br>• to assess the tolerability and safety of doxycycline 200mg/d for 6 weeks<br>• to assess the tolerability and safety of doxycycline 100mg/d for 6 weeks (group A only)
**Trial Endpoints**	**Primary efficacy endpoints**:<br>• Lack of progression of LE (stage reduction or same stage as pre-treatment using the 7-point scale staging according to Dreyer [3]) examined 24 months after treatment onset<br>**Secondary endpoint(s):**<br>• Lack of progression of LE (stage reduction or same stage as pre-

	treatment using the 7-point scale staging according to Dreyer [3]) examined 6 or 12 months after treatment onset<br>• Improvement of LE, i.e. stage reduction (at least one stage compared to pre-treatment) examined 6, 12 and 24 months after treatment onset<br>• Change of LE stages (reduction or increase) compared to baseline assessed at 6, 12 and 24 months after treatment onset<br>• Changes (reduction or increase) of the circumference of the affected limbs compared to baseline circumferences measured by tape measure at 6, 12 and 24 months after treatment onset<br>• Changes of skin thickness of the affected limbs compared to baseline values measured by ultrasound at 6, 12 and 24 months after treatment onset<br>• Changes of the circumference of the affected limbs compared to baseline circumferences measured with an infrared scanner (LymphaTech®) at 6, 12 and 24 months after treatment onset<br>• Changes of the volume of the affected limbs compared to baseline volume measured with an infrared scanner (LymphaTech®) at 6, 12 and 24 months after treatment onset<br>• Changes in the duration of acute attacks compared to pre-treatment evaluated at 6, 12 and 24 months after treatment onset<br>• Changes in the frequency of acute attacks compared to pre-treatment evaluated at 6, 12 and 24 months after treatment onset<br>• Absence of acute attacks evaluated at 6, 12 and 24 months after treatment onset<br>• Changes of the hygiene level compared to pre-treatment assessed at 6, 12 and 24 months<br>• Changes of the quality of life (QoL) compared to pre-treatment assessed at 12 and 24 months after treatment onset<br>• Changes in levels of angiogenic, lymphangiogenic, pro-fibrotic or pro-inflammatory biomarkers (such as VEGF, CECAM-a, MMPS) in blood and/or urine compared to baseline values as a measure for prognostic effects assessed 6, 12 and 24 months after treatment onset<br>• Changes of T cell activation and differentiation marker in the blood such as HLADR, Ki67 and CD38 (activation), PD-1, CTLA-4, Eomes (exhaustion), CD45RA, CD27, CCR7 (differentiation) on CD4 and CD8 T cells compared to baseline levels measured at 6, 12 and 24 months after treatment onset<br>**Assessment of safety**:<br>Adverse events (AE) will be assessed and described in the scope of the daily observed treatment (DOT). This involves a) occurrence of AE, b) intensity of AE (Grade 0 (none), Grade 1 (mild), grade 2 (moderate) grade 3 (severe), c) SAE, d) relation to treatment (definite, probable, possible, remote, not related), e) outcome of AE (restored, improved, unchanged, deteriorated, death, unknown, overcome with sequelae, f)

	intervention
**Subject Number**	To be assessed for eligibility: n ≈ 1500 per study site (Ghana, Tanzania)<br>To be allocated to trial (meeting inclusion criteria):<br>Group A: n = 360 (n = 120 per intervention group and study site)<br>Group B: n = 60 (n = 30 per intervention group and study site)<br>To be analysed (assuming a drop-out rate of 30%):<br>Group A: n ≈ 252 (n ≈ 84 per intervention group and study site)<br>Group B: n ≈ 42 (n ≈ 21 per intervention group and study site)
**Inclusion Criteria**	• Lymphedema of at least one leg grade 1-6 measured on a 7-point scale [3]<br>• Age ≥ 14 years and ≤ 65 years<br>• Men or non-pregnant women. If women of childbearing-potential, they must use an approved, effective method of contraception (including abstinence) before, during and for at least 2 weeks after the completion of the active intervention with doxycycline or placebo<br>• Negative pregnancy test<br>• Body weight ≥ 40 kg<br>• Resident in LF endemic area for ≥ 2 years<br>• Able and willing to give informed consent/ to provide assent to participate in the trial<br>• Ability to use established standardized methods of hygiene and effectively applying it prior to the initiation of the drug treatment
**Exclusion Criteria**	• No lymphedema or lymphedema stage 7<br>• Age < 14 years or > 65 years<br>• Body weight < 40 kg<br>• Pregnant or breastfeeding women<br>• Women of childbearing potential not using an agreed method of contraception (including abstinence; oral contraceptives are not allowed because of interaction with trial drugs)<br>• Clinical or biologic evidence of hepatic or renal dysfunction or disease of the central nervous system (CNS)<br>• Evidence of severe comorbidities except for features of filarial disease<br>• Alcohol or drug abuse<br>• History of adverse reactions to doxycycline or other tetracyclines<br>• Any significant condition (including medical and psychological/ psychiatric disorder) which in the opinion of the study investigator might interfere with the conduct of the study<br>• History of photosensitivity reactions after taking drugs.<br>• Concomitant medication with antacids containing aluminium, magnesium or sucralfate and not able to discontinue<br>• Concomitant medication with other antibiotics than doxycycline and not able to discontinue

	• Concomitant medication with diuretics or sulfonylurea<br>• Concomitant medication with coumarin
**Laboratory Exclusion Criteria**	• Haemoglobin < 8 g/dL<br>• Neutrophil count <2 000/mm^3<br>• Platelet count <100 000/mm^3<br>• Creatinine > 2 times upper limit of normal<br>• AST (GOT) > 2 times upper limit of normal<br>• ALT (GPT) > 2 times upper limit of normal<br>• γ-GT > 2 times upper limit of normal<br>• Positive urine pregnancy test
**Trial Specific Measurements**	• Laboratory assessments<br>• Lymphedema staging on a 7 point scale as described by G. Dreyer [3]<br>• Circumference of the legs measured by tape<br>• Skin thickness of the ankles measured by ultrasound<br>• Circumference and volume of the legs measured with an infrared scanner (LymphaTech®, Atlanta, Georgia, USA)<br>• ADLA questionnaires<br>• Hygiene assessment<br>• Quality of Life (QoL) assessment
**Statistical Rationale**	Statistical analyses will be done first "intention to treat" (ITT) followed by "per protocol" (PP) to confirm the results.<br>Primary statistical Analysis:<br>The frequencies of the participants with "progression" and their confidence intervals (95%) will be calculated and compared between the treatment groups using Fisher's exact test.<br>For group A: Superiority to placebo has to be proven for DOX 200 before testing superiority to placebo for DOX 100. If both tests show superiority to placebo with $\alpha = 0.05$, non-inferiority of DOX 100 to DOX 200 can be tested in a final step.<br>For group B: DOX 200mg/d will be tested for superiority to placebo with an α-level of 5%.<br>Secondary Endpoints:<br>Secondary endpoints will be described as estimators with confidence intervals (95%) for each intervention group and analyzed with adequate statistical methods.<br>Safety Analysis:<br>Frequency of Adverse events and Serious Adverse Events will be analyzed. In this analysis all patients will be included who took the drugs at least for one day.
**Trial procedures**	There will be 15 visits in total (see schedule of activities):<br>Visit 1: Screening<br>Visit 2: Baseline<br>Visit 3: Treatment (42 days)

	Visit 4: Follow-up 2 months after treatment onset<br>Visit 5: Follow-up 4 months after treatment onset<br>Visit 6: Follow-up 6 months after treatment onset<br>Visit 7: Follow-up 8 months after treatment onset<br>Visit 8: Follow-up 10 months after treatment onset<br>Visit 9: Follow-up 12 months after treatment onset<br>Visit 10: Follow-up 14 months after treatment onset<br>Visit 11: Follow-up 16 months after treatment onset<br>Visit 12: Follow-up 18 months after treatment onset<br>Visit 13: Follow-up 20 months after treatment onset<br>Visit 14: Follow-up 22 months after treatment onset<br>Visit 15: Follow-up 24 months after treatment onset
**Study Timeline**	Estimated date of first participant enrolled: Quarter 01/2018<br>Estimated date of last participant enrolled: Quarter 02/2018<br>Estimated date of last participant completed: Quarter 02/2020<br>Total duration of study:<br>~ 30 months (6 months enrolment period + 6 weeks treatment period + 24 months follow-up after treatment start)

3. SCHEDULE OF ACTIVITIES

	**Visit 1 Screening**	**Visit 2 Baseline**[a]	**Visit 3 Treatment**						
			**Day 1**	**Day 2-21**	**Day 22**	**Day 23-41**	**Day 42**	***Range Days 43-49***	**End of treatment (**one day after treatment no. 42)
Informed Consents/ Assents (clinical trial and biobanking)	√								
Demographic data	√								
Lymphedema staging	√	√							
Clinical photographs		√							
History of lymphedema	√								
History of ADLA	√								
Circumference - Tape		√							
Circumference - Lymphatech®		√							
Volume of LE - Lymphatech®		√							
Ultrasound		√							
Medical history	√								
Concomitant medication	√	√	√	√	√	√	√	*(√)*	√
History of relevant medications	√								
Vital signs	√	√							
Physical examination	√	√							
QoL		√							
Hygiene status		√							
Lymphedema management training		√							
Laboratory assessment (blood)	√	*(√)*[a]	*(√)*[b]		√ (before treatment no. 22, AST/ALT/γ-GT, range +2 days)	*√ before treatment no. 22, AST/ALT/γ-GT, range +2 days)*[c]	√ (on the last day of treatment, AST/ALT/γ-GT, range +2 days)	*√(on the last day of treatment, AST/ALT/γ-GT, range +2 days)*[d]	*(√)*[d]
Urine sample collected	√						√	*(√)*[d]	*(√)*[d]
Saliva sample collected	√								
Pregnancy test	√	√	*(√)*[b]	√ (on day 15, range + 2 days)		√ (on day 29, range + 2 days)	√ (on day 42, range + 2 days)		
In-/Exclusion criteria	√	√	*(√)*[b]						
Randomization		√							
Presence for visit				√	√	√	√	*(√)*	√
Individual treatment			√	√	√	√	√	*(√)*	
ADLA questionnaire		√	√	√	√	√	√	*(√)*	√
AEs				√	√	√	√	*(√)*	√
End of study record									

[a] Visit 2 (Baseline) should take place max. 28 days after visit 1 (Screening). If that is not the case, blood tests have to be repeated. [b]Visit 3 (Treatment) should start on the same day or one day after visit 2 (Baseline). If that is not the case, the pregnancy test has to be repeated and in case the period between the two visits is > 28 days also blood tests and check of in- and exclusion criteria have to be repeated.
[c] only to be done if not already done on day 22 [d] only to be done if not already done on day 42

	**Visit 4 2 months follow-up[a]**	**Visit 5 4 months follow-up[b]**	**Visit 6 6 months follow-up[c]**	**Visit 7 8 months follow-up[d]**	**Visit 8 10 months follow-up[e]**	**Visit 9 12 months follow-up[f]**	**Visit 10 14 months follow-up[g]**	**Visit 11 16 months follow-up[h]**	**Visit 12 18 months follow-up[i]**	**Visit 13 20 months follow-up[j]**	**Visit 14 22 months follow-up[k]**	**Visit 15 24 months follow-up[l]**
Informed Consents/ Assents (clinical trial and biobanking)												
Demographic data												
Lymphedema staging			√			√						√
Clinical photographs			√			√						√
History of lymphedema												
History of ADLA												
Circumference - Tape			√			√						√
Circumference - Lymphatech®			√			√						√
Volume of LE - Lymphatech®			√			√						√
Ultrasound			√			√						√
Medical history												
Concomitant medication	√	√	√	√	√	√	√	√	√	√	√	√
History of relevant medications												
Vital signs			√			√						√
Physical examination												
QoL						√						√
Hygiene status		√	√			√			√			√
Lymphedema management training		√	√			√			√			√
Laboratory assessment (blood)			√			√						√
Urine sample collected			√			√						√
Saliva sample collected			√			√						√
Pregnancy test	√		√			√						√
In-/Exclusion criteria												
Randomization												
Presence for visit	√	√	√	√	√	√	√	√	√	√	√	√
Individual treatment												
ADLA questionnaire	√	√	√	√	√	√	√	√	√	√	√	√
AEs	√	√										
End of study record												√

[a] the 2 months follow-up should take place 61 days (± 7 days) after treatment day 1
[b] the 4 months follow-up should take place 121 days (± 10 days) after treatment day 1
[c] the 6 months follow-up should take place 182 days (± 21 days) after treatment day 1
[d] the 8 months follow-up should take place 243 days (± 14 days) after treatment day 1
[e] the 10 months follow-up should take place 303 days (± 14 days) after treatment day 1
[f] the 12 months follow-up should take place 364 days (± 21 days) after treatment day 1
[g] the 14 months follow-up should take place 425 days (± 21 days) after treatment day 1
[h] the 16 months follow-up should take place 485 days (± 21 days) after treatment day 1
[i] the 18 months follow-up should take place 546 days (± 21 days) after treatment day 1
[j] the 20 months follow-up should take place 607 days (± 21 days) after treatment day 1
[k] the 22 months follow-up should take place 667 days (± 21 days) after treatment day 1
[l] the 24 months follow-up should take place 728 days (-28; + 42 days) after treatment day 1

4. BACKGROUND INFORMATION

Lymphatic filariasis (LF), caused by infection with *Wuchereria bancrofti*, *Brugia malayi* and *Brugia timori* is a major neglected tropical disease (NTD) identified as one of WHO's 17 NTDs, has strong links with poverty and is associated with significant clinical morbidity and social stigma. The common clinical manifestations of the disease are hydrocele and lymphedema (LE). The Global Programme to Eliminate Lymphatic Filariasis (GPELF) was launched in 2000 following the adoption of the World Health Assembly Resolution (WHA 50.29) to eliminate the disease as a public health problem by 2020. The twin goals of the programme are: 1) to reduce microfilaremia (Mf) levels using mass drug administration (MDA) and thereby interrupt transmission and 2) to provide morbidity management to alleviate suffering for those who already have disease [4].

Significant progress has been made since the inception of the programme. By 2013, MDA had been implemented in 60 of the 72 endemic countries and a cumulative 4.9 billion doses of the drugs (albendazole (ALB) in combination with either ivermectin (IVM) or diethylcarbamazine (DEC)) to interrupt transmission had been distributed to 1 billion people [5]. Prior to the start of GPELF it was estimated that there were 91.1 million with Mf, 29.9 million cases of hydrocele and 17.7 million cases of LE [6]. A recent assessment of the progress and impact of the programme indicated that it prevented or cured 96.71 million LF cases (79.2 million Mf carriers, 18.7 million hydrocele cases and a minimum of 5.5 million LE cases) resulting in a 59% reduction of initial LF levels. The greatest decrease (68%) was in the number of individuals with microfilaremia, largely due to the widespread adoption of MDA programmes [6].

The progress in reducing morbidity due to the disease has been less striking. In 2013, only 27 countries had reported morbidity management and disability prevention activities and 13 years after the programme was initiated an estimated 19.43-million hydrocele cases and 16.68 million LE cases still remained. While surgery is the treatment of choice for hydrocele and significant reductions in the number of hydrocele cases can be expected with increased access to surgical treatment of hydrocele, no immediate solutions are available for reducing the number of cases of LE. The framework within GPELF to monitor the implementation and effectiveness of improved hygiene measures within the community is still under development. Even if the goal of interruption of transmission of GPELF is achieved and the development of disease in subsequent generations is blocked, currently affected populations will face a lifetime of progressive disability.

Current treatment practices of LE rely on decreasing the number of acute attacks by improving the hygiene of affected limbs, use of appropriate topical antibiotics and antifungals, exercise, elevation of the limb and use of footwear. While this treatment package has been shown to be effective in halting the progression of LE, it requires sustained access to resources required for limb care and strict adherence to the prescribed procedures.

The anti-*Wolbachia* effects of doxycycline and its potential role in filarial infections have been well documented [7, 8]. Recent observations have suggested a potential role for lymphatic endothelium-derived VEGF-C and sVEGFR3 and other angiopoetic factors in the pathogenesis of LE that could be lowered by doxycycline [1, 9]. A six- week course of doxycycline 200 mg daily (DOX 200) prevented progression of LE in patients with active infection of *W. bancrofti* [1]. More recently, in a trial in Ghana, a similar course of doxycycline (DOX 200) decreased severity of mild to moderate LE independent of active filarial infection [2].

The effect of DOX 200 in reversing or stopping the progression of LE of patients with stage 1-3, irrespective of their filarial infection's being active, provides an opportunity to include the drug as a new tool in LF morbidity management programs. However, before recommendations can be made regarding the frequency of its usage or alternate dosing patterns the findings of the Ghana study need

to be replicated in other settings. This multinational trial is designed to confirm the findings of the lone study that documented the efficacy of DOX 200 treatment in patients with stages 1-3 lymphedema, irrespective of active LF infection, as well people with higher grades of LE.
In total 5 different countries will participate in this trial (Mali, Sri Lanka, India, Ghana and Tanzania). The study sites in Mali (NCT02927496), Sri Lanka (NCT02929134) and India (NCT02929121) are funded by the USAID and carried out in collaboration with the NTD Support Center (Taskforce for Global Health, Atlanta, USA). These three study sites focus on the confirmation of the efficacy of DOX 200 by comparing DOX 200mg for 6 weeks versus Placebo.
In a recent randomized, placebo-controlled, double-blind, phase II, clinical trial carried out in men infected with LF and living adult worms detected by ultrasound ("filarial dance sign" (FDS)), treatment with DOX 100mg/d given for 4 or 5 weeks resulted in > 85% macrofilaricidal activity after 18 months which was as good as the "standard" treatment with DOX 200mg/d for 4 weeks (Klarmann, U. et al., 2012, ASTMH abstract No. 522: https://doi.org/10.4269/ajtmh.2012.87.150). The dosage of 100mg/d is expected to have less, if any, adverse events and is more widely used against a variety of infections than 200mg/d. For example,100 mg/day is equivalent to the dosage recommended for malaria prophylaxis in travelers and for acne. Treatment with DOX 100 would also be less costly for health care providers if it would be administered to all patients affected by LE and it might lead to the involvement of more patients in morbidity programs Therefore the two trials in Ghana and Tanzania will add a third treatment arm to investigate whether reduction of the dosage of doxycycline to 100 mg/day for 6 weeks (DOX 100) has an effect similar or equivalent to 200 mg/day.

5. RATIONALE FOR THE STUDY

Current lymphedema management protocols are based on the use of simple measures of hygiene (regular washing with soap and water, skin and nail care), use of topical antibiotics or antifungal agents, exercise and footwear. Previous controlled clinical trials and extensive field experience have shown the benefit of these measures in reducing the frequency of attacks of acute dermato-lymphangio-adenitis (ADLA) that drive the progression of LE [10]. In most endemic countries they now represent the available "standard of care" in the absence of any structured treatment programs for the management of lymphedema of LF.
In the present study, the progression of LE in a group of patients who receive a six-week course of doxycycline 200mg/d (DOX 200) or doxycycline 100mg/d (DOX 100) will be compared with that of a group who receives doxycycline "look-alike" placebo tablets. However, all three groups will be enrolled into a standardized "regimen of hygiene" described above. Thus, patients enrolled in the "placebo" group also will receive the current "standard of care", and the placebo used in the study will help to identify the benefits of DOX on a background of simple hygiene measures. The regimens will be explained to all participants who will be trained to use established standardized methods of hygiene and be effectively applying it prior to the initiation of the drug treatment. In addition, patients will be retrained at 4, 6, 12, 18 and 24 months. A common, generic SOP with handouts that describes methods and the training schedule will be used so that similar methods are employed across all sites. Additionally, all participants will be encouraged to take part in the annually MDA with ALB 400mg and IVM 200µg/kg.
After un-blinding and data analysis, participants will be offered doxycycline treatment if the intervention proves to be more effective in ameliorating LE.

6. STUDY OBJECTIVES

6.1 Primary Objectives

Group A (LE stage 1-3):

- to confirm the efficacy of a 6-week course of daily doxycycline 200mg on lack of progression of filarial LE
- to reduce the dosage of doxycycline from 200mg/d to 100mg/d for 6 weeks for the treatment of filarial LE

Group B (LE stage 4-6):

- to show efficacy of a 6-week course of daily doxycycline 200mg on lack of progression of filarial LE

6.2 Secondary Objectives

- to show efficacy on improvement of filarial LE
- to evaluate changes in the circumference of the affected limbs measured with tape
- to evaluate changes of skin thickness of the affected limbs measured by ultrasound
- to evaluate changes in the circumference of the affected limbs measured with an infrared scanner (LymphaTech®)
- to evaluate changes in the volume of the affected limbs measured with an infrared scanner (LymphaTech®)
- to evaluate changes of the frequency of acute attacks of adenolymphangioadenitis (ADLA)
- to evaluate changes in hygiene
- to evaluate changes in the quality of life (QoL)
- to measure biomarkers (angiogenic, lymphangiogenic, pro-fibrotic or pro-inflammatory such as VEGF, CECAM-a, MMPS) in blood and/or urine that are responsible for the progression of LE
- to measure T cell activation and differentiation marker in blood such as HLADR, Ki67 and CD38 (activation), PD-1, CTLA-4, Eomes (exhaustion), CD45RA, CD27, CCR7 (differentiation) on CD4 and CD8 T cells
- to assess the tolerability and safety of doxycycline 200mg/d for 6 weeks
- to assess the tolerability and safety of doxycycline 100mg/d for 6 weeks (group A only)

7. STUDY DESIGN

7.1 Study design

For group A (LE stages 1-3):	prospective, multi-national , randomized, placebo-controlled, double-blind (observer, provider, patient), parallel-group (3 groups), interventional phase II trial.
For group B (LE stages 4-6):	prospective, multi-national , randomized, placebo-controlled, double-blind (observer, provider, patient), parallel-group (2 groups), interventional phase II pilot-trial.

7.2 Treatment groups

Group A: Patients with lymphedema stage 1-3 caused by *Wuchereria bancrofti* who meet the inclusion/ exclusion criteria will be invited to participate in the clinical trial and randomized to one of the three following treatment groups:

Treatment "DOX 200"
Doxycycline 200mg/d for 6 weeks on top of standard MDA
(ivermectin 200µg/kg plus albendazole 400mg once a year)

Treatment B "**Placebo**" (control):
Placebo matching Doxycycline for 6 weeks on top of standard MDA
(ivermectin 200µg/kg plus albendazole 400mg once a year)

Treatment "DOX 100" (additional arm for group A (LE stage 1-3):
Doxycycline 100mg/d for 6 weeks on top of standard MDA (ivermectin 200µg/kg plus albendazole 400mg once a year)

Group B: Patients with lymphedema stage 4-6 caused by *Wuchereria bancrofti* who meet the inclusion/ exclusion criteria will be invited to participate in the clinical trial and randomized to one of the three following treatment groups:

Treatment "DOX 200"
Doxycycline 200mg/d for 6 weeks on top of standard MDA
(ivermectin 200µg/kg plus albendazole 400mg once a year)

Treatment "Placebo" (control):
Placebo matching Doxycycline for 6 weeks on top of standard MDA
(ivermectin 200µg/kg plus albendazole 400mg once a year)

7.3 Number of subjects

It is planned to enroll a total a number of 420 participants. Participants will be randomized as follows:

Group A (LE stages 1-3): 120 participants will be randomized to treatment "DOX 200",
120 participants to treatment "Placebo",
120 participants to treatment "DOX 100"

Group B (LE stages 4-6): 30 participants will be randomized to treatment "DOX 200",
30 participants to treatment "Placebo".

7.4 Participating trial sites

7.4.1 Description of the study setting of the trial in Ghana

The trial will be conducted in communities of the Kassena-Nankana East and West Districts in the Upper East Region of Ghana where lymphatic filariasis is endemic. A preliminary survey done in more than 40 communities of the study villages (see Figure 1) showed a CFA prevalence of between 5.0 - 16.3% and about 935 lymphedema patients have been identified from these communities. The district capital of the Kassena-Nankana East District is Navrongo, which is about 25-45 minutes drive

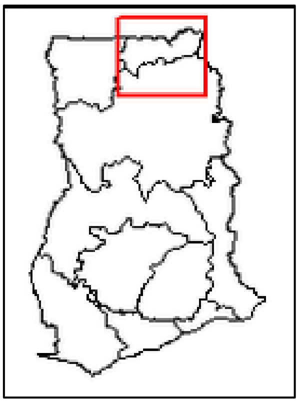

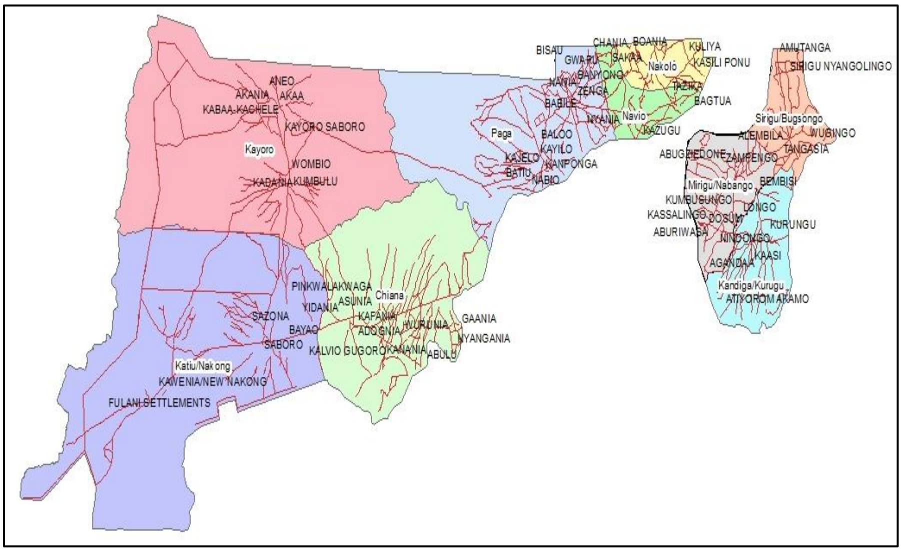

Figure 1.
Trial sites in Ghana

to the study villages where the patients will be recruited. The laboratory where the lab analyses will be done is located in the War Memorial District Hospital in Navrongo and the research team will stay in Bolgatanga during the trial periods for continuous contact and monitoring of the trial participants. Bolgatanga is 30 minutes drive from Navrongo and about 20 minutes drive from some of the study communities.

7.4.2 Description of the study setting of the trial in Tanzania

The trial will be conducted in Lindi region and Pwani region. Lindi is a coastal town located at the far end of the Lindi Bay, on the Indian Ocean in southeastern Tanzania. The town is 450 kilometers south of Dar es Salaam and 105 kilometers north of Mtwara, the southernmost coastal town in Tanzania. Lindi initial LF mapping was conducted 2001 using ICT indicated overall prevalence of 51.83% and 2015- 2016 sentinel site assessment survey (SSA) indicated LF prevalence by FTS of 4.68%.
Pwani region is one of Tanzania's 30 administrative regions. It is bordered to the North by the Tanga region , to the west by the Morogoro region to the east by the Indian ocean and to the south by the Kibaha district. According to the 2012 national census, the region had a population of 1,098,668. The regional capital is the town of Kibaha. Pwani LF initial mapping was conducted from 1998 – 2001 using ICT indicated prevalence of 44.5% and 2015 – 2017 using ICT and FTS indicated overall prevalence of 2.34%.

7.4.3 Description of the study settings of the three USAID funded trials

The effect of a 6-week course doxycycline on lymphedema without active filarial infection has been demonstrated in a single setting in Africa (Ghana). In order to expand the benefits of this observation, similar studies need to be carried out in other endemic settings. Lymphatic filariasis is endemic in many countries of Africa and Asia that are yet to implement morbidity management programmes. In addition, the components of the proposed hygiene package are likely to vary depending on the availability of material and human resources. The conduct of this trial as a multi-national study will not only allow the evaluation of the efficacy of the drug in a variety of settings but also facilitate its rapid adoption by the respective control programmes of endemic countries, if proven to be useful.

The study will be conducted at the following sites comparing doxycycline 200mg for 6 weeks vs. placebo (Treatment C with doxycycline 100mg is not planned for these 3 trial sites):

	Country	Study site	Research Group	Trial registration
Africa	Mali	Sikasso & Koulikoro	ICER-Mali, Filariasis Research Unit	NCT02927496
Asia	India	Ambalappuzha and Cherthala Yaluks of Alappuzha District, Kerala	Filariasis Research Unit, Govt., T.D. Medical College, Alappuzha, Kerala	NCT02929121
Asia	Sri Lanka	Pohlena, Walgama and Madihe suburbs, Galle	FRTSU, Faculty of Medicine, Univ. of Ruhuna, Galle	NCT02929134

The choice of study sites in endemic countries has been made based on the availability of a) adequate numbers of patients with various grades of lymphedema and b) clinical trial teams familiar with lymphedema management procedures and past experience with similar trials.

7.4.4 Overall timeline of the study

Enrolment: 6 - 9 months

Treatment: 2 - 3 months

Follow-ups will take place every two months after treatment onset until the 24 months follow-up (see chapter 3 “Schedule of activities”).

Estimated date of first participant enrolled: Quarter 01/2018
Estimated date of last participant enrolled: Quarter 02/2018 (Ghana)
Estimated date of last participant completed: Quarter 02/2020 (Ghana)
Total duration of study:
~ 30 months (6 months enrolment period + 6 weeks treatment period + 24 months follow-up after treatment start)

Expected duration of a subjects participation: ~ 26 months

7.5 Recruitment

7.5.1 Recruitment procedure in Ghana

Recruitment will be carried out directly in the villages. Before start of recruitment the research team will visit the village elders to explain the planned study in detail. At the next visit all villagers will be invited to come to a public meeting where the study will again be explained in detail. After this meeting all interested volunteers, who are between 14 and 65 years old and signed the Informed Consent Form for screening, will be invited for the first screening which will be carried out by trained members of the research team under the supervision of Investigators. A medical doctor will be present to do the physical examination and to take the medical history of the volunteers. The blood sampling will be done by trained and qualified research personnel.

The screening will be carried out directly in the villages. The research team will use rooms provided by the village or will bring tents that guarantee the privacy of the volunteers during examination. In communities where CHPs premises are present examinations will be done in the CHPs premises.

All volunteers who proved to be eligible for the trial during the screening visit will be invited to come to the enrolment which will also be carried out directly in the village. During enrolment lymphedema specific examinations will be carried out. If the participant is still eligible for the trial after enrolment he/she will be randomized.

Screen failures will be encouraged to take part in the regular MDA ("standard of care") and to carry out intensive hygiene. In case volunteers are excluded before study start because of an illness that has to be treated they will be referred to the next hospital.

7.6 Bias reducing measures

7.6.1 Randomization

The randomization lists will be generated by the manufacturer of the study drugs (Piramal, UK) using block randomization. Patients with LE stage 1-3 (Group A: N = 360) and LE stage 4-6 (Group B: N = 60) will be randomized separately. Consenting subjects will be allocated to treatment sequentially according to the randomization list with treatment allocation being provided in individual envelopes.

7.6.2 Blinding

At each trial site, trial participants, care providers, and outcome assessors as well as the data management located in Bonn, Germany will be blinded to the drugs received by the patients.

7.7 Study Endpoints

7.7.1 Primary Endpoint

- Lack of progression of LE (stage reduction or same stage as pre-treatment using the 7-point scale staging according to Dreyer [3]) examined 24 months after treatment onset

7.7.2 Secondary Endpoints

- Lack of progression of LE (stage reduction or same stage as pre-treatment using the 7-point scale staging according to Dreyer [3]) examined 6 or 12 months after treatment onset
- Improvement of LE, i.e. stage reduction (at least one stage compared to pre-treatment) examined 6, 12 and 24 months after treatment onset
- Change of LE stages (reduction or increase) compared to baseline assessed at 6, 12 and 24 months after treatment onset
- Changes of the circumference of the affected limbs compared to baseline circumferences measured by tape measure at 6, 12 and 24 months after treatment onset
- Changes of skin thickness of the affected limbs compared to baseline values measured by ultrasound at 6, 12 and 24 months after treatment onset
- Changes of the circumference of the affected limbs compared to baseline circumferences measured with an infrared scanner (LymphaTech®) at 6, 12 and 24 months after treatment onset
- Changes of the volume of the affected limbs compared to baseline circumferences measured with an infrared scanner (LymphaTech®) at 6, 12 and 24 months after treatment onset
- Changes in the frequency of acute attacks compared to pre-treatment evaluated at 6, 12 and 24 months after treatment onset
- Changes in the duration of acute attacks compared to pre-treatment evaluated at 6, 12 and 24 months after treatment onset
- Absence of acute attacks evaluated at 6, 12 and 24 months after treatment onset
- Changes of the hygiene level compared to pre-treatment assessed at 6, 12 and 24 months
- Changes of the quality of life (QoL) compared to pre-treatment assessed at 12 and 24 months after treatment onset

- Changes in levels of angiogenic, lymphangiogenic, pro-fibrotic or pro-inflammatory biomarkers (such as VEGF, CECAM-a, MMPS) in blood and/or urine compared to baseline values as a measure for prognostic effects assessed 6, 12 and 24 months after treatment onset
- Changes of T cell activation and differentiation marker in the blood such as HLADR, Ki67 and CD38 (activation), PD-1, CTLA-4, Eomes (exhaustion), CD45RA, CD27, CCR7 (differentiation) on CD4 and CD8 T cells compared to baseline levels measured at 6, 12 and 24 months after treatment onset

7.7.3 Assessment of safety

Adverse events (AE) will be assessed and described in the scope of the daily observed treatment (DOT). This involves a) occurrence of AE, b) intensity of AE (Grade 0 (none), Grade 1 (mild), grade 2 (moderate) grade 3 (severe), c) SAE, d) relation to treatment (definite, probable, possible, remote, not related), e) outcome of AE (restored, improved, unchanged, deteriorated, death, unknown, overcome with sequelae, f) intervention.

7.8 Study Population and Eligibility Criteria

This trial can fulfill its objectives only if appropriate participants are enrolled. The following eligibility criteria are designed to select subjects for whom protocol treatment is considered appropriate. All relevant medical and non-medical conditions will be taken into consideration when deciding whether this protocol is suitable for a particular participant.
Only patients with LE due to LF will be included in this trial. Children < 14 years and persons not capable of giving adequately informed consent will not be included.

7.8.1 Gender Distribution

No gender ratio has been stipulated in this trial as the results of preclinical and clinical studies or medical literature did not indicate any difference in the effect of the trial treatment in terms of efficacy and safety.

7.8.2 Inclusion criteria

Patients eligible for the trial must comply with all of the following at **randomization**:

1. Lymphedema of at least one leg Grade 1-6 measured on a 7-point scale [3]
2. Age ≥ 14 years and ≤ 65 years
3. Men or non-pregnant women. If women of childbearing-potential, they must use an approved, effective method of contraception (including abstinence) before, during and for at least 2 weeks after the completion of the active intervention with doxycycline or placebo
4. Negative pregnancy test
5. Body weight ≥ 40 kg
6. Resident in LF endemic area for ≥ 2 years
7. Able and willing to give informed consent to participate in the trial
8. Ability to use established standardized methods of hygiene and effectively applying it prior to the initiation of the drug treatment

7.8.3 Exclusion criteria

Patients are ineligible to participate in the trial, if they have any of the following:

1. No lymphedema or lymphedema stage 7
2. Age < 14 years or > 65 years
3. Body weight < 40 kg
4. Pregnant or breastfeeding women

5. Women of childbearing potential not using an agreed method of contraception (including abstinence; oral contraceptives are not allowed because of interaction with trial drugs)
6. Clinical or biologic evidence of hepatic or renal dysfunction or disease of the central nervous system (CNS)
7. Evidence of severe comorbidities except for features of filarial disease
8. Alcohol or drug abuse
9. History of adverse reactions to doxycycline or other tetracyclines
10. Any significant condition (including medical and psychological/ psychiatric disorder) which in the opinion of the study investigator might interfere with the conduct of the study
11. History of photosensitivity reactions after taking drugs.
12. Concomitant medication with antacids containing aluminium, magnesium or sucralfate and not able to discontinue
13. Concomitant medication with other antibiotics than doxycycline and not able to discontinue
14. Concomitant medication with diuretics or sulfonylurea
15. Concomitant medication with coumarin

Laboratory values which will lead to exclusion

1. Haemoglobin < 8 g/dL
2. Neutrophil count <2 000/mm^3
3. Platelet count <100 000/mm^3
4. Creatinine > 2 times upper limit of normal
5. AST (GOT) > 2 times upper limit of normal
6. ALT (GPT) > 2 times upper limit of normal
7. y-GT > 2 times upper limit of normal
8. Positive urine pregnancy test

7.8.4 Justification for exclusion of women and children

7.8.4.1 Pregnant Women

Pregnant and/or breastfeeding women will be excluded because doxycycline is contraindicated in pregnancy and during breastfeeding. However, non-pregnant women of childbearing-potential using an approved, effective method of contraception (including abstinence, excluding oral contraceptives because of interaction with trial drugs) before, during and for at least 2 weeks after the completion of the active intervention with doxycycline or placebo are eligible for inclusion. Approved methods of contraception such as implants, intrauterine device (IUD), condoms etc. which are practiced in Ghana would be used.

7.8.4.2 Children

Those less than 14 years of age will not be able to participate because of potential effects on bone development and discoloration of teeth by doxycycline in children.

8. TRIAL PROCEDURES

8.1 Clinical Evaluation

8.1.1 Medical history and physical examination

A complete medical history and physical examination will be performed as part of the baseline evaluation. Subsequent clinical evaluations will focus on the assessment of new symptoms, signs or untoward medical events. Vital signs, including blood pressure, pulse rate, and body temperature will be measured as part of all physical examinations, according to standard nursing practice.

8.1.2 Staging of lymphedema

Staging of LE will be done using the 7 stage classification described by Dreyer et al. [3]. The following procedure for comparisons of LE staging in patients with either one or both legs affected will be used: (a) if only one leg has LE, this leg will be analyzed (b) if both legs are affected, one leg with stage 1-3 and the other leg with stage 4-7, the leg with the lower stage will be chosen for analysis (c) if both legs are affected with stage 1-3, the leg with the higher stage will be chosen for analysis (d) if both legs are affected with stage 4-6, the leg with the lower stage will be chosen for analysis (e) if both legs are affected equally (same stage) one of them will be chosen randomly for analysis.

8.1.3 Assessment of acute dermatolymphangioadenitis (ADLA)

A questionnaire regarding the occurrence of ADLA episodes will be carried out at every contact.

8.1.4 Circumference measurement of legs using a tape measure

Leg circumferences will be measured using a tape measure. Circumference measurements will be made at 10 cm posterior to the tip of the large toe and 12, 20, and 30 cm from the sole of the foot, at least twice as described elsewhere. Averages of the 4 measurements will be determined before treatment and at follow-up.

8.1.5 Circumference measurement of legs using an infrared scanner (LymphaTech®)

A portable infrared scanner has recently been developed (LymphaTech scanner) that can quickly and accurately measure limb circumferences and volumes. This scanner will be used at baseline, 6, 12 and 24 months to measure the circumferences of both legs at 12, 20 and 30 cm. Measurements will be done twice for each leg.

8.1.6 Assessment of the leg volume using an infrared scanner (LymphaTech®)

A portable infrared scanner has recently been developed (LymphaTech scanner) that can quickly and accurately measure limb circumferences and volumes. This scanner will be used at baseline, 6, 12 and 24 months to measure the volume of both legs below 32cm. Measurements will be done twice for each leg.

8.1.7 Training for care and hygiene of affected legs and arms

All patients will be initiated to a programme of cleaning of the affected limb based on the principles outlined in the booklet "New Hope" for persons with lymphedema [3]. The regimens will be explained to all participants who will be trained to use established standardized methods of hygiene and be effectively applying it prior to the initiation of the drug treatment. Each patient will receive soap, towels and plastic bowls for washing the limbs and a diary for recording ADLA attacks. Community Health Volunteers will be engaged to assist persons who cannot read and write to record the ADLA attacks in the diary as done in our previous study [2]. Supplies will be replenished during the follow-up visits, if necessary.

A generic SOP with handouts that describes methods and the training schedule will be utilized so that similar methods are employed across all sites. In addition, patients will be retrained at 4, 6, 12, 18 and 24 months. This will be standardized by the investigators and will include the following:

1. Cleaning of the affected limb daily with soap and water
2. Keeping the affected limb dry
3. Clipping the nails
4. Appropriate antibiotics for ADLA episodes
5. Applying antifungal ointment to webs of the toes, nails and sides of the feet every night
6. Elevation of the affected extremity
7. Limb exercises as instructed
8. Encouraging and monitoring the use of appropriate footwear

8.1.8 Ultrasound

Ultrasonography of the legs to assess lymphedema will be performed as described by Mand et al. [2]. Briefly, measurements will be taken in both legs over the lateral and medial malleoli of each ankle. A generic SOP that will enable the examiner to get reliable and reproducible results and will make comparisons possible between different examiners, different time points and the different trial sites, will be used so that the same method is employed across all sites.

8.1.9 Clinical photographs

Digital clinical photographs of the affected and normal limb will be taken baseline and at the follow-up time points at 6, 12 and 24 months. The distance and lighting will be standardized for each site by the investigator and all efforts to ensure comparability will be taken at each site. The photographs will be stored as digital images and made available for analysis.

8.1.10 Quality of life (QOL) assessment

QOL assessments will be performed at baseline, 12 and 24 months using the 12-item questionnaire WHODAS 2.0 [11], a generic health and disability assessment tool grounded in the conceptual framework of the ICF and capturing an individual’s level of functioning in six major life domains: (i) cognition (understanding and communication); (ii) mobility (ability to move and get around); (iii) self-care (ability to attend to personal hygiene, dressing and eating, and to live alone); (iv) getting along (ability to interact with other people); (v) life activities (ability to carry out responsibilities at home, work and school); (vi) participation in society (ability to engage in community, civil and recreational activities). The questionnaire will be administered using translations in the local language by trained personnel.

8.2 Laboratory Evaluation

8.2.1 Blood

Blood samples with volumes between 5 – 25 ml will be taken during screening (24 ml), before treatment no. 22 (4.9 ml) and after treatment no. 42 (10.4 ml) as well as 6, 12 and 24 months after treatment onset (19.9 ml, respectively) to do the assessments described below as well as in chapter 8.3.

8.2.1.1 Hematology and biochemistry

At baseline venipuncture will be performed for assessing the full bloodcell count, transaminases, creatinine and bilirubin. Transaminases will also be checked before treatment no. 22 and on the last day of treatment (after treatment no. 42).

8.2.1.2 Filarial specific measurements

Active filarial infection will be determined by using the Filarial Test Strip (FTS) In participants positive for FTS, Microfilariae will be counted using a Sedgewick chamber. Fliarial specific measurements will be done baseline as well as 6, 12 and 24 months after treatment onset.

8.2.1.3 Biomarkers in blood

Angiogenic, lymphangiogenic, pro-fibrotic or pro-inflammatory biomarkers such as VEGF, CECAM-a, MMPS are important in lymphangiogenesis and lymphatic filarial infections. Biomarkers will be assessed at baseline, at treatment end as well as 6, 12 and 24 months after treatment start.

8.2.1.4 T cell activation and differentiation marker

T cell activation and differentiation marker such as HLADR, Ki67 and CD38 (activation), PD-1, CTLA-4, Eomes (exhaustion), CD45RA, CD27, CCR7 (differentiation) on CD4 and CD8 T cells will be assessed at baseline as well as 6, 12 and 24 months after treatment start.

8.2.2 Urine

8.2.2.1 Urine dipstick

Urine samples will be collected in clean pots labeled with the barcodes of each study participant for routine urine examination using a dipstick method.

8.2.2.2 Urine pregnancy tests

Pregnancy tests in urine will be carried out during screening, at baseline, prior to the first treatment and after 14, 28 and 42 days of treatment as well as at the 2, 6, 12 and 24 months follow-ups. The HCG accurate pregnancy test kit (Registration number FDB/D16-11170) will be used to carry out these examinations.

8.2.2.3 Biomarkers in urine

Biomarkers such as NATOG and miRNAs will be tested in urine at baseline, treatment end, as well as 6, 12 and 24 months after treatment onset.

8.2.3 Saliva

Saliva samples (2 ml) will be taken baseline and at 6, 12 and 24 months. The saliva samples will be used to look at different biomarkers to develop new diagnostic tools for LF without having to draw blood.

8.3 Schedule of Events

The study consists of the following visits (see also chapter 3 " Schedule of activities"):

Visit 1:	Screening Visit
Visit 2:	Enrolment Visit (max. 28 days after screening)
Visit 3:	Treatment
	Treatment start (Day 1) - Daily observed treatment with 42 doses of Doxycycline - Treatment end (Day 43 + max. 7 days)
Visit 4:	2 months follow-up (Day 61 ± 7 days)
Visit 5:	4 months follow-up (4 month, Day 121 ± 10 days)
Visit 6:	6 months follow-up (6 month, Day 182 ± 21 days)
Visit 7:	8 months follow-up (8 month, Day 243 ± 14 days)
Visit 8:	10 months follow-up (10 month, Day 303 ± 14 days)
Visit 9:	12 months follow-up (12 month, Day 364 ± 21 days)
Visit 10:	14 months follow-up (14 month, Day 425 ± 21 days)
Visit 11:	16 months follow-up (16 month, Day 485 ± 21 days)

Visit 12: 18 months follow-up (18 month, Day 546 ± 21 days)
Visit 13: 20 months follow-up (20 month, Day 607 ± 21 days)
Visit 14: 22 months follow-up (22 month, Day 667 ± 21 days)
Visit 15: 24 months follow-up (24 month, Day 728 -28/+48 days)

8.3.1 Screening (Visit 1)

Members of the research team will identify potential subjects. The following assessments/ procedures will be performed during screening:

- Informed consent for screening procedures
- Demographic data
- Lymphedema staging
- History of lymphedema
- History of acute dermatolymphangioadenitis (ADLA)
- Medical history
- Concomitant medication
- History of trial relevant medication
- Physical examination
- Vital signs
- Laboratory assessment (24 ml of blood): Full blood cell count, Liver Function Tests (AST/ALT/γ-GT), Serum Creatinine, FTS, Microfilariae count (Sedgewick, in FTS positive participants only), biomarkers, T-cell activation markers
- Urine sampling (max. 25 ml)
- Urine pregnancy test (for women of childbearing potential). In addition, women of childbearing potential will be counseled on avoiding pregnancy during the treatment period in a culturally appropriate manner as defined by the local IRB.
- Saliva sampling (2 ml)
- Assessment of Inclusion/Exclusion criteria

8.3.2 Enrolment (Visit 2)

Baseline assessments will include:

- Informed consent for trial
- Concomitant medication
- Physical examination:
- Vital signs
- ADLA questionnaire
- Lymphedema staging
- Circumference of lymphedema - Tape measurement
- Circumference of lymphedema – Infrared scanner (LymphaTech®)
- Volume of lymphedema - Infrared scanner (LymphaTech®)
- Clinical photographs of the affected and normal limbs
- Hygiene status
- Lymphedema management training
- Ultrasound assessment of skin thickness at both ankles
- Quality of life (QOL) questionnaire

- Urine pregnancy test (for women of childbearing potential) In addition, women of childbearing potential will be counseled on avoiding pregnancy during the treatment period in a culturally appropriate manner as defined by the local IRB.
- Randomization

8.3.3 Treatment (Visit 3, day 1 – day 43 + 7 days range)

8.3.3.1 Treatment day 1

- Urine pregnancy test if not done during visit 2 on the same day or one day before
- Concomitant medication
- Participants will receive the first dose of either doxycycline or placebo under supervision of the trial clinicians at the study sites.

8.3.3.2 Treatment days 2 – 42 (+ 7 days range)

- Subsequent doses of the trial drugs will be administered under supervision of the research team. Participants will be expected to come every day to the meeting point in their village.
- The following assessments will be done during each day of treatment:
    - Documentation of participants presence
    - ADLA questionnaire
    - AE/SAE assessment
    - Concomitant medication
    - Individual treatment
- The following additional assessments will be done on predefined days during the treatment period:
    - After 14 days:
        - Urine pregnancy test (for women of childbearing potential)
    - Before treatment no. 22:
        - Laboratory assessment (4.9 ml of blood): serum transaminases
    - After 28 days:
        - Urine pregnancy test (for women of childbearing potential)
    - After 42 days:
        - Urine pregnancy test (for women of childbearing potential)
    - After treatment no. 42:
        - Laboratory assessment (10.4 ml of blood): serum transaminases, biomarkers
        - Urine sampling

8.3.3.3 End of treatment (day 43 + 7 days range)

One day after the last dose of trial drugs, the following assessments will be done:

- Documentation of participants presence
- ADLA questionnaire
- AE/SAE assessment
- Concomitant medication

8.3.4 2 months follow-up

- Documentation of participants presence
- AE/SAE assessment
- Concomitant medication
- ADLA questionnaire
- Urine pregnancy test (for women of childbearing potential)

8.3.5 4 months follow-up

- Documentation of participants presence
- AE/SAE assessment
- Concomitant medication
- ADLA questionnaire
- Hygiene status
- Lymphedema management training

8.3.6 6 months follow-up

- Documentation of participants presence
- Concomitant medication
- Vital signs
- ADLA questionnaire
- Lymphedema staging
- Circumference of lymphedema - Tape measurement
- Circumference of lymphedema – Infrared scanner (LymphaTech®)
- Volume of lymphedema - Infrared scanner (LymphaTech®)
- Clinical photographs of the affected and normal limbs
- Hygiene status
- Lymphedema management training
- Ultrasound assessment of skin thickness at both ankles
- Laboratory assessment (19.9 ml of blood): FTS, Microfilariae count (Sedgewick, in FTS positive participants only), biomarkers, T-cell activation markers
- Urine sampling
- Urine pregnancy test (for women of childbearing potential)
- Saliva sampling

8.3.7 Follow-ups at 8, 10, 14, 16, 20 and 22 months after the first day of treatment

The following assessments will be done 8, 10, 14, 16, 20 and 22 months after treatment onset:

- Documentation of participants presence
- Concomitant medication
- ADLA questionnaire

8.3.8 18 months follow-up

- Documentation of participants presence
- Concomitant medication
- ADLA questionnaire
- Hygiene status
- Lymphedema management training

8.3.9 12 and 24 months follow-ups

- Documentation of participants presence
- Concomitant medication
- Vital signs
- ADLA questionnaire
- Lymphedema staging

- Circumference of lymphedema - Tape measurement
- Circumference of lymphedema – Infrared scanner (LymphaTech®)
- Volume of lymphedema - Infrared scanner (LymphaTech®)
- Clinical photographs of the affected and normal limbs
- Hygiene status
- Lymphedema management training
- Ultrasound assessment of skin thickness at both ankles
- Quality of life (QOL) questionnaire
- Laboratory assessment (19.9 ml of blood): FTS, Microfilariae count (Sedgewick, in FTS positive participants only), biomarkers, T-cell activation markers
- Urine sampling
- Urine pregnancy test (for women of childbearing potential)
- Saliva sampling

9. INVESTIGATIONAL MEDICINAL PRODUCT (IMP)

9.1 General description of the study drug doxycycline

Doxycycline is an already marketed product for the antibiotic treatment of several infectious diseases. The treatment of lymphedema due to lymphatic filariasis is an off label treatment.

9.2 Specification of IMP

Trade name	Remycin®
Name of manufacturer	Remedica, Cyprus
Substance name (if applicable, give substance code)	Doxycycline
Name and dose of active ingredient per unit	Doxycycline hyclate 100mg
Pharmaceutical form	Tablets, film-coated
Mode of administration	Oral
Storage conditions	Between 20 - 25°C

The matching placebo will be manufactured by Piramal Healthcare, Morpeth, United Kingdom. The same company will also do the labeling and blistering of the trial drugs for all 5 trials at the same time.

9.3 Dosage and Route of Administration

Both doxycycline and placebo will be administered ad personam by the trial clinician directly in the villages under supervision (directly observed treatment, DOT) for 6 weeks.
Doxycycline 200mg (2 tablets á 100mg) will be administered orally once daily for 6 weeks. The dosage will be reduced to 1 tablet á 100mg in participants with a body weight ≥ 40kg and < 50kg.
Doxycycline 100mg (1 tablet á 100mg) combined with one tablet of placebo matching doxycycline 100mg will be administered orally once daily for 6 weeks. This dosage will not have to be reduced for participants with a body weight < 50 kg.
Placebo matching doxycycline 100mg (2 tablets) will be administered orally once daily for 6 weeks.
The trial drugs will be blistered and labeled in a way that will make it possible to cut off one side of the blister for the reduction from two tablets to one tablet per day for participants with a body weight < 50kg. In case of the 100mg doxycycline treatment, the part of the blister with the tablets that will not

be administered will always be the part containing the placebos. This will be ensured by the way the labeling will be placed.
The first dose of doxycycline or placebo will be given after all the investigations have been completed, informed consent has been obtained and the patient has been initiated into the programme of basic hygiene. Patients will be encouraged to eat before swallowing the tablets whole with a glass of water. Vomited doses within 15 minutes after intake will be replaced.

9.4 Findings from clinical studies

Doxycycline is a well known and safe drug used for the treatment of several infectious diseases.
Doxycycline with a dosage of 200mg/d or 100mg/d for 4- 6 weeks was already used in several clinical trials in lymphatic filariasis or onchocerciasis [1, 2, 7, 12-15].

9.5 Summary of known and potential risks of doxycycline

9.5.1 Side effects of doxycycline

Very frequent (> 10%) side effects:
Infections and Infestations: Common cold (22%), influenza symptoms (11%)
Gastrointestinal: Nausea (up to 13.4%)
Nervous system: Headache (up to 26%)

Frequent (1-10%) side effects:
Metabolic and Nutritional: Increased blood lactate dehydrogenase (2%), increased blood glucose (1%)
Respiratory: Nasopharyngitis (5%), sore throat (5%), sinus congestion (5%), coughing (4%), sinus headache (up to 4%), sinusitis (3%), bronchitis (3%), nasal congestion (2%), pharyngolaryngeal pain (1%)
Infections and Infestations: infection (2%), fungal infection (2%), influenza (2%)
Reproductive Disorders: Menstrual cramp (4%), bacterial vaginitis (3.3%), vulvovaginal mycotic infection (2%)
Hepatic and Biliary: Increased aspartate aminotransferase (2%)
Body as a whole – general: Injury (5%), pain (up to 4%), back pain (up to 3%), back ache (2%)
Dermatologic: Rash (4%)
Gastrointestinal: Vomiting (8.1%), toothache (7%), tooth disorder (6%), dyspepsia (6%), diarrhea (up to 6%), periodontal abscess (4%), acid indigestion (4%), upper abdominal pain (2%), abdominal distention (1%), abdominal pain (1%), stomach discomfort (1%), dry mouth (1%)
Musculoskeletal: Joint pain (6%), muscle pain (1%)
Cardiovascular: Hypertension (3%), increased blood pressure (2%)
Psychiatric: Anxiety (2%)

Occasionally (0.1 to < 1%) side effects:
Gastrointestinal: Gum pain

Rare *(< 0.1%) side effects:*
Gastrointestinal: Adult tooth staining (at least 1 case)

Frequency not reported:
Hematologic: Hemolytic anemia, thrombocytopenia, neutropenia, eosinophilia
Metabolic and Nutritional: Hypoglycemia, anorexia
Infections and Infestations: Possible overgrowth of nonsusceptible organisms (superinfection)
Renal and Urinary: Dose-related rise in BUN
Reproductive Disorders: Vaginal itch, vaginal candidiasis

Hepatic and Biliary: Acute hepatocellular injury, cholestatic reactions, hepatotoxicity
Body as a whole – general: Microscopic brown-black discoloration of the thyroid gland
Dermatologic: Nail discoloration, phototoxicity, photoallergic reaction, photo-onycholysis, photosensitivity, maculopapular and erythematous rashes, erythema multiforme, Stevens-Johnson syndrome, toxic epidermal necrolysis, exfoliative dermatitis, hyperpigmentation
Gastrointestinal: Clostridium difficile associated diarrhea, esophageal irritation, ulceration, epigastric burning, black hairy tongue, Esophagitis and esophageal ulcerations (most took medication immediately before going to bed)
Nervous system: Sinus headache, dizziness, drowsiness, amnesia, paresthesias of body areas exposed to sunlight, phrenic nerve paralysis after sclerotherapy, benign intracranial hypertension resulting in significant loss of vision
Ocular: Diplopia, papilledema, loss of vision (associated with doxycycline-induced benign intracranial hypertension)
Hypersensitivity: Hypersensitivity reactions (including urticaria, angioneurotic edema, anaphylaxis, anaphylactoid purpura, serum sickness, pericarditis, exacerbation of systemic lupus erythematosus, drug rash with eosinophilia and systemic symptoms [DRESS])
Immunologic: Autoimmune syndromes

Postmarketing reports:
Nervous system: Pseudotumor cerebri (benign intracranial hypertension), headache

9.5.2 Precautions to mitigate drug specific risks (Doxycycline)

Photosensitivity

Photosensitivity manifested by an exaggerated sunburn reaction has been observed in some individuals taking tetracyclines. Patients apt to be exposed to direct sunlight or ultraviolet light will be advised that this reaction can occur with tetracycline drugs. Treatment will be immediately stopped at the first evidence of skin erythema.

Growth and Development

All tetracyclines form a stable calcium complex in any bone-forming tissue. A decrease in fibula growth rate has been observed in prematures given oral tetracycline in doses of 25 mg/kg every six hours. This reaction was shown to be reversible when the drug was discontinued. Results of animal studies indicate that tetracyclines cross the placenta, are found in fetal tissues, and can have toxic effects on the developing fetus (often related to retardation of skeletal development). Evidence of embryotoxicity also has been noted in animals treated early in pregnancy. If any tetracycline is used during pregnancy or if the patient becomes pregnant while taking these drugs, the patient should be apprised of the potential hazard to the fetus. Therefore women taking part in the clinical trial will be informed in detail about the risk associated with a pregnancy during drug intake. Pregnancy tests will be carried out every week and if a positive pregnancy test occurs against all precautions, treatment will be stopped immediately.

Clostridium difficile –associated diarrhea

Clostridium difficile-associated diarrhea (CDAD) has been reported with the use of nearly all systemic antibacterial agents, including Moxifloxacin, with severity ranging from mild diarrhea to fatal colitis. Therefore participants in this trial with diarrhea will be monitored especially for CDAD and in case of suspected or confirmed CDAD treatment will be immediately stopped and the patient will be treated for CDAD as appropriate (fluid and electrolyte management, protein supplementation, antibacterial treatment of C. difficile, surgical evaluation if clinically indicated) until recovery. Since CDAD has been reported to occur over two months after the administration of antibacterial agents, patients will be

strongly encouraged to report any severe diarrhea to the research team also after completion of the treatment.

Superinfection
As with other antibiotic preparations, use of this drug may result in overgrowth of non-susceptible organisms, including fungi. If superinfection occurs, treatment will be immediately stopped and appropriate therapy instituted until recovery.

Benign Intracranial Hypertension (pseudotumor cerebri)
Bulging fontanels in infants and benign intracranial hypertension in adults have been reported in individuals receiving tetracyclines. These conditions disappeared when the drug was discontinued. Therefore treatment of patients with a suspected benign intracranial hypertension (e.g. headache associated with gradual visual field defects, nausea, vomiting, drowsiness) will be stopped immediately and appropriate therapy will be given until full recovery.

9.5.3 General precautions and warnings

Pregnancy and breastfeeding:
To avoid any complications, pregnant or breastfeeding women will be excluded from the clinical trial. Pregnancy tests will be carried out during the screening and right before the first treatment. Additionally, pregnancy tests will be repeated after three and six weeks of treatment. In case of pregnancy in any group, treatment will be stopped immediately. All women will be informed in detail about the risks of getting pregnant during the informed consent procedure and their obligation and responsibility to use effective contraceptive methods excluding daily hormonal contraception as the trial drugs can reduce their efficacy.

9.6 Blistering and Labeling of the IMP

Blistering and labeling of the trial drugs will be done by Piramal Healthcare, Morpeth, United Kingdom. All IMPs will be blistered and labeled for the conduct of clinical trials only.

9.7 Transport of IMP

The trial drugs will be delivered to the trial sites by plane and car at appropriate conditions (temperature not above 25°C).

9.8 Handling of IMP at the Site and Drug Accountability

All study drug will be kept in a secure cabinet or a room with access restricted to the responsible study personnel. Medication must be stored at appropriate conditions (temperature not above 25°C). The storage and handling of all study medication will be supervised by the study pharmacist.
The study pharmacist will be responsible for maintaining accurate records of receipt of all study medication as well as of all medication dispensed to and used by each patient.
At the completion of the study, all unused medication will be destroyed once drug accountability is complete.

9.9 Strategies to improve adherence and compliance

Both doxycycline and placebo will be administered under supervision throughout the treatment period (directly observed treatment). Ideally, patients will be required to come every day to the meeting point in their village to take their drugs under supervision. In case a patient is not present for the DOT, the drugs will be handed out to a village health worker or a family member and the intake by the patient

will be checked at the following day when the patient will have to give back the empty package and the village health worker or family member will be asked to witness the intake. In some cases, patients have to travel for a few days. When they proved their compliance before, the drugs for the travel days will be handed out to the patient and compliance will be reviewed by getting back the empty packages when the patient comes back from travel. In this case the patient will be asked to immediately report any adverse reactions or problems with the trial drugs by mobile phone to the trial clinician or to contact a village health worker or the nearest health facility who will then call the research team.
All patients will be counseled at the time of the initial dose and during the treatment period. Key messages will include:

- The importance of following study guidelines for adherence
- Instructions about taking study pills whole, and what to do in the event of a missed dose.
- Reinforcement that study pills may be doxycycline or placebo
- Emphasize that all participants are expected to benefit from the hygiene intervention.
- Importance of contacting the research team if experiencing problems possibly related to study product
- Patients will be questioned about problems with the drugs and motivated to complete treatment as planned
- In addition, patients will be visited once every two months in the follow-up period to record the occurrence of ADLA attacks and reinforce the hygiene routine.

9.10 Unblinding

The patient, the investigators and all other site staff will be kept blinded throughout the study. All assessments of the patient will be made without knowledge of the treatment and every effort must be made to maintain the blinding. Unblinding will only occur in the case of medical emergencies or pregnancy and only if knowledge about the administered study drug is expected to improve the patient’s treatment.

9.11 Prior and Concomitant Therapy/Medication

9.11.1 Previous therapy / medication of trial specific illness

All previous treatments for managing LE (e.g. Mass drug administration, previous rounds of IVM and/or ALB, last intake of IVM, last intake of ALB) and applied medications will be documented in the CRF according to the memory of the patient.

9.11.2 Previous therapy / medication with doxycycline

All previous treatments and medications with doxycycline during the last year before starting the trial will be documented in the CRF according to the memory of the patient.

9.11.3 Concomitant therapy / medication for other indications

All concomitant therapies/medications other than the trial IMP applied during the trial at the discretion of the trial clinician will be documented in the CRF including the following information:

- brand name,
- indication,
- dose per intake and units,
- route of administration,
- time/duration of treatment (start and stop date of treatment or ongoing instead of the stop date if treatment is still persisting at subject’s termination visit)

9.11.4 Prohibited therapy / Concomitant medication

Diet: There is no dietary restriction and participants will be encouraged to eat within one hour prior to drug intake.
The following therapies / medications are not allowed to be applied during the treatment because of interaction with the trial dugs:

- antacids containing aluminium, magnesium or sucralfate
- other antibiotics than doxycycline
- sulfonylurea, coumarin (doxycycline increases the levels of the coumarin and sulfonylurea which might lead to an adverse event)

If it is important to start treatment with one of the above mentioned prohibited therapies, treatment with the trial drugs will be stopped to prevent the patient from the interactions.
Female patients will be informed in detail that oral contraceptives might not work due to interactions with doxycycline. They will only be included in the clinical trial if they agree to use other agreed methods of contraception.
During the whole study the use of diuretics is not recommended, as they will change the outcome of the measurement. Medication will lead to exclusion of patient data from the analysis.
During treatment and the 24 months follow-up period patients should not receive antifilarial drugs (except during MDA). Short courses of antibiotics are permitted for the treatment of ADLA attacks and infections such as UTI or URTI. Intake of all drugs other than the study drugs will be documented on the diary cards and every two months in the case report forms.

9.11.5 Rescue/Escape/Salvage Therapy

not applicable

10. ADVERSE EVENTS

10.1 Documenting, Recording and Reporting Adverse Events

At each contact with the subject, information regarding adverse events will be elicited by appropriate questioning and examinations and will be:

- immediately documented in the subject’s medical record/source document,
- recorded on the Adverse Event Case Report Form (AE CRF) and
- reported as outlined

10.2 Definitions

10.2.1 Adverse Event (AE)

An adverse event is any untoward or unfavorable medical occurrence in a human subject, including any abnormal sign (e.g., abnormal physical exam or laboratory finding), symptom, or disease, temporally associated with the subject’s participation in the research, whether or not considered related to the research. An unexpected AE is an experience not reported in the current Investigators Brochure or elsewhere.

10.2.2 Unexpected Adverse Event (UAE)

An AE is unexpected if it is not listed in the Investigator’s Brochure (IB) or Package Insert (PI) (for marketed products) or is not listed at the specificity or severity that has been observed. It is the responsibility of the IND Sponsor to make this determination.

10.2.3 Adverse (Drug) Reaction (AR)

An adverse reaction is an adverse event that is caused by an investigational agent (drug or biologic).

10.2.4 Suspected Adverse (Drug) Reaction (SAR)

An adverse event for which there is a reasonable possibility that the investigational agent caused the adverse event. ‘Reasonable possibility’ means that there is evidence to suggest a causal relationship between the drug and the adverse event. A suspected adverse reaction implies a lesser degree of certainty about causality than adverse reaction which implies a high degree of certainty.

10.2.5 Unexpected Adverse (Drug) Reaction (UAR)

An adverse reaction, the nature or severity of which is not consistent with the applicable product information (e.g., Investigator's Brochure for an unapproved investigational product or package insert/summary of product characteristics for an approved product)

10.2.6 Serious Adverse Event (SAE)

A Serious Adverse Event is an AE that results in one or more of the following outcomes:

- death
- a life threatening (i.e., an immediate threat to life) event
- an inpatient hospitalization or prolongation of an existing hospitalization
- a persistent or significant incapacity or substantial disruption of the ability to conduct normal life functions
- a congenital anomaly/birth defect
- a medically important event (Medical and scientific judgment should be exercised in deciding whether expedited reporting is appropriate in other situations, such as important medical events that may not be immediately life threatening or result in death or hospitalization but they may jeopardize the subject or may require intervention to prevent one of the other outcomes listed above.)

10.2.7 Serious Adverse (Drug) Reaction (SAR)

This is defined as an adverse drug reaction that is serious and at least possibly related to the IMP (see SAE criteria above).

10.2.8 Suspected Unexpected Serious Adverse (Drug) Reaction (SUSAR)

A SUSAR is a Suspected Adverse Reaction that is both Serious and Unexpected. Any UAR that at any dose results in death, is life-threatening, requires inpatient hospitalization or prolongation of existing hospitalization, results in persistent or significant disability/incapacity or is a congenital anomaly/birth defect.

10.2.9 Unanticipated Problem (UP)

An Unanticipated Problem is any event, incident, experience, or outcome that is:

1. unexpected in terms of nature, severity, or frequency in relation to
   a. the research risks that are described in the IRB-approved research protocol and informed consent document; Investigator’s Brochure or other study documents; and
   b. the characteristics of the subject population being studied; and
2. possibly, probably, or definitely related to participation in the research; and
3. places subjects or others at a greater risk of harm (including physical, psychological, economic, or social harm) than was previously known or recognized.

10.2.10 Unanticipated Problem that is not an Adverse Event (UPnonAE)

Unanticipated problem that is not an Adverse Event (UPnonAE): An unanticipated problem that does not fit the definition of an adverse event, but which may, in the opinion of the investigator, involve risk to the subject, affect others in the research study, or significantly impact the integrity of research data. Such events would be considered a non-serious UP. For example, we will report occurrences of breaches of confidentiality, accidental destruction of study records, or unaccounted-for study drug

10.3 Criteria to be evaluated by the trial clinician

If a diagnosis is clinically evident (or subsequently determined), the diagnosis rather than the individual signs and symptoms or lab abnormalities will be recorded as the AE.
All AEs occurring from the time the informed consent is signed through the 4 months follow-up will be documented, recorded, and reported. The trial clinician will evaluate all AEs with respect to **Seriousness** (criteria listed above in chapter 10.2), **Severity** (intensity or grade), and **Causality** (relationship to study agent and relationship to research) according to the following guidelines.

10.3.1 Assessment of Intensity

Any adverse event has to be graded regarding its intensity:	
MILD (Grade 1)	Does not interfere with subject's usual activities or is transient, easily tolerated.
MODERATE (Grade 2)	Interferes to some extent with subject's usual activities (which patient is still able to perform).
SEVERE (Grade 3)	Interferes significantly with subject's usual activities (which patient is not able to perform)

10.3.2 Assessment of Adverse Event Intensity for Doxycycline

**Adverse Event**	**Grade**	**Intensity**
Stomach Pain	0<br>1<br>2<br>3	Absent<br>Pain is easily tolerated (able to eat)<br>Pain interferes with daily activities (unable to eat)<br>Pain that prevents daily activities (combined with vomiting and/or diarrhoea)
Loss of appetite	0<br>1<br>2<br>3	Absent<br>Accompanied with nausea<br>Accompanied with nausea and vomiting or diarrhoea<br>Accompanied with nausea and loss of weight
Nausea	0<br>1<br>2<br>3	None<br>Nausea that is easily tolerated (able to eat)<br>Nausea that interferes with daily activity (unable to eat)<br>Nausea that prevents daily activity (combined with vomiting and/or diarrhoea)
Vomiting	0<br>1<br>2<br>3	None<br>Vomiting that is easily tolerated (able to eat)<br>Vomiting that interferes with daily activity (unable to eat)<br>Vomiting that prevents daily activity (combined with nausea and/or diarrhoea)
Diarrhea	0<br>1<br>2	None<br>Diarrhoea that is easily tolerated (up to 3 times per day)<br>Diarrhoea that interferes with daily activity (more than 3 times per day combined with nausea and/or vomiting and weakness)

	3	Diarrhoea that prevents daily activity (combined with nausea, vomiting and/or acholic faeces)
Bloody diarrhea	0<br>1<br>2<br>3	None<br>Fresh or clotted in absence of haemorrhoids<br>Fresh or clotted blood combined with abdominal pain<br>Fresh and clotted blood combined with abdominal pain and fever
Headache	0<br>1<br>2<br>3	None<br>Headache that is easily tolerated<br>Headache that interferes with daily activity<br>Headache that prevents daily activity
Urticaria	0<br>1<br>2<br><br>3	None<br>Requiring no medication<br>Requiring oral and/or topical medication (including steroid) for < 24h<br>Requiring oral, topical and/or medication IV medication (including steroid) for > 24h
Rashes	0<br>1<br>2<br>3	Absent<br>Localized, itching, no blisters, lasting one day<br>Localized itching, with blisters lasting longer than one day<br>Generalized combined with fever
Phototoxicity	0<br>1<br>2<br>3	Absent<br>Erythema in sun exposed skin<br>Erythema in sun exposed skin and rise of skin temperature<br>Erythema in sun exposed skin, blisters and fever
Fever	0<br>1<br>2<br>3	≤ 37.5°C<br>>37.5°C-38°C<br>>38°C-39°C<br>>39°C
Anaphylactic reactions	0<br>1<br>2<br>3	None<br>headache accompanied by rash and itching<br>in addition to 1. blood pressure failure and tachycardia, oedema<br>in addition to 2. bronchial spasms (expiratory stridor), apnoea or cardiac arrest →SAE

10.3.3 Assessment of Seriousness

Determination of the seriousness of the adverse event according to the definitions for a serious adverse event (SAE) given in section 10.2.6.

10.3.4 Assessment of Causality

Causality (likelihood that the event is related to the study agent) will be assessed considering the factors listed under the following categories:

Definitely Related

- reasonable temporal relationship
- follows a known response pattern
- clear evidence to suggest a causal relationship
- there is no alternative etiology

Probably Related

- reasonable temporal relationship
- follows a suspected response pattern (based on similar agents)
- no evidence of a more likely alternative etiology

Possibly Related

- reasonable temporal relationship
- little evidence for a more likely alternative etiology

Unlikely Related

- does not have a reasonable temporal relationship

OR

- good evidence for a more likely alternative etiology

Not Related

- does not have a temporal relationship

OR

- definitely due to an alternative etiology

10.4 Adverse Event Recording

Any AE relevant for the evaluation and analysis of the clinical trial has to be documented in the source data and in the CRF on the respective Adverse Event Report Form.
Documentation and evaluation of each AE occurring between:

- Visit 3 (first day of treatment) and
- Visit 5 (4 months follow-up)

10.5 Adverse Event Reporting

Line listings, frequency tables, and other summary AE data will be submitted to the Sponsor when needed for periodic safety assessments requested by the DSMC, review of annual reports, review of safety reports, and preparation of final study reports.

10.6 Serious Adverse Event Reporting

10.6.1 Serious Adverse Event Reporting

Any SAE (whether or not they are also UPs) ocurring after the subject has received the trial drug for the first time until the 2 months follow-up (Visit 4) will be documented and reported within 48 hours after investigator awareness of the event to the Ghana FDA, the GHS and to the Sponsor informally via email containing at least the minimal criteria (the name of the clinical trial, an identifiable patient, an identifiable reporter, a reaction/event, a causality assessment). The Sponsor will add missing information in consultation with the investigator and submit the completed SAE form to the FDA and the GHS.

FDA: drug.safety@fdaghana.gov.gh

GHS: ghserc@gmail.com

10.7 Reporting of Unanticipated Problems (UPs)

Unanticipated Problems (definition see chapter 10.2.9) that are also adverse events must be reported to the Sponsor and sent by fax or e-mail attachment no later than 7 calendar days of site awareness of the event. UPs that are not AEs are not reported to the Sponsor.

10.8 Follow-up of Adverse Events

Every AE or SAE will be treated according to clinical standards at the discretion of the investigator and followed up until it is resolved. Costs for treatment of AEs/SAEs will be covered by the research team.

10.9 Handling of emergency cases

The study clinicians/physicians will visit the participants in their villages every day during the treatment period to closely supervise possible AEs or SAEs. Additionally the phone numbers of the trial clinicians will be handed to the village health workers as well as to the participants. In case of an emergency between the visits, the clinicians have to be called and will come to the participants whenever needed. In case the participant has to go to a hospital for further examinations and treatment, the transport and medical care will be paid by the research team.

10.10 Deaths

All fatal cases during the treatment period and the time of follow-ups will be accompanied by a formal autopsy report. Deaths occurring in the period between treatment and follow-ups will be evaluated in detail and, if reported immediately to the research team, also accompanied by a formal autopsy.

10.11 Pregnancies

Female participants will be screened for pregnancy before enrolment. Pregnant and/or breastfeeding women will be excluded because doxycycline is contraindicated in pregnancy and during breastfeeding. However, non-pregnant women of childbearing-potential using an approved, effective method of contraception before, during and for at least 2 weeks after the completion of the active intervention with doxycycline or placebo are eligible for inclusion. As doxycycline is reducing the blood level of contraceptives it is recommended to use an additional method (barrier) to prevent a pregnancy during the treatment phase with doxycycline.

Approved methods of contraception such as implants, intrauterine device (IUD), condoms etc. which are practiced in Ghana would be used.

10.11.1 Pregnancy Procedures

Women of childbearing potential are required to have a negative urine pregnancy test to exclude a pregnancy before being enrolled in the clinical trial.

Pregnancy testing will be conducted prior to the first dose of the trial drug and will be repeated after 2, 4 and 6 weeks of treatment.

All women of childbearing potential will be instructed to contact the investigator immediately if they suspect they might be pregnant during the study (for example, missed or late menses). If pregnancy is suspected while the patient is receiving experimental study treatment, this will be withheld immediately until pregnancy can be ruled out with certainty.

If a woman becomes pregnant during the treatment period, treatment with the IMP will be immediately stopped. She will be treated according to clinical routine at the discretion of the treating medical doctor supported by the investigator. The woman will remain in the trial as a participant (Intention-to-treat collective).

10.11.2 Pregnancy Reporting

To ensure the safety of female subjects, each pregnancy that becomes known to the investigator during the trial, must be reported as an event similar to an SAE. Therefore the investigator will record and

report pregnancy information on the appropriate pregnancy report form as an initial report and send it immediately (latest within 24h) to the Sponsor.
The pregnancy itself is not considered to be an AE or SAE but must be followed up until delivery or until pregnancy termination and the outcome of pregnancy should be notified to the Sponsor to determine the outcome of the pregnancy regarding maternal or newborn complications. The investigator will seek and provide this follow-up information after the planned date of delivery. For this purpose the pregnancy report form will be used as follow-up report. Infants should be followed for a minimum of 12 months after delivery.
If the outcome of the pregnancy includes

- Spontaneous, therapeutic abortion or voluntary termination,
- stillbirth,
- neonatal death,
- presence of birth defects, or
- congenital anomaly (including that in an aborted fetus, stillbirth or neonatal death),

the investigator should report this outcome as an SAE.

11. STATISTICAL CONSIDERATIONS

11.1 Hypothesis

Doxycycline is superior to placebo for management of lymphedema in patients from LF-endemic areas.

11.2 Primary target variable

Lack of progression of LE (stage reduction or same stage as pre-treatment) examined 24 months after treatment onset.

11.3 Sample size estimations

11.3.1 Sample size estimations for group A (LE stage 1-3)

- Doxycycline 200mg/d will first be tested for superiority to placebo. When superiority of doxycycline 200mg/d is confirmed, doxycycline 100mg/d will subsequently be tested for superiority to placebo. If both treatment groups rejected their H_0-hypothesis, they will be tested for non-inferiority. With this subsequent design, a two-sided $\alpha = 5\%$ can be maintained for all 3 analyses.
- The estimates for progression are based on the results from the previous study by Mand et al. [2] where a progression of 4.9% was seen in the DOX 200 group after 24 months whereas the placebo-treated patients had a progression of 55.6%. The strict control implementation of hygiene measures in this trial may result in a stronger impact of this intervention on both arms. This would result in a smaller margin to verify the added benefit of doxycycline and as a consequence increase the number of patients in the study. In order to account for this influence in the sample size calculations the progression in the placebo group was assumed to be 25% instead of 55%.
- A dropout rate of 30% was assumed.

11.3.2 Sample size estimations for group B (LE stage 4-6)

- Doxycycline 200mg/d will first be tested for superiority to placebo with α=5% two-sided.
- Since the previous trial [2] was mainly carried out in patients with LE stages 1-3, the results cannot be used as basis for a sample size calculation for the LE-stages 4-6. Therefore a sample size was chosen for this part which is in line with the sample sizes normally taken for pilot trials.
- A dropout rate of 30% was assumed.

11.4 Sample size calculation

11.4.1 Sample size calculation for group A (LE stage 1-3)

Based on the assumption of a progression in the DOX 200 group of 5% and in the placebo group of 25%, there will be power of 95% to show superiority to placebo if 84 participants are included in each treatment group (+ 30% drop-out rate: N = 120/treatment). Using the subsequent design, superiority of DOX 100 to placebo can be shown with a power > 81% if the progression in this group is ≤ 8%.

11.4.2 Sample size calculation for group B (LE stage 4-6)

Since this part of the trial is a non-confirmatory pilot study to gain experience with doxycycline in the treatment of higher LE stages, the sample size cannot be justified by a statistical argumentation. Therefore a sample size of 30 participants (N = 21 + 30% drop-out rate) per treatment arm was chosen which is in line with the sample sizes normally taken for pilot trials.

11.5 Achievement of sample size

The clinical centers involved in the study were selected based on documentation for patient availability and availability of infrastructure and resources for the study (see 7.4.1 and 7.4.2).

11.6 Populations to be analyzed

Different analysis populations will be prepared before de-blinding (Safety population (SAF), Intention-to-treat population (ITT), Per-Protocol population (PP)). The criteria for the exclusion of patients from any of the analysis populations will be defined in the Statistical Analysis Plan (SAP) and will be finalized prior to database lock. The exclusion of participants from the analysis populations will then be decided during the blind data review before unblinding and will be described in detail in the blind data review report.
Since this is a superiority trial the ITT population will be used for all endpoint analyses. The PP population will be used to confirm the results of the ITT analysis. The SAF population will be used for the analysis of adverse events.

11.6.1 Intent-to treat (ITT) population

The ITT population will consist of all randomized patients in the groups to which they were randomly assigned and who took the study drugs at least once.

11.6.2 Per-Protocol (PP) population:

The PP population will consist of all patients who fulfil the protocol in terms of eligibility, compliance, interventions, and outcome assessment without any major protocol deviation.

11.6.3 Safety Population

The safety population will be defined as all patients who received any study drug

11.7 Statistical methodology

A detailed statistical analysis plan (SAP) will be written before final closure of the data file and before de-blinding of the research team. The Data Safety Monitoring Committee (DSMC) will have to agree on the data analysis plan before closure.

11.7.1 Analysis of baseline characteristics

Baseline characteristics, such as demographic and analytical data will be summarized for each intervention group using descriptive statistical methods.

11.7.2 Analysis of the primary endpoint

The frequencies of the participants with "progression" and their confidence intervals (95%) will be calculated and compared between the treatment groups using Fisher's exact test.

11.7.3 Analysis of the secondary endpoints

Quantitative data will be summarized for each intervention group using the mean, the median, standard deviation, the range (minimum and maximum value), 25th - 75th percentiles and 95% confidence intervals. Qualitative data will be summarized for each intervention group using frequency counts, percentages and 95% confidence intervals. Additionally, box plots or other graphical methods may be used to present the data. The groups will be compared using appropriate statistical methods

11.7.4 Safety Analysis

Adverse events (AE) will be assessed in the scope of the daily observed treatment (DOT) and described for each intervention group using frequency counts, percentages and 95% confidence intervals.

11.8 Interim Analysis

An interim analysis is planned at each study site when all patients have completed 12 months of follow-up. A decision at 12 months may be taken to terminate the study because of superiority of the doxycycline arm so that the placebo subjects can be treated. Superiority may be defined as non-progression of grade, reduced ADLA, etc. Details of the parameters to be used for the interim analysis will be defined prior to the closing of the database and incorporated in the SAP.

11.9 Protocol violations

Protocol violations are major deviations from the procedures outlined in this document like:

- non-compliance with investigational medicinal product (missing treatment days at the end of the treatment period (the treatment period should be prolonged for all days the patient was absent or did not get the trial drugs by decision of the trial clinician),
- intake of medications not allowed
- any non-adherence to the protocol that would have an impact to the subject's rights, safety or welfare,
- absence from treatment for more than 3 consecutive treatment days (participants who are absent for more than 3 consecutive treatment days will be requested to finish the whole treatment).
- absence from treatment for more than 7 treatment days (treatment will be stopped if participants are absent for more than 7 treatment days, but the patients will be asked to come for the follow-ups).

After a subject has been enrolled, it is the investigator's responsibility to make a reasonable effort to prevent and correct any protocol violations if necessary and to continue the subject's participation in the trial, if possible. Protocol violations will be reported to the sponsor/sponsor delegated person during the course of the trial in the monitoring reports and reported to the Ethics Committees after completion of the trial. All protocol violations will be listed and the impact on the evaluation of the subjects concerned will be discussed prior to statistical analysis.

11.10 Handling of Drop-outs, Withdrawal, and Missing Data

11.10.1 Screening failures

Subjects dropping out of the trial prior to randomization will be listed as screening failures including the reason.

11.10.2 Drop-out/Withdrawal after randomization but before treatment start

Subjects dropping out of the trial after randomization but before start of treatment will be reported including the reason.

11.10.3 Drop-out/Withdrawal during or after treatment

Subjects dropping out of the trial after randomization during but also after treatment will be analysed using all available data (ITT analysis)..

11.10.4 Missing treatment days

Subjects missing more than 3 consecutive treatment days will be analysed using all available data (ITT analysis) but will be excluded from the PP-analysis.

11.10.5 Replacement of Patients

- In case of subjects dropping out in the period between randomization and treatment start additional subjects will be included in the trial. The new participants will be randomized consecutively.
- In case of subjects dropping out during the first 3 days of treatment, additional subjects may be included in the trial. The new participants will be randomized consecutively. All available data from the participants who were treated at least once and later replaced will still be used for the ITT analysis.

11.11 Statistical report

The statistical evaluation and the statistical report will be performed, evaluated and signed by the responsible biometrician. All data in this report will be strictly confidential.

12.DATA SAFETY AND MONITORING COMMITTEE (DMSC)

A single central DSMC will be established with a charter that defines the roles and responsibilities and details of meeting frequency and communications. The DSMC thus established will, in addition to the Authorities/IRBs involved, have a chance to comment on the content of the protocol. The composition, role of the members and the reporting structure are attached as an appendix to this protocol.
Members of the DMSC are:
David Addis
David Diemert
Martin Peter Grobusch
Sabine Klaeger
Mike Osei-Atweneboana

13. DEFINITION OF END OF TRIAL

13.1 Regular end of trial

The regular end of trial is defined as Last Subject Last Visit, meaning, the trial ends when the last patient will be informed about the best performing treatment. At this visit re-treatment will be offered to patient. This re-treatment is not part of the clinical trial but will be performed by the trial clinicians.
After regular end or early termination of the trial, patients will be treated according to clinical routine at the discretion of the treating physician.

13.2 Termination of the trial for individual subjects

13.2.1 Termination by the participant

A patient may decide to withdraw from the study at any time and for any reason. The Investigator should attempt to determine the reason for the subject's decision. There will be no disadvantage for the participant as a result of a withdrawal. If a participant does not return for a scheduled visit, every effort should be made to contact the participant. The investigator should inquire about the reason for withdrawal and the participant should be followed-up regarding any unresolved adverse events, if possible. In any circumstance, every effort should be made to document the participants' outcome. Therefore all participants, even if the participant was withdrawn from the trial treatment, will be encouraged to come for the follow-up visits.

13.2.2 Termination by the investigator

The investigator may withdraw a patient for any of the following reasons:

- An individual subject's decision.
- Any clinical AE, laboratory abnormality or other medical condition or situation such that continued participation in the study would not be in the best interest of the subject. Subjects will be followed for the duration of the study for indicated safety assessments.
- Non-compliance with study procedures to the extent that it is potentially harmful to the subject or to the integrity of the study data.
- A change in the subject's baseline condition after enrollment so that the subject no longer meets the inclusion/exclusion criteria.

In any circumstance, every effort should be made to document the participants' outcome. Therefore all participants, even if the participant was withdrawn from the trial treatment, will be encouraged to come for the follow-up visits.

13.3 Early termination of the entire trial

The IRBs, the GHS-ERC, the Sponsor, the DSMC or the FDA may halt the study at any time following review of any safety concerns independent of the interim analysis and applicable to all centers. Halting the study requires immediate discontinuation of the study agent administered for all subjects and suspension of enrollment until a decision is made whether or not to continue study agent administration.

The halting criteria (as determined by site investigators) for an individual site include:

- two or more subjects experience the same or similar SAEs that are unexpected and are possibly, probably, or definitely related to the study agent

  OR

- any safety issue that the site investigators determine should halt the study

The halting criteria (as determined by the study DSMC secondary to aggregate data review) for this study include:

- two or more of the same or similar AE in different subjects that are grade 3 or above and are unexpected and possibly, probably, or definitely related to the study agent

  OR

- any safety issue that the study DSMC determines should halt the study

13.4 Report of termination of the trial

When the trial is prematurely terminated, the sponsor should submit a report to the IRBs/ FDA within 30 (thirty) days. This report will include:

- Justification for the premature ending or of the temporary halt of the trial;
- Number of patients receiving treatment at the time of the study termination;
- Proposed management of patients receiving treatment at the time of halt or study termination;
- Implications of the discontinuation on the evaluation of the final results.

13.5 Notification of the end of the trial

The end of the clinical trial is the date of the last visit of the last participant undergoing the trial. At the end of the trial, the sponsor delegated person will notify the IRBs/ FDA about the trial completion and submit a preliminary report on the ethical evaluation within 30 days and a final report within 90 days after completion of the trial.

14. DATA COLLECTION, HANDLING AND RECORD KEEPING

14.1 Data Collection methods

The investigator has ultimate responsibility for the accuracy, authenticity, timely collection and reporting of all clinical, safety, laboratory data entered on the Case Report Forms (CRFs). Data will be captured using paper CRF specially designed for the study and approved by the IRBs and FDA. The CRFs must be signed by the investigator or by an authorized staff member to attest that the data contained on the CRFs is true. Any corrections to entries made in the CRFs, source documents must

be dated, signed and explained (if necessary) and should not obscure the original entry. All paper CRFs will be secured in fireproof locking cabinet.
All clinical and laboratory procedures will be performed according to standard protocols governed by GCP and GLP guidelines, 21 CFR Part 11. Tools for qualitative assessments will be made available and validated prior to use.
All study personnel participating will undergo training in the procedures to be used in the study.
A work instruction's manual / manual of SOPs will be prepared at each site and will be made available to all study personnel and study monitors.

14.2 Electronic data capture (EDC)

Research data collected on paper CRFs will be transcribed and entered onto the REDCap (Research Electronic Data Capture: http://project-redcap.org/) system using double data entry. The EDC application is a secure encrypted web application for building and managing online surveys and databases specifically geared to support data capture for research studies. REDCap meets regulatory requirements for GCP/GCLP, 21 CFR Part 21 and HIPAA compliant with full audit trails capability for tracking data manipulation and user activity, as well as automated export procedures for seamless data downloads to Excel, PDF, and common statistical packages (SPSS, SAS, Stata, R). The equipment for data transfer may include laptops, or mobile applications for tablet computers and/or smartphones with either Android or iOS operating systems. All electronic tools will be password protected.

14.3 Data management

Study data will be managed using REDCap electronic data capture tools hosted at the University Hospital Bonn. REDCap (Research Electronic Data Capture) is a secure, web-based application designed to support data capture for research studies, providing: 1) an intuitive interface for data entry; 2) audit trails for tracking data manipulation and export procedures; 3) automated export procedures for seamless data downloads to common statistical packages; and 4) procedures for importing data from external sources [16].
Details on data management (procedures, responsibilities, data corrections, if any, which may be made by Data Management staff themselves, etc.) will be described in a data management plan prior to the trial. During the trial, the performance of data management and any deviations from the data management plan will be documented in a data management report. Before any data entry is performed, the trial database will be validated and the technical specifications of the database will be documented in a variable plan.
Processing of data is done via Double-Data-Entry. The two entries will then be compared with each other and verified. An audit trail will be created to provide an electronic record of which data were entered or subsequently changed, by whom and when.
SAS software will be used to review the data for completeness, consistency and plausibility. The checks to be programmed will be specified beforehand in a data validation plan, as required by the subject protocol. After running the check programs, the resulting queries will be sent to the investigator for review of his/her data. Answered queries will also be entered twice, verified and the updated data will then be transferred to the database. All programs which can be used to influence the data or the data quality will be validated (e.g. check programs, programs used for the input of external data, etc.).

All data will be checked for consistency and plausibility by the monitor and by the data management. Inconsistencies will be queried and discussed with the investigator. After data clearance the data base will be locked and data will be used for statistical analysis.

14.4 Trial site file

The trial site will be provided with a trial site file (ISF) containing all sponsor-specific essential and trial specific documents. The monitor will regularly check the trial site file for accuracy and completeness. The trial site file has to be stored locked and sure. After end of trial or early termination of the trial, the trial site file should be retained for 10 years at the site.
The ISF includes the subject identification list, where the investigator has to record the trial participation of each subject. This list allows identification of each subject and contains the subject number, the name, telephone number (if applicable) and the date of inclusion of the subject into the trial, and will be reviewed by the monitor for completeness. After end of the trial the subject identification list remains with the trial site.
The investigator should maintain a list of appropriately qualified persons to whom he/she has delegated trial duties. This list will be provided with the ISF, too.

15. MONITORING AND QUALITY ASSURANCE

During the clinical trial, quality control and quality assurance will be endured through monitoring, auditing and inspections by the national authorities.

15.1 Study Monitoring

To ensure accurate, complete, consistent, and reliable data, and ensure the patients´ safety, the investigator’s site and trial procedures will be monitored by a representative of the sponsor. The sponsor’s representative will visit the site:

- to evaluate the progress and recruitment of the trial,
- to review the source documents and CRFs for protocol compliance, accuracy and validation,
- to assess facilities and equipment,
- to check for protocol compliance,
- to assure the AE/SAE reporting,
- to verify proper handling and dispensing of the IMP(s), and other factors.

Frequency and scope of the monitoring visits will be defined in the Monitoring Plan for this trial which also includes the extent of source data verification that is required as a risk-adapted strategy rather than a full monitoring will be conducted.
The investigator agrees to cooperate with the monitor to ensure that any problems detected in the course of these monitoring visits are addressed and resolved, and therefore ensures the accuracy and consistency of the trial with GCP and all applicable laws. The investigator allows the monitor to have access to all trial related original data and documents relevant for the monitoring of the trial.

15.2 Audits and inspections

In accordance with GCP this trial may be selected for audit by representatives of the sponsor or for inspection by site responsible representatives of the competent national authorities.

The investigator agrees to give the auditor access to all relevant documents for review and to support the sponsor to solve possible audit findings concerning the trial conduct at the site.
After every audit the auditee(s) will rerceive an audit confirmation by the auditor. This document has to be filed together with the trial documentation and has to be made available also to the national authorities in case of an inspection.
At the end of the trial, a copy of the audit certificate(s) will be included in the final report.

15.3 Archiving

15.3.1 Archiving by the sponsor

All clinical and experimental data (electronic or paper) and all essential documents inclusively the CRFs shall be kept in a secured place (metallic safety cabinets under lock) for a period of 15 years after completion of the trial and be made readily available for review upon request by the national authorities and the IRBs. The sponsor must archive all trial related documents according to regulatory requirements.

15.3.2 Archiving by the investigator

The investigator will maintain all subject documents as specified in the Essential Documents for conduct of a clinical trial (see ICH-GCP, section 8) and as required by the applicable regulatory requirements after completion of the clinical trial so that they will be available for audits and inspections by the authorities. The investigator will be responsible for the storage.
The following retention periods will apply after completion or stop of the clinical trial:

- all essential documents and trial related data must be retained securely for at least 15 years,
- the subject identification list for at least 15 years,
- medical records and other source documents for the longest possible period allowed by the institution.

The investigator should take arrangements to prevent accidental or premature destruction and illegitimate access to these documents.
To enable evaluations and/or audits from the sponsor or inspections from regulatory authorities, the investigator agrees to keep records, including the identity of all participating subjects (sufficient information to link records, e. g. CRFs and other records), all original signed informed consent forms, copies of all CRFs, serious adverse event reports, source documents, and detailed records of treatment disposition, drug accountability and adequate documentation of relevant correspondence (e. g. letters, meeting minutes, telephone calls reports).

16.ETHICAL CONSIDERATIONS

16.1 Basic principles

The trial will be carried out conforming to the principles of the Declaration of Helsinki 1964 (amended most recently in 2013) and according to Good Clinical Practice (GCP) guidelines and according to 21 CFR Part 11 guidelines. Each trial site will submit a site-specific protocol to its IRBs and National authorities prior to the commencement of the trial at that site. The principal investigator at each site is responsible for the preparation of the protocol based on the core protocol. The composition of the IRBs and procedures for approval of the protocol will be in accordance with the guidelines prescribed by the National authorities where the study will be conducted.
This trial will be registered at the following trial registries:
ISRCTN

PACTR

16.2 Involvement of Ethics Committees and Regulatory Authorities

The protocol, the informed consent documents (ICFs) to be used in this study will be submitted to:

- The Committee for Human Research, Publication and Ethics (CHRPE) of the Kwame Nkrumah University of Science and Technology (KNUST), Kumasi, Ghana
- The Ghana Health Service Ethics Review Committee (GHS-ERC), Accra, Ghana
- The Ghana Food and Drug Authority (Ghana FDA), Accra, Ghana
- The Ethikkommission an der Medizinischen Fakultät der Rheinischen Friedrich-Wilhelms-Universität Bonn, Bonn, Germany

Written documentation of approval of both the protocol and the informed consent will be provided by the sponsor before starting the study.

16.3 Responsibilities of the Investigator

By signing this protocol the investigator declares his/her commitment:

- to not enrol any person dependent on him/her or the sponsor in accordance with the principles of GCP
- to inform the subjects of the transmission of their pseudonymized data and to make sure that subjects unwilling to give consent to the processing of their data are not included into the trial
- to certify that he/she was informed of the pharmacological – toxicological issues and risks of the clinical trial
- to be qualified by education, training and experience to assume responsibility for the proper conduct of the subject
- to be thoroughly familiar with the appropriate use of the trial drug(s), as described in the protocol, the product information and other information sources provided by the sponsor
- to be aware of, and comply with GCP and the applicable regulatory requirements
- to maintain a list of appropriately qualified persons to whom the investigator has delegated significant subject related duties.

The investigator should conduct the clinical trial in compliance with this protocol. For this purpose, the document will be signed by the sponsor and the investigator. As a general rule, the investigator should not deviate from the protocol or make amendments to the protocol without the agreement of the sponsor/authorities/ethics committees (unless subject safety is at risk, see below).

Any deviations from the approved protocol should be documented and explained by the investigator or an individual who is designated by the investigator.

The investigator may deviate from the protocol or make an amendment to the protocol without prior approval of the ethics committee to eliminate immediate risks to the subjects. An amendment if necessary should subsequently be reported to the ethics committees, the sponsor or sponsor delegated person and the competent authorities, giving reasons.

16.4 Protocol amendments

Any substantial amendments to the protocol or subsequent changes to the informed consent form as a result of changes to the protocol must be sent to the IRBs/ FDA. Records of the IRB review and opinion of all documents pertaining to this trial must be kept on file by the investigator and are subject to regulatory authority and/or sponsor inspection during or after completion of the trial.

The sponsor delegated person will submit quarterly progress reports to the FDA providing information

about the recruitment and safety of the clinical trial.
Protocol amendments detailing minor administrative changes should be submitted by the investigator to the IRBs and regulatory authorities for notification purposes as appropriate.

16.5 Benefits and risks of the study

By participating in the study the participant will benefit from thorough medical evaluations as well as receiving training and supplies for local care of lymphedema that by itself should improve his or her health. The two previous studies [1, 2] have already shown a benefit for lymphedema patients by taking doxycycline. Should the present study also be successful, those of the participant that received doxycycline will have had the added benefit of improvement in their disease state; those in the placebo arm of the study will be offered a six-week course of doxycycline at the end of the study. All participants will be informed about the outcome of the study after the 24 months period of supervision.

16.6 Subject information

Written or witnessed thumb print consent will be obtained from every patient or the legal representative (in case of a child between 14 – 17 years) prior to any procedures being done specifically for the study.
Informed consent is a process where information is presented to enable persons to voluntarily decide whether or not to participate as a research subject. It is an on-going conversation between the human research subject and the researchers that begins before consent is given and continues until the end of the subject's involvement in the research. The process of obtaining informed consent will be governed by the guidelines of the respective countries where the trial will take place.
Broadly, discussions about the research will provide essential information about the study and include: purpose, duration, experimental procedures, alternatives, risks and benefits. Subjects will be given the opportunity to ask questions and have them answered. The subjects will sign the informed consent document prior to undergoing any research procedures. The subjects may withdraw consent at any time throughout the course of the trial. A copy of the informed consent document will be given to the subjects for their records. The researcher will document the signing of the consent form in the case report form (CRF) and the original signed consent retained in a separate file along with other protocol specific documents required for compliance with GCP. The rights and welfare of the subjects will be protected by emphasizing to them that the quality of their medical care will not be adversely affected if they decline to participate in this study.
For illiterate patients, study information is given in the presence of an impartial, literate witness, who will read the information sheet to the patient or will witness the complete reading of the information sheet to the patient. The patient will give consent by thumb printing the ICF and the witness states that free, informed consent has been given by his/her signature on the ICF.
Patients with a suspicion of lymphedema will be invited to be screened for inclusion in the study. They will be given a patient informed consent form (ICF) about the study and will be explained the anticipated benefits and the potential risks associated with the protocol procedures. The principal investigator or a person designated by the PI will fully inform the patient, or the patient's legally acceptable representative. The language used will be as non-technical as possible and the patient will not unduly be influenced to participate in the study.
There will be a second informed consent form for "Sample storage, re-utilization and shipment". With this consent form the participant will be asked to give his/her consent for the storage and re-use of the samples taken during the clinical trial also after finalization of the trial to find new ways to better diagnose or treat lymphatic filariasis and the development of lymphedema. Since the development of

new methods takes also place at the IMMIP Bonn, Germany, the LMU, Munich Germany or the partner institutes in Tanzania and Cameroon, the participants will also be asked for their consent to have the samples shipped to Germany, Tanzania or Cameroon. Patients will be told that there will be no negative effect on their participation in the trial if they decide not to sign this second ICF.

16.7 Obtaining informed consent

At each site, the principal investigator with the approval of the IRBs/ National authorities will finalize the consent seeking process including the allocation of responsibility of obtaining consent from trial participants.

16.8 Confidentiality

All records will be kept confidential to the extent provided by national federal, state and local laws. The study monitors and other authorized representatives of the Sponsor may inspect all documents and records required to be maintained by the Investigator, including but not limited to, medical records. Specific study records will be kept in locked cabinets and all computer data, data entry programmes and networking programs will be password protected. Personal information such as patient names, hospital numbers and addresses will not be recorded on the CRFs used. If records are required for examination, this information will be blanked out; however, a secure record of the linkages between individuals and their records will be maintained. Clinical information will not be released without written permission of the subject, except as necessary for monitoring by IRB, the national regulatory authority, or the sponsor's designee. All such information will be anonymized.

16.9 Declaration of interests

Principal investigators for the overall trial and each study site will declare their financial and other competing interests prior to the commencement of the trial and these declarations will be available on file for inspection.

17. ADMINISTRATIVE CONSIDERATIONS

17.1 Patient Insurance

Every subject participating in the trial will be insured against any trial-related illness/injuries pursuant to the legal requirements which may occur during the trial. Excluded from this, however, are injuries to health and deterioration of illnesses already in existence which would have continued to exist even if the subject had not taken part in the clinical trial. The investigator will inform the subject of the existence of the insurance, including the obligations arising from it. The participants must be afford access to insurance documents and provided with a copy of the general conditions of insurance on request. The insurance cover is jeopardized if the subject fails to immediately report (within 7 days) to the investigator or responsible physician any injury to health which might have resulted from the participation in the clinical trial, or if she/he undergoes any other medical treatment (except for emergency treatment) without the investigator's knowledge before her/his participation in the clinical trial has officially ended. In case of any health impairment the subject is obliged to notify the investigator as soon as possible. The investigator is then obliged to notify the insurance and additionally to make a report to the sponsor. The subject insurance will be arranged by the sponsor delegated person.

The insurance company in Ghana will be:

Enterprise Insurance
Kumasi Office
Pentecost House
Asokwa P.O. Box 1000
Adum, Kumasi
Ghana
Email: info.insurance@enterprisegroup.com.gh
Website: www. enterprisegroup.net.gh

This insurance covers trial related injuries to health up to a maximum of 300.000 Ghana Cedis per subject.

17.2 Incentives and expenses

The participants will receive food items such as Milo, Milk, rice etc. worth 50 Ghs. The participants will be visited in their communities early in the morning before they leave for their farms or late in the afternoon after they had come back so there will be a minimal loss of time to them.
Additionally, each participant will receive a lymphedema hygiene kit consisting essentially of a plastic bowl, 5 pieces of soap, 2 cotton towels.
Technical and material support will be provided to community health centers that will house the study.
The sponsor will cover patient care as it is related to the study, for the life of the protocol, and after the protocol for a period of 6 months.

17.3 Trial reports

Quarterly reports of the progress of a clinical trial starting from the date of issuance of the clinical trial certificate will be submitted to the national authority in the recommended format within 21 days after the end of the previous quarter (a quarter will be considered as three months beginning from the date of initiation of the clinical trial).
If the trial does not begin or is delayed as per the date of commencement on the Clinical Trial Certificate issued, the national authority will be informed of the new date of commencement within ninety (90) days of issuance of the Clinical Trial Certificate.

17.3.1 Final Report

In addition to the reports referred to above, the Principal Investigator/Sponsor delegated person who conducted the trial will, not later than 90 days after the completion of the trial, compile and submit a comprehensive formal report to the national authority conforming to the ICH Guideline.
The report (hard and soft copies (1 each)) will include a short but comprehensive summary of the essential findings of the trial and of its methodology and course.

17.4 Publication Policy

The results of the multi-national study should be published in a joint paper with authorship from all participating study sites. In addition, each site will be able to publish its site-specific results independently following the submission of the joint paper or 6 months after closure of the last collaborating study, whichever is earlier.
Authorship will be restricted to those persons that had a significant input into the design, implementation and analysis of the study. Professional writers will not be used.

18.REFERENCES

1. Debrah AY, Mand S, Specht S, et al. Doxycycline reduces plasma VEGF-C/sVEGFR-3 and improves pathology in lymphatic filariasis. PLoS Pathog **2006**; 2(9): e92.
2. Mand S, Debrah AY, Klarmann U, et al. Doxycycline improves filarial lymphedema independent of active filarial infection: a randomized controlled trial. Clin Infect Dis **2012**; 55(5): 621-30.
3. Dreyer G, Addiss D, Dreyer P, Noroes J. Basic Lymphoedema Management. Treatment and prevention of Problems Associated with Lymphatic Filariasis. Hollis, NH, USA: Hollis Publishing Company, **2002**.
4. Gyapong JO, Kumaraswami V, Biswas G, Ottesen EA. Treatment strategies underpinning the global programme to eliminate lymphatic filariasis. Expert Opin Pharmacother **2005**; 6(2): 179-200.
5. Global programme to eliminate lymphatic filariasis: progress report, 2013. Wkly Epidemiol Rec **2014**; 89(38): 409-18.
6. Ramaiah KD, Ottesen EA. Progress and impact of 13 years of the global programme to eliminate lymphatic filariasis on reducing the burden of filarial disease. PLoS Negl Trop Dis **2014**; 8(11): e3319.
7. Taylor MJ, Makunde WH, McGarry HF, Turner JD, Mand S, Hoerauf A. Macrofilaricidal activity after doxycycline treatment of Wuchereria bancrofti: a double-blind, randomised placebo-controlled trial. Lancet **2005**; 365(9477): 2116-21.
8. Turner JD, Mand S, Debrah AY, et al. A randomized, double-blind clinical trial of a 3-week course of doxycycline plus albendazole and ivermectin for the treatment of Wuchereria bancrofti infection. Clin Infect Dis **2006**; 42(8): 1081-9.
9. Coulibaly YI, Dembele B, Diallo AA, et al. A randomized trial of doxycycline for Mansonella perstans infection. N Engl J Med **2009**; 361(15): 1448-58.
10. Shenoy RK, Kumaraswami V, Suma TK, Rajan K, Radhakuttyamma G. A double-blind, placebo-controlled study of the efficacy of oral penicillin, diethylcarbamazine or local treatment of the affected limb in preventing acute adenolymphangitis in lymphoedema caused by brugian filariasis. Ann Trop Med Parasitol **1999**; 93(4): 367-77.
11. Ustun TB, Chatterji S, Kostanjsek N, et al. Developing the World Health Organization Disability Assessment Schedule 2.0. Bull World Health Organ **2010**; 88(11): 815-23.
12. Hoerauf A, Volkmann L, Hamelmann C, et al. Endosymbiotic bacteria in worms as targets for a novel chemotherapy in filariasis. Lancet **2000**; 355(9211): 1242-3.
13. Hoerauf A, Mand S, Adjei O, Fleischer B, Buttner DW. Depletion of wolbachia endobacteria in Onchocerca volvulus by doxycycline and microfilaridermia after ivermectin treatment. Lancet **2001**; 357(9266): 1415-6.
14. Debrah AY, Specht S, Klarmann-Schulz U, et al. Doxycycline Leads to Sterility and Enhanced Killing of Female Onchocerca volvulus Worms in an Area With Persistent Microfilaridermia After Repeated Ivermectin Treatment: A Randomized, Placebo-Controlled, Double-Blind Trial. Clin Infect Dis **2015**; 61(4): 517-26.
15. Hoerauf A. Filariasis: new drugs and new opportunities for lymphatic filariasis and onchocerciasis. Curr Opin Infect Dis **2008**; 21(6): 673-81.
16. Harris PA, Taylor R, Thielke R, Payne J, Gonzalez N, Conde JG. Research electronic data capture (REDCap)--a metadata-driven methodology and workflow process for providing translational research informatics support. J Biomed Inform **2009**; 42(2): 377-81.

19. ABBREVIATIONS

AE	*Adverse Event*
ADLA	*Acute dermatolymphangioadenitis*
ALB	*Albendazole*
AR	*Adverse Reaction*
BMBF	*Federal Ministry of Education and Research, Germany*
CDAD	*Clostridium difficile-associated diarrhea*
CHP	*Community health partnership*
CHRPE	*Committee for Human Research, Publication and Ethics*
CNS	*Central nervous system*
CRF	*Case Report Form*
DEC	*Diethylcarbamazine*
DOT	*Daily observed treatment*
DOX	*Doxycycline*
DSMC	*Data and Safety Monitoring Committee*
ERC	*Ethics Review Committee*
FDA	*Food and Drug Authority*
FTS	*Filarial test strip*
GCP	*Good Clinical Practice*
GH	*Ghana*
GHS	*Ghana Health Service*
GLP	*Good Laboratory Practice*
GPELF	*Global Programme to Eliminate Lymphatic Filariasis*
IB	*Investigator's Brochure*
ICF	*Informed Consent Form*
ICH	*International Conference on Harmonisation*
ICT	*Immunochromatographic test strip*
IMP	*Investigational Medicinal Product*
IMMIP	*Institute for Medical Microbiology, Immunology and Parasitology*
IRB	*Institutional Review Board*
ISF	*Trial site file*
ITT	*Intention to treat*
IVM	*Ivermectin*
KCCR	*Kumasi Centre for Collaborative Research*
KNUST	*Kwame Nkrumah University of Science and Technology*
LE	*Lymphedema*
LF	*Lymphatíc Filariasis*
LMU	*Ludwigs-Maximilian Universitöt*
MedDRA®	*Medical Dictionary for Regulatory Activities*
Mf	*Microfilaria*
MDA	*Mass drug administration*
N	*Number (typically refers to subjects)*
NIMR	*National Institute of Medical Research*
NTD	*Neglected tropical disease*
PI	*Principal Investigator*
PP	*Per protocol*

QoL	*Quality of life*
RCT	*Randomized controlled trial*
REDCap	*Research Electronic Data Capture*
SAE	*Serious Adverse Event*
SAR	*Serious Adverse Reaction*
SDP	*Sponsor delegated person*
SOP	*Standard Operating Procedure*
SSA	*Sentinel site assessment*
SUSAR	*Suspected Unexpected Serious Adverse Reaction*
UAE	*Unexpected Adverse Event*
UAR	*Unexpected Adverse Reaction*
UP	*Unanticipated Problem*
UPnonAE	*Unanticipated problem that is not an Adverse Event*
WHA	*World Health Assembly*
WHO	*World Health Organisation*
