# Supplementary material for: The design and development of a multicentric protocol to investigate the impact of adjunctive doxycycline on the management of peripheral lymphoedema caused by lymphatic filariasis and podoconiosis
Source: Parasit Vectors. 2020 Mar 30;13:155. doi: 10.1186/s13071-020-04024-2 (PMC7106687; doi:10.1186/s13071-020-04024-2)
Supplement: Supplementary file 3 — Additional file 3: Text S3. TAKeOFF podoconiosis protocol. [file 13071_2020_4024_MOESM3_ESM.pdf]

Additional file 3: Text S3. TAKeOFF podoconiosis protocol.

University of Buea

P.O.Box 63, Buea,

Cameroon

SPONSORED BY THE

Federal Ministry of Education and Research

Clinical Trial Protocol

APPLICATION FORM FOR ETHICS APPROVAL

Trial title: Doxycycline for treatment of non-filarial lymphedema due to podoconiosis (PodoLE) - a randomized double blind placebo-controlled trial

TAKeOFF – PodoLEDoxy

Promotor: Federal Ministry of Education and Research (BMBF), Germany

From: Prof. Samuel Wanji
Principal Investigator (PI)
University of Buea, Cameroon

To: The president of the National Ethics Committee for Health Research on Humans

July, 2018

Condential

The content of this document is strictly confidential and may not be copied or made accessible to third parties without the consent of the Institute for Medical Microbiology, Immunology and Parasitology (IMMIP), University Hospital of Bonn, Germany, the Division of Infectious Diseases and Tropical Medicine, Medical Centre of the University of Munich (LMU), Germany and the University of Buea, Cameroon

Title of the project: Doxycycline for treatment of non-filarial lymphedema due to podoconiosis (PodoLE) – a randomized double blind placebo-controlled trial

Short Title: Doxycycline to improve podoconiosis lymphedema

Trial Acronym: TAKeOFF – PodoLEDoxy

Trial Design: randomized double blinded, placebo-controlled, phase II trial

Protocol Code: TAKeOFF-5-0517

Protocol Version: **V** 0.1 / December 15, 2015

Registration Number: ISRCTN11881662

PROJECT INVESTIGATORS AND TRIAL PARTNERS

Sponsor: University of Buea, Cameroon

Principal Investigator: Prof. Samuel Wanji, Public Health Parasitology and Entomology; Department of Microbiology and Parasitology, University of Buea, P.O. Box 63, Buea, Cameroon, **swanji@yahoo.fr**

Institutional Affiliation: Department of Microbiology and Parasitology, Faculty of Science, University of Buea, P.O. Box 63, Buea, Cameroon: Tel.: 237 77 72 43 84

Co-Principal Investigator
Dr. Peter Enyong: Department of Microbiology and Parasitology, Faculty of Science, University of Buea, P.O. Box 63, Buea, Cameroon: Tel.: 237 77 72 43 84

Senior Coordinating Physician: Prof. Dr. Achim Hoerauf: Institute for Medical Microbiology, Immunology and Parasitology (IMMIP); University Hospital of Bonn, Sigmund-Freud-Straße 25, 53127 Bonn, Germany; Tel. No.: +49 228 287 15673, Fax No.: +49 228 287 19573, achim.hoerauf@ukbonn.de

Promotor and source of funding

SPONSORED BY THE
Federal Ministry of Education and Research

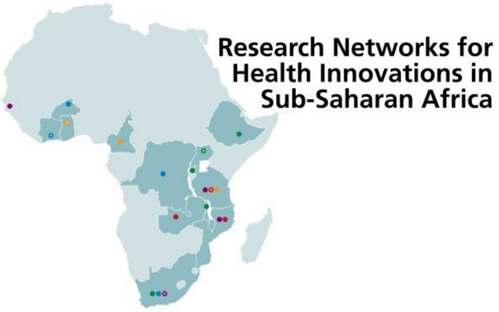

The design, management, analysis and reporting of the study are entirely independent of the manufacturers of doxycycline and placebo.

The Federal Ministry of Education and Research (BMBF), Germany, has no role in the design of this study and will not have any role during its execution, analyses, interpretation of the data, or decision to submit results.

PROTOCOL APPROVAL SIGNATURES

With our signatures we confirm that we are familiar with and understand the protocol and will comply with the principles of Good Clinical Practice (GCP), all applicable local regulations, and the ethical principles laid down in the Declaration of Helsinki.

Principal Investigator

Prof. Samuel Wanji ______________ ___________________________
Date Signature

Co-Principal Investigator

Dr. Peter Enyong ______________ ___________________________
Date Signature

Trial coordinator

Prof. Dr. Achim Hoerauf ______________ ___________________________
Date Signature

Trial clinicians

Dr. Punjom Njefi Yves ______________ ___________________________
Date Signature

Dr. Fozao Afou Adolph ______________ ___________________________
Date Signature

Dr. Djikeussi K. Tatiana ______________ ___________________________
Date Signature

Dr. Vofo Brice ______________ ___________________________
Date Signature

COVER LETTER

From: Samuel Wanji
Principal Investigator (PI)
University of Buea
Faculty of Science
Department of Microbiology and parasitology
P.o. Box 63 Buea, Cameroon
July 28, 2018

To: The president of National Ethics Committee for Health Research on Humans

Dear Sir,

A request for an Ethic Approval of the study protocol for the trial entitled:

"Doxycycline for treatment of non-filarial lymphedema due to podoconiosis (PodoLE) - a randomized double blind placebo-controlled trial"

We have the pleasure to submit to the Ethics Review Committee our request for an ethical approval of the trial protocol entitled **"**Doxycycline for treatment of non-filarial lymphedema due to podoconiosis (PodoLE) - a randomized double blind placebo-controlled trial **".**

We wish to thank the committee for reviewing our research protocol.

We look forward to hearing from you.

Yours sincerely,

Prof. Samuel Wanji

Principal Investigator

TABLE OF CONTENTS

PROJECT INVESTIGATORS AND TRIAL PARTNERS 1

PROTOCOL APPROVAL SIGNATURES 2

COVER LETTER 3

TABLE OF CONTENTS 4

LIST OF ABBREVIATION 12

PART A: TRIAL SUMMARY (ENGLISH AND FRENCH VERSIONS) 14

A1. PROTOCOL SUMMARY (ENGLISH VESION) 15

A2. RESUME DU PROTOCOL (VESION FRANÇAISE) 20

PART B: TRIAL PROTOCOL 25

1. BACKGROUND 26

2. RATIONALE FOR THE TRIAL 27

3. STUDY OBJECTIVES 27

3.1. Primary objectives 27

3.2. Secondary objectives 27

4. TRIAL DESIGN 28

4.1. Design 28

4.2. Participating trial sites 29

4.3. Bias reducing measures 29

4.3.1. Randomization 29

4.3.2. Blinding 29

4.3.3. Assessment of safety 29

4.4. Trial Endpoints 29

4.4.1. Primary Endpoint 29

4.4.2. Secondary Endpoints 29

4.5. Overall timeline of the study 30

4.6. Recruitment 30

4.6.1. Recruitment procedure 30
4.7. Study Population and Eligibility Criteria 31
4.7.1. Gender Distribution 31
4.7.2. Inclusion criteria 31
4.7.3. Exclusion criteria 31
4.7.4. Justification for exclusion of women and children 32
5. TRIAL PROCEDURES 32
5.1. Clinical Evaluation 32
5.1.1. Medical history and physical examination 32
5.1.2. Staging of lymphedema 32
5.1.3. Assessment of acute dermatolymphangioadenitis (ADLA) 34
5.1.4. Circumference measurement of legs using a tape measure 34
5.1.5. Circumference measurement of legs using an infrared scanner (LymphaTech®) 34
5.1.6. Assessment of the leg volume using an infrared scanner (LymphaTech®) 34
5.1.7. Training for care and hygiene of affected legs and arms 34
5.1.8. Ultrasound 35
5.1.9. Clinical photographs 35
5.1.10. Quality of life (QOL) assessment 35
5.2. Laboratory Evaluation 35
5.2.1. Blood 35
5.2.2. Urine 35
5.2.3. Saliva 36
5.3. Schedule of Events 36
5.3.1. Screening (Visit 1) 36
5.3.2. Enrolment (Visit 2) 37
5.3.3. Treatment (Visit 3, day 1 – day 43 + 7 days range) 37
5.3.4. 2 months follow-up 38
5.3.5. 4 months' follow-up 38

5.3.6. 6 months' follow-up 38
5.3.7. Follow-ups at 8, 10, 14, 16, 20 and 22 months after the first day of treatment 39
5.3.8. 18 months' follow-up 39
5.3.9. 12 and 24 months' follow-ups 39
6. INVESTIGATIONAL MEDICINAL PRODUCT (IMP) 40
6.1. General description of the study drug doxycycline 40
6.2. Specification of IMP 40
6.3. Dosage and Route of Administration 40
6.4. Findings from clinical studies 40
6.5. Summary of known and potential risks of doxycycline 40
6.5.1. Side effects of doxycycline....40
6.5.2. General precautions and warnings....42
6.6. Blistering and Labelling of the IMP 42
6.7. Transport of IMP 43
6.8. Handling of IMP at the Site and Drug Accountability 43
6.9. Strategies to improve adherence and compliance.... 43
6.10. Unblinding.... 43
6.11. Prior and Concomitant Therapy/Medication 44
6.11.1. Previous therapy / medication of trial specific illness 44
6.11.2. Previous therapy / medication with doxycycline....44
6.11.3. Prohibited therapy / Concomitant medication 44
7. ADVERSE EVENTS 44
7.1. Documenting, Recording and Reporting Adverse Events.... 44
7.2. Definitions.... 44
7.2.1. Adverse Event (AE) 44
7.2.2. Unexpected Adverse Event (UAE)....45
7.2.3. Adverse (Drug) Reaction (AR)....45
7.2.4. Suspected Adverse (Drug) Reaction (SAR) 45

7.2.5. Unexpected Adverse (Drug) Reaction (UAR) 45
7.2.6. Serious Adverse Event (SAE) 45
7.2.7. Serious Adverse (Drug) Reaction (SAR) 45
7.2.8. Suspected Unexpected Serious Adverse (Drug) Reaction (SUSAR) 45
7.2.9. Unanticipated Problem (UP) 46
7.2.10. Unanticipated Problem that is not an Adverse Event (UPnonAE) 46
7.3. Criteria to be evaluated by the trial clinician 46
7.3.1. Assessment of Intensity 46
7.3.2. Assessment of Adverse Event Intensity for Doxycycline 46
7.3.3. Assessment of Seriousness 48
7.3.4. Assessment of Causality 48
7.4. Adverse Event Recording 48
7.5. Adverse Event Reporting 49
7.6. Serious Adverse Event Reporting 49
7.7. Reporting of Unanticipated Problems (UPs) 49
7.8. Follow-up of Adverse Events 49
7.9. Handling of emergency cases 49
7.10. Deaths 49
7.11. Pregnancies 49
7.11.1. Pregnancy Procedures 50
7.11.2. Pregnancy Reporting 50
8. STATISTICAL CONSIDERATIONS 50
8.1. Hypothesis 50
8.2. Primary target variable 50
8.3. Sample size estimations 51
8.4. Sample size calculation 51
8.5. Achievement of sample size 51
8.6. Populations to be analyzed 51

8.6.1. Intent-to treat (ITT) population 51
8.6.2. Per-Protocol (PP) population: 51
8.6.3. Safety Population 51
8.7. Statistical methodology 51
8.7.1. Analysis of baseline characteristics 52
8.7.2. Analysis of the primary endpoint 52
8.7.3. Analysis of the secondary endpoints 52
8.7.4. Safety Analysis 52
8.8. Interim Analysis 52
8.9. Protocol violations 52
8.10. Handling of Drop-outs, Withdrawal, and Missing Data 53
8.10.1. Screening failures 53
8.10.2. Drop-out/Withdrawal after randomization but before treatment start 53
8.10.3. Drop-out/Withdrawal during or after treatment 53
8.10.4. Missing treatment days 53
8.10.5. Replacement of Patients 53
8.11. Statistical report 53
9. DATA SAFETY AND MONITORING COMMITTEE (DMSC) 53
10. DEFINITION OF END OF TRIAL 54
10.1. Regular end of trial 54
10.2. Termination of the trial for individual subjects 54
10.2.1. Termination by the participant 54
10.2.2. Termination by the investigator 54
10.3. Early termination of the entire trial 54
10.4. Report of termination of the trial 55
10.5. Notification of the end of the trial 55
11. DATA COLLECTION, HANDLING AND RECORD KEEPING 55
11.1. Data Collection methods 55

11.2. Electronic data capture (EDC) 56
11.3. Data management 56
11.4. Trial site file 56
12. MONITORING AND QUALITY ASSURANCE 57
12.1. Study Monitoring 57
12.2. Audits and inspections 57
12.2.1. Archiving by the sponsor 57
12.2.2. Archiving by the investigator 57
13. ETHICAL CONSIDERATIONS 58
13.1. Basic principles 58
13.2. Involvement of Ethics Committees and Regulatory Authorities 58
13.3. Responsibilities of the Investigator 58
13.4. Protocol amendments 59
13.5. Subject information 59
13.6. Obtaining informed consent 60
13.7. Confidentiality 60
13.8. Declaration of interests 60
14. ADMINISTRATIVE CONSIDERATIONS 60
14.1. Patient Insurance 60
14.2. Incentives and expenses 61
14.3. Trial reports 61
14.3.1. Final Report 61
14.4. Publication Policy 61
15. INFORMATION SHEETS 62
15.1. Information sheet for screening and treatment (english version) 62
15.2. Notice d'information pour le depistage et le traitement (version francaise) 68
15.3. Information sheet for sample storage, re-utilization and shipment (english version) 74
15.4. Notice d'information pour le stockage d'echantillons, reutilisation et expedition (version francaise)76

16. INFORMED CONSENT AND ASSENT FORMS 78
16.1. Informed consent form for screening, enrolment and treatment (english version) 78
16.2. Fiche de consentement éclairé pour le depistage, le recrutement et le traitement (version francaise) 81
16.3. Informed consent forms for sample storage, re-utilization and shipment (english version) 84
16.4. Fiche de consentement éclairé pour le stockage d'echantillons, reutilisation et expedition (version francaise)87
16.5. Assent form for screening, enrolment and treatment (english version) 90
16.6. Fiche d'assentiment pour le depistage, le recrutement et le traitement (version francaise) 93
16.7. Assent form for sample storage, re-utilization and shipment (english version) 96
16.8. Fiche d'assentiment pour le stockage d'echantillons, reutilisation et expedition (version francaise) 99
17. CASE REPOR FORMS (CRFs) / Questionaire 103
18. CHRONOGRAM OF ACTIVITIES 226
19. BUDGET AND SOURCE OF FUNDING 228
19.1. Proposed budget 228
19.2. Source of funding 228
20. REFERENCES 229
PART C: ADDITIONAL DOCUMENTS 231
C-1 INVESTIGATOR'S CVs 232
CV PRICIPAL INVESTIGATOR 232
CV CO-INVESTIGATOR 241
CV TRIAL COORDINATOR 242
CV TRIAL CLINITIAN 1 245
CV TRIAL CLINITIAN 2 249
CV TRIAL CLINITIAN 3 252
CV TRIAL CLINITIAN 4 253
C-2 INVESTIGATOR'S BROCHURE 254
C-3 INSURANCE 277

C-3.1 Insurance for participants 277

C-3.2 Insurance for trial clinitians (Medical doctors) 289

C-4 MATERIAL TRANSFERT AGREEMENT 307

C-5 DATA SHARING AGREEMENT 309

C-6 FRAMEWORK CONVENTION 313

C-7 RECEIPT OF PAYEMENT OF SUBMISSION FEES 319

C-8 AUTHORIZATION FOR STUDY SITE 320

LIST OF ABBREVIATION

ADLA	adenolymphangioadenitis
AE	Adverse events
AE CRF	Adverse Event Case Report Form
ALB	Albendazole
ALT	Alanine aminotransferase
AR	Adverse (Drug) Reaction
AST	Aspartate aminotransferase
BUN	Blood urea nitrogen
CDAD	Clostridium difficile-associated diarrhea
CECAM-a	Carcino-embryonic antigen-related cell adhesion molecule
CHP	Community-Based Health Planning and Services
CIOMS	Council for International Organizations of Medical Sciences
CNS	Central nervous systm
CRF	Case Report Form
CRO	Contract Research Organization
DOT	Daily observed treatment
DOX	Doxycycline
DOX 200	doxycycline 200mg/d
DROS	Division for Health Operations Research
DSMB	Data and Safety Monitoring Board
DSMC	Data Safety Monitoring Committee
eCRF	Electronic Case Report Form
EDC	Electronic data capture
FDA	Food and Drug Authorities
FTS	Filariasis test strip
GCP	Good Clinical Practice
GLP	Good Laboratory Practise
HIPAA	Health Insurance Portability and Accountability Act
IB	Investigator's Brochure
ICF	Informed Consent Form
ICH	International Conference on Harmonisation
ICT	Immunochromatographic test
IEC	Independent or Institutional Ethics Committee
IMP	Investigational Medicinal Product
IND	Investigational New Drug Application
IRB	Institutional Review Board
ISF	Trial site file
ITT	Intention to treat
IVM	Ivermectin
LANACOME	Laboratoire National de Contrôle de qualité des Médicaments et d'Expertise
LE	Lymphodema
LF	Lymphatic Filariasis

M	Mossy Changes(+ or -)
MDA	Mass Drug Administration
MedDRA®	Medical Dictionary for Regulatory Activities
miRNA	Micro Ribonucleic Acid
MMPS	Matrix metalloproteinases
MOP	Manual of Procedures
N	Number (typically refers to subjects)
NECHRH	National Ethics Committee for Health Research on Humans
PI	Principal Investigator
PK	Pharmacokinetics
Podo	Podoconiosis
PodoLE	Lymphedema due to podoconiosis
PP	Per-Protocol population
QA	Quality Assurance
QC	Quality Control
QOL	Quality of life
qPCR	Quantitative polymerase chain reaction
REDCap	Research Electronic Data Capture
SAE	Severe or Serious Adverse events
SAF	Safety population
SAP	Statistical Analysis Plan
SAR	Serious Adverse (Drug) Reaction
SAS	Statistical Analysis System
SMC	Safety Monitoring Committee
SmPC	Summary of Product Characteristics
SOP	Standard Operating Procedure
SUSAR	Suspected Unexpected Serious Adverse (Drug) Reaction
TAKeOFF	Tackling the Obstacles to Fight Filariasis and Podoconiosis
TBF	Thick blood film
UAE	Unexpected Adverse Event
UAR	Unexpected Adverse (Drug) Reaction
UK	United Kingdom
UP	Unanticipated Problem
UPnonAE	Unanticipated Problem that is not an Adverse Event
URTI	Upper respiratory tract infection
VEGF	Vascular endothelial growth factor
wb123	*Wuchereria bancrofti* 123 (recombinant antigen)
γ-GT	Gamma-glutamyl transferase

PART A: TRIAL SUMMARY (ENGLISH AND FRENCH VERSIONS)

A1. PROTOCOL SUMMARY (ENGLISH VESION)

**Protocol Title**	Doxycycline for treatment of non-filarial lymphedema due to podoconiosis (PodoLE) - a doubled blind randomized placebo-controlled trial
**Phase of trial**	PodoLE stages 2-4: Phase II
**Short Title**	Doxycycline to improve PodoLE
**Trial Acronym**	TAKeOFF – PodoLEDoxy
**Protocol Code**	TAKeOFF-5-0517
**Trial registration**	ISRCTN: ISRCTN11881662
**Sponsor**	University of Buea
**Source(s) of monetary or material support**	Research Networks for Health Innovations in Sub-Saharan Africa<br>SPONSORED BY THE Federal Ministry of Education and Research
**Health condition**	Podoconiosis (Podo)
**Study Centre**	North West Region, Cameroon
**Interventions**	**Treatment "DOX 200": Doxycycline 200 mg/days for 6 weeks**<br>**Treatment "Placebo"** (control): **Placebo matching Doxycycline for 6 weeks**<br>Treatment will be administered *ad persona* by the trial clinician directly at the Bafut District Hospital, North-West Cameroon in the form of daily observed treatment (DOT)
**Investigational medicinal product, Dose and Mode of Application**	**Treatment "DOX 200":**<br>Trade Name: Remycin®<br>Substance: Doxycycline<br>Manufacturer: Remedica, Cyprus<br>Dose: 2 tablets of 100mg each /day<br>Mode of administration: oral<br>Duration of treatment: 6 weeks (42 days)<br>On/off label use: off label (not registered as drug for the treatment of podoconiosis)<br><br>**Treatment "Placebo",** control:<br>Substance: Placebo matching doxycycline 100 mg containing no active ingredients<br>Manufacturer: Piramal, UK<br>Dose: 2 tablets of placebo matching doxycycline 100mg/day<br>Mode of administration: oral<br>Duration of treatment: 6 weeks (42 days)<br><br>Verum and Placebos will be blistered and labelled by Piramal, Pharma Solutions, UK.
**Trial Population**	Healthy adults (18 – 65 years) with lymphedema stage 2-4 due to podoconiosis (PodoLE).<br>Staging will be carried out using the 5-point scale published by Tekola *et al.*[1].

**Trial Design**	Method of allocation: randomized<br>Masking: double-blind (caregiver, participants, outcome assessors)<br>Control: placebo-controlled<br>Assignment: parallel<br>Purpose: Efficacy of doxycycline treatment lymphedema
**Rationale of the trial**	The previously demonstrated [2, 3] effect of doxycycline in reversing or stopping the progression of filarial lymphedema (LE) in patients with LE stage 1-3, irrespective of their filarial infections being active or not, leads to the assumption that the same effect could also be expected in patients with LE due to podoconiosis (PodoLE). Therefore, this trial is designed to show efficacy of doxycycline (200mg/d for 6 weeks) in patients with PodoLE stage 2-4 [1].
**Trial Objectives**	**Primary Objective:**<br>To show efficacy of a 6-week course of daily doxycycline 200 mg in reversing or altering the progression of lymphedema due to ppodoconiosis.<br>**Secondary Objectives:**<br>• to show efficacy on improvement of PodoLE<br>• to evaluate changes in the circumference of the affected limbs measured with tape<br>• to evaluate changes of skin thickness of the affected limbs measured by ultrasound<br>• to evaluate changes in the circumference of the affected limbs measured with an infrared scanner (LymphaTech®)<br>• to evaluate changes in the volume of the affected limbs measured with an infrared scanner (LymphaTech®)<br>• to evaluate changes of the frequency of acute attacks of dermato-lymphangioadenitis (ADLA)<br>• to evaluate changes in hygiene behaviour<br>• to evaluate changes in the quality of life (QoL)<br>• to measure biomarkers (angiogenic, lymphangiogenic, pro-fibrotic or pro-inflammatory such as VEGF, CECAM-a, MMPS) in blood and/or urine that are responsible for the progression of PodoLE<br>• to assess the tolerability and safety of doxycycline 200mg/d for 6 weeks
**Trial Endpoints**	**Primary efficacy endpoint:**<br>• Lack of progression of PodoLE (stage reduction or same stage as pre-treatment using the 5-point scale staging according to Tekola *et al*,. 2008) examined 24 months after treatment onset<br>**Secondary endpoint(s):**<br>• Lack of progression of PodoLE (stage reduction or same stage as pre-treatment using the 5-point scale staging according to Tekola *et al*,. 2008) examined 6 or 12 months after treatment onset<br>• Improvement of PodoLE, i.e. stage reduction (at least one stage compared to pre-treatment) examined 6, 12 and 24 months after treatment onset<br>• Change of PodoLE stages (reduction or increase) compared to baseline assessed at 6, 12 and 24 months after treatment onset

	• Changes of the circumference of the affected limbs compared to baseline circumferences measured by measuring tape at 6, 12 and 24 months after treatment onset<br>• Changes of skin thickness of the affected limbs compared to baseline values measured by ultrasound at 6, 12 and 24 months after treatment onset<br>• Changes of the circumference of the affected limbs compared to baseline circumferences measured with an infrared scanner (LymphaTech®) at 6, 12 and 24 months after treatment onset<br>• Changes of the volume of the affected limbs compared to baseline volume measured with an infrared scanner (LymphaTech®) at 6, 12 and 24 months after treatment onset<br>• Changes in the duration of acute attacks compared to pre-treatment evaluated at 6, 12 and 24 months after treatment onset<br>• Changes in the frequency of acute attacks compared to pre-treatment evaluated at 6, 12 and 24 months after treatment onset<br>• Absence of acute attacks evaluated at 6, 12 and 24 months after treatment onset<br>• Changes of the hygiene level compared to pre-treatment assessed at 6, 12 and 24 months<br>• Changes of the quality of life (QoL) compared to pre-treatment assessed at 12 and 24 months after treatment onset<br>• Changes in levels of angiogenic, lymphangiogenic, pro-fibrotic or pro-inflammatory biomarkers (such as VEGF, CECAM-a, MMPS) in blood and/or urine compared to baseline values as a measure for prognostic effects assessed 6, 12 and 24 months after treatment onset<br>**Assessment of safety:**<br>Adverse events (AE) will be assessed and described in the scope of the daily observed treatment (DOT). This involves a) occurrence of AE, b) intensity of AE (Grade 0 (none), Grade 1 (mild), grade 2 (moderate) grade 3 (severe), c) SAE, d) relation to treatment (definite, probable, possible, remote, not related), e) outcome of AE (restored, improved, unchanged, deteriorated, death, unknown, overcome with sequelae, f) intervention.
**Subject Number**	To be screened for eligibility: n ≈ 500<br>To be allocated to trial (after meeting inclusion criteria):<br>n = 200 (n = 100 per intervention group: treatment or placebo)
**Inclusion Criteria**	• Lymphedema due to podoconiosis of at least one leg stage 2-4 measured on a 5-point scale [4]<br>• Age ≥ 18 years and ≤ 65 years<br>• Men or non-pregnant women. Women of childbearing-potential must use an approved, effective method of contraception (including abstinence) before, during and for at least 2 weeks after the completion of the active intervention with doxycycline or placebo<br>• Negative pregnancy test<br>• Body weight ≥ 40 kg<br>• Resident in endemic area of podoconiosis for ≥ 2 years<br>• Able and willing to give informed consent to participate in the trial

	• Ability to use established standardized methods of hygiene and effectively applying it prior to the initiation of the drug treatment<br>• Negative test for lymphatic filariasis (LF)
**Exclusion Criteria**	• No lymphedema, stage 1 or stage 5 lymphedema due to podoconiosis or lymphedema due to LF<br>• Age < 18 years or > 65 years<br>• Body weight < 40 kg<br>• Pregnant or breastfeeding women<br>• Women of childbearing potential not using an agreed method of contraception (including abstinence; oral contraceptives are not allowed because of interaction with trial drugs)<br>• Clinical or biologic evidence of hepatic or renal dysfunction or disease of the central nervous system (CNS)<br>• Evidence of severe comorbidities except for features of podoconiosis<br>• Alcohol or drug abuse<br>• History of adverse reactions to doxycycline or other tetracyclines<br>• Any significant condition (including medical and psychological/psychiatric disorder) which in the opinion of the study investigator might interfere with the conduct of the study<br>• History of photosensitivity reactions after taking drugs.<br>• Concomitant medication with antacids containing aluminium, magnesium or sucralfate and not able to discontinue<br>• Concomitant medication with other antibiotics than doxycycline and not able to discontinue<br>• Concomitant medication with loop diuretics or sulfonylurea<br>• Concomitant medication with coumarin
**Laboratory Exclusion Criteria**	• Haemoglobin < 8 gm/dL<br>• Neutrophil count <2 000/mm^3<br>• Platelet count <100 000/mm^3<br>• Creatinine > 2 times upper limit of normal<br>• AST (GOT) > 2 times upper limit of normal<br>• ALT (GPT) > 2 times upper limit of normal<br>• y-GT > 2 times upper limit of normal<br>• Positive urine pregnancy test<br>• Positive FTS result
**Trial Specific Measurements**	• Laboratory assessments<br>• Lymphedema staging on a 5 point scale as described by Tekola [4]<br>• Circumference of the legs measured by tape<br>• Skin thickness of the ankles measured by ultrasound<br>• Circumference and volume of the legs measured with an infrared scanner (LymyphaTech®)<br>• ADLA questionnaires<br>• Hygiene assessment<br>• Quality of Life (QoL) assessment

**Statistical Rationale**	**Primary statistical Analysis:**<br>The frequencies of the participants with "lack of PodoLE progression" and their confidence intervals (95%) will be calculated and compared between the two treatment groups using Fisher's exact test with an α-level of 5%.<br>**Secondary Endpoints:**<br>Secondary endpoints will be described as estimators with confidence intervals (95%) for each intervention group and analysed with adequate statistical methods.<br>**Safety Analysis:**<br>Frequency of Adverse events and Serious Adverse Events will be analysed. In this analysis all patients will be included who took the drugs at least for one day.
**Trial procedures**	There will be 15 visits in total (see schedule of activities):<br>Visit 1: Screening<br>Visit 2: Baseline<br>Visit 3: Treatment (42 days)<br>Visit 4: Follow-up 2 months after treatment onset<br>Visit 5: Follow-up 4 months after treatment onset<br>Visit 6: Follow-up 6 months after treatment onset<br>Visit 7: Follow-up 8 months after treatment onset<br>Visit 8: Follow-up 10 months after treatment onset<br>Visit 9: Follow-up 12 months after treatment onset<br>Visit 10: Follow-up 14 months after treatment onset<br>Visit 11: Follow-up 16 months after treatment onset<br>Visit 12: Follow-up 18 months after treatment onset<br>Visit 13: Follow-up 20 months after treatment onset<br>Visit 14: Follow-up 22 months after treatment onset<br>Visit 15: Follow-up 24 months after treatment onset
**Study Timeline**	Estimated date of first participant enrolled: Quarter 01/2018<br>Estimated date of last participant enrolled: Quarter 03/2018<br>Estimated date of last participant completed: Quarter 04/2020<br>**Total duration of study:**<br>33 months (9 months enrolment period + 6 weeks treatment period + 24 months follow-up after treatment start)

A2. RESUME DU PROTOCOL (VESION FRANÇAISE)

**Titre du protocole**	Doxycycline 200 mg/d pendant 6 semaines pour le traitement du lymphœdème non-filarienne dû à la podoconiose (PodoLE) - Un essai randomisé, contrôlé en double aveugle avec placebo
**Phase of essai**	PodoLE stades 2-4: Phase II
**Short Titre**	Doxycycline pour améliorer le lymphœdème dû à la podoconiose
**Acronyme de l'essai**	TAKeOFF – PodoLEDoxy
**Code du protocole**	TAKeOFF-5-0517
**Numéro d'enregistrement de l'essai**	ISRCTN: ISRCTN11881662
**Sponsor**	Université de Buea
**Source(s) de financement**	Research Networks for Health Innovations in Sub-Saharan Africa<br>SPONSORED BY THE<br>Federal Ministry of Education and Research
**Condition sanitaire**	Podoconiose (Podo)
**Centre d'étude**	Région du Nord-Ouest, Cameroun
**Interventions**	**Traitement "DOX 200": Doxycycline 200 mg/d pendant 6 semaines**<br>**Traitement** "**Placebo**" (contrôle): **Placebo correspondant à la Doxycycline pendant 6 semaines**<br>Les traitement seront administrés *ad personam* par le médecin de l'essai directement dans l'Hopital de district de Bafut au NordOuest du Cameroun sous forme de traitement quotidien observé (DOT)
Médicament de l'essai, dose et mode d'application	**Traitement "DOX 200":**<br>Nom commercial: Remycin®<br>Substance: Doxycycline<br>Fabriquant: Remedica, Cyprus<br>Dose: 2 comprimés á 100 mg/jour<br>Mode d'application: oral<br>Durée de traitement: 6 semaines (42 jours)<br>Utilisation de l'étiquette On/off: étiquette off (non enregistré pour le traitement de la podoconiose)<br><br>**Traitement "Placebo",** contrôle:<br>Substance : Placebo correspondant a la doxycycline 100 mg ne contenant pas de principe actif<br>Fabriquant : Piramal, Royaume Unies<br>Dose: 2 comprimés placebo correspondant à la doxycycline 100mg/jour<br>Mode d'application: oral<br>Durée de traitement: 6 semaines (42 jours)

	Verum et Placebos seront masqués et étiquetés par Piramal, Pharma Solutions, Royaume Unies.
**Population de l'essai**	Adultes sains (18 – 65 ans) avec du lymphœdème au stade 2-4 due à la podoconiose (PodoLE).<br>La détermination du stade de lymphœdème se fera en utilisant l'échelle de 5 points publiée par F Tekola, Z Ayele, D HaileMariam, C Fuller and G Davey [1].
**Conception de l'essai**	Méthode d'allocation : randomisé<br>Masquage : en double aveugle (soignant, participants, évaluateurs de résultats)<br>Contrôle : placebo<br>Affectation : parallèle<br>But: Efficacité
**Contexte et justification de l'essai**	L'effet précédemment démontré (Debrah et al., 2006, Mand et al., 2012) de la doxycycline dans la régression ou l'arrêt de la progression du lymphœdème filarienne (LE) chez les patients avec LE stade 1-3, indépendamment du fait que leurs infections filariennes soient actives ou non, a conduit à l'hypothèse selon lequel le même effet pourrait également être attendu chez les patients avec du LE dû à la podoconiose (PodoLE). Par conséquent, cet essai est conçu pour montrer l'efficacité de la doxycycline (200 mg / j pendant 6 semaines) chez les patients avec du PodoLE stade 2-4 (Tekola et al., 2008).
**Objectifs de l'essai**	**Objectif principal :**<br>Montrer l'efficacité de 6 semaines de prise quotidienne de doxycycline 200 mg sur l'échec de progression du PodoLE<br>**Objectifs secondaires :**<br>• Montrer l'efficacité de la doxycycline sur l'amélioration du PodoLE<br>• Mesurer les changements de la circonférence des membres affectées mesurés avec à l'aide d'un ruban mètre<br>• Mesurer les changements de l'épaisseur de la peau des membres affectées mesurés avec à l'aide d'un scanner à ultrason<br>• Mesurer les changements de la circonférence des membres affectées mesurés avec à l'aide d'un scanner infrarouge (LymphaTech®)<br>• Evaluer les variations des fréquences des épisodes des attaques aigues de dermatolymphangioadénite.<br>• Evaluer les changements du comportement hygiénique<br>• Evaluer les changements de la qualité de vie (QV)<br>• Mesurer les biomarqueurs (angiogéniques, lymphangiogéniques, pro-fibrotiques or pro-inflammatoires tels que VEGF, CECAM-a, MMPS) dans le sang et/ou urine responsables de la progression PodoLE<br>• Evaluer la tolérabilité et l'innocuité de la doxycycline 200 mg / j pendant 6 semaines
**Points d'achèvement de l'essai**	**Point d'achèvement d'efficacité primaire:**<br>• Manque de progression du PodoLE (réduction du stade ou même stade que celui avant traitement en utilisant un échelle a 5 point tel que décrit par Tekola *et al*,. 2008), examinée 24 mois après le début de traitement<br>**Points d'achèvement secondaires:**

	• Manque de progression de PodoLE (réduction de stade ou même stade de prétraitement en utilisant la mise en scène à échelle de 5 points selon Tekola et al., 2008) examinée 6 ou 12 mois après le début du traitement<br>• Amélioration de PodoLE, c'est-à-dire réduction du stade (au moins une étape par rapport au prétraitement) examinée 6, 12 et 24 mois après le début du traitement<br>• Changement des stades PodoLE (réduction ou augmentation) par rapport aux valeurs initiales évaluées à 6, 12 et 24 mois après le début du traitement<br>• Modifications de la circonférence des membres affectés par rapport aux circonférences de base mesurées par un ruban mètre à 6, 12 et 24 mois après le début du traitement<br>• Modifications de l'épaisseur de la peau des membres affectés par rapport aux valeurs initiales mesurées par échographie à 6, 12 et 24 mois après le début du traitement<br>• Modifications de la circonférence des membres affectés par rapport aux circonférences de base mesurées avec un scanner infrarouge (LymphaTech®) à 6, 12 et 24 mois après le début du traitement<br>• Modifications du volume des membres affectés par rapport au volume de base mesuré avec un scanner infrarouge (LymphaTech®) à 6, 12 et 24 mois après le début du traitement<br>• Modifications de la durée des crises aiguës par rapport au prétraitement évalué à 6, 12 et 24 mois après le début du traitement<br>• Évolution de la fréquence des crises aiguës par rapport au pré-traitement évalué à 6, 12 et 24 mois après le début du traitement<br>• Absence d'attaques aiguës évaluées à 6, 12 et 24 mois après le début du traitement<br>• Changements du niveau d'hygiène par rapport au prétraitement évalué à 6, 12 et 24 mois<br>• Changements de la qualité de vie (QV) par rapport au prétraitement évalué à 12 et 24 mois après le début du traitement<br>• Changements des taux de biomarqueurs angiogéniques, lymphangiogéniques, pro-fibrotiques ou pro-inflammatoires (tels que VEGF, CECAM-a, MMPS) dans le sang et / ou l'urine par rapport aux valeurs de référence comme mesure des effets pronostiques évalués 6, 12 et 24 mois après le début du traitement<br><br>**Évaluation de la sécurité** :<br>Les effets indésirables (EI) seront évalués et décrits dans le cadre du traitement quotidien observé (DOT). Cela implique a) l'apparition de l'EI, b) l'intensité de l'EI (Grade 0 (aucun), Grade 1 (léger), Grade 2 (modéré) grade 3 (sévère), c) effets indésirables sévères (SAE), d) relation au traitement (défini, probable, possible, éloigné, non lié), e) résultat de l'EI (rétabli, amélioré, inchangé, détérioré, décès, inconnu, vaincu avec des séquelles, f) intervention
**Nombre de sujets**	Pour l'éligibilité : n ≈ 500<br>A attribuer à l'essai (après avoir rempli les critères d'inclusion):<br>n = 200 (n = 100 par groupe d'intervention)

**Critères d'exclusion**	• Pas de lymphœdème, lymphœdème de stade 1 ou 5 due à la podoconiose ou lymphœdème due à la filariose lymphatique<br>• Age < 18 and ou > 65 and<br>• Poids corporel < 40 kg<br>• Femme enceinte ou allaitante<br>• Femme d'âge reproductrice n'utilisant aucune méthode contraceptive recommandée telle que l'abstinence (les contraceptifs oraux ne sont pas autorisés pour éviter l'interaction avec les médicaments de l'essai)<br>• Preuve clinique ou biologique d'un dysfonctionnement hépatique ou rénal ou d'une maladie du système nerveux central (SNC)<br>• Preuve de comorbidités graves sauf pour les caractéristiques de la podoconiose<br>• L'abus d'alcool ou de drogue<br>• Antécédents de réactions indésirables à la doxycycline ou à d'autres tétracyclines<br>• Toute condition importante (y compris les troubles médicaux et psychologiques / psychiatriques) qui, de l'avis de l'investigateur de l'étude, pourrait interférer avec la conduite de l'étude<br>• Antécédents de réactions de photosensibilité après la prise de médicaments.<br>• Médicament concomitant avec des antiacides contenant de l'aluminium, du magnésium ou du sucralfate et ne pouvant pas arrêter<br>• Médicament concomitant avec d'autres antibiotiques que la doxycycline et ne pouvant pas arrêter<br>• Médicament concomitant avec des diurétiques de l'anse ou une sulfonylurée<br>• Médicament concomitant avec la coumarine
**Critères d'exclusions du laboratoire**	• Hémoglobine < 8 gm/dL<br>• Neutrophiles <2 000/mm^3<br>• Plaquettes <100 000/mm^3<br>• Créatinine > 2 fois supérieure a la borne supérieure de l'intervalle de référence<br>• AST (GOT) > 2 fois supérieure à la borne supérieure de l'intervalle de référence ALT (GPT) > 2 fois supérieure à la borne supérieure de l'intervalle de référence<br>• y-GT > 2 fois supérieure à la borne supérieure de l'intervalle de référence<br>• Test de grossesse positif sur echantillon d'urine<br>• Test positif de FTS pour *W. bancrofti*
**Mesures spécifiques de l'essai**	• Évaluations de laboratoire<br>• Stadification du lymphoedème sur une échelle de 5 points telle que décrite par Tekola (Tekola et al., 2008)<br>• Circonférence des jambes mesurée par ruban mètre<br>• Épaisseur de la peau des chevilles mesurée par échographie<br>• Circonférence et volume des jambes mesurées avec un scanner infrarouge (LymyphaTech®)<br>• Questionnaires ADLA<br>• Évaluation de l'hygiène<br>• Évaluation de la qualité de vie (QV)
**Justification statistique**	Analyses statistiques primaires :<br>Les fréquences des participants avec «manque de progression PodoLE» et leurs intervalles de confiance (95%) seront calculées et comparées entre les deux groupes de traitement en utilisant le test exact de Fisher avec un taux α de 5%.

	Critères secondaires :<br>Les critères d'évaluation secondaires seront décrits comme des estimateurs avec des intervalles de confiance (95%) pour chaque groupe d'intervention et analysés avec des méthodes statistiques adéquates.<br>Analyse de sécurité :<br>La fréquence des événements indésirables et des événements indésirables graves sera analysée. Tous les patients ayant pris du médicament au moins pendant une journée seront inclus dans cette analyse.
**Procédures de l'essai**	Au total, 15 visites sont programmées (voir calendrier des activités):<br>Visite 1: Screening<br>Visite 2: Baseline<br>Visite 3: Traitement (42 jours)<br>Visite 4: Suivi 2 mois après le début de traitement<br>Visite 5: Suivi 4 mois après le début de traitement<br>Visite 6: Suivi 6 mois après le début de traitement<br>Visite 7: Suivi 8 mois après le début de traitement<br>Visite 8: Suivi 10 mois après le début de traitement<br>Visite 9: Suivi 12 mois après le début de traitement<br>Visite 10: Suivi 14 mois après le début de traitement<br>Visite 11: Suivi 16 mois après le début de traitement<br>Visite 12: Suivi 18 mois après le début de traitement<br>Visite 13: Suivi 20 mois après le début de traitement<br>Visite 14: Suivi 22 mois après le début de traitement<br>Visite 15: Suivi 24 mois après le début de traitement
**Durée de l'essai**	Date estimée pour l'inscription du premier participant : Trimestre 01/2018<br>Date estimée pour l'inscription du dernier participant : Trimestre 03/2018<br>Date estimée pour la fin du dernier participant : Trimestre 04/2020<br>Durée totale de l'essai :<br>33 mois (9 mois période d'inscription + 6 semaines période de traitement + 24 mois de suivi après le début du traitement)

PART B: TRIAL PROTOCOL

1. BACKGROUND

Podoconiosis (endemic non-filarial elephantiasis) is a non-infectious geochemical disease arising in barefoot subsistence farmers who are in long-term contact with irritant red clay soil of volcanic origins [5, 6]. The disease causes progressive bilateral swelling of the lower legs. Mineral particles absorbed through skin are taken up by macrophages into the lymphatic system and result in an inflammatory process leading to fibrosis and obstruction of the vessels. This leads initially to swelling of the foot and the lower leg, which progresses to elephantiasis: gross lymphoedema with mossy and nodular changes of the skin. Podoconiosis affects some 4 million people in Africa, Latin America, and a few areas of Asia. It is found in more than ten countries across tropical Africa where irritant soils have been generated by environmental conditions of high altitude (>1,000m) and high annual rainfall (>1,000mm), and are farmed by very poor people who cannot afford shoes or water. Podoconiosis has been described in ten countries across tropical Africa, and has also been reported in tropical areas of Central America and Northern India. The exact global burden is still to be measured, but it is estimated that at least 4 million people are affected worldwide. Cameroon is thought to be among the countries with the highest burden of podoconiosis [7, 8]. Due to lack of understanding of the geographical distribution of the disease, intervention against the disease is minimal.

The current control strategy for podoconiosis consists of integrated approach prevention among school age children through promotion and distribution of protective shoe and lymphedema management. Current treatment practices of LE rely on decreasing the number of acute attacks by improving the hygiene of affected limbs, use of appropriate topical antibiotics and antifungals, exercise, elevation of the limb and use of footwear. While this treatment package has been shown to be effective in halting the progression of LE, it requires sustained access to resources required for limb care and strict adherence to the prescribed procedures.

The anti-*Wolbachia* effects of doxycycline and its potential role in filarial infections have been well documented [9, 10]. Recent observations have suggested a potential role for lymphatic endothelium-derived VEGF-C and sVEGFR3 and other angiopoietic factors in the pathogenesis of LE that could be lowered by doxycycline [2, 11]. A six- week course of doxycycline 200 mg daily (DOX 200) prevented progression of LE in patients with active infection of *W. bancrofti* [2]. More recently, in a trial in Ghana, a similar course of doxycycline (DOX 200) decreased severity of mild to moderate LE independent of active filarial infection [3].

The effect of DOX 200 in reversing or stopping the progression of LE of patients with stage 1-3, irrespective of their filarial infections being active or not, leads to the assumption that the same effect could also be expected in patients with LE due to podoconiosis (PodoLE). Therefore, this trial is designed to show efficacy of doxycycline (200mg/d for 6 weeks) in patients with PodoLE stage 2-4 [1].

2. RATIONALE FOR THE TRIAL

Current lymphedema management protocols are based on the use of simple measures of hygiene (regular washing with soap and water, skin and nail care), use of topical antibiotics or antifungal agents, exercise and footwear. Previous controlled clinical trials and extensive field experience have shown the benefit of these measures in reducing the frequency of attacks of acute dermato-lymphangio-adenitis (ADLA) that drive the progression of LE [12]. In most endemic countries they now represent the available "standard of care" in the absence of any structured treatment programs for the management of lymphedema of LF.

In the present study, the progression of PodoLE in a group of patients who receive a six-week course of doxycycline 200mg/d (DOX 200) will be compared with that of a group who receives doxycycline "look-alike" placebo tablets. However, both groups will be enrolled into a standardized "regimen of hygiene" described above. Thus, patients enrolled in the "placebo" group also will receive the current "standard of care", and the placebo used in the study will help to identify the benefits of DOX 200 on a background of simple hygiene measures. The regimens will be explained to all participants who will be trained to use established standardized methods of hygiene and be effectively applying it prior to the initiation of the drug treatment. In addition, patients will be retrained at every contact point (4, 6, 12 and 18 months). A common, generic SOP with handouts that describes methods and the training schedule will be used so that similar methods are employed across all communities.

After unblinding and data analysis, all participants will be offered doxycycline treatment if the intervention proves to be more effective in ameliorating LE.

3. STUDY OBJECTIVES

3.1. Primary objectives

- To show the efficacy of a 6-week course of daily doxycycline 200 mg on lack of progression of PodoLE stages 2-4.

3.2. Secondary objectives

- to show efficacy on improvement of PodoLE
- to evaluate changes in the circumference of the affected limbs measured with tape
- to evaluate changes of skin thickness of the affected limbs measured by ultrasound
- to evaluate changes in the circumference of the affected limbs measured with an infrared scanner (LymphaTech®)
- to evaluate changes in the volume of the affected limbs measured with an infrared scanner (LymphaTech®)
- to evaluate changes of the frequency of acute attacks of deramtolymphangioadenitis (ADLA)
- to evaluate changes in hygiene
- to evaluate changes in the quality of life (QoL)

- to measure biomarkers (angiogenic, lymphangiogenic, pro-fibrotic or pro-inflammatory such as VEGF, CECAM-a, MMPS) in blood and/or urine that are responsible for the progression of PodoLE
- to assess the tolerability and safety of doxycycline 200 mg/d for 6 weeks
-

4. TRIAL DESIGN

4.1. Design

This trial is planned as a prospective, randomized, placebo-controlled, double-blind, parallel-group, interventional confirmatory II trial.

The trial will be carried out as illustrated in Figure 1.

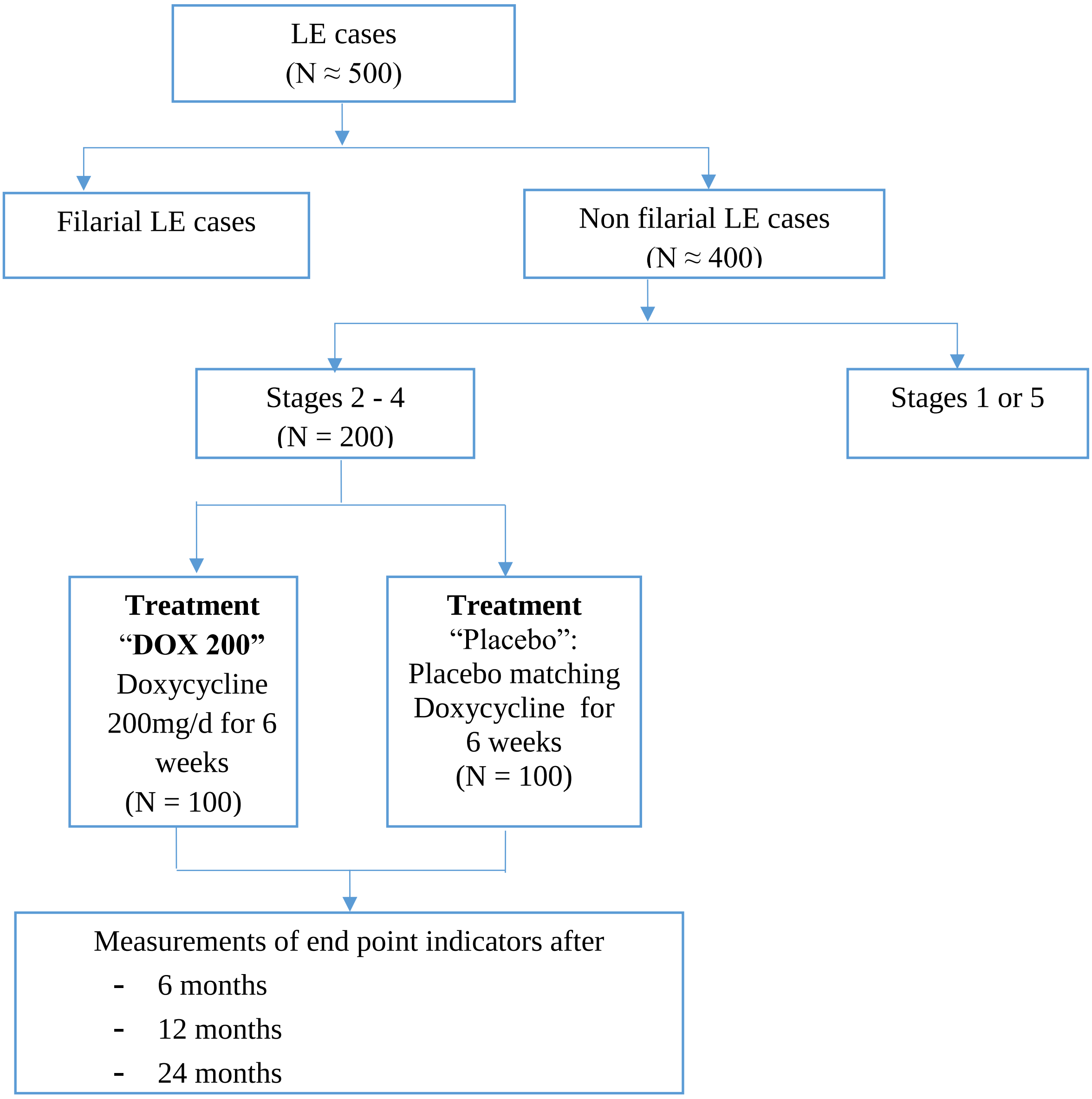

Figure 1. Flow chart of the study

4.2. Participating trial sites

The trial will be conducted in communities of the Districts in the Nord West Region of Cameroon where podoconiosis is endemic. Recent mapping showed a more than 1000 cases of podoconiosis lymphedema in the communities of this region [13].

4.3. Bias reducing measures

4.3.1. Randomization

The randomization lists will be generated by the manufacturer of the study drugs (Piramal, UK) using block randomization. Consenting subjects will be allocated to treatment sequentially according to the randomization list with treatment allocation being provided in individual envelopes.

4.3.2. Blinding

Trial participants, care providers, and outcome assessors as well as the data management located in Bonn, Germany will be blinded to the drugs received by the patients.

4.3.3. Assessment of safety

Adverse events (AE) will be assessed and described in the scope of the daily observed treatment (DOT). This involves a) occurrence of AE, b) intensity of AE (Grade 0 (none), Grade 1 (mild), grade 2 (moderate) grade 3 (severe), c) SAE, d) relation to treatment (definite, probable, possible, remote, not related), e) outcome of AE (restored, improved, unchanged, deteriorated, death, unknown, overcome with sequelae, f) intervention.

4.4. Trial Endpoints

4.4.1. Primary Endpoint

- Lack of progression of PodoLE (stage reduction or same stage as pre-treatment using the 5-point scale staging according to Tekola *et al.* [1]) examined 24 months after treatment onset

4.4.2. Secondary Endpoints

- Lack of progression of PodoLE (stage reduction or same stage as pre-treatment using the 5-point scale staging according to Tekola *et al.* [1]) examined 6 or 12 months after treatment onset
- Improvement of PodoLE, i.e. stage reduction (at least one stage compared to pre-treatment using the 5-point scale staging according to Tekola *et al,.* [1]) examined 6, 12 and 24 months after treatment onset
- Change of PodoLE stages (reduction or increase) compared to baseline assessed at 6, 12 and 24 months after treatment onset
- Changes of the circumference of the affected limbs compared to baseline circumferences measured by tape measure at 6, 12 and 24 months after treatment onset
- Changes of skin thickness of the affected limbs compared to baseline values measured by ultrasound at 6, 12 and 24 months after treatment onset
- Changes of the circumference of the affected limbs compared to baseline circumferences measured with an infrared scanner (LymphaTech®) at 6, 12 and 24 months after treatment onset
- Changes of the volume of the affected limbs compared to baseline circumferences measured with an infrared scanner (LymphaTech®) at 6, 12 and 24 months after treatment onset
- Changes in the frequency of acute attacks compared to pre-treatment evaluated at 6, 12 and 24 months after treatment onset

- Changes in the duration of acute attacks compared to pre-treatment evaluated at 6, 12 and 24 months after treatment onset
- Absence of acute attacks evaluated at 6, 12 and 24 months after treatment onset
- Changes of the hygiene level compared to pre-treatment assessed at 6, 12 and 24 months
- Changes of the quality of life (QoL) compared to pre-treatment assessed at 12 and 24 months after treatment onset
- Changes in levels of angiogenic, lymphangiogenic, pro-fibrotic or pro-inflammatory biomarkers (such as VEGF, CECAM-a, MMPS) in blood and/or urine compared to baseline values as a measure for prognostic effects assessed 6, 12 and 24 months after treatment onset

4.5. Overall timeline of the study

Enrolment: 6 - 9 months

Treatment: 2 - 3 months

Follow-ups will take place every two months after treatment onset until the 24 months follow-up (see chapter 5 "Schedule of activities").

Estimated date of first participant enrolled: Quarter 01/2018

Estimated date of last participant enrolled: Quarter 03/2018

Estimated date of last participant completed: Quarter 04/2020

Total duration of study: 33 months (9 months enrolment period + 6 weeks treatment period + 24 months follow-up after treatment start)

Expected duration of a subjects participation: ~ 26 months

4.6. Recruitment

4.6.1. **Recruitment procedure**

Recruitment will be carried out directly in the villages. Before start of recruitment the research team will visit the village elders to explain the planned study in detail. At the next visit all villagers will be invited to come to a public meeting where the study will again be explained in detail. After this meeting all interested volunteers, who are between 18 and 65 years old and signed the Informed Consent Form for screening, will be invited for the first screening which will be carried out by trained members of the research team under the supervision of Investigators. A medical doctor will be present to do the physical examination and to take the medical history of the volunteers. The blood sampling will be done by trained and qualified research personnel.

The screening will be carried out directly in the villages. The research team will use rooms provided by the village or will bring tents that guarantee the privacy of the volunteers during examination. In communities where CHPs premises are present examinations will be done in the CHPs premises.

All volunteers who proved to be eligible for the trial during the screening visit will be invited to come to the enrolment which will also be carried out directly in the village. During enrolment lymphedema specific examinations will be carried out. If the participant is still eligible for the trial after enrolment he/she will be randomized.

Screen failures will be encouraged to take part in the regular MDA ("standard of care") and to carry out intensive hygiene. In case volunteers are excluded before study start because of an illness that has to be treated they will be referred to the next hospital.

4.7. Study Population and Eligibility Criteria

This trial can fulfill its objectives only if appropriate participants are enrolled. The following eligibility criteria are designed to select subjects for whom protocol treatment is considered appropriate. All relevant medical and non-medical conditions will be taken into consideration when deciding whether this protocol is suitable for a particular participant.

Only patients with PodoLE will be included in this trial. Children < 14 years and persons not capable of giving adequately informed consent will not be included.

4.7.1. Gender Distribution

No gender ratio has been stipulated in this trial as the results of preclinical and clinical studies or medical literature did not indicate any difference in the effect of the trial treatment in terms of efficacy and safety.

4.7.2. Inclusion criteria

Patients eligible for the trial must comply with all of the following at **randomization**:

1. Lymphedema of at least one leg Grade 2-4 measured on a 5-point scale by F Tekola, Z Ayele, D HaileMariam, C Fuller and G Davey [1]
2. Age ≥ 18 years and ≤ 65 years
3. Men or non-pregnant women. If women of childbearing-potential, they must use an approved, effective method of contraception (including abstinence) before, during and for at least 2 weeks after the completion of the active intervention with doxycycline or placebo
4. Negative pregnancy test
5. Body weight ≥ 40 kg
6. Resident in podoconiosis endemic area for ≥ 2 years
7. Able and willing to give informed consent to participate in the trial
8. Ability to use established standardized methods of hygiene and effectively applying it prior to the initiation of the drug treatment
9. Negative test for lymphatic filariasis (LF)

4.7.3. Exclusion criteria

Patients are ineligible to participate in the trial, if they have any of the following:

1. No lymphedema, stage 1 or stage 5 lymphedema due to podoconiosis
2. Lymphedema due to LF
3. Age < 18 years or > 65 years
4. Body weight < 40 kg
5. Pregnant or breastfeeding women
6. Women of childbearing potential not using an agreed method of contraception (including abstinence; oral contraceptives are not allowed because of interaction with trial drugs)
7. Clinical or biologic evidence of hepatic or renal dysfunction or disease of the central nervous system (CNS)
8. Evidence of severe comorbidities except for features of filarial disease
9. Alcohol or drug abuse
10. History of adverse reactions to doxycycline or other tetracyclines
11. Any significant condition (including medical and psychological/ psychiatric disorder) which in the opinion of the study investigator might interfere with the conduct of the study
12. History of photosensitivity reactions after taking drugs.

13. Concomitant medication with antacids containing aluminium, magnesium or sucralfate and not able to discontinue
14. Concomitant medication with other antibiotics than doxycycline and not able to discontinue
15. Concomitant medication with diuretics or sulfonylurea
16. Concomitant medication with coumarin

Laboratory values which will lead to exclusion

1. Haemoglobin < 8 gm/dL
2. Neutrophil count <2 000/mm^3
3. Platelet count <100 000/mm^3
4. Creatinine > 2 times upper limit of normal
5. AST (GOT) > 2 times upper limit of normal
6. ALT (GPT) > 2 times upper limit of normal
7. y-GT > 2 times upper limit of normal
8. Positive urine pregnancy test
9. Positive wb123 or TBF or qPCR for *W. bancrofti*

4.7.4. Justification for exclusion of women and children

4.7.4.1 Pregnant Women

Pregnant and/or breastfeeding women will be excluded because doxycycline is contraindicated in pregnancy and during breastfeeding. However, non-pregnant women of childbearing-potential using an approved, effective method of contraception (including abstinence, excluding oral contraceptives because of interaction with trial drugs) before, during and for at least 2 weeks after the completion of the active intervention with doxycycline or placebo are eligible for inclusion. Since investigation of pregnancy status and use of contraception is a culturally sensitive issue, the approach to be used and the appropriateness of contraception will be defined by the local IRBs.

4.7.4.2 Children

Those less than 18 years of age will not be included .

5. TRIAL PROCEDURES

5.1. Clinical Evaluation

5.1.1. Medical history and physical examination

A complete medical history and physical examination will be performed as part of the baseline evaluation. Subsequent clinical evaluations will focus on the assessment of new symptoms, signs or untoward medical events. Vital signs, including blood pressure, pulse rate, and body temperature will be measured as part of all physical examinations, according to standard nursing practice.

5.1.2. Staging of lymphedema

Staging of PodoLE will be done using the 5 stage classification described by Tekola *et al.* [1]. The field worker is expected to look at *and* examine the right and left leg of each patient in turn, and give a score to each leg separately.

- 'Swelling' here means a general increase in size of part of the foot or leg.

- ‘Reversible swelling’ here means a swelling that is not present when the patient first gets up in the morning and becomes more marked as the day advances.
- ‘Persistent swelling’ here means a swelling that is present all the time.
- ‘Knob or bump’ here means a discrete, hard lump that can be seen *or* felt to protrude from the rest of the foot or leg.
- ‘Ankle’ here means the level of the two ankle bones when the patient is standing.
- ‘Knee’ here means the level of the top of the knee cap when the patient is standing.

In addition to the numerical stage, the field worker should measure the greatest below-knee circumference and record the presence (M+) or absence (M−) of mossy changes. For example, if a patient’s right leg has irreversible below-knee swelling, nodules below the ankle, mossy changes around the heel and a circumference of 48cm, the staging should be recorded as Stage 2, M+, 48.

Stage 1. Swelling reversible overnight

The swelling is not present when the patient first gets up in the morning. The swelling is not present when the patient first gets up in the morning. Changes such as hyperpigmentation and nail dystrophy are unusual, but may be seen. The swelling is usually confined beneath the ankle.

Stage 2. Below-knee swelling that is not completely reversible overnight; if present, knobs/bumps are below the ankle ONLY

Persistent swelling that does not reach above the knee. If present, knobs or bumps do not extend beyond the ankle.

The ‘knobs or bumps’ may take the form of dermal nodules, ridges or bands. Tourniquet-like effects may be observed at this stage or any subsequent stage, depending on the position of dermal ridges and nodules in relation to joints.

Mossy changes may be apparent, but their presence depends on a range of factors including the use of plastic footwear. Interdigital maceration and hyperpigmentation are often present at this stage, and nail dystrophy almost always present.

Stage 3. Below-knee swelling that is not completely reversible overnight; knobs/bumps present above the ankle

Persistent swelling that does not reach above the knee. Knobs or bumps can be seen or felt above the ankle as well as below. Dermal nodules, ridges or bands are seen or felt above the ankle. Tourniquet-like effects are frequently observed at this stage. Any of the other changes mentioned for Stage 2 may also be present.

Stage 4. Above-knee swelling that is not completely reversible overnight; knobs/bumps present at any location

Persistent swelling that is present above the knee. Knobs or bumps can be seen or felt at any place on the foot or leg.

Any of the other changes mentioned for Stage 2 may also be present. In addition, signs of lymphectasia may be apparent, particularly on the thigh.

Stage 5. Joint fixation; swelling at any place in the foot or leg

The ankle or interphalangeal joints becomes fixed and difficult to flex or dorsiflex. This may be accompanied by adhesion and fusion of the toe web spaces, making the toes appear short or indistinct. Sensation is preserved. X-rays show tuft resorption and loss of bone density.

A more detailed and technical description of the changes that *may* be present at each stage is given, though the definitions for each stage remain the same. The stages represent severity of disease, and do not necessarily represent the disease process: it is possible, for example, for an individual to have stage 5 disease but never to have had above-knee swelling. The following terms are used in the descriptions:

- Dermal nodules: elevated, non-translucent lesions >0.5cm diameter, with width approximately equal to length.
- Dermal ridges: elevated lesions >0.5cm width, with length greater than width
- Dermal bands: palpable, but non-elevated ridges
- Mossy changes: round or fusiform, *either* fluid filled (and hence translucent) lesions, *or* papillomatous hyperkeratotic horny lesions giving the skin surface a rough velvet-like appearance.

5.1.3. **Assessment of acute dermatolymphangioadenitis (ADLA)**

A questionnaire regarding the occurrence of ADLA episodes will be carried out at every contact.

5.1.4. **Circumference measurement of legs using a tape measure**

Leg circumferences will be measured using a tape measure. Circumference measurements will be made at 10 cm posterior to the tip of the large toe and 12, 20, and 30 cm from the sole of the foot, at least twice as described elsewhere. Averages of the 4 measurements will be determined before treatment and at follow-up.

5.1.5. **Circumference measurement of legs using an infrared scanner (LymphaTech®)**

A portable infrared scanner has recently been developed (LymphaTech scanner) that can quickly and accurately measure limb circumferences and volumes. This scanner will be used at baseline, 6, 12 and 24 months to measure the circumferences of both legs at 12, 20 and 30 cm. Measurements will be done twice for each leg.

5.1.6. **Assessment of the leg volume using an infrared scanner (LymphaTech®)**

A portable infrared scanner has recently been developed (LymphaTech scanner) that can quickly and accurately measure limb circumferences and volumes. This scanner will be used at baseline, 6, 12 and 24 months to measure the volume of both legs below 32cm. Measurements will be done twice for each leg.

5.1.7. **Training for care and hygiene of affected legs and arms**

All patients will be initiated to a programme of cleaning of the affected limb based on the principles outlined in the booklet "New Hope" for persons with lymphedema [4]. The regimens will be explained to all participants who will be trained to use established standardized methods of hygiene and be effectively applying it prior to the initiation of the drug treatment. Each patient will receive soap, towels and plastic bowls for washing the limbs and a diary for recording ADLA attacks. Supplies will be replenished during the follow-up visits, if necessary.

A generic SOP with handouts that describes methods and the training schedule will be utilized so that similar methods are employed across all sites. In addition, patients will be retrained at 4, 6, 12, 18 and 24 months. This will be standardized by the investigators and will include the following:

1. Cleaning of the affected limb daily with soap and water
2. Keeping the affected limb dry
3. Clipping the nails
4. Appropriate antibiotics for ADLA episodes

5. Applying antifungal ointment to webs of the toes, nails and sides of the feet every night
6. Elevation of the affected extremity
7. Limb exercises as instructed
8. Encouraging and monitoring the use of appropriate footwear

5.1.8. **Ultrasound**

Ultrasonography of the legs to assess lymphedema will be performed as described by Mand et al. [3]. Briefly, measurements will be taken in both legs over the lateral and medial malleoli of each ankle. A generic SOP that will enable the examiner to get reliable and reproducible results and will make comparisons possible between different examiners, different time points and the different trial sites, will be used so that the same method is employed across all sites.

5.1.9. **Clinical photographs**

Digital clinical photographs of the affected and normal limb will be taken baseline and at the follow-up time points at 6, 12 and 24 months. The distance and lighting will be standardized for each site by the investigator and all efforts to ensure comparability will be taken at each site. The photographs will be stored as digital images and made available for analysis.

5.1.10. **Quality of life (QOL) assessment**

QOL assessments will be performed at baseline, 12 and 24 months using the WHOQOL-BREF [14]

5.2. Laboratory Evaluation

5.2.1. **Blood**

Blood samples with volumes between 5 – 25 ml will be taken during screening (25 ml), before treatment no. 22 (5 ml) and after treatment no. 42 (5 ml) as well as 6, 12 and 24 months after treatment onset (25ml, respectively) to do the assessments described below as well as in chapter 10.3.

5.2.1.1 **Hematology and biochemistry**

At baseline venipuncture will be performed for assessing the full bloodcell count, transaminases, creatinine and bilirubin. Transaminases will also be checked before treatment no. 22 and on the last day of treatment (after treatment no. 42).

5.2.1.2 **Biomarkers in blood**

Angiogenic, lymphangiogenic, pro-fibrotic or pro-inflammatory biomarkers such as VEGF, CECAM-a, MMPS are important in lymphangiogenesis and lymphatic filarial infections. Biomarkers will be assessed at baseline, at treatment end as well as 6, 12 and 24 months after treatment start.

5.2.2. **Urine**

5.2.2.1 **Urine dipstick**

Urine samples will be collected in clean cups labelled with the barcodes of each study participant for routine urine examination using a dipstick method.

5.2.2.2 **Urine pregnancy tests**

Pregnancy tests will be carried out using urine during screening, at baseline, prior to the first treatment and after 14, 28 and 42 days of treatment as well as at the 2, 6, 12 and 24 months' follow-ups.

5.2.2.3 **Biomarkers in urine**

Biomarkers and miRNAs will be tested in urine at baseline, at the end of treatment, as well as 6, 12 and 24 months after treatment onset.

5.2.3. **Saliva**

Saliva samples (2 ml) will be taken at baseline and at 6, 12 and 24 months. The saliva samples will be used to look at different biomarkers to develop new diagnostic tools for podoconiosis without having to draw blood.

5.3. Schedule of Events

The study consists of the following visits (see also section 5 " Schedule of activities"):

Visit 1: Screening Visit

Visit 2: Baseline Visit (max. 28 days after screening)

Visit 3: Treatment

Treatment start (Day 1) - Daily observed treatment with 42 doses of Doxycycline –

Treatment end (Day 43 + max. 7 days)

Visit 4: 2 months follow-up (Day 61 ± 7 days)

Visit 5: 4 months follow-up (4 month, Day 121 ± 10 days)

Visit 6: 6 months follow-up (6 month, Day 182 ± 21 days)

Visit 7: 8 months follow-up (8 month, Day 243 ± 14 days)

Visit 8: 10 months follow-up (10 month, Day 303 ± 14 days)

Visit 9: 12 months follow-up (12 month, Day 364 ± 21 days)

Visit 10: 14 months follow-up (14 month, Day 425 ± 21 days)

Visit 11: 16 months follow-up (16 month, Day 485 ± 21 days)

Visit 12: 18 months follow-up (18 month, Day 546 ± 21 days)

Visit 13: 20 months follow-up (20 month, Day 607 ± 21 days)

Visit 14: 22 months follow-up (22 month, Day 667 ± 21 days)

Visit 15: 24 months follow-up (24 month, Day 728 -28/+48 days)

5.3.1. **Screening (Visit 1)**

Members of the research team will identify potential subjects. The following assessments/ procedures will be performed during screening:

- Informed consent for screening, treatment and biobanking
- Collection of demographic data
- Lymphedema staging
- History of lymphedema
- History of acute dermatolymphangioadenitis (ADLA)
- Medical history
- Concomitant medication

- History of trial with relevant medication
- Physical examination
- Vital signs
- Laboratory assessment (25 ml of blood): Full blood cell count, Liver Function Tests (AST/ALT/γ-GT), Serum Creatinine, biomarkers
- Urine sampling (max. 25 ml)
- Urine pregnancy test (for women of childbearing potential). In addition, women of childbearing potential will be counseled on avoiding pregnancy during the treatment period in a culturally appropriate manner.
- Saliva sampling (2 ml)
- Assessment of Inclusion/Exclusion criteria

5.3.2. **Enrolment (Visit 2)**

Baseline assessments will include:

- Concomitant medication
- Physical examination:
- Vital signs
- ADLA questionnaire
- Lymphedema staging
- Circumference of lymphedema - Tape measurement
- Circumference of lymphedema – Infrared scanner (LymphaTech®)
- Volume of lymphedema - Infrared scanner (LymphaTech®)
- Clinical photographs of the affected and normal limbs
- Hygiene status
- Lymphedema management training
- Ultrasound assessment of skin thickness at both ankles
- Quality of life (QOL) questionnaire
- Urine pregnancy test (for women of childbearing potential) In addition, women of childbearing potential will be counseled on avoiding pregnancy during the treatment period in a culturally appropriate manner.
- Randomization

5.3.3. **Treatment (Visit 3, day 1 – day 43 + 7 days range)**

5.3.3.1 **Treatment day 1**

- Urine pregnancy test if not done during visit 2 on the same day or one day before
- Verification and documentation any concomitant medication
- Participants will receive the first dose of either doxycycline or placebo under supervision of the trial clinicians at the study sites.

5.3.3.2 **Treatment days 2 – 42 (+ 7 days range)**

- Subsequent doses of the trial drugs will be administered under supervision of the research team. Participants will be expected to come every day to the meeting point in their village.
- The following assessments will be done during each day of treatment:
    - Documentation of participant’s presence

    - Administration of ADLA questionnaire
    - Assessment of AE/SAE
    - Verification and documentation any concomitant medication
    - Direct observed treatment
- The following additional assessments will be done on predefined days during the treatment period:
    - After 14 days:
        - Urine pregnancy test (for women of childbearing potential)
    - Before treatment no. 22:
        - Laboratory assessment (5 ml of blood): serum transaminases
    - After 28 days:
        - Urine pregnancy test (for women of childbearing potential)
    - After 42 days:
        - Urine pregnancy test (for women of childbearing potential)
    - After treatment no. 42:
        - Laboratory assessment (5 ml of blood): serum transaminases, biomarkers
        - Urine sampling

5.3.3.3 **End of treatment (day 43 + 7 days range)**

One day after the last dose of trial drugs, the following assessments will be done:

- Documentation of participants presence
- Administration of ADLA questionnaire
- Assessment of AE/SAE
- Verification and documentation of concomitant medication

5.3.4. **2 months follow-up**

- Documentation of participants presence
- Administration of ADLA questionnaire
- Assessment of AE/SAE
- Verification and documentation of concomitant medication
- Urine pregnancy test (for women of childbearing potential)

5.3.5. **4 months’ follow-up**

- Documentation of participants’ presence
- Administration of ADLA questionnaire
- Assessment of AE/SAE
- Verification and documentation of concomitant medication
- Verification and documentation of hygiene status
- Lymphedema management training

5.3.6. **6 months’ follow-up**

- Documentation of participants’ presence
- Verification and documentation of concomitant medication
- Assessment of vital signs
- Administration of ADLA questionnaire
- Lymphedema staging

- Circumference of lymphedema - Tape measurement
- Circumference of lymphedema – Infrared scanner (LymphaTech®)
- Volume of lymphedema - Infrared scanner (LymphaTech®)
- Clinical photographs of the affected and normal limbs
- Verification and documentation of hygiene status
- Lymphedema management training
- Ultrasound assessment of skin thickness at both ankles
- Laboratory assessment (25 ml of blood): FTS, Microfilariae count (Sedgewick, in FTS positive participants only), biomarkers, T-cell activation markers
- Urine sampling
- Urine pregnancy test (for women of childbearing potential)
- Saliva sampling

5.3.7. **Follow-ups at 8, 10, 14, 16, 20 and 22 months after the first day of treatment**

The following assessments will be done 8, 10, 14, 16, 20 and 22 months after treatment onset:

- Documentation of participants' presence
- Verification and documentation of concomitant medication
- Administration of ADLA questionnaire

5.3.8. **18 months' follow-up**

- Documentation of participants' presence
- Concomitant medication
- ADLA questionnaire
- Verification and documentation of hygiene status
- Lymphedema management training

5.3.9. **12 and 24 months' follow-ups**

- Documentation of participants' presence
- Concomitant medication
- Assessment of vital signs
- Administration of ADLA questionnaire
- Lymphedema staging
- Circumference of lymphedema - Tape measurement
- Circumference of lymphedema – Infrared scanner (LymphaTech®)
- Volume of lymphedema - Infrared scanner (LymphaTech®)
- Clinical photographs of the affected and normal limbs
- Verification and documentation of hygiene status
- Lymphedema management training
- Ultrasound assessment of skin thickness at both ankles
- Quality of life (QOL) questionnaire
- Laboratory assessment (25 ml of blood): biomarkers
- Urine sampling
- Urine pregnancy test (for women of childbearing potential)
- Saliva sampling

6. INVESTIGATIONAL MEDICINAL PRODUCT (IMP)

6.1. General description of the study drug doxycycline

Doxycycline is an already marketed product for the antibiotic treatment of several infectious diseases. The treatment of lymphedema due to lymphatic filariasis is an off label treatment.

6.2. Specification of IMP

Trade name	Remycin®
Name of manufacturer	Remedica, Cyprus
Substance name (if applicable, give substance code)	Doxycycline
Name and dose of active ingredient per unit	Doxycycline hyclate 100mg
Pharmaceutical form	Tablets, film-coated
Mode of administration	oral
Storage conditions	between 20 - 25°C

The matching placebo has been manufactured, blistered and labeled by Piramal Healthcare, Morpeth, UK..

6.3. Dosage and Route of Administration

Both doxycycline and placebo will be administered *ad persona* by the trial clinician directly in the villages under supervision (directly observed treatment, DOT) for 6 weeks.

Doxycycline 200mg (2 tablets of 100mg) will be administered orally once daily for 6 weeks. The dosage will be reduced to 1 tablet o 100mg in participants with a body weight ≥ 40kg and < 50kg.

Placebo matching doxycycline 100 mg (2 tablets) will be administered orally once daily for 6 weeks.

The first dose of doxycycline or placebo will be given after all the investigations have been completed, informed consent has been obtained and the patient has been initiated into the programme of basic hygiene. Patients will be encouraged to eat before swallowing the tablets whole with a glass of water. Vomited doses within 15 minutes after intake will be replaced.

6.4. Findings from clinical studies

Doxycycline is a well-known and safe drug used for the treatment of several infectious diseases.

Doxycycline with a dosage of 200 mg/d or 100mg/d for 4- 6 weeks was already used in several clinical trials in lymphatic filariasis or onchocerciasis [2, 3, 9, 15-18].

6.5. Summary of known and potential risks of doxycycline

6.5.1. Side effects of doxycycline

: Common cold (22%), influenza symptoms (11%)

Nausea (up to 13.4%)

Headache (up to 26%)

: Increased blood lactate dehydrogenase (2%), increased blood glucose (1%)

Nasopharyngitis (5%), sore throat (5%), sinus congestion (5%), coughing (4%), sinus headache (up to 4%), sinusitis (3%), bronchitis (3%), nasal congestion (2%), pharyngolaryngeal pain (1%)

infection (2%), fungal infection (2%), influenza (2%)

Menstrual cramp (4%), bacterial vaginitis (3.3%), vulvovaginal mycotic infection (2%)

Increased aspartate aminotransferase (2%)

Injury (5%), pain (up to 4%), back pain (up to 3%), back ache (2%)

Rash (4%)

Vomiting (8.1%), toothache (7%), tooth disorder (6%), dyspepsia (6%), diarrhea (up to 6%), periodontal abscess (4%), acid indigestion (4%), upper abdominal pain (2%), abdominal distention (1%), abdominal pain (1%), stomach discomfort (1%), dry mouth (1%)

Joint pain (6%), muscle pain (1%)

Cardiovascular: Hypertension (3%), increased blood pressure (2%)

Psychiatric: Anxiety (2%)

Gum pain

Rare Adult tooth staining (at least 1 case)

Frequency not reported:

Hemolytic anemia, thrombocytopenia, neutropenia, eosinophilia,Hypoglycemia, anorexia, possible overgrowth of nonsusceptible organisms (superinfection), dose-related rise in BUN, vaginal itch, vaginal candidiasis, acute hepatocellular injury, cholestatic reactions, hepatotoxicity, microscopic brown-black discoloration of the thyroid gland, nail discoloration, phototoxicity, photoallergic reaction, photo-onycholysis, photosensitivity, maculopapular and erythematous rashes, erythema multiforme, Stevens-Johnson syndrome, toxic epidermal necrolysis, exfoliative dermatitis, hyperpigmentation, clostridium difficile associated diarrhea, esophageal irritation, ulceration, epigastric burning, black hairy tongue, esophagitis and esophageal ulcerations (most took medication immediately before going to bed), sinus headache, dizziness, drowsiness, amnesia, paresthesias of body areas exposed to sunlight, phrenic nerve paralysis after sclerotherapy, benign intracranial hypertension resulting in significant loss of vision, diplopia, papilledema, loss of vision (associated with doxycycline-induced benign intracranial hypertension) Hypersensitivity: Hypersensitivity reactions (including urticaria, angioneurotic edema, anaphylaxis, anaphylactoid purpura, serum sickness, pericarditis, exacerbation of systemic lupus erythematosus, drug rash with eosinophilia and systemic symptoms (DRESS), autoimmune syndromes Pseudotumor cerebri (benign intracranial hypertension), headache

Photosensitivity

Photosensitivity manifested by an exaggerated sunburn reaction has been observed in some individuals taking tetracyclines. Patients apt to be exposed to direct sunlight or ultraviolet light will be advised that this reaction can occur with tetracycline drugs. Treatment will be immediately stopped at the first evidence of skin erythema.

Growth and Development

All tetracyclines form a stable calcium complex in any bone-forming tissue. A decrease in fibula growth rate has been observed in prematures given oral tetracycline in doses of 25 mg/kg every six hours. This reaction

was shown to be reversible when the drug was discontinued. Results of animal studies indicate that tetracyclines cross the placenta, are found in fetal tissues, and can have toxic effects on the developing fetus (often related to retardation of skeletal development). Evidence of embryotoxicity also has been noted in animals treated early in pregnancy. If any tetracycline is used during pregnancy or if the patient becomes pregnant while taking these drugs, the patient should be apprised of the potential hazard to the fetus. Therefore, women taking part in the clinical trial will be informed in detail about the risk associated with a pregnancy during drug intake. Pregnancy tests will be carried out every 2 weeks and if a positive pregnancy test occurs against all precautions, treatment will be stopped immediately.

Clostridium difficile –associated diarrhea

Clostridium difficile-associated diarrhea (CDAD) has been reported with the use of nearly all systemic antibacterial agents, including Moxifloxacin, with severity ranging from mild diarrhea to fatal colitis. Therefore, participants in this trial with diarrhea will be monitored especially for CDAD and in case of suspected or confirmed CDAD treatment will be immediately stopped and the patient will be treated for CDAD as appropriate (fluid and electrolyte management, protein supplementation, antibacterial treatment of C. difficile, surgical evaluation if clinically indicated) until recovery. Since CDAD has been reported to occur over two months after the administration of antibacterial agents, patients will be strongly encouraged to report any severe diarrhea to the research team also after completion of the treatment.

Superinfection

As with other antibiotic preparations, use of this drug may result in overgrowth of non-susceptible organisms, including fungi. If superinfection occurs, treatment will be immediately stopped and appropriate therapy instituted until recovery.

Benign Intracranial Hypertension (pseudotumor cerebri)

Bulging fontanels in infants and benign intracranial hypertension in adults have been reported in individuals receiving tetracyclines. These conditions disappeared when the drug was discontinued. Therefore, treatment of patients with a suspected benign intracranial hypertension (e.g. headache associated with gradual visual field defects, nausea, vomiting, drowsiness) will be stopped immediately and appropriate therapy will be given until full recovery.

6.5.2. General precautions and warnings

Pregnancy and breastfeeding:

To avoid any complications, pregnant or breastfeeding women will be excluded from the clinical trial. Pregnancy tests will be carried out during the screening and right before the first treatment. Additionally, pregnancy tests will be repeated after every two weeks of treatment. In case of pregnancy in any group, treatment will be stopped immediately. All women will be informed in detail about the risks of getting pregnant during the informed consent procedure and their obligation and responsibility to use effective contraceptive methods excluding daily hormonal contraception as the trial drugs can reduce their efficacy.

6.6. Blistering and Labelling of the IMP

Blistering and labelling of the trial drugs will be done by Piramal Healthcare, Morpeth, United Kingdom. All IMPs will be blistered and labelled for the conduct of clinical trials only.

6.7. Transport of IMP

The trial drugs will be delivered to the trial sites by plane and car at appropriate conditions (temperature not above 25°C).

6.8. Handling of IMP at the Site and Drug Accountability

All study drug will be kept in a secure cabinet or a room with access restricted to the responsible study personnel. Medication must be stored at appropriate conditions (temperature not above 25°C). The storage and handling of all study medication will be supervised by the study pharmacist.

The study pharmacist will be responsible for maintaining accurate records of receipt of all study medication as well as of all medication dispensed to and used by each patient.

At the completion of the study, all unused medication will be destroyed once drug accountability is completed.

6.9. Strategies to improve adherence and compliance

Both doxycycline and placebo will be administered under supervision throughout the treatment period (directly observed treatment). Ideally, patients will be required to come every day to the meeting point in their village to take their drugs under supervision. In case a patient is not present for the DOT, the drugs will be handed out to a village health worker or a family member and the intake by the patient will be checked at the following day when the patient will have to give back the empty package and the village health worker or family member will be asked to witness the intake. In some cases, patients have to travel for a few days. When they proved their compliance before, the drugs for the travel days will be handed out to the patient and compliance will be reviewed by getting back the empty packages when the patient comes back from travel. In this case the patient will be asked to immediately report any adverse reactions or problems with the trial drugs by mobile phone to the trial clinician.

All patients will be counselled at the time of the initial dose and during the treatment period. Key messages will include:

- The importance of following study guidelines for adherence
- Instructions about taking study pills whole, and what to do in the event of a missed dose.
- Reinforcement that study pills may be doxycycline or placebo
- Emphasize that all participants are expected to benefit from the hygiene intervention.
- Importance of contacting the research team if experiencing problems possibly related to study product
- Patients will be questioned about problems with the drugs and motivated to complete treatment as planned
- In addition, patients will be visited once every two months in the follow-up period to record the occurrence of ADLA attacks and reinforce the hygiene routine.

6.10. Unblinding

The patient, the investigators and all other site staff will be kept blinded throughout the study. All assessments of the patient will be made without knowledge of the treatment and every effort must be made to maintain the blinding. Unblinding will only occur in the case of medical emergencies or pregnancy and only if knowledge about the administered study drug is expected to improve the patient’s treatment.

6.11. Prior and Concomitant Therapy/Medication

6.11.1. Previous therapy / medication of trial specific illness

All previous treatments for managing LE (e.g. Mass drug administration, previous rounds of IVM and/or ALB, last intake of IVM, last intake of ALB) and applied medications will be documented in the CRF according to the memory of the patient.

6.11.2. Previous therapy / medication with doxycycline

All previous treatments and medications with doxycycline during the last year before starting the trial will be documented in the CRF according to the memory of the patient.

6.11.3. Prohibited therapy / Concomitant medication

Diet: There is no dietary restriction and participants will be encouraged to eat within one hour prior to drug intake.

The following therapies / medications are not allowed to be applied during the treatment because of interaction with the trial dugs:

- antacids containing aluminum, magnesium or sucralfate
- other antibiotics than doxycycline
- sulfonylurea, coumarin (doxycycline increases the levels of the coumarin and sulfonylurea which might lead to an adverse event)

If it is important to start treatment with one of the above mentioned prohibited therapies, treatment with the trial drugs will be stopped to prevent the patient from the interactions.

Female patients will be informed in detail that oral contraceptives might not work due to interactions with doxycycline. They will only be included in the clinical trial if they agree to use other agreed methods of contraception.

During the whole study the use of diuretics is not recommended, as they will change the outcome of the measurement. Medication will lead to exclusion of patient data from the analysis.

During treatment and the 24 months' follow-up period, short courses of antibiotics are permitted for the treatment of ADLA attacks and infections such as UTI or URTI. Intake of all drugs other than the study drugs will be documented on the treatment registers and every two months in the case report forms.

7. ADVERSE EVENTS

7.1. Documenting, Recording and Reporting Adverse Events

At each contact with the subject, information regarding adverse events will be elicited by appropriate questioning and examinations and will be:

- immediately documented in the subject's medical record/source document,
- recorded on the Adverse Event Case Report Form (AE CRF) and
- reported as outlined

7.2. Definitions

7.2.1. Adverse Event (AE)

An adverse event is any untoward or unfavorable medical occurrence in a human subject, including any abnormal sign (e.g., abnormal physical exam or laboratory finding), symptom, or disease, temporally associated with the subject's participation in the research, whether or not considered related to the research. An unexpected AE is an experience not reported in the current Investigators Brochure or elsewhere.

7.2.2. **Unexpected Adverse Event (UAE)**

An AE is unexpected if it is not listed in the Investigator's Brochure (IB) or Package Insert (PI) (for marketed products) or is not listed at the specificity or severity that has been observed. It is the responsibility of the IND Sponsor to make this determination.

7.2.3. **Adverse (Drug) Reaction (AR)**

An adverse reaction is an adverse event that is caused by an investigational agent (drug or biologic).

7.2.4. **Suspected Adverse (Drug) Reaction (SAR)**

An adverse event for which there is a reasonable possibility that the investigational agent caused the adverse event. 'Reasonable possibility' means that there is evidence to suggest a causal relationship between the drug and the adverse event. A suspected adverse reaction implies a lesser degree of certainty about causality than adverse reaction which implies a high degree of certainty.

7.2.5. **Unexpected Adverse (Drug) Reaction (UAR)**

An adverse reaction, the nature or severity of which is not consistent with the applicable product information (e.g., Investigator's Brochure for an unapproved investigational product or package insert/summary of product characteristics for an approved product)

7.2.6. **Serious Adverse Event (SAE)**

A Serious Adverse Event is an AE that results in one or more of the following outcomes:

- death
- a life threatening (i.e., an immediate threat to life) event
- an inpatient hospitalization or prolongation of an existing hospitalization
- a persistent or significant incapacity or substantial disruption of the ability to conduct normal life functions
- a congenital anomaly/birth defect
- a medically important event (Medical and scientific judgment should be exercised in deciding whether expedited reporting is appropriate in other situations, such as important medical events that may not be immediately life threatening or result in death or hospitalization but they may jeopardize the subject or may require intervention to prevent one of the other outcomes listed above.)

7.2.7. **Serious Adverse (Drug) Reaction (SAR)**

This is defined as an adverse drug reaction that is serious and at least possibly related to the IMP (see SAE criteria above).

7.2.8. **Suspected Unexpected Serious Adverse (Drug) Reaction (SUSAR)**

A SUSAR is a Suspected Adverse Reaction that is both Serious and Unexpected. Any UAR that at any dose results in death, is life-threatening, requires inpatient hospitalization or prolongation of existing hospitalization, results in persistent or significant disability/incapacity or is a congenital anomaly/birth defect.

7.2.9. **Unanticipated Problem (UP)**

An Unanticipated Problem is any event, incident, experience, or outcome that is:

1. unexpected in terms of nature, severity, or frequency in relation to
   a. the research risks that are described in the IRB-approved research protocol and informed consent document; Investigator's Brochure or other study documents; and
   b. the characteristics of the subject population being studied; and
2. possibly, probably, or definitely related to participation in the research; and
3. places subjects or others at a greater risk of harm (including physical, psychological, economic, or social harm) than was previously known or recognized.

7.2.10. **Unanticipated Problem that is not an Adverse Event (UPnonAE)**

Unanticipated problem that is not an Adverse Event (UPnonAE): An unanticipated problem that does not fit the definition of an adverse event, but which may, in the opinion of the investigator, involve risk to the subject, affect others in the research study, or significantly impact the integrity of research data. Such events would be considered a non-serious UP. For example, we will report occurrences of breaches of confidentiality, accidental destruction of study records, or unaccounted-for study drug.

7.3. Criteria to be evaluated by the trial clinician

If a diagnosis is clinically evident (or subsequently determined), the diagnosis rather than the individual signs and symptoms or lab abnormalities will be recorded as the AE.

All AEs occurring from the time the informed consent is signed through the 4 months follow-up will be documented, recorded, and reported. The trial clinician will evaluate all AEs with respect to **Seriousness** (criteria listed above in section 12.2), **Severity** (intensity or grade), and **Causality** (relationship to study agent and relationship to research) according to the following guidelines.

7.3.1. **Assessment of Intensity**

Any adverse event has to be graded regarding its intensity:	
MILD (Grade 1)	Does not interfere with subject's usual activities or is transient, easily tolerated.
MODERATE (Grade 2)	Interferes to some extent with subject's usual activities (which patient is still able to perform).
SEVERE (Grade 3)	Interferes significantly with subject's usual activities (which patient is not able to perform)

7.3.2. **Assessment of Adverse Event Intensity for Doxycycline**

Adverse Event	Grade	Intensity
Stomach Pain	0<br>1<br>2<br>3	Absent<br>Pain is easily tolerated (able to eat)<br>Pain interferes with daily activities (unable to eat)<br>Pain that prevents daily activities (combined with vomiting and/or diarrhoea)
Loss of appetite	0<br>1	Absent<br>Accompanied with nausea

	2<br>3	Accompanied with nausea and vomiting or diarrhoea<br>Accompanied with nausea and loss of weight
Nausea	0<br>1<br>2<br>3	None<br>Nausea that is easily tolerated (able to eat)<br>Nausea that interferes with daily activity (unable to eat)<br>Nausea that prevents daily activity (combined with vomiting and/or diarrhoea)
Vomiting	0<br>1<br>2<br>3	None<br>Vomiting that is easily tolerated (able to eat)<br>Vomiting that interferes with daily activity (unable to eat)<br>Vomiting that prevents daily activity (combined with nausea and/or diarrhoea)
Diarrhea	0<br>1<br>2<br>3	None<br>Diarrhoea that is easily tolerated (up to 3 times per day)<br>Diarrhoea that interferes with daily activity (more than 3 times per day combined with nausea and/or vomiting and weakness)<br>Diarrhoea that prevents daily activity (combined with nausea, vomiting and/or acholic faeces)
Bloody diarrhea	0<br>1<br>2<br>3	None<br>Fresh or clotted in absence of haemorrhoids<br>Fresh or clotted blood combined with abdominal pain<br>Fresh and clotted blood combined with abdominal pain and fever
Headache	0<br>1<br>2<br>3	None<br>Headache that is easily tolerated<br>Headache that interferes with daily activity<br>Headache that prevents daily activity
Urticaria	0<br>1<br>2<br>3	None<br>Requiring no medication<br>Requiring oral and/or topical medication (including steroid) for < 24h<br>Requiring oral, topical and/or medication IV medication (including steroid) for > 24h
Rashes	0<br>1<br>2<br>3	Absent<br>Localized, itching, no blisters, lasting one day<br>Localized itching, with blisters lasting longer than one day<br>Generalized combined with fever
Phototoxicity	0<br>1<br>2<br>3	Absent<br>Erythema in sun exposed skin<br>Erythema in sun exposed skin and rise of skin temperature<br>Erythema in sun exposed skin, blisters and fever
Fever	0<br>1<br>2<br>3	≤ 37.5°C<br>>37.5°C-38°C<br>>38°C-39°C<br>>39°C
Anaphylactic reactions	0<br>1	None<br>headache accompanied by rash and itching

	2<br>3	in addition to 1. blood pressure failure and tachycardia, oedema<br>in addition to 2. bronchial spasms (expiratory stridor), apnoea or cardiac arrest →SAE

7.3.3. **Assessment of Seriousness**

Determination of the seriousness of the adverse event according to the definitions for a serious adverse event (SAE) given in section 12.2.6.

7.3.4. **Assessment of Causality**

Causality (likelihood that the event is related to the study agent) will be assessed considering the factors listed under the following categories:

Definitely Related

- reasonable temporal relationship
- follows a known response pattern
- clear evidence to suggest a causal relationship
- there is no alternative etiology

Probably Related

- reasonable temporal relationship
- follows a suspected response pattern (based on similar agents)
- no evidence of a more likely alternative etiology

Possibly Related

- reasonable temporal relationship
- little evidence for a more likely alternative etiology

Unlikely Related

- does not have a reasonable temporal relationship

OR

- good evidence for a more likely alternative etiology

Not Related

- does not have a temporal relationship

OR

- definitely due to an alternative etiology

7.4. Adverse Event Recording

Any AE relevant for the evaluation and analysis of the clinical trial has to be documented in the source data and in the CRF on the respective Adverse Event Report Form.
Documentation and evaluation of each AE occurring between:

- Visit 3 (first day of treatment) and
- Visit 5 (4 months follow-up)

7.5. Adverse Event Reporting

Line listings, frequency tables, and other summary AE data will be submitted to the Sponsor when needed for periodic safety assessments requested by the DSMC, review of annual reports, review of safety reports, and preparation of final study reports.

7.6. Serious Adverse Event Reporting

Any SAE (whether or not they are also UPs) ocurring after the subject has received the trial drug for the first time until the 2 months follow-up (Visit 4) will be documented and reported within 48 hours after investigator awareness of the event to the National Ethics Committee for Health Research on Humans (NECHRH) and the Division for Health Operations Research (DROS) of the MINSANTE informally via email containing at least the minimal criteria (the name of the clinical trial, an identifiable patient, an identifiable reporter, a reaction/event, a causality assessment).

Adress:

7.7. Reporting of Unanticipated Problems (UPs)

Unanticipated Problems (definition see section 7.2.9) that are also adverse events will be reported to the NECHRH and the DROS and sent by fax or e-mail attachment no later than 7 calendar days of site awareness of the event.

7.8. Follow-up of Adverse Events

Every AE or SAE will be treated according to clinical standards at the discretion of the investigator and followed up until it is resolved. Costs for treatment of AEs/SAEs will be covered by the research team.

7.9. Handling of emergency cases

The trial clinicians will visit the participants in their villages every day during the treatment period to closely supervise possible AEs or SAEs. Additionally, the phone numbers of the trial clinicians will be handed to the village health workers as well as to the participants. In case of an emergency between the visits, the clinicians have to be called and will come to the participants whenever needed. In case the participant has to go to a hospital for further examinations and treatment, the transport, accommodation and medical care will be paid by the research team.

7.10. Deaths

All fatal cases during the treatment period and the time of follow-ups will be accompanied by a formal autopsy report. Deaths occurring in the period between treatment and follow-ups will be evaluated in detail and, if reported immediately to the research team, also accompanied by a formal autopsy.

7.11. Pregnancies

Female participants will be screened for pregnancy before enrolment. Pregnant and/or breastfeeding women will be excluded because doxycycline is contraindicated in pregnancy and during breastfeeding. However, non-pregnant women of childbearing-potential using an approved, effective method of contraception before,

during and for at least 2 weeks after the completion of the active intervention with doxycycline or placebo are eligible for inclusion. As doxycycline is reducing the blood level of contraceptives it is recommended to use an additional method (barrier) to prevent a pregnancy during the treatment phase with doxycycline.

7.11.1. **Pregnancy Procedures**

All women of childbearing potential will be instructed to contact the investigator immediately if they suspect they might be pregnant during the study (for example, missed or late menses). If pregnancy is suspected while the patient is receiving experimental study treatment, this will be withheld immediately until pregnancy can be ruled out with certainty.

If a woman becomes pregnant during the treatment period, treatment with the IMP will be immediately stopped. She will be treated according to clinical routine at the discretion of the treating medical doctor supported by the investigator. The woman will remain in the trial as a participant (Intention-to-treat collective).

7.11.2. **Pregnancy Reporting**

To ensure the safety of female subjects, each pregnancy that becomes known to the investigator during the trial, must be reported as an event similar to an SAE. Therefore, the investigator will record and report pregnancy information on the appropriate pregnancy report form as an initial report and send it immediately (latest within 24h) to the Sponsor.
The pregnancy itself is not considered to be an AE or SAE but must be followed up until delivery or until pregnancy termination and the outcome of pregnancy should be notified to the Sponsor to determine the outcome of the pregnancy regarding maternal or newborn complications. The investigator will seek and provide this follow-up information after the planned date of delivery. For this purpose, the pregnancy report form will be used as follow-up report. Infants should be followed for a minimum of 6 months.

If the outcome of the pregnancy includes

- Spontaneous, therapeutic abortion or voluntary termination,
- stillbirth,
- neonatal death,
- presence of birth defects, or
- congenital anomaly (including that in an aborted fetus, stillbirth or neonatal death),

the investigator should report this outcome as an SAE.

8. STATISTICAL CONSIDERATIONS

8.1. Hypothesis

Doxycycline is superior to placebo for management of lymphedema in patients from podoconiosis-endemic areas.

8.2. Primary target variable

Lack of progression of LE (stage reduction or same stage as pre-treatment using the 5-point scale staging according to Tekola *et al.* [1]) examined 24 months after treatment onset.

8.3. Sample size estimations

Doxycycline 200mg/d will be tested for superiority to placebo at a two-sided α = 5%. The estimates for progression are based on the results from the previous study by Mand et al. [3] where a progression of 4.9% was seen in the DOX 200 group after 24 months whereas the placebo-treated patients had a progression of 55.6%. The strict control implementation of hygiene measures in this trial may result in a stronger impact of this intervention on both arms. This would result in a smaller margin to verify the added benefit of doxycycline and as a consequence increase the number of patients in the study. In order to account for this influence in the sample size calculations the progression in the placebo group was assumed to be 25% instead of 55%. A dropout rate of 30% was assumed.

8.4. Sample size calculation

Based on the assumption of a progression in the DOX 200 group of 5% and in the placebo group of 25%, there will be power of 90% to show superiority to placebo if 71 participants are included in each treatment group (+ 30% drop-out rate: N = 100/treatment).

8.5. Achievement of sample size

The clinical centers involved in the study were selected based on documentation for patient availability and availability of infrastructure and resources for the study.

8.6. Populations to be analyzed

Different analysis populations will be prepared before de-blinding (Safety population (SAF), Intention-to-treat population (ITT), Per-Protocol population (PP)). The criteria for the exclusion of patients from any of the analysis populations will be defined in the Statistical Analysis Plan (SAP) and will be finalized prior to database lock. The exclusion of participants from the analysis populations will then be decided during the blind data review before unblinding and will be described in detail in the blind data review report.

The SAF population will be used for the analysis of adverse events.

8.6.1. Intent-to treat (ITT) population

The ITT population will consist of all randomized patients in the groups to which they were randomly assigned and who took the study drugs at least once.

8.6.2. Per-Protocol (PP) population:

The PP population will consist of all patients who fulfil the protocol in terms of eligibility, compliance, interventions, and outcome assessment without any major protocol deviation.

8.6.3. Safety Population

The safety population will be defined as all patients who received any study drug

8.7. Statistical methodology

A detailed statistical analysis plan (SAP) will be written before final closure of the data file and before de-blinding of the research team. The Data Safety Monitoring Committee (DSMC) will have to agree on the data analysis plan before closure.

8.7.1. Analysis of baseline characteristics

Baseline characteristics, such as demographic and analytical data will be summarized for each intervention group using descriptive statistical methods.

8.7.2. Analysis of the primary endpoint

The frequencies of the participants with “progression” and their confidence intervals (95%) will be calculated and compared between the treatment groups using Fisher’s exact test.

8.7.3. Analysis of the secondary endpoints

Quantitative data will be summarized for each intervention group using the mean, the median, standard deviation, the range (minimum and maximum value), 25th - 75th percentiles and 95% confidence intervals. Qualitative data will be summarized for each intervention group using frequency counts, percentages and 95% confidence intervals. Additionally, box plots or other graphical methods may be used to present the data. The groups will be compared using appropriate statistical methods.

8.7.4. Safety Analysis

Adverse events (AE) will be assessed in the scope of the daily observed treatment (DOT) and described for each intervention group using frequency counts, percentages and 95% confidence intervals.

8.8. Interim Analysis

An interim analysis is planned at each study site when all patients have completed 12 months of follow-up. A decision at 12 months may be taken to terminate the study because of superiority of the doxycycline arm so that the placebo subjects can be treated. Superiority may be defined as non-progression of grade, reduced ADLA, etc. Details of the parameters to be used for the interim analysis will be defined prior to the closing of the database and incorporated in the SAP.

8.9. Protocol violations

Protocol violations are major deviations from the procedures outlined in this document like:

- non-compliance with investigational medicinal product (missing treatment days at the end of the treatment period (the treatment period should be prolonged for all days the patient was absent or did not get the trial drugs by decision of the trial clinician),
- intake of medications not allowed
- any non-adherence to the protocol that would have an impact to the subject’s rights, safety or welfare,
- absence from treatment for more than 3 consecutive treatment days (participants who are absent for more than 3 consecutive treatment days will be requested to finish the whole treatment).
- absence from treatment for more than 7 treatment days (treatment will be stopped if participants are absent for more than 7 treatment days, but the patients will be asked to come for the follow-ups).

After a subject has been enrolled, it is the investigator’s responsibility to make a reasonable effort to prevent and correct any protocol violations if necessary and to continue the subject’s participation in the trial, if possible. Protocol violations will be reported to the sponsor/sponsor delegated person during the course of the trial in the monitoring reports and reported to the Ethics Committees after completion of the trial. All protocol violations will be listed and the impact on the evaluation of the subjects concerned will be discussed prior to statistical analysis.

8.10. Handling of Drop-outs, Withdrawal, and Missing Data

8.10.1. Screening failures

Subjects dropping out of the trial prior to randomization will be listed as screening failures including the reason.

8.10.2. Drop-out/Withdrawal after randomization but before treatment start

Subjects dropping out of the trial after randomization but before start of treatment will be reported including the reason.

8.10.3. Drop-out/Withdrawal during or after treatment

Subjects dropping out of the trial after randomization during but also after treatment will be analysed using all available data (ITT analysis).

8.10.4. Missing treatment days

Subjects missing more than 3 consecutive treatment days will be analysed using all available data (ITT analysis) but will be excluded from the PP-analysis.

8.10.5. Replacement of Patients

- In case of subjects dropping out in the period between randomization and treatment start additional subjects will be included in the trial. The new participants will be randomized consecutively.
- In case of subjects dropping out during the first 3 days of treatment, additional subjects may be included in the trial. The new participants will be randomized consecutively. All available data from the participants who were treated at least once and later replaced will still be used for the ITT analysis.

8.11. Statistical report

The statistical evaluation and the statistical report will be performed, evaluated and signed by the responsible biometrician. All data in this report will be strictly confidential.

9. DATA SAFETY AND MONITORING COMMITTEE (DMSC)

A single central DSMC will be established with a charter that defines the roles and responsibilities and details of meeting frequency and communications. The DSMC thus established will, in addition to the Authorities/IRBs involved, have a chance to comment on the content of the protocol. The composition, role of the members and the reporting structure will be finalized and reported to the National Ethic Committee for Human Research in Yaoundé, the Institutional Review board of the Faculty of Health sciences, University of Buea, before start of recruitment.

10. DEFINITION OF END OF TRIAL

10.1. Regular end of trial

The regular end of trial is defined as Last Subject Last Visit, meaning, the trial ends when the last patient will be informed about the best performing treatment. At this visit re-treatment will be offered to patient. This re-treatment is not part of the clinical trial but will be performed by the trial clinicians.
After regular end or early termination of the trial, patients will be treated according to clinical routine at the discretion of the treating physician.

10.2. Termination of the trial for individual subjects

10.2.1. Termination by the participant

A patient may decide to withdraw from the study at any time and for any reason. The Investigator should attempt to determine the reason for the subject's decision. There will be no disadvantage for the participant as a result of a withdrawal. If a participant does not return for a scheduled visit, every effort should be made to contact the participant. The investigator should inquire about the reason for withdrawal and the participant should be followed-up regarding any unresolved adverse events, if possible. In any circumstance, every effort should be made to document the participants' outcome. Therefore all participants, even if the participant was withdrawn from the trial treatment, will be encouraged to come for the follow-up visits.

10.2.2. Termination by the investigator

The investigator may withdraw a patient for any of the following reasons:

- An individual subject's decision.
- Any clinical AE, laboratory abnormality or other medical condition or situation such that continued participation in the study would not be in the best interest of the subject. Subjects will be followed for the duration of the study for indicated safety assessments.
- Non-compliance with study procedures to the extent that it is potentially harmful to the subject or to the integrity of the study data.
- A change in the subject's baseline condition after enrollment so that the subject no longer meets the inclusion/exclusion criteria.

In any circumstance, every effort should be made to document the participants' outcome. Therefore all participants, even if the participant was withdrawn from the trial treatment, will be encouraged to come for the follow-up visits.

10.3. Early termination of the entire trial

The NECHRH, the DROS, the University of Buea, the DSMC may halt the study at any time following review of any safety concerns independent of the interim analysis. Halting the study requires immediate discontinuation of the study agent administered for all subjects and suspension of enrollment until a decision is made whether or not to continue study agent administration.

The halting criteria (as determined by site investigators) for an individual site include:

- two or more subjects experience the same or similar SAEs that are unexpected and are possibly, probably, or definitely related to the study agent

OR

- any safety issue that the site investigators determine should halt the study

The halting criteria (as determined by the study DSMC secondary to aggregate data review) for this study include:

- two or more of the same or similar AE in different subjects that are grade 3 or above and are unexpected and possibly, probably, or definitely related to the study agent

OR

- any safety issue that the study DSMC determines should halt the study

10.4. Report of termination of the trial

When the trial is prematurely terminated, the sponsor should submit a report to the NECHRH and the DROS within 30 (thirty) days. This report will include:

- Justification for the premature ending or of the temporary halt of the trial;
- Number of patients receiving treatment at the time of the study termination;
- Proposed management of patients receiving treatment at the time of halt or study termination;
- Implications of the discontinuation on the evaluation of the final results.

10.5. Notification of the end of the trial

The end of the clinical trial is the date of the last visit of the last participant undergoing the trial. At the end of the trial, the sponsor delegated person will notify the NECHRH about the trial completion and submit a preliminary report on the ethical evaluation within 30 days and a final report within 90 days after completion of the trial.

11. DATA COLLECTION, HANDLING AND RECORD KEEPING

11.1. Data Collection methods

The investigator has ultimate responsibility for the accuracy, authenticity, timely collection and reporting of all clinical, safety, laboratory data entered on the Case Report Forms (CRFs). Data will be captured using paper CRF specially designed for the study and approved by the IRBs. The CRFs must be signed by the investigator or by an authorized staff member to attest that the data contained on the CRFs is true. Any corrections to entries made in the CRFs, source documents must be dated, signed and explained (if necessary) and should not obscure the original entry. All paper CRFs will be secured in fireproof locking cabinet.

All clinical and laboratory procedures will be performed according to standard protocols governed by GCP and GLP guidelines, 21 CFR Part 11. Tools for qualitative assessments will be made available and validated prior to use.

All study personnel participating will undergo training in the procedures to be used in the study.

A work instruction's manual / manual of SOPs will be prepared at each site and will be made available to all study personnel and study monitors.

11.2. Electronic data capture (EDC)

Research data collected on paper CRFs will be transcribed and entered onto the REDCap (Research Electronic Data Capture: http://project-redcap.org/) system using double data entry. The EDC application is a secure encrypted web application for building and managing online surveys and databases specifically geared to support data capture for research studies. REDCap meets regulatory requirements for GCP/GCLP, 21 CFR Part 21 and HIPAA compliant with full audit trails capability for tracking data manipulation and user activity, as well as automated export procedures for seamless data downloads to Excel, PDF, and common statistical packages (SPSS, SAS, Stata, R). The equipment for data transfer may include laptops, or mobile applications for tablet computers and/or smartphones with either Android or iOS operating systems. All electronic tools will be password protected.

11.3. Data management

Study data will be managed using REDCap electronic data capture tools hosted at the University Hospital Bonn. REDCap (Research Electronic Data Capture) is a secure, web-based application designed to support data capture for research studies, providing: 1) an intuitive interface for data entry; 2) audit trails for tracking data manipulation and export procedures; 3) automated export procedures for seamless data downloads to common statistical packages; and 4) procedures for importing data from external sources [19].

Details on data management (procedures, responsibilities, data corrections, if any, which may be made by Data Management staff themselves, etc.) will be described in a data management plan prior to the trial. During the trial, the performance of data management and any deviations from the data management plan will be documented in a data management report. Before any data entry is performed, the trial database will be validated and the technical specifications of the database will be documented in a variable plan.

Processing of data is done via Double-Data-Entry. The two entries will then be compared with each other and verified. An audit trail will be created to provide an electronic record of which data were entered or subsequently changed, by whom and when.

SAS software will be used to review the data for completeness, consistency and plausibility. The checks to be programmed will be specified beforehand in a data validation plan, as required by the subject protocol. After running the check programs, the resulting queries will be sent to the investigator for review of his/her data. Answered queries will also be entered twice, verified and the updated data will then be transferred to the database. All programs which can be used to influence the data or the data quality will be validated (e.g. check programs, programs used for the input of external data, etc.).

All data will be checked for consistency and plausibility by the monitor and by the data management. Inconsistencies will be queried and discussed with the investigator. After data clearance the data base will be locked and data will be used for statistical analysis.

11.4. Trial site file

The trial site will be provided with a trial site file (ISF) containing all sponsor-specific essential and trial specific documents. The monitor will regularly check the trial site file for accuracy and completeness. The trial site file has to be stored locked and sure. After end of trial or early termination of the trial, the trial site file should be retained for 10 years at the site.

The ISF includes the subject identification list, where the investigator has to record the trial participation of each subject. This list allows identification of each subject and contains the subject number, the name, telephone number (if applicable) and the date of inclusion of the subject into the trial, and will be reviewed by the monitor for completeness. After end of the trial the subject identification list remains with the trial site.

The investigator should maintain a list of appropriately qualified persons to whom he/she has delegated trial duties. This list will be provided with the ISF, too.

12. MONITORING AND QUALITY ASSURANCE

During the clinical trial, quality control and quality assurance will be endured through monitoring, auditing and inspections by the national authorities.

12.1. Study Monitoring

To ensure accurate, complete, consistent, and reliable data, and ensure the patients´ safety, the investigator's site and trial procedures will be monitored by a representative of the sponsor. The sponsor's representative will visit the site:

- to evaluate the progress and recruitment of the trial,
- to review the source documents and CRFs for protocol compliance, accuracy and validation,
- to assess facilities and equipment,
- to check for protocol compliance,
- to assure the AE/SAE reporting,
- to verify proper handling and dispensing of the IMP(s), and other factors.

Frequency and scope of the monitoring visits will be defined in the Monitoring Plan for this trial which also includes the extent of source data verification that is required as a risk-adapted strategy rather than a full monitoring will be conducted.
The investigator agrees to cooperate with the monitor to ensure that any problems detected in the course of these monitoring visits are addressed and resolved, and therefore ensures the accuracy and consistency of the trial with GCP and all applicable laws. The investigator allows the monitor to have access to all trial related original data and documents relevant for the monitoring of the trial.

12.2. Audits and inspections

In accordance with GCP this trial may be selected for audit by representatives of the sponsor or for inspection by site responsible representatives of the competent national authorities.
The investigator agrees to give the auditor access to all relevant documents for review and to support the sponsor to solve possible audit findings concerning the trial conduct at the site.
After every audit the auditee(s) will rerceive an audit confirmation by the auditor. This document has to be filed together with the trial documentation and has to be made available also to the national authorities in case of an inspection.
At the end of the trial, a copy of the audit certificate(s) will be included in the final report. Archiving

12.2.1. Archiving by the sponsor

All clinical and experimental data (electronic or paper) and all essential documents inclusively the CRFs shall be kept in a secured place (metallic safety cabinets under lock) for a period of 15 years after completion of the trial and be made readily available for review upon request by the national authorities and the IRBs. The sponsor must archive all trial related documents according to regulatory requirements.

12.2.2. Archiving by the investigator

The investigator will maintain all subject documents as specified in the Essential Documents for conduct of a clinical trial (see ICH-GCP, section 8) and as required by the applicable regulatory requirements after completion of the clinical trial so that they will be available for audits and inspections by the authorities. The investigator will be responsible for the storage.
The following retention periods will apply after completion or stop of the clinical trial:

- all essential documents and trial related data must be retained securely for at least 15 years,
- the subject identification list for at least 15 years,
- medical records and other source documents for the longest possible period allowed by the institution.

The investigator should take arrangements to prevent accidental or premature destruction and illegitimate access to these documents.
To enable evaluations and/or audits from the sponsor or inspections from regulatory authorities, the investigator agrees to keep records, including the identity of all participating subjects (sufficient information to link records, e. g. CRFs and other records), all original signed informed consent forms, copies of all CRFs, serious adverse event reports, source documents, and detailed records of treatment disposition, drug accountability and adequate documentation of relevant correspondence (e. g. letters, meeting minutes, telephone calls reports).

13. ETHICAL CONSIDERATIONS

13.1. Basic principles

The trial will be carried out conforming to the principles of the Declaration of Helsinki 1964 (amended most recently in 2013) and according to Good Clinical Practice (GCP) guidelines and according to 21 CFR Part 11 guidelines. The principal investigator for this trial will submit a protocol to NECHRH prior to the commencement of the trial at that site.

This trial is registered under ISRCTN11881662.

13.2. Involvement of Ethics Committees and Regulatory Authorities

The protocol, the informed consent documents (ICFs) to be used in this study will be submitted to:

- The National Ethic Committee for Human Research, Yaoundé
- The institutional Review board, Faculty of Heath Sciences, University of Buea
- The Ethikkommission an der Medizinischen Fakultät der Rheinischen Friedrich-Wilhelms-Universität Bonn, Bonn, Germany.

Written documentation of approval of both the protocol and the informed consent will be provided by the sponsor before starting the study.

13.3. Responsibilities of the Investigator

By signing this protocol the investigator declares his/her commitment:

- to not enrol any person dependent on him/her or the sponsor in accordance with the principles of GCP
- to inform the subjects of the transmission of their pseudonymized data and to make sure that subjects unwilling to give consent to the processing of their data are not included into the trial
- to certify that he/she was informed of the pharmacological – toxicological issues and risks of the clinical trial
- to be qualified by education, training and experience to assume responsibility for the proper conduct of the subject

- to be thoroughly familiar with the appropriate use of the trial drug(s), as described in the protocol, the product information and other information sources provided by the sponsor
- to be aware of, and comply with GCP and the applicable regulatory requirements
- to maintain a list of appropriately qualified persons to whom the investigator has delegated significant subject related duties.

The investigator should conduct the clinical trial in compliance with this protocol. For this purpose, the document will be signed by the sponsor and the investigator. As a general rule, the investigator should not deviate from the protocol or make amendments to the protocol without the agreement of the sponsor/authorities/ethics committees (unless subject safety is at risk, see below).

Any deviations from the approved protocol should be documented and explained by the investigator or an individual who is designated by the investigator.

The investigator may deviate from the protocol or make an amendment to the protocol without prior approval of the ethics committee to eliminate immediate risks to the subjects. An amendment if necessary should subsequently be reported to the ethics committees, the sponsor or sponsor delegated person and the competent authorities, giving reasons.

13.4. Protocol amendments

Any substantial amendments to the protocol or subsequent changes to the informed consent form as a result of changes to the protocol must be sent to the NECHRH. Records of the NECHRH review and opinion of all documents pertaining to this trial must be kept on file by the investigator and are subject to regulatory authority and/or sponsor inspection during or after completion of the trial.
Protocol amendments detailing minor administrative changes should be submitted by the investigator to the NECHRH and regulatory authorities for notification purposes as appropriate.

13.5. Subject information

Written or witnessed thumb print consent will be obtained from every patient prior to any procedures being done specifically for the study.

Informed consent is a process where information is presented to enable persons to voluntarily decide whether or not to participate as a research subject. It is an on-going conversation between the human research subject and the researchers that begins before consent is given and continues until the end of the subject's involvement in the research.

Broadly, discussions about the research will provide essential information about the study and include: purpose, duration, experimental procedures, alternatives, risks and benefits. Subjects will be given the opportunity to ask questions and have them answered. The subjects will sign the informed consent document prior to undergoing any research procedures. The subjects may withdraw consent at any time throughout the course of the trial. A copy of the informed consent document will be given to the subjects for their records. The researcher will document the signing of the consent form in the case report form (CRF) and the original signed consent retained in a separate file along with other protocol specific documents required for compliance with GCP. The rights and welfare of the subjects will be protected by emphasizing to them that the quality of their medical care will not be adversely affected if they decline to participate in this study.

For illiterate patients, study information is given in the presence of an impartial, literate witness, who will read the information sheet to the patient or will witness the complete reading of the information sheet to the patient. The patient will give consent by thumb printing the ICF and the witness states that free, informed consent has been given by his/her signature on the ICF.

Patients with a suspicion of lymphedema will be invited to be screened for inclusion in the study. They will be given a patient informed consent form (ICF) about the study and will be explained the anticipated benefits and the potential risks associated with the protocol procedures. The principal investigator or a person designated by the PI will fully inform the patient, or the patient's legally acceptable representative. The language used will be as non-technical as possible and the patient will not unduly be influenced to participate in the study.

There will be a second informed consent form for "Sample storage, re-utilization and shipment". With this consent form the participant will be asked to give his/her consent for the storage and re-use of the samples taken during the clinical trial also after finalization of the trial to find new ways to better diagnose or treat podoconiosis. Since the development of new methods also takes place at the IMMIP Bonn, Germany, the LMU, Munich Germany or the partner institutes in Ghana and Tanzania, the participants will also be asked for their consent to have the samples shipped to Germany, Tanzania or Ghana. Patients will be told that there will be no negative effect on their participation in the trial if they decide not to sign this second ICF.

13.6. Obtaining informed consent

The principal investigator with the approval of the NECHRH will finalize the consent seeking process including the allocation of responsibility of obtaining consent from trial participants.

13.7. Confidentiality

All records will be kept confidential to the extent provided by national federal, state and local laws. The study monitors and other authorized representatives of the Sponsor may inspect all documents and records required to be maintained by the Investigator, including but not limited to, medical records. Specific study records will be kept in locked cabinets and all computer data, data entry programmes and networking programs will be password protected. Personal information such as patient names, hospital numbers and addresses will not be recorded on the CRFs used. If records are required for examination, this information will be blanked out; however, a secure record of the linkages between individuals and their records will be maintained. Clinical information will not be released without written permission of the subject, except as necessary for monitoring by NECHRH, the national regulatory authority, or the sponsor's designee. All such information will be anonymized.

13.8. Declaration of interests

Principal investigators for the overall trial and each study site will declare their financial and other competing interests prior to the commencement of the trial and these declarations will be available on file for inspection.

14. ADMINISTRATIVE CONSIDERATIONS

14.1. Patient Insurance

Every subject participating in the trial will be insured against any trial-related illness/injuries pursuant to the legal requirements which may occur during the trial. Excluded from this, however, are injuries to health and deterioration of illnesses already in existence which would have continued to exist even if the subject had not taken part in the clinical trial. The investigator will inform the subject of the existence of the insurance, including the obligations arising from it. The participants must be afford access to insurance documents and provided with a copy of the general conditions of insurance on request. The insurance cover is jeopardized if the subject fails to immediately report to the investigator or responsible physician any injury to health which

might have resulted from the participation in the clinical trial, or if she/he undergoes any other medical treatment (except for emergency treatment) without the investigator's knowledge before her/his participation in the clinical trial has officially ended. In case of any health impairment the subject is obliged to notify the investigator as soon as possible. The investigator is then obliged to notify the insurance and additionally to make a report to the sponsor. The subject insurance will be arranged by the sponsor delegated person.

The insurance company in Cameroon is : CPA; compagnie profesionnelle d'assurance du Cameroun

Email: cpasiege@yahoo.fr

Website: www.cpa-cameroun.com

This insurance covers trial related injuries to health up to a maximum of 2.000.000 CFA (3500 USD) per subject.

14.2. Incentives and expenses

Monetary compensation limited to loss of wages and transportation costs will be made to the subjects involved in this study in accordance with the guidelines prescribed by the national regulatory authorities and the respective IRBs.

Each participant will receive a lymphedema hygiene kit consisting essentially of a plastic bowl, 5 pieces of soap, 2 cotton towels.

Technical and material support will be provided to community health centers that will house the study.

The sponsor will cover patient care as it is related to the study, for the life of the protocol, and after the protocol for a reasonable short amount of time as jointly agreed.

14.3. Trial reports

Quarterly reports of the progress of a clinical trial starting from the date of issuance of the clinical trial certificate will be submitted to the University of Buea within 21 days after the end of the previous quarter (a quarter will be considered as three months beginning from the date of initiation of the clinical trial).

14.3.1. Final Report

In addition to the reports referred to above, the Principal Investigator/Sponsor delegated person who conducted the trial will, not later than 90 days after the completion of the trial, compile and submit a comprehensive formal report to DROS/MINSANTE.

The report (hard and soft copies (1 each)) will include a short but comprehensive summary of the essential findings of the trial and of its methodology and course.

14.4. Publication Policy

The results of the study should be published in peer reviewed journal. Authorship will be restricted to those persons that had a significant input into the design, implementation and analysis of the study. Professional writers will not be used.

15. INFORMATION SHEETS

15.1. Information sheet for screening and treatment (english version)

Patient Information Sheet for Screening, Enrolment and Treatment

TAKeOFF – PodoLEDoxy

Title: Doxycycline for treatment of non-filarial lymphedema due to podoconiosis (PodoLE) - a randomized double blind placebo-controlled trial

Principal Investigator: Prof. Samuel Wanji; Department of Microbiology and Parasitology, University of Buea, P.O. Box 63, Buea, Cameroon, swanji@yahoo.fr

Protocol identifying number: **TAKeOFF-5-0117**

Dear Madam / Sir,

You (or your child) are suspected to have non-filarial lymphedema (swelling of your leg) due to podoconiosis and are being invited to participate in a research study. This study investigates the effect of an antibiotic, doxycycline, in improving your condition. It also investigates whether this treatment can be beneficial to others who suffer from lymphedema. For you to decide whether you want to take part or not in this research, you have to know the risks and benefits so that you can make a well thought out decision. This preliminary and common information procedure is called “informed consent”.
Clinical trials are needed to gain or to widen the experience about efficacy and safety of drugs. It is therefore regulated by law to perform clinical trials before new drugs are approved or the application of already approved drugs can be expanded.

The study procedures are divided into:
Screening (1 day),
Enrolment (1 day),
Treatment (6 weeks) and
Supervision (24 months)

The conduct of this clinical trial by the University of Buea in collaboration with the Universities of Bonn and Munich in Germany, was approved by the Cameroon National Ethics Committee for Health Research on Humans. The Principal Investigator is Dr. Samuel Wanji, Faculty of Sciences, University of Buea, Cameroon; Tel.:+237 77 72 43 84, email: swanji@yahoo.fr.

Why is this study being done?

The current treatment of podoconiosis lymphedema focuses on improving the cleanliness of your leg, exercising and keeping the leg raised whenever possible as well as using shoes or slippers at all times. Additionally, your doctor might have prescribed antibiotics and antifungals for occasional acute inflammatory attacks.

In a recent study, a six-week course of doxycycline 200 mg (an antibiotic) prevented worsening and decreased the severity of lymphedema in patients who were treated with this drug. We ask you to participate in a study that will confirm this finding before it can be recommended for treatment of podoconiosis all over the world. The study for which we request your involvement will investigate the effect of doxycycline together with standard hygiene measures compared to standard hygiene measures alone (placebo matching doxycycline will be used). About 200 patients from the North-West region of Cameroon will participate in this study which is planned to last for about 3 years.

Why is the screening necessary?

Participation in the study is only possible if your lymphedema is due podoconios. The study will enroll participants with lymphedema stage 2 (mild) to 4 (advanced). Individuals with other diseases or conditions, who might not tolerate the study treatment, will be identified during the screening and excluded for participation. This document provides you with information on the study. Please read it or listen carefully when it is read to you. After you have asked any questions you may have, you will be asked whether you are willing to take part in the study. If you wish to proceed, you should date and sign/thumbprint the sheet together with the investigator in charge. You will receive one copy and we will keep one copy of the signed document. If you are found to be eligible for the study following this screening process in which some tests will be undertaken as described below, you will be enrolled into the study. Your participation in this research is entirely voluntary. There are no costs for you. It is your choice whether to participate or not. Whether you choose to participate or not will not alter your usual health care or involve any penalty or loss of benefits to which you are otherwise entitled. If you decide to participate in the study now, you may withdraw at any time and for any reason without penalty or loss of benefits.

What happens at the screening?

If you are interested in taking part in this study, you will be asked questions about your health and you are required to undergo a physical examination to determine your eligibility to participate in this study. Blood (25 ml, 3 tubes, about 2 and a half spoonfuls) and urine samples will be obtained for testing. These are to identify any illnesses other than your lymphedema. If you are a woman you will undergo a urine test to detect if you are pregnant. You will also be counseled against pregnancy during the treatment period. If you are found to have a condition that makes you ineligible to participate, you will be referred to the health authorities to receive treatment for that condition.

What will happen during the study?

Enrolment

At enrolment additional parameters will be obtained, including measurement of the lymphedema and a questionnaire with 12 questions to get an impression of the quality of your life. In the event the screening procedure will have occured more than 28 days prior to your enrolment, a small volume of blood will be drawn to recheck your health status for your safety. The size of your legs will be measured with tape and a scanner and the thickness of your skin will be determined using ultrasound, which are all painless procedures. Photographs of your limbs will be taken. In addition, you will be trained to follow simple hygiene measures of cleaning and taking care of your swollen leg (washing, care of the nails, exercises and use of footwear). You will have to follow these hygiene measures during the entire treatment period and beyond for a total duration of 3 years. Supplies will be provided to you for free at every visit of the research team.

Treatment

The study will investigate the effect of doxycycline together with standard hygiene measures compared to standard hygiene measures alone. About 200 individuals from trhe North-West region will participate in this study that will last for about 3 years. If you agree to participate in the study, you will receive either 2 tablets of doxycycline 100 mg, or a look-alike-placebo (2 tablets) for 6 weeks. However, the treatment you receive will be determined by chance (like drawing lots) and you cannot choose which treatment you receive. Harlf of the participants will receive 200 mg doxycycline and half will receive will receive a placebo tablets. This process is a common way to study the effect of new drugs and treatments. The treatment will include 84 tablets if you have a body weight above 50kg, two of the tablets taken once per day for 42 days under supervision. In case you have a body weight below 50kg, the amount of tablets will be reduced to 42 tablets, one per day. A trial clinician or pharmacist will come every day to deliver the tablets and ask for any side effects or problems. A blood test to ensure that your body is tolerating the treatment well (half a spoonful, 5 ml) will be done after you have taken your tablets for 21 days and will be repeated on your last day of treatment. Urine pregnancy tests (only for female patients) will be obtained before you take the first tablet and again every two weeks during treatment.

Supervision

The effect of the treatment will not be seen immediately, but after several months. For that reason the long supervision period of 24 months is necessary. After the treatment is finished you will be seen by members of the study team once every 2 months to record any acute attacks during the intervening period and to ensure that you are following the prescribed program of hygiene. A hygiene training will be carried out at 4, 6, 12, 18 and 24 months. In addition, at 6, 12 and 24 months you will undergo the same examinations carried out similar to the procedures at the beginning of the study (measurement of legs, skin thickness using ultrasound and photographs, blood draw of 25 ml, (3 tubes, about 2 and a half spoonfuls) and a quality of life questionnaire).

Potential risks:

Doxycycline

Doxycycline is an already marketed product for the treatment of several infectious diseases. It is an antibiotic, which has been used for decades and most side effects of this drug are well known. If, unexpectedly, new undesired effects of doxycycline will be reported, you will be immediately notified. Problems reported after doxycycline treatment are uncommon and include yellow colored teeth (especially in small children), sensitivity to sunlight (you are advised to avoid long periods in the sun without protection), diarrhea, nausea and discomfort on swallowing. Fungal infections are more common in patients receiving doxycycline treatment. Effects on the kidneys, liver or blood are rare, but a blood testing after 3 and 6 weeks of treatment will confirm that the treatment is tolerated without elevation of your liver values.

It is not allowed to use doxycycline during pregnancy, when breastfeeding or in children less than 8 years because it may damage developing bones. Because doxycycline may make oral contraceptives less effective, you are advised to use other effective methods of contraception (including abstinence) before, during and for at least 2 weeks after the completion of the treatment. If you are a woman and found to be pregnant during the treatment phase of the study you will be counseled about the potential risks and excluded from any further participation in the treatment. However, you will continue to benefit from medical care offered by the team to the study participants. Although many commonly used drugs (including iron for anemia, some drugs for controlling seizures and antacids for heartburn) may make doxycycline less effective, doxycycline has little effect on

other drugs with the exception of iron. Therefore, you will be asked about any medication you are taking and advised if necessary.

Placebo

One third of the patients in this study will receive an inactive, look-alike treatment to allow the study team to assess the effects of doxycycline in an unbiased manner. Although the placebo treatment is not active itself, all subjects in the study will undergo the same study regime including all physical assessments, measurements and limb hygiene training that are currently considered to be the best treatment available.
In the event that you experience any adverse effect of the study drug during the course of this study, you should immediately contact the doctor in charge of the study, who will determine what action to take. You will be informed of any new important knowledge about the drugs used in the study that may lead to you changing your mind about continuing. It is well possible that there will not be any adverse effects but you need to know that they might occur.

For women of childbearing age

All drugs are potentially dangerous to the unborn child. This is why you can only be included in the study if you are not pregnant and are using effective contraceptive methods. To avoid unnecessary risks, and with your consent, you will undergo a pregnancy test before you can be included in the study. You have to be aware that you should not get pregnant before, during and at least 2 weeks after treatment because the treatment could harm your unborn child. Therefore you should use an effective barrier method of contraception such as condoms, or other preventive methods including abstinence. If you are already taking birth control pills by mouth, you should use a barrier method of birth control in addition for the duration of the study treatment period since the efficiency of birth control pills may be reduced. Appropriate advice will be given during the consent and enrolment process and supplies may be provided should you request them. The pregnancy test will be repeated every 2 weeks until end of treatment. If you think you have become pregnant during the study and especially during the treatment period, you should inform the study doctor immediately. If you do become pregnant during the treatment period, you will be withdrawn immediately from treatment and followed up as appropriate. The study doctor (on behalf of the study sponsors) will follow up the progress of your pregnancy until after the birth of the baby.

Blood Drawing

The potential risks of the needle stick for blood drawing include pain, fainting, infection and bruising, or a small hematoma. The bruising may last up to 72 hours. Rarely, a swelling (hematoma) may appear which is easily treated with local pressure. Infections from the needle puncture are rare, but if this does occur, appropriate treatment will be given. At the screening and after 6, 12 and 24 months a blood draw to rule out concomitant diseases and for measuring lymphedema and inflammation associated factors will be done, requiring 25 ml of blood (3 tubes, about 2 and a half spoonfuls). During the treatment phase (after you have taken the tablets for 21 days and at the end of your treatment), a small amount of blood (5 ml, half a spoonful) will be taken to analyse liver enzymes (Gamma-GT, AST, ALT). This is done for safety reasons to ensure that you tolerate the treatment well.

Potential benefits:

By participating in the study you will benefit from thorough medical evaluations as well as receiving training and supplies for local care of lymphedema that by itself should improve your health. Two other studies some years ago have already shown a benefit for lymphedema by taking doxycycline.

Should the present study also be successful, those of you having received doxycycline will have had the added benefit of improvement in their disease state; those in the placebo arm of the study will be offered a six-week course of doxycycline at the end of the study. All participants will be informed about the outcome of the study after the 24 months period of supervision.

Other information:

During treatment you are advised not to take any medicine other than those that have been prescribed to you by the study doctor, as it could result in side effects or alter your blood test results. If you need treatment for some illnesses, then you should inform the study doctor.

Home visits

You will be asked to provide the research staff with contact details of 2 people who are not staying with you but who can be contacted when you are needed during screening, treatment and follow ups. We will need to visit you if you fail to keep your appointment or miss any treatment doses.

Specimen storage

At 5 time points during the study (at enrollment, at the end of treatment and after 6, 12 and 24 months) blood, urine and also saliva samples will be taken for measurements of factors related to inflammation and development of lymphedema. This blood, urine and saliva will be stored until the measurements can be performed in a specialized lab. At the end of the study the remaining blood, urine and saliva samples will be destroyed, unless you give your permission for further use of the samples for science in an additional consent form.

Reimbursement:

You will receive food items such as, milk, rice etc. worth about 5.000 CFA at each visit to compensate for your time spent on this study.
Should you have any injury or illness as a direct result of the study medication within 60 days of completing the treatment phase or study-specific procedures during the duration of the study you will be provided with local medical care free of charge. Should it be necessary to transfer you for care elsewhere, these costs will be covered by the sponsor through a specific insurance or guarantee.

Confidentiality:

The medical records of this study will be kept strictly confidential. Only authorized study personnel will have access to your data and samples. You will be identified by a code and not by your name. You will not be personally identified in any publication about this study. Data, photographs of your limbs and/or samples, which may be transferred to the collaborating institutes in Germany, Tanzania or Ghana will be provided in a non-identifiable way. However, the records of this study may be reviewed by inspectors of regulatory authorities, as well as study monitors, ethics committees and auditors who ensure the quality of the study. This is in accordance with regulatory requirements and patient's rights. By signing/thumbprinting the consent form, you agree to these procedures of handling your data.

What if the researchers learn new information during this study?

Results of this study or other scientific research may affect your willingness to continue to take part in this study. During the course of the study, you will be informed of any significant new findings

(good or bad), such as changes in the risks or benefits resulting from participation in the research, or new alternatives that might cause you to change your mind about continuing in the study. If the study investigators learn new information of this kind, the study investigators will share it with you. If new information is provided to you, your consent to continue participating in this study will be re-obtained.

Withdrawing from the study:

If you wish to interrupt your treatment or to withdraw your consent before the end of the study, please contact the doctor in charge. In addition, the study doctor may stop your participation in the treatment if you develop medical problems or if you do not take the medication and/or meet the appointment schedule as directed. In any case, you will neither be penalized nor lose any benefits to which you are otherwise entitled.

Whom do I call if I have questions or problems?

Mr. Bonekeh Yiyih John (Tel. No.: +237 677578405) has described to you what is going to be done, the risks, hazards, and benefits involved. You can contact the principal coordinator of the study Professor Wanji Samuel (Tel. No.: +237 677724384), and the trial clinicians (medical doctors), Dr. Djikeussi K. Tatiana (numéro de téléphone: +237 655190562) ; Dr. Vofo Brice (Tel: +237 674295139) ; Dr. Punjom Njefi yves (Tel: +237 675079280) ; Dr. Fozao Afou Adolph (Tel: +237 677802159), if you have any questions or problems regarding the clinical trial. Your contact at the Cameroon national Ethic Committee is +237 243 67 43 39.

15.2. Notice d'information pour le depistage et le traitement (version francaise)

Fiche d'information du patient pour le dépistage, le recrutement et le traitement

TAKeOFF – PodoLEDoxy

Titre : Doxycycline pour le traitement du lymphœdème de type Podoconiose (PodoLE) - un essai randomisée, en double aveugle, contrôlée contre placebo

Investigateur principal : Prof. Samuel Wanji; Département de Microbiologie et Parasitologie, Université de Buea, BP. 63, Buea, Cameroun, swanji@yahoo.fr

Numéro d'identification du protocole: **TAKeOFF-5-0117**

Cher(e) Madame ou Monsieur,

Vous (ou votre enfant) êtes soupçonné d'avoir un lymphœdème de type Podoconiose (gonflement de la jambe) et êtes invité (e) à participer à une étude de recherche. Cette étude examine l'effet d'un antibiotique, la doxycycline, dans l'amélioration de votre état. Il examine également si ce traitement peut être bénéfique pour les autres qui souffrent de lymphœdème. Pour que vous décidiez si vous voulez participer ou non à cette recherche, vous devez connaître les risques et les avantages afin que vous puissiez prendre une décision bien réfléchie. Cette procédure d'information préalable et commune est appelée "consentement éclairé".

Des essais cliniques sont nécessaires pour acquérir ou élargir l'expérience sur l'efficacité et l'innocuité des médicaments. Il est donc réglementé par la loi d'effectuer des essais cliniques avant que de nouveaux médicaments ne soient approuvés ou que l'application de médicaments déjà approuvés puisse être étendue.

Les procédures d'étude sont divisées en:
Dépistage (1 jour),
Inscription (1 jour),
Traitement (6 semaines) et
Supervision (24 mois)
La conduite de cet essai clinique par l'équipe de recherche du Département de Microbiologie et de parasitologie , Faculté des sciences, Université de Buea Cameroun, B.P. 63, Buea, Cameroun, en collaboration avec les Universités de Bonn et de Munich en Allemagne, a été approuvée par le comité national d'éthique du Cameroun. Le chercheur principal est: Prof. Samuel Wanji, parasitologie et entomologie de la santé publique; Chef de Département de Microbiologie et de Parasitologie, Université de Buea, B.P. 63, Buea, Cameroun, Tél .: +237 77 72 43 84, email: swanji@yahoo.fr.

Pourquoi cette étude est-elle en cours?

Le traitement actuel du lymphœdème est axé sur l'amélioration de la propreté et hygiène de la jambe, l'exercice et le maintien de la jambe dans la mesure du possible, ainsi que l'utilisation de chaussures ou de pantoufles en tout temps. De plus, votre médecin pourrait vous avoir prescrit des antibiotiques et des antifongiques pour des crises inflammatoires aiguës occasionnelles. Dans une étude récente, une cure de six semaines de doxycycline à 200 mg (un antibiotique) a empêché l'aggravation et diminué la sévérité du lymphoedème chez les patients qui ont été traités avec ce médicament. Nous vous demandons de participer à une étude qui confirmera cette découverte avant qu'elle puisse être proposée aux patients atteints de lymphœdème de type podoconiose. L'étude pour laquelle nous demandons votre participation examinera l'effet de la doxycycline avec les mesures d'hygiène standard par rapport aux mesures d'hygiène standard seules (un placebo correspondant à la doxycycline sera utilisé). Environ 200 patients du Nord-Ouest Cameroun participeront à étude qui devrait durer environ 3 ans.

Pourquoi le dépistage est-il nécessaire?

La participation à l'étude n'est possible que si votre lymphœdème est causée par des paticules de poussières volcaniques. L'étude recrutera des participants avec lymphoedème stade 2 (debut) à 4 (avancé). Les personnes atteintes d'autres maladies ou affections, qui pourraient ne pas tolérer le traitement en cours d'étude, seront identifiées lors du dépistage et exclus de l'‘étude. Cette feuille vous fournit des informations sur l'étude. S'il vous plaît lisez-la ou écoutez attentivement quand il vous est lu. Après avoir posé toutes les questions que vous pourriez avoir, il vous sera demandé si vous souhaitez participer à l'étude. Si vous souhaitez continuer, vous devriez dater et signer / ou poser votr empreinte sur la fiche avec l'enquêteur responsable. Vous recevrez une copie et nous conserverons une copie du document signé. Si vous êtes jugé admissible à l'étude à la suite de ce processus de sélection au cours duquel certains tests seront effectués comme décrit ci-dessous, vous serez inscrit à l'étude. Votre participation à cette recherche est entièrement volontaire. Il n'y a pas de frais pour vous. C'est votre choix de participer ou non. Que vous choisissiez de participer ou non ne changera pas vos soins de santé habituels ou n'impliquera aucune pénalité ou perte d'avantages auxquels vous avez autrement droit. Si vous décidez de participer à l'étude maintenant, vous pouvez vous retirer à tout moment et pour n'importe quelle raison sans pénalité ni perte d'avantages.

Que se passe-t-il lors du dépistage?

Si vous êtes intéressé à prendre part à cette étude, on vous posera des questions sur votre santé et vous devrez subir un examen physique pour déterminer votre admissibilité à participer à cette étude. Du sang (25 ml, 3 tubes, environ 2 cuillerées et demie) et des échantillons d'urine seront prélevés pour être testés. Ceux-ci vont permettre d'identifier toutes les maladies autres que votre lymphoedème. Si vous êtes une femme, vous subirez un test d'urine pour détecter si vous êtes enceinte. Vous serez également conseillées de ne pas tomber enceinte pendant la période de traitement. Si vous êtes atteint d'une affection qui vous rend inadmissible à participer, vous serez référé aux autorités sanitaires pour recevoir un traitement pour cette affection.

Que va-t-il se passer pendant l'étude?

Inscription

Lors de l'inscription, des paramètres supplémentaires seront obtenus, y compris la mesure du lymphœdème et un questionnaire avec 12 questions pour avoir une idée de la qualité de votre vie. Dans le cas où la procédure de dépistage aura eu lieu plus de 28 jours avant votre inscription, un

petit volume de sang sera prélevé pour revérifier votre état de santé pour votre sécurité. La taille de vos jambes sera mesurée avec du ruban adhésif et un scanner et l'épaisseur de votre peau sera déterminée en utilisant l'échographie, qui sont toutes des procédures indolores. Des photos de vos membres seront prises. De plus, vous serez formé à suivre des mesures d'hygiène simples de nettoyage et de soin de votre jambe enflée (lavage, soin des ongles, exercices et utilisation de chaussures). Vous devrez suivre ces mesures d'hygiène pendant toute la durée du traitement et au-delà pour une durée totale de 3 ans. Les materiels vous seront fournies gratuitement à chaque visite de l'équipe de recherche.

Traitement
L'étude examinera l'effet de la doxycycline avec les mesures d'hygiène standard par rapport aux mesures d'hygiène standard seules. Environ 200 personnes participeront à cette étude qui durera environ 2 ans. Si vous acceptez de participer à l'étude, vous recevrez 200 mg de doxycycline, ou un comprimé semblable (un placebo) pendant 6 semaines. Cependant, le traitement que vous recevrez sera déterminé par hasard (comme le tirage au sort) et vous ne pourrez pas choisir le traitement que vous recevrez. Une moitié des participants recevra 200 mg de doxycycline et une autre moitié recevra un comprimé placebo. Ce processus est un moyen courant d'étudier l'effet de nouveaux médicaments et traitements. Le traitement comprendra 84 comprimés si vous avez un poids supérieur à 50 kg, deux comprimés pris une fois par jour pendant 42 jours sous surveillance. Dans le cas où vous avez un poids corporel inférieur à 50 kg, la quantité de comprimés sera réduite à 42 comprimés, un par jour. Un medecin ou un pharmacien viendra tous les jours délivrer les comprimés et demander des effets secondaires ou des problèmes. Un test sanguin pour s'assurer que votre corps tolère bien le traitement (une demi-cuillerée, 5 ml) sera effectué après avoir pris vos comprimés pendant 21 jours et sera répété le dernier jour de votre traitement. Les tests de grossesse urinaire (uniquement pour les patientes) seront obtenus avant de prendre le premier comprimé et de nouveau toutes les deux semaines pendant le traitement.

Supervision
L'effet du traitement ne sera pas visible immédiatement, mais après plusieurs mois. Pour cette raison, la longue période de surveillance de 24 mois est nécessaire. Une fois le traitement terminé, les membres de l'équipe d'étude vous verront une fois tous les 2 mois pour enregistrer toute crise aiguë pendant la période intermédiaire et vous assurer que vous suivez le programme d'hygiène prescrit. Une formation à l'hygiène sera réalisée à 4, 6, 12, 18 et 24 mois. De plus, à 6, 12 et 24 mois, vous subirez les mêmes examens que ceux pratiqués au début de l'étude (mesure des jambes, épaisseur de la peau à l'aide d'ultrasons et de photos, prélèvement sanguin de 25 ml, (3 tubes, environ 2 cuillères à soupe et demi) et un questionnaire sur la qualité de vie).

Potentiels risques

Doxycycline
La doxycycline est un produit déjà commercialisé pour le traitement de plusieurs maladies infectieuses. C'est un antibiotique qui a été utilisé pendant des décennies et la plupart des effets secondaires de ce médicament sont bien connus. Si, de façon inattendue, de nouveaux effets indésirables de la doxycycline sont signalés, vous en serez immédiatement informé(e)s. Les problèmes signalés après un traitement à la doxycycline sont rares et comprennent des dents jaunes (surtout chez les jeunes enfants), une sensibilité au soleil (il est conseillé d'éviter de longues périodes au soleil sans protection), de la diarrhée, des nausées et une gêne à avaler. Les infections fongiques sont plus fréquentes chez les patients recevant un traitement à la doxycycline. Effets sur les reins, le foie ou le sang sont rares, mais un test sanguin après 3 et 6 semaines de traitement confirmera que le traitement est toléré sans élévation de vos valeurs biochimiques du foie.
Il est interdit d'utiliser la doxycycline pendant la grossesse, l'allaitement ou chez les enfants de moins de 8 ans, car cela peut endommager les os en développement. Parce que la doxycycline peut rendre les contraceptifs oraux moins efficaces, il est conseillé d'utiliser d'autres méthodes de contraception efficaces (y compris l'abstinence) avant, pendant et pendant au moins 2 semaines après la fin du traitement. Si vous êtes une femme et que vous êtes enceinte pendant la phase de

traitement de l'étude, vous serez conseillée sur les risques potentiels et exclue de toute participation ultérieure au traitement. Cependant, vous continuerez à bénéficier des soins médicaux offerts par l'équipe aux participants à l'étude.

Bien que de nombreux médicaments couramment utilisés (dont le fer contre l'anémie, certains médicaments contre les crises convulsives et les antiacides contre les brûlures d'estomac) rendent la doxycycline moins efficace, la doxycycline a peu d'effet sur les autres médicaments, à l'exception du fer. Par conséquent, vous serez interrogé sur tout médicament que vous prenez et conseillé si nécessaire.

Placebo

Un tiers des patients de cette étude recevront un traitement inactif et similaire pour permettre à l'équipe d'étude d'évaluer les effets de la doxycycline de manière non biaisée. Bien que le traitement par placebo ne soit pas actif lui-même, tous les sujets de l'étude subiront le même régime d'étude incluant toutes les évaluations physiques, les mesures et la formation à l'hygiène des membres actuellement considérés comme les meilleurs traitements disponibles.

Dans le cas où vous ressentez un effet indésirable du médicament à l'étude au cours de cette étude, vous devez immédiatement contacter le médecin responsable de l'étude, qui déterminera les mesures à prendre. Vous serez informé de toute nouvelle connaissance importante sur les médicaments utilisés dans l'étude qui peut vous amener à changer d'avis sur la poursuite. Il est bien possible qu'il n'y ait pas d'effets indésirables, mais vous devez savoir qu'ils peuvent se produire.

Pour les femmes en âge de procréer

Tous les médicaments sont potentiellement dangereux pour l'enfant à naître. C'est pourquoi vous ne serez inclues dans l'étude si vous n'êtes pas enceinte et utilisez des méthodes contraceptives efficaces pendant la duree de l'étude. Pour éviter des risques inutiles, et avec votre consentement, vous subirez un test de grossesse avant de pouvoir être inclus dans l'étude. Vous devez être conscient que vous ne devriez pas tomber enceinte avant, pendant et au moins 2 semaines après le traitement, car le traitement pourrait nuire à votre enfant à naître. Par conséquent, vous devez utiliser une méthode contraceptive efficace comme les préservatifs ou d'autres méthodes préventives, y compris l'abstinence. Si vous prenez déjà des pilules contraceptives par voie orale, vous devez utiliser une méthode contraceptive de barrière en plus de la durée de la période de traitement à l'étude, car l'efficacité des pilules contraceptives peut être réduite. Des conseils appropriés seront donnés au cours du processus de consentement et d'inscription et des fournitures peuvent être fournies si vous en faites la demande. Le test de grossesse sera répété toutes les 2 semaines jusqu'à la fin du traitement. Si vous pensez être tombée enceinte pendant l'étude et surtout pendant la période de traitement, vous devez en informer immédiatement le médecin de l'étude. Si vous devenez enceinte pendant la période de traitement, vous serez immédiatement retiré du traitement et suivi de façon appropriée. Le médecin de l'étude (au nom des promoteurs de l'étude) suivra les progrès de votre grossesse jusqu'à la naissance du bébé.

Prelevement de sang

Les risques potentiels de la piqûre d'aiguille pour la prise de sang comprennent la douleur, l'évanouissement, l'infection et les ecchymoses, ou un petit hématome. Les ecchymoses peuvent durer jusqu'à 72 heures. Rarement, un gonflement (hématome) peut apparaître qui est facilement traité avec la pression locale. Les infections dues à la ponction à l'aiguille sont rares, mais si cela se produit, un traitement approprié sera administré. Au dépistage et après 6, 12 et 24 mois, une prise de sang pour exclure les maladies concomitantes et pour mesurer le lymphœdème et les facteurs associés à l'inflammation sera faite, nécessitant 25 ml de sang (3 tubes, environ 2 cuillères et demie) Pendant le traitement phase (après avoir pris les comprimés pendant 21 jours et à la fin de votre traitement) une petite quantité de sang (5 ml, une demi-cuillerée) sera prélevée pour analyser les enzymes hépatiques (Gamma-GT, AST, ALT). Ceci est fait pour des raisons de sécurité afin de s'assurer que vous tolérez bien le traitement.

Bénefices Potentiels:

En participant à l'étude, vous bénéficierez d'évaluations médicales approfondies ainsi que de la formation et des fournitures pour les soins locaux de lymphœdème qui, en soi, devrait améliorer votre santé. Deux autres études il y a quelques années ont déjà montré un bénéfice pour le lymphoedème en prenant de la doxycycline. Si la présente étude a également du succès, ceux d'entre vous qui ont reçu de la doxycycline auront eu le bénéfice supplémentaire d'une amélioration de leur état pathologique; ceux du groupe placebo de l'étude se verront offrir un cours de six semaines de doxycycline à la fin de l'étude. Tous les participants seront informés des résultats de l'étude après la période de supervision de 24 mois.

Autres informations:

Pendant le traitement, il est conseillé de ne pas prendre d'autres médicaments que ceux qui vous ont été prescrits par le médecin de l'étude, car cela pourrait entraîner des effets secondaires ou modifier les résultats de votre test sanguin. Si vous avez besoin d'un traitement pour certaines maladies, vous devez en informer le médecin de l'étude.

Visites à domicile

Il vous sera demandé de fournir au personnel de recherche les coordonnées de 2 personnes qui ne restent pas avec vous mais qui peuvent être contactées lorsque vous en avez besoin pendant le dépistage, le traitement et les suivis. Nous aurons besoin de vous rendre visite si vous ne respectez pas votre rendez-vous ou si vous manquez des doses de traitement.

Stockage des échantillons

À 5 moments de l'étude (à l'inclusion, à la fin du traitement et après 6, 12 et 24 mois), des échantillons de sang, d'urine et de salive seront prélevés pour mesurer les facteurs liés à l'inflammation et au développement du lymphœdème. Ce sang, l'urine et la salive seront stockés jusqu'à ce que les mesures puissent être effectuées dans un laboratoire spécialisé. À la fin de l'étude, les échantillons de sang, d'urine et de salive restants seront détruits, à moins que vous ne donniez votre permission pour une utilisation ultérieure des échantillons à des fins scientifiques dans un formulaire de consentement supplémentaire.

Remboursement:

Vous recevrez à chaque visite des denrées alimentaires telles que du lait, du riz, etc. d'une valeur d'environ 5.000 Fcfa pour compenser le temps que vous avez consacré à cette étude.
Si vous avez une blessure ou une maladie résultant directement du médicament à l'étude dans les 60 jours suivant la fin de la phase de traitement ou des procédures spécifiques à l'étude pendant la durée de l'étude, vous recevrez gratuitement des soins médicaux locaux. S'il s'avère nécessaire de vous transférer pour des soins ailleurs, ces frais seront couverts par le promoteur au moyen d'une assurance ou d'une garantie spécifique.

Confidentialité:

Les dossiers médicaux de cette étude resteront strictement confidentiels. Seul le personnel autorisé de l'étude aura accès à vos données et échantillons. Vous serez identifié par un code et non par votre nom. Vous ne serez identifié personnellement dans aucune publication concernant cette étude. Les données, photographies de vos membres et / ou échantillons qui peuvent être transférés aux instituts collaborateurs en Allemagne, en Tanzanie ou au Ghana seront fournis de manière non identifiable. Cependant, les dossiers de cette étude peuvent être examinés par les inspecteurs des autorités de réglementation, ainsi que par les contrôleurs d'étude, les comités d'éthique et les vérificateurs qui assurent la qualité de l'étude. Ceci est en conformité avec les exigences

réglementaires et les droits du patient. En signant / empreinte digitale le formulaire de consentement, vous acceptez ces procédures de traitement de vos données.

Et si les chercheurs apprennent de nouvelles informations au cours de cette étude?

Les résultats de cette étude ou d'autres recherches scientifiques peuvent affecter votre volonté de continuer à participer à cette étude. Au cours de l'étude, vous serez informé des nouveaux résultats significatifs (bons ou mauvais), tels que les changements dans les risques ou les avantages résultant de la participation à la recherche, ou de nouvelles alternatives qui pourraient vous faire changer d'avis sur la poursuite dans l'étude. Si les enquêteurs de l'étude apprennent de nouvelles informations de ce type, les enquêteurs de l'étude le partageront avec vous. Si de nouvelles informations vous sont fournies, votre consentement à continuer de participer à cette étude sera de nouveau obtenu.

Retrait de l'étude:

Si vous souhaitez interrompre votre traitement ou retirer votre consentement avant la fin de l'étude, veuillez contacter le médecin responsable. De plus, le médecin de l'étude peut interrompre votre participation au traitement si vous développez des problèmes médicaux ou si vous ne prenez pas le médicament et / ou si vous respectez le calendrier de rendez-vous tel qu'indiqué. Dans tous les cas, vous ne serez pas pénalisé ni ne perdrez aucun avantage auquel vous avez autrement droit.

Qui dois-je appeler si j'ai des questions ou des problèmes?

Mr. Bonekeh Yiyih John (numéro de téléphone: +237 677578405) de l'equipe de recherche vous a décrit ce qui va être fait, les risques, les dangers et les avantages impliqués. Vous pouvez contacter le Coordinateur principal de l'étude, le Professeur Samuel Wanji de l'Université de Buea, (numéro de téléphone: +237 77 72 43 84), les medecins cliniciens de l'étude, Dr. Djikeussi K. Tatiana (numéro de téléphone: +237 655190562) ; Dr. Vofo Brice (numéro de téléphone: +237 674295139) ; Dr. Punjom Njefi yves (numéro de téléphone: +237 675079280) ; Dr. Fozao Afou Adolph (numéro de téléphone: +237 677802159), si vous avez des questions ou des problèmes concernant l'essai clinique. Vous pouvez également contacter le Comité national d'éthique de la recherche pour la santé humaine (CNERSH), qui a approuvé notre plan d'études. Si vous souhaitez trouver plus d'informations sur ce comité ou souhaitez poser des questions sur cette étude et vos droits en tant que participant, vous pouvez contacter le comité en appelant le (+237) 243 67 43 39.

15.3. Information sheet for sample storage, re-utilization and shipment (english version)

Patient Information Sheet for Sample Storage, Re-utilization and Shipment

TAKeOFF – PodoLEDoxy

Title: Doxycycline for treatment of non-filarial lymphedema due to podoconiosis (PodoLE) - a randomized double blind placebo-controlled trial

Principal Investigator: Prof. Samuel Wanji; Department of Microbiology and Parasitology, University of Buea, P.O. Box 63, Buea, Cameroon, swanji@yahoo.fr

Protocol identifying number: **TAKeOFF-5-0117**

Dear Madam or Sir,

You (or your child) are participating in the clinical trial TAKeOFF-PodoLEDoxy. During the clinical trial, blood, urine and saliva samples will be collected from you. A portion of the samples will directly be used to ensure your safe participation in the study; others will be used for research purposes directly linked to the trial. The research team would like to store your samples in freezers at the laboratories in Bamenda, the University of Buea, Cameroon and in the laboratories of the partners institutes in Germany (Institute for Medical Microbiology, Immunology and Parasitology (IMMIP), University Hospital of Bonn, Department for Infectious Diseases & Tropical Medicine, Klinikum of the University of Munich), Tanzania (The National Institute for Medical Research (NIMR)), Ghana (the Kumasi Centre for Collaborative Research (KCCR) in Kumasi) to re-use them eventually for further studies that will help to better understand podoconiosis or to develop new diagnostic tools.

In this Patient Information document, we will explain the reasons for these further studies to you. The permission for sample storage, re-utilization and shipment is voluntary. Your samples will only be stored, re-used or shipped if you sign this informed consent form and with the approval from the responsible ethics committee. You will receive one copy and we will keep one copy of the signed document. Refusing to give your permission will not alter your usual health care or involve any penalty or loss of benefits to which you are otherwise entitled and you can still participate in this study. In case you refuse to give your permission, your samples will be destroyed and discarded after completion of the trial.

What happens with the blood samples?

During screening and after 6, 12 and 24 months, blood samples will be taken (25 ml, 3 tubes which is about 2 and a half spoonfuls) for measuring research parameters associated with the clinical study. These parameters are related to the development and progress of lymphedema like tissue

growing factors. During screening the blood will also be used for for liver and kidney function test (Gamma-GT, AST, ALT, creatinine) to ensure that you are suitable for the study. After 3 and 6 weeks of treatment with doxycycline a small amount of blood (5 ml 1 tube, about half a spoonful of blood) will be taken and liver values will be measured again to ensure that you are tolerating the medicine well. Leftovers of the samples will be stored in a -80° freezer, aliquots will be used in Cameroon, Ghana, Germany or Tanzania to look at different parameters before and after treatment. This is necessary to better understand the development of podoconiosis and to find new diagnostic parameters, for example, molecules which are more sensitive to diagnose the disease.

What happens with the urine samples?

Urine samples will be taken from women of child-bearing potential at screening and during the treatment phase. Additionally, urine samples from all participants will be taken at enrolment, after 6 weeks of treatment and at 6, 12 and 24 months. The urine samples (up to 25 ml) will be stored and aliquots will be used in Cameroon, Ghana, Germany or Tanzania to look at different parameters before and after treatment to better understand the disease and to find new diagnostic tools. This is important, for example, to find markers for podoconiosis without having to draw fresh blood.

What happens with the Saliva samples?

Saliva samples (2 ml) of all participants will be taken at enrolment and at 6, 12 and 24 months. The saliva samples will be stored and aliquots will be used in Cameroon, Ghana, Germany or Tanzania to look at different parameters before and after treatment to better understand the disease and to find new diagnostic tools. This is important, for example, to find markers for podoconiosis without having to draw fresh blood.

Confidentiality:

Your data and samples will be coded in a non-identifiable way and all information will be kept confidential. During the ongoing study, only the researchers will be able to trace which blood, urine or saliva sample belongs to the participant in order to give the correct information to the respective persons.

No profit:

None of the remaining samples will be sold and any research which uses the samples will first have to be approved by the responsible ethics committee.

Whom do I call if I have questions or want to withdraw my consent?

Mr. Bonekeh Yiyih John (Tel. No.: +237 677578405) has described to you what is going to be done, the risks, hazards, and benefits involved. You can contact the principal coordinator of the study Professor Wanji Samuel (Tel. No.: +237 677724384), and the trial clinicians (medical doctors), Dr. Djikeussi K. Tatiana (numéro de téléphone: +237 655190562) ; Dr. Vofo Brice (Tel: +237 674295139) ; Dr. Punjom Njefi yves (Tel: +237 675079280) ; Dr. Fozao Afou Adolph (Tel: +237 677802159), if you have any questions or problems regarding the clinical trial. Your contact at the Cameroon national Ethic Committee is +237 243 67 43 39.

15.4. Notice d'information pour le stockage d'echantillons, reutilisation et expedition (version francaise)

Fiche d'information du patient pour le stockage d'échantillons, réutilisation et expédition

TAKeOFF – PodoLEDoxy

Titre : Doxycycline pour le traitement du lymphœdème de type Podoconiose (PodoLE) - un essai randomisée, en double aveugle, contrôlée contre placebo

Investigateur principal : Prof. Samuel Wanji; Département de Microbiologie et Parasitologie, Université de Buea, BP. 63, Buea, Cameroun, swanji@yahoo.fr

Numéro d'identification du protocole : **TAKeOFF-5-0117**

Cher(e) Madame ou Monsieur,

Vous (ou votre enfant) participe(z) à essai clinique TAKeOFF-PodoLEDoxy. Pendant l'essai clinique, des échantillons de sang, d'urines et de salive vous seront prélevés. Une partie des échantillons sera directement utiliser pour assurer votre participation en toute sécurité à l'étude ; d'autres parties seront utiliser à des fins de recherche directement liées à l'essai. L'équipe de recherche souhaite stocker vos échantillons dans des congélateurs des laboratoires de Bamenda, de l'Université de Buea et des laboratoires des instituts partenaires du Ghana au Centre de Recherche Collaborative de Kumasi (KCCR) à Kumasi, en Allemagne (Institut de Microbiologie Médicale, Immunologie et Parasitologie (IMMIP), Hôpital Universitaire de Bonn, Département de Maladies Infectieuses et de Médecine Tropical, Klinikum de l'Université de Munich), en Tanzanie (Institut National de Recherche Médicale (INRM)) ; et les utiliser éventuellement pour d'autres études approfondies qui aideront à mieux comprendre la podoconiose ou à développer de nouveaux outils de diagnostic.

Dans cette Notice d'information du patient nous vous expliquerons les raisons pour ces études approfondies. La permission pour le stockage, la réutilisation et l'expédition des échantillons est volontaire. Vos échantillons ne seront stockés, réutiliser ou expédie que si vous signez cette fiche de consentement éclairé et avec l'approbation du comité d'éthiques responsable. Vous recevrez une copie et nous conserveront une copie de ce document signé. Refuser de donner votre permission ne changera pas la qualité de soins médicaux que vous recevez habituellement et n'impliquera aucune pénalité ou perte d'avantages aux quels vous avez droit et vous pourriez toujours participer à cette étude. Au cas où vous refuseriez de donner votre permission, vos échantillons seront détruits et jetés après la fin de l'essai.

Qu'est-ce qui se passe avec l'échantillon de sang?

Pendant le dépistage et 6,12 et 24 mois après, des échantillons sanguins seront collectés (25ml, 3tubes équivalents à 2cuillieres et demie) pour mesurer les paramètres de recherche associes à l'étude clinique. Ces paramètres sont liés au développement et à la progression des facteurs de croissance tissulaire du lymphœdème comme, des marqueurs d'activation immunitaire et des biomarqueurs spécifiques. Pendant le dépistage, le sang

sera également utilisé pour mesurer les paramètres de fonction hépatique et rénale (Gamma-GT, ASAT, ALAT, créatinine) pour s’assurer que vous êtes qualifié pour l’étude. Après 3 et 6 semaines de traitement avec la Doxycycline, une petite quantité de sang (1 tube de 5ml, environ une demi-cuillère de sang) sera prélever et les valeurs hépatiques seront mesurer à nouveau pour s’assurer que vous tolérez bien le médicament. Le reste des échantillons seront stockés à -80 dégrées dans un congélateur a au Cameroun, au Ghana, en Allemagne et en Tanzanie pour rechercher des diffèrent paramètres avant et après le traitement. Ceci est nécessaire pour mieux comprendre le développement de lymphoedeme de type podoconiose et pour rechercher des nouveaux paramètres diagnostiques, par exemple, des molécules plus sensibles au diagnostic de la maladie.

Qu’est-ce qui se passe avec les échantillons d’urines?

Des échantillons d’urine seront prélevés des femmes en âge de procréer pendant le dépistage et pendant la phase de traitement. De plus, des échantillons d’urines de tous les participants seront prélevés à l’inscription, après 6 semaines de traitement et a 6, 12 et 24mois. Les échantillons d’urines (jusqu’à 25ml) seront stockés et des aliquotes seront utiliser au Ghana, en Allemagne, en Tanzanie ou au Cameroun pour rechercher des différents paramètres avant et après le traitement afin de mieux comprendre la maladie et de trouver des nouveaux outils de diagnostics. Ceci est important, par exemple, pour trouver des marqueurs de la podoconiose sans avoir à prélever du sang frais.

Qu’est-ce qui se passe avec les échantillons de salive?

Des échantillons de salive (2ml) de tous les participants seront prélevés à l’inscription et à 6, 12 et 24 mois. Ces échantillons de salive seront stockés et des aliquotes seront utilisées au Ghana, en Allemagne, en Tanzanie ou au Cameroun pour rechercher des différents paramètres avant et après le traitement afin de mieux comprendre la maladie et de trouver de nouveaux outils de diagnostics. Ceci est important, par exemple, pour trouver des marqueurs de la podoconiose sans avoir à prélever du sang frais.

Confidentialité

Vos données et échantillons seront codées d’une manière non identifiable et toutes les informations resteront confidentielles. Pendant l’étude en cours, seul les chercheurs seront en mesure de tracer quel échantillon de sang, d’urine ou de salive appartient à un participant afin de donner l’information correcte aux personnes respectives.

Pas de profit

Aucun des échantillons restant ne sera vendu et toute recherche qui utilise les échantillons devra d'abord être approuvée par un comité d'éthique responsable.

Qui dois-je appeler si j'ai des questions ou si je veux retirer mon consentement ?

Mr. Bonekeh Yiyih John (numéro de téléphone : +237 677578405) de l’equipe de recherche vous a décrit ce qui va être fait, les risques, les dangers et les avantages impliqués. Vous pouvez contacter le Coordonateur principal de l’étude, le Professeur Samuel Wanji de l’Université de Buea, (numéro de téléphone : +237 77 72 43 84), les medecins cliniciens de l’étude, Dr. Djikeussi K. Tatiana (numéro de téléphone: +237 655190562) ; Dr. Vofo Brice (numéro de téléphone: +237 674295139) ; Dr. Punjom Njefi yves (numéro de téléphone: +237 675079280) ; Dr. Fozao Afou Adolph (numéro de téléphone: +237 677802159), si vous avez des questions ou des problèmes concernant l'essai clinique. Vous pouvez également contacter le Comité national d’éthique de la recherche pour la santé humaine (CNERSH), qui a approuvé notre plan d’études. Si vous souhaitez trouver plus d’informations sur ce comité ou souhaitez poser des questions sur cette étude et vos droits en tant que participant, vous pouvez contacter le comité en appelant le (+237) 243 67 43 39.

16.INFORMED CONSENT AND ASSENT FORMS

16.1. Informed consent form for screening, enrolment and treatment (english version)

TAKeOFF – PodoLEDoxy

Title of the study: Doxycycline for treatment of non-filarial lymphedema due to podoconiosis (PodoLE) - a randomized double blind placebo-controlled trial

Principal Investigator: Prof. Samuel Wanji; Department of Microbiology and Parasitology, University of Buea, P.O. Box 63, Buea, Cameroon, swanji@yahoo.fr

Tackling the Obstacles to Fight Filariasis and Podoconiosis

Protocol identifying number: **TAKeOFF-5-0117**

Consent form for for screening, enrolment and treatment for adults participating in the TAKeOFF-PodoLEDoxy study

Name of the participant (in block letters): ______________________________

Date of birth (year of birth, if exact date is unknown): |__|__| / |__|__| / |__|__|__|__|
(dd / mm / yyyy)

Indiv. No.: |__|__| - |__|__|__| - |__|__|__|
(country) - (village code) - (consecutive patient no)

I hereby certify that the contents of the Informed Consent Form have been read by me/interpreted and explained to me in detail in a language I understand (Pidgin, English, French)

By:______________________________
(Name of investigator in block letters)

I fully understand the content of the Informed Consent Form. The information given to me has permitted me to make a fully informed and free decision about my participation in the clinical trial. I am aware that my participation is voluntary and that I can withdraw at any time and for any reason without penalty or loss of benefits. By signing this consent form, I do not waive any legal rights, and the investigator(s) or sponsor are not relieved of any liability they may have.

I thereby append my signature/mark (right thumbprint) to this Informed Consent Form, as evidence of my agreement to participate in all procedures required for the clinical trial.

Date: |__|__| / |__|__| / |__|__|__|__|
(dd / mm / yyyy)

____
(Participant's signature)

____
(Participant's name in block letters)

If needed, thumbprint of participant:

WITNESS (independent from research team)

I hereby certify that I was present when the contents of the Informed Consent Form were read/ interpreted and explained in the (Pidgin, English, French) language to

____
(Name of participant in block letters)

He/she seems to have fully understood the contents of the Informed Consent Form before appending his/her signature or making his/her mark (right thumb print) to this Informed Consent Form in my presence as evidence of his/her agreement to participate in the above mentioned clinical trial.

Date: |__|__| / |__|__| / |__|__|__|__|
(dd / mm / yyyy)

____
(Signature of the witness)

____
(Name of the witness in block letters)

INFORMANT (Investigator)

I hereby certify that the contents of the Informed Consent Form were interpreted and explained by me in the (Pidgin, English, French) language to

____
(Name of participant in block letters)

He/she seems to have fully understood the contents of the Informed Consent Form before appending his/her signature or making his/her mark (right thumb print) to this Informed Consent Form in my presence as evidence of his/her agreement to participate in the above mentioned clinical trial.

Date: |__|__| / |__|__| / |__|__|__|__|
(dd / mm / yyyy)

____
(Signature of the investigator)

____
(Name of the investigator in block letters)

**Assessment of informed consent:**		
Do you understand the consent form?	☐ yes	☐ no
Did you have enough time for your personal decision?	☐ yes	☐ no
Do you understand that blood will be drawn during this study?	☐ yes	☐ no
**If female:** Do you understand that you should not get pregnant before, during and at least for 2 weeks after the treatment period and that you have to use other methods than oral contraceptive medication (e.g. condoms, contraceptive coil, diaphragm, abstinence) to prevent pregnancy?	☐ yes	☐ no

**If male:**	☐ not applicable	
Can you refuse to participate in the study at any time?	☐ yes	☐ no
Is there any charge for taking part in the clinical trial?	☐ yes	☐ no
Are you forced to participate in the clinical trial?	☐ yes	☐ no
Do you have any questions?	☐ yes	☐ no

If yes, documentation of questions:

____

____

____

____

____

Do you know who to call if you have questions?	☐ yes	☐ no
Do you agree, that photographs of your limbs will be taken?	☐ yes	☐ no

16.2. Fiche de consentement éclairé pour le depistage, le recrutement et le traitement (version francaise)

TAKeOFF – PodoLEDoxy
Titre : Doxycycline pour le traitement du lymphœdème de type Podoconiose (PodoLE) - un essai randomisée, en double aveugle, contrôlée contre placebo

Investigateur principal : Prof. Samuel Wanji; Département de Microbiologie et Parasitologie, Université de Buea, BP. 63, Buea, Cameroun, swanji@yahoo.fr

Numéro d'identification du protocole: **TAKeOFF-5-0117**

Formulaire de consentement éclairé pour le depistage, le recrutement et le traitement pour adulte participant à l'étude TAKeOFF-PodoLEDoxy

Nom du participant (en majuscules): ________________________________

Date de naissance (année, si la date exacte est inconnue): |__|__| / |__|__| / |__|__|__|__|
(dd / mm / yyyy)

No. Indiv. : |__|__| - |__|__|__| - |__|__|__|
(Pays) - (code village) - (N° consecutive du patient)

J'atteste que le contenu du formulaire de consentement éclairé a été lu par moi / interprété et expliqué en détail dans une langue que je comprends (Pidgin, Anglais, Français)

Par:________________________________
(Nom de l'enquêteur en lettres majuscule)

Je comprends parfaitement le contenu du formulaire de consentement éclairé. L'information qui m'est donnée m'a permis de prendre une décision en toute connaissance de cause quant à ma participation à l'essai clinique. Je suis conscient que ma participation est volontaire et que je peux me retirer à tout moment et pour n'importe quelle raison sans pénalité ni perte d'avantages. En signant ce formulaire de consentement, je ne renonce à aucun droit légal, et l'investigateur (s) ou le sponsor ne sont pas dégagés de toute responsabilité qu'ils pourraient avoir.
J'ajoute ainsi ma signature / empreinte (empreinte du pouce) à ce formulaire de consentement éclairé, comme preuve de mon consentement à participer à toutes les procédures requises pour l'essai clinique.

Date: |__|__| / |__|__| / |__|__|__|__|
(jj / mm / aaaa)

Si nécessaire, empreinte du participant:

____
(Signature du participant)

____
(Nom du participant en lettres majuscules)

TÉMOIN (indépendant de l'équipe de recherche)

Je certifie par la présente que j'étais présent lorsque le contenu du Formulaire de consentement éclairé a été lu / interprété et expliqué dans la langue (Pidgin, Anglais, Français)

____

(Nom du participant en lettres majuscules)

Il/elle semble avoir parfaitement compris(e) le contenu du Formulaire de consentement éclairé avant d'apposer sa signature ou d'apposer sa marque (empreinte du pouce droit) sur ce Formulaire de consentement éclairé en ma présence comme preuve de son consentement à participer à l'essai clinique mentionné ci-dessus.

Date: |__|__| / |__|__| / |__|__|__|__|
(dd / mm / yyyy)

____

(Signature du témoin)

____

(Nom du témoin en lettres majuscules)

INFORMATEUR (Enquêteur)

Je certifie par la présente que le contenu du formulaire de consentement éclairé a été interprété et expliqué par moi dans la langue (Pidgin, Anglais, Français)

____

(Nom du participant en lettres majuscules)

Il semble avoir parfaitement compris le contenu du Formulaire de consentement éclairé avant d'apposer sa signature ou d'apposer sa marque (empreinte du pouce droit) sur ce Formulaire de consentement éclairé en ma présence comme preuve de son consentement à participer à l'essai clinique mentionné ci-dessus.

Date: |__|__| / |__|__| / |__|__|__|__|
(jj / mm / aaaa)

____

(Signature de l'enquêteur)

____

(Nom de l'enquêteur en lettres majuscules)

Évaluation du consentement éclairé:

Comprenez-vous le formulaire de consentement? ☐ oui ☐ non

As-tu eu assez de temps pour ta décision personnelle? ☐ oui ☐ non

Comprenez-vous que du sang sera prélevé au cours de cette étude? ☐ oui ☐ non

Si vous êtes une femme: Est-ce que vous comprenez que vous ne devriez pas tomber enceinte? avant, pendant et au moins pendant 2 semaines après la période de traitement et que vous devez utiliser d'autres méthodes que la contraception orale médicaments (par exemple préservatifs, spirale contraceptive, diaphragme, abstinence, DIU) pour prévenir la grossesse? ☐ oui ☐ non

Si vous etes un homme: ☐ non applicable

Pouvez-vous refuser de participer à l'étude à tout moment? ☐ oui ☐ non

Y a-t-il des frais pour participer à l'essai clinique? ☐ oui ☐ non

Es-tu obligé de participer à l'essai clinique? ☐ oui ☐ non

Avez-vous des questions? ☐ oui ☐ non

Si oui, documentation des questions:

____

____

____

____

____

Savez-vous qui appeler si vous avez des questions? ☐ oui ☐ non

Est-ce que vous êtes d'accord, que les photographies de les membres seront prises? ☐ oui ☐ non

16.3. Informed consent forms for sample storage, re-utilization and shipment (english version)

TAKeOFF – PodoLEDoxy
Title: Doxycycline for treatment of non-filarial lymphedema due to podoconiosis (PodoLE) - a randomized double blind placebo-controlled trial

Principal Investigator: Prof. Samuel Wanji; Department of Microbiology and Parasitology, University of Buea, P.O. Box 63, Buea, Cameroon, swanji@yahoo.fr

Tackling the Obstacles to Fight Filariasis and Podoconiosis

Protocol identifying number: **TAKeOFF-5-0117**

Additional consent form for adults participating in the TAKeOFF-PodoLEDoxy study for sample storage, re-utilization and shipment

Name of the participant (in block letters): ____

Date of birth (year of birth, if exact date is unknown): |__|__| / |__|__| / |__|__|__|__|
(dd / mm / yyyy)

Indiv. No.: |__|__| - |__|__|__|__| - |__|__|__|
(country) - (village code) - (consecutive patient no)

I hereby certify that the contents of the Informed Consent Form have been read by me/ interpreted and explained to me in detail in a language I understand (Pidgin, English, French), (*underline applicable*).

By:____
(Name of investigator in block letters)

The information given to me has permitted me to make a fully informed and free decision about the storage, re-utilization and shipment of aliquots of my blood, urine, saliva and any tissue samples. If any of the blood, urine, saliva and tissue samples I have provided for this research project is unused or leftover.

Blood

☐ I give permission for my blood sample to be stored.

☐ I give permission for aliquots of my blood sample to be shipped to the German Universities of Bonn or Munich or to the institutes in Tanzania and Ghana

☐ I give my permission for aliquots of my blood sample to be stored and used in future research which has received proper ethical approval from the responsible ethics committee

or

☐ I give permission for aliquots of my blood sample to be stored and used in future research

except for research about ____________________ (name type of research)

Urine

☐ I give permission for my urine sample to be stored.

☐ I give permission for my urine sample to be shipped to the German Universities of Bonn or Munich or to the institutes in Tanzania and Ghana

☐ I give my permission for my urine sample to be stored and used in future research of any type which has received proper ethical approval from the responsible ethics committee

or

☐ I give permission for urine sample to be stored and used in future research except for research about ____________________________________ (name type of research)

Saliva

☐ I give permission for my saliva to be stored.

☐ I give permission for my saliva to be shipped to the German Universities of Bonn or Munich or to the institutes in Tanzania and Ghana

☐ I give my permission for saliva sample to be stored and used in future research of any type which has received proper ethical approval from the responsible ethics committee

or

☐ I give permission for saliva to be stored and used in future research except for research about ____________________________________ (name type of research)

I am aware that I can withdraw my consent at any time and for any reason without penalty or loss of benefits. By signing this consent form, I do not waive any legal rights, and the investigator(s) or sponsor are not relieved of any liability they may have.

I thereby append my signature/ mark (right thumbprint) to this Informed Consent Form, as evidence of my agreement to storage, re-utilization and shipment of my samples.

Date: |__|__| / |__|__| / |__|__|__|__|
(dd / mm / yyyy)

If needed, thumbprint of participant:

____
(Participant's signature)

____
(Participant's name in block letters)

WITNESS (independent from research team)

I hereby certify that I was present when the contents of the Informed Consent Form were read/ interpreted and explained in the language (Pidgin, English, French) (*underline applicable)* to

____

(Name of participant in block letters)

He/she seems to have fully understood the contents of the Informed Consent Form before appending his/her signature or making his/her mark (right thumb print) to this Informed Consent Form in my presence as evidence of his/her agreement to storage, re-utilization and shipment of his/ her samples.

Date: |__|__| / |__|__| / |__|__|__|__|
(dd / mm / yyyy)

____
(Signature of the witness)

____
(Name of the witness in block letters)

Informant (Investigator)

I hereby certify that the contents of the Informed Consent Form were interpreted and explained by me in a language he/she understands ((Pidgin, English, French) (*underline applicable)*) to

____
(Name of participant in block letters)

He/she seems to have fully understood the contents of the Informed Consent Form before appending his/her signature or making his/her mark (right thumb print) to this Informed Consent Form in my presence as evidence of his/her agreement to storage, re-utilization and shipment of samples.

Date: |__|__| / |__|__| / |__|__|__|__|
(dd / mm / yyyy)

____
(Signature of the investigator)

____
(Name of the investigator in block letters)

16.4. Fiche de consentement éclairé pour le stockage d'echantillons, reutilisation et expedition (version francaise)

TAKeOFF – PodoLEDoxy

Titre : Doxycycline pour le traitement du lymphœdème de type Podoconiose (PodoLE) - un essai randomisée, en double aveugle, contrôlée contre placebo

Investigateur principal : Prof. Samuel Wanji; Département de Microbiologie et Parasitologie, Université de Buea, BP. 63, Buea, Cameroun, swanji@yahoo.fr

Tackling the Obstacles to Fight Filariasis and Podoconiosis

Numéro d'identification du protocole : **TAKeOFF-5-0117**

Fiche de consentement additionnel pour des adultes qui participent à l'étude TAKeOFF-PodoLEDoxy pour le stockage, réutilisation et l'expédition des échantillons

Nom du participant (en majuscule) : ____

Date de naissance (année de naissance si date exact est inconnu) :

|__|__| / |__|__| / |__|__|__|__|
(Jj / mm / aaaa)

No. Individuel: |__|__| - |__|__|__| - |__|__|__|
(Pays) - (code du village) - (numéro du patient consécutif)

Je certifie par la présente que le contenu du formulaire de consentement éclairé a été lu par moi / interprété et expliqué en détail à moi dans une langue que je comprends (Pidgin, Anglais, Français) (*souligner si applicable*).

Par : ____
(Nom de l'investigateur en majuscule)

Les informations qui m'ont été communiquées m'ont permis de prendre une décision en toute connaissance de cause concernant le stockage, la réutilisation et l'expédition d'aliquotes de mon sang, d'urine, de salive et de tout échantillon de tissu. Si l'un des échantillons de sang, d'urine, de salive et de tissus que j'ai fournis pour ce projet de recherche est inutilisé ou reste.

<u>Sang</u>

☐ Je donne la permission de stocker mon échantillon de sang.

☐ Je donne la permission pour que des aliquotes de mon échantillon de sang soient expédiés à l'Université Allemand de Bonn ou Munich ou aux instituts en Tanzanie et au Ghana

☐ Je donne ma permission pour que des aliquotes de mon échantillon de sang sois stocker et utiliser dans des recherches futures qui auront reçu l'approbation éthique du comité d'éthique responsable

ou

☐ Je donne ma permission pour que des aliquotes de mon échantillon de sang sois stocker et utiliser dans des recherches futures sauf des recherches sur ________________ (nom du type de recherche)

Urine

☐ Je donne la permission de stocker mon échantillon d'urine.

☐ Je donne la permission d'expédie mon échantillon d'urine à l'Université Allemand de Bonn ou Munich ou aux instituts en Tanzanie et au Cameroun

☐ Je donne ma permission de stocker et d'utiliser mon échantillon d'urine dans n'importe quel type de recherches future qui auront reçu l'approbation éthique du comité d'éthique responsable

ou

☐ Je donne la permission de stocker et d'utiliser mon échantillon d'urine dans des recherches future sauf des recherches sur ________________ (nom du type de recherche)

Salive

☐ Je donne la permission de stocker ma salive.

☐ Je donne la permission d'expédie ma salive à l'Université Allemand de Bonn ou Munich ou aux instituts en Tanzanie et au Cameroun

☐ Je donne ma permission de stocker et d'utiliser mon échantillon de salive dans n'importe quel type de recherches future qui auront reçu l'approbation éthique du comité d'éthique responsable

ou

☐ Je donne la permission de stocker et d'utiliser la salive dans des recherches future sauf des recherches sur ________________ (nom du type de recherche)

Je suis conscient que je peux retirer mon consentement à tout moment et pour n'importe quelle raison sans pénalité ni perte d'avantages. En signant ce formulaire de consentement, je ne renonce à aucun droit légal, et l'investigateur (s) ou le sponsor ne sont pas dégagés de toute responsabilité qu'ils pourraient avoir.

J'ajoute ainsi ma signature / empreinte (empreinte du pouce droit) à ce formulaire de consentement éclairé, comme preuve de mon accord pour le stockage, la réutilisation et l'expédition de mes échantillons.

Date: |__|__| / |__|__| / |__|__|__|__|
(Jj / mm / aaaa)

Si besoin, empreinte du participant:

☐

____
(Signature du participant)

____
(Nom du participant en majuscule)

TEMOIN (indépendant de l’équipe de recherche)

Je certifie par la présente que j'étais présent lorsque le contenu du formulaire de consentement éclairé a été lu / interprété et expliqué en langue (Pidgin, Anglais, Français) (*souligner si applicable*).

____

(Nom du participant en majuscule)

Il / elle semble avoir parfaitement compris le contenu du Formulaire de consentement éclairé avant d'apposer sa signature ou d'apposer sa marque (empreinte du pouce droit) sur ce Formulaire de consentement éclairé en ma présence comme preuve de son consentement au stockage, réutilisation et expédition de ses échantillons.

Date: |__|__| / |__|__| / |__|__|__|__|
(Jj / mm / aaaa)

____

(Signature du témoin)

____

(Nom du témoin en majuscule)

Informateur (Enquêteur)

Je certifie par la présente que le contenu du formulaire de consentement éclairé a été interprété et expliqué par moi dans une langue qu'il/elle comprend (Pidgin, Anglais, Français) (*souligner si applicable*).

____

(Nom du participant en majuscule)

Il / elle semble avoir parfaitement compris le contenu du Formulaire de consentement éclairé avant d'apposer sa signature ou d'apposer sa marque (empreinte du pouce droit) sur ce Formulaire de consentement éclairé en ma présence comme preuve de son consentement au stockage, réutilisation et expédition d'échantillons.

Date: |__|__| / |__|__| / |__|__|__|__|
(Jj / mm / aaaa)

____

(Signature de l’enquêteur)

____

(Nom de l’enquêteur en majuscule)

16.5. Assent form for screening, enrolment and treatment (english version)

TAKeOFF – PodoLEDoxy
Title: Doxycycline for treatment of non-filarial lymphedema due to podoconiosis (PodoLE) - a randomized double blind placebo-controlled trial

Principal Investigator: Prof. Samuel Wanji; Department of Microbiology and Parasitology, University of Buea, P.O. Box 63, Buea, Cameroon, swanji@yahoo.fr

Protocol identifying number: **TAKeOFF-5-0117**

Assent form for for screening, enrolment and treatment for adolescents (aged 14 to 20 years) participating in the TAKeOFF-PodoLEDoxy study

Name of the participant (in block letters): ________________________________

Date of birth (year of birth, if exact date is unknown): |__|__| / |__|__| / |__|__|__|__|
(dd / mm / yyyy)

Indiv. No.: |__|__| - |__|__|__|__| - |__|__|__|__|
(country) - (village code) - (consecutive patient no)

I hereby certify that the contents of the Informed Consent Form have been read by me/ interpreted and explained to me and my parents in detail in the (Pidgin, English, French) language.
By:________________________________
(Name of investigator in block letters)

I fully understand the content of the Informed Consent Form. The information given to me has permitted me to make a fully informed and free decision about my participation in the clinical trial. I am aware that my participation is voluntary and that I can withdraw at any time and for any reason without penalty or loss of benefits. By signing this consent form, I do not waive any legal rights, and the investigator(s) or sponsor are not relieved of any liability they may have.

I thereby append my signature/mark (right thumbprint) to this Informed Consent Form, as evidence of my agreement to participate in all procedures required for the clinical trial.

Date: |__|__| / |__|__| / |__|__|__|__|
(dd / mm / yyyy)

If needed, thumbprint of participant:

____
(Participant's signature)

____
(Participant's name in block letters)

Parent's signature

I hereby certify that I was present when the contents of the Informed Consent Form were read/interpreted and explained in the language (Pidgin, English, French) language to my son/daughter

____
(Name of participant in block letters)

He/she seems to have fully understood the contents of the Informed Consent Form before appending his/her signature or making his/her mark (right thumb print) to this Informed Consent Form. As the legal representative I hereby agree with the participation.

Date: |__|__| / |__|__| / |__|__|__|__|
(dd / mm / yyyy)

If needed, thumbprint of parent:

____
(Signature of the parent)

____
(Name of the parent in block letters)

→ WITNESS (independent from research team)

I hereby certify that I was present when the contents of the Informed Consent Form were read/ interpreted and explained in the language (Pidgin, English, French) language to

____
(Name of participant in block letters)

and his/her legal representative.

He/she and his/her legal representative seem to have fully understood the contents of the Informed Consent Form before appending their signatures or making their marks (right thumb print) to this Informed Consent Form in my presence as evidence of their agreement to participate in the above mentioned clinical trial.

Date: |__|__| / |__|__| / |__|__|__|__|
(dd / mm / yyyy)

____
(Signature of the witness)

____
(Name of the witness in block letters)

→ INFORMANT (Investigator)

I hereby certify that the contents of the Informed Consent Form were interpreted and explained by me in the (Pidgin, English, French) language to

____
(Name of participant in block letters)

and his/her legal representative.

He/she and his/her legal representative seem to have fully understood the contents of the Informed

Consent Form before appending their signatures or making their marks (right thumb print) to this Informed Consent Form in my presence as evidence of their agreement to participate in the above mentioned clinical trial.

Date: |__|__| / |__|__| / |__|__|__|__|
(dd / mm / yyyy)

____
(Signature of the investigator)

____
(Name of the investigator in block letters)

Assessment of informed consent:

Do you understand the consent form?	☐ yes	☐ no
Did you have enough time for your personal decision?	☐ yes	☐ no
Do you understand that blood will be drawn during this study?	☐ yes	☐ no
**If female:** Do you understand that you should not get pregnant before, during and at least for 2 weeks after the treatment period and that you have to use other methods than oral contraceptive medication (e.g. condoms, contraceptive coil, diaphragm, abstinence) to prevent pregnancy?	☐ yes	☐ no
**If male:**	☐ not applicable	
Can you refuse to participate in the study at any time?	☐ yes	☐ no
Is there any charge for taking part in the clinical trial?	☐ yes	☐ no
Are you forced to participate in the clinical trial?	☐ yes	☐ no
Do you have any questions?	☐ yes	☐ no

If yes, documentation of questions:

____

____

____

____

____

Do you know who to call if you have questions?	☐ yes	☐ no
Do you agree, that photographs of your limbs will be taken?	☐ yes	☐ no

16.6. Fiche d'assentiment pour le depistage, le recrutement et le traitement (version francaise)

TAKeOFF – PodoLEDoxy

Titre : Doxycycline pour le traitement du lymphœdème de type Podoconiose (PodoLE) - un essai randomisée, en double aveugle, contrôlée contre placebo

Investigateur principal : Prof. Samuel Wanji; Département de Microbiologie et Parasitologie, Université de Buea, BP. 63, Buea, Cameroun, swanji@yahoo.fr

Numéro d'identification du protocole: **TAKeOFF-5-0117**

Formulaire d'assentiment pour le depistage, le recrutement et le traitement pour adolescents (âgés de 14 à 20 ans) participant à l'étude TAKeOFF-PodoLEDoxy

Nom du participant (en majuscules): ____

Date de naissance (année, si la date exacte est inconnue) : |__|__| / |__|__| / |__|__|__|__|
(dd / mm / yyyy)

No Indiv..: |__|__| - |__|__|__| - |__|__|__|
(Pays) - (code village) - (N° consécutif du patient)

Je certifie par la présente que le contenu du formulaire de consentement éclairé a été lu par moi / interprété et expliqué à moi et mes parents en détail dans la langue (Pidgin, Anglais, Français).
Par:____
(Nom de l'enquêteur en lettres majuscules)

Je comprends parfaitement le contenu du formulaire de consentement éclairé. L'information qui m'est donnée m'a permis de prendre une décision en toute connaissance de cause quant à ma participation à l'essai clinique. Je suis conscient que ma participation est volontaire et que je peux me retirer à tout moment et pour n'importe quelle raison sans pénalité ni perte d'avantages. En signant ce formulaire de consentement, je ne renonce à aucun droit légal, et l'investigateur (s) ou le sponsor ne sont pas dégagés de toute responsabilité qu'ils pourraient avoir.
J'ajoute ainsi ma signature / empreinte (empreinte du pouce) à ce formulaire de consentement éclairé, comme preuve de mon consentement à participer à toutes les procédures requises pour l'essai clinique.

Date: |__|__| / |__|__| / |__|__|__|__|
(jj / mm / aaaa)

Si nécessaire, empreinte du participant:

____
(Signature du participant)

____
(Nom du participant en lettres majuscules)

La signature des parents

Je certifie par la présente que j'étais présent lorsque le contenu du formulaire de consentement éclairé a été lu / interprété et expliqué dans la langue (Pidgin, Anglais, Français) à mon fils / ma fille

____
(Nom du participant en lettres majuscules)

Il / elle semble avoir parfaitement compris le contenu du Formulaire de consentement éclairé avant d'apposer sa signature ou d'apposer sa marque (empreinte du pouce droit) sur ce formulaire de consentement éclairé. En tant que représentant légal, je suis d'accord avec la participation.

Date: |__|__| / |__|__| / |__|__|__|__|
(dd / mm / yyyy)

Si nécessaire, empreinte du parent:

____
(Signature du parent)

____
(Nom du parent en majuscules)

TÉMOIN (indépendant de l'équipe de recherche)

Je certifie par la présente que j'étais présent lorsque le contenu du Formulaire de consentement éclairé a été lu / interprété et expliqué dans la langue (Pidgin, Anglais, Français)

____
(Nom du participant en lettres majuscules)

et son représentant légal.

Il / elle et son / sa représentant (e) légal (e) semblent avoir bien compris le contenu du Formulaire de consentement éclairé avant d'apposer leur signature ou de faire leur marque (empreinte imprimée) en ma présence comme preuve de leur consentement à participer à l'essai clinique mentionné ci-dessus.

Date: |__|__| / |__|__| / |__|__|__|__|
(dd / mm / yyyy)

____
(Signature du témoin)

____
(Nom du témoin en lettres majuscules)

INFORMATEUR (Enquêteur)

Je certifie par la présente que le contenu du formulaire de consentement éclairé a été interprété et expliqué par moi dans la langue (Pidgin, Anglais, Français)

____
(Nom du participant en lettres majuscules)

et son représentant légal.

Il / elle et son / sa représentant (e) légal (e) semblent avoir bien compris le contenu du Formulaire de consentement éclairé avant d'apposer leur signature ou de faire leur marque (empreinte imprimée) en ma présence comme preuve de leur consentement à participer à l'essai clinique mentionné ci-dessus.

Date: |__|__| / |__|__| / |__|__|__|__|
(dd / mm / yyyy)

____
(Signature de l'enquêteur)

____
(Nom de l'enquêteur en lettres majuscules)

Évaluation du consentement éclairé:

Comprenez-vous le formulaire de consentement? ☐ oui ☐ non

As-tu eu assez de temps pour ta décision personnelle? ☐ oui ☐ non

Comprenez-vous que du sang sera prélevé au cours de cette étude? ☐ oui ☐ non

Si vous êtes une femme: Est-ce que vous comprenez que vous ne devriez pas tomber enceinte? avant, pendant et au moins pendant 2 semaines après la période de traitement et que vous devez utiliser d'autres méthodes que la contraception orale médicaments (par exemple préservatifs, spirale contraceptive, diaphragme, abstinence, DIU) pour prévenir la grossesse? ☐ oui ☐ non

Si vous etes un homme: ☐ non applicable

Pouvez-vous refuser de participer à l'étude à tout moment? ☐ oui ☐ non

Y a-t-il des frais pour participer à l'essai clinique? ☐ oui ☐ non

Es-tu obligé de participer à l'essai clinique? ☐ oui ☐ non

Avez-vous des questions? ☐ oui ☐ non

Si oui, documentation des questions:

____

____

____

____

____

Savez-vous qui appeler si vous avez des questions? ☐ oui ☐ non

Est-ce que vous êtes d'accord, que les photographies de les membres seront prises? ☐ oui ☐ non

16.7. Assent form for sample storage, re-utilization and shipment (english version)

TAKeOFF – PodoLEDoxy
Title: Doxycycline for treatment of non-filarial lymphedema due to podoconiosis (PodoLE) - a randomized double blind placebo-controlled trial

Principal Investigator: Prof. Samuel Wanji; Department of Microbiology and Parasitology, University of Buea, P.O. Box 63, Buea, Cameroon, swanji@yahoo.fr

Protocol identifying number: **TAKeOFF-5-0117**

Additional consent form for adults participating in the TAKeOFF-PodoLEDoxy study for sample storage, re-utilization and shipment

Name of the participant (in block letters): ____

Date of birth (year of birth, if exact date is unknown): |__|__| / |__|__| / |__|__|__|__|
(dd / mm / yyyy)

Indiv. No.: |__|__| - |__|__|__| - |__|__|__|
(country) - (village code) - (consecutive patient no)

I hereby certify that the contents of the Informed Consent Form have been read by me/ interpreted and explained to me in detail in a language I understand (Pidgin, English, French), (*underline applicable*).

By:____
(Name of investigator in block letters)

The information given to me has permitted me to make a fully informed and free decision about the storage, re-utilization and shipment of aliquots of my blood, urine, saliva and any tissue samples. If any of the blood, urine, saliva and tissue samples I have provided for this research project is unused or leftover.

Blood

☐ I give permission for my blood sample to be stored.

☐ I give permission for aliquots of my blood sample to be shipped to the German Universities of Bonn or Munich or to the institutes in Tanzania and Ghana

☐ I give my permission for aliquots of my blood sample to be stored and used in future research which has received proper ethical approval from the responsible ethics committee

or

☐ I give permission for aliquots of my blood sample to be stored and used in future research except for research about ____________________ (name type of research)

Urine

☐ I give permission for my urine sample to be stored.

☐ I give permission for my urine sample to be shipped to the German Universities of Bonn or Munich or to the institutes in Tanzania and Ghana

☐ I give my permission for my urine sample to be stored and used in future research of any type which has received proper ethical approval from the responsible ethics committee

or

☐ I give permission for urine sample to be stored and used in future research except for research about ____________________________________ (name type of research)

Saliva

☐ I give permission for my saliva to be stored.

☐ I give permission for my saliva to be shipped to the German Universities of Bonn or Munich or to the institutes in Tanzania and Ghana

☐ I give my permission for saliva sample to be stored and used in future research of any type which has received proper ethical approval from the responsible ethics committee

or

☐ I give permission for saliva to be stored and used in future research except for research about ____________________________________ (name type of research)

I am aware that I can withdraw my consent at any time and for any reason without penalty or loss of benefits. By signing this consent form, I do not waive any legal rights, and the investigator(s) or sponsor are not relieved of any liability they may have.

I thereby append my signature/ mark (right thumbprint) to this Informed Consent Form, as evidence of my agreement to storage, re-utilization and shipment of my samples.

Date: |__|__| / |__|__| / |__|__|__|__|
(dd / mm / yyyy)

If needed, thumbprint of participant:

____
(Participant's signature)

____
(Participant's name in block letters)

WITNESS (independent from research team)

I hereby certify that I was present when the contents of the Informed Consent Form were read/ interpreted and explained in the language (Pidgin, English, French) (*underline applicable)* to

____
(Name of participant in block letters)

He/she seems to have fully understood the contents of the Informed Consent Form before appending his/her signature or making his/her mark (right thumb print) to this Informed Consent Form in my presence as evidence of his/her agreement to storage, re-utilization and shipment of his/ her samples.

Date: |__|__| / |__|__| / |__|__|__|__|
(dd / mm / yyyy)

____
(Signature of the witness)

____
(Name of the witness in block letters)

Informant (Investigator)

I hereby certify that the contents of the Informed Consent Form were interpreted and explained by me in a language he/she understands ((Pidgin, English, French) (*underline applicable)*) to

____
(Name of participant in block letters)

He/she seems to have fully understood the contents of the Informed Consent Form before appending his/her signature or making his/her mark (right thumb print) to this Informed Consent Form in my presence as evidence of his/her agreement to storage, re-utilization and shipment of samples.

Date: |__|__| / |__|__| / |__|__|__|__|
(dd / mm / yyyy)

____
(Signature of the investigator)

____
(Name of the investigator in block letters)

16.8. Fiche d'assentiment pour le stockage d'echantillons, reutilisation et expedition (version francaise)

TAKeOFF – PodoLEDoxy
Titre : Doxycycline pour le traitement du lymphœdème de type Podoconiose (PodoLE) - un essai randomisée, en double aveugle, contrôlée contre placebo

Investigateur principal : Prof. Samuel Wanji; Département de Microbiologie et Parasitologie, Université de Buea, BP. 63, Buea, Cameroun, swanji@yahoo.fr

Numéro d'identification du protocole : **TAKeOFF-5-0117**

Formulaire d'assentiment supplémentaire pour les adolescents (âgés de 14 à 20 ans) participant à l'étude TAKeOFF-PodoLEDoxy pour le stockage, la réutilisation et l'expédition d'échantillon

Nom du participant (en majuscule) : ____

Date de naissance (année de naissance si date exact est inconnu) :

|__|__| / |__|__| / |__|__|__|__|
(Jj / mm / aaaa)

No. Individuel: |__|__| - |__|__|__| - |__|__|__|

(Pays) - (code du village) - (numéro du patient consécutif)

Je certifie par la présente que le contenu du formulaire de consentement éclairé a été lu par moi / interprété et expliqué à mes parents et moi en détail en une langue que je comprends (Pidgin, Anglais, Français) (*souligner si applicable*).

Par : ____

(Nom de l'investigateur en majuscule)

Les informations qui m'ont été communiquées m'ont permis de prendre une décision en toute connaissance de cause concernant le stockage, la réutilisation et l'expédition de mes échantillons de sang et d'urine. Si l'un des échantillons de sang ou d'urine que j'ai fournis pour ce projet de recherche est inutilisé ou reste lorsque le projet est terminé, je donne la permission de :

Sang

☐ Je donne la permission de stocker mon échantillon de sang.

☐ Je donne la permission pour que mon échantillon de sang sois expédie à l'Université Allemand de Bonn ou Munich ou aux instituts en Tanzanie et au Cameroun

☐ Je donne ma permission pour que mon échantillon de sang soit stocké et utiliser dans n'importe quel type de recherches future qui auront reçu l'approbation éthique du comité d'éthique responsable

ou

☐ Je donne ma permission pour que mon échantillon de sang soit stocké et utiliser dans des recherches futures sauf des recherches sur ________________ (nom du type de recherche)

Urine

☐ Je donne la permission de stocker mon échantillon d'urine.

☐ Je donne la permission d'expédie mon échantillon d'urine à l'Université Allemand de Bonn ou Munich ou aux instituts en Tanzanie et au Cameroun

☐ Je donne ma permission de stocker et d'utiliser mon échantillon d'urine dans n'importe quel type de recherches future qui auront reçu l'approbation éthique du comité d'éthique responsable

ou

☐ Je donne la permission pour que mon échantillon d'urine soit stocké et utiliser dans des recherches futures sauf des recherches sur ________________ (nom du type de recherche)

Salive

☐ Je donne la permission de stocker ma salive.

☐ Je donne la permission d'expédie ma salive à l'Université Allemand de Bonn ou Munich ou aux instituts en Tanzanie et au Cameroun

☐ Je donne ma permission de stocker et d'utiliser mon échantillon de salive dans n'importe quel type de recherches future qui auront reçu l'approbation éthique du comité d'éthique responsable

ou

☐ Je donne la permission pour que la salive sois stocker et utiliser dans des recherches future sauf des recherches sur ________________ (nom du type de recherche)

Je suis conscient que je peux retirer mon assentiment à tout moment et pour n'importe quelle raison sans pénalité ni perte d'avantages. En signant ce formulaire de consentement, je ne renonce à aucun droit légal, et l'investigateur (s) ou le sponsor ne sont pas dégagés de toute responsabilité qu'ils pourraient avoir.

J'ajoute ainsi ma signature / empreinte (empreinte du pouce droit) à ce formulaire de consentement éclairé, comme preuve de mon accord pour le stockage, la réutilisation et l'expédition de mes échantillons.

Date: |__|__| / |__|__| / |__|__|__|__|
(Jj / mm / aaaa)

Si besoin, empreinte du participant:

____
(Signature du participant)

____
(Nom du participant en majuscule)

Signature du Parent

Je certifie par la présente que j'étais présent lorsque le contenu du formulaire de consentement éclairé a été lu / interprété et expliqué à mon fils/ ma fille dans une langue qu'il/elle comprend (Pidgin, Anglais, Français) (*souligner si applicable*).

____

(Nom du participant en majuscule)

Il / elle semble avoir parfaitement compris le contenu du Formulaire de consentement éclairé avant d'apposer sa signature ou d'apposer sa marque (empreinte du pouce droit) sur ce Formulaire de consentement éclairé. En tant que représentant légal, j'accepte par la présente son consentement au stockage, à la réutilisation et à l'expédition des échantillons.

Date: |__|__| / |__|__| / |__|__|__|__|
(Jj / mm / aaaa)

Si besoin, empreinte du participant:

____
(Signature du parent)

____
(Nom du parent en majuscule)

TEMOIN (indépendant de l'équipe de recherche)

Je certifie par la présente que j'étais présent lorsque le contenu du formulaire de consentement éclairé a été lu / interprété et expliqué en langue (Pidgin, Anglais, Français) (*souligner si applicable*).

____

(Nom du participant en majuscule)

et son/sa représentant légal.

Il / elle et son/sa représentant légal semble avoir parfaitement compris le contenu du Formulaire de consentement éclairé avant d'apposer leurs signatures ou d'apposer leur marques (empreintes des pouces droit) sur ce Formulaire de consentement éclairé en ma présence comme preuve de leur consentement au stockage, réutilisation et d'expédition des échantillons.

Date: |__|__| / |__|__| / |__|__|__|__|
(Jj / mm / aaaa)

____
(Signature du témoin)

____
(Nom du témoin en majuscule)

Informateur (Enquêteur)

Je certifie par la présente que le contenu du formulaire de consentement éclairé a été interprété et expliqué par moi dans une langue qu'il/elle comprend (Pidgin, Anglais, Français) (*souligner si applicable*).

____

(Nom du participant en majuscule)

et son/sa représentant légal.

Il / elle et son/sa représentant légal semble avoir parfaitement compris le contenu du Formulaire de consentement éclairé avant d'apposer leur signatures ou d'apposer leur marques (empreintes des pouces droit) sur ce Formulaire de consentement éclairé en ma présence comme preuve de leur consentement au stockage, réutilisation et expédition des échantillons.

Date: |__|__| / |__|__| / |__|__|__|__|
(Jj / mm / aaaa)

____

(Signature de l'enquêteur)

____

(Nom de l'enquêteur en majuscule)

17.**CASE REPOR FORMS (CRFs) / Questionaire**

TAKeOFF

PodoLEDoxy

“Doxycycline for treatment of non-filarial lymphedema due to podoconiosis (PodoLE) - a randomized double blind placebo-controlled trial”

CASE REPORT FORM (CRF)

Indiv. No.: |__|__| - |__|__|__| - |__|__|__|
(country) - (Village code) - (consecutive number)

Attach participant`s barcode label here and on duplicate

☐ **Group A (LE stage 1 – 3)**

☐ **Group B (LE stage 4 – 6)**

Randomization code: |__|__|__|

Principal Investigators

Dr. Alexander Yaw Debrah
Faculty of Allied Health Sciences
Kwame Nkrumah University of Science and Technology (KNUST)
Kumasi, Ghana
Tel. No.: +233 20 934 1317
Fax No.: +233 3220 62017
Email: **yadebrah@yahoo.com**

Dr. Inge Kroidl
Department for Infectious Diseases & Tropical Medicine
Klinikum of the University of Munich, LMU
Leopoldstrasse 5
80802 Munich, Germany
Tel.: +49 89 2180 17637
Fax: +49 89 33 6038
Email: ikroidl@lrz.uni-muenchen.de

Dr. Upendo Mwingira
The National Institute of Medical Research (NIMR)
3 Barak Obama Drive
P.O.Box 9653
11101 Dar es Salaam, Tanzania
Tel. No.: +255 783 276 177
Fax No.: +255 22 2121376
Email: umwingira@yahoo.com

Prof. Dr.Achim Hoerauf
Institute for Medical Microbiology, Immunology and Parasitology (IMMIP)
University Hospital of Bonn
Sigmund-Freud-Straße 25
53127 Bonn, Germany
Tel. No.: +49 228 287 15673
Fax No.: +49 228 287 19573
Email: **achim.hoerauf@ukbonn.de**

**PodoLEDoxy**	Indiv. No.: |__|__|-|__|__|__|-|__|__|__|
Date of visit: |__|__| / |__|__| / |__|__|__|__| (dd/mm/yyyy)	**VISIT 1 SCREENING**

INFORMED CONSENTS/ ASSENTS

Patient was informed and gave consent/ assent for the clinical trial: ☐ yes ☐ no

Date of informed consent/ assent for clinical trial: |__|__| / |__|__| / |__|__|__|__| (dd/mm/yyyy)

Patient was informed and gave consent/ assent for biobanking: ☐ yes ☐ no

Date of informed consent/ assent for biobanking: |__|__| / |__|__| / |__|__|__|__| (dd/mm/yyyy)

Copies of patient information sheets and signed informed consents/assents given to patient: ☐ yes ☐ no

DEMOGRAPHIC DATA

Sex: ☐ female ☐ male

If "female": Pregnant: ☐ yes ☐ no

Breastfeeding: ☐ yes ☐ no

Age: |__|__| (years)

Weight: |__|__|__|.|__| (kg)

In endemic area since: |__|__| (years)

LYMPHEDEMA STAGING

Stage	Description	Right Leg	Left Leg
0	No abnormality	☐	☐
1	Swelling is reversible (goes away) overnight	☐	☐
2	Swelling is not reversible (doesn't go away)	☐	☐
3	Presence of shallow skin folds<br>(base of fold can be seen by straightening the ankle)	☐	☐
4	Presence of skin knobs	☐	☐
5	Presence of deep skin folds (base of fold can only be seen if edges are actively separated by hand)	☐	☐
6	Presence of "mossy lesions"<br>Warty looking epidermal skin lesions.	☐	☐
7	Unable to care for self or perform daily activities	☐	☐

HISTORY OF LYMPHEDEMA

First realization of leg swelling: |__|__| years or |__|__|__| months ago ☐ not known

Family members affected:

☐ Mother ☐Father ☐Sisters ☐Brothers ☐Daughters ☐Sons ☐None

☐ Other Family Members (specify:________________________________)

PodoLEDoxy

Indiv. No.: |__|__|-|__|__|__|-|__|__|__|

Date of visit: |__|__| / |__|__| / |__|__|__|__| (dd/mm/yyyy)

VISIT 1
SCREENING

HISTORY OF ACUTE DERMATOLYMPHANGIOADENITIS (ADLA)

History of ADLA attacks: ☐ yes ☐ no

If "yes":

Occurance of first ADLA attack: |__|__| years or |__|__|__| months ago ☐ not known

Last attack: |__|__| / |__|__|__|__| (mm/yyyy) Duration of last attack: |__|__|__| (days)

Fever during last attack: ☐ yes ☐ no Lymph node swelling during last attack: ☐ yes ☐ no

Peeling of limb after last attack: ☐ yes ☐ no Able to work during last attack: ☐ yes ☐ no

Cause of last attack: ☐ injury ☐ entry lesion ☐ other (specify:__________________) ☐ not known

Occupation (specify: ___________________________________)

Intensity of work: ☐ light ☐ heavy ☐ not working ☐ other (specify:______________________)

Number of attacks within the last year: |__|__|__| ☐ not known

Duration of attacks (average within the last year): |__|__|__| (days)

CONCOMITANT MEDICATION

Drug intake: ☐ yes ☐ no

*If "**yes**", please record all medications (prescription and non-prescription) currently being taken by patient and include any medication taken within the last 14 days on the "Concomitant Medication" form at the end of this CRF*

HISTORY OF TRIAL RELEVANT MEDICATION

MDA *(Ivermectin + Albendazole)*:

MDA ever taken: ☐ yes ☐ no

If " yes": Intake of MDA within the last year: ☐ yes ☐ no

No. of previous MDA rounds: |__|__| ☐ not known

Adverse events after MDA: ☐ yes ☐ no If "yes", specify ______________________

Doxycycline

Doxycycline ever taken in life: ☐ yes ☐ no ☐ not known

If "yes": Intake of Doxycycline within the last year: ☐ yes ☐ no

Adverse events after Doxycycline: ☐ yes ☐ no If "yes", specify ____________________

Family planning medication

If "female": Family planning medication: ☐ yes ☐ no

*If "**yes**", enter on the "Concomitant Medication" form at the end of this CRF*

**PodoLEDoxy**	Indiv. No.: |__|__|-|__|__|__|-|__|__|__|
Date of visit: |__|__| / |__|__| / |__|__|__|__| (dd/mm/yyyy)	**VISIT 1 SCREENING**

MEDICAL HISTORY

	Describe:	How long ago?		Duration of illness?		If ongoing
☐ Cardiovascular		|__|__|	☐ days ☐ weeks ☐ months	|__|__|	☐ days ☐ weeks ☐ months	☐ controlled ☐ uncontrolled
☐ Respiratory		|__|__|	☐ days ☐ weeks ☐ months	|__|__|	☐ days ☐ weeks ☐ months	☐ controlled ☐ uncontrolled
☐ Gastrointestinal		|__|__|	☐ days ☐ weeks ☐ months	|__|__|	☐ days ☐ weeks ☐ months	☐ controlled ☐ uncontrolled
☐ Hepatic		|__|__|	☐ days ☐ weeks ☐ months	|__|__|	☐ days ☐ weeks ☐ months	☐ controlled ☐ uncontrolled
☐ Neurological		|__|__|	☐ days ☐ weeks ☐ months	|__|__|	☐ days ☐ weeks ☐ months	☐ controlled ☐ uncontrolled
☐ Musculoskeletal		|__|__|	☐ days ☐ weeks ☐ months	|__|__|	☐ days ☐ weeks ☐ months	☐ controlled ☐ uncontrolled
☐ Skin-/Mucous Membrane		|__|__|	☐ days ☐ weeks ☐ months	|__|__|	☐ days ☐ weeks ☐ months	☐ controlled ☐ uncontrolled
☐ Endocrine Disorders		|__|__|	☐ days ☐ weeks ☐ months	|__|__|	☐ days ☐ weeks ☐ months	☐ controlled ☐ uncontrolled
☐ Psychological		|__|__|	☐ days ☐ weeks ☐ months	|__|__|	☐ days ☐ weeks ☐ months	☐ controlled ☐ uncontrolled
☐ Relevant Allergies		|__|__|	☐ days ☐ weeks ☐ months	|__|__|	☐ days ☐ weeks ☐ months	☐ controlled ☐ uncontrolled
☐ Surgical Procedure		|__|__|	☐ days ☐ weeks ☐ months	|__|__|	☐ days ☐ weeks ☐ months	☐ controlled ☐ uncontrolled
☐ Other: ____________________		|__|__|	☐ days ☐ weeks ☐ months	|__|__|	☐ days ☐ weeks ☐ months	☐ controlled ☐ uncontrolled
☐ Other: ____________________		|__|__|	☐ days ☐ weeks ☐ months	|__|__|	☐ days ☐ weeks ☐ months	☐ controlled ☐ uncontrolled
☐ Other: ____________________		|__|__|	☐ days ☐ weeks ☐ months	|__|__|	☐ days ☐ weeks ☐ months	☐ controlled ☐ uncontrolled

VITAL SIGNS

Blood pressure: |__|__|__| / |__|__|__| mmHg Hypertension: ☐ yes ☐ no
systolic diastolic

If "hypertensive": Known hypertension: ☐ yes ☐ no Hypertensive medication: ☐ yes ☐ no

*If "**yes**", enter on the "Concomitant Medication" form at the end of this CRF*

Pulse rate: |__|__|__| bpm Temperature: |__|__|.|__|°C

PHYSICAL EXAMINATION

Indicate current medical conditions by ticking appropriate boxes. If abnormality, specify condition(s):

	Normal	Abnormal	Condition
Ear, nose, throat	☐	☐	
Eyes	☐	☐	
Cardiovascular	☐	☐	
Chest and lungs	☐	☐	
Abdomen	☐	☐	
Nervous system	☐	☐	
Musculo-skeletal	☐	☐	
Skin and mucosa	☐	☐	
Peripheral vascular	☐	☐	
Other	☐	☐	
Other	☐	☐	
Other	☐	☐	

LABORATORY ASSESSMENT (BLOOD)

Blood sample taken: ☐ yes ☐ no

If "no", state reason: ☐ refused ☐ absent ☐ other (specify: ________________________________)

If "yes": Sampling date: |__|__| / |__|__| / |__|__|__|__| Sampling time: |__|__| : |__|__| ☐ AM ☐ PM

(dd/mm/yyyy) (h:min)

Please attach corresponding barcode label(s) on the "Laboratory documentation sheet".
Please enter results on the „Laboratory assessment" form at the end of this CRF.

**PodoLEDoxy**	Indiv. No.: |__|__|-|__|__|__|-|__|__|__|
Date of visit: |__|__| / |__|__| / |__|__|__|__| (dd/mm/yyyy)	**VISIT 1 SCREENING**

**URINE SAMPLING**
Urine sample taken: ☐ yes ☐ no<br>If "no", state reason: ☐ refused ☐ absent ☐ other (specify: ________________________________)<br>If "yes": Sampling date: |__|__| / |__|__| / |__|__|__|__| Sampling time: |__|__| : |__|__| ☐ AM ☐ PM<br>(dd/mm/yyyy) (h:min)<br>*Please attach corresponding barcode label(s) on the "Laboratory documentation sheet".*

**PodoLEDoxy**	Indiv. No.: |__|__|-|__|__|__|-|__|__|__|
Date of visit: |__|__| / |__|__| / |__|__|__|__| (dd/mm/yyyy)	**VISIT 1 SCREENING**

SALIVA SAMPLING

Saliva sample taken: ☐ yes ☐ no

If "no", state reason: ☐ refused ☐ absent ☐ other (specify: _____________________________)

If "yes": Sampling date: \|__\|__\| / \|__\|__\| / \|__\|__\|__\|__\| Sampling time: \|__\|__\| : \|__\|__\| ☐ AM ☐ PM

(dd/mm/yyyy) (h:min)

Please attach corresponding barcode label(s) on the "Laboratory documentation sheet".

PREGNANCY TEST

Sampling date: \|__\|__\| / \|__\|__\| / \|__\|__\|__\|__\| Sampling time: \|__\|__\| : \|__\|__\| ☐ AM ☐ PM

(dd/mm/yyyy) (h:min)

Date analyzed: \|__\|__\| / \|__\|__\| / \|__\|__\|__\|__\| Time analyzed: \|__\|__\| : \|__\|__\| ☐ AM ☐ PM

(dd/mm/yyyy) (h:min)

Result: ☐ neg. ☐ pos. ☐ not applicable (☐ ≥ 55 years, ☐ surgically sterilized)

REMARKS – SCREENING – VISIT 1

**PodoLEDoxy**	Indiv. No.: |__|__|-|__|__|__|-|__|__|__|
Date of visit: |__|__| / |__|__| / |__|__|__|__| (dd/mm/yyyy)	**VISIT 1 SCREENING**

INCLUSION CRITERIA

(tick one box for each question)	**yes**	**no**
Lymphedema of the leg of grade 1-6 measured on a 7 point scale (according to Dreyer)?	☐	☐
Is the age of the patient between 14 and 65 years (inclusive)?	☐	☐
Man or non-pregnant woman. If woman of childbearing-potential, she must use an approved, effective method of contraception (including abstinence) before, during and for at least 2 weeks after the completion of the active intervention with doxycycline or placebo	☐	☐
If the patient is female, does she have a negative pregnancy test? ☐ not applicable	☐	☐
Is the weight of the patient ≥40 kg?	☐	☐
Has the patient been resident in a LF endemic area for ≥2 years?	☐	☐
Is the patient able and willing to sign or thumbprint an informed consent or to provide assent?	☐	☐
Is the patient able to use standardised methods of hygiene and apply it?	☐	☐

If any **INCLUSION CRITERIA** question is answered **NO**, this patient must **NOT** enter this study.

EXCLUSION CRITERIA

(tick one box for each question)	**yes**	**no**
Does the patient have no lymphedema or lymphedema stage 7?	☐	☐
Is the patient aged < 14 years or >65 years?	☐	☐
Is the body weight less than 40kg?	☐	☐
Is the patient pregnant or breast-feeding? ☐ not applicable (male)	☐	☐
Is the patient unwilling to use an agreed method of contraception?	☐	☐
Is there evidence of hepatic or renal dysfunction or a disease of the central nervous system (CNS)?	☐	☐
Evidence of severe comorbidity other than filariasis	☐	☐
Does the patient have a history of alcohol or drug abuse?	☐	☐
Does the patient have a history of allergy to doxycycline or other tetracyclines?	☐	☐
Does the patient have any any significant condition (including medical and psychological/ psychiatric disorder) which in the opinion of the study investigator might interfere with the conduct of the study?	☐	☐
Did the patient have photosensitivity reactions in the past after taking drugs?	☐	☐

**PodoLEDoxy**	Indiv. No.: |__|__|-|__|__|__|-|__|__|__|
Date of visit: |__|__| / |__|__| / |__|__|__|__| (dd/mm/yyyy)	**VISIT 1 SCREENING**

Does the patient take diuretics, sulfonylurea or coumarin on a regular basis?	☐	☐
Does the patient currently take antacids containing aluminium, magnesium or sucralfate and is not able to doscontinue?	☐	☐
Does the patient currently take antibiotics other than doxycycline and is not able to discontinue?	☐	☐

EXCLUSION CRITERIA

(tick one box for each question)	**yes**	**no**
**Does the patient have any of the following laboratory results at the screening visit prior the first dose of treatment?**:		
Haemoglobin < 8 gm/dL	☐	☐
Neutrophil count <2 000/mm^3	☐	☐
Platelet count <100 000/mm^3	☐	☐
Creatinine > 2 x upper limit of normal (ULN)	☐	☐
AST (GOT) > 2 x upper limit of normal (ULN)	☐	☐
ALT (GPT) > 2 x upper limit of normal (ULN)	☐	☐
γ-GT > 2 x upper limit of normal (ULN)	☐	☐
Positive urine pregnancy test ☐ not applicable	☐	☐

If any **EXCLUSION CRITERIA** question is answered **YES**, this patient must **NOT** enter this study.

Is the patient suitable for enrolment into the study? ☐ yes ☐ no

If "no", state the reason(s):____

VISIT 1 – SCREENING - INVESTIGATOR'S STATEMENT

I confirm that I have carefully examined all entries on the Visit 1 – Screening - Case Report Form pages for this subject. All information entered by myself or my colleagues is, to the best of my knowledge, correct as of the date below.

Date: |__|__| / |__|__| / |__|__|__|__|

(dd/mm/yyyy)

____

(Investigator`s signature)

____

(Investigator`s name print)

**PodoLEDoxy**	Indiv. No.: |__|__|-|__|__|__|-|__|__|__| Randomization Code: |__|__|__|
Date of visit: |__|__| / |__|__| / |__|__|__|__| (dd/mm/yyyy)	**VISIT 2<br>BASELINE**

CONCOMITANT MEDICATION

Change of medication since last visit? ☐ yes ☐ no

*If "**yes**", please enter on the „Concomitant Medication" form at the end of this CRF.*

VITAL SIGNS

Blood pressure: |__|__|__| / |__|__|__| mmHg Hypertension: ☐ yes ☐ no
systolic diastolic

If "hypertensive": Known hypertension: ☐ yes ☐ no Hypertensive medication: ☐ yes ☐ no

*If "**yes**", enter on the "Concomitant Medication" form at the end of this CRF*

Pulse rate: |__|__|__| bpm Temperature: |__|__|.|__|°C Weight: |__|__|__|.|__| kg

PHYSICAL EXAMINATION

Indicate current medical conditions by ticking appropriate boxes. If abnormality, specify condition(s):

	Normal	Abnormal	Condition
Ear, nose, throat	☐	☐	
Eyes	☐	☐	
Cardiovascular	☐	☐	
Chest and lungs	☐	☐	
Abdomen	☐	☐	
Nervous system	☐	☐	
Musculo-skeletal	☐	☐	
Skin and mucosa	☐	☐	
Peripheral vascular	☐	☐	
Other	☐	☐	
Other	☐	☐	
Other	☐	☐	

CLINICAL PHOTOGRAPHS

Clinical photographs taken: ☐ yes ☐ no (explain:________________________________)

If "yes", Photograph References/No.:____

**PodoLEDoxy**	Indiv. No.: |__|__|-|__|__|__|-|__|__|__| Randomization Code: |__|__|__|
Date of visit: |__|__| / |__|__| / |__|__|__|__| (dd/mm/yyyy)	**VISIT 2 BASELINE**

LYMPHEDEMA STAGING

Stage	Description	Right Leg	Left Leg
0	No abnormality	☐	☐
1	Swelling is reversible (goes away) overnight	☐	☐
2	Swelling is not reversible (doesn't go away)	☐	☐
3	Presence of shallow skin folds<br>(base of fold can be seen by straightening the ankle)	☐	☐
4	Presence of skin knobs	☐	☐
5	Presence of deep skin folds (base of fold can only be seen if edges are actively separated by hand)	☐	☐
6	Presence of "mossy lesions"<br>Warty looking epidermal skin lesions.	☐	☐
7	Unable to care for self or perform daily activities	☐	☐

ADLA QUESTIONNAIRE

ADLA attacks since last visit: ☐ yes ☐ no

If "yes": Start date of last attack: |__|__| / |__|__|__|__| (mm/yyyy) Ongoing?: ☐ yes ☐ no

Duration of last attack: |__|__|__| (days) Number of attacks since last visit: |__|__|__|

Fever during last attack: ☐ yes ☐ no Lymphnode swelling during last attack: ☐ yes ☐ no

Peeling of limb after last attack: ☐ yes ☐ no Able to work during last attack: ☐ yes ☐ no

Occupation (specify:_____________________________________)

HYGIENE STATUS

Hygiene assessment done: ☐ yes ☐ no

If "yes", date: |__|__| / |__|__| / |__|__|__|__| (dd/mm/yyyy)

LYMPHEDEMA MANAGEMENT TRAINING

Training has been given: ☐ yes ☐ no

If "yes", date: |__|__| / |__|__| / |__|__|__|__| (dd/mm/yyyy)

CIRCUMFERENCE MEASUREMENT OF LYMPHEDEMA –TAPE

Examiner: ____

Date: |__|__| / |__|__| / |__|__|__|__| (dd/mm/yyyy) Time of measurement**:** |__|__|:|__|__| (h:min) ☐ AM ☐ PM

Main activity in the previous two hours before measurement: ☐ sitting ☐ walking ☐ standing ☐ farming ☐ fishing ☐ cooking ☐ driving ☐ child care ☐ other:___________________________

	**Circumferences of left leg**		**Circumferences of right leg**	
	1st measurement	2nd measurement	1st measurement	2nd measurement
A (10cm from tip of 1st toe)	|__|__|.|__| cm	|__|__|.|__| cm	|__|__|.|__| cm	|__|__|.|__| cm
B (12cm)	|__|__|.|__| cm	|__|__|.|__| cm	|__|__|.|__| cm	|__|__|.|__| cm
C (20 cm)	|__|__|.|__| cm	|__|__|.|__| cm	|__|__|.|__| cm	|__|__|.|__| cm
D (30cm)	|__|__|.|__| cm	|__|__|.|__| cm	|__|__|.|__| cm	|__|__|.|__| cm

CIRCUMFERENCE MEASUREMENT OF LYMPHEDEMA –LYMPHATECH® SCANNER

Examiner: ____

Date: |__|__| / |__|__| / |__|__|__|__| (dd/mm/yyyy) Time of measurement**:** |__|__|:|__|__| (h:min) ☐ AM ☐ PM

Main activity in the previous two hours before measurement: ☐ sitting ☐ walking ☐ standing ☐ farming ☐ fishing ☐ cooking ☐ driving ☐ child care ☐ other:___________________________

	**Circumferences of left leg**		**Circumferences of right leg**	
	1st measurement	2nd measurement	1st measurement	2nd measurement
A (12 cm)	|__|__|.|__| cm	|__|__|.|__| cm	|__|__|.|__| cm	|__|__|.|__| cm
B (20 cm)	|__|__|.|__| cm	|__|__|.|__| cm	|__|__|.|__| cm	|__|__|.|__| cm
C (30 cm)	|__|__|.|__| cm	|__|__|.|__| cm	|__|__|.|__| cm	|__|__|.|__| cm

VOLUME OF LYMPHEDEMA –LYMPHATECH® SCANNER

Examiner: ____

Date: |__|__| / |__|__| / |__|__|__|__| (dd/mm/yyyy) Time of measurement**:** |__|__|:|__|__| (h:min) ☐ AM ☐ PM

Main activity in the previous two hours before measurement: ☐ sitting ☐ walking ☐ standing

☐ farming ☐ fishing ☐ cooking ☐ driving ☐ child care ☐ other:___________________________

	1st measurement	2nd measurement
Volume of left leg	|__|__|__|__|__|__| ml	|__|__|__|__|__|__| ml
Volume of right leg	|__|__|__|__|__|__| ml	|__|__|__|__|__|__| ml

LymphaTech Photograph References/No.:____

ULTRASOUND ASSESSMENT OF SKIN THICKNESS AT BOTH ANKLES

Examiner: ____

Date: |__|__| / |__|__| / |__|__|__|__| (dd/mm/yyyy) Time of measurement**:** |__|__| : |__|__| (h:min) ☐ AM ☐ PM

Main activity in the previous two hours before measurement: ☐ sitting ☐ walking ☐ standing ☐ farming ☐ fishing ☐ cooking ☐ driving ☐ child care ☐ other:___________________________

Presence of acute conditions:

Pregnancy: ☐ yes ☐ no

Febrile illness: ☐ yes ☐ no

Heart condition requiring diuretics: ☐ yes ☐ no

Adenolymphangitis attack: ☐ yes ☐ no , if "yes": ☐ left leg ☐ right leg

Recent leg trauma: ☐ yes ☐ no , if "yes": ☐ left leg ☐ right leg:

Other: ☐ yes ☐ no, if "yes", describe:______________________________

Has the patient been bed-bound more than one day within the past week? ☐ yes ☐ no

If "yes", describe:____

Are open wounds present? ☐ yes ☐ no

If "yes", ☐ left leg/acute ☐ left leg/chronic ☐ right leg/acute ☐ right leg/chronic

Even when open wounds are present, ultrasound should be done as long as it does not require placing the ultrasound probe within the field of the wound. If measurement cannot be done without placing the probe in the wound, skip that location and write "wound" in the notes section below.

Skin measurement

	1st measurement	2nd measurement	Notes
Left lateral malleolus	|__|.|__|__| cm	|__|.|__|__| cm	
Left medial malleolus	|__|.|__|__| cm	|__|.|__|__| cm	

**PodoLEDoxy**	Indiv. No.: |__|__|-|__|__|__|-|__|__|__| Randomization Code: |__|__|__|

Date of visit: |__|__| / |__|__| / |__|__|__|__| (dd/mm/yyyy)	**VISIT 2<br>BASELINE**

Right lateral malleolus	|__|.|__|__| cm	|__|.|__|__| cm	
Right medial malleolus	|__|.|__|__| cm	|__|.|__|__| cm	

Ultrasound References/No.:____ Photograph

QUALITY OF LIFE QUESTIONNAIRE

QoL questionnaire carried out: ☐ yes ☐ no

If "yes", date: |__|__| / |__|__| / |__|__|__|__| (dd/mm/yyyy)

PREGNANCY TEST (URINE)

Sampling date: |__|__| / |__|__| / |__|__|__|__| (dd/mm/yyyy) Sampling time: |__|__| : |__|__| ☐ AM ☐ PM (h:min)

Date analyzed: |__|__| / |__|__| / |__|__|__|__| (dd/mm/yyyy) Time analyzed: |__|__| : |__|__| ☐ AM ☐ PM (h:min)

Result: ☐ neg. ☐ pos. ☐ not applicable (☐ ≥ 55 years, ☐ surgically sterilized)

Contraceptive advice given ☐ yes ☐ no if "yes", by:________________________

REMARKS – BASELINE – VISIT 2

VISIT 2 - BASELINE - INVESTIGATOR'S STATEMENT

I confirm that I have carefully examined all entries on the Visit 2 – Baseline Case Report Form pages for this subject. All information entered by myself or my colleagues is, to the best of my knowledge, correct as of the date below.

Date: |__|__| / |__|__| / |__|__|__|__| (dd/mm/yyyy)

____
(Investigator`s signature)

____
(Investigator`s name print)

RANDOMIZATION

Patient belongs to ☐ **Group A: Stage 1-3**

☐ **Group B: Stage 4-6**

Has the consent form for the trial been signed by the patient, witness and investigator: ☐ yes ☐ no

Does the patient understand that the study will last 2 years after treatment start: ☐ yes ☐ no

Does he/she understand that treatment has to be supervised for length of the treatment phase of the study:

☐ yes ☐ no

Does the patient meet all entry criteria in the study?: ☐ yes ☐ no

If **"no",** state reason(s): ____

If all questions answered with "**yes**", allocate randomization code.

Randomization Code: |__|__|__|__|__|

Record this number on the top of each following page of this case report form

Stick the label from medication package below (original CRF and on duplicate).

label from medication package

Randomized by: ______________________________ Date: |__|__| / |__|__| / |__|__|__|__|
(Investigator`s name print) (dd/mm/yyyy)

____
(Investigator`s signature)

**PodoLEDoxy**	Indiv. No.: |__|__|-|__|__|__|-|__|__|__| Randomization Code: |__|__|__|
Date of visit: |__|__| / |__|__| / |__|__|__|__| (dd/mm/yyyy)	**VISIT 3 - TREATMENT Day 1**

PREGNANCY TEST (URINE) BEFORE 1ST TREATMENT
(only necessary if treatment start is not on the same day or one day after visit 2)

Sampling date: |__|__| / |__|__| / |__|__|__|__| (dd/mm/yyyy) Sampling time: |__|__| : |__|__| ☐ AM ☐ PM (h:min)

Date analyzed: |__|__| / |__|__| / |__|__|__|__| (dd/mm/yyyy) Time analyzed: |__|__| : |__|__| ☐ AM ☐ PM (h:min)

Result: ☐ neg. ☐ pos. ☐ not applicable (☐ ≥ 55 years, ☐ surgically sterilized)

Contraceptive advice given ☐ yes ☐ no if "yes", by:_______________________

ADLA QUESTIONNAIRE *(only necessary if treatment start is not on the same as visit 2)*

ADLA attack since last visit: ☐ yes ☐ no Ongoing?: ☐ yes ☐ no

If "yes": Fever during attack: ☐ yes ☐ no Lymphnode swelling during attack: ☐ yes ☐ no

Peeling of limb after attack: ☐ yes ☐ no Able to work during attack: ☐ yes ☐ no

CONCOMITANT MEDICATION *(only necessary if treatment start is not on the same as visit 2)*

Change of medication since last visit? ☐ yes ☐ no

*If "**yes**", please enter on the "Concomitant Medication" form at the end of this CRF.*

INDIVIDUAL TREATMENT

Study drugs taken: ☐ yes ☐ no If "yes": **Treatment No.:** |__|__|

If "no", state reason: ☐ medical ☐ other (describe:____)

PROBLEMS/ REMARKS

☐ yes ☐ no

If "yes", describe:____

**PodoLEDoxy**	Indiv. No.: |__|__|-|__|__|__|-|__|__|__| Randomization Code: |__|__|__|
Date of visit: |__|__| / |__|__| / |__|__|__|__| (dd/mm/yyyy)	**VISIT 3 - TREATMENT**<br>**Day 2**

PRESENCE

Participant present for visit: ☐ yes ☐ no

If "no", participant: ☐ traveled with drugs ☐ is absent but drugs were given to health worker/family member/neighbor and drug intake was confirmed

☐ is absent (state reason:____)

ADLA QUESTIONNAIRE *(only to be filled in if participant present for visit)*

ADLA attack since last visit: ☐ yes ☐ no Ongoing?: ☐ yes ☐ no

If "yes": Fever during attack: ☐ yes ☐ no Lymphnode swelling during attack: ☐ yes ☐ no

Peeling of limb after attack: ☐ yes ☐ no Able to work during attack: ☐ yes ☐ no

ADVERSE EVENTS *(only to be filled in if participant present for visit)*

Appearance of AEs / SAEs since last drug intake? ☐ yes ☐ no

*If "**yes**", please enter on the "Adverse Events" form at the end of this CRF.*

CONCOMITANT MEDICATION *(only to be filled in if participant present for visit)*

Change of medication since last visit? ☐ yes ☐ no

*If "**yes**", please enter on the "Concomitant Medication" form at the end of this CRF.*

INDIVIDUAL TREATMENT

Study drugs taken: ☐ yes ☐ no If "yes": **Treatment No.:** |__|__|

If "no", state reason: ☐ medical ☐ other (describe:____)

PROBLEMS/ REMARKS

☐ yes ☐ no

If "yes", describe:____

**PodoLEDoxy**	Indiv. No.: |__|__|-|__|__|__|-|__|__|__| Randomization Code: |__|__|__|
Date of visit: |__|__| / |__|__| / |__|__|__|__| (dd/mm/yyyy)	**VISIT 3 - TREATMENT**<br>**Day 3**

PRESENCE

Participant present for visit: ☐ yes ☐ no

If "no", participant: ☐ traveled with drugs ☐ is absent but drugs were given to health worker/family member/neighbor and drug intake was confirmed

☐ is absent (state reason:____)

ADLA QUESTIONNAIRE *(only to be filled in if participant present for visit)*

ADLA attack since last visit: ☐ yes ☐ no Ongoing?: ☐ yes ☐ no

If "yes": Fever during attack: ☐ yes ☐ no Lymphnode swelling during attack: ☐ yes ☐ no

Peeling of limb after attack: ☐ yes ☐ no Able to work during attack: ☐ yes ☐ no

ADVERSE EVENTS *(only to be filled in if participant present for visit)*

Appearance of AEs / SAEs since last drug intake? ☐ yes ☐ no

*If "**yes**", please enter on the "Adverse Events" form at the end of this CRF.*

CONCOMITANT MEDICATION *(only to be filled in if participant present for visit)*

Change of medication since last visit? ☐ yes ☐ no

*If "**yes**", please enter on the "Concomitant Medication" form at the end of this CRF.*

INDIVIDUAL TREATMENT

Study drugs taken: ☐ yes ☐ no If "yes": **Treatment No.:** |__|__|

If "no", state reason: ☐ medical ☐ other (describe:____)

PROBLEMS/ REMARKS

☐ yes ☐ no

If "yes", describe:____

**PodoLEDoxy**	Indiv. No.: |__|__|-|__|__|__|-|__|__|__| Randomization Code: |__|__|__|
Date of visit: |__|__| / |__|__| / |__|__|__|__| (dd/mm/yyyy)	**VISIT 3 - TREATMENT**<br>**Day 4**

PRESENCE

Participant present for visit: ☐ yes ☐ no

If "no", participant: ☐ traveled with drugs ☐ is absent but drugs were given to health worker/family member/neighbor and drug intake was confirmed

☐ is absent (state reason:____)

ADLA QUESTIONNAIRE *(only to be filled in if participant present for visit)*

ADLA attack since last visit: ☐ yes ☐ no Ongoing?: ☐ yes ☐ no

If "yes": Fever during attack: ☐ yes ☐ no Lymphnode swelling during attack: ☐ yes ☐ no

Peeling of limb after attack: ☐ yes ☐ no Able to work during attack: ☐ yes ☐ no

ADVERSE EVENTS *(only to be filled in if participant present for visit)*

Appearance of AEs / SAEs since last drug intake? ☐ yes ☐ no

*If "**yes**", please enter on the "Adverse Events" form at the end of this CRF.*

CONCOMITANT MEDICATION *(only to be filled in if participant present for visit)*

Change of medication since last visit? ☐ yes ☐ no

*If "**yes**", please enter on the "Concomitant Medication" form at the end of this CRF.*

INDIVIDUAL TREATMENT

Study drugs taken: ☐ yes ☐ no If "yes": **Treatment No.:** |__|__|

If "no", state reason: ☐ medical ☐ other (describe:____)

PROBLEMS/ REMARKS

☐ yes ☐ no

If "yes", describe:____

**PodoLEDoxy**	Indiv. No.: |__|__|-|__|__|__|-|__|__|__| Randomization Code: |__|__|__|
Date of visit: |__|__| / |__|__| / |__|__|__|__| (dd/mm/yyyy)	**VISIT 3 - TREATMENT**<br>**Day 5**

PRESENCE

Participant present for visit: ☐ yes ☐ no

If "no", participant: ☐ traveled with drugs ☐ is absent but drugs were given to health worker/family member/neighbor and drug intake was confirmed

☐ is absent (state reason:____)

ADLA QUESTIONNAIRE *(only to be filled in if participant present for visit)*

ADLA attack since last visit: ☐ yes ☐ no Ongoing?: ☐ yes ☐ no

If "yes": Fever during attack: ☐ yes ☐ no Lymphnode swelling during attack: ☐ yes ☐ no

Peeling of limb after attack: ☐ yes ☐ no Able to work during attack: ☐ yes ☐ no

ADVERSE EVENTS *(only to be filled in if participant present for visit)*

Appearance of AEs / SAEs since last drug intake? ☐ yes ☐ no

*If "**yes**", please enter on the "Adverse Events" form at the end of this CRF.*

CONCOMITANT MEDICATION *(only to be filled in if participant present for visit)*

Change of medication since last visit? ☐ yes ☐ no

*If "**yes**", please enter on the "Concomitant Medication" form at the end of this CRF.*

INDIVIDUAL TREATMENT

Study drugs taken: ☐ yes ☐ no If "yes": **Treatment No.:** |__|__|

If "no", state reason: ☐ medical ☐ other (describe:____)

PROBLEMS/ REMARKS

☐ yes ☐ no

If "yes", describe:____

**LEDoxy**	Indiv. No.: |__|__|-|__|__|__|-|__|__|__| Randomization Code: |__|__|__|
Date of visit: |__|__| / |__|__| / |__|__|__|__| (dd/mm/yyyy)	**VISIT 3 - TREATMENT**<br>**Day 6**

PRESENCE

Participant present for visit: ☐ yes ☐ no

If "no", participant: ☐ traveled with drugs ☐ is absent but drugs were given to health worker/family member/neighbor and drug intake was confirmed

☐ is absent (state reason:____)

ADLA QUESTIONNAIRE *(only to be filled in if participant present for visit)*

ADLA attack since last visit: ☐ yes ☐ no Ongoing?: ☐ yes ☐ no

If "yes": Fever during attack: ☐ yes ☐ no Lymphnode swelling during attack: ☐ yes ☐ no

Peeling of limb after attack: ☐ yes ☐ no Able to work during attack: ☐ yes ☐ no

ADVERSE EVENTS *(only to be filled in if participant present for visit)*

Appearance of AEs / SAEs since last drug intake? ☐ yes ☐ no

*If "**yes**", please enter on the "Adverse Events" form at the end of this CRF.*

CONCOMITANT MEDICATION *(only to be filled in if participant present for visit)*

Change of medication since last visit? ☐ yes ☐ no

*If "**yes**", please enter on the "Concomitant Medication" form at the end of this CRF.*

INDIVIDUAL TREATMENT

Study drugs taken: ☐ yes ☐ no If "yes": **Treatment No.:** \|__\|__\|

If "no", state reason: ☐ medical ☐ other (describe:____)

PROBLEMS/ REMARKS

☐ yes ☐ no

If "yes", describe:____

# LEDoxy	Indiv. No.: |__|__|-|__|__|__|-|__|__|__| Randomization Code: |__|__|__|

Date of visit: |__|__| / |__|__| / |__|__|__|__| (dd/mm/yyyy)	**VISIT 3 - TREATMENT**<br>**Day 7**

PRESENCE

Participant present for visit: ☐ yes ☐ no

If "no", participant: ☐ traveled with drugs ☐ is absent but drugs were given to health worker/family member/neighbor and drug intake was confirmed

☐ is absent (state reason:____)

ADLA QUESTIONNAIRE *(only to be filled in if participant present for visit)*

ADLA attack since last visit: ☐ yes ☐ no Ongoing?: ☐ yes ☐ no

If "yes": Fever during attack: ☐ yes ☐ no Lymphnode swelling during attack: ☐ yes ☐ no

Peeling of limb after attack: ☐ yes ☐ no Able to work during attack: ☐ yes ☐ no

ADVERSE EVENTS *(only to be filled in if participant present for visit)*

Appearance of AEs / SAEs since last drug intake? ☐ yes ☐ no

*If "**yes**", please enter on the "Adverse Events" form at the end of this CRF.*

CONCOMITANT MEDICATION *(only to be filled in if participant present for visit)*

Change of medication since last visit? ☐ yes ☐ no

*If "**yes**", please enter on the "Concomitant Medication" form at the end of this CRF.*

INDIVIDUAL TREATMENT

Study drugs taken: ☐ yes ☐ no If "yes": **Treatment No.:** |__|__|

If "no", state reason: ☐ medical ☐ other (describe:____)

PROBLEMS/ REMARKS

☐ yes ☐ no

If "yes", describe:____

**LEDoxy**	Indiv. No.: |__|__|-|__|__|__|-|__|__|__| Randomization Code: |__|__|__|
Date of visit: |__|__| / |__|__| / |__|__|__|__| (dd/mm/yyyy)	**VISIT 3 - TREATMENT**<br>**Day 8**

PRESENCE

Participant present for visit: ☐ yes ☐ no

If "no", participant: ☐ traveled with drugs ☐ is absent but drugs were given to health worker/family member/neighbor and drug intake was confirmed

☐ is absent (state reason:____)

ADLA QUESTIONNAIRE *(only to be filled in if participant present for visit)*

ADLA attack since last visit: ☐ yes ☐ no Ongoing?: ☐ yes ☐ no

If "yes": Fever during attack: ☐ yes ☐ no Lymphnode swelling during attack: ☐ yes ☐ no

Peeling of limb after attack: ☐ yes ☐ no Able to work during attack: ☐ yes ☐ no

ADVERSE EVENTS *(only to be filled in if participant present for visit)*

Appearance of AEs / SAEs since last drug intake? ☐ yes ☐ no

*If "**yes**", please enter on the "Adverse Events" form at the end of this CRF.*

CONCOMITANT MEDICATION *(only to be filled in if participant present for visit)*

Change of medication since last visit? ☐ yes ☐ no

*If "**yes**", please enter on the "Concomitant Medication" form at the end of this CRF.*

INDIVIDUAL TREATMENT

Study drugs taken: ☐ yes ☐ no If "yes": **Treatment No.:** |__|__|

If "no", state reason: ☐ medical ☐ other (describe:____)

PROBLEMS/ REMARKS

☐ yes ☐ no

If "yes", describe:____

**LEDoxy**	Indiv. No.: |__|__|-|__|__|__|-|__|__|__| Randomization Code: |__|__|__|
Date of visit: |__|__| / |__|__| / |__|__|__|__| (dd/mm/yyyy)	**VISIT 3 - TREATMENT**<br>**Day 9**

PRESENCE

Participant present for visit: ☐ yes ☐ no

If "no", participant: ☐ traveled with drugs ☐ is absent but drugs were given to health worker/family member/neighbor and drug intake was confirmed

☐ is absent (state reason:____)

ADLA QUESTIONNAIRE *(only to be filled in if participant present for visit)*

ADLA attack since last visit: ☐ yes ☐ no   Ongoing?: ☐ yes ☐ no

If "yes": Fever during attack: ☐ yes ☐ no   Lymphnode swelling during attack: ☐ yes ☐ no

Peeling of limb after attack: ☐ yes ☐ no   Able to work during attack: ☐ yes ☐ no

ADVERSE EVENTS *(only to be filled in if participant present for visit)*

Appearance of AEs / SAEs since last drug intake? ☐ yes ☐ no

*If "**yes**", please enter on the "Adverse Events" form at the end of this CRF.*

CONCOMITANT MEDICATION *(only to be filled in if participant present for visit)*

Change of medication since last visit? ☐ yes ☐ no

*If "**yes**", please enter on the "Concomitant Medication" form at the end of this CRF.*

INDIVIDUAL TREATMENT

Study drugs taken: ☐ yes ☐ no   If "yes": **Treatment No.:** |__|__|

If "no", state reason: ☐ medical ☐ other (describe:____)

PROBLEMS/ REMARKS

☐ yes ☐ no

If "yes", describe:____

**LEDoxy**	Indiv. No.: |__|__|-|__|__|__|-|__|__|__| Randomization Code: |__|__|__|
Date of visit: |__|__| / |__|__| / |__|__|__|__| (dd/mm/yyyy)	**VISIT 3 - TREATMENT**<br>**Day 10**

PRESENCE

Participant present for visit: ☐ yes ☐ no

If "no", participant: ☐ traveled with drugs ☐ is absent but drugs were given to health worker/family member/neighbor and drug intake was confirmed

☐ is absent (state reason:____)

ADLA QUESTIONNAIRE *(only to be filled in if participant present for visit)*

ADLA attack since last visit: ☐ yes ☐ no Ongoing?: ☐ yes ☐ no

If "yes": Fever during attack: ☐ yes ☐ no Lymphnode swelling during attack: ☐ yes ☐ no

Peeling of limb after attack: ☐ yes ☐ no Able to work during attack: ☐ yes ☐ no

ADVERSE EVENTS *(only to be filled in if participant present for visit)*

Appearance of AEs / SAEs since last drug intake? ☐ yes ☐ no

*If "**yes**", please enter on the "Adverse Events" form at the end of this CRF.*

CONCOMITANT MEDICATION *(only to be filled in if participant present for visit)*

Change of medication since last visit? ☐ yes ☐ no

*If "**yes**", please enter on the "Concomitant Medication" form at the end of this CRF.*

INDIVIDUAL TREATMENT

Study drugs taken: ☐ yes ☐ no If "yes": **Treatment No.:** |__|__|

If "no", state reason: ☐ medical ☐ other (describe:_______________________________________)

PROBLEMS/ REMARKS

☐ yes ☐ no

If "yes", describe:____

**LEDoxy**	Indiv. No.: |__|__|-|__|__|__|-|__|__|__| Randomization Code: |__|__|__|
Date of visit: |__|__| / |__|__| / |__|__|__|__| (dd/mm/yyyy)	**VISIT 3 - TREATMENT**<br>**Day 11**

PRESENCE

Participant present for visit: ☐ yes ☐ no

If "no", participant: ☐ traveled with drugs ☐ is absent but drugs were given to health worker/family member/neighbor and drug intake was confirmed

☐ is absent (state reason:____)

ADLA QUESTIONNAIRE *(only to be filled in if participant present for visit)*

ADLA attack since last visit: ☐ yes ☐ no Ongoing?: ☐ yes ☐ no

If "yes": Fever during attack: ☐ yes ☐ no Lymphnode swelling during attack: ☐ yes ☐ no

Peeling of limb after attack: ☐ yes ☐ no Able to work during attack: ☐ yes ☐ no

ADVERSE EVENTS *(only to be filled in if participant present for visit)*

Appearance of AEs / SAEs since last drug intake? ☐ yes ☐ no

*If "**yes**", please enter on the "Adverse Events" form at the end of this CRF.*

CONCOMITANT MEDICATION *(only to be filled in if participant present for visit)*

Change of medication since last visit? ☐ yes ☐ no

*If "**yes**", please enter on the "Concomitant Medication" form at the end of this CRF.*

INDIVIDUAL TREATMENT

Study drugs taken: ☐ yes ☐ no If "yes": **Treatment No.: |__|__|**

If "no", state reason: ☐ medical ☐ other (describe:____)

PROBLEMS/ REMARKS

☐ yes ☐ no

If "yes", describe:____

**LEDoxy**	Indiv. No.: |__|__|-|__|__|__|-|__|__|__| Randomization Code: |__|__|__|
Date of visit: |__|__| / |__|__| / |__|__|__|__| (dd/mm/yyyy)	**VISIT 3 - TREATMENT**<br>**Day 12**

PRESENCE

Participant present for visit: ☐ yes ☐ no

If "no", participant: ☐ traveled with drugs ☐ is absent but drugs were given to health worker/family member/neighbor and drug intake was confirmed

☐ is absent (state reason:________________________________)

ADLA QUESTIONNAIRE *(only to be filled in if participant present for visit)*

ADLA attack since last visit: ☐ yes ☐ no Ongoing?: ☐ yes ☐ no

If "yes": Fever during attack: ☐ yes ☐ no Lymphnode swelling during attack: ☐ yes ☐ no

Peeling of limb after attack: ☐ yes ☐ no Able to work during attack: ☐ yes ☐ no

ADVERSE EVENTS *(only to be filled in if participant present for visit)*

Appearance of AEs / SAEs since last drug intake? ☐ yes ☐ no

*If "**yes**", please enter on the "Adverse Events" form at the end of this CRF.*

CONCOMITANT MEDICATION *(only to be filled in if participant present for visit)*

Change of medication since last visit? ☐ yes ☐ no

*If "**yes**", please enter on the "Concomitant Medication" form at the end of this CRF.*

INDIVIDUAL TREATMENT

Study drugs taken: ☐ yes ☐ no If "yes": **Treatment No.:** |__|__|

If "no", state reason: ☐ medical ☐ other (describe:________________________________)

PROBLEMS/ REMARKS

☐ yes ☐ no

If "yes", describe:________________________________

**LEDoxy**	Indiv. No.: |__|__|-|__|__|__|-|__|__|__| Randomization Code: |__|__|__|
Date of visit: |__|__| / |__|__| / |__|__|__|__| (dd/mm/yyyy)	**VISIT 3 - TREATMENT**<br>**Day 13**

PRESENCE

Participant present for visit: ☐ yes ☐ no

If "no", participant: ☐ traveled with drugs ☐ is absent but drugs were given to health worker/family member/neighbor and drug intake was confirmed

☐ is absent (state reason:____)

ADLA QUESTIONNAIRE *(only to be filled in if participant present for visit)*

ADLA attack since last visit: ☐ yes ☐ no   Ongoing?: ☐ yes ☐ no

If "yes": Fever during attack: ☐ yes ☐ no   Lymphnode swelling during attack: ☐ yes ☐ no

Peeling of limb after attack: ☐ yes ☐ no   Able to work during attack: ☐ yes ☐ no

ADVERSE EVENTS *(only to be filled in if participant present for visit)*

Appearance of AEs / SAEs since last drug intake? ☐ yes ☐ no

*If "**yes**", please enter on the "Adverse Events" form at the end of this CRF.*

CONCOMITANT MEDICATION *(only to be filled in if participant present for visit)*

Change of medication since last visit? ☐ yes ☐ no

*If "**yes**", please enter on the "Concomitant Medication" form at the end of this CRF.*

INDIVIDUAL TREATMENT

Study drugs taken: ☐ yes ☐ no   If "yes": **Treatment No.:** |__|__|

If "no", state reason: ☐ medical ☐ other (describe:______________________________________)

PROBLEMS/ REMARKS

☐ yes ☐ no

If "yes", describe:____

**LEDoxy**	Indiv. No.: |__|__|-|__|__|__|-|__|__|__| Randomization Code: |__|__|__|
Date of visit: |__|__| / |__|__| / |__|__|__|__| (dd/mm/yyyy)	**VISIT 3 - TREATMENT**<br>**Day 14**

PRESENCE

Participant present for visit: ☐ yes ☐ no

If "no", participant: ☐ traveled with drugs ☐ is absent but drugs were given to health worker/family member/neighbor and drug intake was confirmed

☐ is absent (state reason:________________________________)

ADLA QUESTIONNAIRE *(only to be filled in if participant present for visit)*

ADLA attack since last visit: ☐ yes ☐ no Ongoing?: ☐ yes ☐ no

If "yes": Fever during attack: ☐ yes ☐ no Lymphnode swelling during attack: ☐ yes ☐ no

Peeling of limb after attack: ☐ yes ☐ no Able to work during attack: ☐ yes ☐ no

ADVERSE EVENTS *(only to be filled in if participant present for visit)*

Appearance of AEs / SAEs since last drug intake? ☐ yes ☐ no

*If "**yes**", please enter on the "Adverse Events" form at the end of this CRF.*

CONCOMITANT MEDICATION *(only to be filled in if participant present for visit)*

Change of medication since last visit? ☐ yes ☐ no

*If "**yes**", please enter on the "Concomitant Medication" form at the end of this CRF.*

INDIVIDUAL TREATMENT

Study drugs taken: ☐ yes ☐ no If "yes": **Treatment No.:** |__|__|

If "no", state reason: ☐ medical ☐ other (describe:________________________________)

PROBLEMS/ REMARKS

☐ yes ☐ no

If "yes", describe:________________________________

**LEDoxy**	Indiv. No.: |__|__|-|__|__|__|-|__|__|__| Randomization Code: |__|__|__|
Date of visit: |__|__| / |__|__| / |__|__|__|__| (dd/mm/yyyy)	**VISIT 3 - TREATMENT**<br>**Day 15**

PRESENCE

Participant present for visit: ☐ yes ☐ no

If "no", participant: ☐ traveled with drugs ☐ is absent but drugs were given to health worker/family member/neighbor and drug intake was confirmed

☐ is absent (state reason:____)

ADLA QUESTIONNAIRE *(only to be filled in if participant present for visit)*

ADLA attack since last visit: ☐ yes ☐ no Ongoing?: ☐ yes ☐ no

If "yes": Fever during attack: ☐ yes ☐ no Lymphnode swelling during attack: ☐ yes ☐ no

Peeling of limb after attack: ☐ yes ☐ no Able to work during attack: ☐ yes ☐ no

ADVERSE EVENTS *(only to be filled in if participant present for visit)*

Appearance of AEs / SAEs since last drug intake? ☐ yes ☐ no

*If "**yes**", please enter on the "Adverse Events" form at the end of this CRF.*

CONCOMITANT MEDICATION *(only to be filled in if participant present for visit)*

Change of medication since last visit? ☐ yes ☐ no

*If "**yes**", please enter on the "Concomitant Medication" form at the end of this CRF.*

PREGNANCY TEST (URINE)

Sampling date: |__|__| / |__|__| / |__|__|__|__| (dd/mm/yyyy) Sampling time: |__|__| : |__|__| ☐ AM ☐ PM (h:min)

Date analyzed: |__|__| / |__|__| / |__|__|__|__| (dd/mm/yyyy) Time analyzed: |__|__| : |__|__| ☐ AM ☐ PM (h:min)

Result: ☐ neg. ☐ pos. ☐ not applicable (☐ ≥ 55 years, ☐ surgically sterilized)

If pregnancy test positive, stop treatment!

INDIVIDUAL TREATMENT

Study drugs taken: ☐ yes ☐ no If "yes": **Treatment No.:** |__|__|

If "no", state reason: ☐ medical ☐ other (describe:______________________________________)

PROBLEMS/ REMARKS

☐ yes ☐ no

If "yes", describe:____

LEDoxy

Indiv. No.: |__|__|-|__|__|__|-|__|__|__|
Randomization Code: |__|__|__|

Date of visit: |__|__| / |__|__| / |__|__|__|__| (dd/mm/yyyy)

VISIT 3 - TREATMENT
Day 16

PRESENCE

Participant present for visit: ☐ yes ☐ no
If "no", participant: ☐ traveled with drugs ☐ is absent but drugs were given to health worker/family member/neighbor and drug intake was confirmed
☐ is absent (state reason:________________________________)

ADLA QUESTIONNAIRE *(only to be filled in if participant present for visit)*

ADLA attack since last visit: ☐ yes ☐ no   Ongoing?: ☐ yes ☐ no
If "yes": Fever during attack: ☐ yes ☐ no   Lymphnode swelling during attack: ☐ yes ☐ no
Peeling of limb after attack: ☐ yes ☐ no   Able to work during attack: ☐ yes ☐ no

ADVERSE EVENTS *(only to be filled in if participant present for visit)*

Appearance of AEs / SAEs since last drug intake? ☐ yes ☐ no
*If "**yes**", please enter on the "Adverse Events" form at the end of this CRF.*

CONCOMITANT MEDICATION *(only to be filled in if participant present for visit)*

Change of medication since last visit? ☐ yes ☐ no
*If "**yes**", please enter on the "Concomitant Medication" form at the end of this CRF.*

PREGNANCY TEST (URINE) *(only to be done if not done on day 15)*

Sampling date: |__|__| / |__|__| / |__|__|__|__| (dd/mm/yyyy)   Sampling time: |__|__| : |__|__| (h:min) ☐ AM ☐ PM
Date analyzed: |__|__| / |__|__| / |__|__|__|__| (dd/mm/yyyy)   Time analyzed: |__|__| : |__|__| (h:min) ☐ AM ☐ PM
Result: ☐ neg. ☐ pos. ☐ not applicable (☐ ≥ 55 years, ☐ surgically sterilized)
If pregnancy test positive, stop treatment!

INDIVIDUAL TREATMENT

Study drugs taken: ☐ yes ☐ no   If "yes": **Treatment No.:** |__|__|
If "no", state reason: ☐ medical ☐ other (describe:________________________________)

PROBLEMS/ REMARKS

☐ yes ☐ no
If "yes", describe:________________________________

LEDoxy	Indiv. No.: |__|__|-|__|__|__|-|__|__|__| Randomization Code: |__|__|__|
Date of visit: |__|__| / |__|__| / |__|__|__|__| (dd/mm/yyyy)	**VISIT 3 - TREATMENT<br>Day 17**

PRESENCE

Participant present for visit: ☐ yes ☐ no

If "no", participant: ☐ traveled with drugs ☐ is absent but drugs were given to health worker/family member/neighbor and drug intake was confirmed

☐ is absent (state reason:____)

ADLA QUESTIONNAIRE *(only to be filled in if participant present for visit)*

ADLA attack since last visit: ☐ yes ☐ no Ongoing?: ☐ yes ☐ no

If "yes": Fever during attack: ☐ yes ☐ no Lymphnode swelling during attack: ☐ yes ☐ no

Peeling of limb after attack: ☐ yes ☐ no Able to work during attack: ☐ yes ☐ no

ADVERSE EVENTS *(only to be filled in if participant present for visit)*

Appearance of AEs / SAEs since last drug intake? ☐ yes ☐ no

*If "**yes**", please enter on the "Adverse Events" form at the end of this CRF.*

CONCOMITANT MEDICATION *(only to be filled in if participant present for visit)*

Change of medication since last visit? ☐ yes ☐ no

*If "**yes**", please enter on the "Concomitant Medication" form at the end of this CRF.*

PREGNANCY TEST (URINE) *(only to be done if not done on day 15 or 16)*

Sampling date: |__|__| / |__|__| / |__|__|__|__| (dd/mm/yyyy) Sampling time: |__|__| : |__|__| (h:min) ☐ AM ☐ PM

Date analyzed: |__|__| / |__|__| / |__|__|__|__| (dd/mm/yyyy) Time analyzed: |__|__| : |__|__| (h:min) ☐ AM ☐ PM

Result: ☐ neg. ☐ pos. ☐ not applicable (☐ ≥ 55 years, ☐ surgically sterilized)

If pregnancy test positive, stop treatment!

INDIVIDUAL TREATMENT

Study drugs taken: ☐ yes ☐ no If "yes": **Treatment No.:** |__|__|

If "no", state reason: ☐ medical ☐ other (describe:____)

PROBLEMS/ REMARKS

☐ yes ☐ no

If "yes", describe:____

**LEDoxy**	Indiv. No.: |__|__|-|__|__|__|-|__|__|__| Randomization Code: |__|__|__|
Date of visit: |__|__| / |__|__| / |__|__|__|__| (dd/mm/yyyy)	**VISIT 3 - TREATMENT**<br>**Day 18**

PRESENCE

Participant present for visit: ☐ yes ☐ no

If "no", participant: ☐ traveled with drugs ☐ is absent but drugs were given to health worker/family member/neighbor and drug intake was confirmed

☐ is absent (state reason:____)

ADLA QUESTIONNAIRE *(only to be filled in if participant present for visit)*

ADLA attack since last visit: ☐ yes ☐ no Ongoing?: ☐ yes ☐ no

If "yes": Fever during attack: ☐ yes ☐ no Lymphnode swelling during attack: ☐ yes ☐ no

Peeling of limb after attack: ☐ yes ☐ no Able to work during attack: ☐ yes ☐ no

ADVERSE EVENTS *(only to be filled in if participant present for visit)*

Appearance of AEs / SAEs since last drug intake? ☐ yes ☐ no

*If "**yes**", please enter on the "Adverse Events" form at the end of this CRF.*

CONCOMITANT MEDICATION *(only to be filled in if participant present for visit)*

Change of medication since last visit? ☐ yes ☐ no

*If "**yes**", please enter on the "Concomitant Medication" form at the end of this CRF.*

INDIVIDUAL TREATMENT

Study drugs taken: ☐ yes ☐ no If "yes": **Treatment No.:** |__|__|

If "no", state reason: ☐ medical ☐ other (describe:________________________________)

PROBLEMS/ REMARKS

☐ yes ☐ no

If "yes", describe:____

LEDoxy

Indiv. No.: |__|__|-|__|__|__|-|__|__|__|

Randomization Code: |__|__|__|

Date of visit: |__|__| / |__|__| / |__|__|__|__|
(dd/mm/yyyy)

VISIT 3 - TREATMENT
Day 19

PRESENCE

Participant present for visit: ☐ yes ☐ no

If "no", participant: ☐ traveled with drugs ☐ is absent but drugs were given to health worker/family member/neighbor and drug intake was confirmed

☐ is absent (state reason:____)

ADLA QUESTIONNAIRE *(only to be filled in if participant present for visit)*

ADLA attack since last visit: ☐ yes ☐ no Ongoing?: ☐ yes ☐ no

If "yes": Fever during attack: ☐ yes ☐ no Lymphnode swelling during attack: ☐ yes ☐ no

Peeling of limb after attack: ☐ yes ☐ no Able to work during attack: ☐ yes ☐ no

ADVERSE EVENTS *(only to be filled in if participant present for visit)*

Appearance of AEs / SAEs since last drug intake? ☐ yes ☐ no

*If "**yes**", please enter on the "Adverse Events" form at the end of this CRF.*

CONCOMITANT MEDICATION *(only to be filled in if participant present for visit)*

Change of medication since last visit? ☐ yes ☐ no

*If "**yes**", please enter on the "Concomitant Medication" form at the end of this CRF.*

INDIVIDUAL TREATMENT

Study drugs taken: ☐ yes ☐ no If "yes": **Treatment No.: |__|__|**

If "no", state reason: ☐ medical ☐ other (describe:____)

PROBLEMS/ REMARKS

☐ yes ☐ no

If "yes", describe:____

LEDoxy

Indiv. No.: |__|__|-|__|__|__|-|__|__|__|
Randomization Code: |__|__|__|

Date of visit: |__|__| / |__|__| / |__|__|__|__|
(dd/mm/yyyy)

VISIT 3 - TREATMENT
Day 20

PRESENCE

Participant present for visit: ☐ yes ☐ no
If "no", participant: ☐ traveled with drugs ☐ is absent but drugs were given to health worker/family member/neighbor and drug intake was confirmed
☐ is absent (state reason:____)

ADLA QUESTIONNAIRE *(only to be filled in if participant present for visit)*

ADLA attack since last visit: ☐ yes ☐ no Ongoing?: ☐ yes ☐ no
If "yes": Fever during attack: ☐ yes ☐ no Lymphnode swelling during attack: ☐ yes ☐ no
Peeling of limb after attack: ☐ yes ☐ no Able to work during attack: ☐ yes ☐ no

ADVERSE EVENTS *(only to be filled in if participant present for visit)*

Appearance of AEs / SAEs since last drug intake? ☐ yes ☐ no
*If "**yes**", please enter on the "Adverse Events" form at the end of this CRF.*

CONCOMITANT MEDICATION *(only to be filled in if participant present for visit)*

Change of medication since last visit? ☐ yes ☐ no
*If "**yes**", please enter on the "Concomitant Medication" form at the end of this CRF.*

INDIVIDUAL TREATMENT

Study drugs taken: ☐ yes ☐ no If "yes": **Treatment No.:** |__|__|
If "no", state reason: ☐ medical ☐ other (describe:____)

PROBLEMS/ REMARKS

☐ yes ☐ no
If "yes", describe:____

LEDoxy

Indiv. No.: |__|__|-|__|__|__|-|__|__|__|

Randomization Code: |__|__|__|

Date of visit: |__|__| / |__|__| / |__|__|__|__|
(dd/mm/yyyy)

VISIT 3 - TREATMENT
Day 21

PRESENCE

Participant present for visit: ☐ yes ☐ no

If "no", participant: ☐ traveled with drugs ☐ is absent but drugs were given to health worker/family member/neighbor and drug intake was confirmed

☐ is absent (state reason:________________________________)

ADLA QUESTIONNAIRE *(only to be filled in if participant present for visit)*

ADLA attack since last visit: ☐ yes ☐ no Ongoing?: ☐ yes ☐ no

If "yes": Fever during attack: ☐ yes ☐ no Lymphnode swelling during attack: ☐ yes ☐ no

Peeling of limb after attack: ☐ yes ☐ no Able to work during attack: ☐ yes ☐ no

ADVERSE EVENTS *(only to be filled in if participant present for visit)*

Appearance of AEs / SAEs since last drug intake? ☐ yes ☐ no

*If "**yes**", please enter on the "Adverse Events" form at the end of this CRF.*

CONCOMITANT MEDICATION *(only to be filled in if participant present for visit)*

Change of medication since last visit? ☐ yes ☐ no

*If "**yes**", please enter on the "Concomitant Medication" form at the end of this CRF.*

INDIVIDUAL TREATMENT

Study drugs taken: ☐ yes ☐ no If "yes": **Treatment No.:** |__|__|

If "no", state reason: ☐ medical ☐ other (describe:________________________________)

PROBLEMS/ REMARKS

☐ yes ☐ no

If "yes", describe:________________________________

LEDoxy

Indiv. No.: |__|__|-|__|__|__|-|__|__|__|
Randomization Code: |__|__|__|

Date of visit: |__|__| / |__|__| / |__|__|__|__|
(dd/mm/yyyy)

VISIT 3 - TREATMENT
Day 22

PRESENCE

Participant present for visit: ☐ yes ☐ no
If "no", participant: ☐ traveled with drugs ☐ is absent but drugs were given to health worker/family member/neighbor and drug intake was confirmed
☐ is absent (state reason:____)

ADLA QUESTIONNAIRE *(only to be filled in if participant present for visit)*

ADLA attack since last visit: ☐ yes ☐ no Ongoing?: ☐ yes ☐ no
If "yes": Fever during attack: ☐ yes ☐ no Lymphnode swelling during attack: ☐ yes ☐ no
Peeling of limb after attack: ☐ yes ☐ no Able to work during attack: ☐ yes ☐ no

ADVERSE EVENTS *(only to be filled in if participant present for visit)*

Appearance of AEs / SAEs since last drug intake? ☐ yes ☐ no
*If "**yes**", please enter on the "Adverse Events" form at the end of this CRF.*

CONCOMITANT MEDICATION *(only to be filled in if participant present for visit)*

Change of medication since last visit? ☐ yes ☐ no
*If "**yes**", please enter on the "Concomitant Medication" form at the end of this CRF.*

LABORATORY ASSESSMENT BEFORE TREATMENT NO.22

Blood sample taken: ☐ yes ☐ no
If "no", state reason: ☐ refused ☐ absent ☐ other (specify: ________________________________)
If "yes": Sampling date: |__|__| / |__|__| / |__|__|__|__| (dd/mm/yyyy) Sampling time: |__|__| : |__|__| (h:min) ☐ AM ☐ PM
Please attach corresponding barcode label(s) on the "Laboratory documentation sheet".
Please enter results on the „Laboratory assessment" form at the end of this CRF.

INDIVIDUAL TREATMENT

Study drugs taken: ☐ yes ☐ no If "yes": **Treatment No.:** |__|__|
If "no", state reason: ☐ medical ☐ other (describe:________________________________)

PROBLEMS/ REMARKS

☐ yes ☐ no
If "yes", describe:____

**LEDoxy**	Indiv. No.: |__|__|-|__|__|__|-|__|__|__| Randomization Code: |__|__|__|
Date of visit: |__|__| / |__|__| / |__|__|__|__| (dd/mm/yyyy)	**VISIT 3 - TREATMENT**<br>**Day 23**

PRESENCE

Participant present for visit: ☐ yes ☐ no

If "no", participant: ☐ traveled with drugs ☐ is absent but drugs were given to health worker/family member/neighbor and drug intake was confirmed

☐ is absent (state reason:____)

ADLA QUESTIONNAIRE *(only to be filled in if participant present for visit)*

ADLA attack since last visit: ☐ yes ☐ no Ongoing?: ☐ yes ☐ no

If "yes": Fever during attack: ☐ yes ☐ no Lymphnode swelling during attack: ☐ yes ☐ no

Peeling of limb after attack: ☐ yes ☐ no Able to work during attack: ☐ yes ☐ no

ADVERSE EVENTS *(only to be filled in if participant present for visit)*

Appearance of AEs / SAEs since last drug intake? ☐ yes ☐ no

*If "**yes**", please enter on the "Adverse Events" form at the end of this CRF.*

CONCOMITANT MEDICATION *(only to be filled in if participant present for visit)*

Change of medication since last visit? ☐ yes ☐ no

*If "**yes**", please enter on the "Concomitant Medication" form at the end of this CRF.*

LABORATORY ASSESSMENT BEFORE TREATMENT NO.22 *(only applicable if not done on day 22)*

Blood sample taken: ☐ yes ☐ no

If "no", state reason: ☐ refused ☐ absent ☐ other (specify: ____)

If "yes": Sampling date: |__|__| / |__|__| / |__|__|__|__| (dd/mm/yyyy) Sampling time: |__|__| : |__|__| (h:min) ☐ AM ☐ PM

Please attach corresponding barcode label(s) on the "Laboratory documentation sheet".
Please enter results on the „Laboratory assessment" form at the end of this CRF.

INDIVIDUAL TREATMENT

Study drugs taken: ☐ yes ☐ no If "yes": **Treatment No.:** |__|__|

If "no", state reason: ☐ medical ☐ other (describe:____)

PROBLEMS/ REMARKS

☐ yes ☐ no

If "yes", describe:____

LEDoxy

Indiv. No.: |__|__|-|__|__|__|-|__|__|__|
Randomization Code: |__|__|__|

Date of visit: |__|__| / |__|__| / |__|__|__|__|
(dd/mm/yyyy)

VISIT 3 - TREATMENT
Day 24

PRESENCE

Participant present for visit: ☐ yes ☐ no
If "no", participant: ☐ traveled with drugs ☐ is absent but drugs were given to health worker/family member/neighbor and drug intake was confirmed
☐ is absent (state reason:____)

ADLA QUESTIONNAIRE *(only to be filled in if participant present for visit)*

ADLA attack since last visit: ☐ yes ☐ no Ongoing?: ☐ yes ☐ no
If "yes": Fever during attack: ☐ yes ☐ no Lymphnode swelling during attack: ☐ yes ☐ no
Peeling of limb after attack: ☐ yes ☐ no Able to work during attack: ☐ yes ☐ no

ADVERSE EVENTS *(only to be filled in if participant present for visit)*

Appearance of AEs / SAEs since last drug intake? ☐ yes ☐ no
*If "**yes**", please enter on the "Adverse Events" form at the end of this CRF.*

CONCOMITANT MEDICATION *(only to be filled in if participant present for visit)*

Change of medication since last visit? ☐ yes ☐ no
*If "**yes**", please enter on the "Concomitant Medication" form at the end of this CRF.*

LABORATORY ASSESSMENT BEFORE TREATMENT NO.22 *(only applicable if not done on day 22 or 23)*

Blood sample taken: ☐ yes ☐ no
If "no", state reason: ☐ refused ☐ absent ☐ other (specify: ________________________________)
If "yes": Sampling date: |__|__| / |__|__| / |__|__|__|__| (dd/mm/yyyy) Sampling time: |__|__| : |__|__| (h:min) ☐ AM ☐ PM
Please attach corresponding barcode label(s) on the "Laboratory documentation sheet".
Please enter results on the „Laboratory assessment" form at the end of this CRF.

INDIVIDUAL TREATMENT

Study drugs taken: ☐ yes ☐ no If "yes": **Treatment No.:** |__|__|
If "no", state reason: ☐ medical ☐ other (describe:________________________________)

PROBLEMS/ REMARKS

☐ yes ☐ no
If "yes", describe:____

**LEDoxy**	Indiv. No.: |__|__|-|__|__|__|-|__|__|__| Randomization Code: |__|__|__|
Date of visit: |__|__| / |__|__| / |__|__|__|__| (dd/mm/yyyy)	**VISIT 3 - TREATMENT**<br>**Day 25**

PRESENCE

Participant present for visit: ☐ yes ☐ no

If "no", participant: ☐ traveled with drugs ☐ is absent but drugs were given to health worker/family member/neighbor and drug intake was confirmed

☐ is absent (state reason:____)

ADLA QUESTIONNAIRE *(only to be filled in if participant present for visit)*

ADLA attack since last visit: ☐ yes ☐ no Ongoing?: ☐ yes ☐ no

If "yes": Fever during attack: ☐ yes ☐ no Lymphnode swelling during attack: ☐ yes ☐ no

Peeling of limb after attack: ☐ yes ☐ no Able to work during attack: ☐ yes ☐ no

ADVERSE EVENTS *(only to be filled in if participant present for visit)*

Appearance of AEs / SAEs since last drug intake? ☐ yes ☐ no

*If "**yes**", please enter on the "Adverse Events" form at the end of this CRF.*

CONCOMITANT MEDICATION *(only to be filled in if participant present for visit)*

Change of medication since last visit? ☐ yes ☐ no

*If "**yes**", please enter on the "Concomitant Medication" form at the end of this CRF.*

LABORATORY ASSESSMENT BEFORE TREATMENT NO.22 *(only applicable if not done on day 22 – 24)*

Blood sample taken: ☐ yes ☐ no

If "no", state reason: ☐ refused ☐ absent ☐ other (specify: ____)

If "yes": Sampling date: |__|__| / |__|__| / |__|__|__|__| (dd/mm/yyyy) Sampling time: |__|__| : |__|__| (h:min) ☐ AM ☐ PM

Please attach corresponding barcode label(s) on the "Laboratory documentation sheet".
Please enter results on the „Laboratory assessment" form at the end of this CRF.

INDIVIDUAL TREATMENT

Study drugs taken: ☐ yes ☐ no If "yes": **Treatment No.:** |__|__|

If "no", state reason: ☐ medical ☐ other (describe:____)

PROBLEMS/ REMARKS

☐ yes ☐ no

If "yes", describe:____

LEDoxy

Indiv. No.: |__|__|-|__|__|__|-|__|__|__|
Randomization Code: |__|__|__|

Date of visit: |__|__| / |__|__| / |__|__|__|__| (dd/mm/yyyy)

VISIT 3 - TREATMENT
Day 26

PRESENCE

Participant present for visit: ☐ yes ☐ no
If "no", participant: ☐ traveled with drugs ☐ is absent but drugs were given to health worker/family member/neighbor and drug intake was confirmed
☐ is absent (state reason:____)

ADLA QUESTIONNAIRE *(only to be filled in if participant present for visit)*

ADLA attack since last visit: ☐ yes ☐ no Ongoing?: ☐ yes ☐ no
If "yes": Fever during attack: ☐ yes ☐ no Lymphnode swelling during attack: ☐ yes ☐ no
Peeling of limb after attack: ☐ yes ☐ no Able to work during attack: ☐ yes ☐ no

ADVERSE EVENTS *(only to be filled in if participant present for visit)*

Appearance of AEs / SAEs since last drug intake? ☐ yes ☐ no
*If "**yes**", please enter on the "Adverse Events" form at the end of this CRF.*

CONCOMITANT MEDICATION *(only to be filled in if participant present for visit)*

Change of medication since last visit? ☐ yes ☐ no
*If "**yes**", please enter on the "Concomitant Medication" form at the end of this CRF.*

LABORATORY ASSESSMENT BEFORE TREATMENT NO.22 *(only applicable if not done on day 22 - 25)*

Blood sample taken: ☐ yes ☐ no
If "no", state reason: ☐ refused ☐ absent ☐ other (specify: ________________________________)
If "yes": Sampling date: |__|__| / |__|__| / |__|__|__|__| (dd/mm/yyyy) Sampling time: |__|__| : |__|__| (h:min) ☐ AM ☐ PM
Please attach corresponding barcode label(s) on the "Laboratory documentation sheet".
Please enter results on the „Laboratory assessment" form at the end of this CRF.

INDIVIDUAL TREATMENT

Study drugs taken: ☐ yes ☐ no If "yes": **Treatment No.:** |__|__|
If "no", state reason: ☐ medical ☐ other (describe:________________________________)

PROBLEMS/ REMARKS

☐ yes ☐ no
If "yes", describe:____

**LEDoxy**	Indiv. No.: |__|__|-|__|__|__|-|__|__|__| Randomization Code: |__|__|__|
Date of visit: |__|__| / |__|__| / |__|__|__|__| (dd/mm/yyyy)	**VISIT 3 - TREATMENT**<br>**Day 27**

PRESENCE

Participant present for visit: ☐ yes ☐ no

If "no", participant: ☐ traveled with drugs ☐ is absent but drugs were given to health worker/family member/neighbor and drug intake was confirmed

☐ is absent (state reason:____)

ADLA QUESTIONNAIRE *(only to be filled in if participant present for visit)*

ADLA attack since last visit: ☐ yes ☐ no Ongoing?: ☐ yes ☐ no

If "yes": Fever during attack: ☐ yes ☐ no Lymphnode swelling during attack: ☐ yes ☐ no

Peeling of limb after attack: ☐ yes ☐ no Able to work during attack: ☐ yes ☐ no

ADVERSE EVENTS *(only to be filled in if participant present for visit)*

Appearance of AEs / SAEs since last drug intake? ☐ yes ☐ no

*If "**yes**", please enter on the "Adverse Events" form at the end of this CRF.*

CONCOMITANT MEDICATION *(only to be filled in if participant present for visit)*

Change of medication since last visit? ☐ yes ☐ no

*If "**yes**", please enter on the "Concomitant Medication" form at the end of this CRF.*

LABORATORY ASSESSMENT BEFORE TREATMENT NO.22 *(only applicable if not done on day 22 - 26)*

Blood sample taken: ☐ yes ☐ no

If "no", state reason: ☐ refused ☐ absent ☐ other (specify: ________________________________)

If "yes": Sampling date: \|__\|__\| / \|__\|__\| / \|__\|__\|__\|__\| (dd/mm/yyyy) Sampling time: \|__\|__\| : \|__\|__\| (h:min) ☐ AM ☐ PM

Please attach corresponding barcode label(s) on the "Laboratory documentation sheet".
Please enter results on the „Laboratory assessment" form at the end of this CRF.

INDIVIDUAL TREATMENT

Study drugs taken: ☐ yes ☐ no If "yes": **Treatment No.:** \|__\|__\|

If "no", state reason: ☐ medical ☐ other (describe:________________________________)

PROBLEMS/ REMARKS

☐ yes ☐ no

If "yes", describe:____

**LEDoxy**	Indiv. No.: |__|__|-|__|__|__|-|__|__|__| Randomization Code: |__|__|__|
Date of visit: |__|__| / |__|__| / |__|__|__|__| (dd/mm/yyyy)	**VISIT 3 - TREATMENT**<br>**Day 28**

PRESENCE

Participant present for visit: ☐ yes ☐ no

If "no", participant: ☐ traveled with drugs ☐ is absent but drugs were given to health worker/family member/neighbor and drug intake was confirmed

☐ is absent (state reason:____)

ADLA QUESTIONNAIRE *(only to be filled in if participant present for visit)*

ADLA attack since last visit: ☐ yes ☐ no   Ongoing?: ☐ yes ☐ no

If "yes": Fever during attack: ☐ yes ☐ no   Lymphnode swelling during attack: ☐ yes ☐ no

Peeling of limb after attack: ☐ yes ☐ no   Able to work during attack: ☐ yes ☐ no

ADVERSE EVENTS *(only to be filled in if participant present for visit)*

Appearance of AEs / SAEs since last drug intake? ☐ yes ☐ no

*If "**yes**", please enter on the "Adverse Events" form at the end of this CRF.*

CONCOMITANT MEDICATION *(only to be filled in if participant present for visit)*

Change of medication since last visit? ☐ yes ☐ no

*If "**yes**", please enter on the "Concomitant Medication" form at the end of this CRF.*

LABORATORY ASSESSMENT BEFORE TREATMENT NO.22 *(only applicable if not done on day 22 - 27)*

Blood sample taken: ☐ yes ☐ no

If "no", state reason: ☐ refused ☐ absent ☐ other (specify: ________________________________)

If "yes": Sampling date: |__|__| / |__|__| / |__|__|__|__| (dd/mm/yyyy) Sampling time: |__|__| : |__|__| (h:min) ☐ AM ☐ PM

Please attach corresponding barcode label(s) on the "Laboratory documentation sheet".
Please enter results on the „Laboratory assessment" form at the end of this CRF.

INDIVIDUAL TREATMENT

Study drugs taken: ☐ yes ☐ no   If "yes": **Treatment No.:** |__|__|

If "no", state reason: ☐ medical ☐ other (describe:________________________________)

PROBLEMS/ REMARKS

☐ yes ☐ no

If "yes", describe:____

LEDoxy

Indiv. No.: |__|__|-|__|__|__|-|__|__|__|
Randomization Code: |__|__|__|

Date of visit: |__|__| / |__|__| / |__|__|__|__|
(dd/mm/yyyy)

VISIT 3 - TREATMENT
Day 29

PRESENCE

Participant present for visit: ☐ yes ☐ no
If "no", participant: ☐ traveled with drugs ☐ is absent but drugs were given to health worker/family member/neighbor and drug intake was confirmed
☐ is absent (state reason:____)

ADLA QUESTIONNAIRE *(only to be filled in if participant present for visit)*

ADLA attack since last visit: ☐ yes ☐ no Ongoing?: ☐ yes ☐ no
If "yes": Fever during attack: ☐ yes ☐ no Lymphnode swelling during attack: ☐ yes ☐ no
Peeling of limb after attack: ☐ yes ☐ no Able to work during attack: ☐ yes ☐ no

ADVERSE EVENTS *(only to be filled in if participant present for visit)*

Appearance of AEs / SAEs since last drug intake? ☐ yes ☐ no
*If "**yes**", please enter on the "Adverse Events" form at the end of this CRF.*

CONCOMITANT MEDICATION *(only to be filled in if participant present for visit)*

Change of medication since last visit? ☐ yes ☐ no
*If "**yes**", please enter on the "Concomitant Medication" form at the end of this CRF.*

LABORATORY ASSESSMENT BEFORE TREATMENT NO.22 *(only applicable if not done on day 22 - 28)*

Blood sample taken: ☐ yes ☐ no
If "no", state reason: ☐ refused ☐ absent ☐ other (specify: ________________________________)
If "yes": Sampling date: |__|__| / |__|__| / |__|__|__|__| Sampling time: |__|__| : |__|__| ☐ AM ☐ PM
(dd/mm/yyyy) (h:min)
Please attach corresponding barcode label(s) on the "Laboratory documentation sheet".
Please enter results on the „Laboratory assessment" form at the end of this CRF.

PREGNANCY TEST (URINE)

Sampling date: |__|__| / |__|__| / |__|__|__|__| Sampling time: |__|__| : |__|__| ☐ AM ☐ PM
(dd/mm/yyyy) (h:min)
Date analyzed: |__|__| / |__|__| / |__|__|__|__| Time analyzed: |__|__| : |__|__| ☐ AM ☐ PM
(dd/mm/yyyy) (h:min)
Result: ☐ neg. ☐ pos. ☐ not applicable (☐ ≥ 55 years, ☐ surgically sterilized)
If pregnancy test positive, stop treatment!

INDIVIDUAL TREATMENT

Study drugs taken: ☐ yes ☐ no If "yes": **Treatment No**|__|__|
If "no", state reason: ☐ medical ☐ other (describe:________________________________)

PROBLEMS/ REMARKS

☐ yes ☐ no

If "yes", describe:____

**LEDoxy**	Indiv. No.: |__|__|-|__|__|__|-|__|__|__| Randomization Code: |__|__|__|
Date of visit: |__|__| / |__|__| / |__|__|__|__| (dd/mm/yyyy)	**VISIT 3 - TREATMENT**<br>**Day 30**

PRESENCE

Participant present for visit: ☐ yes ☐ no

If "no", participant: ☐ traveled with drugs ☐ is absent but drugs were given to health worker/family member/neighbor and drug intake was confirmed

☐ is absent (state reason:____)

ADLA QUESTIONNAIRE *(only to be filled in if participant present for visit)*

ADLA attack since last visit: ☐ yes ☐ no Ongoing?: ☐ yes ☐ no

If "yes": Fever during attack: ☐ yes ☐ no Lymphnode swelling during attack: ☐ yes ☐ no

Peeling of limb after attack: ☐ yes ☐ no Able to work during attack: ☐ yes ☐ no

ADVERSE EVENTS *(only to be filled in if participant present for visit)*

Appearance of AEs / SAEs since last drug intake? ☐ yes ☐ no

*If "**yes**", please enter on the "Adverse Events" form at the end of this CRF.*

CONCOMITANT MEDICATION *(only to be filled in if participant present for visit)*

Change of medication since last visit? ☐ yes ☐ no

*If "**yes**", please enter on the "Concomitant Medication" form at the end of this CRF.*

PREGNANCY TEST (URINE) *(only to be done if not done on day 29)*

Sampling date: |__|__| / |__|__| / |__|__|__|__| (dd/mm/yyyy) Sampling time: |__|__| : |__|__| ☐ AM ☐ PM (h:min)

Date analyzed: |__|__| / |__|__| / |__|__|__|__| (dd/mm/yyyy) Time analyzed: |__|__| : |__|__| ☐ AM ☐ PM (h:min)

Result: ☐ neg. ☐ pos. ☐ not applicable (☐ ≥ 55 years, ☐ surgically sterilized)

If pregnancy test positive, stop treatment!

INDIVIDUAL TREATMENT

Study drugs taken: ☐ yes ☐ no If "yes": **Treatment No.:** |__|__|

If "no", state reason: ☐ medical ☐ other (describe:____)

PROBLEMS/ REMARKS

☐ yes ☐ no

If "yes", describe:____

**LEDoxy**	Indiv. No.: |__|__|-|__|__|__|-|__|__|__| Randomization Code: |__|__|__|
Date of visit: |__|__| / |__|__| / |__|__|__|__| (dd/mm/yyyy)	**VISIT 3 - TREATMENT**<br>**Day 31**

PRESENCE

Participant present for visit: ☐ yes ☐ no

If "no", participant: ☐ traveled with drugs ☐ is absent but drugs were given to health worker/family member/neighbor and drug intake was confirmed

☐ is absent (state reason:____)

ADLA QUESTIONNAIRE *(only to be filled in if participant present for visit)*

ADLA attack since last visit: ☐ yes ☐ no Ongoing?: ☐ yes ☐ no

If "yes": Fever during attack: ☐ yes ☐ no Lymphnode swelling during attack: ☐ yes ☐ no

Peeling of limb after attack: ☐ yes ☐ no Able to work during attack: ☐ yes ☐ no

ADVERSE EVENTS *(only to be filled in if participant present for visit)*

Appearance of AEs / SAEs since last drug intake? ☐ yes ☐ no

*If "**yes**", please enter on the "Adverse Events" form at the end of this CRF.*

CONCOMITANT MEDICATION *(only to be filled in if participant present for visit)*

Change of medication since last visit? ☐ yes ☐ no

*If "**yes**", please enter on the "Concomitant Medication" form at the end of this CRF.*

PREGNANCY TEST (URINE) *(only to be done if not done on day 29 or 30)*

Sampling date: |__|__| / |__|__| / |__|__|__|__| (dd/mm/yyyy) Sampling time: |__|__| : |__|__| ☐ AM ☐ PM (h:min)

Date analyzed: |__|__| / |__|__| / |__|__|__|__| (dd/mm/yyyy) Time analyzed: |__|__| : |__|__| ☐ AM ☐ PM (h:min)

Result: ☐ neg. ☐ pos. ☐ not applicable (☐ ≥ 55 years, ☐ surgically sterilized)

If pregnancy test positive, stop treatment!

INDIVIDUAL TREATMENT

Study drugs taken: ☐ yes ☐ no If "yes": **Treatment No.:** |__|__|

If "no", state reason: ☐ medical ☐ other (describe:__________________________________)

PROBLEMS/ REMARKS

☐ yes ☐ no

If "yes", describe:____

PRESENCE

Participant present for visit: ☐ yes ☐ no

If "no", participant: ☐ traveled with drugs ☐ is absent but drugs were given to health worker/family member/neighbor and drug intake was confirmed

☐ is absent (state reason:______________________________)

ADLA QUESTIONNAIRE *(only to be filled in if participant present for visit)*

ADLA attack since last visit: ☐ yes ☐ no Ongoing?: ☐ yes ☐ no

If "yes": Fever during attack: ☐ yes ☐ no Lymphnode swelling during attack: ☐ yes ☐ no

Peeling of limb after attack: ☐ yes ☐ no Able to work during attack: ☐ yes ☐ no

ADVERSE EVENTS *(only to be filled in if participant present for visit)*

Appearance of AEs / SAEs since last drug intake? ☐ yes ☐ no

*If "**yes**", please enter on the "Adverse Events" form at the end of this CRF.*

CONCOMITANT MEDICATION *(only to be filled in if participant present for visit)*

Change of medication since last visit? ☐ yes ☐ no

*If "**yes**", please enter on the "Concomitant Medication" form at the end of this CRF.*

INDIVIDUAL TREATMENT

Study drugs taken: ☐ yes ☐ no If "yes": **Treatment No.:** |__|__|

If "no", state reason: ☐ medical ☐ other (describe:______________________________)

PROBLEMS/ REMARKS

☐ yes ☐ no

If "yes", describe:______________________________

LEDoxy

Indiv. No.: |__|__|-|__|__|__|-|__|__|__|
Randomization Code: |__|__|__|

Date of visit: |__|__| / |__|__| / |__|__|__|__|
(dd/mm/yyyy)

VISIT 3 - TREATMENT
Day 33

PRESENCE

Participant present for visit: ☐ yes ☐ no
If "no", participant: ☐ traveled with drugs ☐ is absent but drugs were given to health worker/family member/neighbor and drug intake was confirmed
☐ is absent (state reason:________________________________)

ADLA QUESTIONNAIRE *(only to be filled in if participant present for visit)*

ADLA attack since last visit: ☐ yes ☐ no Ongoing?: ☐ yes ☐ no
If "yes": Fever during attack: ☐ yes ☐ no Lymphnode swelling during attack: ☐ yes ☐ no
Peeling of limb after attack: ☐ yes ☐ no Able to work during attack: ☐ yes ☐ no

ADVERSE EVENTS *(only to be filled in if participant present for visit)*

Appearance of AEs / SAEs since last drug intake? ☐ yes ☐ no
*If "**yes**", please enter on the "Adverse Events" form at the end of this CRF.*

CONCOMITANT MEDICATION *(only to be filled in if participant present for visit)*

Change of medication since last visit? ☐ yes ☐ no
*If "**yes**", please enter on the "Concomitant Medication" form at the end of this CRF.*

INDIVIDUAL TREATMENT

Study drugs taken: ☐ yes ☐ no If "yes": **Treatment No.:** |__|__|
If "no", state reason: ☐ medical ☐ other (describe:________________________________)

PROBLEMS/ REMARKS

☐ yes ☐ no
If "yes", describe:________________________________

**LEDoxy**	Indiv. No.: |__|__|-|__|__|__|-|__|__|__| Randomization Code: |__|__|__|
Date of visit: |__|__| / |__|__| / |__|__|__|__| (dd/mm/yyyy)	**VISIT 3 - TREATMENT**<br>**Day 34**

PRESENCE

Participant present for visit: ☐ yes ☐ no

If "no", participant: ☐ traveled with drugs ☐ is absent but drugs were given to health worker/family member/neighbor and drug intake was confirmed

☐ is absent (state reason:____)

ADLA QUESTIONNAIRE *(only to be filled in if participant present for visit)*

ADLA attack since last visit: ☐ yes ☐ no Ongoing?: ☐ yes ☐ no

If "yes": Fever during attack: ☐ yes ☐ no Lymphnode swelling during attack: ☐ yes ☐ no

Peeling of limb after attack: ☐ yes ☐ no Able to work during attack: ☐ yes ☐ no

ADVERSE EVENTS *(only to be filled in if participant present for visit)*

Appearance of AEs / SAEs since last drug intake? ☐ yes ☐ no

*If "**yes**", please enter on the "Adverse Events" form at the end of this CRF.*

CONCOMITANT MEDICATION *(only to be filled in if participant present for visit)*

Change of medication since last visit? ☐ yes ☐ no

*If "**yes**", please enter on the "Concomitant Medication" form at the end of this CRF.*

INDIVIDUAL TREATMENT

Study drugs taken: ☐ yes ☐ no If "yes": **Treatment No.:** |__|__|

If "no", state reason: ☐ medical ☐ other (describe:____)

PROBLEMS/ REMARKS

☐ yes ☐ no

If "yes", describe:____

LEDoxy

Indiv. No.: |__|__|-|__|__|__|-|__|__|__|
Randomization Code: |__|__|__|

Date of visit: |__|__| / |__|__| / |__|__|__|__|
(dd/mm/yyyy)

VISIT 3 - TREATMENT
Day 35

PRESENCE

Participant present for visit: ☐ yes ☐ no

If "no", participant: ☐ traveled with drugs ☐ is absent but drugs were given to health worker/family member/neighbor and drug intake was confirmed

☐ is absent (state reason:____)

ADLA QUESTIONNAIRE *(only to be filled in if participant present for visit)*

ADLA attack since last visit: ☐ yes ☐ no Ongoing?: ☐ yes ☐ no

If "yes": Fever during attack: ☐ yes ☐ no Lymphnode swelling during attack: ☐ yes ☐ no

Peeling of limb after attack: ☐ yes ☐ no Able to work during attack: ☐ yes ☐ no

ADVERSE EVENTS *(only to be filled in if participant present for visit)*

Appearance of AEs / SAEs since last drug intake? ☐ yes ☐ no

*If "**yes**", please enter on the "Adverse Events" form at the end of this CRF.*

CONCOMITANT MEDICATION *(only to be filled in if participant present for visit)*

Change of medication since last visit? ☐ yes ☐ no

*If "**yes**", please enter on the "Concomitant Medication" form at the end of this CRF.*

INDIVIDUAL TREATMENT

Study drugs taken: ☐ yes ☐ no If "yes": **Treatment No.:** |__|__|

If "no", state reason: ☐ medical ☐ other (describe:______________________________________)

PROBLEMS/ REMARKS

☐ yes ☐ no

If "yes", describe:____

LEDoxy

Indiv. No.: |__|__|-|__|__|__|-|__|__|__|
Randomization Code: |__|__|__|

Date of visit: |__|__| / |__|__| / |__|__|__|__|
(dd/mm/yyyy)

VISIT 3 - TREATMENT
Day 36

PRESENCE

Participant present for visit: ☐ yes ☐ no
If "no", participant: ☐ traveled with drugs ☐ is absent but drugs were given to health worker/family member/neighbor and drug intake was confirmed
☐ is absent (state reason:________________________________)

ADLA QUESTIONNAIRE *(only to be filled in if participant present for visit)*

ADLA attack since last visit: ☐ yes ☐ no Ongoing?: ☐ yes ☐ no
If "yes": Fever during attack: ☐ yes ☐ no Lymphnode swelling during attack: ☐ yes ☐ no
Peeling of limb after attack: ☐ yes ☐ no Able to work during attack: ☐ yes ☐ no

ADVERSE EVENTS *(only to be filled in if participant present for visit)*

Appearance of AEs / SAEs since last drug intake? ☐ yes ☐ no
*If "**yes**", please enter on the "Adverse Events" form at the end of this CRF.*

CONCOMITANT MEDICATION *(only to be filled in if participant present for visit)*

Change of medication since last visit? ☐ yes ☐ no
*If "**yes**", please enter on the "Concomitant Medication" form at the end of this CRF.*

INDIVIDUAL TREATMENT

Study drugs taken: ☐ yes ☐ no If "yes": **Treatment No.:** |__|__|
If "no", state reason: ☐ medical ☐ other (describe:________________________________)

PROBLEMS/ REMARKS

☐ yes ☐ no
If "yes", describe:____

**LEDoxy**	Indiv. No.: |__|__|-|__|__|__|-|__|__|__| Randomization Code: |__|__|__|
Date of visit: |__|__| / |__|__| / |__|__|__|__| (dd/mm/yyyy)	**VISIT 3 - TREATMENT**<br>**Day 37**

PRESENCE

Participant present for visit: ☐ yes ☐ no

If “no”, participant: ☐ traveled with drugs ☐ is absent but drugs were given to health worker/family member/neighbor and drug intake was confirmed

☐ is absent (state reason:____)

ADLA QUESTIONNAIRE *(only to be filled in if participant present for visit)*

ADLA attack since last visit: ☐ yes ☐ no Ongoing?: ☐ yes ☐ no

If “yes”: Fever during attack: ☐ yes ☐ no Lymphnode swelling during attack: ☐ yes ☐ no

Peeling of limb after attack: ☐ yes ☐ no Able to work during attack: ☐ yes ☐ no

ADVERSE EVENTS *(only to be filled in if participant present for visit)*

Appearance of AEs / SAEs since last drug intake? ☐ yes ☐ no

*If “**yes**”, please enter on the “Adverse Events“ form at the end of this CRF.*

CONCOMITANT MEDICATION *(only to be filled in if participant present for visit)*

Change of medication since last visit? ☐ yes ☐ no

*If “**yes**”, please enter on the “Concomitant Medication“ form at the end of this CRF.*

INDIVIDUAL TREATMENT

Study drugs taken: ☐ yes ☐ no If “yes”: **Treatment No.:** |__|__|

If “no”, state reason: ☐ medical ☐ other (describe:____)

PROBLEMS/ REMARKS

☐ yes ☐ no

If “yes”, describe:____

**PodoLEDoxy**	Indiv. No.: |__|__|-|__|__|__|-|__|__|__| Randomization Code: |__|__|__|
Date of visit: |__|__| / |__|__| / |__|__|__|__| (dd/mm/yyyy)	**VISIT 3 - TREATMENT**<br>**Day 38**

PRESENCE

Participant present for visit: ☐ yes ☐ no

If "no", participant: ☐ traveled with drugs ☐ is absent but drugs were given to health worker/family member/neighbor and drug intake was confirmed

☐ is absent (state reason:_______________________________________)

ADLA QUESTIONNAIRE *(only to be filled in if participant present for visit)*

ADLA attack since last visit: ☐ yes ☐ no Ongoing?: ☐ yes ☐ no

If "yes": Fever during attack: ☐ yes ☐ no Lymphnode swelling during attack: ☐ yes ☐ no

Peeling of limb after attack: ☐ yes ☐ no Able to work during attack: ☐ yes ☐ no

ADVERSE EVENTS *(only to be filled in if participant present for visit)*

Appearance of AEs / SAEs since last drug intake? ☐ yes ☐ no

*If "**yes**", please enter on the "Adverse Events" form at the end of this CRF.*

CONCOMITANT MEDICATION *(only to be filled in if participant present for visit)*

Change of medication since last visit? ☐ yes ☐ no

*If "**yes**", please enter on the "Concomitant Medication" form at the end of this CRF.*

INDIVIDUAL TREATMENT

Study drugs taken: ☐ yes ☐ no If "yes": **Treatment No.:** |__|__|

If "no", state reason: ☐ medical ☐ other (describe:_______________________________________)

PROBLEMS/ REMARKS

☐ yes ☐ no

If "yes", describe:____

**PodoLEDoxy**	Indiv. No.: |__|__|-|__|__|__|-|__|__|__| Randomization Code: |__|__|__|
Date of visit: |__|__| / |__|__| / |__|__|__|__| (dd/mm/yyyy)	**VISIT 3 - TREATMENT**<br>**Day 39**

PRESENCE

Participant present for visit: ☐ yes ☐ no

If "no", participant: ☐ traveled with drugs ☐ is absent but drugs were given to health worker/family member/neighbor and drug intake was confirmed

☐ is absent (state reason:________________________________)

ADLA QUESTIONNAIRE *(only to be filled in if participant present for visit)*

ADLA attack since last visit: ☐ yes ☐ no Ongoing?: ☐ yes ☐ no

If "yes": Fever during attack: ☐ yes ☐ no Lymphnode swelling during attack: ☐ yes ☐ no

Peeling of limb after attack: ☐ yes ☐ no Able to work during attack: ☐ yes ☐ no

ADVERSE EVENTS *(only to be filled in if participant present for visit)*

Appearance of AEs / SAEs since last drug intake? ☐ yes ☐ no

*If "**yes**", please enter on the "Adverse Events" form at the end of this CRF.*

CONCOMITANT MEDICATION *(only to be filled in if participant present for visit)*

Change of medication since last visit? ☐ yes ☐ no

*If "**yes**", please enter on the "Concomitant Medication" form at the end of this CRF.*

INDIVIDUAL TREATMENT

Study drugs taken: ☐ yes ☐ no If "yes": **Treatment No.:** |__|__|

If "no", state reason: ☐ medical ☐ other (describe:________________________________)

PROBLEMS/ REMARKS

☐ yes ☐ no

If "yes", describe:________________________________

PodoLEDoxy

Indiv. No.: |__|__|-|__|__|__|-|__|__|__|
Randomization Code: |__|__|__|

Date of visit: |__|__| / |__|__| / |__|__|__|__|
(dd/mm/yyyy)

VISIT 3 - TREATMENT
Day 40

PRESENCE

Participant present for visit: ☐ yes ☐ no
If "no", participant: ☐ traveled with drugs ☐ is absent but drugs were given to health worker/family member/neighbor and drug intake was confirmed
☐ is absent (state reason:____)

ADLA QUESTIONNAIRE *(only to be filled in if participant present for visit)*

ADLA attack since last visit: ☐ yes ☐ no Ongoing?: ☐ yes ☐ no
If "yes": Fever during attack: ☐ yes ☐ no Lymphnode swelling during attack: ☐ yes ☐ no
Peeling of limb after attack: ☐ yes ☐ no Able to work during attack: ☐ yes ☐ no

ADVERSE EVENTS *(only to be filled in if participant present for visit)*

Appearance of AEs / SAEs since last drug intake? ☐ yes ☐ no
*If "**yes**", please enter on the "Adverse Events" form at the end of this CRF.*

CONCOMITANT MEDICATION *(only to be filled in if participant present for visit)*

Change of medication since last visit? ☐ yes ☐ no
*If "**yes**", please enter on the "Concomitant Medication" form at the end of this CRF.*

INDIVIDUAL TREATMENT

Study drugs taken: ☐ yes ☐ no If "yes": **Treatment No.:** |__|__|
If "no", state reason: ☐ medical ☐ other (describe:_______________________________________)

PROBLEMS/ REMARKS

☐ yes ☐ no
If "yes", describe:____

**LEDoxy**	Indiv. No.: |__|__|-|__|__|__|-|__|__|__| Randomization Code: |__|__|__|
Date of visit: |__|__| / |__|__| / |__|__|__|__| (dd/mm/yyyy)	**VISIT 3 - TREATMENT**<br>**Day 41**

PRESENCE

Participant present for visit: ☐ yes ☐ no

If "no", participant: ☐ traveled with drugs ☐ is absent but drugs were given to health worker/family member/neighbor and drug intake was confirmed

☐ is absent (state reason:____)

ADLA QUESTIONNAIRE *(only to be filled in if participant present for visit)*

ADLA attack since last visit: ☐ yes ☐ no Ongoing?: ☐ yes ☐ no

If "yes": Fever during attack: ☐ yes ☐ no Lymphnode swelling during attack: ☐ yes ☐ no

Peeling of limb after attack: ☐ yes ☐ no Able to work during attack: ☐ yes ☐ no

ADVERSE EVENTS *(only to be filled in if participant present for visit)*

Appearance of AEs / SAEs since last drug intake? ☐ yes ☐ no

*If "**yes**", please enter on the "Adverse Events" form at the end of this CRF.*

CONCOMITANT MEDICATION *(only to be filled in if participant present for visit)*

Change of medication since last visit? ☐ yes ☐ no

*If "**yes**", please enter on the "Concomitant Medication" form at the end of this CRF.*

INDIVIDUAL TREATMENT

Study drugs taken: ☐ yes ☐ no If "yes": **Treatment No.: |__|__|**

If "no", state reason: ☐ medical ☐ other (describe:____)

PROBLEMS/ REMARKS

☐ yes ☐ no

If "yes", describe:____

**PodoLEDoxy**	Indiv. No.: |__|__|-|__|__|__|-|__|__|__| Randomization Code: |__|__|__|
Date of visit: |__|__| / |__|__| / |__|__|__|__| (dd/mm/yyyy)	**VISIT 3 - TREATMENT**<br>**Day 42**

PRESENCE

Participant present for visit: ☐ yes ☐ no

If “no”, participant: ☐ traveled with drugs ☐ is absent but drugs were given to health worker/family member/neighbor and drug intake was confirmed

☐ is absent (state reason:____)

ADLA QUESTIONNAIRE *(only to be filled in if participant present for visit)*

ADLA attack since last visit: ☐ yes ☐ no Ongoing?: ☐ yes ☐ no

If “yes”: Fever during attack: ☐ yes ☐ no Lymphnode swelling during attack: ☐ yes ☐ no

Peeling of limb after attack: ☐ yes ☐ no Able to work during attack: ☐ yes ☐ no

ADVERSE EVENTS *(only to be filled in if participant present for visit)*

Appearance of AEs / SAEs since last drug intake? ☐ yes ☐ no

*If “**yes**”, please enter on the “Adverse Events“ form at the end of this CRF.*

CONCOMITANT MEDICATION *(only to be filled in if participant present for visit)*

Change of medication since last visit? ☐ yes ☐ no

*If “**yes**”, please enter on the “Concomitant Medication“ form at the end of this CRF.*

INDIVIDUAL TREATMENT

Study drugs taken: ☐ yes ☐ no If “yes”: **Treatment No.:** |__|__| *If treatment is fulfilled (treatment No. 42) use “End of Treatment “ form (page 65) on the following day.*

If “no”, state reason: ☐ medical ☐ other (describe:______________________________________)

PREGNANCY TEST (URINE)

Sampling date: |__|__| / |__|__| / |__|__|__|__| (dd/mm/yyyy) Sampling time: |__|__| : |__|__| ☐ AM ☐ PM (h:min)

Date analyzed: |__|__| / |__|__| / |__|__|__|__| (dd/mm/yyyy) Time analyzed: |__|__| : |__|__| ☐ AM ☐ PM (h:min)

Result: ☐ neg. ☐ pos. ☐ not applicable (☐ ≥ 55 years, ☐ surgically sterilized)

LABORATORY ASSESSMENT (BLOOD) AFTER TREATMENT NO.42

Blood sample taken: ☐ yes ☐ no

If “no”, state reason: ☐ refused ☐ absent ☐ other (specify: ________________________________)

If “yes”: Sampling date: |__|__| / |__|__| / |__|__|__|__| (dd/mm/yyyy) Sampling time: |__|__| : |__|__| ☐ AM ☐ PM (h:min)

Please attach corresponding barcode label(s) on the “Laboratory documentation sheet”.
Please enter results on the „Laboratory assessment“ form at the end of this CRF.

URINE SAMPLING AFTER TREATMENT NO.42

Urine sample taken: ☐ yes ☐ no

If “no”, state reason: ☐ refused ☐ absent ☐ other (specify: ________________________________)

If “yes”: Sampling date: |__|__| / |__|__| / |__|__|__|__| Sampling time: |__|__| : |__|__| ☐ AM ☐ PM

(dd/mm/yyyy) (h:min)

Please attach corresponding barcode label(s) on the "Laboratory documentation sheet".

PROBLEMS/ REMARKS

☐ yes ☐ no If "yes", describe:____

PodoLEDoxy

Indiv. No.: |__|__|-|__|__|__|-|__|__|__|
Randomization Code: |__|__|__|

Date of visit: |__|__| / |__|__| / |__|__|__|__|
(dd/mm/yyyy)

VISIT 3 - TREATMENT
Day 43

PRESENCE *(range days – in case of 42 fullfilled treatments use "end of treatment" form on page 65)*

Participant present for visit: ☐ yes ☐ no
If "no", participant: ☐ traveled with drugs ☐ is absent but drugs were given to health worker/family member/neighbor and drug intake was confirmed
☐ is absent (state reason:____________________________________)

ADLA QUESTIONNAIRE *(only to be filled in if participant present for visit)*

ADLA attack since last visit: ☐ yes ☐ no Ongoing?: ☐ yes ☐ no
If "yes": Fever during attack: ☐ yes ☐ no Lymphnode swelling during attack: ☐ yes ☐ no
Peeling of limb after attack: ☐ yes ☐ no Able to work during attack: ☐ yes ☐ no

ADVERSE EVENTS *(only to be filled in if participant present for visit)*

Appearance of AEs / SAEs since last drug intake? ☐ yes ☐ no
*If "**yes**", please enter on the "Adverse Events" form at the end of this CRF.*

CONCOMITANT MEDICATION *(only to be filled in if participant present for visit)*

Change of medication since last visit? ☐ yes ☐ no
*If "**yes**", please enter on the "Concomitant Medication" form at the end of this CRF.*

INDIVIDUAL TREATMENT

Study drugs taken: ☐ yes ☐ no If "yes": **Treatment No.:** |__|__| *If treatment is fulfilled (treatment No. 42) use "End of Treatment " form (page 65) on the following day.*
If "no", state reason: ☐ medical ☐ other (describe:____________________________________)

PREGNANCY TEST (URINE) *(only to be done if not done on day 42)*

Sampling date: |__|__| / |__|__| / |__|__|__|__| (dd/mm/yyyy) Sampling time: |__|__| : |__|__| ☐ AM ☐ PM (h:min)
Date analyzed: |__|__| / |__|__| / |__|__|__|__| (dd/mm/yyyy) Time analyzed: |__|__| : |__|__| ☐ AM ☐ PM (h:min)
Result: ☐ neg. ☐ pos. ☐ not applicable (☐ ≥ 55 years, ☐ surgically sterilized)

LABORATORY ASSESSMENT (BLOOD) AFTER TREATMENT NO.42 *(only applicable if not done on day 42)*

Blood sample taken: ☐ yes ☐ no
If "no", state reason: ☐ refused ☐ absent ☐ other (specify: ____________________________________)
If "yes": Sampling date: |__|__| / |__|__| / |__|__|__|__| (dd/mm/yyyy) Sampling time: |__|__| : |__|__| ☐ AM ☐ PM (h:min)
Please attach corresponding barcode label(s) on the "Laboratory documentation sheet".
Please enter results on the „Laboratory assessment" form at the end of this CRF.

URINE SAMPLING AFTER TREATMENT NO.42 *(only applicable if not done on day 42)*

Urine sample taken: ☐ yes ☐ no
If "no", state reason: ☐ refused ☐ absent ☐ other (specify: ____________________________________)
If "yes": Sampling date: |__|__| / |__|__| / |__|__|__|__| Sampling time: |__|__| : |__|__| ☐ AM ☐ PM

Date of visit: |__|__| / |__|__| / |__|__|__|__| (dd/mm/yyyy)	**VISIT 3 - TREATMENT**<br>**Day 43**

(dd/mm/yyyy) (h:min)

Please attach corresponding barcode label(s) on the "Laboratory documentation sheet".

PROBLEMS/ REMARKS

☐ yes ☐ no If "yes", describe:____

**PodoLEDoxy**	Indiv. No.: |__|__|-|__|__|__|-|__|__|__| Randomization Code: |__|__|__|
Date of visit: |__|__| / |__|__| / |__|__|__|__| (dd/mm/yyyy)	**VISIT 3 - TREATMENT**<br>**Day 44**

PRESENCE *(range days – in case of 42 fullfilled treatments use "end of treatment" form on page 65)*

Participant present for visit: ☐ yes ☐ no

If "no", participant: ☐ traveled with drugs ☐ is absent but drugs were given to health worker/family member/neighbor and drug intake was confirmed

☐ is absent (state reason:____)

ADLA QUESTIONNAIRE *(only to be filled in if participant present for visit)*

ADLA attack since last visit: ☐ yes ☐ no Ongoing?: ☐ yes ☐ no

If "yes": Fever during attack: ☐ yes ☐ no Lymphnode swelling during attack: ☐ yes ☐ no

Peeling of limb after attack: ☐ yes ☐ no Able to work during attack: ☐ yes ☐ no

ADVERSE EVENTS *(only to be filled in if participant present for visit)*

Appearance of AEs / SAEs since last drug intake? ☐ yes ☐ no

*If "**yes**", please enter on the "Adverse Events" form at the end of this CRF.*

CONCOMITANT MEDICATION *(only to be filled in if participant present for visit)*

Change of medication since last visit? ☐ yes ☐ no

*If "**yes**", please enter on the "Concomitant Medication" form at the end of this CRF.*

INDIVIDUAL TREATMENT

Study drugs taken: ☐ yes ☐ no If "yes": **Treatment No.:** |__|__| *If treatment is fulfilled (treatment No. 42) use "End of Treatment " form (page 65) on the following day.*

If "no", state reason: ☐ medical ☐ other (describe:____)

PREGNANCY TEST (URINE) *(only to be done if not done on day 42 or 43)*

Sampling date: |__|__| / |__|__| / |__|__|__|__| (dd/mm/yyyy) Sampling time: |__|__| : |__|__| (h:min) ☐ AM ☐ PM

Date analyzed: |__|__| / |__|__| / |__|__|__|__| (dd/mm/yyyy) Time analyzed: |__|__| : |__|__| (h:min) ☐ AM ☐ PM

Result: ☐ neg. ☐ pos. ☐ not applicable (☐ ≥ 55 years, ☐ surgically sterilized)

LABORATORY ASSESSMENT (BLOOD) AFTER TREATMENT NO.42 *(only applicable if not done on day 42 or 43)*

Blood sample taken: ☐ yes ☐ no

If "no", state reason: ☐ refused ☐ absent ☐ other (specify: ____)

If "yes": Sampling date: |__|__| / |__|__| / |__|__|__|__| (dd/mm/yyyy) Sampling time: |__|__| : |__|__| (h:min) ☐ AM ☐ PM

Please attach corresponding barcode label(s) on the "Laboratory documentation sheet".
Please enter results on the „Laboratory assessment" form at the end of this CRF.

URINE SAMPLING AFTER TREATMENT NO.42 *(only applicable if not done on day 42 or 43)*

Urine sample taken: ☐ yes ☐ no

If "no", state reason: ☐ refused ☐ absent ☐ other (specify: ____)

If "yes": Sampling date: |__|__| / |__|__| / |__|__|__|__| (dd/mm/yyyy) Sampling time: |__|__| : |__|__| ☐ AM ☐ PM (h:min)

Please attach corresponding barcode label(s) on the "Laboratory documentation sheet".

PROBLEMS/ REMARKS

☐ yes ☐ no If "yes", describe:____

# PodoLEDoxy	Indiv. No.: |__|__|-|__|__|__|-|__|__|__| Randomization Code: |__|__|__|

Date of visit: |__|__| / |__|__| / |__|__|__|__| (dd/mm/yyyy)	**VISIT 3 - TREATMENT**<br>**Day 45**

PRESENCE *(range days – in case of 42 fullfilled treatments use "end of treatment" form on page 65)*

Participant present for visit: ☐ yes ☐ no
If "no", participant: ☐ traveled with drugs ☐ is absent but drugs were given to health worker/family member/neighbor and drug intake was confirmed
☐ is absent (state reason:____)

ADLA QUESTIONNAIRE *(only to be filled in if participant present for visit)*

ADLA attack since last visit: ☐ yes ☐ no Ongoing?: ☐ yes ☐ no
If "yes": Fever during attack: ☐ yes ☐ no Lymphnode swelling during attack: ☐ yes ☐ no
Peeling of limb after attack: ☐ yes ☐ no Able to work during attack: ☐ yes ☐ no

ADVERSE EVENTS *(only to be filled in if participant present for visit)*

Appearance of AEs / SAEs since last drug intake? ☐ yes ☐ no
*If "**yes**", please enter on the "Adverse Events" form at the end of this CRF.*

CONCOMITANT MEDICATION *(only to be filled in if participant present for visit)*

Change of medication since last visit? ☐ yes ☐ no
*If "**yes**", please enter on the "Concomitant Medication" form at the end of this CRF.*

INDIVIDUAL TREATMENT

Study drugs taken: ☐ yes ☐ no If "yes": **Treatment No.: |__|__|** *If treatment is fulfilled (treatment No. 42) use "End of Treatment " form (page 66) on the following day.*
If "no", state reason: ☐ medical ☐ other (describe:____)

LABORATORY ASSESSMENT (BLOOD) AFTER TREATMENT NO.42 *(only applicable if not done on day 42 - 44)*

Blood sample taken: ☐ yes ☐ no
If "no", state reason: ☐ refused ☐ absent ☐ other (specify: ________________________________)
If "yes": Sampling date: |__|__| / |__|__| / |__|__|__|__| Sampling time: |__|__| : |__|__| ☐ AM ☐ PM
(dd/mm/yyyy) (h:min)
Please attach corresponding barcode label(s) on the "Laboratory documentation sheet".
Please enter results on the „Laboratory assessment" form at the end of this CRF.

URINE SAMPLING AFTER TREATMENT NO.42 *(only applicable if not done on day 42 - 44)*

Urine sample taken: ☐ yes ☐ no
If "no", state reason: ☐ refused ☐ absent ☐ other (specify: ________________________________)
If "yes": Sampling date: |__|__| / |__|__| / |__|__|__|__| Sampling time: |__|__| : |__|__| ☐ AM ☐ PM
(dd/mm/yyyy) (h:min)
Please attach corresponding barcode label(s) on the "Laboratory documentation sheet".

PROBLEMS/ REMARKS

☐ yes ☐ no

If "yes", describe:____

**PodoLEDoxy**	Indiv. No.: |__|__|-|__|__|__|-|__|__|__| Randomization Code: |__|__|__|
Date of visit: |__|__| / |__|__| / |__|__|__|__| (dd/mm/yyyy)	**VISIT 3 - TREATMENT**<br>**Day 46**

PRESENCE *(range days – in case of 42 fullfilled treatments use "end of treatment" form on page 66)*

Participant present for visit: ☐ yes ☐ no

If "no", participant: ☐ traveled with drugs ☐ is absent but drugs were given to health worker/family member/neighbor and drug intake was confirmed

☐ is absent (state reason:____)

ADLA QUESTIONNAIRE *(only to be filled in if participant present for visit)*

ADLA attack since last visit: ☐ yes ☐ no Ongoing?: ☐ yes ☐ no

If "yes": Fever during attack: ☐ yes ☐ no Lymphnode swelling during attack: ☐ yes ☐ no

Peeling of limb after attack: ☐ yes ☐ no Able to work during attack: ☐ yes ☐ no

ADVERSE EVENTS *(only to be filled in if participant present for visit)*

Appearance of AEs / SAEs since last drug intake? ☐ yes ☐ no

*If "**yes**", please enter on the "Adverse Events" form at the end of this CRF.*

CONCOMITANT MEDICATION *(only to be filled in if participant present for visit)*

Change of medication since last visit? ☐ yes ☐ no

*If "**yes**", please enter on the "Concomitant Medication" form at the end of this CRF.*

INDIVIDUAL TREATMENT

Study drugs taken: ☐ yes ☐ no If "yes": **Treatment No.:** |__|__| *If treatment is fulfilled (treatment No. 42) use "End of Treatment " form (page 66) on the following day.*

If "no", state reason: ☐ medical ☐ other (describe:____)

LABORATORY ASSESSMENT (BLOOD) AFTER TREATMENT NO.42 *(only applicable if not done on day 42 - 45)*

Blood sample taken: ☐ yes ☐ no

If "no", state reason: ☐ refused ☐ absent ☐ other (specify: ______________________________________)

If "yes": Sampling date: |__|__| / |__|__| / |__|__|__|__| (dd/mm/yyyy) Sampling time: |__|__| : |__|__| (h:min) ☐ AM ☐ PM

Please attach corresponding barcode label(s) on the "Laboratory documentation sheet".
Please enter results on the „Laboratory assessment" form at the end of this CRF.

URINE SAMPLING AFTER TREATMENT NO.42 *(only applicable if not done on day 42 - 45)*

Urine sample taken: ☐ yes ☐ no

If "no", state reason: ☐ refused ☐ absent ☐ other (specify: ____________________________________)

If "yes": Sampling date: |__|__| / |__|__| / |__|__|__|__| (dd/mm/yyyy) Sampling time: |__|__| : |__|__| (h:min) ☐ AM ☐ PM

Please attach corresponding barcode label(s) on the "Laboratory documentation sheet".

PROBLEMS/ REMARKS

☐ yes ☐ no

If "yes", describe:____

**PodoLEDoxy**	Indiv. No.: |__|__|-|__|__|__|-|__|__|__| Randomization Code: |__|__|__|
Date of visit: |__|__| / |__|__| / |__|__|__|__| (dd/mm/yyyy)	**VISIT 3 - TREATMENT**<br>**Day 47**

PRESENCE *(range days – in case of 42 fullfilled treatments use "end of treatment" form on page 66)*

Participant present for visit: ☐ yes ☐ no

If "no", participant: ☐ traveled with drugs ☐ is absent but drugs were given to health worker/family member/neighbor and drug intake was confirmed

☐ is absent (state reason:____)

ADLA QUESTIONNAIRE *(only to be filled in if participant present for visit)*

ADLA attack since last visit: ☐ yes ☐ no Ongoing?: ☐ yes ☐ no

If "yes": Fever during attack: ☐ yes ☐ no Lymphnode swelling during attack: ☐ yes ☐ no

Peeling of limb after attack: ☐ yes ☐ no Able to work during attack: ☐ yes ☐ no

ADVERSE EVENTS *(only to be filled in if participant present for visit)*

Appearance of AEs / SAEs since last drug intake? ☐ yes ☐ no

*If "**yes**", please enter on the "Adverse Events" form at the end of this CRF.*

CONCOMITANT MEDICATION *(only to be filled in if participant present for visit)*

Change of medication since last visit? ☐ yes ☐ no

*If "**yes**", please enter on the "Concomitant Medication" form at the end of this CRF.*

INDIVIDUAL TREATMENT

Study drugs taken: ☐ yes ☐ no If "yes": **Treatment No.:** \|__\|__\| *If treatment is fulfilled (treatment No. 42) use "End of Treatment " form (page 66) on the following day.*

If "no", state reason: ☐ medical ☐ other (describe:____)

LABORATORY ASSESSMENT (BLOOD) AFTER TREATMENT NO.42 *(only applicable if not done on day 42 - 46)*

Blood sample taken: ☐ yes ☐ no

If "no", state reason: ☐ refused ☐ absent ☐ other (specify: _____________________________________)

If "yes": Sampling date: \|__\|__\| / \|__\|__\| / \|__\|__\|__\|__\| (dd/mm/yyyy) Sampling time: \|__\|__\| : \|__\|__\| (h:min) ☐ AM ☐ PM

Please attach corresponding barcode label(s) on the "Laboratory documentation sheet".
Please enter results on the „Laboratory assessment" form at the end of this CRF.

URINE SAMPLING AFTER TREATMENT NO.42 *(only applicable if not done on day 42 - 46)*

Urine sample taken: ☐ yes ☐ no

If "no", state reason: ☐ refused ☐ absent ☐ other (specify: _____________________________________)

If "yes": Sampling date: \|__\|__\| / \|__\|__\| / \|__\|__\|__\|__\| (dd/mm/yyyy) Sampling time: \|__\|__\| : \|__\|__\| (h:min) ☐ AM ☐ PM

Please attach corresponding barcode label(s) on the "Laboratory documentation sheet".

**PodoLEDoxy**	Indiv. No.: |__|__|-|__|__|__|-|__|__|__| Randomization Code: |__|__|__|
Date of visit: |__|__| / |__|__| / |__|__|__|__| (dd/mm/yyyy)	**VISIT 3 - TREATMENT**<br>**Day 47**

PROBLEMS/ REMARKS

☐ yes ☐ no

If "yes", describe:____

PodoLEDoxy

Indiv. No.: |__|__|-|__|__|__|-|__|__|__|
Randomization Code: |__|__|__|

Date of visit: |__|__| / |__|__| / |__|__|__|__|
(dd/mm/yyyy)

VISIT 3 - TREATMENT
Day 48

PRESENCE *(range days – in case of 42 fullfilled treatments use "end of treatment" form on page 66)*

Participant present for visit: ☐ yes ☐ no
If "no", participant: ☐ traveled with drugs ☐ is absent but drugs were given to health worker/family member/neighbor and drug intake was confirmed
☐ is absent (state reason:____)

ADLA QUESTIONNAIRE *(only to be filled in if participant present for visit)*

ADLA attack since last visit: ☐ yes ☐ no Ongoing?: ☐ yes ☐ no
If "yes": Fever during attack: ☐ yes ☐ no Lymphnode swelling during attack: ☐ yes ☐ no
Peeling of limb after attack: ☐ yes ☐ no Able to work during attack: ☐ yes ☐ no

ADVERSE EVENTS *(only to be filled in if participant present for visit)*

Appearance of AEs / SAEs since last drug intake? ☐ yes ☐ no
*If "**yes**", please enter on the "Adverse Events" form at the end of this CRF.*

CONCOMITANT MEDICATION *(only to be filled in if participant present for visit)*

Change of medication since last visit? ☐ yes ☐ no
*If "**yes**", please enter on the "Concomitant Medication" form at the end of this CRF.*

INDIVIDUAL TREATMENT

Study drugs taken: ☐ yes ☐ no If "yes": **Treatment No.:** |__|__| *If treatment is fulfilled (treatment No. 42) use "End of Treatment " form (page 66) on the following day.*
If "no", state reason: ☐ medical ☐ other (describe:_______________________________________)

LABORATORY ASSESSMENT (BLOOD) AFTER TREATMENT NO.42 *(only applicable if not done on day 42 - 47)*

Blood sample taken: ☐ yes ☐ no
If "no", state reason: ☐ refused ☐ absent ☐ other (specify: ________________________________)
If "yes": Sampling date: |__|__| / |__|__| / |__|__|__|__| (dd/mm/yyyy) Sampling time: |__|__| : |__|__| (h:min) ☐ AM ☐ PM
Please attach corresponding barcode label(s) on the "Laboratory documentation sheet".
Please enter results on the „Laboratory assessment" form at the end of this CRF.

URINE SAMPLING AFTER TREATMENT NO.42 *(only applicable if not done on day 42 - 47)*

Urine sample taken: ☐ yes ☐ no
If "no", state reason: ☐ refused ☐ absent ☐ other (specify: ________________________________)
If "yes": Sampling date: |__|__| / |__|__| / |__|__|__|__| (dd/mm/yyyy) Sampling time: |__|__| : |__|__| (h:min) ☐ AM ☐ PM
Please attach corresponding barcode label(s) on the "Laboratory documentation sheet".

PodoLEDoxy

Indiv. No.: |__|__|-|__|__|__|-|__|__|__|
Randomization Code: |__|__|__|

Date of visit: |__|__| / |__|__| / |__|__|__|__|
(dd/mm/yyyy)

VISIT 3 - TREATMENT
Day 48

PROBLEMS/ REMARKS

☐ yes ☐ no

If "yes", describe:____

**PodoLEDoxy**	Indiv. No.: |__|__|-|__|__|__|-|__|__|__| Randomization Code: |__|__|__|
Date of visit: |__|__| / |__|__| / |__|__|__|__| (dd/mm/yyyy)	**VISIT 3 - TREATMENT**<br>**Day 49**

PRESENCE *(range days – in case of 42 fullfilled treatments use "end of treatment" form on page 66)*

Participant present for visit: ☐ yes ☐ no

If "no", participant: ☐ traveled with drugs ☐ is absent but drugs were given to health worker/family member/neighbor and drug intake was confirmed

☐ is absent (state reason:____)

ADLA QUESTIONNAIRE *(only to be filled in if participant present for visit)*

ADLA attack since last visit: ☐ yes ☐ no Ongoing?: ☐ yes ☐ no

If "yes": Fever during attack: ☐ yes ☐ no Lymphnode swelling during attack: ☐ yes ☐ no

Peeling of limb after attack: ☐ yes ☐ no Able to work during attack: ☐ yes ☐ no

ADVERSE EVENTS *(only to be filled in if participant present for visit)*

Appearance of AEs / SAEs since last drug intake? ☐ yes ☐ no

*If "**yes**", please enter on the "Adverse Events" form at the end of this CRF.*

CONCOMITANT MEDICATION *(only to be filled in if participant present for visit)*

Change of medication since last visit? ☐ yes ☐ no

*If "**yes**", please enter on the "Concomitant Medication" form at the end of this CRF.*

INDIVIDUAL TREATMENT

Study drugs taken: ☐ yes ☐ no If "yes": **Treatment No.:** \|__\|__\| *If treatment is fulfilled (treatment No. 42) use "End of Treatment " form (page 66) on the following day.*

If "no", state reason: ☐ medical ☐ other (describe:____)

LABORATORY ASSESSMENT (BLOOD) AFTER TREATMENT NO.42 *(only applicable if not done on day 42 - 48)*

Blood sample taken: ☐ yes ☐ no

If "no", state reason: ☐ refused ☐ absent ☐ other (specify: ____________________________________)

If "yes": Sampling date: \|__\|__\| / \|__\|__\| / \|__\|__\|__\|__\| (dd/mm/yyyy) Sampling time: \|__\|__\| : \|__\|__\| (h:min) ☐ AM ☐ PM

Please attach corresponding barcode label(s) on the "Laboratory documentation sheet".
Please enter results on the „Laboratory assessment" form at the end of this CRF.

URINE SAMPLING AFTER TREATMENT NO.42 *(only applicable if not done on day 42 - 48)*

Urine sample taken: ☐ yes ☐ no

If "no", state reason: ☐ refused ☐ absent ☐ other (specify: __________________________________)

If "yes": Sampling date: \|__\|__\| / \|__\|__\| / \|__\|__\|__\|__\| (dd/mm/yyyy) Sampling time: \|__\|__\| : \|__\|__\| (h:min) ☐ AM ☐ PM

Please attach corresponding barcode label(s) on the "Laboratory documentation sheet".

PROBLEMS/ REMARKS

☐ yes ☐ no

If "yes", describe:____

**PodoLEDoxy**	Indiv. No.: |__|__|-|__|__|__|-|__|__|__| Randomization Code: |__|__|__|
Date of visit: |__|__| / |__|__| / |__|__|__|__| (dd/mm/yyyy)	**VISIT 3**<br>**END OF TREATMENT**

PRESENCE

Participant present for visit: ☐ yes ☐ no

If "no", state reason: ______________________________

ADLA QUESTIONNAIRE *(only to be filled in if participant present for visit)*

ADLA attack since last visit: ☐ yes ☐ no Ongoing?: ☐ yes ☐ no

If "yes": Fever during attack: ☐ yes ☐ no Lymphnode swelling during attack: ☐ yes ☐ no

Peeling of limb after attack: ☐ yes ☐ no Able to work during attack: ☐ yes ☐ no

ADVERSE EVENTS *(only to be filled in if participant present for visit)*

Appearance of AEs / SAEs since last drug intake? ☐ yes ☐ no

*If "**yes**", please enter on the "Adverse Events" form at the end of this CRF.*

CONCOMITANT MEDICATION *(only to be filled in if participant present for visit)*

Change of medication since last visit? ☐ yes ☐ no

*If "**yes**", please enter on the "Concomitant Medication" form at the end of this CRF.*

PREGNANCY TEST (URINE) *(only to be done if not done on day 42 - 44)*

Sampling date: |__|__| / |__|__| / |__|__|__|__| (dd/mm/yyyy) Sampling time: |__|__| : |__|__| ☐ AM ☐ PM (h:min)

Date analyzed: |__|__| / |__|__| / |__|__|__|__| (dd/mm/yyyy) Time analyzed: |__|__| : |__|__| ☐ AM ☐ PM (h:min)

Result: ☐ neg. ☐ pos. ☐ not applicable (☐ ≥ 55 years, ☐ surgically sterilized)

LABORATORY ASSESSMENT (BLOOD) AFTER TREATMENT NO.42 *(only applicable if not done on day 42 - 49)*

Blood sample taken: ☐ yes ☐ no

If "no", state reason: ☐ refused ☐ absent ☐ other (specify: ______________________________)

If "yes": Sampling date: |__|__| / |__|__| / |__|__|__|__| (dd/mm/yyyy) Sampling time: |__|__| : |__|__| ☐ AM ☐ PM (h:min)

Please attach corresponding barcode label(s) on the "Laboratory documentation sheet".
Please enter results on the „Laboratory assessment" form at the end of this CRF.

URINE SAMPLING AFTER TREATMENT NO.42 *(only applicable if not done on day 42 - 49)*

Urine sample taken: ☐ yes ☐ no

If "no", state reason: ☐ refused ☐ absent ☐ other (specify: ______________________________)

If "yes": Sampling date: |__|__| / |__|__| / |__|__|__|__| (dd/mm/yyyy) Sampling time: |__|__| : |__|__| ☐ AM ☐ PM (h:min)

Please attach corresponding barcode label(s) on the "Laboratory documentation sheet".

VISIT 3 – TREATMENT – INVESTIGATOR'S STATEMENT

I confirm that I have carefully examined all entries on the Visit 3 – Treatment – Case Report Form pages for this subject. All information entered by myself or my colleagues is, to the best of my knowledge, correct as of the date below.

**PodoLEDoxy**	Indiv. No.: |__|__|-|__|__|__|-|__|__|__| Randomization Code: |__|__|__|
Date of visit: |__|__| / |__|__| / |__|__|__|__| (dd/mm/yyyy)	**VISIT 3**<br>**END OF TREATMENT**

Date: |__|__| / |__|__| / |__|__|__|__|

(dd/mm/yyyy)

____

(Investigator`s signature)

____

(Investigator`s name print)

PodoLEDoxy

Indiv. No.: |__|__|-|__|__|__|-|__|__|__|
Randomization Code: |__|__|__|

Date of visit: |__|__| / |__|__| / |__|__|__|__| (dd/mm/yyyy)

VISIT 4
2 MONTHS FOLLOW-UP

PRESENCE

Participant present for visit: ☐ yes ☐ no
If "no", state reason: ☐ refused ☐ traveled ☐ moved ☐ not traceable ☐ other
If "other", describe:____________________

ADVERSE EVENTS

Appearance of AEs / SAEs since last vist? ☐ yes ☐ no
*If "**yes**", please enter on the "Adverse Events" form at the end of this CRF.*

CONCOMITANT MEDICATION

Change of medication since last visit? ☐ yes ☐ no
*If "**yes**", please enter on the "Concomitant Medication" form at the end of this CRF.*

ADLA QUESTIONNAIRE

ADLA attacks since last visit: ☐ yes ☐ no If "yes": Start date of last attack: |__|__| / |__|__|__|__| (mm/yyyy)
Ongoing?: ☐ yes ☐ no If "not ongoing": Duration of last attack: |__|__|__| (days)
Fever during last attack: ☐ yes ☐ no Lymphnode swelling during last attack: ☐ yes ☐ no
Peeling of limb after last attack: ☐ yes ☐ no Able to work during last attack: ☐ yes ☐ no
Occupation (specify:____________________)
Intensity of work: ☐ light ☐ heavy ☐ not working ☐ other (specify:____________________)
Number of attacks since last visit: |__|__|__| Duration of attacks (average since last visit): |__|__|__| (days)

PREGNANCY TEST (URINE)

Sampling date: |__|__| / |__|__| / |__|__|__|__| (dd/mm/yyyy) Sampling time: |__|__| : |__|__| (h:min) ☐ AM ☐ PM
Date analyzed: |__|__| / |__|__| / |__|__|__|__| (dd/mm/yyyy) Time analyzed: |__|__| : |__|__| (h:min) ☐ AM ☐ PM
Result: ☐ neg. ☐ pos. ☐ not applicable (☐ ≥ 55 years, ☐ surgically sterilized)

REMARKS - VISIT 4 - 2 MONTHS FOLLOW-UP

VISIT 4 - 2 MONTHS FOLLOW-UP - INVESTIGATOR'S STATEMENT

I confirm that I have carefully examined all entries on the Visit 4 Case Report Form pages for this subject. All information entered by myself or my colleagues is, to the best of my knowledge, correct as of the date below.

Date: |__|__| / |__|__| / |__|__|__|__|
(dd/mm/yyyy)

____
(Investigator`s signature)

____
(Investigator`s name print)

PodoLEDoxy

Indiv. No.: |__|__|-|__|__|__|-|__|__|__|
Randomization Code: |__|__|__|

Date of visit: |__|__| / |__|__| / |__|__|__|__| (dd/mm/yyyy)

VISIT 5
4 MONTHS FOLLOW-UP

PRESENCE

Participant present for visit: ☐ yes ☐ no
If "no", state reason: ☐ refused ☐ traveled ☐ moved ☐ not traceable ☐ other
If "other", describe:____

ADVERSE EVENTS

Appearance of AEs / SAEs since last vist? ☐ yes ☐ no
*If "**yes**", please enter on the "Adverse Events" form at the end of this CRF.*

CONCOMITANT MEDICATION

Change of medication since last visit? ☐ yes ☐ no
*If "**yes**", please enter on the "Concomitant Medication" form at the end of this CRF.*

ADLA QUESTIONNAIRE

ADLA attacks since last visit: ☐ yes ☐ no If "yes": Start date of last attack: |__|__| / |__|__|__|__| (mm/yyyy)
Ongoing?: ☐ yes ☐ no If "not ongoing": Duration of last attack: |__|__|__| (days)
Fever during last attack: ☐ yes ☐ no Lymphnode swelling during last attack: ☐ yes ☐ no
Peeling of limb after last attack: ☐ yes ☐ no Able to work during last attack: ☐ yes ☐ no
Occupation (specify:__________________________________)
Intensity of work: ☐ light ☐ heavy ☐ not working ☐ other (specify:__________________________________)
Number of attacks since last visit: |__|__|__| Duration of attacks (average since last visit): |__|__|__| (days)

HYGIENE STATUS

Hygiene assessment done: ☐ yes ☐ no If "yes", date: |__|__| / |__|__| / |__|__|__|__| (dd/mm/yyyy)

LYMPHEDEMA MANAGEMENT TRAINING

Training has been given: ☐ yes ☐ no If "yes", date: |__|__| / |__|__| / |__|__|__|__| (dd/mm/yyyy)

REMARKS - VISIT 5 - 4 MONTHS FOLLOW-UP

VISIT 5 - 4 MONTHS FOLLOW-UP - INVESTIGATOR'S STATEMENT

I confirm that I have carefully examined all entries on the Visit 5 Case Report Form pages for this subject. All information entered by myself or my colleagues is, to the best of my knowledge, correct as of the date below.

Date: |__|__| / |__|__| / |__|__|__|__|

(dd/mm/yyyy)

____

(Investigator`s signature)

____

(Investigator`s name print)

**PodoLEDoxy**	Indiv. No.: |__|__|-|__|__|__|-|__|__|__| Randomization Code: |__|__|__|
Date of visit: |__|__| / |__|__| / |__|__|__|__| (dd/mm/yyyy)	**VISIT 6**<br>**6 MONTHS FOLLOW-UP**

PRESENCE

Participant present for visit: ☐ yes ☐ no

If "no", state reason: ☐ refused ☐ traveled ☐ moved ☐ not traceable ☐ other

If "other", describe:____

CONCOMITANT MEDICATION

Change of medication since last visit? ☐ yes ☐ no

*If "**yes**", please enter on the „Concomitant Medication" form at the end of this CRF.*

VITAL SIGNS

Blood pressure: |__|__|__| / |__|__|__| mmHg (systolic / diastolic) Hypertension: ☐ yes ☐ no

If "hypertensive": Known hypertension: ☐ yes ☐ no Hypertensive medication: ☐ yes ☐ no

*If "**yes**", enter on the "Concomitant Medication" form at the end of this CRF*

Pulse rate: |__|__|__| bpm Temperature: |__|__|.|__|°C Weight: |__|__|__|.|__| kg

CLINICAL PHOTOGRAPHS

Clinical photographs taken: ☐ yes ☐ no

If "yes", Photograph References/No.:____

LYMPHEDEMA STAGING

Stage	Description	Right Leg	Left Leg
0	No abnormality	☐	☐
1	Swelling is reversible (goes away) overnight	☐	☐
2	Swelling is not reversible (doesn't go away)	☐	☐
3	Presence of shallow skin folds<br>(base of fold can be seen by straightening the ankle)	☐	☐
4	Presence of skin knobs	☐	☐
5	Presence of deep skin folds(base of fold can only be seen if edges are actively separated by hand)	☐	☐
6	Presence of "mossy lesions"<br>Warty looking epidermal skin lesions.	☐	☐
7	Unable to care for self or perform daily activities	☐	☐

**PodoLEDoxy**	Indiv. No.: |__|__|-|__|__|__|-|__|__|__| Randomization Code: |__|__|__|

Date of visit: |__|__| / |__|__| / |__|__|__|__| (dd/mm/yyyy)	**VISIT 6**<br>**6 MONTHS FOLLOW-UP**

ADLA QUESTIONNAIRE

ADLA attacks since last visit: ☐ yes ☐ no If "yes": Start date of last attack: |__|__| / |__|__|__|__| (mm/yyyy)

Ongoing?: ☐ yes ☐ no If "not ongoing": Duration of last attack: |__|__|__| (days)

Fever during last attack: ☐ yes ☐ no Lymphnode swelling during last attack: ☐ yes ☐ no

Peeling of limb after last attack: ☐ yes ☐ no Able to work during last attack: ☐ yes ☐ no

Occupation (specify:____________________________________)

Intensity of work: ☐ light ☐ heavy ☐ not working ☐ other (specify:_____________________)

Number of attacks since last visit: |__|__|__| Duration of attacks (average since last visit): |__|__|__| (days)

HYGIENE STATUS

Hygiene assessment done: ☐ yes ☐ no

If "yes", date: |__|__| / |__|__| / |__|__|__|__| (dd/mm/yyyy)

LYMPHEDEMA MANAGEMENT TRAINING

Training has been given: ☐ yes ☐ no

If "yes", date: |__|__| / |__|__| / |__|__|__|__| (dd/mm/yyyy)

CIRCUMFERENCE MEASUREMENT OF LYMPHEDEMA -TAPE

Examiner: ______________________ Time of baseline measurement: |__|__|:|__|__| (h:min) ☐ AM ☐ PM

Date: |__|__| / |__|__| / |__|__|__|__| (dd/mm/yyyy) Time of measurement**:** |__|__|:|__|__| (h:min) ☐ AM ☐ PM

Measurements MUST be taken within 2 hours of the baseline time of measurement. If >2 hours before or after the baseline time, patient must return within the appropriate time window.

Main activity in the previous two hours before measurement: ☐ sitting ☐ walking ☐ standing ☐ farming ☐ fishing ☐ cooking ☐ driving ☐ child care ☐ other:_________________________

	**Circumferences of left leg**		**Circumferences of right leg**	
	1st measurement	2nd measurement	1st measurement	2nd measurement
A (10cm from tip of 1st toe)	|__|__|.|__| cm	|__|__|.|__| cm	|__|__|.|__| cm	|__|__|.|__| cm

B (12cm)	|__|__|.|__| cm	|__|__|.|__| cm	|__|__|.|__| cm	|__|__|.|__| cm
C (20 cm)	|__|__|.|__| cm	|__|__|.|__| cm	|__|__|.|__| cm	|__|__|.|__| cm
D (30cm)	|__|__|.|__| cm	|__|__|.|__| cm	|__|__|.|__| cm	|__|__|.|__| cm

**PodoLEDoxy**	Indiv. No.: |__|__|-|__|__|__|-|__|__|__| Randomization Code: |__|__|__|
Date of visit: |__|__| / |__|__| / |__|__|__|__| (dd/mm/yyyy)	**VISIT 6**<br>**6 MONTHS FOLLOW-UP**

CIRCUMFERENCE MEASUREMENT OF LYMPHEDEMA –LYMPHATECH® SCANNER

Examiner: ________________________ Time of baseline measurement: |__|__|:|__|__| ☐ AM ☐ PM

(h:min)

Date: |__|__| / |__|__| / |__|__|__|__| Time of measurement: |__|__|:|__|__| ☐ AM ☐ PM

(dd/mm/yyyy) (h:min)

Measurements MUST be taken within 2 hours of the baseline time of measurement. If >2 hours before or after the baseline time, patient must return within the appropriate time window.

Main activity in the previous two hours before measurement: ☐ sitting ☐ walking ☐ standing ☐ farming ☐ fishing ☐ cooking ☐ driving ☐ child care ☐ other:___________________________

	**Circumferences of left leg**		**Circumferences of right leg**	
	1st measurement	2nd measurement	1st measurement	2nd measurement
A (12 cm)	|__|__|.|__| cm	|__|__|.|__| cm	|__|__|.|__| cm	|__|__|.|__| cm
B (20 cm)	|__|__|.|__| cm	|__|__|.|__| cm	|__|__|.|__| cm	|__|__|.|__| cm
C (30 cm)	|__|__|.|__| cm	|__|__|.|__| cm	|__|__|.|__| cm	|__|__|.|__| cm

VOLUME OF LYMPHEDEMA –LYMPHATECH® SCANNER

Examiner: ________________________ Time of baseline measurement: |__|__|:|__|__| ☐ AM ☐ PM

(h:min)

Date: |__|__| / |__|__| / |__|__|__|__| Time of measurement: |__|__|:|__|__| ☐ AM ☐ PM

(dd/mm/yyyy) (h:min)

Measurements MUST be taken within 2 hours of the baseline time of measurement. If >2 hours before or after the baseline time, patient must return within the appropriate time window.

Main activity in the previous two hours before measurement: ☐ sitting ☐ walking ☐ standing ☐ farming ☐ fishing ☐ cooking ☐ driving ☐ child care ☐ other:___________________________

	1st measurement	2nd measurement
Volume of left leg	|__|__|__|__|__| ml	|__|__|__|__|__| ml
Volume of right leg	|__|__|__|__|__| ml	|__|__|__|__|__| ml

LymphaTech References/No.:____ Photograph

**PodoLEDoxy**	Indiv. No.: |__|__|-|__|__|__|-|__|__|__| Randomization Code: |__|__|__|
Date of visit: |__|__| / |__|__| / |__|__|__|__| (dd/mm/yyyy)	**VISIT 6**<br>**6 MONTHS FOLLOW-UP**

ULTRASOUND ASSESSMENT OF SKIN THICKNESS AT BOTH ANKLES

Examiner: ________________________ Time of baseline measurement: |__|__|:|__|__| ☐ AM ☐ PM

(h:min)

Date: |__|__| / |__|__| / |__|__|__|__| Time of measurement: |__|__|:|__|__| ☐ AM ☐ PM

(dd/mm/yyyy) (h:min)

Measurements MUST be taken within 2 hours of the baseline time of measurement. If >2 hours before or after the baseline time, patient must return within the appropriate time window.

Main activity in the previous two hours before measurement: ☐ sitting ☐ walking ☐ standing ☐ farming ☐ fishing ☐ cooking ☐ driving ☐ child care ☐ other:___________________________

Presence of acute conditions:

Pregnancy: ☐ yes ☐ no

Febrile illness: ☐ yes ☐ no

Heart condition requiring diuretics: ☐ yes ☐ no

Adenolymphangitis attack: ☐ yes ☐ no if "yes": ☐ left leg ☐ right leg

Recent leg trauma: ☐ yes ☐ no if "yes": ☐ left leg ☐ right leg:

Other: ☐ yes ☐ no if "yes", describe:______________________________

Has the patient been bed-bound more than one day within the past week? ☐ yes ☐ no

If "yes", describe:____

Are open wounds present? ☐ yes ☐ no

If "yes", ☐ left leg/acute ☐ left leg/chronic ☐ right leg/acute ☐ right leg/chronic

Even when open wounds are present, ultrasound should be done as long as it does not require placing the ultrasound probe within the field of the wound. If measurement cannot be done without placing the probe in the wound, skip that location and write "wound" in the notes section below.

Skin measurement

	1st measurement	2nd measurement	Notes
Left lateral malleolus	|__|.|__|__| cm	|__|.|__|__| cm	
Left medial malleolus	|__|.|__|__| cm	|__|.|__|__| cm	
Right lateral malleolus	|__|.|__|__| cm	|__|.|__|__| cm	
Right medial malleolus	|__|.|__|__| cm	|__|.|__|__| cm	

Ultrasound Photograph References/No.:____

LABORATORY ASSESSMENT (BLOOD)

Blood sample taken: ☐ yes ☐ no

If “no”, state reason: ☐ refused ☐ absent ☐ other (specify: ________________________________)

If “yes”: Sampling date: |__|__| / |__|__| / |__|__|__|__| Sampling time: |__|__| : |__|__| ☐ AM ☐ PM

(dd/mm/yyyy) (h:min)

Please attach corresponding barcode label(s) on the “Laboratory documentation sheet”.
Please enter results on the „Laboratory assessment“ form at the end of this CRF.

URINE SAMPLING

Urine sample taken: ☐ yes ☐ no

If “no”, state reason: ☐ refused ☐ absent ☐ other (specify: ________________________________)

If “yes”: Sampling date: |__|__| / |__|__| / |__|__|__|__| Sampling time: |__|__| : |__|__| ☐ AM ☐ PM

(dd/mm/yyyy) (h:min)

Please attach corresponding barcode label(s) on the “Laboratory documentation sheet”.

SALIVA SAMPLING

Saliva sample taken: ☐ yes ☐ no

If “no”, state reason: ☐ refused ☐ absent ☐ other (specify: ________________________________)

If “yes”: Sampling date: |__|__| / |__|__| / |__|__|__|__| Sampling time: |__|__| : |__|__| ☐ AM ☐ PM

(dd/mm/yyyy) (h:min)

Please attach corresponding barcode label(s) on the “Laboratory documentation sheet”.

PREGNANCY TEST

Sampling date: |__|__| / |__|__| / |__|__|__|__| Sampling time: |__|__| : |__|__| ☐ AM ☐ PM

(dd/mm/yyyy) (h:min)

Date analyzed: |__|__| / |__|__| / |__|__|__|__| Time analyzed: |__|__| : |__|__| ☐ AM ☐ PM

(dd/mm/yyyy) (h:min)

Result: ☐ neg. ☐ pos. ☐ not applicable (☐ ≥ 55 years, ☐ surgically sterilized)

REMARKS - VISIT 6 - 6 MONTHS FOLLOW-UP

VISIT 6 - 6 MONTHS FOLLOW-UP - INVESTIGATOR'S STATEMENT

I confirm that I have carefully examined all entries on the Visit 6 Case Report Form pages for this subject. All information entered by myself or my colleagues is, to the best of my knowledge, correct as of the date below.

Date: |__|__| / |__|__| / |__|__|__|__|

(dd/mm/yyyy)

____

(Investigator`s signature)

____

(Investigator`s name print)

**PodoLEDoxy**	Indiv. No.: |__|__|-|__|__|__|-|__|__|__| Randomization Code: |__|__|__|
Date of visit: |__|__| / |__|__| / |__|__|__|__| (dd/mm/yyyy)	**VISIT 7**<br>**8 MONTHS FOLLOW-UP**

PRESENCE

Participant present for visit: ☐ yes ☐ no

If "no", state reason: ☐ refused ☐ traveled ☐ moved ☐ not traceable ☐ other

If "other", describe:____

CONCOMITANT MEDICATION

Change of medication since last visit? ☐ yes ☐ no

*If "**yes**", please enter on the "Concomitant Medication" form at the end of this CRF.*

ADLA QUESTIONNAIRE

ADLA attacks since last visit: ☐ yes ☐ no If "yes": Start date of last attack: |__|__| / |__|__|__|__|
(mm/yyyy)

Ongoing?: ☐ yes ☐ no If "not ongoing": Duration of last attack: |__|__|__|
(days)

Fever during last attack: ☐ yes ☐ no Lymphnode swelling during last attack: ☐ yes ☐ no

Peeling of limb after last attack: ☐ yes ☐ no Able to work during last attack: ☐ yes ☐ no

Occupation (specify:_____________________________________)

Intensity of work: ☐ light ☐ heavy ☐ not working ☐ other (specify:______________________)

Number of attacks since last visit: |__|__|__| Duration of attacks (average since last visit): |__|__|__|

(days)

REMARKS - VISIT 7 - 8 MONTHS FOLLOW-UP

VISIT 7 - 8 MONTHS FOLLOW-UP - INVESTIGATOR'S STATEMENT

I confirm that I have carefully examined all entries on the Visit 7 Case Report Form pages for this subject. All information entered by myself or my colleagues is, to the best of my knowledge, correct as of the date below.

Date: |__|__| / |__|__| / |__|__|__|__|

(dd/mm/yyyy)

____
(Investigator`s signature)

____
(Investigator`s name print)

PodoLEDoxy

Indiv. No.: |__|__|-|__|__|__|-|__|__|__|
Randomization Code: |__|__|__|

Date of visit: |__|__| / |__|__| / |__|__|__|__|
(dd/mm/yyyy)

VISIT 8
10 MONTHS FOLLOW-UP

PRESENCE

Participant present for visit: ☐ yes ☐ no
If "no", state reason: ☐ refused ☐ traveled ☐ moved ☐ not traceable ☐ other
If "other", describe:____

CONCOMITANT MEDICATION

Change of medication since last visit? ☐ yes ☐ no
*If "**yes**", please enter on the "Concomitant Medication" form at the end of this CRF.*

ADLA QUESTIONNAIRE

ADLA attacks since last visit: ☐ yes ☐ no If "yes": Start date of last attack: |__|__| / |__|__|__|__|
(mm/yyyy)

Ongoing?: ☐ yes ☐ no If "not ongoing": Duration of last attack: |__|__|__|
(days)

Fever during last attack: ☐ yes ☐ no Lymphnode swelling during last attack: ☐ yes ☐ no
Peeling of limb after last attack: ☐ yes ☐ no Able to work during last attack: ☐ yes ☐ no

Occupation (specify:_____________________________________)
Intensity of work: ☐ light ☐ heavy ☐ not working ☐ other (specify:______________________)
Number of attacks since last visit: |__|__|__| Duration of attacks (average since last visit): |__|__|__| (days)

REMARKS - VISIT 8 - 10 MONTHS FOLLOW-UP

VISIT 8 - 10 MONTHS FOLLOW-UP - INVESTIGATOR'S STATEMENT

I confirm that I have carefully examined all entries on the Visit 8 Case Report Form pages for this subject. All information entered by myself or my colleagues is, to the best of my knowledge, correct as of the date below.

Date: |__|__| / |__|__| / |__|__|__|__|
(dd/mm/yyyy)

____
(Investigator`s signature)

____
(Investigator`s name print)

**PodoPodoLEDoxy**	Indiv. No.: |__|__|-|__|__|__|-|__|__|__| Randomization Code: |__|__|
Date of visit: |__|__| / |__|__| / |__|__|__|__| (dd/mm/yyyy)	**VISIT 9<br>12 MONTHS FOLLOW-UP**

PRESENCE

Participant present for visit: ☐ yes ☐ no

If "no", state reason: ☐ refused ☐ traveled ☐ moved ☐ not traceable ☐ other

If "other", describe:____

CONCOMITANT MEDICATION

Change of medication since last visit? ☐ yes ☐ no

*If "**yes**", please enter on the „Concomitant Medication" form at the end of this CRF.*

VITAL SIGNS

Blood pressure: |__|__|__| / |__|__|__| mmHg Hypertension: ☐ yes ☐ no
systolic diastolic

If "hypertensive": Known hypertension: ☐ yes ☐ no Hypertensive medication: ☐ yes ☐ no

*If "**yes**", enter on the "Concomitant Medication" form at the end of this CRF*

Pulse rate: |__|__|__| bpm Temperature: |__|__|.|__|°C Weight: |__|__|__|.|__| kg

CLINICAL PHOTOGRAPHS

Clinical photographs taken: ☐ yes ☐ no

If "yes", Photograph References/No.:____

LYMPHEDEMA STAGING

Stage	Description	Right Leg	Left Leg
0	No abnormality	☐	☐
1	Swelling is reversible (goes away) overnight	☐	☐
2	Swelling is not reversible (doesn't go away)	☐	☐
3	Presence of shallow skin folds<br>(base of fold can be seen by straightening the ankle)	☐	☐
4	Presence of skin knobs	☐	☐
5	Presence of deep skin folds (base of fold can only be seen if edges are actively separated by hand)	☐	☐
6	Presence of "mossy lesions"<br>Warty looking epidermal skin lesions.	☐	☐
7	Unable to care for self or perform daily activities	☐	☐

**PodoPodoLEDoxy**	Indiv. No.: |__|__|-|__|__|__|-|__|__|__| Randomization Code: |__|__|__|
Date of visit: |__|__| / |__|__| / |__|__|__|__| (dd/mm/yyyy)	**VISIT 9**<br>**12 MONTHS FOLLOW-UP**

ADLA QUESTIONNAIRE

ADLA attacks since last visit: ☐ yes ☐ no If "yes": Start date of last attack: |__|__| / |__|__|__|__| (mm/yyyy)

Ongoing?: ☐ yes ☐ no If "not ongoing": Duration of last attack: |__|__|__| (days)

Fever during last attack: ☐ yes ☐ no Lymphnode swelling during last attack: ☐ yes ☐ no

Peeling of limb after last attack: ☐ yes ☐ no Able to work during last attack: ☐ yes ☐ no

Occupation (specify:_________________________________)

Intensity of work: ☐ light ☐ heavy ☐ not working ☐ other (specify:____________________)

Number of attacks since last visit: |__|__|__| Duration of attacks (average since last visit): |__|__|__| (days)

HYGIENE STATUS

Hygiene assessment done: ☐ yes ☐ no

If "yes", date: |__|__| / |__|__| / |__|__|__|__| (dd/mm/yyyy)

LYMPHEDEMA MANAGEMENT TRAINING

Training has been given: ☐ yes ☐ no

If "yes", date: |__|__| / |__|__| / |__|__|__|__| (dd/mm/yyyy)

CIRCUMFERENCE MEASUREMENT OF LYMPHEDEMA -TAPE

Examiner: ______________________ Time of baseline measurement: |__|__|:|__|__| (h:min) ☐ AM ☐ PM

Date: |__|__| / |__|__| / |__|__|__|__| (dd/mm/yyyy) Time of measurement: |__|__|:|__|__| (h:min) ☐ AM ☐ PM

Measurements MUST be taken within 2 hours of the baseline time of measurement. If >2 hours before or after the baseline time, patient must return within the appropriate time window.

Main activity in the previous two hours before measurement: ☐ sitting ☐ walking ☐ standing ☐ farming ☐ fishing ☐ cooking ☐ driving ☐ child care ☐ other:_________________________

	**Circumferences of left leg**		**Circumferences of right leg**	
	1st measurement	2nd measurement	1st measurement	2nd measurement
A (10cm from tip of 1st toe)	|__|__|.|__| cm	|__|__|.|__| cm	|__|__|.|__| cm	|__|__|.|__| cm

**PodoPodoLEDoxy**	Indiv. No.: |__|__|-|__|__|__|-|__|__|__| Randomization Code: |__|__|__|
Date of visit: |__|__| / |__|__| / |__|__|__|__| (dd/mm/yyyy)	**VISIT 9**<br>**12 MONTHS FOLLOW-UP**

B (12cm)	|__|__|.|__| cm	|__|__|.|__| cm	|__|__|.|__| cm	|__|__|.|__| cm
C (20 cm)	|__|__|.|__| cm	|__|__|.|__| cm	|__|__|.|__| cm	|__|__|.|__| cm
D (30cm)	|__|__|.|__| cm	|__|__|.|__| cm	|__|__|.|__| cm	|__|__|.|__| cm

CIRCUMFERENCE MEASUREMENT OF LYMPHEDEMA –LYMPHATECH® SCANNER

Examiner: ________________________ Time of baseline measurement: |__|__|:|__|__| ☐ AM ☐ PM

(h:min)

Date: |__|__| / |__|__| / |__|__|__|__| Time of measurement**:** |__|__|:|__|__| ☐ AM ☐ PM

(dd/mm/yyyy) (h:min)

Measurements MUST be taken within 2 hours of the baseline time of measurement. If >2 hours before or after the baseline time, patient must return within the appropriate time window.

Main activity in the previous two hours before measurement: ☐ sitting ☐ walking ☐ standing ☐ farming ☐ fishing ☐ cooking ☐ driving ☐ child care ☐ other:___________________________

	**Circumferences of left leg**		**Circumferences of right leg**	
	1st measurement	2nd measurement	1st measurement	2nd measurement
A (12 cm)	|__|__|.|__| cm	|__|__|.|__| cm	|__|__|.|__| cm	|__|__|.|__| cm
B (20 cm)	|__|__|.|__| cm	|__|__|.|__| cm	|__|__|.|__| cm	|__|__|.|__| cm
C (30 cm)	|__|__|.|__| cm	|__|__|.|__| cm	|__|__|.|__| cm	|__|__|.|__| cm

VOLUME OF LYMPHEDEMA –LYMPHATECH® SCANNER

Examiner: ________________________ Time of baseline measurement: |__|__|:|__|__| ☐ AM ☐ PM

(h:min)

Date: |__|__| / |__|__| / |__|__|__|__| Time of measurement**:** |__|__|:|__|__| ☐ AM ☐ PM

(dd/mm/yyyy) (h:min)

Measurements MUST be taken within 2 hours of the baseline time of measurement. If >2 hours before or after the baseline time, patient must return within the appropriate time window.

Main activity in the previous two hours before measurement: ☐ sitting ☐ walking ☐ standing ☐ farming ☐ fishing ☐ cooking ☐ driving ☐ child care ☐ other:___________________________

	1st measurement	2nd measurement

**PodoPodoLEDoxy**	Indiv. No.: |__|__|-|__|__|__|-|__|__|__| Randomization Code: |__|__|__|

Date of visit: |__|__| / |__|__| / |__|__|__|__| (dd/mm/yyyy)	**VISIT 9**<br>**12 MONTHS FOLLOW-UP**

Volume of left leg	|__|__|__|__|__| ml	|__|__|__|__|__| ml
Volume of right leg	|__|__|__|__|__| ml	|__|__|__|__|__| ml
LymphaTech References/No.:____		Photograph

**PodoPodoLEDoxy**	Indiv. No.: |__|__|-|__|__|__|-|__|__|__| Randomization Code: |__|__|__|
Date of visit: |__|__| / |__|__| / |__|__|__|__| (dd/mm/yyyy)	**VISIT 9**<br>**12 MONTHS FOLLOW-UP**

ULTRASOUND ASSESSMENT OF SKIN THICKNESS AT BOTH ANKLES

Examiner: ________________________ Time of baseline measurement: |__|__|:|__|__| ☐ AM ☐ PM
(h:min)

Date: |__|__| / |__|__| / |__|__|__|__| Time of measurement: |__|__|:|__|__| ☐ AM ☐ PM
(dd/mm/yyyy) (h:min)

Measurements MUST be taken within 2 hours of the baseline time of measurement. If >2 hours before or after the baseline time, patient must return within the appropriate time window.

Main activity in the previous two hours before measurement: ☐ sitting ☐ walking ☐ standing ☐ farming ☐ fishing ☐ cooking ☐ driving ☐ child care ☐ other:__________________________

Presence of acute conditions:

Pregnancy: ☐ yes ☐ no

Febrile illness: ☐ yes ☐ no

Heart condition requiring diuretics: ☐ yes ☐ no

Adenolymphangitis attack: ☐ yes ☐ no if “yes”: ☐ left leg ☐ right leg

Recent leg trauma: ☐ yes ☐ no if “yes”: ☐ left leg ☐ right leg:

Other: ☐ yes ☐ no if “yes”, describe:______________________________

Has the patient been bed-bound more than one day within the past week? ☐ yes ☐ no

If “yes”, describe:____

Are open wounds present? ☐ yes ☐ no

If “yes”, ☐ left leg/acute ☐ left leg/chronic ☐ right leg/acute ☐ right leg/chronic

Even when open wounds are present, ultrasound should be done as long as it does not require placing the ultrasound probe within the field of the wound. If measurement cannot be done without placing the probe in the wound, skip that location and write “wound” in the notes section below.

Skin measurement

	1st measurement	2nd measurement	Notes
Left lateral malleolus	|__|.|__|__| cm	|__|.|__|__| cm	
Left medial malleolus	|__|.|__|__| cm	|__|.|__|__| cm	
Right lateral malleolus	|__|.|__|__| cm	|__|.|__|__| cm	
Right medial malleolus	|__|.|__|__| cm	|__|.|__|__| cm	

Ultrasound Photograph References/No.:____

**PodoPodoLEDoxy**	Indiv. No.: |__|__|-|__|__|__|-|__|__|__| Randomization Code: |__|__|__|
Date of visit: |__|__| / |__|__| / |__|__|__|__| (dd/mm/yyyy)	**VISIT 9**<br>**12 MONTHS FOLLOW-UP**

QUALITY OF LIFE QUESTIONNAIRE

QoL questionnaire carried out: ☐ yes ☐ no

If “yes”, date: |__|__| / |__|__| / |__|__|__|__|
(dd/mm/yyyy)

**PodoPodoLEDoxy**	Indiv. No.: |__|__|-|__|__|__|-|__|__|__| Randomization Code: |__|__|__|

Date of visit: |__|__| / |__|__| / |__|__|__|__| (dd/mm/yyyy)	**VISIT 9**<br>**12 MONTHS FOLLOW-UP**

LABORATORY ASSESSMENT (BLOOD)

Blood sample taken: ☐ yes ☐ no

If "no", state reason: ☐ refused ☐ absent ☐ other (specify: ________________________________)

If "yes": Sampling date: |__|__| / |__|__| / |__|__|__|__| Sampling time: |__|__| : |__|__| ☐ AM ☐ PM

(dd/mm/yyyy) (h:min)

Please attach corresponding barcode label(s) on the "Laboratory documentation sheet".
Please enter results on the „Laboratory assessment" form at the end of this CRF.

URINE SAMPLING

Urine sample taken: ☐ yes ☐ no

If "no", state reason: ☐ refused ☐ absent ☐ other (specify: ________________________________)

If "yes": Sampling date: |__|__| / |__|__| / |__|__|__|__| Sampling time: |__|__| : |__|__| ☐ AM ☐ PM

(dd/mm/yyyy) (h:min)

Please attach corresponding barcode label(s) on the "Laboratory documentation sheet".

SALIVA SAMPLING

Saliva sample taken: ☐ yes ☐ no

If "no", state reason: ☐ refused ☐ absent ☐ other (specify: ________________________________)

If "yes": Sampling date: |__|__| / |__|__| / |__|__|__|__| Sampling time: |__|__| : |__|__| ☐ AM ☐ PM

(dd/mm/yyyy) (h:min)

Please attach corresponding barcode label(s) on the "Laboratory documentation sheet".

PREGNANCY TEST

Sampling date: |__|__| / |__|__| / |__|__|__|__| Sampling time: |__|__| : |__|__| ☐ AM ☐ PM

(dd/mm/yyyy) (h:min)

Date analyzed: |__|__| / |__|__| / |__|__|__|__| Time analyzed: |__|__| : |__|__| ☐ AM ☐ PM

(dd/mm/yyyy) (h:min)

Result: ☐ neg. ☐ pos. ☐ not applicable (☐ ≥ 55 years, ☐ surgically sterilized)

REMARKS - VISIT 9 - 12 MONTHS FOLLOW-UP

VISIT 9 - 12 MONTHS FOLLOW-UP - INVESTIGATOR'S STATEMENT

I confirm that I have carefully examined all entries on the Visit 9 Case Report Form pages for this subject. All information entered by myself or my colleagues is, to the best of my knowledge, correct as of the date below.

Date: |__|__| / |__|__| / |__|__|__|__|

(dd/mm/yyyy)

____

(Investigator`s signature)

____

(Investigator`s name print)

**LEDoxy**	Indiv. No.: |__|__|-|__|__|__|-|__|__|__| Randomization Code: |__|__|__|

Date of visit: |__|__| / |__|__| / |__|__|__|__| (dd/mm/yyyy)	**VISIT 10**<br>**14 MONTHS FOLLOW-UP**

PRESENCE

Participant present for visit: ☐ yes ☐ no

If "no", state reason: ☐ refused ☐ traveled ☐ moved ☐ not traceable ☐ other

If "other", describe:____

CONCOMITANT MEDICATION

Change of medication since last visit? ☐ yes ☐ no

*If "**yes**", please enter on the "Concomitant Medication" form at the end of this CRF.*

ADLA QUESTIONNAIRE

ADLA attacks since last visit: ☐ yes ☐ no If "yes": Start date of last attack: |__|__| / |__|__|__|__| (mm/yyyy)

Ongoing?: ☐ yes ☐ no If "not ongoing": Duration of last attack: |__|__|__| (days)

Fever during last attack: ☐ yes ☐ no Lymphnode swelling during last attack: ☐ yes ☐ no

Peeling of limb after last attack: ☐ yes ☐ no Able to work during last attack: ☐ yes ☐ no

Occupation (specify:___________________________________)

Intensity of work: ☐ light ☐ heavy ☐ not working ☐ other (specify:______________________)

Number of attacks since last visit: |__|__|__| Duration of attacks (average since last visit): |__|__|__| (days)

REMARKS - VISIT 10 - 14 MONTHS FOLLOW-UP

VISIT 10 - 14 MONTHS FOLLOW-UP - INVESTIGATOR'S STATEMENT

I confirm that I have carefully examined all entries on the Visit 10 Case Report Form pages for this subject. All information entered by myself or my colleagues is, to the best of my knowledge, correct as of the date below.

Date: |__|__| / |__|__| / |__|__|__|__| (dd/mm/yyyy)

____
(Investigator`s signature)

____
(Investigator`s name print)

**PodoLEDoxy**	Indiv. No.: |__|__|-|__|__|__|-|__|__|__| Randomization Code: |__|__|__|
Date of visit: |__|__| / |__|__| / |__|__|__|__| (dd/mm/yyyy)	**VISIT 11**<br>**16 MONTHS FOLLOW-UP**

PRESENCE

Participant present for visit: ☐ yes ☐ no

If "no", state reason: ☐ refused ☐ traveled ☐ moved ☐ not traceable ☐ other

If "other", describe:____

CONCOMITANT MEDICATION

Change of medication since last visit? ☐ yes ☐ no

*If "**yes**", please enter on the "Concomitant Medication" form at the end of this CRF.*

ADLA QUESTIONNAIRE

ADLA attacks since last visit: ☐ yes ☐ no If "yes": Start date of last attack: |__|__| / |__|__|__|__| (mm/yyyy)

Ongoing?: ☐ yes ☐ no If "not ongoing": Duration of last attack: |__|__|__| (days)

Fever during last attack: ☐ yes ☐ no Lymphnode swelling during last attack: ☐ yes ☐ no

Peeling of limb after last attack: ☐ yes ☐ no Able to work during last attack: ☐ yes ☐ no

Occupation (specify:_____________________________________)

Intensity of work: ☐ light ☐ heavy ☐ not working ☐ other (specify:______________________)

Number of attacks since last visit: |__|__|__| Duration of attacks (average since last visit): |__|__|__| (days)

REMARKS - VISIT 11 - 16 MONTHS FOLLOW-UP

VISIT 11 - 16 MONTHS FOLLOW-UP - INVESTIGATOR'S STATEMENT

I confirm that I have carefully examined all entries on the Visit 11 Case Report Form pages for this subject. All information entered by myself or my colleagues is, to the best of my knowledge, correct as of the date below.

Date: |__|__| / |__|__| / |__|__|__|__| (dd/mm/yyyy)

____
(Investigator`s signature)

____
(Investigator`s name print)

PodoLEDoxy

Indiv. No.: |__|__|-|__|__|__|__|-|__|__|__|

Randomization Code: |__|__|__|

Date of visit: |__|__| / |__|__| / |__|__|__|__|
(dd/mm/yyyy)

VISIT 12
18 MONTHS FOLLOW-UP

PRESENCE

Participant present for visit: ☐ yes ☐ no

If "no", state reason: ☐ refused ☐ traveled ☐ moved ☐ not traceable ☐ other

If "other", describe:____

CONCOMITANT MEDICATION

Change of medication since last visit? ☐ yes ☐ no

*If "**yes**", please enter on the "Concomitant Medication" form at the end of this CRF.*

ADLA QUESTIONNAIRE

ADLA attacks since last visit: ☐ yes ☐ no If "yes": Start date of last attack: |__|__| / |__|__|__|__|
(mm/yyyy)

Ongoing?: ☐ yes ☐ no If "not ongoing": Duration of last attack: |__|__|__|
(days)

Fever during last attack: ☐ yes ☐ no Lymphnode swelling during last attack: ☐ yes ☐ no

Peeling of limb after last attack: ☐ yes ☐ no Able to work during last attack: ☐ yes ☐ no

Occupation (specify:________________________________)

Intensity of work: ☐ light ☐ heavy ☐ not working ☐ other (specify:____________________)

Number of attacks since last visit: |__|__|__| Duration of attacks (average since last visit): |__|__|__|
(days)

HYGIENE STATUS

Hygiene assessment done: ☐ yes ☐ no If "yes", date: |__|__| / |__|__| / |__|__|__|__|
(dd/mm/yyyy)

LYMPHEDEMA MANAGEMENT TRAINING

Training has been given: ☐ yes ☐ no If "yes", date: |__|__| / |__|__| / |__|__|__|__|
(dd/mm/yyyy)

REMARKS - VISIT 12 - 18 MONTHS FOLLOW-UP

VISIT 12 - 18 MONTHS FOLLOW-UP - INVESTIGATOR'S STATEMENT

I confirm that I have carefully examined all entries on the Visit 12 Case Report Form pages for this subject. All information entered by myself or my colleagues is, to the best of my knowledge, correct as of the date below.

Date: |__|__| / |__|__| / |__|__|__|__|

(dd/mm/yyyy)

____
(Investigator`s signature)

____
(Investigator`s name print)

**PodoLEDoxy**	Indiv. No.: |__|__|-|__|__|__|-|__|__|__| Randomization Code: |__|__|__|
Date of visit: |__|__| / |__|__| / |__|__|__|__| (dd/mm/yyyy)	**VISIT 13**<br>**20 MONTHS FOLLOW-UP**

PRESENCE

Participant present for visit: ☐ yes ☐ no

If "no", state reason: ☐ refused ☐ traveled ☐ moved ☐ not traceable ☐ other

If "other", describe:____

CONCOMITANT MEDICATION

Change of medication since last visit? ☐ yes ☐ no

*If "**yes**", please enter on the "Concomitant Medication" form at the end of this CRF.*

ADLA QUESTIONNAIRE

ADLA attacks since last visit: ☐ yes ☐ no If "yes": Start date of last attack: |__|__| / |__|__|__|__| (mm/yyyy)

Ongoing?: ☐ yes ☐ no If "not ongoing": Duration of last attack: |__|__|__| (days)

Fever during last attack: ☐ yes ☐ no Lymphnode swelling during last attack: ☐ yes ☐ no

Peeling of limb after last attack: ☐ yes ☐ no Able to work during last attack: ☐ yes ☐ no

Occupation (specify:_____________________________________)

Intensity of work: ☐ light ☐ heavy ☐ not working ☐ other (specify:______________________)

Number of attacks since last visit: |__|__|__| Duration of attacks (average since last visit): |__|__|__| (days)

REMARKS - VISIT 13 - 20 MONTHS FOLLOW-UP

VISIT 13 - 20 MONTHS FOLLOW-UP - INVESTIGATOR'S STATEMENT

I confirm that I have carefully examined all entries on the Visit 13 Case Report Form pages for this subject. All information entered by myself or my colleagues is, to the best of my knowledge, correct as of the date below.

Date: |__|__| / |__|__| / |__|__|__|__| (dd/mm/yyyy)

____
(Investigator`s signature)

____
(Investigator`s name print)

**PodoLEDoxy**	Indiv. No.: |__|__|-|__|__|__|-|__|__|__| Randomization Code: |__|__|__|
Date of visit: |__|__| / |__|__| / |__|__|__|__| (dd/mm/yyyy)	**VISIT 14**<br>**22 MONTHS FOLLOW-UP**

PRESENCE

Participant present for visit: ☐ yes ☐ no

If "no", state reason: ☐ refused ☐ traveled ☐ moved ☐ not traceable ☐ other

If "other", describe:____

CONCOMITANT MEDICATION

Change of medication since last visit? ☐ yes ☐ no

*If "**yes**", please enter on the "Concomitant Medication" form at the end of this CRF.*

ADLA QUESTIONNAIRE

ADLA attacks since last visit: ☐ yes ☐ no If "yes": Start date of last attack: |__|__| / |__|__|__|__| (mm/yyyy)

Ongoing?: ☐ yes ☐ no If "not ongoing": Duration of last attack: |__|__|__| (days)

Fever during last attack: ☐ yes ☐ no Lymphnode swelling during last attack: ☐ yes ☐ no

Peeling of limb after last attack: ☐ yes ☐ no Able to work during last attack: ☐ yes ☐ no

Occupation (specify:_________________________)

Intensity of work: ☐ light ☐ heavy ☐ not working ☐ other (specify:_______________)

Number of attacks since last visit: |__|__|__| Duration of attacks (average since last visit): |__|__|__| (days)

REMARKS - VISIT 14 - 22 MONTHS FOLLOW-UP

VISIT 14 - 22 MONTHS FOLLOW-UP - INVESTIGATOR'S STATEMENT

I confirm that I have carefully examined all entries on the Visit 14 Case Report Form pages for this subject. All information entered by myself or my colleagues is, to the best of my knowledge, correct as of the date below.

Date: |__|__| / |__|__| / |__|__|__|__| (dd/mm/yyyy)

____
(Investigator`s signature)

____
(Investigator`s name print)

PodoLEDoxy

Indiv. No.: |__|__|-|__|__|__|-|__|__|__|
Randomization Code: |__|__|__|

Date of visit: |__|__| / |__|__| / |__|__|__|__|
(dd/mm/yyyy)

VISIT 15
24 MONTHS FOLLOW-UP

PRESENCE

Participant present for visit: ☐ yes ☐ no

If "no", state reason: ☐ refused ☐ traveled ☐ moved ☐ not traceable ☐ other

If "other", describe:____

CONCOMITANT MEDICATION

Change of medication since last visit? ☐ yes ☐ no

*If "**yes**", please enter on the „Concomitant Medication" form at the end of this CRF.*

VITAL SIGNS

Blood pressure: |__|__|__| / |__|__|__| mmHg Hypertension: ☐ yes ☐ no
systolic diastolic

If "hypertensive": Known hypertension: ☐ yes ☐ no Hypertensive medication: ☐ yes ☐ no

*If "**yes**", enter on the "Concomitant Medication" form at the end of this CRF*

Pulse rate: |__|__|__| bpm Temperature: |__|__|.|__|°C Weight: |__|__|__|.|__| kg

CLINICAL PHOTOGRAPHS

Clinical photographs taken: ☐ yes ☐ no

If "yes", Photograph References/No.:____

LYMPHEDEMA STAGING

Stage	Description	Right Leg	Left Leg
0	No abnormality	☐	☐
1	Swelling is reversible (goes away) overnight	☐	☐
2	Swelling is not reversible (doesn't go away)	☐	☐
3	Presence of shallow skin folds<br>(base of fold can be seen by straightening the ankle)	☐	☐
4	Presence of skin knobs	☐	☐
5	Presence of deep skin folds (base of fold can only be seen if edges are actively separated by hand)	☐	☐
6	Presence of "mossy lesions"<br>Warty looking epidermal skin lesions.	☐	☐
7	Unable to care for self or perform daily activities	☐	☐

**PodoLEDoxy**	Indiv. No.: |__|__|-|__|__|__|-|__|__|__| Randomization Code: |__|__|__|
Date of visit: |__|__| / |__|__| / |__|__|__|__| (dd/mm/yyyy)	**VISIT 15**<br>**24 MONTHS FOLLOW-UP**

ADLA QUESTIONNAIRE

ADLA attacks since last visit: ☐ yes ☐ no If "yes": Start date of last attack: |__|__| / |__|__|__|__| (mm/yyyy)

Ongoing?: ☐ yes ☐ no If "not ongoing": Duration of last attack: |__|__|__| (days)

Fever during last attack: ☐ yes ☐ no Lymphnode swelling during last attack: ☐ yes ☐ no

Peeling of limb after last attack: ☐ yes ☐ no Able to work during last attack: ☐ yes ☐ no

Occupation (specify:_________________________________)

Intensity of work: ☐ light ☐ heavy ☐ not working ☐ other (specify:____________________)

Number of attacks since last visit: |__|__|__| Duration of attacks (average since last visit): |__|__|__| (days)

HYGIENE STATUS

Hygiene assessment done: ☐ yes ☐ no

If "yes", date: |__|__| / |__|__| / |__|__|__|__| (dd/mm/yyyy)

LYMPHEDEMA MANAGEMENT TRAINING

Training has been given: ☐ yes ☐ no

If "yes", date: |__|__| / |__|__| / |__|__|__|__| (dd/mm/yyyy)

CIRCUMFERENCE MEASUREMENT OF LYMPHEDEMA -TAPE

Examiner: ______________________ Time of baseline measurement: |__|__|:|__|__| ☐ AM ☐ PM (h:min)

Date: |__|__| / |__|__| / |__|__|__|__| (dd/mm/yyyy) Time of measurement: |__|__|:|__|__| ☐ AM ☐ PM (h:min)

Measurements MUST be taken within 2 hours of the baseline time of measurement. If >2 hours before or after the baseline time, patient must return within the appropriate time window.

Main activity in the previous two hours before measurement: ☐ sitting ☐ walking ☐ standing ☐ farming ☐ fishing ☐ cooking ☐ driving ☐ child care ☐ other:_________________________

	**Circumferences of left leg**		**Circumferences of right leg**	
	1st measurement	2nd measurement	1st measurement	2nd measurement
A (10cm from tip of 1st toe)	|__|__|.|__| cm	|__|__|.|__| cm	|__|__|.|__| cm	|__|__|.|__| cm

**PodoLEDoxy**	Indiv. No.: |__|__|-|__|__|__|-|__|__|__| Randomization Code: |__|__|__|
Date of visit: |__|__| / |__|__| / |__|__|__|__| (dd/mm/yyyy)	**VISIT 15**<br>**24 MONTHS FOLLOW-UP**

B (12cm)	|__|__|.|__| cm	|__|__|.|__| cm	|__|__|.|__| cm	|__|__|.|__| cm
C (20 cm)	|__|__|.|__| cm	|__|__|.|__| cm	|__|__|.|__| cm	|__|__|.|__| cm
D (30cm)	|__|__|.|__| cm	|__|__|.|__| cm	|__|__|.|__| cm	|__|__|.|__| cm

CIRCUMFERENCE MEASUREMENT OF LYMPHEDEMA –LYMPHATECH® SCANNER

Examiner: ________________________ Time of baseline measurement: |__|__|:|__|__| ☐ AM ☐ PM
(h:min)

Date: |__|__| / |__|__| / |__|__|__|__| Time of measurement**:** |__|__|:|__|__| ☐ AM ☐ PM
(dd/mm/yyyy) (h:min)

Measurements MUST be taken within 2 hours of the baseline time of measurement. If >2 hours before or after the baseline time, patient must return within the appropriate time window.

Main activity in the previous two hours before measurement: ☐ sitting ☐ walking ☐ standing ☐ farming ☐ fishing ☐ cooking ☐ driving ☐ child care ☐ other:___________________________

	**Circumferences of left leg**		**Circumferences of right leg**	
	1st measurement	2nd measurement	1st measurement	2nd measurement
A (12 cm)	|__|__|.|__| cm	|__|__|.|__| cm	|__|__|.|__| cm	|__|__|.|__| cm
B (20 cm)	|__|__|.|__| cm	|__|__|.|__| cm	|__|__|.|__| cm	|__|__|.|__| cm
C (30 cm)	|__|__|.|__| cm	|__|__|.|__| cm	|__|__|.|__| cm	|__|__|.|__| cm

VOLUME OF LYMPHEDEMA –LYMPHATECH® SCANNER

Examiner: ________________________ Time of baseline measurement: |__|__|:|__|__| ☐ AM ☐ PM
(h:min)

Date: |__|__| / |__|__| / |__|__|__|__| Time of measurement**:** |__|__|:|__|__| ☐ AM ☐ PM
(dd/mm/yyyy) (h:min)

Measurements MUST be taken within 2 hours of the baseline time of measurement. If >2 hours before or after the baseline time, patient must return within the appropriate time window.

Main activity in the previous two hours before measurement: ☐ sitting ☐ walking ☐ standing ☐ farming ☐ fishing ☐ cooking ☐ driving ☐ child care ☐ other:___________________________

	1st measurement	2nd measurement

**PodoLEDoxy**	Indiv. No.: |__|__|-|__|__|__|-|__|__|__| Randomization Code: |__|__|__|

Date of visit: |__|__| / |__|__| / |__|__|__|__| (dd/mm/yyyy)	**VISIT 15**<br>**24 MONTHS FOLLOW-UP**

Volume of left leg	|__|__|__|__|__| ml	|__|__|__|__|__| ml
Volume of right leg	|__|__|__|__|__| ml	|__|__|__|__|__| ml
LymphaTech References/No.:____		Photograph

**PodoLEDoxy**	Indiv. No.: |__|__|-|__|__|__|-|__|__|__| Randomization Code: |__|__|__|
Date of visit: |__|__| / |__|__| / |__|__|__|__| (dd/mm/yyyy)	**VISIT 15**<br>**24 MONTHS FOLLOW-UP**

ULTRASOUND ASSESSMENT OF SKIN THICKNESS AT BOTH ANKLES

Examiner: ________________________ Time of baseline measurement: |__|__|:|__|__| ☐ AM ☐ PM

(h:min)

Date: |__|__| / |__|__| / |__|__|__|__| Time of measurement: |__|__|:|__|__| ☐ AM ☐ PM

(dd/mm/yyyy) (h:min)

Measurements MUST be taken within 2 hours of the baseline time of measurement. If >2 hours before or after the baseline time, patient must return within the appropriate time window.

Main activity in the previous two hours before measurement: ☐ sitting ☐ walking ☐ standing ☐ farming ☐ fishing ☐ cooking ☐ driving ☐ child care ☐ other:__________________________

Presence of acute conditions:

Pregnancy: ☐ yes ☐ no

Febrile illness: ☐ yes ☐ no

Heart condition requiring diuretics: ☐ yes ☐ no

Adenolymphangitis attack: ☐ yes ☐ no if "yes": ☐ left leg ☐ right leg

Recent leg trauma: ☐ yes ☐ no if "yes": ☐ left leg ☐ right leg:

Other: ☐ yes ☐ no if "yes", describe:______________________________

Has the patient been bed-bound more than one day within the past week? ☐ yes ☐ no

If "yes", describe:____

Are open wounds present? ☐ yes ☐ no

If "yes", ☐ left leg/acute ☐ left leg/chronic ☐ right leg/acute ☐ right leg/chronic

Even when open wounds are present, ultrasound should be done as long as it does not require placing the ultrasound probe within the field of the wound. If measurement cannot be done without placing the probe in the wound, skip that location and write "wound" in the notes section below.

Skin measurement

	1st measurement	2nd measurement	Notes
Left lateral malleolus	|__|.|__|__| cm	|__|.|__|__| cm	
Left medial malleolus	|__|.|__|__| cm	|__|.|__|__| cm	
Right lateral malleolus	|__|.|__|__| cm	|__|.|__|__| cm	
Right medial malleolus	|__|.|__|__| cm	|__|.|__|__| cm	

Ultrasound Photograph References/No.:____

**PodoLEDoxy**	Indiv. No.: |__|__|-|__|__|__|-|__|__|__| Randomization Code: |__|__|__|
Date of visit: |__|__| / |__|__| / |__|__|__|__| (dd/mm/yyyy)	**VISIT 15**<br>**24 MONTHS FOLLOW-UP**

QUALITY OF LIFE QUESTIONNAIRE

QoL questionnaire carried out: ☐ yes ☐ no

If "yes", date: |__|__| / |__|__| / |__|__|__|__|
(dd/mm/yyyy)

LABORATORY ASSESSMENT (BLOOD)

Blood sample taken: ☐ yes ☐ no

If "no", state reason: ☐ refused ☐ absent ☐ other (specify: ________________________________)

If "yes": Sampling date: |__|__| / |__|__| / |__|__|__|__| Sampling time: |__|__| : |__|__| ☐ AM ☐ PM
(dd/mm/yyyy) (h:min)

Please attach corresponding barcode label(s) on the "Laboratory documentation sheet".
Please enter results on the „Laboratory assessment" form at the end of this CRF.

URINE SAMPLING

Urine sample taken: ☐ yes ☐ no

If "no", state reason: ☐ refused ☐ absent ☐ other (specify: ________________________________)

If "yes": Sampling date: |__|__| / |__|__| / |__|__|__|__| Sampling time: |__|__| : |__|__| ☐ AM ☐ PM
(dd/mm/yyyy) (h:min)

Please attach corresponding barcode label(s) on the "Laboratory documentation sheet".

SALIVA SAMPLING

Saliva sample taken: ☐ yes ☐ no

If "no", state reason: ☐ refused ☐ absent ☐ other (specify: ________________________________)

If "yes": Sampling date: |__|__| / |__|__| / |__|__|__|__| Sampling time: |__|__| : |__|__| ☐ AM ☐ PM
(dd/mm/yyyy) (h:min)

Please attach corresponding barcode label(s) on the "Laboratory documentation sheet".

PREGNANCY TEST

Sampling date: |__|__| / |__|__| / |__|__|__|__| Sampling time: |__|__| : |__|__| ☐ AM ☐ PM
(dd/mm/yyyy) (h:min)

Date analyzed: |__|__| / |__|__| / |__|__|__|__| Time analyzed: |__|__| : |__|__| ☐ AM ☐ PM
(dd/mm/yyyy) (h:min)

Result: ☐ neg. ☐ pos. ☐ not applicable (☐ ≥ 55 years, ☐ surgically sterilized)

**PodoLEDoxy**	Indiv. No.: |__|__|-|__|__|__|-|__|__|__| Randomization Code: |__|__|__|
Date of visit: |__|__| / |__|__| / |__|__|__|__| (dd/mm/yyyy)	**VISIT 15**<br>**24 MONTHS FOLLOW-UP**

REMARKS - VISIT 15 - 24 MONTHS FOLLOW-UP

VISIT 15 - 24 MONTHS FOLLOW-UP - INVESTIGATOR’S STATEMENT

I confirm that I have carefully examined all entries on the Visit 15 Case Report Form pages for this subject. All information entered by myself or my colleagues is, to the best of my knowledge, correct as of the date below.

Date: |__|__| / |__|__| / |__|__|__|__|

(dd/mm/yyyy)

____

(Investigator`s signature)

____

(Investigator`s name print)

**PodoLEDoxy**	Indiv. No.: |__|__|-|__|__|__|-|__|__|__| Randomization Code: |__|__|__|
Date of visit: |__|__| / |__|__| / |__|__|__|__| (dd/mm/yyyy)	**END OF STUDY**

Date of subject completion or discontinuation from the study: |__|__| / |__|__| / |__|__|__|__|
(dd/mm/yyyy)

Did the subject discontinue the study prematurely? ☐ yes ☐ no

If "yes", tick the primary reason for discontinuation:

Consent withdrawn	☐	
Adverse event	☐	
Lost during treatment	☐	
Lost to follow up	☐	
Protocol violation	☐	Specify:______________________________
Other	☐	Specify:______________________________

END OF STUDY - INVESTIGATOR'S STATEMENT

I confirm that I have carefully examined all entries on the previous Case Report Form pages for this subject. All information entered by myself or my colleagues is, to the best of my knowledge, correct as of the date below.

Date: |__|__| / |__|__| / |__|__|__|__|
(dd/mm/yyyy)

____
(Investigator`s signature)

____
(Investigator`s name print)

**PodoLEDoxy**	Indiv. No.: |__|__|-|__|__|__|- |__|__|__| Randomization Code: |__|__|__|	**LABORATORY ASSESSMENT**

LABORATORY ASSESSMENT (BLOOD) – SCREENING – VISIT 1

Sampling date: |__|__| / |__|__| / |__|__|__|__| (dd/mm/yyyy) Sampling time: |__|__| : |__|__| ☐ AM ☐ PM (h:min)

FULL BLOODCELL COUNT

Date analyzed: |__|__| / |__|__| / |__|__|__|__| (dd/mm/yyyy)

Haemoglobin: |__|__|__|__|.|__|__| g/dl

Neutrophils: |__|__|__|__|.|__|__| x 10^9/L

Platelets: |__|__|__|__|.|__|__| x 10^9/L

BIOCHEMISTRY

Creatinine: |__|__|__|__|.|__|__| ☐ µmol/l ☐ mg/dl Date analyzed: |__|__| / |__|__| / |__|__|__|__| (dd/mm/yyyy)

AST (GOT): |__|__|__|__|.|__|__| U/L Date analyzed: |__|__| / |__|__| / |__|__|__|__| (dd/mm/yyyy)

ALT (GPT): |__|__|__|__|.|__|__| U/L Date analyzed: |__|__| / |__|__| / |__|__|__|__| (dd/mm/yyyy)

γ-GT: |__|__|__|__|.|__|__| U/L Date analyzed: |__|__| / |__|__| / |__|__|__|__| (dd/mm/yyyy)

FILARIAL TEST STRIP (FTS)

Date analyzed: |__|__| / |__|__| / |__|__|__|__| (dd/mm/yyyy) Time analyzed: |__|__| : |__|__| ☐ AM ☐ PM (h:min)

FTS Result: : ☐ neg. ☐ pos

MICROFILARIAE COUNT - SEDGEWICK *(only to be done if FTS positive!)*

Date analyzed: |__|__| / |__|__| / |__|__|__|__| (dd/mm/yyyy) Time analyzed: |__|__| : |__|__| ☐ AM ☐ PM (h:min)

Sedgewick: |__|__|__|__|__| mf/ml

Attach corresponding barcode label(s) on the "Laboratory documentation sheet".

LABORATORY ASSESSMENT (BLOOD) – BEFORE TREATMENT NO.22

Sampling date: |__|__| / |__|__| / |__|__|__|__| (dd/mm/yyyy) Sampling time: |__|__| : |__|__| ☐ AM ☐ PM (h:min)

BIOCHEMISTRY

AST (GOT): |__|__|__|__|.|__|__| U/L Date analyzed: |__|__| / |__|__| / |__|__|__|__| (dd/mm/yyyy)

ALT (GPT): |__|__|__|__|.|__|__| U/L Date analyzed: |__|__| / |__|__| / |__|__|__|__| (dd/mm/yyyy)

y-GT: |__|__|__|__|.|__|__| U/L Date analyzed: |__|__| / |__|__| / |__|__|__|__| (dd/mm/yyyy)

Attach corresponding barcode label(s) on the "Laboratory documentation sheet".

LABORATORY ASSESSMENT (BLOOD) – AFTER TREATMENT NO.42

Sampling date: |__|__| / |__|__| / |__|__|__|__| (dd/mm/yyyy) Sampling time: |__|__| : |__|__| ☐ AM ☐ PM (h:min)

BIOCHEMISTRY

AST (GOT): |__|__|__|__|__|.|__|__| U/L Date analyzed: |__|__| / |__|__| / |__|__|__|__| (dd/mm/yyyy)

ALT (GPT): |__|__|__|__|__|.|__|__| U/L Date analyzed: |__|__| / |__|__| / |__|__|__|__| (dd/mm/yyyy)

y-GT: |__|__|__|__|__|.|__|__| U/L Date analyzed: |__|__| / |__|__| / |__|__|__|__| (dd/mm/yyyy)

Attach corresponding barcode label(s) on the "Laboratory documentation sheet".

LABORATORY ASSESSMENT (BLOOD) – VISIT 6 – 6 MONTHS FOLLOW UP

Sampling date: |__|__| / |__|__| / |__|__|__|__| (dd/mm/yyyy) Sampling time: |__|__| : |__|__| ☐ AM ☐ PM (h:min)

FILARIAL TEST STRIP (FTS)

Date analyzed: |__|__| / |__|__| / |__|__|__|__| (dd/mm/yyyy) Time analyzed: |__|__| : |__|__| ☐ AM ☐ PM (h:min)

FTS Result: : ☐ neg. ☐ pos

MICROFILARIAE COUNT - SEDGEWICK ***(only to be done if FTS positive!)***

Date analyzed: |__|__| / |__|__| / |__|__|__|__| (dd/mm/yyyy) Time analyzed: |__|__| : |__|__| ☐ AM ☐ PM (h:min)

Sedgewick: |__|__|__|__|__|__| mf/ml

Attach corresponding barcode label(s) on the "Laboratory documentation sheet".

LABORATORY ASSESSMENT (BLOOD) – VISIT 9 – 12 MONTHS FOLLOW UP

Sampling date: |__|__| / |__|__| / |__|__|__|__| (dd/mm/yyyy) Sampling time: |__|__| : |__|__| ☐ AM ☐ PM (h:min)

FILARIAL TEST STRIP (FTS)

Date analyzed: |__|__| / |__|__| / |__|__|__|__| (dd/mm/yyyy) Time analyzed: |__|__| : |__|__| ☐ AM ☐ PM (h:min)

FTS Result: : ☐ neg. ☐ pos

MICROFILARIAE COUNT - SEDGEWICK ***(only to be done if FTS positive!)***

Date analyzed: |__|__| / |__|__| / |__|__|__|__| (dd/mm/yyyy) Time analyzed: |__|__| : |__|__| ☐ AM ☐ PM (h:min)

Sedgewick: |__|__|__|__|__|__| mf/ml

Attach corresponding barcode label(s) on the "Laboratory documentation sheet".

LABORATORY ASSESSMENT (BLOOD) – VISIT 15 – 24 MONTHS FOLLOW UP

Sampling date: |__|__| / |__|__| / |__|__|__|__| (dd/mm/yyyy) Sampling time: |__|__| : |__|__| ☐ AM ☐ PM (h:min)

FILARIAL TEST STRIP (FTS)

Date analyzed: |__|__| / |__|__| / |__|__|__|__| (dd/mm/yyyy) Time analyzed: |__|__| : |__|__| ☐ AM ☐ PM (h:min)

FTS Result: : ☐ neg. ☐ pos

MICROFILARIAE COUNT - SEDGEWICK ***(only to be done if FTS positive!)***

Date analyzed: |__|__| / |__|__| / |__|__|__|__| (dd/mm/yyyy) Time analyzed: |__|__| : |__|__| ☐ AM ☐ PM (h:min)

Sedgewick: |__|__|__|__|__|__| mf/ml

Attach corresponding barcode label(s) on the "Laboratory documentation sheet".

**PodoLEDoxy**	Indiv. No.: |__|__|-|__|__|__|__|-|__|__|__| Randomization Code: |__|__|__|	**CONCOMITANT MEDICATION** Page |__|

CONCOMITANT MEDICATION

Medication name (Trade or generic name)	Dose			Application form**	Route of administration***	Indication	Duration		ongoing
	Dose	Unit	Freq*				Start (dd/mm/yyyy)	Stop (dd/mm/yyyy)	
1.			|__|	|__|	|__|		|__|__|/|__|__|/|__|__|__|__|	|__|__|/|__|__|/|__|__|__|__|	☐
2.			|__|	|__|	|__|		|__|__|/|__|__|/|__|__|__|__|	|__|__|/|__|__|/|__|__|__|__|	☐
3.			|__|	|__|	|__|		|__|__|/|__|__|/|__|__|__|__|	|__|__|/|__|__|/|__|__|__|__|	☐
4.			|__|	|__|	|__|		|__|__|/|__|__|/|__|__|__|__|	|__|__|/|__|__|/|__|__|__|__|	☐
5.			|__|	|__|	|__|		|__|__|/|__|__|/|__|__|__|__|	|__|__|/|__|__|/|__|__|__|__|	☐
6.			|__|	|__|	|__|		|__|__|/|__|__|/|__|__|__|__|	|__|__|/|__|__|/|__|__|__|__|	☐
7.			|__|	|__|	|__|		|__|__|/|__|__|/|__|__|__|__|	|__|__|/|__|__|/|__|__|__|__|	☐
8.			|__|	|__|	|__|		|__|__|/|__|__|/|__|__|__|__|	|__|__|/|__|__|/|__|__|__|__|	☐
9.			|__|	|__|	|__|		|__|__|/|__|__|/|__|__|__|__|	|__|__|/|__|__|/|__|__|__|__|	☐
10.			|__|	|__|	|__|		|__|__|/|__|__|/|__|__|__|__|	|__|__|/|__|__|/|__|__|__|__|	☐

* 1 = 1x /day, 2 = 2x /day, 3 = 3x /day, 4 = 4x /day, 5 = 5x /day, 6 = weekly, 7 = when needed

** 1 = Tablet, 2 = Cream, 3 = Spray, 4 = Syringe, 5 = Suppository, 6 = Drop, 7 = Infusion , 8 = herbal elixir
*** 1 = p.o., 2 = i.v., 3 = i.m., 4 = s.c., 5 = rectal, 6 = dermal, 7 = p.inh.

CONCOMITANT MEDICATION

Medication name (Trade or generic name)	Dose			Application form**	Route of administration***	Indication	Duration		ongoing
	Dose	Unit	Freq*				Start (dd/mm/yyyy)	Stop (dd/mm/yyyy)	
11.			|__|	|__|	|__|		|__|__|/|__|__|/|__|__|__|__|	|__|__|/|__|__|/|__|__|__|__|	☐
12.			|__|	|__|	|__|		|__|__|/|__|__|/|__|__|__|__|	|__|__|/|__|__|/|__|__|__|__|	☐
13.			|__|	|__|	|__|		|__|__|/|__|__|/|__|__|__|__|	|__|__|/|__|__|/|__|__|__|__|	☐
14.			|__|	|__|	|__|		|__|__|/|__|__|/|__|__|__|__|	|__|__|/|__|__|/|__|__|__|__|	☐
15.			|__|	|__|	|__|		|__|__|/|__|__|/|__|__|__|__|	|__|__|/|__|__|/|__|__|__|__|	☐
16.			|__|	|__|	|__|		|__|__|/|__|__|/|__|__|__|__|	|__|__|/|__|__|/|__|__|__|__|	☐
17.			|__|	|__|	|__|		|__|__|/|__|__|/|__|__|__|__|	|__|__|/|__|__|/|__|__|__|__|	☐
18.			|__|	|__|	|__|		|__|__|/|__|__|/|__|__|__|__|	|__|__|/|__|__|/|__|__|__|__|	☐
19.			|__|	|__|	|__|		|__|__|/|__|__|/|__|__|__|__|	|__|__|/|__|__|/|__|__|__|__|	☐
20.			|__|	|__|	|__|		|__|__|/|__|__|/|__|__|__|__|	|__|__|/|__|__|/|__|__|__|__|	☐

* 1 = 1x /day, 2 = 2x /day, 3 = 3x /day, 4 = 4x /day, 5 = 5x /day, 6 = weekly, 7 = when needed

** 1 = Tablet, 2 = Cream, 3 = Spray, 4 = Syringe, 5 = Suppository, 6 = Drop, 7 = Infusion, 8 = herbal elixir

*** 1 = p.o., 2 = i.v., 3 = i.m., 4 = s.c., 5 = rectal, 6 = dermal, 7 = p.inh.

**PodoLEDoxy**	Indiv. No.: |__|__|-|__|__|__|__|-|__|__|__| Randomization Code: |__|__|__|	**CONCOMITANT MEDICATION** Page |__|

CONCOMITANT MEDICATION

**Medication name (Trade or generic name)**	**Dose**			**Application form****	**Route of administration*****	**Indication**	**Duration**		**ongoing**
	**Dose**	**Unit**	**Freq***				**Start (dd/mm/yyyy)**	**Stop (dd/mm/yyyy)**	
21.			|__|	|__|	|__|		|__|__|/|__|__|/|__|__|__|__|	|__|__|/|__|__|/|__|__|__|__|	☐
22.			|__|	|__|	|__|		|__|__|/|__|__|/|__|__|__|__|	|__|__|/|__|__|/|__|__|__|__|	☐
23.			|__|	|__|	|__|		|__|__|/|__|__|/|__|__|__|__|	|__|__|/|__|__|/|__|__|__|__|	☐
24.			|__|	|__|	|__|		|__|__|/|__|__|/|__|__|__|__|	|__|__|/|__|__|/|__|__|__|__|	☐
25.			|__|	|__|	|__|		|__|__|/|__|__|/|__|__|__|__|	|__|__|/|__|__|/|__|__|__|__|	☐
26.			|__|	|__|	|__|		|__|__|/|__|__|/|__|__|__|__|	|__|__|/|__|__|/|__|__|__|__|	☐
27.			|__|	|__|	|__|		|__|__|/|__|__|/|__|__|__|__|	|__|__|/|__|__|/|__|__|__|__|	☐
28.			|__|	|__|	|__|		|__|__|/|__|__|/|__|__|__|__|	|__|__|/|__|__|/|__|__|__|__|	☐
29.			|__|	|__|	|__|		|__|__|/|__|__|/|__|__|__|__|	|__|__|/|__|__|/|__|__|__|__|	☐
30.			|__|	|__|	|__|		|__|__|/|__|__|/|__|__|__|__|	|__|__|/|__|__|/|__|__|__|__|	☐

* 1 = 1x /day, 2 = 2x /day, 3 = 3x /day, 4 = 4x /day, 5 = 5x /day, 6 = weekly, 7 = when needed

** 1 = Tablet, 2 = Cream, 3 = Spray, 4 = Syringe, 5 = Suppository, 6 = Drop, 7 = Infusion , 8 = herbal elixir
*** 1 = p.o., 2 = i.v., 3 = i.m., 4 = s.c., 5 = rectal, 6 = dermal, 7 = p.inh.

ADVERSE EVENTS (AE/SAE)

No.	Description	Duration: Start date (dd/mm/yyyy)	Duration: End date (dd/mm/yyyy)	ongoing	Grade 1 = mild 2 = moderate 3 = severe	SAE 0 = no 1 = yes	SAE-Criteria*	AE related to treatment**	Outcome of AE***	Therapy of AE 0 = no 1 = yes (if yes, please specify and enter on "concomitant medication" sheet)	Change of study medication****
|__|		|__|__|/|__|__|/|__|__|__|__|	|__|__|/|__|__|/|__|__|__|__|	☐	|__|	|__|	|__|	|__|	|__|	|__|	|__|
|__|		|__|__|/|__|__|/|__|__|__|__|	|__|__|/|__|__|/|__|__|__|__|	☐	|__|	|__|	|__|	|__|	|__|	|__|	|__|
|__|		|__|__|/|__|__|/|__|__|__|__|	|__|__|/|__|__|/|__|__|__|__|	☐	|__|	|__|	|__|	|__|	|__|	|__|	|__|
|__|		|__|__|/|__|__|/|__|__|__|__|	|__|__|/|__|__|/|__|__|__|__|	☐	|__|	|__|	|__|	|__|	|__|	|__|	|__|
|__|		|__|__|/|__|__|/|__|__|__|__|	|__|__|/|__|__|/|__|__|__|__|	☐	|__|	|__|	|__|	|__|	|__|	|__|	|__|
|__|		|__|__|/|__|__|/|__|__|__|__|	|__|__|/|__|__|/|__|__|__|__|	☐	|__|	|__|	|__|	|__|	|__|	|__|	|__|
|__|		|__|__|/|__|__|/|__|__|__|__|	|__|__|/|__|__|/|__|__|__|__|	☐	|__|	|__|	|__|	|__|	|__|	|__|	|__|

* SAE criteria: 1 = Death, 2 = Life-threatening, 3 = Hospitalization or prolongation of existing hospitalization, 4 = Persistent or significant disability/incapacity, 5 = Congenital anomaly/birth defect
** Relation to treatment: 1 = Definitely, 2 = Probably, 3 = Possibly, 4 = Unlikely, 5 = Not related
*** Outcome of AE: 1 = Restored, 2 = Improved, 3 = Unchanged, 4 = Deteriorated, 5 = Death, 6 = Unknown, 7 = Overcome with sequelae
**** Change of study medication: 1 = Paused, 2 = Discontinued, 3 = Reduced, 4 = Increased, 5 = Unchanged, 6 = Unknown, 7 = Not applicable

ADVERSE EVENTS (AE/SAE)

<table>
<tr><th></th><th></th><th colspan="3">Duration</th><th>Grade</th><th>SAE</th><th>SAE-Criteria*</th><th>AE related to [illegible]</th><th>Outcome of [illegible]</th><th>Therapy of AE</th><th>Change of study [illegible]</th></tr>
<tr><td>No.</td><td>Description</td><td>Start date
(dd/mm/yyyy)</td><td>End date
(dd/mm/yyyy)</td><td>ongoing</td><td>1 = mild
2 = moderate
3 = severe</td><td>0 = no
1 = yes</td><td></td><td></td><td></td><td>0 = no
1 = yes
(if yes, please specify and enter on “concomitant medication” sheet)</td><td></td></tr>
<tr><td>|__|</td><td></td><td>|__|__|/|__|__|/|__|__|__|__|</td><td>|__|__|/|__|__|/|__|__|__|__|</td><td>☐</td><td>|__|</td><td>|__|</td><td>|__|</td><td>|__|</td><td>|__|</td><td>|__|</td><td>|__|</td></tr>
<tr><td>|__|</td><td></td><td>|__|__|/|__|__|/|__|__|__|__|</td><td>|__|__|/|__|__|/|__|__|__|__|</td><td>☐</td><td>|__|</td><td>|__|</td><td>|__|</td><td>|__|</td><td>|__|</td><td>|__|</td><td>|__|</td></tr>
<tr><td>|__|</td><td></td><td>|__|__|/|__|__|/|__|__|__|__|</td><td>|__|__|/|__|__|/|__|__|__|__|</td><td>☐</td><td>|__|</td><td>|__|</td><td>|__|</td><td>|__|</td><td>|__|</td><td>|__|</td><td>|__|</td></tr>
<tr><td>|__|</td><td></td><td>|__|__|/|__|__|/|__|__|__|__|</td><td>|__|__|/|__|__|/|__|__|__|__|</td><td>☐</td><td>|__|</td><td>|__|</td><td>|__|</td><td>|__|</td><td>|__|</td><td>|__|</td><td>|__|</td></tr>
<tr><td>|__|</td><td></td><td>|__|__|/|__|__|/|__|__|__|__|</td><td>|__|__|/|__|__|/|__|__|__|__|</td><td>☐</td><td>|__|</td><td>|__|</td><td>|__|</td><td>|__|</td><td>|__|</td><td>|__|</td><td>|__|</td></tr>
<tr><td>|__|</td><td></td><td>|__|__|/|__|__|/|__|__|__|__|</td><td>|__|__|/|__|__|/|__|__|__|__|</td><td>☐</td><td>|__|</td><td>|__|</td><td>|__|</td><td>|__|</td><td>|__|</td><td>|__|</td><td>|__|</td></tr>
<tr><td>|__|</td><td></td><td>|__|__|/|__|__|/|__|__|__|__|</td><td>|__|__|/|__|__|/|__|__|__|__|</td><td>☐</td><td>|__|</td><td>|__|</td><td>|__|</td><td>|__|</td><td>|__|</td><td>|__|</td><td>|__|</td></tr>
</table>

* SAE criteria: 1 = Death, 2 = Life-threatening, 3 = Hospitalization or prolongation of existing hospitalization, 4 = Persistent or significant disability/incapacity, 5 = Congenital anomaly/birth defect

** Relation to treatment: 1 = Definitely, 2 = Probably, 3 = Possibly, 4 = Unlikely, 5 = Not related

*** Outcome of AE: 1 = Restored, 2 = Improved, 3 = Unchanged, 4 = Deteriorated, 5 = Death, 6 = Unknown, 7 = Overcome with sequelae

**** Change of study medication: 1 = Paused, 2 = Discontinued, 3 = Reduced, 4 = Increased, 5 = Unchanged, 6 = Unknown, 7 = Not applicable

18.CHRONOGRAM OF ACTIVITIES

	**Visit 1 Screening**	**Visit 2 Baseline**[a]	**Visit 3 Treatment**						
			**Day 1**	**Day 2-21**	**Day 22**	**Day 23-41**	**Day 42**	***Range Days 43-49***	**End of treatment (**one day after treatment no. 42)
Informed Consents/ Assents (clinical trial and biobanking)	√								
Demographic data	√								
Lymphedema staging	√	√							
Clinical photographs		√							
History of lymphedema	√								
History of ADLA	√								
Circumference - Tape		√							
Circumference - Lymphatech®		√							
Volume of LE - Lymphatech®		√							
Ultrasound		√							
Medical history	√								
Concomitant medication	√	√	√	√	√	√	√	*(√)*	√
History of relevant medications	√								
Vital signs	√	√							
Physical examination	√	√							
QoL		√							
Hygiene status		√							
Lymphedema management training		√							
Laboratory assessment (blood)	√	*(√)*[a]	*(√)*[b]		√ (before treatment no. 22, AST/ALT/γ-GT, range +2 days)	*√ before treatment no. 22, AST/ALT/γ-GT, range +2 days)*[c]	√ (on the last day of treatment, AST/ALT/γ-GT, range +2 days)	*√(on the last day of treatment, AST/ALT/γ-GT, range +2 days)*[d]	*(√)* [d]
Urine sample collected	√						√	*(√)* [d]	*(√)* [d]
Saliva sample collected	√								
Pregnancy test	√	√	*(√)*[b]	√ (on day 15, range + 2 days)		√ (on day 29, range + 2 days)	√ (on day 42, range + 2 days)		
In-/Exclusion criteria	√	√	*(√)*[b]						
Randomization		√							
Presence for visit				√	√	√	√	*(√)*	√
Individual treatment			√	√	√	√	√	*(√)*	
ADLA questionnaire		√	√	√	√	√	√	*(√)*	√
AEs				√	√	√	√	*(√)*	√
End of study record									

[a] Visit 2 (Baseline) should take place max. 28 days after visit 1 (Screening). If that is not the case, blood tests have to be repeated. [b]Visit 3 (Treatment) should start on the same day or one day after visit 2 (Baseline). If that is not the case, the pregnancy test has to be repeated and in case the period between the two visits is > 28 days also blood tests and check of in- and exclusion criteria have to be repeated.
[c] only to be done if not already done on day 22 [d] only to be done if not already done on day 42

	**Visit 4**	**Visit 5**	**Visit 6**	**Visit 7 8 months**	**Visit 8**	**Visit 9**	**Visit 10**	**Visit 11**	**Visit 12**	**Visit 13**	**Visit 14**	**Visit 15 24 months**

	2 months follow-up[a]	4 months follow-up[b]	6 months follow-up[c]	follow-up[d]	10 months follow-up[e]	12 months follow-up[f]	14 months follow-up[g]	16 months follow-up[h]	18 months follow-up[i]	20 months follow-up[j]	22 months follow-up[k]	follow-up[l]
Informed Consents/ Assents (clinical trial and biobanking)												
Demographic data												
Lymphedema staging			√			√						√
Clinical photographs			√			√						√
History of lymphedema												
History of ADLA												
Circumference - Tape			√			√						√
Circumference - Lymphatech®			√			√						√
Volume of LE - Lymphatech®			√			√						√
Ultrasound			√			√						√
Medical history												
Concomitant medication	√	√	√	√	√	√	√	√	√	√	√	√
History of relevant medications												
Vital signs			√			√						√
Physical examination												
QoL						√						√
Hygiene status		√	√			√			√			√
Lymphedema management training		√	√			√			√			√
Laboratory assessment (blood)			√			√						√
Urine sample collected			√			√						√
Saliva sample collected			√			√						√
Pregnancy test	√		√			√						√
In-/Exclusion criteria												
Randomization												
Presence for visit	√	√	√	√	√	√	√	√	√	√	√	√
Individual treatment												
ADLA questionnaire	√	√	√	√	√	√	√	√	√	√	√	√
AEs	√	√										
End of study record												√

[a] the 2 months follow-up should take place 61 days (± 7 days) after treatment day 1
[b] the 4 months follow-up should take place 121 days (± 10 days) after treatment day 1
[c] the 6 months follow-up should take place 182 days (± 21 days) after treatment day 1
[d] the 8 months follow-up should take place 243 days (± 14 days) after treatment day 1
[e] the 10 months follow-up should take place 303 days (± 14 days) after treatment day 1
[f] the 12 months follow-up should take place 364 days (± 21 days) after treatment day 1
[g] the 14 months follow-up should take place 425 days (± 21 days) after treatment day 1
[h] the 16 months follow-up should take place 485 days (± 21 days) after treatment day 1
[i] the 18 months follow-up should take place 546 days (± 21 days) after treatment day 1
[j] the 20 months follow-up should take place 607 days (± 21 days) after treatment day 1
[k] the 22 months follow-up should take place 667 days (± 21 days) after treatment day 1
[l] the 24 months follow-up should take place 728 days (-28; + 42 days) after treatment day 1

19.BUDGET AND SOURCE OF FUNDING

19.1. Proposed **budget**

Budget Items	Proposed budget (CFA)		
	Year1	Year2	Year3
**Personnel**	7,000,000.00	7,000,000.00	7,000,000.00
**Supplies**	1,500,000.00	1,500,000.00	1,800,000.00
**Equipments**	7,000,000.00	-	-
**Patient cost (drugs, hospitalization, etc)**	-	4,000,000.00	2,500,000.00
**Local travel / field work (travel, hotel, perdiem)**	1,000,000.00	2,000,000.00	2,500,000.00
**International travel for research staff**	2,700,000.00	2,700,000.00	2,700,000.00
**Visiting experts (Accomodations)**	-	1,000,000.00	1,000,000.00
**Training (tuition, stipend)**	1,500,000.00	1,500,000.00	1,500,000.00
**Library**	250,000.00	250,000.00	250,000.00
**Data Analysis**	1,200,000.00	1,200,000.00	1,200,000.00
**Other expenditures**	1,500,000.00	1,500,000.00	1,500,000.00
**Total**	**68,250,000.00**		

19.2. Source of funding

SPONSORED BY THE
Federal Ministry of Education and Research

Research Networks for Health Innovations in Sub-Saharan Africa

The design, management, analysis and reporting of the study are entirely independent of the manufacturers of doxycycline and placebo.

The Federal Ministry of Education and Research (BMBF), Germany, has no role in the design of this study and will not have any role during its execution, analyses, interpretation of the data, or decision to submit results.

20. REFERENCES

1. Tekola F, Ayele Z, HaileMariam D, Fuller C, Davey G: **Development and testing of a de novo clinical staging system for podoconiosis (endemic non-filarial elephantiasis)**. *Tropical medicine & international health : TM & IH* 2008, **13**(10):1277-1283.
2. Debrah AY, Mand S, Specht S, Marfo-Debrekyei Y, Batsa L, Pfarr K, Larbi J, Lawson B, Taylor M, Adjei O *et al*: **Doxycycline reduces plasma VEGF-C/sVEGFR-3 and improves pathology in lymphatic filariasis**. *PLoS Pathog* 2006, **2**(9):e92.
3. Mand S, Debrah AY, Klarmann U, Batsa L, Marfo-Debrekyei Y, Kwarteng A, Specht S, Belda-Domene A, Fimmers R, Taylor M *et al*: **Doxycycline improves filarial lymphedema independent of active filarial infection: a randomized controlled trial**. *Clin Infect Dis* 2012, **55**(5):621-630.
4. Dreyer G, Addiss D, Dreyer P, Noroes J: **Basic Lymphoedema Management. Treatment and prevention of Problems Associated with Lymphatic Filariasis.** Hollis, NH, USA: Hollis Publishing Company; 2002.
5. Davey G, Newport M: **Podoconiosis: the most neglected tropical disease?** . *Lancet* 2007, **369**:888-889.
6. Davey G, Tekola F, Newport MJ: **Podoconiosis: non-infectious geochemical elephantiasis**. *Trans R Soc Trop Med Hyg* 2007 **101**(12):1175-1180.
7. Price EW, Henderson WJ: **Endemic elephantiasis of the lower legs in the United Cameroon Republic**. *Trop Geogr Med* 1981, **33**(1):23-29.
8. Wanji S, Tendongfor N, Esum M, Che JN, Mand S, Tanga Mbi C, Enyong P, Hoerauf A: **Elephantiasis of non-filarial origin (podoconiosis) in the highlands of north-western Cameroon**. *Ann Trop Med Parasitol* 2008, **102**(6):529-540.
9. Taylor MJ, Makunde WH, McGarry HF, Turner JD, Mand S, Hoerauf A: **Macrofilaricidal activity after doxycycline treatment of Wuchereria bancrofti: a double-blind, randomised placebo-controlled trial**. *Lancet* 2005, **365**(9477):2116-2121.
10. Turner JD, Mand S, Debrah AY, Muehlfeld J, Pfarr K, McGarry HF, Adjei O, Taylor MJ, Hoerauf A: **A randomized, double-blind clinical trial of a 3-week course of doxycycline plus albendazole and ivermectin for the treatment of Wuchereria bancrofti infection**. *Clin Infect Dis* 2006, **42**(8):1081-1089.
11. Coulibaly YI, Dembele B, Diallo AA, Lipner EM, Doumbia SS, Coulibaly SY, Konate S, Diallo DA, Yalcouye D, Kubofcik J *et al*: **A randomized trial of doxycycline for Mansonella perstans infection**. *N Engl J Med* 2009, **361**(15):1448-1458.

12. Shenoy RK, Kumaraswami V, Suma TK, Rajan K, Radhakuttyamma G: **A double-blind, placebo-controlled study of the efficacy of oral penicillin, diethylcarbamazine or local treatment of the affected limb in preventing acute adenolymphangitis in lymphoedema caused by brugian filariasis**. *Ann Trop Med Parasitol* 1999, **93**(4):367-377.
13. Wanji S, Kengne-Ouafo JA, Datchoua-Poutcheu FR, Njouendou AJ, Tayong DB, Sofeu-Feugaing DD, Amvongo-Adjia N, Fovennso BA, Longang-Tchounkeu YF, Tekola-Ayele F *et al*: **Detecting and staging podoconiosis cases in North West Cameroon: positive predictive value of clinical screening of patients by community health workers and researchers**. *BMC Public Health* 2016, **16**:997.
14. Organization WH: **WHOQOL-BREF: introduction, administration, scoring and generic version of the assessment: field trial version, December 1996**. 1996.
15. Hoerauf A, Volkmann L, Hamelmann C, Adjei O, Autenrieth IB, Fleischer B, Buttner DW: **Endosymbiotic bacteria in worms as targets for a novel chemotherapy in filariasis**. *Lancet* 2000, **355**(9211):1242-1243.
16. Hoerauf A, Mand S, Adjei O, Fleischer B, Buttner DW: **Depletion of wolbachia endobacteria in Onchocerca volvulus by doxycycline and microfilaridermia after ivermectin treatment**. *Lancet* 2001, **357**(9266):1415-1416.
17. Debrah AY, Specht S, Klarmann-Schulz U, Batsa L, Mand S, Marfo-Debrekyei Y, Fimmers R, Dubben B, Kwarteng A, Osei-Atweneboana M *et al*: **Doxycycline Leads to Sterility and Enhanced Killing of Female Onchocerca volvulus Worms in an Area With Persistent Microfilaridermia After Repeated Ivermectin Treatment: A Randomized, Placebo-Controlled, Double-Blind Trial**. *Clin Infect Dis* 2015, **61**(4):517-526.
18. Hoerauf A: **Filariasis: new drugs and new opportunities for lymphatic filariasis and onchocerciasis**. *Curr Opin Infect Dis* 2008, **21**(6):673-681.
19. Harris PA, Taylor R, Thielke R, Payne J, Gonzalez N, Conde JG: **Research electronic data capture (REDCap)--a metadata-driven methodology and workflow process for providing translational research informatics support**. *J Biomed Inform* 2009, **42**(2):377-381.

PART C: ADDITIONAL DOCUMENTS

C-1 INVESTIGATOR'S CVs

CV PRICIPAL INVESTIGATOR

NAME<br>**WANJI SAMUEL**	POSITION TITLE<br>**Professor of Public Health Parasitology and Entomology**
eRA COMMONS USER NAME (credential, e.g., [illegible]	

EDUCATION/TRAINING (*Begin with baccalaureate or other initial professional education, such as nursing, include postdoctoral training and residency training if applicable.)*

INSTITUTION AND LOCATION	DEGREE *(if applicable)*	MM/YY	FIELD OF STUDY
Saint Thomas d'Aquin College, Bafoussam, Cameroon	Baccalaureat	07/1982	Biology-Mathematics
University of Yaounde, Cameroon	Licence	07/1985	Zoology
University of Orleans, France	Maitrise	06/1987	Biology of Organisms and Populations
University of Orsay, France	Certificate	09/1987	Fundamental and Applied Entomology
University of Montpellier, France	Master of Science	08/1989	Parasitology and Vector Biology
University of Montpellier, France	Doctorate (PhD)	12/1992	Parasitology
University of Nantes, France	Habilitation à Diriger des Recherches	05/2007	Publich Health Parasitology

A. Positions and Honors

Positions and Employment

2011: Head of Department Microbiology and Parasitology, University of Buea

2010: Qualified to the Grade of Professor of University in France: Specialty; Public Health Parasitology and Entomology

2008: Associate Professor of Public Health Parasitology and Entomology, Department of Biochemistry-Microbiology, University of Buea, Cameroon

2001-2006 Coordinator of the Biochemistry degree Programme, Department of Biochemistry and Microbiology, University of Buea

Since 2000: Executive Director of the Research Foundation in Tropical diseases and Environment, Buea, Cameroon

2002: Senior lecturer, Molecular and Public Health Parasitology and Entomology, Department of Life Sciences, University of Buea

1998: Lecturer, Molecular and Public Health Parasitology and Entomology Department of Life Sciences, University of Buea

1996: Assistant Lecturer, Molecular and Public Health Parasitology and Entomology Department of Life Sciences, University of Buea

1995: « senior instructor », Molecular and Public Health Parasitology and Entomology Department of Life Sciences, University of Buea

1994: Postdoctoral fellow, department of Pharmacology and Therapeutics, University Teaching Hospital, Enugu, Nigeria

1990-1993: PhD student, «Laboratoire de Biologie Parasitaire et Chimiothérapie, Muséum, National d'Histoire Naturelle, Paris, France».

Other Experience and Professional Memberships

Member of the *American society of Tropical Medicine and Hygiene (ASTMH)* (2003)

Member of the *French Society of Parasitology* (*SFP*) (2003)

Member of the *Cameroonian Society of Parasitology (SCP)* (2002)

Member of the *Interuniversity permanent consultative committee, Cameroon (CCIU)* (2000)

Editorial Board and Reviewers for Scientific journals

-Parasites and Vectors

- PLoS Neglected Tropical Diseases

-American journal of Tropical medicine and Hygiene

Consultancy work

+For the Special Programme for Research&Training in Tropical Diseases (TDR)/ UNICEF/UNDP/World Bank/WHO:

-Development and validation of the Rapid Assessment Procedures for Loiasis (RAPLOA)

-Community-directed interventions for major health problems in Africa

+*For the African Programme for Onchocerciasis Control (APOC)*:

-Mapping of Loiasis using RAPLOA in different African countries (Democratic Republic of Congo, Republic of Congo, Sudan, Angola, Equatorial Guinea, Cameroon)

+ Technical adviser for the African Programme for Onchocerciasis Control

B. Peer-reviewed Publications

1 **Wanji S**., Cabaret J. C., Bonnand N., Bain O.: The fate of the filaria *Monanema Martini* in two rodents hosts: recovery rate, migration and localisation. *Ann. Parasitol. Hum. Comp.*, 1990, 65, 80-88.

2 Vuong P. N., **Wanji S., S**akka L., Klager S., Bain O.: The murid filaria *Monanema Martini:* A model for Onchocerciasis Part I. - Description of lesions. *Ann. Parasitol. Hum. Comp.*, 1991, 66(3), 109-120.

3 Vuong P. N., Traore S., **Wanji S.,** Diarabassoula S., Balaton A., Bain O.: Ivermectin in Human Onchocerciasis: a clinical study of skin lesions three days after treatment. *Ann. Parasitol. Hum. Comp.*, 1992.

4 Spratt D., Vuong P. N., **Wanji S.**, Aimard L., Bain O.: Onchocerciasis-like lesions induced by *Cercopithifilaria Johnstoni* in its natural host and rat. *Ann. Parasitol. Hum. comp.*, 1993.

5 Bain O., **Wanji S.** Petit G., Paperna I., Finkelman S.: Filaires Splendidofilarinae de lézards: Nouvelles espèces, redescription, cycle chez le Phlébotome. *Systematic Parasitology*, 1993, 26: 97-115.

6 Aimard L., **Wanji S.** Vuong P. N., Petit G., Bain O.: Ophtamological study of the lesions induced by filarial worms with dermal microfilariae *Monanema Martini* in its murid hosts. *Current Eye Research,* 1993, 12, 885-891.

7 Wanji S., Vuong P. N., Gantier J. C., Bougnoux M. E., Breton B., Bain O.: Drug trials with *Monanema Martini* on the adult worms, the dermal microfilariae and the natural host.*, Parasite, 1994, vol. I suppl. I., 33.*

8 Achu-Kwi, Daiser W. H., Renz A., Wahl G., **Wanji S**.: Prepatency period and some aspects of epizootiology of *Onchocerca Ochengi* infection in cattle in the Adamaoua Plateau, Cameroon. *Parasite, 1994, vol. I, suppl. I, 10.*

9 Bain O., **Wanji S.**, Vuong P. N., Petit G., Breton B., And Gantier J. C.: Cardio-pulmonary location of lymphatic filariae. *Progress in Lymphology. Sept. 20-26, 1993. Witte, M. H., Witte C. l. (eds). The intern. Soc. Lymphol., Zurich, Switzerland and Tucson, Arizona, 1994.*

10 **Wanji S.,** Gantier J. C., Petit G., Rapp J., Bain O.: The murid filaria *Monanema martini*: a model for Onchocerciasis; relationship between infective larvae, adult worms and microfiladermia. *Trop, Med. Parasitol*., 1994, 45, 107-111.

11 Bain O., **Wanji S.**, Vuong P. N., Marechal P., Legoff L., Petit G.: Larval biology of six filariae of the sub-family Onchocercinae in a vertebrate host. *Parasite,* 1994, 1, 241-254.

12 Vuong P. N., **Wanji S**., Prodhom J., Bain O.: Nodules sous-cutanées et lésions engendrées par diverses Onchocerques chez des bovins africains. *Revue Elev. Med. Vet. Pays Trop.*, 1994, 47(1): 47-51.

13 Breton B., Diagne M., **Wanji S.**, Bougnoux M.E., Chandre F., Marechal P., Petit G., Vuong P.N., Bain O.: Ivermectin and moxidectin in two filarial systems: resistance of *Monanema martini*; inhibition of *Litomosoides sigmodontis* insemination. *Parasitologia*, 1997, 38.

14 Bain O., **Wanji S**., Enyong P. Petit G., Noireau F., Eberhard M. I., Wahl G.: New features on the moults and morphogenesis of the human filaria *Loa loa*: Consequences. *Parasite*, 1998.

15 Fontenille D., **Wanji S**., Djouaka R., Awono-Ambene Hp.: *Anopheles hancocki*, vecteur secondaire du paludisme au Cameroun. *Bulletin de liaison et de documentation de l'OCEAC*, 2000, 33(2), 23-26.

16 Esum M., **Wanji S**., Tendongfor N., Enyong P. Co-endemicity of loiasis and onchocerciasis in the South West Province of Cameroon: implications for the mass treatment with ivermectin. *Transactions of the Royal Society of Tropical Medicine and Hygiene*, 2001, 95, 673-676.

17 **Wanji S**., Takougang I., Yenshu E., Meremikwu M., Enyong P., Braide E., Remme H. (**2001)** Rapid Assessment Procedures for Loiasis. *UNDP/World Bank/WHO Special Programme for Research &Training in Tropical Disease TDR/IDE/RP/RAPL/01.1.*

18 **Wanji S.,** Tendongfor N. Esum M., Enyong P., (**2002**) *Chrysops silacea* biting densities and transmission potential in an endemic area of human loiasis in South-West Cameroon. *Tropical Medicine and International Health*, 7(4), 371-377.

19 **Wanji S**., Tendongfor N., Vuong P.N., Enyong P., and Bain O. The migration and localization of *Loa loa* infective and fourth stage larvae in normal and immuno-suppressed rodents. *Ann. Trop. Med. Parasitol*, 2002, 96(8)**,** 823-830.

20 Takougang I., Meremikwu M., **Wanji S**., Yenshu E., Enyong P., Braide E., Lanlem Sb., Meli J., Kale O., Remme Jh. Rapid Assessment method for prevalence and intensity of *Loa loa* infection. *Bull World Health Organ*, 2002, 80(11), 852-8.

21 **Wanji S**., Tanke N.T., NdindengS., Ajonina C., Tendongfor N., Fontenille D. Anopheles species of the Mount Cameroon region; biting habits, feeding behavior and Entomological inoculation rates. *Tropical Medicine and International Health*, 2003, 8(7), 1-7.

22 **Wanji S**., Tendongfor N, Esum M., NdindengS., Enyong P. Heterogeneity in the prevalence and intensity of loiasis in five contrasting bio-ecological zones of Cameroon. *Transactions of the Royal Society of Tropical Medicine and Hygiene*, 2003, 97, 1-8.

23 **Wanji S.**, Tendongfor N., Esum M., NdindengS., Enyong P. Epidemiology of Concomitant infections due to *Loa loa*, *Mansonella perstans* and *Onchocerca volvulus* in rain forest villages of Cameroon. *Med. Microbiology Immunol* (Berl), 2003, 192(1), 15-21.

24 Buttner D. W., **Wanji S**., Bazzocchi C., Bain O., Fischer P. Obligatory symbiotic Wolbachia endobacteria are absent from *Loa loa. Filaria journal*, 2003, 2(1):10.

25 MCGarry H. F, Gill E., Pfarr, K., Hoerauf A., Akue J. P., Enyong P., **Wanji S**., Kläger S., Bianco T., Klion A., Nutman T., Soboslay P., Beeching N., Taylor M. J. Evidence against *Wolbachia* symbiosis in *Loa loa. Filarial journal*, 2003, 2(1): 9.

26 Brieger W. R., Okeibunor J. C., Abiose A.O., Ndyomugyenyi R., Kisoka W., **Wanji S.**, Elhassan E., Amazigo U. V. Feasibility of measuring compliance to annual ivermectin treatment in the African Programme for Onchocerciasis Control. *Trop Med Int Health*, 2007, 12(2):260-268.

27 Turner J.D., Langley R.S., Johnston K.L., Egerton G., **Wanji S.**, Taylor M.J. Wolbachia endosymbiotic bacteria of Brugia malayi mediate macrophage tolerance to TLR- and CD40-specific stimuli in a MyD88/TLR2-dependent manner. *J Immunol.*, 2006, 177(2): 1240-1249.

28 **Wanji S.,** Tendongfor N., Esum M., Yundze S. S., Taylor M. J., Enyong P. Combined Utilisation of Rapid Assessment Procedures for Loiasis (RAPLOA) and Onchocerciasis (REA) in Rain Forest Villages of Cameroon. *Filaria J.*, 2005, 4(1): 2.

29 Thomson M.C., Obsomer V., Kamgno J., Gardon J., **Wanji S.**, Takougang I., Enyong P., Remme J.H., Molyneux D.H., Boussinesq M. Mapping the distribution of *Loa loa* in Cameroon in support of the African Programme for Onchocerciasis Control. *Filaria J.*, 2004, 3(1): 7.

30 **Wanji S.**, Tendongfor N., Esum M., Che J. N., Mand S., Tanga Mbi C., Enyong P., and Hoerauf A. Elephantiasis of non-filarial origin (podoconiosis) in the highlands of north-west Cameroon. *Annals of Tropical Medicine & Parasitology*, 2008, 102(6), 1-12.

31 Wanji S., Kimbi H.K., Eyong J.E., Tendongfor N., Ndamukong J.L. Performance and usefulness of the Hexagon rapid diagnostic test in children with asymptomatic malaria living in the Mount Cameroon region. *Malar J*., 2008, 7:89.

32 Allen J.E., Adjei O., Bain O., Hoerauf A., Hoffmann W.H., Makepeace B.L., Schulz-Key H., Tanya V.N., Trees A.J., Wanji S., Taylor D.W. Of mice, cattle, and humans: the immunology and treatment of river blindness. *PLoS Negl Trop Dis*., 2008, 2(4):e217.

33 Lhermitte-Vallarino N., Barbuto M., Ineich I., **Wanji S.**, Lebreton M., Chirio L., Bain O. First report of Rhabdias (Nematoda: Rhabdiasoidea) from lungs of montane chameleons in Cameroon: description of two new species and notes on biology. *Parasite*, 2008, 15(4): 553-64.

34 Nkengazong L., Njiokou F., Teukeng F., Enyong P., **Wanji S**. Reassessment of the endemicity level of urinary schistosomiasis in the Kotto-Barombi focus (South-west Cameroon) and impact of mass drug administration (MDA) on the parasitic indices. *Journal of Cell and Animal Biology*, 2009, 3(9), 159-164.

35 Taylor M. J., Awadzi K., Basáñez M.G., Biritwum N., Boakye D., Boatin B., Bockarie M., Churcher T. S, Debrah A., Edwards G., Hoerauf A., Mand S., Matthews G., Osei-Atweneboana M., Prichard R. K., **Wanji S.**, Adjei O. Onchocerciasis Control: Vision for the Future from a Ghanian perspective. *Parasit Vectors*,2009,2(1): 7.

36 **Wanji S**., Mafo F. F., Tendongfor N., Tanga M. C., Tchuente F., Bilong C. F., Njine T. Spatial distribution, environmental and physico-chemical characterization of Anopheles breeding sites in the Mount Cameroon region. *J. vector borne Dis.*, 2009, 46, 75-80.

37 Specht S., **Wanji S.** New insights into the biology of filarial infections. *J Helminthol.*, 2009. 83(2), 199-202.

38 Nkengazong L., Njiokou F., **Wanji S.**, Teukeng F., Enyong P. and Asonganyi T. Prevalence of soil transmitted helminths and impact of Albendazole on parasitic indices in Kotto Barombi and Marumba II villages (South-West Cameroon). *African Journal of Environmental Science & Technology*, 2010, 4 (3), 115-121.

39 Lhermitte-Vallarino N., Barbuto M., Junker K., Boistel R., Ineich I., **Wanji S.,** Bain O. *Rhabdias rhampholeonis* n. sp. and *R. mariauxi* n. sp. (Nematoda, Rhabdiasoidea), first lung worms from leaf chameleons: description, barcoding and notes on biology. *Parasitology International*, 2009a, 58, 375-383.

40 Mackenzie C. D., Huntington M. K., **Wanji S.**, Lovato R. V., Eversole R. R., Geary T. G. The Association of Adult *Onchocerca volvulus* with Lymphatic Vessels. *Journal of Parasitology*, 2010, 96(1), 219-221.

41 CDI STUDY GROUP (**WANJI S**). Community-directed interventions for priority health problems in Africa: results of a multicountry study. *Bull World Health Organ.*, 2010, 88(7), 509-518.

42 Tekle A. H., Zoure H., **Wanji S.**, Leak S., Noma M., Remme J.H., Amazigo U. Integrated rapid mapping of onchocerciasis and loiasis in the Democratic Republic of Congo: Impact on control strategies. *Acta Trop*., 2010, 120 Suppl. 1, S81-S90.

43 Junker K., Lhermitte-Vallarino N., Barbuto M., Ineich I., **Wanji S.**, Bain O. New species of Rhabdias (Nematoda: Rhabdiasidae) from afrotropical anurans, including molecular evidence and notes on biology. *Folia Parasitol (Praha)*, 2010, 57(1), 47-61.

44 Turner J. D., Tendongfor N., Esum M., Johnston K. L., Langley R. S., Ford L., Faragher B., Specht S., Mand S., Hoerauf A., Enyong P., **Wanji S.**, Taylor M. J. Macrofilaricidal activity after doxycycline only treatment of *Onchocerca volvulus* in an area of *Loa loa* co-endemicity: a randomized controlled trial. *PLoS Negl Trop Dis*., 2010, 4(4):e660.

45 Tanga M. C., Ngundu W. I., Judith N., Mbuh J., Tendongfor N., Simard F., **Wanji S**.Climate Change and altitudinal structuring of malaria vectors in south-western Cameroon: their relation to malaria transmission. *Trans R Soc Trop Med Hyg*., 2010, 104(7), 453-460.

46 Attout T., Hoerauf A., Dénécé G., Debrah A. Y., Marfo-Debrekyei Y., Boussinesq M., **Wanji S.**, Martinez V., Mand S., Adjei O., Bain O., Specht S., Martin C. Lymphatic vascularisation and involvement of Lyve-1+ macrophages in the human *Onchocerca* nodule. *PLoS One*, 2009, 4(12):e8234.

47 Brieger W. R., Okeibunor J. C., Abiose A.O., **Wanji S.**, Elhassan E., Ndyomugyenyi R., Amazigo U. Compliance with eight years of annual ivermectin treatment of onchocerciasis in Cameroon and Nigeria. *Parasit Vectors*, 2011, 4(1),152.

48 Zouré H. G., **Wanji S.**, Noma M., Amazigo U. V., Diggle P. J., Tekle A. H., Remme J. H. The Geographic Distribution of Loa loa in Africa: Results of Large-Scale Implementation of the Rapid Assessment Procedure for Loiasis (RAPLOA). *PLoS Negl Trop Dis*., 2011, 5(6):e1210.

49 Ferri E., Bain O., Barbuto M., Martin C-, Lo N., Uni S., Landmann F., Baccei S. G., Guerrero R., de Souza Lima S., Bandi C., **Wanji S.**, Diagne M., Casiraghi M. New insights into the evolution of wolbachia infections in filarial nematodes inferred from a large range of screened species. *PLoS One*, 2011, 6(6), e20843 (1-17).

50 Brieger W. R., Okeibunor J. C., Abiose A. O., Ndyomugyenyi R., **Wanji S.**, Elhassan E., Amazigo U. V. Characteristics of persons who complied with and failed to comply with annual ivermectin treatment. *Trop Med Int Health*, 2012, 17(7), 920-930.

51 **Wanji S.**, Kengne-Ouafo A. J., Eyong E. E., Kimbi H. K., Tendongfor N., Ndamukong-Nyanga J. L., Nana-Djeunga H. C., Bourguinat C., Sofeu-Feugaing D. D., Charvet C. L. Genetic diversity of *Plasmodium falciparum* merozoite surface protein-1 block 2 in sites of contrasting altitudes and malaria endemicities in the Mount Cameroon region. *Am J Trop Med Hyg*, 2012, 86(5), 764-774.

52 Tchounkeu Y. F., Onyeneho N. G., **Wanji S.**, Kabali A. T., Manianga C., Amazigo U. V., Amuyunzu-Nyamongo M. Changes in stigma and discrimination of onchocerciasis in Africa. *Trans R Soc Trop Med Hyg*, 2012, 106(6), 340-347.

53 Tamarozzi F., Tendongfor N., Enyong P. A., Esum M., Faragher B., **Wanji S.**, Taylor M. J. Long term impact of large scale community-directed delivery of doxycycline for the treatment of onchocerciasis. *Parasit Vectors*, 2012, 5:53.

54 Davey G., Bockarie M., **Wanji S.**, Addiss D., Fuller C., Fox L., Mycoskie M., Gruin M., Tsegaye A., Ayele F. T., Newport M. Launch of the international podoconiosis initiative. *Lancet*, 2012, 379(9820):1004. No abstract available.

55 Tendongfor N., **Wanji S.**, Ngwa J. C., Esum M. E., Specht S., Enyong P., Matthaei K. I., Hoerauf A. The human parasite *Loa loa* in cytokine and cytokine receptor gene knock out BALB/c mice: survival, development and localization. *Parasit Vectors*, 2012, 5:43.

56 **Wanji S.**, Akotshi D. O., Mutro M. N., Tepage F., Ukety T. O., Diggle P. J., Remme J. H. Validation of the rapid assessment procedure for loiasis (RAPLOA) in the democratic republic of Congo. *Parasit Vectors*, 2012, 5:25.

57 Albers A., Esum M. E., Tendongfor N., Enyong P., Klarmann U., **Wanji S.**, Hoerauf A., Pfarr K. Retarded Onchocerca volvulus L1 to L3 larval development in the *Simulium damnosum* vector after anti-wolbachial treatment of the human host. *Parasit Vectors*, 2012, 5:12.

Research Support

Research Grant Executed

1996/1998: “Vaccination against filarial infection: Use of *Loa loa* and murine model for the identification of protective mechanisms and antigens”

Sponsorisé par la Commission Européenne, Science, Recherche et développement RTD: EU INCO-DC. Contract N° IC 18-CT95-0026

1998/99: récipiendaire de la bourse post-doctorale “bourse d’execellence” de l’AUPELF-UREF “Francophonie Universitaire et de la recherche” pour six mois de stage en France pour apprendre les techniques de biologie moléculaire appliquées aux parasitoses. **Stages à:** INRA Toulouse, Laboratoire de Pharmacologie Expérimentale et de Toxicologie; IRD Montpellier: Laboratoire de Parasitologie et Entomologie Moléculaire.

1998/2001: “Evaluation du couple Loa-Mandrill comme un nouveau outil de criblage tertiaire des candidats vaccins et nouveaux médicaments contre les filaires” sponsorisé par AUPELF-UREF “Francophonie Universitaire et de la recherche” Jeune equipe de recherche (jer) 3011

2000/2001: “Etudes comparée de différentes méthodes de diagnostic de la loase dans différents faciès épidémiologiques au Cameroun”. UNDP/World Bank/WHO Special Programme for Research and training in Tropical diseases (TDR) ID No. A00555

2000/2005: “Vaccination against Onchocerciasis (River Blindness) (VARBO)”: sponsorise par la Commission Européenne, EU INCO-D EC Contract IC A4CT 1999-10002: **Contractor**

2002/2005: “ANTIBIOTIC targeting of Wolbachia endosymbiotic bacteria as a new approach to the treatment of filarial infection and disease”

Sponsorisé par la commission Européenne, EU INCO-DC. Contract N° IC A4-CT20002-10031: **sub-contractor**

2002/2004: “Etude de la Biologie et du rôle vectoriel des Anophèles en zone d’altitude: Comparaison de deux zones biogéographiques au Cameroun et à Madagascar” : sponsorise par le Programme d’Action de soutien à la Formation et à la Recherche du Fonds International de Cooperation Universitaire (FICU), AUF

2002/2004: “Paludisme d’altitude”

Sponsored by the programme « Jeune équipe associée de l’Institut de Recherche pour le développent » (IRD”), France

2006: “Compliance to annual ivermectin treatment”. Sponsored by the African Programme for onchocerciasis control (APOC)

2004/2007: « Les interventions sanitaires sous directives communautaires contre les problèmes majeurs de santé publique au Cameroon:Sponsored byUNDP/World Bank/WHO Special Programme for Research and training in Tropical diseases (TDR)/TDA30101H

2004/2010: Development of Loa/primate models for the investigations of Ivermectin SAEs in Loa loa Patioents: Sponsored by Mectizan Donation Programme

2009/2011: “Wolbachia endobacteria in filarial infections-Exploring their usefulness as targets for novel chemotherapies that are antifilarial pathology and interrupt transmission” *Sponsored by the Volkswagen Foundation Ref (I/84159)*

2009/2012: “NO BUG-Novel release system and Bio-based Utilities for insect repellent textiles and Garments”, *Sponsored by the European Commission, FP7 programme*

2010/2014: “Enhanced Protective immunity against filarial infections” *Sponsored by the European Commission, FP7 programme*

2011/2013: Genetic monitoring for ivermectin resistance in *Onchocerca volvulus*/Development ofMarkers for Ivermectin Resistance monitoring in *Onchocerca volvulus* (MIRO): sponsored byUNDP/World Bank/WHO Special Programme for Research and training in Tropical diseases (TDR)/TDAA90485_

CV CO-INVESTIGATOR

CURRICULUM VITAE

1. NAME: ENYONG **Date of birth**: 27/01/1942

SURNAME: AYUK PETER **Nationality**: CAMEROON

Sex: MALE

2. Degree(s) (*subjects, Universities or school, Year*)

-Doctorate Degree, Animal Biology: Entomology, 1987, University of Paris XI - Orsay, France

-M.Sc. Chemistry/Biology, - Entomology, 1975, University of Paris XI- Orsay, France

-BSc. Chemistry/Biology, 1973, University of Paris XI - Orsay, France

-Certificate - Applied Medical Entomology, 1974, University of Paris XI, Orsay, France

3. Professional Experience

- 1987-1993 - Deputy Chief of Station, Tropical Medicine Research Station , Kumba, Cameroon
- 1993 - 2003 Chief of Station, Tropical Medicine Research Station in Kumba
- Since 2000: Deputy Director of the Research Foundation in Tropical diseases and Environment, Buea, Cameroon

Selected References

- KATABARWA M. N., EYAMBA A., NWANE P., **ENYONG P.**, YAYA S., BALDIAGAÏ J., MADI T. K., YOUGOUDA A., ANDZE G. O-, AND RICHARDS F. O.: (2011) Seventeen Years of Annual Distribution of Ivermectin Has Not Interrupted Onchocerciasis Transmission in North Region, Cameroon. *Am. J. Trop. Med. Hyg*., 85 (6), 1041-1049.

- COFFENG L. E., FOBI G., OZOHC G., BISSEK A. C., NLATTÉ B. O., ENYONG P., OLINGA J. M., ZOURÉ H. G. M., HABBEMA J. D. F., STOLK W. A., DE VLAS S. J., BOUSSINESQ M., NOMA M. : (2012) Concurrence of dermatological and ophthalmological morbidity in Onchocerciasis. *Trans. R. Soc. Trop. Med. Hyg*., 106 (6), 243-251.

-WANJI S**.,** TENDONGFOR N., ESUM M., **ENYONG P**.: (2002) *Chrysops silacea* biting densities and transmission potential in an endemic area of human loiasis in South-West Cameroon. *Tropical Medicine and International Health*, 7(4), 371-377**.**

-WANJI S., TENDONGFOR N., ESUM M., NDINDENG S. AND **ENYONG P**.:(2003) Heterogeneity in the prevalence and intensity of loiasis in five contrasting bio-ecological zones of Cameroon. *Trans R Soc Trop Med Hyg*, 97, 1-8.

- NKENGAZONG L., NJIOKOU F., TEUKENG F., **ENYONG P.,** WANJI S.: (2009) Reassessment of the endemicity level of urinary schistosomiasis in the Kotto-Barombi focus (South-west Cameroon) and impact of mass drug administration (MDA) on the parasitic indices. *Journal of Cell and Animal Biology*, 3 (9), 159-164.

CV TRIAL COORDINATOR

CV Achim Hörauf, Prof. Dr. med.
Date of Birth July 26, 1962

Director Institute for Medical Microbiology, Immunology and Parasitology (IMMIP), University Hospital of Bonn, Sigmund-Freud Str. 25, 53105, Bonn
Telephone: +49 228 287 15673
E-mail: achim.hoerauf@ukb.uni-bonn.de

Academic positions

2003-present Full Professor (C4) and Director of the Institute for Medical Microbiology, Immunology and Parasitology (IMMIP), University Hospital of Bonn
2001-2003 Head, Department of Helminthology, Bernhard Nocht Institute for Tropical Medicine (BNI), Hamburg
1995-2001 Independent laboratory head, BNI, Hamburg
1990-1994 Fellow and post-doc, Institute for Clinical Microbiology, Immunology and Hygiene, University of Erlangen

Academic education

1998 Habilitation in Medical Microbiology and Immunology at the University Hamburg

1997 Fellow of Medical Microbiology and Infection Epidemiology

1987-1989 MD in clinical immunology at Medical Clinic III, University of Erlangen

1983-1989 Study of Medicine at the University of Erlangen including exchange rotations abroad at Duke University in Durham, NC, USA and Galway University, Ireland

Funding (ongoing in the last 5 years, sorted by expiry)

2016 - 2021 PI, Research networks for health innovation in Subsaharan Africa – Tackling the Obstacles of Filariasis and Podoconiosis Disease Control (TAKeOFF), (German Ministry for Research and Education, “BMBF”).
2011 – 2020 Coordinator and PI, partner site (“Standort”) Bonn-Cologne, Deutsches Zentrum für Infektionsforschung (DZIF) (German Ministry for Research and Education, “BMBF”).
2006 – 2020 Principal Investigator, German Quality Control for parasite serology (“Ringversuche in der Parasitenserologie”) - Cooperation project with INSTAND e.V., Düsseldorf, the official partner in laboratory QM for the German Board of Medical Doctors (“Bundesärztekammer).
2013 – 2018 Steering Committee Member, PI, Excellence Cluster 1023 “Immunosensation” (DFG).
2013 – 2018 PI, “In vivo evaluation of anti-filarial drugs” (funded by “Drugs for Neglected Disease Initiative”, *DNDi*).
2015 – 2018 PI, “In vitro and in vivo support for Macrofilaricidal Drug Accelerator” (Bill & Melinda Gates Foundation, BMGF).
2010 – 2017 PI, Project “Death to Onchocerciasis and Lymphatic Filariasis” (DOLF) (BMGF).
2007 – 2016 PI, Anti-Wolbachia Consortium (A-WOL), Project “Anti-symbiotic treatment of filariasis" (BMGF).
2009 – 2016 Co-PI, Project “Genetics of Lymphedema and Hydrocele in Filariasis” (DFG).
2009 – 2016 Co-PI, Project “Post-Genomic Strategies for New Antibiotic Drugs and Targets” (DFG Forschergruppe 854).
2008 – 2016 PI, Project “Molecular mechanisms of IgG4 induction by human antigen-specific regulatory T cells” (DFG).
2013 – 2015 Coordinator and PI, Project “Rapid and high-throughput diagnosis of Onchocerca volvulus infections” (BMGF).

2010 – 2014 PI, Project “Enhanced Protective Immunity Against Filariasis” (EPIAF) (European Commission).
2010 – 2014 PI, SFB 704, TP A3, “Identification of helminth components which induce antigen-specific regulatory T cells“ (DFG).
2007 – 2012 PI, Project *“Wolbachia* endobacteria in filarial infections” (Volkswagen Foundation).

Awards, Activities and Honours (Selection)

1999 Main bi-annual award, German Society for Tropical Medicine (DTG)

2001 Martini-Prize (bi-annual) for best translational research of the University Clinic Eppendorf/Hamburg

2002 Main annual award, German Society for Hygiene und Microbiology (DGHM)

2010-present Coordinator, Bonn-Cologne site, "German Center for Infectious Diseases Research" ("Deutsches Zentrum für Infektionsforschung", DZIF)

2010-2016 President elect / President /Past President, Paul Ehrlich Society for Anti-infective Chemotherapy ("Paul Ehrlich Gesellschaft für Chemotherapie", PEG e.V.)

2015 Memento Prize for Neglected Diseases BUKO Initiative and DAHW ("Deutsches Aussätzigen Hilfswerk") for NTDs

2015 The Innovation Award 2015 by BioRegions, North-Rhine-Westfalia, Germany

2016 Congress President, Annual Meeting of the German Society of Tropical Medicine, (DTG), Bonn, 7-8 October 2016

2016-2019 Member of the DFG Fachkollegium "Mikrobiologie, Virologie und Immunologie"

Selected publications

a) In peer review journals

1. Kroidl I, Saathoff E, Maganga L, Makunde WH, **Hoerauf A**, Geldmacher C, Clowes P, Maboko L, Hoelscher M. 2016. Effect of *Wuchereria bancrofti* infection on HIV incidence in southwest Tanzania: a prospective cohort study. Lancet, doi: 10.1016/S0140-6736(16)31252-1

2. Debrah AY, Specht S, Klarmann-Schulz U, Batsa L, Mand S, Marfo-Debrekyei Y, Fimmers R, Dubben B, Kwarteng A, Osei-Atweneboana M, Boakye D, Ricchiuto A, Büttner M, Adjei O, Mackenzie CD, **Hoerauf A**. 2015. Doxycycline leads to sterility and enhanced killing of female *Onchocerca volvulus* worms in an area with persistent microfilaridermia after repeated ivermectin treatment: A randomized, placebo-controlled, double-blind trial. Clin Infect Dis. 61:517-26.

3. Arndts K, Specht S, Debrah AY, Tamarozzi F, Klarmann Schulz U, Mand S, Batsa L, Kwarteng A, Taylor M, Adjei O, Martin C, Layland L, **Hoerauf A.** 2014. Immunoepidemiological profiling of onchocerciasis patients reveals associations with microfilaria loads and ivermectin intake on both individual and community levels. PLoS Negl Trop Dis. 8(2):e2679.

4. Mand S, Debrah AY, Klarmann U, Batsa L, Marfo-Debrekyei Y, Kwarteng A, Specht S, Belda-Domene A, Fimmers R, Taylor M, Adjei O, **Hoerauf A**. Doxycycline improves filarial lymphedema independent of active filarial infection: A randomized controlled trial. Clin Infect Dis. 2012;55(5):621-30.

5. **Hoerauf A**. 2009. *Mansonella perstans*--the importance of an endosymbiont. N Engl J Med 361:1502-4.

6. Taylor MJ, Makunde WH, McGarry HF, Turner JD, Mand S, **Hoerauf A**. 2005. Macrofilaricidal activity after doxycycline treatment of *Wuchereria bancrofti*: a double- blind, randomised placebo-controlled trial. Lancet 365:2116-21

7. Debrah AY, Mand S, Specht S, Marfo-Debrekyei Y, Batsa L, Pfarr K, Larbi J, Lawson B, Taylor M, Adjei O, **Hoerauf A**. Doxycycline reduces plasma VEGF-C/sVEGFR-3 and improves pathology in lymphatic filariasis. PLoS Pathog. 2006 Sep;2(9):e92.

8. Taylor MJ, Makunde WH, McGarry HF, Turner JD, Mand S, **Hoerauf A**. Macrofilaricidal activity after doxycycline treatment of *Wuchereria bancrofti*: a double-blind, randomised placebo-controlled trial. Lancet. 2005 Jun 18-24;365(9477):2116-21.

9. **Hoerauf A**, Mand S, Adjei O, Fleischer B, Büttner DW. Depletion of *wolbachia* endobacteria in *Onchocerca volvulus* by doxycycline and microfilaridermia after ivermectin treatment. Lancet. 2001 May 5;357(9266):1415-6.

10. **Hoerauf A**, Volkmann L, Hamelmann C, Adjei O, Autenrieth IB, Fleischer B, Büttner DW. Endosymbiotic bacteria in worms as targets for a novel chemotherapy in filariasis. Lancet. 2000 Apr 8;355(9211):1242-3.

b) Patents

Granted patents

Hörauf A, Pfarr K, König G, Specht S, Schiefer S, Schäberle T, Schmitz A, Kehraus S. *Compounds for use in the treatment of filariasis* (Therapy). U.S.A. Patent US 9 168 244 B2

Patents pending

Hörauf A, Pfarr K, König G, Specht S, Schiefer S, Schäberle T, Schmitz A, Kehraus S. *Compounds for use in the treatment of filariasis* (Prophylaxis). U.S.A. Patent-pending: US 14 658 272

Hörauf A, Pfarr K, König G, Specht S, Schiefer S, Schäberle T, Schmitz A, Kehraus S. *Compounds for use in the treatment of filariasis*. E.U. Patent-pending: EP 12 721 456.7

CV TRIAL CLINITIAN 1

Dr DJIKEUSSI KATCHO TATIANA
Date de naissance : 07 janvier 1986
Contact : (+237) 675498532/ 655190562
Email : tdjikeuss@gmail.com
Mariée, 2 enfants (5 ans et 6mois)

I- Domaine de compétence :

Médecine générale, Epidémiologie, Sante publique (Médecine préventive, vaccinologie, surveillance épidémiologique), Médecine des voyages, Economie de la santé (Financement basé sur la performance), Administration des services hospitaliers
Bilingue (Français et Anglais).

II- Expériences professionnelles :

Avril 2013 à 2015- Centre de Vaccinations Internationales de Douala- Médecin chef
- coordination des activités de vaccinations internationale, de consultation et de conseil en médecine des voyages

- Coordination des activités du programme Elargi de Vaccination dans la Région du Littoral.

- Elaboration du plan de contingence pour l'amélioration de la couverture vaccinale au Littoral.

- Participation au Projet de renforcement du PEV de routine dans les zones insulaires au Littoral.

- Participation au Project de Riposte à l'épidémie de Rougeole à Manoka Littoral

Mars 2012 à 2015- Cadre d'appui au Centre de Prévention et de la Lutte contre les épidémies - Délégation régionale de la santé publique du Littoral

- Campagne de sensibilisation de masse sur les techniques de potabilisation de l'eau et le cholera dans les marchés et écoles du Wouri

- Préparation de la prise en charge gratuite et efficace des cas de choléra dans les CTC de la Région.

- Supervisions des activités des mobilisateurs communautaires chargés de la distribution des comprimés Aquatabs dans les District de Santé du Wouri.

- Mise en place d'un dispositif de lutte contre la vente illicite des eaux en sachets en collaboration avec le MINEE ET MINCOMMERCE.

- Collecte, traitement et analyse des données des districts sur mes Maladies à Potentiels Epidémiques.

- Supervision des activités de Surveillance Intégrée de Maladies et réponse.
- Formateur national pour la préparation à la riposte contre la maladie à virus Ebola (Membre de l'équipe d'élaboration des directives nationales et du dispositif de riposte au port et aéroport international de Douala).

Juillet 2012 à 2015: Coordination des activités de lutte contre la cécité dans la Région du Littoral
- collecte et analyse des données de soins ophtalmologiques

- campagnes de chirurgies de la cataracte

- campagnes de dépistage des troubles de la vue en milieu scolaire estudiantin

- planification et évaluation régionale du programme de lutte contre la cécité

- Enquête de base régionale sur la prévalence de la cécité évitable et les ressources humaines et matérielles en soins oculaires

Mars 2011 à Mars 2012 : Médecin vacataire en service de médecine et au centre de traitement agrée des PVVIH à L'hôpital Laquintinie de Douala

Expériences en cliniques hospitalières

 Clinique gap santé de Bonamoussadi

 Polyclinique le Maïmounide

 Centre pédiatrique et neurologique du Chatelet à Bonaberi

III –Education

 Universitaire

➢ **2015 -2017** :CDC CERTIFICATE in Field Epidemiology Training Program, MINSANTE-CDC Atlanta

➢ **2015 – 2017** : Master of sciences in epidemiology : University of Buéa Cameroon

➢ **2013- 2014** : Master de santé Publique option Epidémiologie Recherche Clinique et évaluation (Ecole de santé Publique de Nancy)

➢ **2012- 2013** : DIU prise en charge de l'infection à VIH - Faculté de médecine de l'Université de Montpellier I

➢ **2009 – 2010 :** Doctorat en Médecine Générale - Université Des Montagnes- Cameroun

➢ **2007 – 2009:** Master en sciences de la santé – Université Des Montagnes

➢ **2003 – 2007 :** Licence en sciences de la santé – Université Des Montagnes

 Secondaire second cycle

1997- 2003 : Baccalauréat de l'enseignement général série D au Lycée Bilingue de Mbouda.

IV- Training

➢ Formation en urgences et premiers secours de la Croix Rouge Camerounaise (Yaoundé 2011)

➢ Formation en Surveillance Intégrée des maladies et riposte par l'OMS dans le cadre du projet SURVAC (Renforcement de la surveillance en Afrique Centrale _Douala Mars 2013)

➢ Formation des formateurs nationaux en santé Maternelle et Infantile par l'ONG PEPFAR et MINSANTE (Douala Juillet 2013)

➢ Cours international sur le Financement Basée sur la Performance par la Banque Mondiale et Fonds Régional Pour la promotion de la santé au Littoral _ Douala Septembre 2013)

➢ Formation des formateurs nationaux pour la riposte contre la fièvre Hémorragique à Ebola (CDC Atlanta et Ministère de la santé Direction de la lutte contre la maladie, OMS _ Douala Aout 2014)

➢ Formation des formateurs nationaux pour la gestion des urgences sanitaires et catastrophes, Ministère de la santé Direction de la lutte contre la maladie, CDC_ Yaoundé 2015

➢ Formation en épidémiologie de terrain Ministère de la santé Direction de la lutte contre la maladie, CDC_ Yaoundé 2015 à 2017

➢ Formation en Quantum GIS pour cartographie et georeferencement, Ministère de la santé, Direction de la lutte contre la maladie, CDC_ Yaoundé 2017

V- Autres

Outils informatique : Microsoft Word, Microsoft Excel, Access, Epi info.
Vie associative : Membre du corps des médecins Camerounais, membre de l'association des insuffisants rénaux dialysés et transplantés (Conseiller technique), membre de la Société Internationale de Médecine des Voyages.

VI- REFERENCES

1. DR ETOUNDI, MD Urgentiste
Directeur de la lutte contre la maladie, les épidémies et les pandémies, Ministère de la santé Publique du Cameroun Yaoundé, Cameroun

Email: dretoundi@yahoo.fr
Tel (mobile): 237 677702167

2. Dr. SUME Gerald ETAPELONG, MD, MPH, MFELTP
OMS, Yaoundé, Cameroun
Email: sumegerald@gmail.com
Tel (mobile): 237674 18 01 11/Tel (mobile): 237 694 23 82 04/Tel (Office): 237 33 42 36 33.

3. Pr. Wanji Samuel, Professeur Parasitologie
Département de santé publique, de parasitologie et de biologie des affections transmises par des vecteurs, Université de Buea
Email: swanji@yaoo.fr , **Tel (mobile): 237 /694727715**

CV TRIAL CLINITIAN 2

(+237) 674 29 51 39 |
vofobrice@gmail.com

vofobrice@yahoo.fr

VOFO NGUEDIA BRICE

CIVIL STATUS	Date/Place of birth	:	16 October, 1989/ Bamenda
	Nationality	:	Cameroonian
	Gender	:	Male
	Marital status	:	Married

Career objective

I am eager to pursue a career in clinical medicine including medical research. During my early years in research, I combined clinical skills and laboratory practice in the execution of my final year research thesis and since then, that has been my aspiration. In achieving this, I have shown myself to be dynamic and persistent in achieving my goals, whatever may be. I would readily rate myself as a fast learner and a fine doctor, patient and hardworking, with good basic clinical, surgical, laboratory, and radiological skills. A strong background working with minority and low income population. I have also demonstrated strong leadership and interpersonal skills, a firm sense of responsibility and capacity to work under pressure, in the various leadership positions I have occupied. I am perfectly bilingual, and possess strong verbal and written communication skills.

education

Medical Doctor Degree. [2008-2015]

UNIVERSITY OF BUÉA, CAMEROON.

Title of qualification: Doctor of Medicine.

Thesis: **"calabar chalk" consumption among pregnant women in the Yaoundé Central and Buea Regional Hospitals: prevalence, association with lead levels in the umbilical cord and foetal outcomes""**

Course work: General Medicine

Certificates in Research ethics and Good Clinical Practice.

- **Participation at the Good Clinical Practice workshop organised by the Food and Drugs Authority of Ghana, 2017.**
- **Training from the TREE Training Program in Research Ethics Evaluation**: Modules 1, 2, 3.1, 3.2 and 3.3
- **Training on Ethics of Research Involving Human Participants.**

G.C.E. Advance Level. [2006-2008]
Presbyterian Secondary School Mankon, BAMENDA, CAMEROON.
All 5 subjects: Further Math (A), Math (A), Physics (A) Biology (B), Chemistry (B)

G.C.E. Ordinary Level. [2001-2006]
Government Bilingual High School Mankon, BAMENDA, CAMEROON.
All 11 subjects: Maths(A), Physics(A), Chemistry(A), Biology(A), History(A) Geography(A), French(A), English(B), Additional maths(B), Economics(B), Religion(B)

professional experience

1. **WORK AS MEDICAL DOCTOR| AUGUST 2016 TILL DATE**
   SUB-DIVISIONAL HEALTH CENTER NTAM
2. **WORK AS MEDICAL DOCTOR |** OCTOBER 2015 –JULY 2016
   CAMEROON BAPTIST CONVENTION, HEALTH BOARD.
   My duties were consultation, ward rounds, surgeries, calls, and assisting specialists in patient care and research.

Skills

1. **Medicine (Technical skills)**
   - Good clinical skills in the management of infectious and non-infectious disease, emergency medicine, critical care.
   - Good basic surgical skills in a variety of surgeries e.g lipomectomies, appendectomies, caesarean sections, and hernia repairs.
   - Good in the interpretation of x-rays, CT scans, MRI. Also trained in conducting ultrasound scans.
   - Basic ophthalmology assessment skills (visual field, visual acuity, etc)

2. **Information Technology:**
   - Competent and experienced in **Microsoft Office** (Word, Excel, PowerPoint)
   - Highly Competent in **Epi info** and **SPSS** statistical software
   - Great mastery of Internet Operations
   - Contemporary and passionate about new technologies

3. **Leadership and Interpersonal skills:**
   - Strong skills to collaborate with people from different walks of life
   - **Class delegate** all through the seven years of Medical School
   - Academic affairs adviser, of the Faculty of Health Sciences, University of Buea. 2010-2011.
   - Faculty Vice president, 2011-2012
   - **Faculty President** 2012-2013.

- Honours/Awards: Student leadership Honours, 2012-2013
- South west Youth Elite Representative to the National Assembly, 2013

4. Professional:

- Cooperative, flexible and team work conscious.
- Strong work ethic.
- Work without supervision and can work under varied conditions
- Self-confident and able to handle stress/failure/rejection

5. **Communication:**

- **English** – Academic language (speak, write, read) : **Excellent**
- **French** – Home language (speak, write, read): **Excellent**
- Express creative ideas verbally and in writing
- Write medical reports.

Publications

Awareness of breast cancer and breast self-examination among female undergraduate students in a higher teachers training college in Cameroon. *Pan African Medical Journal* 2017.

Spontaneous Expulsion of Foetal Bones 3 months after Termination of Pregnancy: *International journal of clinical and medical imaging IJCMI* 2014,1-225.

Professional membership

Cameroon Medical Council | 2016

HOBBIES AND OTHERS

Sports (lawn Tennis)

Competent Chest player

Technology (computer, smartphones, applications)

Travelling

Watching movies

Driver’s license category B

References **Available on demand**

CV TRIAL CLINITIAN 3

CURRICULUM VITAE

Name: FOZAO AFOU ADOLPH.
Profession: Medical Doctor.
Address: District Health Service Bafut, North West Region, Cameroon.
Telephone: +(237) 677 80 21 59
e-mail : fozaoafouadolph@yahoo.com

EDUCATION AND QUALIFICATIONS

1992: Doctorate of Medicine - Faculty of Medicine and Biomedical Sciences University of Yaounde.
1984 : General Certificate of Education Advance Level - Cameroon College of Arts and Sciences Kumba.
1982 : General Certificate of Education Ordinary Level - Our Lady Seat of Wisdom College Fontem.
1977: First School Leaving Certificate - Presbyterian Primary School Fosimondi.

WORK EXPERIENCE

31/03/2014 UNTIL NOW: DISTRICT MEDICAL OFFICER BAFUT
23/03/2010 TO 31/03/2014: DISTRICT MEDICAL OFFICER BALI
24/08/2001 TO 23/03/2010: DISTRICT MEDICAL OFFICER NDU
29/03/1993 TO 24/08/2001: SUB-DIVISIONAL CHIEF OF SERVICE PUBLIC HEALTH TUBAH

OTHER KNOWLEDGE

2017: Certificate in performance Based Financing (**PBF**) with Distinction - Douala
2010: Certificate Medical Advisor Mutual Health - Yaounde
2009: Certificate on Mid-Level management course for EPI Managers - Bamenda
1997: Certificate in Management for the Health Care Sector. Inter-University Institute for Training and Development Brussels Belgium.
1995: Participated in a workshop on Early Diagnosis of African Trypanosomiasis in Women. Geneva
1993: Certificate in Clinical Family Planning - Bamenda

STUDY AND WORKING LANGUAGES

FIRST LANGUAGE: ENGLISH
SECOND LANGUAGE: FRENCH

Bafut the, 31/07/2017

CV TRIAL CLINITIAN 4

CURRICULUM VITAE

Name PUNJOM NJEFI YVES
Profession Medical Doctor
Address: Bafut District Hospital, North West Region, Cameroon
Telephone: +(237) 675 07 92 80
e-mail:

EDUCTION AND QUALIFICATIONS

2015: Advanced Masters in Public Health Methodology(Hons) – Université Libres de Bruxelles

2010: Doctorate of Medicine – Faculty of Medicine and biomedical Sciences(Hons), University of Yaounde I

2003: General Certificate of Education Advanced Level – St. Bedes College, Ashing Kom

2001: General Certificate of Education Ordinary Level – Bishop Rogan College, Buea

1996: First School Leaving Certificate – St. Michal`s Primary School, Nkwen

WORK EXPERIENCE

31/03/2014 TILL DATE: DIRECTOR – BAFUT DISTRICT HOSPITAL

25/05/2013 TO 31/03/2014: INTERIM DIRECTOR – BATIBO DISTRICT HOSPITAL

24/12/2010 TO 25/05/2013: MEDICAL DOCTOR – BATIBO DICTRICT HOPITAL

OTHER KNOWLEDGE

2017: Regional Trainer for North west Fund for Health in PMTCT, Family Planning and Emergency Obstetric and neonatal Care(EmONC).

2012 TO DATE: National Trainer for PEPFER in PMTCT, Family Planning and Emergency Obstetric and neonatal Care(EmONC).

2012: Training on the appropriate use of Malaria Rapid tests
Training Management and screening of HIV and Tuberculosis

STUDY AND WORKING LANGUAGES

FIRST LANGUAGE: ENGLISH

SECOND LANGUAGE: FRENCH

28/07/2017

[signature]

C-2 INVESTIGATOR'S BROCHURE

INVESTIGATOR'S BROCHURE

A double-blind, randomized, 24-month study, to compare the efficacy of doxycycline once daily for 6 weeks versus placebo in improving non-filarial lymphedema due to podoconiosis

Research Networks for Health Innovations in Sub-Saharan Africa

SPONSORED BY THE Federal Ministry of Education and Research

TAKeOFF Tackling the Obstacles to Fight Filariasis and Podoconiosis

ukb universitäts klinikumbonn Institute for Medical Microbiology, Immunology and Parasitology

University of Buea – Université de Buea – Knowledge with Wisdom

Author: Pr Samuel Wanji

Edition Number:1

Release Date: November 2017

1. Introduction

Podoconiosis (endemic non-filarial elephantiasis) is a non-infectious geochemical disease arising in barefoot subsistence farmers who are in long-term contact with irritant red clay soil of volcanic origins (Davey, 2007a, 2007 b). The disease causes progressive bilateral swelling of the lower legs. Mineral particles absorbed through skin are taken up by macrophages into the lymphatic system and result in an inflammatory process leading to fibrosis and obstruction of the vessels. This leads initially to swelling of the foot and the lower leg, which progresses to elephantiasis: gross lymphoedema with mossy and nodular changes of the skin. Podoconiosis affects some 4 million people in Africa, Latin America, and a few areas of Asia. It is found in more than ten countries across tropical Africa where irritant soils have been generated by environmental conditions of high altitude (>1,000m) and high annual rainfall (>1,000mm), and are farmed by very poor people who cannot afford shoes or water. Podoconiosis has been described in ten countries across tropical Africa, and has also been reported in tropical areas of Central America and Northern India. The exact global burden is still to be measured, but it is estimated that at least 4 million people are affected worldwide. Cameroon is thought to be among the countries with the highest burden of podoconiosis (Price,1981, Wanji et al, 2008). Due to lack of understanding of the geographical distribution of the disease, intervention against the disease is minimal.

The current control strategy for podoconiosis consists of integrated approach prevention among school age children through promotion and distribution of protective shoe and lymphedema management. Current treatment practices of LE rely on decreasing the number of acute attacks by improving the hygiene of affected limbs, use of appropriate topical antibiotics and antifungals, exercise, elevation of the limb and use of footwear. While this treatment package has been shown to be effective in halting the progression of LE, it requires sustained access to resources required for limb care and strict adherence to the prescribed procedures.

The anti-*Wolbachia* effects of doxycycline and its potential role in filarial infections have been well documented (Taylor et al, 2005; Turner et al, 2006). Recent observations have suggested a potential role for lymphatic endothelium-derived VEGF-C and sVEGFR3 and other angiopoietic factors in the pathogenesis of LE that could be lowered by doxycycline (Debrah et al, 2006; Coulibally et al 2009). A six- week course of doxycycline 200 mg daily (DOX 200) prevented progression of LE in patients with active infection of *W. bancrofti* (Debrah et al, 2006). More recently, in a trial in Ghana, a similar course of doxycycline (DOX 200) decreased severity of mild to moderate LE independent of active filarial infection (Mand et al, 2012).

The effect of DOX 200 in reversing or stopping the progression of LE of patients with stage 1-3, irrespective of their filarial infections being active or not, leads to the assumption that the same effect could also be expected in patients with LE due to podoconiosis (PodoLE). Therefore, this trial is designed to show efficacy of doxycycline (200mg/d for 6 weeks) in patients with PodoLE stage 2-4 (Tekola et al, 2008).

2. Explanation for choice of comparators.

Current lymphedema management protocols are based on the use of simple measures of hygiene (regular washing with soap and water, skin and nail care), use of topical antibiotics or antifungal agents, exercise and footwear. Previous controlled clinical trials and extensive field experience have shown the benefit of these measures in reducing the frequency of attacks of acute dermato-lymphangio-adenitis (ADLA) that drive the progression of lymphedema (Shenoy et al, 1999). In most endemic countries they now represent the available "standard of care" in the absence of any structured treatment programs for the management of lymphedema.

In the present study, the progression of lymphedema in a group of patients who receive a six-week course of doxycycline will be compared with that of a group who receives doxycycline "look-alike"

placebo tablets. However, both groups will be enrolled into a standardized "regimen of hygiene" described above. Thus, patients enrolled in the "placebo" group also will receive the current standard of care, and the placebo used in the study will help to identify the benefits of doxycycline on a background of simple hygiene measures. The regimens will be explained to all participants who will be trained to use established standardized methods of hygiene and be effectively applying it prior to the initiation of the drug treatment. In addition, patients will be retrained at every contact point (4, 6, 12 and 18 months). A common, generic SOP with handouts that describes methods and the training schedule will be used so that similar methods are employed across all sites.

Coulibaly YI, et al. (2009) A randomized trial of doxycycline for Mansonella perstans infection. N Engl J Med 361(15):1448-58.
Davey G, Newport M: Podoconiosis: the most neglected tropical disease? . *Lancet* 2007a, 369:888-889.

Davey G, Tekola F, Newport MJ: Podoconiosis: non-infectious geochemical elephantiasis. *Trans R Soc Trop Med Hyg* 2007b 101(12):1175-1180.

Debrah, A.Y., et al. (2006) Doxycycline reduces plasma VEGF-C/sVEGFR-3 and improves pathology in lymphatic filariasis. PLoS Pathog 2(9):e92

Mand, S., et al. (2012) Doxycycline Improves Filarial Lymphedema Independent of Active Filarial Infection: A Randomized Controlled Trial. Clin Infect Dis. 55(5):621-30. doi: 10.1093/cid/cis486

Price EW, Henderson WJ: Endemic elephantiasis of the lower legs in the United Cameroon Republic. *Trop Geogr Med* 1981, 33(1):23-29.

Shenoy RK, et al. (1999) A double-blind, placebo-controlled study of the efficacy of oral penicillin, diethylcarbamazine or local treatment of the affected limb in preventing acute adenolymphangitis in lymphoedema caused by brugian filariasis. Ann Trop Med Parasitol 93:367–377.

Taylor, M.J. et al.(2005) Macrofilaricidal activity after doxycycline treatment of Wucheraria bancrofti: a double-blind, randomised placebo-controlled trial. Lancet 365(9477):2116-21

Turner, J.D., et al.(2006) A randomized, double blind clinical trial of a 3-week course of doxycycline plus albendazole and ivermectin for the treatment of Wucheraria bancrofti infection. Clin Infect Dis 42(8):1081-9

Wanji S, Tendongfor N, Esum M, Che JN, Mand S, Tanga Mbi C, Enyong P, Hoerauf A: Elephantiasis of non-filarial origin (podoconiosis) in the highlands of north-western Cameroon. *Ann Trop Med Parasitol* 2008, 102(6):529-540.

3. Details of active medical product

Doxycycline is a well-known medicine that has been in widespread use since the 1970's for a large number of infectious disease indications. At low doses it has been used for many years for the management of acne in teenagers. More recently it has become one of the main treatments for the prophylaxis of malaria. As such it is used over prolonged periods with daily dosing. It is also being

investigated as a macrofilaricide in both onchocerciasis and lymphatic filariasis. Given the extensive experience with doxycycline over many years, an examination of its preclinical characteristics is considered unnecessary, and the material presented below is based on the accepted Summary of Product Characteristics of the European Medicines Agency who are responsible for the licencing of the active doxycycline product 'Remycin' manufactured and marketed by Remedica, Limassol, Cyprus, which is being used in this study.

3.1 Name of the medicinal product

DOXYCYCLINE TABLETS BP 100mg

3.2 Qualitative and quantitative composition

Each tablet contains Doxycycline hyclate equivalent to 100mg of Doxycycline base.

For the full list of excipients, see section 6.1.

3.3 Pharmaceutical form

Plain white biconvex film-coated tablets.

3.4 Clinical particulars
3.4.1 Therapeutic indications

Doxycycline Tablets are used in the treatment of a variety of infections caused by susceptible strains of Gram-positive and Gram-negative bacteria and certain other micro-organisms.

A) Respiratory tract infections: Pneumonia and other lower tract respiratory tract infections due to susceptible strains of *Streptococcus pneumoniae, Haemophilia influenzae, Klebsiella pneumoniae* and other organisms. *Mycoplasma pneumoniae* pneumonia. Treatment of chronic bronchitis. Sinusitis.

B) Urinary tract infections: Infections caused by susceptible strains of Klebsiella species, Enterobacter species. *Escherichia coli, Streptococcus faecalis* and other organisms.

C) Sexually transmitted diseases: Infections due to *Chlamydia trachomatis* including uncomplicated urethral, endocervical or rectal infections. Non-gonococcal, urethritis caused by *Ureaplasma urealyticum*. Chancroid infections due to *alymmatobacterium granulomatis.* Alternative drug in the treatment of gonorrhoea and syphilis.

D) Dermatological infections: Acne vulgaris when antibiotic therapy is considered necessary.

3.4.2 Other clinical uses

Since doxycycline is a member of the tetracycline series of antibiotics, it may be expected to be useful in the treatment of infections which respond to other tetracyclines, such as:

A) Ophthalmic infections: Due to susceptible strains of gonococci, staphylococci and *Haemophilus influenzae*. Doxycycline Capsules are indicated in the treatment of trachoma, although the infectious agent is not always eliminated, as judged by immunofluorescence.

B) Rickettsial infections: Rocky Mountain spotted fever, typhus group, Q fever and *Coxiella endocarditis* and tick fevers.

C) Miscellaneous: Psittacosis, cholera, meliodosis, leptospirosis, other infections due to susceptible strains of Yersinia species, Brucella species (in combination with Streptomycin), Clostridium species, *Francisella tularensis* and chloroquine-resistant falciparum malaria.

D) Doxycycline Tablets are indicated for prophylaxis in the following conditions: Scrub typhus, travellers' diarrhoea (enterotoxigenic *Escherichia coli*), leptospirosis.

3.4.3 Posology and method of administration

<u>Posology</u>

Adults: The usual dose of Doxycycline Capsules for the treatment of acute infections in adults is 200mg on the first day (administered as a single dose or divided into two equal doses with a twelve hour interval), followed by a maintenance dose of 100mg/day. In the management of more severe infections (particularly chronic infections of the urinary tract), 200mg daily should be given throughout the treatment period. Exceeding the recommended dosage may result in an increased incidence of side effects.

Therapy should be continued at least 24-48 hours after symptoms and fever have subsided. When used in streptococcal infections, therapy should be continued for 10 days to prevent the development of rheumatic fever or glomerulonephritis.

Specific infections:

Acne vulgaris: 50mg daily with food or fluid for 6-12 weeks.

Sexually transmitted diseases: 100mg twice daily for 7 days is recommended in the following infections: uncomplicated gonococcal infections (except anorectal infections in men); uncomplicated urethral, endocervical or rectal infection caused by *Chlamydia trachomatis*; non-gonococcal urethritis caused by *Ureaplasma realyticum.*

Acute epididymo-orchitis caused by Chlamydia trachomatis or Neisseria gonorrhoeae 100mg twice daily for 10 days.

Primary and secondary syphilis: 300mg a day in divided doses for at least 10 days.

Louse-borne and tick-borne relapsing fevers: A single dose of 100mg or 200mg according to severity.

Chloroquine-resistant falciparum malaria: Treatment - 200mg daily for at least 7 days. Due to the potential severity of the infection, a rapid-acting schizonticide such as quinine should always be given in conjunction with doxycycline; quinine dosage recommendations vary in different areas.

Prophylaxis – 100mg daily starting at least 7 days prior to entering a malaria endemic area and continuing for 4 weeks after return.

For the prevention of scrub typhus: 200mg as a single dose.

For the prevention of travellers' diarrhoea in adults: 200mg on the first day of travel (administered as a single dose or as 100mg every 12 hours) followed by 100mg daily throughout the stay in the area. Data on the use of the drug prophylactically are not available beyond 21 days.

For the prevention of leptospirosis: 200mg once each week throughout the stay in the area and 200mg at the completion of the trip. Data on the use of the drug prophylactically are not available beyond 21 days.

3.4.4 Special Populations

Paediatric population: Not recommended for children below the age of 12 years. NOTE: in this study only individuals of the ages18-65 will be recruited and those adults below 50kg body weight are given a reduced dosage of 100mg daily.

Elderly: Doxycycline may be prescribed in the usual dose with no special precautions. No dosage adjustment is necessary.

Renal impairment: Studies to date have indicated that administration of doxycycline at the usual recommended doses does not lead to excessive accumulation of the antibiotic in patients with renal impairment.

The anti-anabolic action of the tetracyclines may cause an increase in blood urea. Studies to date indicate that this does not occur with the use of doxycycline in patients with impaired renal function.

Haemodialysis does not alter the serum half-life of doxycycline.

3.4.5 Method of administration

The tablets should be swallowed with plenty of fluid in either the resting or standing position and well before going to bed for the night to reduce the likelihood of oesophageal irritation and ulceration.

If gastric irritation occurs, it is recommended that tablets be given with food or milk. Studies indicate that the absorption of doxycycline is not notably influenced by simultaneous ingestion of food or milk.

3.5 Safety
3.5.1Contra-indications

• Hypersensitivity to the active substance, any of the tetracyclines or to any of the excipients listed in section 6.1.

• The use of drugs of the tetracycline class during tooth development (pregnancy, infancy and childhood to the age of 12 years) may cause permanent discolouration of the teeth (yellow-grey-brown). This adverse reaction is more common during long-term use of the drugs but has been observed following repeated short-term courses. Enamel hypoplasia has also been reported. Doxycycline is contra-indicated in these groups of patients.

• *Paediatric population:* Contraindicated in children under the age of 12 years. As with other tetracyclines, doxycycline forms a stable calcium complex in any bone-forming tissue. A decrease in the fibula growth rate has been observed in premature infants given oral tetracyclines in doses of 25mg/kg every 6 hours. This reaction was shown to be reversible when the drug was discontinued. (See above about use during tooth development).

• *Pregnancy:* Doxycycline is contra-indicated in pregnancy. It appears that the risks associated with the use of tetracyclines during pregnancy are predominantly due to effects on teeth and skeletal development. (See above about use during tooth development).

• *Breastfeeding:* Tetracylines are excreted into milk and are therefore contra-indicated in breastfeeding mothers. (See above about use during tooth development).

3.5.2 Special warnings and precautions for use

Photosensitivity: Photosensitivity manifested by an exaggerated sunburn reaction has been observed in some individuals taking tetracyclines, including doxycycline. Patients likely to be exposed to direct sunlight or ultraviolet light should be advised that this reaction can occur with tetracycline drugs and treatment should be discontinued at the first evidence of skin erythema.

Use in patients with impaired hepatic function: Doxycycline should be administered with caution to patients with hepatic impairment or those receiving potentially hepatotoxic drugs. Abnormal hepatic function has been reported rarely and has been caused by both the oral and parenteral administration of tetracyclines, including doxycycline.

Use in patients with renal impairment: Excretion of doxycycline by the kidney is about 40%/72 hours in individuals with normal renal function. This percentage excretion may fall to a range as low as 1-5%/72 hours in individuals with severe renal insufficiency (creatinine clearance below 10ml/min). Studies have shown no significant difference in the serum half-life of doxycycline in individuals with normal and severely impaired renal function. Haemodialysis does not alter the serum half-life of doxycycline. The anti-anabolic action of the tetracyclines may cause an increase in blood urea. Studies to date indicate that this anti-anabolic effect does not occur with the use of doxycycline in patients with impaired renal function.

Microbiological overgrowth: The use of antibiotics may occasionally result in over-growth of non-susceptible organisms, including *Candida*. If a resistant organism appears, the antibiotic should be discontinued and appropriate therapy instituted.

Pseudomembranous colitis has been reported with nearly all antibacterial agents, including doxycycline, and has ranged in severity from mild to life-threatening. It is important to consider this diagnosis in patients who present with diarrhoea subsequent to the administration of antibacterial agents.

Oesophagitis: instances of oesophagitis and oesophageal ulcerations have been reported in patients receiving capsule and tablet forms of drugs in the tetracycline class, including doxycycline. Most of these patients took medications immediately before going to bed or with inadequate amounts of fluid.

Bulging fontanelles in infants and benign intracranial hypertension in juveniles and adults have been reported in individuals receiving full therapeutic drugs. These conditions disappeared rapidly when the drug was discontinued.

Porphyria: There have been rare reports of porphyria in patients receiving tetracyclines.

Venereal disease: When treating venereal diseases, where coexistent syphilis is suspected, proper diagnostic procedures, including dark-field examinations, should be utilised. In all such cases monthly serological tests should be made for at least four months.

Beta-haemolytic streptococci infections: Infections due to Group A beta-haemolytic Streptococci should be treated for at least 10 days.

Myasthenia gravis: Due to a potential for weak neuromuscular blockade, care should be taken in administering tetracyclines to patients with myasthenia gravis.

Systemic lupus erythematous: Tetracyclines can cause exacerbation of systemic lupus erythematosus (SLE).

Methoxyflurane: Caution is advised in administering tetracyclines with methoxyflurane (see section 3.5.3).

3.5.3 Interaction with other medicinal products and other forms of interaction

There have been reports of prolonged prothrombin time in patients taking warfarin and doxycycline.

Tetracyclines depress plasma prothrombin activity and reduced doses of concomitant anticoagulants may be necessary.

Since bacteriostatic drugs may interfere with the bactericidal action of penicillin, it is advisable to avoid giving doxycycline in conjunction with penicillin.

Absorption of doxycycline may be impaired by concurrently administered antacids containing aluminium, calcium, magnesium or other drugs containing these cations; oral zinc, iron salts or bismuth preparations. Dosages should be maximally separated.

Phenobarbital, carbamazepine, primidone and phenytoin may increase the metabolism of doxycycline (reduced half-life). An increase in the daily dosage of doxycycline should be considered.

Alcohol may decrease the half-life of doxycycline.

The concurrent use of tetracyclines and methoxyflurane has been reported to result in fatal renal toxicity. See section 3.5.2.

Doxycycline may increase the plasma concentration of ciclosporin. Co-administration should only be undertaken with appropriate monitoring.

Drugs that induce hepatic enzymes such as rifampicin may accelerate the decomposition of doxycycline, thereby decreasing its half-life. Sub-therapeutic doxycycline concentrations may result. Monitoring concurrent use is advised and an increase in doxycycline dose may be required.

Laboratory test interactions

False elevations of urinary catecholamine levels may occur due to interference with the fluorescence test.

3.5.4 Fertility, pregnancy and lactation

See “Contra-indications”, section 3.5.1.

3.5.5 Effects on ability to drive and use machines

The effect of doxycycline on the ability to drive or operate heavy machinery has not been studied. There is no evidence to suggest that doxycycline may affect these abilities.

3.6 Undesirable effects

The following adverse reactions have been observed in patients receiving tetracyclines, including doxycycline.

Hypersensitivity reactions, including anaphylactic shock, anaphylaxis, anaphylactoid reactions, anaphylactoid purpura, hypotension, pericarditis, angioneurotic oedema, exacerbation of systemic lupus erythematosus, dyspnoea, serum sickness, peripheral oedema, tachycardia and urticaria.

Infections and infestations: As with all antibiotics, overgrowth of non-susceptible organisms may cause candidiasis, glossitis, staphylococcal enterocolitis, pseudomembranous colitis (with *Clostridium difficile* overgrowth) and inflammatory lesions (with candidal overgrowth) in the anogenital region.

Blood and lymphatic system disorders: Haemolytic anaemia, thrombocytopenia, neutropenia, porphyria and eosinophilia have been reported with tetracyclines.

Endocrine disorders: When given over prolonged periods, tetracyclines have been reported to produce brown-black microscopic discoloration of thyroid tissue. No abnormalities of thyroid function are known to occur.

Nervous system disorders: Headache. Bulging fontanelles in infants and benign intracranial hypertension in juveniles and adults have been reported in some individuals receiving full therapeutic dosages of tetracyclines. These are reversible on stopping the drug. Symptoms include blurring of vision, scotomata and diplopia. Permanent visual loss has been reported.

Ear and labyrinth disorders: Tinnitus.

Gastrointestinal disorders: Gastro-intestinal symptoms are usually mild and seldom necessitate discontinuation of treatment. Abdominal pain, stomatitis, anorexia, nausea, vomiting, diarrhoea, dyspepsia and rarely dysphagia. Oesophagitis and oesophageal ulceration have been reported in patients receiving doxycycline. A significant proportion of these cases has occurred with the hydrochloride salt in the capsule form. (See section 3.5.2). Tetracyclines may cause discoloration of teeth and enamel hypoplasia, but usually only after long-term use.

Hepato-biliary disorders: Transient increases in liver function tests, hepatitis, jaundice, hepatic failure and pancreatitis have been reported rarely.

Skin and subcutaneous tissue disorders: Rashes including maculopapular and erythematous rashes occur, exfoliative dermatitis, erythema multiforme, Stevens- Johnson syndrome and toxic epidermal necrolysis, photo-onycholysis. Photosensitivity (see section 3.5.2).

Musculo-skeletal, connective tissue and bone disorders: Arthralgia and myalgia.

Renal and urinary disorders: Increased blood urea. (See section 3.5.2).

Reproductive system and breast disorders: vaginitis.

3.7 Overdose

Acute over dosage with antibiotics is rare. In the event of over dosage, gastric lavage plus appropriate supportive treatment is indicated.

Dialysis does not alter serum half-life and thus would not be of benefit in treating cases of overdosage.

4. Pharmacological properties
4.1 Pharmacodynamic properties

Pharmacotherapeutic group: tetracyclines, ATC code: J01AA02

Doxycycline is primarily a bacteriostatic antibiotic.

4.2 Mechanism of action

The main mechanism of action of doxycycline is on protein synthesis. Doxycycline passes directly through the lipid bilayer of the bacterial cell wall and an energy dependent active transport system pumps the drug through the inner cytoplasmic membrane. Once inside the cell doxycycline inhibits protein synthesis by binding to 30S ribosomes and prevents the addition of amino acids to the growing peptide chain. Doxycycline will impair protein synthesis in mammalian cells at very high concentrations but these cells lack the active transport system found in bacteria.

4.3 Pharmacokinetic properties

<u>Absorption</u>

Doxycycline is almost completely absorbed and is not subject to pre-systemic metabolism, the mean bioavailability being approximately 93%.

Distribution

Tissue distribution is good and Doxycycline has a strong affinity for renal and lung tissue. Plasma protein binding is in the range 82-93% and Doxycycline is transferred into breast milk. The volume of distribution for doxycycline ranges from 0.9-1.8 lkg^{-1} and the plasma half-life ranges from 18-22 hours.

Biotransformation

No significant metabolism occurs.

Elimination

Doxycycline is cleared intact by renal and biliary mechanisms.

5 Preclinical safety data

Not applicable.

6. Pharmaceutical particulars
6.1 List of excipients

Also contains:

[Excipient list to be added]

6.2 Incompatibilities

None known.

6.3 Shelf life

PVC Blister packs: Five years.

6.4 Special precautions for storage

Store below 25°C in a dry place.

6.5 Nature and contents of container

The product is supplied in blister packs in cartons:

a) Carton: Printed carton manufactured from white folding box board.

b) Blister pack: (i) 250µm white rigid PVC. (ii) Surface printed 20µm hard temper aluminium foil with 5-7g/M² PVC and PVdC compatible heat seal lacquer on the reverse side.

6.6 Special precautions for disposal and other handling

Not applicable.

E-2 DATA SAFETY MONITORING COMMITTEE (DSMC)

30June2016

NEGLECTED TROPICAL DISEASES
SUPPORT CENTER

CHARTER FOR THE DATA SAFETY MONITORING COMMITTEE (DSMC)

Version 1.0, dated 9 June 2016

Study: –LeDoxy Phase III

Title: **A multi-center, double-blind, randomized, 24-month study, to compare the efficacy of doxycycline once daily versus placebo in improving filarial lymphedema independent of active filarial infection**

DSMC Approved:

Name: **Signature:** **Date:**

____

DSMC Chairperson

____

____

1

30June2016

Table of Contents

01 PURPOSE 3
02 RESPONSIBILITIES 3
Schedule of meetings 3
Composition of DSMC and Quorum 4
Meeting Format 4
Data to be reviewed 5
Meeting minutes 5
04 CONFIDENTIALITY 5
05 CONFLICT OF INTEREST GUIDELINES 5
06 REIMBURSEMENT 6
07 SPONSOR LIAISONS 6
08 PROCEDURES FOR COMMUNICATING RECOMMENDATIONS TO THE SPONSORS 6
09 RESPONSIBILITIES OF THE SPONSORS 7
10 RESPONSIBILITIES OF THE DESIGNATED STATISTICIAN 8
Appendix A: DSMC Members 9
Appendix B General Considerations for the DSMC 10

30June2016

01 PURPOSE

The purpose of this charter is to describe the procedures that the DSMC will follow during its review of tolerance and adverse event data in the attainment of study endpoints for multicentre study LeDOXY conducted in 5 countries (Mali, Ghana, Tanzania, India, Sri Lanka), and will also review data generated from other clinical and preclinical studies when these are relevant to the overall remit of the DSMC.

02 RESPONSIBILITIES

The overall responsibility of the DSMC is to protect the ethical and safety interests of subjects recruited into the LeDOXY study while protecting as far as possible the scientific validity of the data. The DSMC will meet at predefined times (see Section 03) to evaluate primary endpoints, review safety data to identify potential treatment harm and all cause mortality/morbidity, and to identify potential treatment benefit.

Specific responsibilities include:

1. Select from their number the Chairperson responsible for the operation of the DSMC
2. Review the DSMC Charter supplied by the Sponsors, and make any recommendations for changes to the Sponsors, agree and sign the Charter;
3. Determination of the type of information that they require for effective review of efficacy/safety results relevant to their responsibilities as members of the DSMC;
4. Recommendation of the format for the presentation of the information in (2);
5. Review data collection methods, and safety monitoring procedures as presented in the Case Record Form (CRF) and other documents supplied for their review and make recommendations for additions or adjustments
6. In the event that a member is unable to continue participation on the DSMC, the DSMC Chairman may recommend a replacement to the Sponsors. The Sponsors have final decision as to the replacement. No substitution of members, either by the Chairman or other members, is permissible for meetings.

03 CONDUCT OF DSMC MEETINGS

Schedule of meetings

The first meeting will be convened at the earliest opportunity after DSMC members have been selected and agreed to sit on the committee, and prior to the initiation of the study

3

Subsequent reviews will be held twice-yearly or more frequently, if necessary as determined by DSMC chairman.

The DSMC will convene meetings (face to face or teleconference calls) before, at regular intervals during the study and after the study. The DSMC will conduct an initial meeting before the initiation of the study (TBD). After the first meeting, the DSMC may request the Sponsors to amend the charter drafted by the Sponsors. The subsequent meetings will normally be held every six months, or on completion of specific enrolment targets as determined by the DSMC, and as necessary in the event of serious adverse events being encountered. The frequency of scheduled meetings will depend on subject enrolment, information accumulated and safety event rates. Where possible the meetings will be scheduled at a day and time convenient to the members of the DSMC. A schedule of subsequent meetings will be determined by the DSMC.

Composition of DSMC and Quorum

The composition of the DSMC (names and affiliations of committee members) is shown in Appendix A.

A quorum (3 members) of committee members is required at scheduled meetings, phone conferences, or unscheduled meetings. However, all proposals, motions, or recommendations to be made to the Sponsors that alter the course of the study (e.g. major protocol modification) must be reached by consensus of all members (i.e. each member must be able to live with the decision made and must be able to support it). All members are required to agree to any recommendation related to discontinuing the study.

The DSMC also may convene an Ad-hoc Advisory Committee should they deem it necessary for review of specific cases/safety concerns. In this case, the Sponsors must be notified prior to the next scheduled meeting and all *ad-hoc* members must sign a sponsor confidentiality agreement prior to the meeting. *Ad-hoc* members would act as consultants with the particular expertise required by the presenting situation, but would not participate in the consensus decision. *Ad-hoc* members cannot be principal/sub-investigators, nor can they knowingly administer medical care to a study subject.

No DSMC member may delegate his/her participation in case of absence. The DSMC statistician must be present for any agreement related to increased frequency of events. Sponsor employees, do **not** actively participate in the decision-making of the DSMC.

Meeting Format

Meetings will consist of open and closed portions. During the initial open portion of a meeting, the Sponsors may be invited to make brief presentations and be available for questions if

requested from the DSMC. No sponsor employee or member of the study team may attend closed portions of any meeting, unless agreed by the DSMC chair.

Data to be reviewed

The DSMC will receive the following data*:*

- Recruitment data supplied by study site [enrolment, defaulter and loss to follow up rates] (quarterly)
- Serious Adverse Events (as they occur and in summary quarterly)
- Adverse events (semi-annually)
- Laboratory safety data (semi-annually)
- Any other data identified as a result of the former analyses (as required)
- No interim analysis is planned. However should the DSMC consider that one should be conducted based on their consideration of the data obtained and reviewed by them, a plan of the data content and output will be developed in consultation with the Designated Statistician, and approved by the DSMC prior to any analysis

Meeting minutes

After each meeting, summary minutes of open and closed sessions will be prepared and distributed in a timely manner, reviewed and approved at the subsequent meeting. If no actions are necessary, a statement to this effect is relayed to the Sponsors (see Section 07 - Sponsor Liaisons).

At the end of the study a copy of the minutes will be forwarded to the Sponsors for archiving with other documentation pertaining to the study.

04 CONFIDENTIALITY

All members must have signed a Confidentiality Agreement with the Sponsors. In addition, members must treat as confidential the reports, meeting discussions, minutes, and recommendations of the DSMC.

05 CONFLICT OF INTEREST GUIDELINES

Members of the DSMC must declare the extent of any financial or other interests, including equity holdings in any pharmaceutical company that might be considered to have a potential financial benefit deriving from a successful outcome of the LeDOXY study upon appointment to the Committee and in the event of any material change(s) during their tenure.

Certain activities are not viewed as constituting conflicts of interest but must be reported annually to the Sponsors: the participation as members in educational activities supported by the Sponsors for other projects of the Sponsors; the participation as members in other research products supported by the Sponsors; occasional scientific consulting to the Sponsors on issues not related to the product in the trial.

06 REIMBURSEMENT

DSMC members will be reimbursed for reasonable expenses related to attending meetings, such as telephone costs, travel costs, accommodation, and meals. No other payment or future consideration will be provided.

07 SPONSOR LIAISONS

The following are contacts for the Sponsors:

- **Study Coordinator** (recommendations of study conduct, issues/"no issues" statements or concerns surrounding DSMC activities, notification of action items):
- **Designated Statistician** (questions on the data packages)
- **Safety Officer** (provision of information on component drugs and data from other studies)**: Dr John Horton, 24, The Paddock, Hitchin, Herts SG4 9EF UK. Phone: +44(0)1462-624081. email: hedgepigs@aol.com**

If issues arise that require further input from the Sponsors, other contacts may be made. Depending on the situation, the DSMC Chair and the Study Coordinator will decide on the appropriate individual to contact.

08 PROCEDURES FOR COMMUNICATING RECOMMENDATIONS TO THE SPONSORS

DSMC recommendations or "no issues" statements are transmitted in writing to the Study Coordinator within three working days (or less if considered urgent) of the meeting. The Study Coordinator will be responsible for ensuring that this information is communicated to the study sites. Based on its periodic review, the DSMC may, if warranted, recommend one of the following actions to the Sponsors after due considerations as outlined in Appendix B.

a. Continue the study according to the protocol and any related amendments.

b. Continue the study, but modify the study protocol. Modifications may include, but are not limited to, changes in inclusion/exclusion criteria, frequency of safety monitoring, alterations in study procedures, and follow-up period for purposes of safety as defined in the protocol.

c. Discontinue the study (with provisions for orderly discontinuation in accordance with good medical practice).

At the point when, in the opinion of the DSMC, there is evidence of a meaningful difference in terms of specific adverse event rates, incidence of serious adverse events or evidence of a significant difference in efficacy between treatment arms such that a specific recommendation related to alteration of the study would be made, the Designated Statistician supporting the DSMC will identify the appropriate treatment groups. The members of the DSMC will predefine the parameters to be examined and the timing of any examinations.

09 RESPONSIBILITIES OF THE SPONSORS

1. All personnel having direct responsibility for the conduct of the trial will remain blinded to treatment groups for all data until such time that the study is completed by protocol design or the DSMC recommends to the Sponsors the termination of a treatment arm or the entire study.

2. Make resources available to the DSMC as required to carry out its designated functions.

   a. Provide a contact for the distribution of information required by the DSMC.
   b. Ensure all relevant data is provided to the Designated Statistician to be processed in the format agreed by the DSMC. This will be developed as required by the DSMC in collaboration with the Designated Statistician

3. Notify DSMC in writing on the details of recruitment by site at 3 monthly intervals. These reports will also indicate the numbers of patients completed treatment, withdrawn, or at specified points in follow up.

4. Inform DSMC in writing of any potential safety concern(s) that were previously unreported.

5. Review the DSMC recommendations. In the event that the Sponsor disagrees with the DSMC recommendation, the Sponsors will put together their position and the Sponsor's Regulatory Affairs Department(s) will forward all their opinions to all Regulatory Authorities in the participating countries, and the Ethics Committees of the study sites.

6. Communicate DSMC recommendations in writing to the investigators and other relevant groups.

7. The Sponsors will remain responsibility for expedited regulatory reporting of Serious Adverse Events, pregnancies and device incidents/near incidents according to regulations.

30June2016

10 RESPONSIBILITIES OF THE DESIGNATED STATISTICIAN

The Designated Statistician will be responsible for the following:

1. Ensure personnel directly involved with the study remain blinded to all investigational product treatment codes until the study has completed per protocol or is terminated for cause.
2. The study statistician will designate the treatment code given to the DSMC (i.e. A, B; C, D, etc., where the order is random and rotated after each safety review).
3. The Designated Statistician will carry out all necessary processing of the data and prepare presentations of the data in the agreed format defined by the DSMC as necessary.

30June2016

Appendix A: DSMC Members

The DSMC consists of 4 individuals. The membership includes appropriate independent physicians, and a statistician. All members have experience and expertise in their field of practice and in the conduct of clinical trials. NOTE: Committee members may not participate in the study as principal or co-investigators, or as study subject care physicians nor can they knowingly administer medical care to a study subject. A Designated Statistician who is independent of the sponsors will support the DSMC.

A Safety Officer appointed by the Sponsors who is not directly involved in the conduct of the study, may be present at meetings during the open session but does not participate in the DSMC decision-making process. The Safety Officer is there to answer DSMC questions and to be a resource if the DSMC requests additional data or follow-up information. All Sponsor's employees will be excluded from DSMC deliberations/closed portions of meetings, unless otherwise agreed by the Chair.

Membership of the DSMC

**Full Name, Degree**	**Address**	**E-mail / Telephone**
**David Addiss, MD, MPH**	Director, Children Without Worms, TFGH<br>325 Swanton Way<br>Decatur, GA 30030	daddiss@taskforce.org<br>404.592.1415
**David J Diemert, MD, FRCPC**	The George Washington University, Department of Microbiology, Immunology and Tropical Medicine.<br>Director of Clinical Trials, Sabin Vaccine Institute Product Development Partnership,<br>Albert B Sabin Vaccine Institute<br>2300 Eye Street NW, Ross Hall 723-D<br>Washington, DC 20037	ddiemert@gwu.edu<br>202.994.2909
**Martin Peter Grobusch, MD, PhD, MSc, FRCP**	Professor (Chair) of Tropical Medicine and Head<br>Center of Tropical Medicine and Travel Medicine, Department of Infectious Diseases<br>Amsterdam Medical Center, University of Amsterdam, The Netherlands	m.p.grobusch@amc.uva.nl
**Sabine Klager, PhD, MSc, DLSHTM, CSci**	Operations Director, Cambridge Clinical Trials Unit<br>Cambridge University Hospitals NHS Foundation Trust – Addenbrooke's Hospital<br>Box 401, Hills Road<br>Cambridge, CB2 0QQ	sabine.klager@addenbrookes.nhs.uk<br>01223 348179 ext 58179

9

Appendix B General Considerations for the DSMC

This appendix lists some of the considerations to be taken into account by the DSMC. These issues include both the magnitude of the observed differences and their consistency as well as the importance of the differences to the health and the safety of the subjects in the study. It is important for these issues to be stated in advance to assure both the subjects and the investigators, who will be masked to the aggregate data, that the DSMC will carefully consider the issues of safety and recommend protocol changes if questions of safety arise.

Serious adverse events and withdrawals will be monitored by the DSMC Chair as they occur and additional safety measures may be requested by the DSMC. If important adverse events occur between planned meetings, and a substantial trend emerges, an emergency meeting of the DSMC will be called. It is important to recognise that the DSMC can review all the relevant data available and may request additional analyses before they make any suggestions that the study be modified.

Interpretation of safety data is very complex and requires both clinical and statistical experts reviewing the data in concert. A number of considerations for interpretation of these data can be stated and these include:

a. Whether the results could be explained by possible differences in the baseline variables between the groups;
b. Whether the ascertainment of outcomes could be biased because of the differences in treatment programs (ascertainment bias);
c. Whether the results are consistent for other variables which should be associated with the primary outcome variable in question;
d. Whether the results are consistent among various subgroups of subjects and across the various centres involved in the study;
e. Whether the risk which is under consideration is outweighed by assessment of the overall benefits of therapy;
f. Whether the results could be due to concomitant therapy and not due to the differences in the treatment groups;
g. Whether it is likely that the current trends could be reversed if the trial were to be continued unmodified;
h. Whether and how much additional precision could be obtained by continuing the trial under the present protocol; and,

30June2016

i. Whether there would be significant loss in the overall assessment of the validity of the trial by the medical community by discontinuation or change in the protocol.

All of these considerations require expert evaluation and are the major role of the DSMC. The DSMC will consider these issues on a regular basis to assure the safety of the subjects and to assure the investigators and the medical community that the risks of this study are being evaluated and the subject's safety is being kept foremost in mind.

At the point when, in the opinion of the DSMC, there is evidence of a meaningful difference between treatment arms such that a specific recommendation related to alteration of the study would be made, the Designated Statistician supporting the DSMC will identify the appropriate treatment groups. The parameters to be examined and the timing of any examination will predefined by the members of the DSMC

11

C-3 INSURANCE

C-3.1 Insurance for participants

COMPAGNIE PROFESSIONNELLE D'ASSURANCE DU CAMEROUN
S.A. au capital de 1 500 000 000 de Francs CFA entièrement libérés
Entreprise régie par le Code des Assurances - Arrêté d'agrément N° 00167/BIS/MINEFI/DCE/A du 03/09/1997
Siège Social et Direction Générale : 15, Rue Castelnau Akwa
R.C. N° 017867 - N° Contribuable : M O 99700005601 B
B.P. 54 Douala - Tél. : (237) 233 43 43 81 / 233 43 43 82 / 699 68 92 94 - Fax : (237) 233 43 43 84
E-mail : cpasiege@yahoo.fr - www.cpa-cameroun.com

POLICE
D'ASSURANCE
INDIVIDUELLE ACCIDENTS
GROUPE

N° 100311217DTR/2017

SOUSCRIPTEUR: UNIVERSITY OF BUEA
TAKEOFF RESEARCH PROJECT
Clinical trial on Doxycyline treatment of lymphodema due to podoconiosis

DUREE : DOUZE(12) Mois
EFFET : 1er JANVIER 2018
EXPIRATION : 31 DECEMBRE 2018

Accidents - Maladies - Corps de véhicule - Marchandises transportées - Incendie - Autres dommages aux biens
Responsabilité civile - Crédit - Caution - Pertes pécuniaires - Protection juridique - Assistance

ENGAGEMENT PROFESSIONNEL GARANTI

Entre

UNIVERSITY OF BUEA (TAKEOFF RESEARCH PROJECT)
B P 63 BUEA – CAMEROUN

Dénommée ci-après, le SOUSCRIPTEUR et/ou ASSURE

Et

COMPAGNIE PROFESSIONNELLE D'ASSURANCE DU CAMEROUN (CPA)

Siège Social & Direction Générale
15, Rue Castelnau – Akwa
B.P. 54 Douala – CAMEROUN
Tél : 33 43 43 81 / 33 43 43 82 / 33 42 81 17
Fax : 33 43 43 84

Dénommé ci-après, l'ASSUREUR

Il a été convenu ce qui suit :

Le contrat sera régi par les Dispositions du Code des Etats membre de la Conférence Interafricaine des Marchés d'Assurance (CIMA) et ses Décrets subséquents.

CONDITIONS PARTICULIERES

Souscripteur : UNIVERSITY OF BUEA (TAKEOFF RESEARCH PROJECT)

Assurés : voir liste

INTERMEDIAIRE : BD SECURITE
Police N° : 100311217DTD/2017
Code Branche : 112
Risque : INDIVIDUELLE ACCIDENTS

Durée : DOUZE (12) MOIS
Effet : 1er JANVIER 2018
Echéance : 31 DECEMBRE 2018

DECOMPTE DE LA PRIME

PRIME NETTE	ACCESSOIRES	SOUS TOTAL	TVA	CP	PRIME TTC
4.776.834	50.000	4.826.834	929.166	244.000	6.000.000

La présente police est constituée par :

- Les Conditions Générales
- Les Conditions Particulières
-

D'autre part, il est convenu que les Conditions Particulières annulent et remplacent toutes les dispositions des Conditions Générales qui seraient plus restrictives pour l'assuré ou qui présenteraient, par rapport à celle-ci, une divergence ou une incompatibilité.

Fait en deux (02) exemplaires à Douala, le 27 novembre 2017.-

LE SOUSCRIPTEUR

Samuel Wanji
PROFESSOR OF PUBLIC HEALTH
PRINCIPAL INVESTIGATOR

UNIVERSITY OF BUEA – TAKeOFF RESEARCH PROJECT

POUR LA COMPAGNIE

2

Aux conditions générales et particulières qui suivent, la COMPAGNIE PROFESSIONNELLE D'ASSURANCE DU CAMEROUN (CPA) S.A. couvre les personnes dont les noms figurent sur la liste en annexe, aux conditions de garanties suivantes.

ARTICLE 1 – OBJET DU CONTRAT

Le présent contrat a pour objet d'indemniser les assurés et /ou leurs ayants droits dans la limite des capitaux ci-dessous, en cas de décès, d'incapacité permanente totale ou partielle consécutive à un accident dont pourraient être victime les assurés en tant que personnel de l'Université de BUEA intervenant dans le projet dénommé « Take-off research project »

ARTICLE 2 – PLAFOND ANNUEL DES GARANTIES
La garantie est acquise aux assurés à concurrence (par personne dénommée)

GARANTIES	CAPITAUX ASSURES ET PRIMES PAR ASURE (en F CFA)
- Décès accidentel	2.000.000
- Invalidité Permanente	2.000 000
- Frais de traitement	150.000
-Frais d'obsèques	300.000
**PRIME ANNUELLE TTC PAR ASSURE (en F CFA)**	**30.000**

Effectif : 200 personnes dont la liste est attendue

ARTICLE 3 - **EXCLUSIONS**
Outres les exclusions figurant à l'article 6 des conditions générales, sont formellement exclus de la garantie, les accidents intentionnellement causés ou provoqués par l'assuré ou les bénéficiaires de la police, ceux provenant de folie, d'engins de guerre non éclatés, d'insurrection ou d'émeute, ainsi que ceux provenant de maladie que quelque nature qu'elle soit .
La garantie ne s'applique ni à la mort ni à l'infirmité résultant de congestion, d'empoisonnement, d'érysipèle, de refroidissement, d'ulcère variqueux, de congélation ou tout autre effet de température ou de dépression atmosphérique, ruptures musculaires, efforts, tours de reins.

Ne sont pas garanties non plus les accidents résultant d'opérations chirurgicales n'ayant pas pour cause un accident garanti par le présent contrat, chasse à courre ou aux bêtes féroces.

3

Dans tous les cas, les accidents survenus au cours de matchs ou paris sont formellement exclus de l'assurance.

ARTICLE 4 – PRIME

Le souscripteur s'engage à payer à la signature du présent contrat, la somme de (6.000.000) FCFA toutes taxes comprises suivant le décompte ci-après :

PRIME NETTE ANNUELLE		4.776.834 F.CFA
Accessoires		50.000
T.V.A	:	929.166
Cout de police	:	244.000
**PRIME TTC**	**:**	**6.000.000 FCFA**

ARTICLE 5– FONCTIONNEMENT DU CONTRAT

Toute incorporation ou retrait d'un assuré doit être signalé à l'assureur par lettre recondamnée avec accusé de réception ou contre signée. Un avenant établi à cet effet par l'assureur, constatera le montant de la prime à payer par le souscripteur ou à ristourner par l'assureur au souscripteur.

ARTICLE 6 – **DUREE DU CONTRAT**

Le présent contrat est souscrit pour une durée ferme de **DOUZE MOIS** à compter du **1er janvier 2018.**

Fait en trois exemplaires, à Douala le 27 novembre 2017.

LE SOUSCRIPTEUR | **POUR LA COMPAGNIE**

Samuel Wanji
PROFESSOR OF PUBLIC HEALTH
PRINCIPAL INVESTIGATOR

4

ASSURANCE CONTRE LES ACCIDENTS CORPORELS

CONDITIONS GENERALES

Contrat régi par le code des Assurances CIMA, les conditions générales et les conditions particulières jointes

Article Premier DEFINITIONS
Pour l'application de l'ensemble des dispositions du présent contrat, on entend par :

Accidents corporels : toute atteinte corporelle non intentionnelle, de la part de l'assuré, provenant de l'action soudaine d'une cause extérieure.

Assuré : c'est la personne sur la tête de laquelle repose l'assurance.

Bénéficiaire : toute personne désignée aux conditions particulières au profit de laquelle a été souscrit le capital payable en cas de décès accidentel de l'assuré ou à défaut, les ayants droit de ce dernier.

Souscripteur : personne physique ou morale qui, dans un contrat, signe la police d'assurance et paie la prime y afférente.

Maladie : Toute altération de la santé constatée par une autorité médicale compétente.

La loi : le code des assurances de la CIMA.

Prestations : les indemnités prévues aux conditions particulières.

Sinistre : c'est la réalisation d'un événement garanti au contrat.

Article 2 : RISQUES GARANTIS.

Le présent contrat a pour objet de garantir à l'assuré le paiement des indemnités stipulées aux conditions particulières ou par avenant, à l'occasion des accidents corporels dont il pourrait être victime dans l'exercice de sa profession ou au cours de sa vie privée.

Sont compris dans l'assurance :

1°/ Les accidents survenus à la suite d'attentat, d'agression, d'asphyxie accidentelle, par dégagement de gaz ou vapeurs ou par immersion dans l'eau, de tentative de sauvetage de personnes ou de biens ;

2°/ Les accidents causés par la foudre, l'incendie, les tremblements de terre ou la violence des éléments atmosphériques ;

3°/ Les inoculations infectieuses dues à des piqûres anatomiques ou septiques, les cas de rage ou de charbon consécutifs à des morsures d'animaux ou piqûres d'insectes ;

4°/ Les accidents éprouvés en temps de paix, pendant l'accomplissement de période d'instruction militaire n'excédant pas trente jours, y compris les accidents d'aviation survenus au cours desdites périodes aux militaires de réserve, autres que les parachutistes et les militaires appartenant au personnel naviguant de l'armée de l'Air et l'Aéronautique maritime.

Article 3 RISQUES DE CIRCULATION - SPORTS

1°/ Sont garantis sans surprime, les accidents résultant :
a) de l'usage de bicyclette avec ou sans moteur ; de l'usage, avec ou sans conduite, de véhicules automobiles ou hippomobiles ;

b) de la pratique, à titre d'amateur, des sports ci-après : gymnastique, athlétisme, escrime, chasse (sauf chasse à courre), natation, pêche, canotage, équitation, golf, tennis, jeux de boules, excursions en montagne sur routes et sentiers ;

c) Les accidents survenus à l'assuré en tant que simple passager d'un avion de sociétés de transports aériens agréées pour les transports publics de personnes et ce, dans les limites territoriales de la garantie prévues à l'article 3 ci-dessous.

2°/ Ne sont garantis que moyennant stipulation expresse aux conditions particulières et paiement d'une surprime, et sans qu'il soit dérogé à l'exclusion formulée plus loin pour les compétitions, courses, matches, concours ou paris :

a) Les accidents résultant de l'usage, même à titre de passager, de cycle à moteur d'une cylindrée supérieure à 50 cm3, de motocyclette, side-car ou tricar ;
b) Les accidents provenant de l'exercice des sports non désignés ci-dessus et notamment : football « association » ou « rugby », patinage, hockey, polo, chasse à courre, yachting en mer, ascensions de montagnes ou glaciers au-dessus de 2.000 mètres, ski, luge, bobsleigh, skelton, base-ball, yachting à moteur, motonautisme, ski nautique, vol à voile, etc.

Ne peut être comprise dans l'assurance la pratique des sports exceptionnellement dangereux désignés ci-après: boxe, catch, judo, pancrace, chasse aux bêtes féroces.

Conditions Générales Individuelle Accidents - C. P. A
Mise à jour 1er octobre 2011

Article 4 LIMITES TERRITORIALES DE LA GARANTIES

L'assurance produit effet dans le monde entier.

Article 5 VOYAGES

L'assurance garantit également les risques afférents à l'usage de moyens de transports aériens et maritimes dans le monde entier, mais ce exclusivement :

- en ce qui concerne les risques aériens : pour les accidents définis à l'article 2, alinéa c) ci-dessus ;

- en ce qui concerne les risques maritimes : pour les accidents survenus à l'assuré en tant que passager à bord de bateaux de lignes régulières et, d'une façon générale, de tous bateaux autorisés à effectuer des transports de passagers à titre payant.

Article 6 EXCLUSIONS

Sont exclus de la garantie du contrat :
1°/ Les maladies de quelque nature qu'elles soient, professionnelles ou autres, les lésions causées par les rayons X, le radium et ses composés.

2°/ Les accidents occasionnés par une infirmité, ou par une maladie grave permanente de l'assuré, par leurs suites ou complications ;

3°/ L'insolation, la congélation sauf si elles sont la conséquence directe d'un accident garanti, l'anévrisme, la congestion ; les cas d'empoisonnement, d'érésipèle, de rhumatismes, ulcère variqueux, de lumbago, de rupture de muscles, d'effort, de tour de reins, de hernie, lors même que ces affections seraient d'origine traumatique

4°/ Les accidents causés par l'ivresse, les conséquences d'opérations chirurgicales subies par l'assuré et non nécessitées par un accident garanti par le présent contrat.

5°/ Les accidents occasionnés directement ou indirectement :

- soit par la guerre étrangère ; il appartient à l'assuré de prouver que le sinistre résulte d'un fait autre que le fait de guerre étrangère ;

- soit par la guerre civile, l'insurrection, l'émeute ou le mouvement populaire ; il appartient à la Société de prouver que l'accident résulte de l'un de ces événements ;

- soit par la désintégration du noyau atomique ;
6°/ Les accidents résultant de l'emploi d'un mode de locomotion aérienne dans d'autres conditions que celles définies ci-dessus (article 2,1° c) ;

7°/ Les suicides ou tentative de suicide avant deux ans. En outre, si l'assuré perd la vie par le fait intentionnel d'un bénéficiaire, ce dernier est déchu de tous droits sur le capital assuré qui restera néanmoins payable aux autres bénéficiaires ou ayants droit ;

8°/ Les accidents survenus en cas de participation en tant que concurrent à des compétitions, courses, matches, paris et concours, mutilations volontaires de même qu'à la suite de rixe sauf le cas de légitime défense.

INDEMNITES ASSUREES

Article 6 DECES

En cas de décès survenu immédiatement des suites directes d'un accident garanti, le capital est payé aux bénéficiaires désignés. En cas de prédécès des bénéficiaires ou si l'assuré n'en a désigné aucun, le capital est payable aux ayants droit de l'assuré.

S'il y a plusieurs bénéficiaires, tout payement à effectuer à la suite du décès de l'assuré est indivisible à l'égard de la société qui réglera sur quittance collective des intéressés.

Le paiement du capital est effectué dans les quinze jours qui suivent la remise des pièces ci-après :

- certificat médical constatant le décès accidentel et acte de décès de l'assuré - certificat de vie du ou des bénéficiaires et pièces justificatives de leurs titres et qualités d'ayants droit.

Article 8 INFIRMITE PERMANENTE

Aucune indemnité ne peut être exigée par l'assuré avant que l'infirmité ait été reconnue définitive, c'est-à-dire avant guérison ou consolidation complète ; elle sera payée dans le délai d'un mois à compter de ce jour.

Toutefois, si la consolidation n'était pas acquise dans l'année suivant l'accident, la Société verserait à l'assuré, sur sa demande une provision égale à la moitié de l'indemnité minima qui est susceptible de lui être due au jour de la consolidation. Si cette provision se révélait lors de la consolidation définitive, supérieure à l'indemnité effectivement due par la Société, l'assuré devrait restituer le trop perçu.

Le degré d'infirmité permanente sera déterminé sur les bases du barème ci-annexé.

Article 9 INCAPACITE TEMPORAIRE

En cas d'incapacité temporaire, la Société garantit à l'assuré une allocation quotidienne pendant le temps où il ne peut plus se livrer à ses occupations, suit un traitement médical et se soumet au repos nécessaire à sa guérison. Elle est due à partir du premier jour du traitement médical, le jour même de l'accident ne comptant pas.
Si l'assuré exerce une profession, cette allocation est payée en totalité pendant le nombre de jour où il a été complètement empêché - du fait de l'accident - de se livrer à un travail quelconque, fût-ce même de direction ou de surveillance. Elle sera réduite de moitié dès que l'assuré pourra vaquer partiellement à son travail ou aura recouvré en partie la faculté de surveiller ou de diriger les travaux de sa profession.

Si l'assuré n'exerce aucune profession, la location, est payée en totalité pendant tout le temps où il a été obligé de garder la chambre.

L'indemnité quotidienne ne peut être due que pendant une durée maxima de 300 jours à compter de celui de l'accident ; elle sera versée dans le délai d'un mois après guérison de l'assuré.

Article 10 FRAIS MEDICAUX ET PHARMACEUTIQUES

Le remboursement des frais médicaux, chirurgicaux, hospitalisations et pharmaceutiques peut être assuré moyennant stipulation expresse aux conditions particulières et paiement de la prime correspondante.

Lorsque ce risque est garanti la Société n'aura à intervenir que dans la mesure où le remboursement de ces frais n'incombe pas par ailleurs aux assurances sociales ou à un tiers, au titre de la législation sur les accidents du travail ou des règles de droit commun.

Article 11 AGGRAVATION INDEPENDANTE DU FAIT ACCIDENTEL

Toute les fois que les conséquences d'un accident seront aggravées par l'état constitutionnel de la victime, par l'action d'une maladie ou d'une infirmité - autre que celles prévues à l'article 12 et excluant tout droit à l'indemnité - par un manque de soins constatés ou un traitement empirique, l'indemnité sera calculée, non pas sur les suites effectives du cas, mais sur celles qu'il aurait eues chez un sujet de santé normale soumis à un traitement médical rationnel.

Article. 12 - CUMUL DES INDEMNITES

L'allocation quotidienne se cumule avec celle des indemnités pouvant être dues pour le décès ou l'infirmité. Par contre, un même accident ne peut donner droit qu'à l'une ou l'autre des indemnités prévues pour les cas de décès ou d'infirmité. Par contre, un même accident ne peut donner droit qu'à l'une ou l'autre des indemnités prévues pour les cas de décès ou d'infirmité.

En aucun autre cas, un sinistre déjà réglé sur les bases du présent contrat, et pour lequel une quittance régulière aura été donnée à la société, ne peut donner lieu à révision.

Article. 13 ADMISSION A L'ASSURANCE.

Ne peuvent être assurées ou demeurer assurées les personnes infirmes, celles qui ont été atteintes d'une attaque quelconque de paralysie, d'apoplexie ou d'épilepsie, d'affection mentale ou de maladie de moelle épinière ou du cerveau ; pour les contrats en cours, la garantie n'est acquise à l'assuré que si, au jour du sinistre, il n'est pas atteint d'infirmité ou frappé d'une des maladies mentionnées ci-dessus.

En conséquence, l'assuré ou le contractant devra déclarer tant à la souscription qu'en cours de contrat s'il est atteint d'une de ces maladies ou d'une infirmité. Il lui sera remboursé la fraction des primes non absorbée du jours de sa déclaration à la prochaine échéance.

Les personnes âgées de 65 ans ne peuvent être admises à l'assurance. Si l'assuré atteint l'âge de 65 ans en cours de contrat, la société aura la faculté, à compter de ce moment, de résilier le contrat quelle que soit sa durée, pour l'expiration de chaque année d'assurance, en prévenant l'assuré, par lettre recommandée, un mois à l'avance.

Article 14 BASES DE L'ASSURANCE

L'assurance est faite sur la base des réponses aux questions posées par l'assureur notamment sur le formulaire de déclaration de risque de l'assuré qui s'engage à en laisser vérifier l'exactitude à toute époque pendant la durée du contrat par les délégués de la société.

Article 15 MODIFICATION DU RISQUE

Toutes modifications du risque assuré doivent être déclarées par lettre recommandée, conformément à l'article 15 du code CIMA.

Il en est ainsi notamment des modifications susceptibles d'aggraver le risque par exemple : si l'assuré vient à exercer sa profession dans des conditions autres que celles déclarées, s'il change de profession ou d'occupation ou s'il change de résidence.

Au reçu de la déclaration, la société aura la faculté, soit de résilier le contrat, soit de proposer un nouveau taux de prime ou de réduire proportionnellement les indemnités assurées.

Si cette déclaration préalable a été faite et si la modification eût entraîné une augmentation de prime, l'indemnité - en cas de sinistre - sera réduite dans le rapport existant entre la prime payée et celle qui aurait due être perçue. Par contre, si la prime a été fixée en raison des circonstances spéciales mentionnées dans le contrat et ayant constitué une aggravation du risque, l'assuré ou le contractant aura le droit, les circonstances venant à disparaître, de résilier le contrat sans indemnité, à moins que la société ne consente la diminution de prime correspondante à compter de la prochaine échéance, d'après le tarif applicable lors de la souscription du contrat.

Article 16 SANCTIONS

Toute réticence ou déclaration intentionnellement fausse, toute omission ou déclaration inexacte de la part de l'assuré ou du contractant, entraîne l'application des sanctions prévues ***aux articles*** **18 et 19** du code CIMA : **(Article 18) Fausse déclaration intentionnelle : indépendamment des causes ordinaires de nullité, et sous réserve des dispositions de l'article 80, le contrat d'assurance est nul en cas de réticence ou de fausse déclaration intentionnelle de la part de l'assuré, quand cette réticence ou cette fausse déclaration change l'objet du risque ou en diminue l'opinion pour l'assureur, alors même que le risque omis ou dénaturé par l'assuré a été sans influence sur le sinistre. Les primes payées demeurent alors acquises à l'assureur, qui a droit au paiement de toutes les primes échues à titre de dommages et intérêts. (Article 19) Fausse déclaration non intentionnelle : L'omission ou la déclaration inexacte de la part de l'assuré dont la mauvaise foi n'est pas établie n'entraîne pas la nullité de l'assurance. Si elle est constatée avant tout sinistre, l'assureur a le droit de maintenir le contrat, moyennant une augmentation de prime acceptée par l'assuré, soit de résilier le contrat dix jours après notification adressée à l'assuré par lettre recommandée ou contresignée, en restituant la portion de la prime payée pour le temps où l'assurance cours. Dans le cas où la**

constatation n'a lieu qu'après un sinistre, l'indemnité est réduite en proportion du taux des primes payées par rapport au taux des primes qui auraient été dues, si les risques avaient été complètement et exactement déclarés.

Article 17 ASSURANCES MULTIPLES

L'assuré ou le contractant est tenu de déclarer les assurances de même nature qu'il a contractées ou qu'il contractera à d'autres sociétés. Dans ce cas, la société pourra résilier le contrat.

En cas de sinistre, et à défaut de cette déclaration préalable, il est convenu que :

- si l'omission est intentionnelle de la part de l'assuré ou du contractant, il sera fait application des sanctions prévues par l'article 18 du Code CIMA.

- si l'omission n'est pas intentionnelle, le règlement sera soumis à un Comité d'arbitrage qui aura à apprécier si l'assureur aurait contracté dans le cas où il aurait eu la connaissance de l'autre assurance et à fixer éventuellement dans quelle proportion l'indemnité due sera réduite.

Ce Comité d'arbitrage sera composé comme suit :

- un arbitre désigné par l'assureur ;
- un arbitre désigné par l'assuré ou ses ayants droit ;
- et en cas de désaccord des deux arbitres, un tiers arbitre désigné à raison de ses compétences juridiques ou techniques par les deux précédents ou en cas de désaccord entre eux, par le Président du Tribunal compétent à la requête de la partie la plus diligente.

Ces arbitres seront dispensés de toutes formalités judiciaires ; ils pourront s'abstenir de suivre les règles de droit commun.

Chaque partie paiera les honoraires de son arbitre et ceux du troisième par moitié.

Article 18 PAIEMENT DES PRIMES

La prime et les accessoires de prime dont le montant est stipulé au contrat, ainsi que les impôts et taxes sur les contrats d'assurance dont la récupération n'est pas interdite, sont payables d'avance au domicile de l'assureur ou de l'intermédiaire, dans les conditions prévues à l'article 541 du Code des Assurances.

A défaut de paiement d'une prime ou d'une fraction de prime dans le délai convenu, le contrat est résilié de plein droit, la portion de prime courue reste acquise à l'assureur, sans préjudice des éventuels frais de poursuite de recouvrement.

Article. 19 OBLIGATIONS EN CAS D'ACCIDENTS.

a). L'Assuré ou ses ayants droit, le contractant s'il y a lieu, ou toute personne agissant au nom de l'un de ceux-ci, sont tenus, sous peine de déchéance, de faire la déclaration de tout accident, dans les cinq jours où il est parvenu à leur connaissance, sauf cas fortuit ou de force majeure.

Cette déclaration doit être faite par écrit, ou verbalement contre récépissé, à la société ou à l'Agence indiquée sur le contrat.

Le déclarant précisera les noms, prénoms, âge, profession et domicile du blessé, les dates, causes et circonstances de l'accident ainsi que les noms et les adresses des témoins s'il y en.a.

b) Les personnes désignées au premier alinéa devront avoir immédiatement recours à des soins médicaux et généralement faire le possible pour le rétablissement du blessé. Elles devront également transmettre à leurs frais, dans le délai de 10 jours à compter de l'accident, un certificat du médecin appelé à donner les premiers soins, relatant la nature de l'acte, des blessures, l'état actuel du blessé et les conséquences probables de l'accident.
S'il n'est pas en état de reprendre ses occupations à la date fixée par le médecin, le blessé devra, dans les 5 jours de cette date, transmettre un nouveau certificat médical, sous peine d'être considéré comme guéri à l'expiration du délai fixé par le précédent certificat.

En cas de simple retard apporté dans la transmission de ces pièces médicales, la Société aura la faculté de réclamer une indemnité proportionnée au dommage que ce retard lui aura causé.

Les médecins de la Société devront avoir libre accès auprès de l'assuré dans tous les cas et à toute époque, sous peine pour celui-ci de se trouver déchu de tout droit à indemnité.
En outre, au cas où un assuré refuserait de se soumettre au contrôle des Délégués de la Société, et si après avis de la Société donné quarante-huit heures à l'avance par lettre recommandée, il persistait dans son refus, il serait déchu de tout droit à l'indemnité. Il en serait de même au cas où malgré le préavis indiqué ci-dessus, la Société se verrait, du fait de l'assuré, dans l'impossibilité d'exercer son contrôle.

Il est expressément convenu que si l'assuré emploie sciemment des documents ou moyens mensongers, il sera entièrement déchu de tout droit à une indemnité ; il en sera de même en cas de réticence dans la déclaration d'un sinistre, tendant à exagérer ou à dénaturer les suites de cet accident, à en déguiser les causes ou à en prolonger les conséquences.

Article 20 ESTIMATION DES INDEMNITES

Les causes du décès, de l'incapacité permanente ou temporaire, le taux de l'infirmité, la durée de l'incapacité temporaire, les conséquences de l'accident telles qu'elles sont prévues aux articles 6, 7, 8 et 9 ci-dessus sont constatées, soit d'un commun accord entre la Société et l'assuré ou, en cas de décès, les bénéficiaires, soit à défaut d'accord, par deux médecins désignés, chacun par l'une des parties. S'il y a divergence entre eux, ces deux médecins s'en adjoindront un troisième pour les départager.

Faute par l'assuré ou les bénéficiaires de désigner leur médecin dans les quarante-huit heures à dater du jour où ils y auront été invités, par lettre recommandée, ou par deux médecins déjà désignés de s'entendre sur le choix du troisième, le médecin manquant sera nommé d'office par voie de référé du Président du Tribunal compétent à la requête de la Société dans le premier cas, de la partie la

plus diligente dans le second, l'autre partie ayant été convoquée par lettre recommandée.

Dans tous les cas, les médecins opéreront en commun à la majorité des voix et seront dispensés du serment ainsi que de toutes formalités judiciaires.

Chaque partie paiera les honoraires du médecin désigné par elle : ceux du troisième seront partagés par moitié entre la Société et l'assuré ou les bénéficiaires.

Tant que cette expertise médicale amiable, que chaque partie a la faculté de provoquer n'aura pas eu lieu, les parties s'interdisent d'avoir recours à la voie judiciaire pour le règlement de l'indemnité en litige.

Article 21 RECOURS CONTRE LE TIERS RESPONSABLE

L'assuré ou ses ayants droit conservent tout recours contre le tiers responsable de l'accident, sans que la Société soit subrogée dans leurs droits et actions après paiement de la somme assurée.

Article 22 PRISE D'EFFET DU CONTRAT

La prise d'effet du présent contrat est subordonnée au paiement intégral de la prime.

Les mêmes dispositions s'appliquent à tout avenant au contrat.

Article 23 DUREE DU CONTRAT

Le présent contrat est conclu pour la durée prévue aux Conditions Particulières.

Lorsque le contrat prévoit une clause de tacite reconduction, il est, à l'expiration de la période d'assurance en cours, reconduit automatiquement d'année en année, sauf dénonciation par l'une ou autre des parties. La dénonciation doit intervenir au moins deux mois avant cette expiration, dans les formes prévues à l'article 22 du Code des Assurances.
A chaque échéance, l'assureur est tenu d'aviser à la dernière adresse connue, au moins 45 jours à l'avance, l'assuré ou la personne chargée du paiement de la prime, de la date d'échéance et du montant dont il est redevable.

Cet avis, matérialisé par une lettre avec accusé de réception ou décharge devra rappeler que le contrat sera résilié de plein droit si la prime de renouvellement n'est pas payée dans le délai prévu à l'article 13 du Code des Assurances.

Article 24 RESILIATION APRES SINISTRE

Le contrat peut être résilié avant sa date d'expiration normale dans les cas et conditions fixées ci-après :

1°/ Par le souscripteur ou l'assureur :

a) A chaque échéance annuelle du contrat, moyennant deux mois au moins de préavis si le contrat est à tacite reconduction. (article 21 du Code des Assurances)

b) En cas de changement de domicile, de profession, en cas de retraite professionnelle ou de cessation définitive d'activités professionnelles, ou en cas de changement de situation ou de régime matrimonial, à la condition que ces évènements soient liés au risque (article 25 du Code des Assurances).

2°/ Par l'assureur

a) En cas d'aggravation du risque (article 15 du Code des Assurances)

b) En cas d'omission ou d'inexactitude de bonne foi dans la déclaration du risque à la souscription ou en cours de contrat (article 19 du Code des Assurances).

c) Après sinistre, l'assureur se réserve, dans ce cas, le droit de résilier le contrat, dans un délai de trois mois, après qu'il ait eu connaissance du sinistre. La résiliation après sinistre ne prendra effet qu'après l'expiration d'un préavis d'un mois à dater de la notification à l'assuré par lettre recommandée ou acte extrajudiciaire ou par tout autre moyen.

   Passé ce délai d'un mois après qu'il aura eu connaissance du sinistre, l'assureur ne pourra se prévaloir de celui-ci pour résilier le contrat, s'il a déjà accepté le paiement d'une prime ou d'une fraction de prime correspondant à une période postérieure audit sinistre.

   S'il est fait usage de la faculté prévue à l'alinéa ci-dessus, le souscripteur aura le droit, dans le délai d'un mois à compter de la notification de la résiliation du présent contrat, de résilier les autres contrats qu'il peut avoir souscrit chez l'assureur ; cette résiliation par le souscripteur prendrait effet un mois après la notification à l'assureur.

   L'usage de la faculté prévue aux deux alinéas précédents entraîne restitution par l'assureur des portions de primes afférentes à la période pour laquelle les risques ne sont plus garantis.

d) En cas de faillite ou de liquidation judiciaire de l'assuré (article 17, alinéa 1 du Code des Assurances).

3°/ Par le souscripteur

a) En cas disparition des circonstances aggravantes mentionnées dans la police et si l'assureur ne consent pas la diminution de prime correspondante (article 15 du Code des Assurances) ;

b) En cas de résiliation par l'assureur du contrat après sinistre, le souscripteur a le droit, dans un délai d'un mois à dater de la notification de la résiliation .par l'assureur, de résilier les autres contrats détenus auprès dudit assureur, avec effet, un mois après la notification à ce dernier ;

c) En cas de retraite professionnelle ou de cessation définitive d'activité professionnelle.

4°/ Par la masse des créanciers du souscripteur

En cas de faillite ou de liquidation judiciaire de celui-ci (article 17, alinéa 1du Code des Assurances).

5°/ De plein droit

a) En cas de perte totale de la chose assurée (article 39 du Code des Assurances).

b) En cas de retrait de l'agrément de l'assureur (article 17, alinéa 2 du Code des Assurances) ;

c) En cas de réquisition de propriété, d'usage ou de service, conformément à la législation en vigueur.

d) En cas de non paiement de la prime dans les conditions et formes prévues à l'article 13 du Code des Assurances.

e) En cas d'émission de chèque ou d'effet impayé, lorsque la régularisation n'a pas été effectuée dans les délais prévus à l'article 13-1 du Code des Assurances.

Dans tous les cas de résiliation hormis ceux prévus au point d) et e) ci-dessus, l'assureur doit au souscripteur la fraction de prime afférente à la période pendant laquelle le risque n'est plus garanti.

Lorsque le souscripteur ou l'héritier a la faculté de demander la résiliation, il peut le faire à son choix, soit par une déclaration faite contre récépissé au siège social ou chez le représentant de l'assureur dans la localité, soit par acte extrajudiciaire, soit par lettre recommandée. La résiliation par l'assureur doit être notifiée par lettre recommandée adressée au dernier domicile connu du souscripteur.

La Société a la faculté de résilier le contrat après chaque sinistre, la résiliation ne prenant effet qu'un mois après notification à l'assuré ou au Contractant, conformément aux prescriptions de l'article 23 du code CIMA

Lorsque la Société use de cette faculté, l'assuré a le droit, dans le délai d'un mois à compter du jour où la résiliation du contrat sinistré lui est notifiée, de résilier les autres contrats qu'il peut avoir souscrits, la résiliation prenant effet un mois après notification à la Société.

La faculté de résiliation réservée à la Société et à l'assuré, par application des deux précédents alinéas, comporte restitution par la Société des fractions de primes afférentes à la période pour laquelle les risques ne sont plus garantis.

Article 25

En cas de décès de l'assuré le contrat sera résilié, les primes échues restant acquises à la société.

Article 26

Pour tout assuré mobilisé ou engagé volontaire, la garantie sera suspendue le matin à zéro heure du jour où il doit se présenter à l'autorité militaire et les primes cesseront d'être dues. Elle reprendra ses effets dès le retour à la vie civile et la période en cours sera prorogée d'une durée égale à celle de la suspension.

Article 27

Les cas de faillite ou de liquidation judiciaire de l'assuré seront réglés conformément aux dispositions (article 17 du code CIMA) : **L'assurance subsiste en cas de faillite ou de liquidation judiciaire de l'assuré. Le syndic ou le débiteur autorisé par le juge ou le liquidateur selon le cas et l'assureur conservent le droit de résilier le contrat pendant un délai de trois mois à compter de la date du jugement de faillite ou de liquidation judiciaire. La portion de prime afférente au temps pendant lequel l'assureur ne couvre plus le risque est restituée au débiteur.**

En cas de faillite d'une entreprise d'assurance, les contrats qu'elle détient dans son porte feuille cessent de plein droit d'avoir effet le quarantième jour à midi, à compter de la publication dans un journal d'annonces légales, de la décision du retrait de l'agrément. Les primes sont dues proportionnellement à la période de garantie. Le syndic peut surseoir au paiement des sinistres.

En cas de retrait de l'agrément de la société, les dispositions de l'article 325-11 du code CIMA seront appliquées.

Article 28 PRESCRIPTION

Toute action dérivant du présent contrat est prescrite par deux ans a compter de l'événement qui y donne naissance dans les termes des articles 28 du Code CIMA.

BAREME SERVANT A LA DETERMINATION DU DEGRE D'IRNFIMITE PERMANENTE

1- INFIRMITE PERMANENTE TOTAL

- Cécité complète.....100%
- Perte totale des deux bras ou des deux mains.....100%
- Perte totale des deux jambes ou des deux pieds.....100%
- Perte totale d'un bras et d'une jambe - d'un bras et d'un pied - d'une main et d'une jambe - d'une main et d'un pied.....100%
- Quadriplégie, hémiplégie ou paraplégie complète (grabataire).....100%
- Aliénation mentale totale et incurable excluant t tout travail rémunérateur.....100%

II - INFIRMITE PERMANENTE PARTIELLE

A - TETE

- Perte complète de la vision d'un oeil (avec ou sans énucléation) ou réduction de la moitié de la fonction total de chacun des yeux.....25%
- Perte de substance du crâne dans toute son épaisseur :
  a) surface d'au moins 12 cm².....50%
  b) surface de 6 à 12 cm².....30%
  c) surface de 3 à 6 cm².....16%
  d) surface inférieure à 3 cm² .... Maximum.....10%
- surdité incurable et absolue des deux oreilles.....40%
- surdité incurable et absolue d'une oreille.....10%

- Ablation du maxillaire inférieur :
  a) totale.....35%
  b) partielle (branche montante en totalité ou demi du corps maxillaire).....25%
- Perte de toutes les dents supérieures et inférieures.....10%

B - MEMBRES

Membres supérieures	Droit	Gauche
- Amputation du bras ou de la main	60%	50%
- Perte totale du mouvement de l'épaule	30%	25%
- Perte totale du mouvement du coude	25%	20%
- Perte totale du mouvement du poignet	20%	15%
- Paralysie totale du membre supérieur	60%	50%
- Paralysie complète du nerf circonflexe	20%	15%
- Paralysie complète du nerf médian	40%	30%
- Paralysie complète du nerf cubital au coude	20%	15%
- Paralysie compléter du nerf cubital au poignet	12%	8%
- Paralysie complète du nerf radial, lésion au ni- veau de la gouttière de torsion	30%	20%
- Paralysie complète du nerf radial à l'avant-bras	30%	20%
- Fracture non consolidée du bras (pseudarthrose constituée):		
a) des deux os	25%	20%
b) d'un seul os	12%	7%
- Amputation du pouce :		
a) totale	20%	15%
b) partielle (phalange unguéale)	5%	3%
- Ankylose du pouce :		
a) totale	12%	8%
b) partielle (phalange unguéale)	5%	3%
- Amputation de l'index :		
a) totale	12%	8%
d) deux phalanges	8%	6%
c) une phalange	3%	2%
- Amputation totale ou médius	8%	6%
- Amputation totale de l'annulaire	6%	4%
- Amputation totale de l'auriculaire	5%	3%
- Amputation totale de deux de ces trois derniers doigts	15%	10%
- Amputation totale du pouce et de l'index	30%	25%
- Amputation totale du pouce et d'un doigt autre que l'index	25%	20%
- Amputation totale de l'index et d'un doigt autre que le pouce	20%	15%

Membres inférieurs

- Amputation de la cuisse :
a) au 1/3 supérieur.....60%
b) aux 2/3 inférieurs.....50%
- Amputation de la jambe.....40%
- Amputation du pied :
  a) totale.....35%
  b) sous-astragalienne.....30%
  c) médio-tarsienne.....30%
  d) tarso-métatarsinne.....25%
- Amputation de tous les orteils d'un pied.....15%
- Amputation du gros orteil.....7%
- Perte totale des quatre derniers orteils d'un pied.....6%
- Amputation d'un orteil (autre que le gros orteil).....1%
  a) en mauvaises attitude (flexion, adduction ou abduction).....40%
  b) en rectitude.....30%
- Perte totale des mouvements du genou (ankylose) :
  a) en flexion.....40%
  b) dans la rectitude.....20%
- Perte totale des mouvements de 'articulation tibio-tarsienne :
  a) en position favorable.....10%
  b) en position défavorable.....20%
- Fracture non consolidée de la cuisse ou des deux os de la jambe (pseudarthrose constituée).....40%
- Fracture mal consolidée d'un pied.....20%
- Fracture mal consolidée d'une rotule.....20%
- Raccourcissement d'un membre inférieur :
  a) d'au moins 8 cm.....15%
  b) de 5 cm.....9%
  c) de 3 cm.....4%
- Paralysie totale du membre inférieur.....55%
- Paralysie complète du sciatique poplité externe.....25%
- Paralysie complète du sciatique du poplité interne.....15%

C - TRONC

- Immobilisation d'un segment de la colonne vertébrale avec déviation prononcée et en position très gênante.....30%
- Fracture de côté avec déformation thoracique persistante et troubles fonctionnels.....10%

Conditions Générales Individuelle Accidents - C. P. A 7
Mise à jour 1er octobre 2011

C-3.2 Insurance for trial clinitians (Medical doctors)

COMPAGNIE PROFESSIONNELLE D'ASSURANCE DU CAMEROUN
S.A. au capital de 1 500 000 000 de Francs CFA entièrement libérés
Entreprise régie par le Code des Assurances - Arrêté d'agrément N° 00167/BIS/MINEFI/DCE/A du 03/09/1997
Siège Social et Direction Générale : 15, Rue Castelnau Akwa
R.C. N° 017867 - N° Contribuable : M O 99700005601 B
B.P. 54 Douala - Tél. : (237) 233 43 43 81 / 233 43 43 82 / 699 68 92 94 - Fax : (237) 233 43 43 84
E-mail : cpasiege@yahoo.fr - www.cpa-cameroun.com

POLICE

D'ASSURANCE

RESPONSABILITE CIVILE MEDICALE

N° 100358117DTR/2017

SOUSCRIPTEUR: UNIVERSITY OF BUEA
TAKEOFF RESEARCH PROJECT
Clinical trial on Doxycyline treatment of lymphodema due to podoconiosis

DUREE : UN (01) AN
EFFET : 1er DECEMBRE 2018
EXPIRATION : 31 DECEMBRE 2018

Accidents - Maladies - Corps de véhicule - Marchandises transportées - Incendie - Autres dommages aux biens
Responsabilité civile - Crédit - Caution - Pertes pécuniaires - Protection juridique - Assistance

ENGAGEMENT PROFESSIONNEL GARANTI

Entre

UNIVERSITY OF BUEA TAKEOFF RESEARCH PROJECT
Clinical trial on Doxycyline treatment of lymphodema due to podoconiosis

B.P 874 BUEA – CAMEROUN

Dénommée ci-après, le SOUSCRIPTEUR et/ou ASSURE

Et

COMPAGNIE PROFESSIONNELLE D'ASSURANCE DU CAMEROUN (CPA)

Siège Social & Direction Générale
15, Rue Castelnau – Akwa
B.P. 54 Douala – CAMEROUN
Tél : 33 43 43 81 / 33 43 43 82 / 33 42 81 17
Fax : 33 43 43 84

Dénommé ci-après, l'ASSUREUR

Il a été convenu ce qui suit :

Le contrat sera régi par les Dispositions du Code des Etats membre de la Conférence Interafricaine des Marchés d'Assurance (CIMA) et ses Décrets subséquents.

CONDITIONS PARTICULIERES

Souscripteur/Assuré	:	UNIVERSITY OF BUEA (TAKEOFF RESEARCH PROJECT) Clinical trial on Doxycyline treatment of lymphodema due to podoconiosis
Adresse	:	B.P. 874 BUEA – CAMEROUN
Police N°	:	100358117DTR/2017
Code Branche	:	581
Risque	:	RESPONSABILITE CIVILE MEDICALE
Durée	:	UN (01) AN
Effet	:	1er JANVIER 2018
Echéance	:	31 DECEMBRE 2018

DECOMPTE DE LA PRIME

PRIME NETTE	ACCESSOIRES	SOUS TOTAL	TVA	CP	PRIME TTC
1.800.000	10.000	1.810.000	348.425	50.000	2.208.425

La présente police est constituée par :

- Les Conditions Générales
- Les Conditions Particulières
- Les Clauses

D'autre part, il est convenu que les Conditions Particulières annulent et remplacent toutes les dispositions des Conditions Générales qui seraient plus restrictives pour l'assuré ou qui présenteraient, par rapport à celle-ci, une divergence ou une incompatibilité.

Fait en deux (02) exemplaires à Douala, le 27 novembre 2017

LE SOUSCRIPTEUR — **POUR LA COMPAGNIE**

Samuel Wanji
PROFESSOR OF PUBLIC HEALTH
PRINCIPAL INVESTIGATOR

UNIVERSITY OF BUEA TAKeOFF RESEARCH PROJECT — TAKeOFF Tackling the Obstacles to Fight Filariasis and Podoconiosis

2

Les présentes Conditions Particulières, seules s'appliquent lorsqu'elles sont contraires aux dispositions des Conditions générales en annexe, ou sont plus favorables à l'Assuré.

DECLARATIONS DU SOUSCRIPTEUR

1/ l'assuré déclare travailler avec douze personnes dont :

- **04 médecins**
- **04 infirmiers**
- **04 laborantins**

2/ ACTIVITE : CLINICAL TRIAL ON DOXYCYLINE TREATMENT OF LYMPHODEMA DUE TO PODOCONIOSIS.

CHAPITRE I : ASSURANCE RESPONSABILITE CIVILE EXPLOITATION

ARTICLE 1 - DEFINITIONS

SOUSCRIPTEUR

La personne qui demande l'établissement du contrat, le signe et s'engage à en payer la prime.

ASSURE

L'Assuré et ses associés à l'occasion de leurs activités communes.

- Lorsque l'assuré est une personne morale, le Président, les Administrateurs, les Directeurs Généraux et les gérants de la Société assurée, dans l'exercice de leurs fonctions.
- Les préposés, les stagiaires rémunérés ou non de l'assuré dans l'exercice de leurs fonctions.
- Les personnes salariées d'entreprises avec lesquelles l'assuré a passé une convention d'exploitation, les dites personnes devenant préposés de l'assuré.

ASSUREUR :

La Société d'assurances auprès de laquelle le contrat est souscrit.

3

LIVRAISON :

La remise effective par l'assuré d'un produit ou matériel à un tiers dès lors que cette remise fait perdre à l'assuré son pouvoir d'usage et de contrôle sur ce bien ou sur ce produit.

Le terme **LIVRAISON** est également applicable à la réception (même provisoire) des travaux exécutés et des prestations de services effectués.

DOMMAGES

Préjudice corporel, matériel ou immatériel résultant de tout événement fortuit, imprévu et extérieur à la victime ou à la chose endommagée.

CORPOREL : Préjudice pécuniaire résultant de toute atteinte physique subie par une personne.

MATERIEL : Préjudice pécuniaire résultant de toute détérioration ou destruction d'une chose ou substance, toute atteinte physique à des animaux.

IMMATERIEL : Préjudice pécuniaire résultant de la privation de jouissance d'un droit, toute interruption d'un service rendu par une personne par un bien meuble ou immeuble ou de la perte d'un bénéfice, et qu'entraîne directement la survenance de dommages corporels ou matériels garantis par le contrat.

TIERS : Toute personne autre que :

- Le conjoint de l'assuré,
- l'assuré et à l'occasion de leurs activités communes ses associés.
- Les ascendants et descendants de l'assuré et de leurs conjoints,
- Lorsque l'assuré est une personne morale, le Président, les Administrateurs, les Directeurs Généraux et les Gérants de la Société.
- Les préposés et salariés de l'assuré responsables dans l'exercice de leurs fonctions.

SINISTRE :

Toute réclamation formulée entre les dates de prise d'effet et d'expiration des effets du contrat étant entendu que l'ensemble des réclamations se rattachant à un même fait dommageable constitue un seul et même sinistre.

FRANCHISE : Part du sinistre indemnisable restant toujours à la charge de l'assuré.

PRIME : La somme que doit verser le Souscripteur en contrepartie de la garantie de l'assureur.

ANNEE D'ASSURANCE

Période comprise entre deux échéances annuelles de primes. Toutefois, si la date de la prise d'effet du contrat est distincte de l'échéance annuelle, il faut entendre par « Année d'Assurance », la période comprise entre cette date et la prochaine échéance annuelle. Si le contrat expire entre deux échéances annuelles, la dernière date d'échéance annuelle et la date d'expiration du contrat.

ECHEANCE PRINCIPALE :

La date à laquelle est due la prime.

ARTICLE 2 – OBJET DE LA GARANTIE

Le présent contrat garantit l'assuré contre les conséquences pécuniaires de la Responsabilité Civile que celui-ci peut encourir en vertu des Articles 1382 à 1386 du Code Civil en raison des dommages corporels, matériels et immatériels causés aux tiers par un accident dans l'exercice de la profession déclarée ci-dessus, par un incendie et/ou Explosion, des dégâts des eaux résultant de la profession de l'assuré à l'extérieur de son établissement.

ARTICLE 3 – EXCLUSIONS

Outre les exclusions comme il est dit aux conditions Générales, sont également exclues de la garantie, les conséquences pécuniaires de la Responsabilité Civile encourue par l'Assuré :

3.1 En vertu d'obligations contractuelles,
3.2 A raison des dommages corporels, matériels et immatériels causés par toutes atteintes à l'environnement,
3.3 A raison des dommages résultant de la participation de l'assuré ou des personnes dont il est civilement responsable,
– en tant que concurrents ou organisateurs à des matches, paris, compétitions, concours, courses et leurs essais,
- en tant qu'organisateurs à des foires et expositions,
3.4 A raison des dommages corporels causés par tout matériel ou installation dont l'assuré ou les personnes dont il est civilement responsable ont la propriété, la conduite, la garde ou l'usage, à savoir :
- **Le matériel et les installations ferroviaires notamment les voies et raccordements et le matériel roulant sur ces voies.**
- **Tous engins flottants et aériens, tous véhicules aériens, maritimes, fluviaux ou lacustres,**

3.5 A raison des dommages aux existants,
3.6 Tous dommages corporels, matériels et immatériels découlant de l'activité d'expertise, d'audit et de conseil

5

ARTICLE : 4 GARANTIES COMPLEMENTAIRES

Par dérogation aux Conditions Générales, la garantie du présent contrat est étendue aux conséquences pécuniaires de la responsabilité Civile pouvant incomber à l'assuré dans les cas suivants :

4. 1 INCENDIE / EXPLOSION

Du fait des dommages matériels et immatériels consécutifs résultant d'incendie, d'explosion, d'incident d'origine électrique, de fumées, de gaz et survenus :

- Hors des locaux professionnels de l'assuré, à l'occasion notamment de ses activités de livraison, de montage ou installation ;
- Dans les installations de l'assuré ayant reçu une affectation temporaire de chantier,
- La garantie est également acquise à l'assuré pour les dommages énoncés ci-dessus causés à des voisins ou des tiers dont l'origine peut être située dans un bâtiment ou sur des terrains non boisés appartenant à l'assuré, loués ou occupés par lui, lorsqu'ils ne proviennent pas d'une explosion ou de la communication de l'incendie de ses immeubles ou de matériels, marchandises ou approvisionnements qui y sont entreposés.

SONT EXCLUS LES DOMMAGES :

4.1.1 MATERIELS & IMMATERIELS CONSECUTIFS

- **Résultant d'incendie, d'explosion, d'incident d'origine électrique, de fumées, de gaz provenant des communications ou survenus dans les locaux (bâtiments proprement dits, leurs installations, leur contenu) dont l'assuré est propriétaire, locataire ou qu'il occupe de façon durable pour les besoins de sa profession.**

4.1.2 MATERIELS & IMMATERIELS CONSECUTIFS DONNANT LIEU :

a) **A la responsabilité Locative de l'assuré en tant que locataire ou occupant à l'égard des propriétaires des immeubles occupés par lui, telle qu'elle résulte de l'application des articles 1733, 1734, 1735 et 1302 du Code Civil.**

b) **Au recours des locataires dans le cas où l'assuré est propriétaire.**

4.1.3 Provoqués par des véhicules terrestres à moteur faisant l'objet de législation sur l'assurance automobile obligatoire et dont l'assuré est propriétaire, locataire ou gardien.

4.1.4 Résultant d'un excès de chaleur sans embrasement,

4.1.5 Subis par les fournitures et matériaux non réceptionnés de l'assuré.

4.2 EAUX ET FLUIDES DIVERS

6

Du fait des dommages matériels et immatériels consécutifs survenus EXCLUSIVEMENT hors des locaux professionnels de l'assuré à l'occasion notamment de ses activités de livraison, de montage ou installation, causés au tiers et résultant de l'action des eaux ou autres fluides.

SONT EXCLUS LES DOMMAGES :

4.2.1 Matériels et Immatériels Consécutifs

- **Résultant de l'action des eaux ou autres liquides et causés :**
- **Par fuites ou débordements provenant des locaux ou survenues dans les locaux (les bâtiments proprement dits et leur contenu, leurs installations d'adduction, de distribution, d'évacuation, installation de chauffage central, réservoirs, chenaux, etc..) dont l'assuré est propriétaire, locataire ou qu'il occupe de façon durable pour les besoins de sa profession.**
- **Ou par infiltration ou débordement de bassins, nappes naturels ou artificielles, cours d'eau, ainsi que tous dommages résultant d'une modification du régime des eaux, tels que tarissement de points d'eau ou dessèchement de nappes ou terrains.**

CHAPITRE II : ASSURANCE RESPONSABILITE CIVILE PROFESSIONNELLE

ARTICLE 1 : OBJET DE LA GARANTIE

Par la présente assurance Responsabilité Civile Professionnelle, la compagnie garantit les conséquences de la responsabilité civile pouvant incomber à l'assuré à raison des dommages corporels ou matériels résultant d'accidents causés à autrui, dans l'exercice sa profession y compris :

a) Aux termes des articles 1382 à 1386 du code civil et à raisons des dommages corporels, matériels et immatériels qui en sont la conséquence, causés aux patients ou aux tiers par suite d'accidents survenus dans l'exercice de leur profession.

b) Aux termes des dispositions légales en vigueur en raison des dommages corporels, matériels et immatériels causés aux malades en traitement ou en consultation à la suite :

- D'erreurs ou négligences commises par le personnel dans l'exécution des ordonnances, la fourniture des médicaments, les indications résultant des analyses ou pour tout autre cause.
- D'erreurs professionnelles commises dans l'exercice de leur art par les membres du personnel médical, paramédical, soignant ou assimilé ou par les aides bénévoles.

7

ARTICLE 2 : CHAMP D'APPLICATION DE LA GARANTIE :

La garantie s'applique aux réclamations écrites, formulées amiablement ou judiciairement auprès de l'Assuré au cours de la période comprise entre la date d'effet et de résiliation du contrat.
La garantie ne s'applique pas pour des faits générateurs d'origine antérieure à la date de prise d'effet du contrat, mais dont les conséquences surviennent pendant la période de validité du contrat.

ARTICLE 3 : CONDITIONS D' APPLICATION DE LA GARANTIE :

Sont considérés comme formant un seul et même sinistre, les réclamations relatives à des dommages résultant d'une même faute ou d'une même négligence. Dans ce cas, la totalité du sinistre est imputée à l'année d'assurance au cours de laquelle a été présentée la première réclamation.
Toutefois, la garantie ne joue que pour les réclamations présentées pendant la période de validité du Contrat.

ARTICLE 4 : SAUVEGARDE DES DROITS DES PERSONNES LESEES :

Ne sont pas opposables aux personnes lésées et à leurs ayants - droit ni de déchéances motivées par un manquement de l'Assuré à ses obligations, commis postérieurement au sinistre, ni les franchises relatives aux dommages corporels.
Dans ces cas, l'Assureur procède au paiement de l'indemnité pour le compte de l'Assuré responsable.

Il peut exercer contre ce dernier une action en remboursement pour toutes les sommes qu'il a ainsi payées à sa place.

ARTICLE 5 : RENONCIATION DU DROIT A RECOURS

L' Assureur renonce à tous recours contre les personnes physiques ou morales, de droit public ou de droit privé et leur personnel, avec lesquelles l'assuré a ou peut avoir des communautés d'intérêts de travaux, de personnel ou encore des liaisons de nature contractuelle, chaque fois que l'assuré a effectué pareille renonciation, étant précisé que l'assureur conserve sont droit de recours contre les sous-traitants.

ARTICLE 6 : EXCLUSIONS :

Outre les exclusions prévues par l'article 4 des Conditions Générales sont également exclus:

- Les conséquences des expérimentations de produits pharmaceutiques effectués avant leur commercialisation soit dans le cadre de la recherche, soit pour la constitution du dossier de demande d'autorisation de mise sur le marché ;

- Tous les actes médicaux prohibés par la loi

- Les conséquences de la divulgation des documents ou des renseignements secrets sauf si cette divulgation est le fait des préposés de l'assuré ;

ARTICLE 7 : ETENDUE TERRITORIALE

Les garanties du présent Contrat s'exercent uniquement en **REPUBLIQUE DU CAMEROUN**

Chapitre III : NATURE ET MONTANT DES GARANTIES.

NATURE DES GARANTIES	MONTANT DES GARANTIES
**RESPONSABILITE CIVILE EXPLOITATION**	
- Dommages Corporels<br>- Dommages Exceptionnels	500.000.000 F.CFA
- Lésions corporelles provenant d'intoxications.....	125.000.000 F.CFA
- Dommages Matériels et Immatériels consécutifs Dont :	50.000.000 F.CFA
- Incendie/Explosion hors locaux professionnels	20.000.000 F.CFA
- Dégâts des Eaux hors locaux professionnels	10.000.000 F.CFA
- Défenses et Recours	1.000.000 F.CFA
**RESPONSABILITE CIVILE PROFESSIONNELLE**	
- Tous dommages confondus	25.000.000 F.CFA

FRANCHISES : **- RC EXPLOITATION : 10% minimum 150.000 F.CFA hors corporel**

- RC PROFESSIONNELLE : 10% minimum 500.000 F.CFA hors corporel

ARTICLE 8 : DECOMPTE DE LA PRIME

La prime totale à payer par le souscripteur au titre du présent contrat s'élève à deux millions deux cent huit mille quatre cent vingt cinq francs (2.208.4025) F CFA décomptée comme suit :

Prime nette	:	1.800.000
Accessoires	:	10.000
T V A	:	348.425
CP	:	50.000
PRIME T T C	:	2.208.425

ARTICLE 9 : DUREE DU CONTRAT

La présente police est souscrite pour une durée ferme de DOUZE (12) mois, allant du **1er JANVIER 2018 AU 31 DECEMBRE 2018.** Elle pourra être renouvelée sur demande de l'assuré et après acceptation de l'assureur.

Fait en deux (02) exemplaires à Douala le, 27 novembre 2017.

LE SOUSCRIPTEUR **P/LA COMPAGNIE**

Samuel Wanji
PROFESSOR OF PUBLIC HEALTH
PRINCIPAL INVESTIGATOR

UNIVERSITY OF BUEA · TAKeOFF RESEARCH PROJECT

9

CONDITIONS GENERALES ASSURANCE TOUS RISQUES CHANTIER

Le présent contrat est régi par le code des Assurances CIMA, Les présentes conditions générales et celles particulières jointes

ARTICLE 1er - DEFINITIONS

On entend par :

a) **SOUSCRIPTEUR :**

La personne physique ou morale, telle qu'elle est désignée aux Conditions particulières, ou toute personne qui lui serait substituée par accord des parties.

b) **ASSURE :**

1) Pendant toute la durée du contrat :

- Le Souscripteur sauf s'il est également le Maître de l'ouvrage
- Toute entreprise tenue de travailler sur le chantier, les divers sous-traitants et fournisseurs directs du chantier.

2) Jusqu'à la réception provisoire :

- Le Maître de l'Ouvrage tel qu'il est désigné aux Conditions particulières.

c) **TIERS :**

Toute personne physique ou morale autre que le Souscripteur ou l'Assuré ci-dessus définis, sauf dérogation prévue à l'article 2 § C.

d) **ACCIDENT :**

Tout événement soudain et extérieur à la victime ou au bien endommagé, constituant la cause, soit d'une atteinte corporelle à la victime, soit d'une détérioration ou destruction du bien endommagé.

OBJET DU CONTRAT

Le présent contrat garantit l'Assuré sous réserve des exclusions expressément énumérées plus loin, contre ceux des risques ci-après, désignés comme couverts aux Conditions particulières.

ARTICLE 2 – RISQUES GARANTIS

A – RISQUES – DOMMAGES - PENDANT LA PERIODE DE CONSTRUCTION

Toutes pertes ou dommages subis par les biens suivants, alors qu'ils se trouvent sur le lieu du chantier, appartenant à l'Assuré ou dont il a la possession, la garde, ou la détention ;

1) L'ouvrage, objet du Marché spécifié aux Conditions particulières, jusqu'à sa réception provisoire ;

2) L'ouvrage provisoire prévu à ce Marché ou nécessaire à son exécution ;

3) Les matériaux et matériels approvisionnés sur le chantier et destinés à être incorporés dans les ouvrages définitifs ou provisoires ;

4) Les installations provisoires de chantier ainsi que les matériels et engins de chantier, désignés aux conditions particulières, utilisés pour l'exécution du Marché.

B – RISQUES GARANTIS PENDANT LA PERIODE DE MAINTENANCE

a) Pour les travaux de construction ou de Génie Civil :

Toutes pertes ou dommages subis par tout ou partie de l'ouvrage provenant exclusivement :

- Soit de vice de matériaux ou de mise en œuvre défectueuse pour une cause antérieure à cette réception provisoire ;
- Soit de l'utilisation ou de l'occupation par l'Assuré d'une partie quelconque de l'ouvrage, après sa prise de possession par le Maître de l'ouvrage ou après réception, et ce pour autant que la Responsabilité de l'Assuré soit engagée.

b) Pour les travaux de montage ou d'installation de machines :

Toutes pertes ou dommages subis par tout ou partie des machines et installations qui proviendraient exclusivement d'accidents résultant de négligence, maladresse, fausse manœuvre, imputables à l'Assuré et qui surviendraient pendant cette période, lors de l'exécution des tâches incombant à l'assuré.

C – RISQUES « RESPONSABILITE CIVILE »

Les conséquences pécuniaires de la Responsabilité Civile délictuelle ou quasi-délictuelle incombant à

l'Assuré en raison des dommages corporels résultant d'accident, d'incendie ou d'explosion et des dommages matériels résultant d'accident, ainsi que des dommages immatériels qui en sont la conséquence subis par un tiers, imputables à l'exécution de l'ouvrage et survenant pendant la période de validité du contrat, les entreprises coopérant à cet ouvrage étant considérées comme tiers entre elles, mais seulement en ce qui concerne les dommages corporels.

Moyennant mention aux Conditions particulières et surprime, cette garantie est étendue à certaines conséquences pécuniaires de la Responsabilité Civile incombant à l'Assuré en raison des dommages corporels et matériels autres que ceux prévus aux paragraphe A et B du présent article et immatériels qui en sont la conséquence, mises conventionnellement à sa charge par les clauses et conditions du Marché.

Ne sont pas considérés comme constituant un accident les dommages résultant d'une façon normalement prévisible et/ou inéluctable, de la nature même de l'activité de l'Assuré ou des modalités d'exécution des travaux, tels qu'ils ont été prescrits ou acceptés par la Direction de l'entreprise assurée.

ARTICLE 3 - RISQUES EXCLUS

Sont exclus de la garantie :

A – EN CE QUI CONCERNE L'ENSEMBLE DES RISQUES COUVERTS :

1) Les dommages intentionnellement causés ou provoqués par l'Assuré ainsi que ceux provenant de vices ou défauts existant au moment de la souscription du contrat et intentionnellement non déclarés à l'Assureur :

2) Les dommages résultant d'un arrêt même partiel des travaux ;

3) Les dommages occasionnés par la guerre étrangère, la guerre civile, les émeutes ou les mouvements populaires ;
Les dommages occasionnés par les grèves ou les insurrections ;
Les dommages occasionnés par un acte de terrorisme ou de sabotage accompli dans le cadre d'actions concertées de terrorisme ou de sabotage, ces sinistres étant assimilés, en ce qui concerne la charge de la preuve, à ceux occasionnés par la guerre civile ;

4) Les dommages résultant des effets directs ou indirects d'explosion, de dégagement de chaleur, d'irradiation, provenant de transmutation de noyau d'atome ou de la radioactivité, ainsi que des effets de radiations provoquées par l'accélération artificielle de particules.

B – EN CE QUI CONCERNE LES RISQUES «DOMMAGES » (Article 2, § A)

Les pertes ou dommages qui surviendraient à l'ouvrage après prise de possession par le Maître de l'ouvrage ou après réception, sauf ceux qui seraient éventuellement garantis au titre de l'article 2, § B, du présent contrat.

C - EN CE QUI CONCERNE LES RISQUES « DOMMAGES » ET « PERIODE DE MAINTENANCE » (Article 2, § A et B) :

1) Les pertes résultant de la dépréciation due à l'usure, à la corrosion, au vieillissement ou de la détérioration provenant d'une altération de substance ainsi que celle causées directement par les pannes ou dérèglements mécaniques ou électriques, les dommages accidentels dont ces phénomènes seraient la cause restant toutefois couverts ;

2) Les dommages résultant de réparations provisoires ou de fortune, ainsi que ceux résultant des recherches expérimentales ;

3) – a) Tous les frais, quels qu'ils soient, qui seraient engagés pour rechercher ou supprimer des défauts, pour rectifier des vices de plan, pour mettre les biens faisant l'objet des garanties du présent contrat en conformité avec les spécifications techniques du Marché et du Cahier des Charges, ou pour apporter à ces biens une modification ou un perfectionnement quelconque ;

b) Sont également exclus les pertes ou dommages causés directement par ces malfaçons, matériaux défectueux, édifications d'ouvrages en non conformité avec les prescriptions du Cahier des Charges, vices de plan. Il est toutefois précisé que cette exclusion des pertes ou dommages ainsi définis ne s'applique qu'aux machines, matériels, et/ou à la partie de l'ouvrage directement touchés par l'événement ;

Restent par contre garantis, dans le cadre de la présente assurance, les pertes ou dommages atteignant d'autres machines, matériels ou parties d'ouvrage et qui seraient la conséquence des évènements visés au présent paragraphe b).

4) Les dommages dus à l'inobservation inexcusable des règles de l'art définies par les documents techniques élaborés par les organisations professionnelles ou par les prescriptions techniques des constructeurs de matériels ou machines lorsque cette inobservation est le fait de la Direction de l'entreprise assurée ou des personnes substituées à cette Direction sur le chantier ET EN CE QUI CONCERNE SEULEMENT LES TRAVAUX DE BATIMENT ET DE GENIE CIVIL et sauf stipulation contraire, les dommages dus à une erreur de conception ;

5) Les pertes ou dommages survenant :

- aux appareils de navigation aérienne, maritime ou fluviale,
- aux véhicules servant au transport de personnes et appartenant, loués ou confiés à l'Assuré ou à son personnel ;
- à tous véhicules automoteurs sauf lorsqu'ils sont utilisés sur le chantier en tant qu'outils de travail et pour autant qu'ils ne soient pas couverts d'autre part ;
- aux espèces, valeurs, dessins, titres et archives de toute nature ;

6) Les pertes ou manquants constatés pendant ou après inventaire ainsi que les vols commis par les membres de la famille de l'Assuré visés par l'Art. 318 et 55 du Code Pénal ou par ses préposés pendant leur service ;

7) Le préjudice résultant de tous dommages indirects tels que le chômage, la privation de jouissance, la dépréciation, ainsi que les amendes ou pénalités de quelque nature qu'elles soient pour retard de livraison, inobservation des délais ou toute autre cause.

D – EN CE QUI CONCERNE LES RISQUES « PERIODE DE MAINTENANCE » ET « RESPONSABILITE CIVILE » (Article 2, § B et C) :

1) Les dommages causés par les appareils de navigation aérienne, maritime ou fluviale ainsi que par les véhicules terrestres à moteur soumis à l'obligation légale d'assurance ;

2) Sauf dérogation aux Conditions Particulières et indépendamment des garanties pouvant être accordées au titre de l'article 2 § B, les responsabilités que l'Assuré aurait acceptées par convention ou contrat et qu'il n'aurait pas encourues sans ces conventions ou contrats ;

3) Les dommages immatériels qui ne seraient pas la conséquence d'un dommage matériel garanti.

E – EN CE QUI CONCERNE LES RISQUES « RESPONSABILITE CIVILE » (Article 2, § C) :

1) Les dommages subis par le conjoint, les ascendants et les descendants de l'Assuré responsable du sinistre ;

2) Les dommages subis par les salariés ou préposés de l'Assuré responsable lorsque les dommages sont survenus dans l'exercice de leurs fonctions professionnelles ;

3) Lorsque l'Assuré est une personne morale, les dommages subis par ses représentants légaux, si ces dommages sont survenus au cours de leurs activités professionnelles ;

4) Les dommages causés aux biens dont l'Assuré ou les personnes dont il est civilement responsable ont la propriété, la garde, la possession ou la détention.

ARTICLE 4 – PERIODE DE GARANTIE.

A - En ce qui concerne les risques définis à l'article 2, § A, la garantie commence pour chaque bien ou objet assuré, après son déchargement sur le chantier et se termine à la fin des essais dont la durée, sauf convention spéciale mentionnée aux Conditions Particulières, est limitée à quatre semaines ou à la première des dates suivantes, si elle est antérieure à la fin des essais :

- Mise en service ;
- Réception provisoire, formelle ou de fait ;
- Prise de possession par le Maître d'ouvrage.

Si une partie seulement des travaux, constructions, usines ou machines a été réceptionnée, mise en service ou prise en charge par le Maître d'Ouvrage comme il est dit ci-dessus, la garantie est maintenue pour les autres parties.

B - En ce qui concerne les risques définis à l'Article 2, § B, la garantie prend effet immédiatement après la fin de la période de garantie définie au § A précèdent et ce pour une durée fixée aux Conditions Particulières.

C - En ce qui concerne les risques définis à l'Article 2 - § C, la période de garantie est celle de la validité du contrat, sauf stipulation contraire prévue aux Conditions Particulières.

ARTICLE 5 – MONTANT DE LA GARANTIE

La garantie est délivrée dans la limite des sommes et sous déduction des franchises respectivement fixées aux Conditions Particulières.

Cette garantie est réduite de plein droit, après sinistre, du montant de l'indemnité correspondante tant que le Souscripteur n'a pas demandé, par lettre recommandée, le rétablissement de la garantie en s'engageant à payer la prime complémentaire calculée sur les bases de la prime initiale, au prorata de la durée restant à courir.

ARTICLE 6 – FORMATION ET EFFET DU CONTRAT.

La prise d'effet du présent contrat est subordonnée au paiement intégral de la prime.

Les mêmes dispositions s'appliquent à tout avenant au contrat.

ARTICLE 7 – RESILIATION DU CONTRAT.

Le contrat peut être résilié avant sa date d'expiration normale dans les cas et conditions fixées ci-après :

1°/ Par le souscripteur ou l'assureur :

a) A chaque échéance annuelle du contrat, moyennant deux mois au moins de

5) Les pertes ou dommages survenant :

- aux appareils de navigation aérienne, maritime ou fluviale,
- aux véhicules servant au transport de personnes et appartenant, loués ou confiés à l'Assuré ou à son personnel ;
- à tous véhicules automoteurs sauf lorsqu'ils sont utilisés sur le chantier en tant qu'outils de travail et pour autant qu'ils ne soient pas couverts d'autre part ;
- aux espèces, valeurs, dessins, titres et archives de toute nature ;

6) Les pertes ou manquants constatés pendant ou après inventaire ainsi que les vols commis par les membres de la famille de l'Assuré visés par l'Art. 318 et 55 du Code Pénal ou par ses préposés pendant leur service ;

7) Le préjudice résultant de tous dommages indirects tels que le chômage, la privation de jouissance, la dépréciation, ainsi que les amendes ou pénalités de quelque nature qu'elles soient pour retard de livraison, inobservation des délais ou toute autre cause.

D – EN CE QUI CONCERNE LES RISQUES « PERIODE DE MAINTENANCE » ET « RESPONSABILITE CIVILE » (Article 2, § B et C) :

1) Les dommages causés par les appareils de navigation aérienne, maritime ou fluviale ainsi que par les véhicules terrestres à moteur soumis à l'obligation légale d'assurance ;

2) Sauf dérogation aux Conditions Particulières et indépendamment des garanties pouvant être accordées au titre de l'article 2 § B, les responsabilités que l'Assuré aurait acceptées par convention ou contrat et qu'il n'aurait pas encourues sans ces conventions ou contrats ;

3) Les dommages immatériels qui ne seraient pas la conséquence d'un dommage matériel garanti.

E – EN CE QUI CONCERNE LES RISQUES « RESPONSABILITE CIVILE » (Article 2, § C) :

1) Les dommages subis par le conjoint, les ascendants et les descendants de l'Assuré responsable du sinistre ;

2) Les dommages subis par les salariés ou préposés de l'Assuré responsable lorsque les dommages sont survenus dans l'exercice de leurs fonctions professionnelles ;

3) Lorsque l'Assuré est une personne morale, les dommages subis par ses représentants légaux, si ces dommages sont survenus au cours de leurs activités professionnelles ;

4) Les dommages causés aux biens dont l'Assuré ou les personnes dont il est civilement responsable ont la propriété, la garde, la possession ou la détention.

ARTICLE 4 – PERIODE DE GARANTIE.

A - En ce qui concerne les risques définis à l'article 2, § A, la garantie commence pour chaque bien ou objet assuré, après son déchargement sur le chantier et se termine à la fin des essais dont la durée, sauf convention spéciale mentionnée aux Conditions Particulières, est limitée à quatre semaines ou à la première des dates suivantes, si elle est antérieure à la fin des essais :

- Mise en service ;
- Réception provisoire, formelle ou de fait ;
- Prise de possession par le Maître d'ouvrage.

Si une partie seulement des travaux, constructions, usines ou machines a été réceptionnée, mise en service ou prise en charge par le Maître d'Ouvrage comme il est dit ci-dessus, la garantie est maintenue pour les autres parties.

B - En ce qui concerne les risques définis à l'Article 2, § B, la garantie prend effet immédiatement après la fin de la période de garantie définie au § A précèdent et ce pour une durée fixée aux Conditions Particulières.

C - En ce qui concerne les risques définis à l'Article 2 - § C, la période de garantie est celle de la validité du contrat, sauf stipulation contraire prévue aux Conditions Particulières.

ARTICLE 5 – MONTANT DE LA GARANTIE

La garantie est délivrée dans la limite des sommes et sous déduction des franchises respectivement fixées aux Conditions Particulières.

Cette garantie est réduite de plein droit, après sinistre, du montant de l'indemnité correspondante tant que le Souscripteur n'a pas demandé, par lettre recommandée, le rétablissement de la garantie en s'engageant à payer la prime complémentaire calculée sur les bases de la prime initiale, au prorata de la durée restant à courir.

ARTICLE 6 – FORMATION ET EFFET DU CONTRAT.

La prise d'effet du présent contrat est subordonnée au paiement intégral de la prime.

Les mêmes dispositions s'appliquent à tout avenant au contrat.

ARTICLE 7 – RESILIATION DU CONTRAT.

Le contrat peut être résilié avant sa date d'expiration normale dans les cas et conditions fixées ci-après :

1°/ Par le souscripteur ou l'assureur :

a) A chaque échéance annuelle du contrat, moyennant deux mois au moins de

Il doit en outre déclarer, en cours de contrat, les circonstances nouvelles qui ont pour conséquence, soit d'aggraver les risques, soit d'en créer de nouveaux et rendent de ce fait inexactes ou caduques les réponses faites à l'assureur, notamment dans le formulaire mentionné au 2° ci-dessus.

B) En cours de contrat, le Souscripteur doit déclarer à l'Assureur, par lettre recommandée, toutes les modifications affectant les éléments constitutifs du risque suivants : nature et consistance de l'ouvrage, moyens d'exécution, durée des travaux, ainsi que ceux spécifiés aux Conditions Particulières.

Cette déclaration doit être faite préalablement à la modification si celle-ci résulte du fait de l'Assuré et dans les autres cas, dans les quinze jours à partir du moment où le Souscripteur en a eu connaissance.

Si ces modifications constituent une aggravation telle que, si le nouvel état de chose avait existé lors de la souscription, l'Assureur n'aurait pas contracté, ou ne l'aurait fait que moyennant une prime plus élevée,

La DECLARATION DOIT EN ETRE FAITE SOUS PEINE DES SANCTIONS PREVUES AU PARAGRAPHE C) CI-DESSOUS.

Dans le cas d'une telle aggravation, L'Assureur a la faculté, soit de résilier le contrat moyennant un préavis de dix jours par lette recommandée, soit de proposer un nouveau taux de prime. Si le Souscripteur n'accepte pas celui-ci, l'Assureur peut résilier le contrat.

C) Toute réticence ou déclaration intentionnellement fausse, toute omission ou déclaration inexacte entraîne, lorsqu'elle change l'objet du risque ou en diminue l'opinion pour l'Assureur, l'application, suivant le cas, des sanctions prévues aux articles 18 et 19 du Code des Assurances. Les sanctions opposables au Souscripteur le sont également à toute personne ayant la qualité d'Assuré.

ARTICLE 9 – DECLARATION DES AUTRES ASSURANCES

Si les risques couverts par le présent contrat sont ou viennent à faire l'objet d'une autre assurance, le Souscripteur doit le déclarer à l'Assureur dans les formes et délais prévus à l'article 8. Par ailleurs, celui qui est assuré auprès de plusieurs assureurs par plusieurs polices, pour un même intérêt, contre un même risque, doit donner immédiatement à chaque assureur connaissance des autres assureurs.

L'assuré doit, lors de cette communication, faire connaître le nom de l'assureur avec lequel une autre assurance a été contractée et indiquer la somme assurée.

Quand plusieurs assurances contre un même risque sont contractées de manière dolosive ou frauduleuse, les sanctions prévues à l'article 33, premier alinéa, sont applicables.

Quand elles sont contractées sans fraude, chacune d'elle produit ses effets dans les limites des garanties du contrat et dans le respect des dispositions de l'article 31, quelle que soit la date à laquelle l'assurance aura été souscrite. Dans ces limites, le bénéficiaire du contrat peut obtenir l'indemnisation de ses dommages en s'adressant à l'assureur de son choix.

Dans les rapports entre assureurs, la contribution de chacun d'eux est déterminée en appliquant au montant du dommage le rapport existant entre l'indemnité qu'il aurait versée s'il avait été seul et le montant cumulé des indemnités qui auraient été à la charge de chaque assureur s'il avait été seul.

ARTICLE 10 – CALCUL DE LA PRIME

La prime afférente au présent contrat est calculée par application, aux capitaux représentatifs du prix des biens assurés, des taux ressortis aux Conditions Particulières.

A) EN CE QUI CONCERNE LES TRAVAUX ASSURES,

Ces capitaux devront représenter leur prix pour le Maître de l'Ouvrage, tel qu'il ressort du Marché au jour de la souscription du contrat. L'exacte déclaration de ce prix à la souscription du contrat exclut l'application ultérieure de la règle proportionnelle prévue par l'article 35 du Code des Assurances; en contrepartie, s'il apparaît après achèvement des travaux que le Maître de l'Ouvrage est redevable au Souscripteur pour quelque raison que ce soit d'une majoration du prix total initialement envisagé, le Souscripteur devra le déclarer à l'Assureur et supporter un ajustement proportionnel de prime.

B) EN CE QUI CONCERNE LES MATERIELS, ENGINS, INSTALLATIONS ET EQUIPEMENTS DE CHANTIER, ces capitaux devront représenter la valeur maximum de remplacement de l'ensemble. Si la valeur déclarée n'est pas cette valeur de remplacement, la règle proportionnelle prévue à l'article 15 ci-dessous sera appliquée.

ARTICLE 11 – PAIEMENT DES PRIMES.

La prime et les accessoires de prime dont le montant est stipulé au contrat, ainsi que les impôts et taxes sur les contrats d'assurance dont la récupération n'est pas interdite, sont payables d'avance au domicile de l'assureur ou de l'intermédiaire, dans les conditions prévues à l'article 541 du Code des Assurances.

A défaut de paiement d'une prime ou d'une fraction de prime dans le délai convenu, le contrat est résilié de plein droit, la portion de prime

courue reste acquise à l'assureur, sans préjudice des éventuels frais de poursuite de recouvrement.

ARTICLE 12 – OBLIGATIONS DE L'ASSURE EN CAS DE SINISTRE

Aussitôt qu'un sinistre se déclare, le Souscripteur et l'Assuré doivent user de tous les moyens en leur pouvoir pour en arrêter les progrès, sauver les objets assurés et veiller ensuite à leur conservation.

Ils doivent, sous peine de déchéance, dès qu'ils ont connaissance d'un sinistre susceptible d'engager la garantie du présent contrat, et au plus tard dans les cinq jours, sauf cas fortuit ou de force majeure, en donner avis par écrit ou verbalement contre récépissé au Siège Social de l'Assureur ou à l'Agence indiquée sur le contrat.

Ils doivent, en outre, dans le plus bref délai :

1) indiquer à l'Assureur la date et le lieu du sinistre, ses circonstances et ses causes connues ou présumées, la nature et le montant approximatif des dommages ;

2) fournir à l'Assureur un état estimatif, certifié sincère et signé par eux, des objets assurés détruits et sauvés ;

3) transmettre à l'Assureur tous avis, lettres, convocations, assignations, actes extrajudiciaires et pièces de procédure qui seraient adressés, remis ou signifiés à eux-mêmes ou à leurs préposés, concernant un sinistre susceptible d'engager une responsabilité couverte par le présent contrat ;

4) en cas de vol ou de perte, aviser dans les quarante-huit heures l'Assureur et les Autorités locales de Police ou toute autre Autorité compétente en la matière, déposer une plainte ;

5) en cas de dommages causés à des tiers, déclarer les noms et adresses des lésés, ceux des témoins et éventuellement, de l'auteur responsable, l'importance et la nature du sinistre et, d'une manière générale, donner tous renseignements utiles à l'appréciation des responsabilités encourues et des réparations éventuellement dues.

Faute par le Souscripteur ou l'Assuré de remplir tout ou partie des obligations prévues aux cinq paragraphes ci-dessus et sauf les cas fortuit ou de force majeure, l'Assureur peut lui réclamer une indemnité proportionnée au dommage que le manquement de l'Assuré ou du Souscripteur peut lui causer.

L'assuré qui, de mauvaise foi, exagère le montant des dommages, prétend détruits des objets n'existant pas lors du sinistre, dissimule ou soustrait tout ou partie des objets assurés, emploie sciemment comme justification des moyens frauduleux ou des documents mensongers, est déchu de tout droit à la garantie pour le sinistre en cause

ARTICLE 13 – EXPERTISE – SAUVETAGE

En cas de contestation sur les dommages, l'expertise amiable, sous réserve des droits respectifs des parties, est de rigueur.

L'Assureur et le Souscripteur choisissent chacun leur expert en vue de déterminer l'origine et le montant des dommages sur les bases du présent contrat. Si les experts ainsi désignés ne sont pas d'accord, ils s'adjoignent un troisième expert. Les trois experts opèrent en commun à la majorité des voix.

Faute par l'une des partie de nommer un expert ou pour les deux experts de s'entendre sur le choix du troisième, la désignation est effectuée par le Président du Tribunal compétent du lieu où le sinistre s'est produit. Cette nomination est faite sur simple requête signée des deux parties ou de l'une d'elles seulement, l'autre ayant été convoquée par lettre recommandée.

Chaque partie paie les frais et honoraires de son expert ; les honoraires du tiers expert et les frais de sa nomination, s'il y a lieu, sont supportés par moitié par chacune des parties.

L'Assuré ne peut faire aucun délaissement des objets garantis. Le sauvetage reste sa propriété, même en cas de contestation sur sa valeur.

Faute d'accord sur l'estimation, la vente amiable, ou la vente aux enchères du sauvetage sur le matériel et marchandises, chacune des parties peut demander, par simple requête au Président du Tribunal compétent du lieu du sinistre, la désignation d'un expert pour procéder à l'estimation du sauvetage.

ARTICLE 14 – ESTIMATION DES BIENS ASSURES.

L'assurance ne garantit à l'Assuré que la réparation de ses pertes réelles ou de celles dont il est responsable.

La somme assurée ne pouvant être considérée comme preuve ni de l'existence, ni de la valeur des objets sinistrés au moment du sinistre, l'Assuré est tenu d'en justifier par tous les moyens et documents en son pouvoir, ainsi que de l'importance du dommage.

Les bâtiments assurés, y compris les caves et fondations, abstraction faite de la valeur du sol, sont estimés d'après leur valeur réelle au prix de reconstruction au jour du sinistre, vétusté déduite. Toutefois, s'il s'agit de menues réparations, il n'est pas tenu compte de la vétusté.

En ce qui concerne les bâtiments construits sur le terrain d'autrui, l'indemnité en cas de reconstruction sur les lieux loués entreprise dans un délai d'un an à partir de la clôture de

l'expertise, est versée au fur et à mesure de l'exécution des travaux. En cas de non reconstruction, s'il résulte d'un acte ayant date certaine avant le sinistre que l'Assuré devait à une époque quelconque être remboursé par le propriétaire du sol de tout ou partie des constructions, l'indemnité ne peut excéder la somme stipulée au bail à cet effet ; à défaut de convention ou dans le silence de celle-ci l'Assuré n'a droit qu'à la valeur des matériaux évalués comme matériaux de démolition.

Les objets mobiliers et le matériel industriel assurés sont estimés d'après leur valeur de remplacement au jour du sinistre, vétusté déduite. Les matières premières, les denrées et les marchandises assurées sont évaluées au prix d'achat pour l'Assuré, calculé au dernier cours précédent le sinistre, ce prix étant majoré, s'il y a lieu, des frais de transport.

Les objets fabriqués ou en cours de fabrication sont estimés à leur prix de revient, c'est-à-dire au prix évalué comme à l'alinéa précédent, des matières premières et produits utilisés pour leur fabrication, majoré des frais de fabrication déjà faits et d'une part proportionnelle des frais généraux.

ARTICLE 15 – REGLE PROPORTIONNELLE.

En cas de sinistre survenu aux matériels, engins, installations et équipements de chantier et s'il résulte des estimations que la valeur des biens dépasse la somme garantie, l'Assuré est considéré comme son propre Assureur pour l'excédent et supporte en conséquence une part proportionnelle des dommages conformément à l'article 35 du Code des Assurances.

Toutefois, il ne sera pas fait application de la règle proportionnelle si l'excédent est inférieur à 10 %.

ARTICLE 16 – SAUVEGARDE DES DROITS DE L'ASSUREUR.

Aucune reconnaissance de responsabilité, aucune transaction intervenant en dehors de l'Assureur ne lui seront opposables.
Toutefois, n'est pas considéré comme une reconnaissance de responsabilité l'aveu d'un fait matériel, ni le seul fait d'avoir procuré à la victime un secours urgent, lorsqu'il s'agit d'un acte d'assistance que toute personne a le devoir légal ou moral d'accomplir.

Seul l'Assureur a le droit de transiger avec la personne lésée ou ses ayants droit dans la limite de sa garantie.

ARTICLE 17 – OBLIGATIONS DE L'ASSUREUR EN CAS DE SINISTRE.

Les frais de procès, de quittance et autres frais de règlement ne viennent pas en déduction du montant de la garantie.

Toutefois, en cas de condamnation à un montant supérieur à celui de la garantie souscrite, ils seront supportés par l'Assureur et l'Assuré dans la proportion de leur part respective dans la condamnation.

Si l'indemnité allouée à une victime ou à ses ayants droit consiste en une rente et si une acquisition de titres est ordonnée pour la sûreté de son paiement, l'Assureur emploie à la constitution de cette garantie la somme disponible dans les limites fixées par le contrat.

Si aucune garantie spéciale n'est ordonnée par une décision judiciaire, la valeur de la rente en capital est calculée d'après les règles applicables pour le calcul de la réserve mathématique de cette rente. Si cette valeur est inférieure ou égale à la somme disponible, la rente est intégralement à la charge de l'Assureur ; si elle lui est supérieure, la rente n'est à la charge de l'Assureur que proportionnellement à sa part dans la valeur de la rente en capital.

ARTICLE 18 - PROCEDURE.

En cas d'action mettant en cause une responsabilité assurée par le présent contrat, et dans la limite de sa garantie :

a) Devant les juridictions Civiles ou Administratives, l'Assureur assure la défense de l'Assuré, dirige le procès et conserve le libre exercice des voies de recours ;

b) Devant les juridictions pénales si la ou les victimes n'ont pas été désintéressées, l'Assureur a la faculté de diriger la défense des intérêts civils ou de s'y associer et, au nom de l'Assuré civilement responsable, d'exercer les voies de recours.
Toutefois, si l'Assuré a été cité comme prévenu, l'Assureur ne pourra exercer les voies de recours qu'avec l'accord de celui-ci, exception faite du pourvoi en cassation lorsqu'il est limité aux intérêts civils.

ARTICLE 19 – OBLIGATION DE L'ASSURE EN CAS DE RECUPERATION D'OBJETS VOLES.

En cas de vol, l'Assuré doit aviser immédiatement l'Assureur, par lettre recommandée, de la récupération de tout ou partie des objets disparus à quelque époque que ce soit.

Si les objets disparus sont récupérés en tout ou partie avant le paiement de l'indemnité, l'Assuré devra en prendre possession et l'Assureur ne sera tenu qu'au paiement des détériorations subies.

Si les objets sont récupérés après le paiement de l'indemnité, l'Assuré aura la faculté d'en reprendre la possession moyennant le remboursement de l'indemnité, sous déduction des détériorations éventuellement subies, à condition d'en faire la demande dans le délai d'un

mois à dater du jour où il aura été avisé de la récupération.

Dans tous les cas, l'Assuré sera indemnisé par l'Assureur des frais qu'il aura engagés raisonnablement en vue de la récupération.

ARTICLE 20 - PAIEMENT DES INDEMNITES.

Le paiement des indemnités est effectué au Siège Social de l'Assureur ou au Bureau de l'Agence où le contrat a été souscrit ou transféré, dans le délai de quinze jours à compter de l'accord des parties ou de la décision judiciaire devenue exécutoire. En cas d'opposition, ce délai ne court que du jour de la mainlevée.

ARTICLE 21 - SUBROGATION.

L'assureur est subrogé, conformément à l'article 42 du Code des Assurances, jusqu'à concurrence de l'indemnité payée par lui, dans les droits et actions de l'Assuré contre tous responsables du sinistre.

Si la subrogation, ne peut plus, par le fait de l'Assuré, s'opérer en faveur de l'Assureur, celui-ci est déchargé de sa garantie envers l'Assuré dans la mesure où aurait pu s'exercer la subrogation.

ARTICLE 22 - DISPOSITIONS DIVERSES.

A) L'Assuré est tenu, dans l'exécution des travaux, de respecter : les prescriptions administratives et techniques en vigueur et doit à tout moment, permettre aux représentants de l'Assureur de pénétrer sur le chantier et de procéder aux vérifications qu'il jugerait utiles.

B) Le Souscripteur s'engage à faire connaître à l'Assureur les dates auxquelles auront lieu les divers transferts de propriété.

C) En cas de litige entre les parties contractantes, les Tribunaux camerounais seront seuls compétents.

ARTICLE 23 - PRESCRIPTION.

Toute action dérivant du présent contrat est prescrite par deux ans à compter de l'événement qui y donne naissance, dans les conditions prévues aux articles 28 et 29 du Code des Assurances des Etats membres de la CIMA.

C-4 MATERIAL TRANSFERT AGREEMENT

This is an agreement made between the

RECIPIENT INSTITUTION NAME (hereafter "RECIPIENT"), the Institute of Microbiology, Immunology and Parasitology

RECIPIENT SCIENTISTS: 1. **Prof Dr Achim Hoerauf**

and the

PROVIDER INSTITUTION NAME (hereafter "PROVIDERS"), represented by the Department of Microbiology and Parasitology, Faculty of Science, University of Buea

PROVIDER SCIENTISTS: 1. **Prof Samuel Wanji**

INTRODUCTION

A. The Recipient and the Provider are collaborating on a research project, funded by the BMBF (German Ministry of Research and Education) entitled **"Tackling the obstacles of Lymphatic Filariasis and Podoconiosis-TAKeOFF"**

B. In order for the Project to take place, the Provider needs to provide the Recipient with the Materials. This Agreement sets out the terms on which the Provider will provide the Materials to the Recipient.

C. The Project is expected to benefit Cameroon in the following ways:

a) set up a lymphodema treatment centre in the district hospital of Bafut, a health district that is highly endemic for podoconiosis

b) Development of a new diagnostic tool to differentiate lymphodema due to Podoconiosis and Lymphodema due to Lymphatic Filariasis

c) Capacitating community health workers in the use SMS to identify and report to the health system on lymphodema cases due to Podoconiosis in their communities

d) To demonstrate the usefulness of the doxycycline treatment to alleviate the suffer of podoconiosis patients by improving their living conditions

Accepted by:

**Provider Scientist:**	**Recipient Scientist:**
**Signature**………………………..	**Signature**…………………………
**Printed Name:** Dr Njouendou Abdel	**Printed Name:** Dr Ute.Klarmann-Schulz
**Institution:** University of Buea	**Institution**: University of Bonn
**Provider Institution Approval**	**Recipient Institution Approval**
**Signature** ……………….….	Signature…………………….
**Printed Name**: Prof Samuel Wanji	Printed Name: Prof Achim Hoerauf
. **Date:** ………………….	Date: ……………………

C-5 DATA SHARING AGREEMENT

DATA SHARING AGREEMENT between

The Cameroon team Department of Microbiology and Parasitology, University of Buea

And the Institute of Microbiology, Immunmology and Parasitology, University Clinic Bonn

I. ENTITIES RECEIVING AND PROVIDING DATA

ENTITY RECEIVING DATA: CONTACT PERSON: **Achim Hoerauf** TITLE: **Professor**

ENTITY PROVIDING DATA: CONTACT PERSON : **Samuel Wanji**

TITLE: **Professor and Head of Department**

ADDRESS: Department of Microbiology and Parasitology, Faculty of Science, University of Buea, P.O. Box 63, Buea, Cameroon

II. PURPOSE, AUTHORITY AND TERM OF AGREEMENT

A. PURPOSE

To discover the potential resistance markers of O volvulus to ivermectin in the Cameroon population, of worms, the Research Foundation for Tropical Diseases and the Environment (REFTOTDE), Cameroon, Centre for Research on Filariasis and other Tropical Diseases (CRFilMT), Cameroon, McGill University, Canada and the Institut de Recherche pour le Développement (IRD), France are entering into an agreement which will allow the exchange of data and clarification of data access and utilization. The Cameroon team and IRD, will provide data collected to McGill University for analyses in the framework of resistance markers discovery and refining and development of a surveillance tool for onchocerciasis elimination.

B. PERIOD OF PERFORMANCE

This Agreement shall be effective when signed by both parties and shall continue until terminated pursuant to the termination clause contained herein.

III DESCRIPTION OF DATA/DATA WORKPLAN

The following data will be provided under this agreement: The Cameroon team will will provide data on the phenotypical chaterization of O. volvulus worm populations generated during the project and in a previously project aiming still at discovering resistance markers of O. volvulus to ivermectin in Cameroon. All data generated by this project shall be approved for dissemination by the National Ethics Committee and the ministry of Public Health of Cameroon, the Research Ethics Board of McGill University and the Ethics Review Committee of WHO.

IV. ACCESS TO DATA

A. METHOD OF ACCESS AND TRANSFER Data will be obtained in the following manner: the data will circulate between partners in a password-protected hard drive.

B. PERSONS HAVING ACCESS TO DATA All persons who will have access to data must complete a data privacy training through seminars and workshops.

C. FREQUENCY OF DATA EXCHANGE Data will be exchanged as needed to meet reporting requirements as well as on an ongoing basis between the Cameron team, IRD and McGill University for the entire length of the project.

V. SECURITY OF DATA

McGill University will not attempt to identify individuals' records by any method. Datasets containing protected health information (PHI) shall be encrypted. All reasonable precautions shall be taken to secure the data from individuals who do not specifically have authorized access. Data shall be kept on a password-protected file server located in a secure environment. Project data will be kept in a separate directory on the server which is also password-protected and will be accessible only by McGill University evaluators or staff members specifically authorized access as provided in this Agreement.

VI. CONFIDENTIALITY

A. REGULATIONS COVERING CONFIDENTIALITY OF DATA The use and disclosure of information obtained under this contract shall be subject to penalties identified in law. The Cameroon team, IRD and McGill University shall maintain the confidentiality of any information which may, in any manner, identify individual subjects.

As this project is specific to Cameroonians, all data collected will have the potential to identify Cameroonian tribes, elders, adults, youth, children, etc>. Confidentiality of all data therefore must be ensured.

B. NON-DISCLOSURE OF DATA The Cameroon team, IRD and McGill University shall not disclose, in whole or in part, the data described in this agreement to any individual or agency not specifically authorized by this agreement.

Data shall be provided on a timely basis. The Cameroon team, IRD and McGill University will document uses and users of the data and will report this information routinely back to the Principal Investigator.

C. The Cameroon team, IRD and McGill University will not disclose directly to, or use for the benefit of, any third party confidential information, knowledge or data acquired by virtue of its relationship with the other party named in this Agreement, without the prior written approval of the other party. It is understood and agreed by the parties that the obligations of this paragraph shall survive the expiration of termination of this Agreement.

VII. PAYMENT

Payment shall be done according to the clauses in the subcontract agreement (Decide

up on by the parties).

VIII. PROPERTY RIGHTS

Original materials prepared by either party, including, without limitation: reports, proposals, analysis, writings, sound recordings, pictorial reproductions or materials of any type whatsoever, are and shall remain the joint property of the research team. The Cameroon team, IRD and McGill University will assert no right, claim or interest of any nature whatsoever with respect thereto, including specifically but, without limitation, any claim to statutory copyright or patent.

Data Use and Ownership

The McGill University may request data, use approval from The Cameroon team and IRD for development of papers or reports.

The Cameroon team, IRD and McGill University shall be cited in reports, presentations, and scientific papers with respect to their contribution in data generation.

At least forty five (45) days prior to submission or presentation at a meeting/conference, any of party (submitting) will furnish a copy of a proposed publication or presentation to other parties for review, comments and approval. The other parties will have thirty (30) days after receipt of the proposed materials to request that submitting party removes confidential or sensitive information from the materials or rewrite the materials to protect confidential information from disclosure. The

materials may use, reuse and analyze, for teaching and research purposes, the data and findings as reviewed by and approved by the other parties.

IX. TERMINATION

Either party may terminate this Agreement upon 30 days prior written notification to the other party. If this Agreement is so terminated, the parties shall be liable only for performance rendered or costs incurred in accordance with the terms of this Agreement prior to the effective date of termination.

X. RIGHT OF INSPECTION

McGill University shall provide The Cameroon team and IRD the right of access to its facilities at all reasonable times, in order to monitor and evaluate performance, compliance, and/or quality assurance under this contract.

XI. ALL WRITINGS CONTAINED HEREIN

This Agreement contains all the terms and conditions agreed upon by the parties. No other understandings, oral or otherwise, regarding the subject matter of this Agreement shall be deemed to exist or to bind any of the parties hereto.

MCGILL UNIVERSITY, CANADA ________________________ Name/Title Date

________________________ Name/Title Date

THE CAMEROON TEAM (Research Foundation for Tropical Diseases and the Environment, Cameroon and Centre for Research on Filariasis and other Tropical Diseases (CRFilMT), Cameroon),

________________________ Name/Title Date

________________________ Name/Title Date

INSTITUT DE RECHERCHE POUR LE DEVELOPPEMENT (IRD), France

________________________ Name/Title Date

____

C-6 FRAMEWORK CONVENTION

**REPUBLIQUE DU CAMEROUN**	**REPUBLIC OF CAMEROON**
Paix-Travail-Patrie	*Peace-Work-Fatherland*

001 [illegible]

FRAMEWORK CONVENTION

Between

THE MINISTRY OF PUBLIC HEALTH

And

THE UNIVERSITY OF BUEA

For the implementation of the University of Buea "TAKEOFF" research project in the Cameroon Health System, sponsored by the German Ministry of Research.

Between the undersigned

The Ministry of Public Health

Hereafter referred to as "MOH"

On the one hand,

And the University of Buea,

Hereafter referred to as "UB"

On the other hand,

Collectively referred to as the Parties.

Considering the promotion of collaboration between the public and private sectors through Law No. 96/003 of January 1996 on the Framework Law in Health, to ensure quality care;

Mindful of Presidential Decree No. 95/013 of 7 February 1995 to organize basic health services in health districts;

Mindful of the 2016-2027 Health Sector Strategy, basis for the health sector reform and the Health Sector Partnership Strategy in the development of the contract approach in the form of Conventions, Execution Contracts and others, as the case may be;

It was agreed and decided as follows:

2

Article 1.-PURPOSE

The purpose of this Framework Convention shall be to implement the University of Buea "TAKEOFF" research project in the Cameroon Health System, sponsored by the German Ministry of Research.

Article 2.-DURATION

This Framework Convention shall have a term of five years renewable by mutual agreement.

Article 3.-SCOPE

For the purposes of this Framework Convention, "partnership" shall refer to any activity defined or carried out by mutual agreement between the Ministry of Public Health and the University of Buea in the interests of both Parties.

This Framework Convention shall include the following:

(1) The implementation of the University of Buea "TAKEOFF" research project in the Cameroon Health System, sponsored by the Research Networks for Health Innovations in Sub-Saharan Africa with funding from the German Ministry of Education and Research.

(2) The establishment of a management unit for lymphoedema due to pocodoniosis at the Bafut District Hospital in the North West Region of Cameroon where this pathology is highly endemic.

Article 4.-COMMITMENTS OF THE PARTIES

(1) General Provisions:

- Continue regular exchanges as part of their respective missions;
- Increase exchanges between the staff of both Parties in order to find solutions to the problems of both Parties;
- Make every effort to ensure the financing of activities resulting from this partnership.

(2) Special provisions

The Ministry of Public Heal shall undertake to:

- issue the Administrative Authorisation for Research;

3

- make available to the team of researchers from the University of Buea, the health system platform (infrastructure and personnel) necessary to carry out the research project.

The University of Buea shall undertake to:

- request ethics clearance from the National Ethics Committee for Research in Human Health;
- request the Administrative Authorisation for Research;
- use resources from the Research Networks for Health Innovations in Sub-Saharan Africa and made available by the German Ministry of Education and Research as part of the "TAKEOFF" research project;
- improve the infrastructure and technical level of the Bafut District Hospital for the management of lymphoedema related to podoconiosis;
- train the health personnel on the management of lymphoedema related to podoconiosis;
- train community health workers on the use of mobile phones to detect and refer cases of podoconiosis to the Management Centre of the Bafut District Hospital.

Article 5.-MONITORING COMMITTEE

(1) The Monitoring Committee shall be responsible for implementing this Framework Convention. To this effect, it shall:
- review activity and evaluation reports;
- formulate opinions on strategic orientations;
- discuss and approve annual work plans and activity reports;
- discuss the main problems encountered and anticipate solutions;
- make any proposal for the proper execution of the Convention;
- send its reports to the Ministry of Public Health.

(2) The Committee may invite any other person based on their competencies.

(3) The Monitoring Committee shall include the following:

Chairperson: The Director of Diseases, Epidemics and Pandemics Control, or his Representative;

Vice-Chairperson: The Vice-Chancellor of the University of Buea;

Members:

- One representative of the Cooperation Division;
- One representative of the National Ethics Committee for Research in Human Health;
- One representative of the Division of Operational Research in Health;
- Two representatives of the University of Buea;
- One representative of the German Ministry of Education and Research;
- The Bafut District Medical Officer.

Article 6.-PAYMENT TERMS

A financial support of FCFA twenty-nine million, five hundred and twenty thousand (29 520 000), that is € 45 000, from the budget of the TAKEOFF research project, shall be used to improve the infrastructure and technical platform of the Bafut District Hospital which will host the Centre for the management of lymphoedema related to podoconiosis (rehabilitation of the building, office suplies, minor surgery equipment and plaster, patient beds, Internet connection).

Article 7.-AMENDMENTS

Amendments may be made to this Framework Convention. The Party initiating the amendment shall notify the other Party through recommended letter with acknowledgement of receipt.

Article 8.-APPLICABLE LAW

This Framework Convention shall be governed by the Cameroonian Law, and disputes arising from the interpretation or execution thereof shall be settled through arbitration and, where necessary, before the competent courts.

Article 9.-DENUNCIATION OR TERMINATION

In the event of non-respect of the commitments of one or the other party, this Framework Convention may be denounced, and the requesting Party shall notify the other Party through recommended letter with acknowledgement of receipt.

The MOH and the UB shall reserve the right to terminate this Framework Convention in the event where either of the co-contracting Parties no longer respect any of their commitments.

REPUBLIC OF CAMEROON République du Cameroun UNIVERSITY OF BUEA Université de Buéa Le Recteur VICE-CHANCELLOR Ministère de l'Enseignement Supérieur MINISTRY OF HIGHER EDUCATION

Article 10.-LANGUAGES

The original copies of this Framework Convention shall be drafted and signed in English and French.

Article 11.-DOMICILIATION OF THE PARTIES

For the purposes of this Framework Convention, the Parties shall use the following addresses:

- **For the Ministry of Public Health**
  In Yaounde,
  Tel : (237)
  Fax : (237)
  Website : www.minsante.gov.cm

- **For the University of Buea**
  P.O. Box: 63 Buea South West Region
  Tel : (237) 233 32 21 34/ 233 32 26 90/ 233 32 27 06
  Fax : (237) 233 32 22 72
  Website : www.ubuea.cm

Article 12.-ENTRY INTO FORCE

This Framework Convention shall enter into force upon the date of signature by both Parties.

Done in four original copies

In Yaounde, 18 JAN 2018

**For the University of Buea**	**For the Ministry of Public Health**
**The Vice-Chancellor**	**The Minister**
**Mr. NGOMO Horace MANGA**	**Mr. André MAMA FOUDA**

6

C-7 RECEIPT OF PAYEMENT OF SUBMISSION FEES

SCB Cameroun

BORDEREAU DE VERSEMENT ESPECES DEPLACE Réf : 00063-118-020435-181217

Le 18 Decembre 2017 à 14:24

Client

COMITE NATIONAL ETHIQUE
RECHE
Adresse YAOUNDE
BP
Tel 699767427

Opération

Agence de la transaction 00063-BUEA
Devise FRANC CFA
Compte 97662023130-57 **Agence** 00001-CA YDE VOGT
Caisse CAISSE ESPECES XAF 003
Montant du versement 200 000 XAF

Origine des fonds ethic clearance

Billetage

Valeur	Nombre	Montant
10 000	20	200 000

Remettant NCHAWA NJIKI

**Montant net**	200 000 XAF
**Commission**	0 XAF
**TVA**	0 XAF

Imp. Montparnasse Tél/Fax : 222 22 01 65 Ydé

Nous portons au crédit du compte N° 97662023130-57 la somme de 200 000 XAF soit **deux cents mille Francs CFA** en date de valeur du 19/12/2017

Client	Guichetier

SOCIETE COMMERCIALE DE BANQUE CAMEROUN GROUPE ATTIJARIWAFA BANK S.A. avec Conseil d'Administration au capital de 10.54 Milliards de FCFA
RCCM N° 89.S.152 - RC/YAO/2007M/391 - RC/YAO/2011/M/92
Direction générale: 530, Rue du Roi George - B.P. 300 DOUALA (Cameroun) - Tél. : (237) 333 43 54 00 - Fax : (237) 333 43 54 13

Réf. SCB 132

C-8 AUTHORIZATION FOR STUDY SITE

REPUBLIQUE DU CAMEROUN<br>Paix-Travail-Patrie<br>----------------<br>MINISTERE DE LA SANTE PUBLIQUE<br>-----------------<br>DELEGATION REGIONALE DE LA SANTE PUBLIQUE DU NORD OUEST<br>----------------<br>SERVICE DE DISTRICT DE SANTE DE BAFUT<br>------------------	REPUBLIC OF CAMEROON<br>Peace-Work-Fatherland<br>----------------<br>MINISTRY OF PUBLIC HEALTH<br>-----------------<br>REGIONAL DELEGATION OF PUBLIC HEALTH NORTH WEST REGION<br>-------------------<br>BAFUT HEALTH DISTRICT SERVICES<br>--------------------

N° 18 /L/MPH/RDPH/BHD/DMO/18 Bafut,the 24 JAN 2018

AUTHORIZATION TO CARRY OUT RESEARCH

I the undersigned, District Medical Officer for Bafut health District hereby give approval to carry out research,in the Bafut Health District title:**TAKeOFF – LEDoxy: Doxycycline 200mg/d versus 100mg/d for 6 weeks to improve filarial lymphedema (LE) - a multinational, randomized, placebo-controlled trial,** by the TAKeOFF research team of the University of Buea.

This study which aims to show the efficacy of a 6-week course of daily doxycycline 200 mg in reversing or altering the progression of lymphedema due to podoconiosis, was presentedto us by the Principal Investigator, ProfessorWanji Samuel of the University of Buea, and we found it relevant as a research trial for the benefit of the local population.
It is a two-year duration project as from January 2018 to 2020, thatinvestigates the effect of an antibiotic, doxycycline, in improving lymphedema. It also investigates the potential of this treatment to treat those who suffer from lymphedema. By participating in the study, the population of the Bafuthealth district which is highly concerned by lymphedema will benefit from free thorough medical evaluations as well as receiving training and supplies for local care and management of lymphedema that by itself should improve health while reinforcing monitoring system andthe district hospital's infrastructures.

Given the collaboration needed to collect information necessary for this study; considering the strict respect of the protocol validated by the National Ethics Committee.

This authorization is issued to serve the purpose for which it is intended.

District Medical Officer
BafutHealth District

P.O.

Dr. Njoh P. Yves
MEDICAL DOCTOR
B.Sc, MD (HONS), MPHM (HONS)
