# Supplementary material for: The design and development of a multicentric protocol to investigate the impact of adjunctive doxycycline on the management of peripheral lymphoedema caused by lymphatic filariasis and podoconiosis
Source: Parasit Vectors. 2020 Mar 30;13:155. doi: 10.1186/s13071-020-04024-2 (PMC7106687; doi:10.1186/s13071-020-04024-2)
Supplement: Supplementary file 4 — Additional file 4: Table S1. Study schedule. [file 13071_2020_4024_MOESM4_ESM.docx]

**Additional file 4: Table S1: Study schedule**

**Enrolment and Treatment**

| **SCHEDULE OF ACTIVITIES** |  |  |  |  |  |  |  |
| --- | --- | --- | --- | --- | --- | --- | --- |
|  | **Visit 1**  **Screening** | **Visit 2**  **Baseline^a^** |  |  |  |  |  |
|  |  |  | **Day 1** | **Day 2-21** | **Day 22** | **Day 23-41** | **End of Treatment Day 42** |
| Informed Consents/ Assents (clinical trial and biobanking) | √ |  |  |  |  |  |  |
| Demographic data | √ |  |  |  |  |  |  |
| Lymphoedema staging | √ | √ |  |  |  |  |  |
| Clinical photographs |  | √ |  |  |  |  |  |
| History of lymphoedema | √ |  |  |  |  |  |  |
| History of ADLA | √ |  |  |  |  |  |  |
| Circumference - Tape |  | √ |  |  |  |  |  |
| Circumference - Lymphatech® |  | √ |  |  |  |  |  |
| Volume of LE - Lymphatech® |  | √ |  |  |  |  |  |
| Ultrasound |  | √ |  |  |  |  |  |
| Medical history | √ |  |  |  |  |  |  |
| Concomitant medication | √ | √ | √ | √ | √ | √ | √ |
| History of relevant medications | √ |  |  |  |  |  |  |
| Vital signs | √ | √ |  |  |  |  |  |
| Physical examination | √ | √ |  |  |  |  |  |
| Quality of Life Questionnaire |  | √ |  |  |  |  |  |
| Hygiene Evaluation |  | √ |  |  |  |  |  |
| Lymphoedema management training |  | √ |  |  |  |  |  |
| Laboratory assessment (blood) | √ | *(√)^a^* | *(√)^b^* |  | √ (before treatment on d 22, AST/ALT/γ-GT, range +2 days) |  | √ (on the last day of treatment, AST/ALT/γ-GT, range +2 days) |
| Urine sample collected | √ | √ | √ | √ |  | √ | √ |
| Saliva sample collected | √ |  |  |  |  |  |  |
| Pregnancy test | √ | √ | *(√)^b^* | √ (on day 15, range + 2 days) |  | √ (on day 29, range + 2 days) | √ (on day 42, range + 2 days) |
| In-/Exclusion criteria | √ | √ | *(√)^b^* |  |  |  |  |
| Randomization |  | √ |  |  |  |  |  |
| Presence for visit |  |  |  | √ | √ | √ | √ |
| Individual treatment |  |  | √ | √ | √ | √ | √ |
| ADLA questionnaire |  | √ | √ | √ | √ | √ | √ |
| AEs |  |  |  | √ | √ | √ | √ |
| End of study record |  |  |  |  |  |  |  |

^a^ Visit 2 (Baseline) should take place max. 28 days after visit 1 (Screening). If that is not the case, blood tests have to be repeated. ^b^Visit 3 (Treatment) should start on the same day or one day after visit 2 (Baseline). If that is not the case, the pregnancy test has to be repeated and in case the period between the two visits is > 28 days also blood tests and check of in- and exclusion criteria have to be repeated.

**Follow-up: end of treatment to study completion**

|  | **Visit 4**  **2 month follow-up^a^** | **Visit 5**  **4 month follow-up^b^** | **Visit 6**  **6 month follow-up^c^** | **Visit 7**  **8 month follow-up^d^** | **Visit 8**  **10 month follow-up^e^** | **Visit 9**  **12 month follow-up^f^** | **Visit 10**  **14 month follow-up^g^** | **Visit 11**  **16 month follow-up^h^** | **Visit 12**  **18 month follow-up^i^** | **Visit 13**  **20 month follow-up^j^** | **Visit 14**  **22 month follow-up^k^** | **Visit 15**  **24 month follow-up^l^** |
| --- | --- | --- | --- | --- | --- | --- | --- | --- | --- | --- | --- | --- |
| Lymphoedema staging |  |  | √ |  |  | √ |  |  | √ |  |  | √ |
| Clinical photographs |  |  | √ |  |  | √ |  |  | √ |  |  | √ |
| Circumference - Tape |  |  | √ |  |  | √ |  |  |  |  |  | √ |
| Circumference - Lymphatech® |  |  | √ |  |  | √ |  |  |  |  |  | √ |
| Volume of LE - Lymphatech® |  |  | √ |  |  | √ |  |  |  |  |  | √ |
| Ultrasound (LEDoxy only) |  |  | √ |  |  | √ |  |  |  |  |  | √ |
| Concomitant medication | √ | √ | √ | √ | √ | √ | √ | √ | √ | √ | √ | √ |
| Vital signs |  |  | √ |  |  | √ |  |  |  |  |  | √ |
| Quality of Life Questionnaire |  |  |  |  |  | √ |  |  |  |  |  | √ |
| Hygiene evaluation |  | √ | √ |  |  | √ |  |  | √ |  |  | √ |
| Lymphoedema management training |  | √ | √ |  |  | √ |  |  | √ |  |  | √ |
| Laboratory assessment (blood) |  |  | √ |  |  | √ |  |  |  |  |  | √ |
| Urine sample collected |  |  | √ |  |  | √ |  |  |  |  |  | √ |
| Saliva sample collected (TAKEoff only) |  |  | √ |  |  | √ |  |  |  |  |  | √ |
| Pregnancy test | √ |  | √ |  |  | √ |  |  |  |  |  | √ |
| Presence for visit | √ | √ | √ | √ | √ | √ | √ | √ | √ | √ | √ | √ |
| ADLA questionnaire | √ | √ | √ | √ | √ | √ | √ | √ | √ | √ | √ | √ |
| AEs | √ | √ |  |  |  |  |  |  |  |  |  |  |
| End of study record |  |  |  |  |  |  |  |  |  |  |  | √ |

^a^ the 2 months follow-up should take place 61 days (± 7 days) after treatment day 1 ^b^ the 4 months follow-up should take place 121 days (± 10 days) after treatment day 1

^c^ the 6 months follow-up should take place 182 days (± 21 days) after treatment day 1 ^d^ the 8 months follow-up should take place 243 days (± 14 days) after treatment day 1

^e^ the 10 months follow-up should take place 303 days (± 14 days) after treatment day 1 ^f^ the 12 months follow-up should take place 364 days (± 21 days) after treatment day 1

^g^ the 14 months follow-up should take place 425 days (± 21 days) after treatment day 1 ^h^ the 16 months follow-up should take place 485 days (± 21 days) after treatment day 1

^i^ the 18 months follow-up should take place 546 days (± 21 days) after treatment day 1 ^j^ the 20 months follow-up should take place 607 days (± 21 days) after treatment day 1

^k^ the 22 months follow-up should take place 667 days (± 21 days) after treatment day 1 ^l^ the 24 months follow-up should take place 728 days (- 28 days; + 56 days) after treatment day 1
